# Supplementary material for: Identification of stable reference genes for quantitative PCR in koalas
Source: Sci Rep. 2018 Feb 20;8:3364. doi: 10.1038/s41598-018-21723-0 (PMC5820254; doi:10.1038/s41598-018-21723-0)
Supplement: Supplementary file 1 — Supplementary information [file 41598_2018_21723_MOESM1_ESM.pdf]

Supplementary Information

Title: Identification of stable reference genes for quantitative PCR in koalas

N. Sarker[1], J. Fabijan[2], R.D. Emes[3,4], F. Hemmatzadeh[2], J. Meers[1], J. Moreton[4], H. Owen[1], J.M. Seddon[1], G. Simmons[1], N. Speight[2], D. Trott[2], L. Woolford[2], and R.E Tarlinton[3]*

1. School of Veterinary Science, The University of Queensland, Gatton, QLD, Australia
2. School of Animal and Veterinary Sciences, The University of Adelaide, Roseworthy, SA, Australia
3. School of Veterinary Medicine and Science, University of Nottingham, Sutton Bonington Campus, Loughborough, LE12 5RD, United Kingdom
4. Advanced Data Analysis Centre (ADAC), University of Nottingham, Sutton Bonington, United Kingdom

*Corresponding author:

Dr Rachael Tarlinton

Email: rachael.tarlinton@nottingham.ac.uk

Supplementary Table S1: Demographic information of koalas used in this study

ID	Zone	Sex	Body weight	Tooth wear (Martin)	Body Score (1-5)	Clinical condition (based on pathological examination)	RNA value (A260/A280)	RNA value (A260/A230)
Q1	Queensland	F	5	4	4	Liver mass	2.1	2.21
Q2		F	4.8	4	3	Urogenital Chlamydiosis	2.04	2.12
Q3		M	5.1	4	1.5	Severe pyogranulomatous nephritis; mild choroiditis	2.06	2.14
Q4		M	6.4	4	2.5	Ocular Chlamydiosis	2.09	2.23
Q5		F	5	4	0	Urogenital Chlamydiosis	2.14	2.29
Q6		M	7.7	5	1	Ocular Chlamydiosis	2.11	2.31
Q7		M	6.8	4	3.5	Ocular Chlamydiosis	2.01	2.14
Q8		M	4.9	4	0.5	Multifocal fibrosis	2	2.11
Q9		M	6	4	0	Myocardial fibrosis	2.17	2.23
Q10		M	7.6	4	2	Chronic keratitis	2.13	2.24
Q11		M	7	4	0.5	Pleural fibrosis	2.04	2.17
S1	South Australia	M	7.4	5	3	Osteomyelitis; Oxolate nephrosis	2.11	2.23
S2		M	7	2	3	Osteomyelitis	2.09	2.21
S3		F	4.7	4	2	Oxalate nephrosis	2.05	2.17
S4		M	7.8	2	3	Gastrointestinal perforation	2.14	2.26
S5		M	8	4	3	Ocular Chlamydiosis	2.01	2.14
S6		F	3.6	1	5	Scoliosis and kyphosis, pneumonia	2.11	2.25
S7		M	7.27	2	3	Oxalate nephrosis	2.05	2.11
S8		F	4.46	3	2	Oxalate nephrosis	2.04	2.16

Supplementary Table S2: Descriptive statistics of RT-qPCR CT values of the analysed genes (a) Queensland population (b) South Australia population and (c) Combination of all samples from both populations

(a)

	Candidate Reference Gene												
	**ACTB**	**Smap2**	**Sec22b**	**Bet1**	**Grk2**	**Hmg20a**	**Nckap1l**	**Ndufaf3**	**Pdap1**	**Stx12**	**Chchd2**	**Tmem97**	**GAPDH**
Minimum	13.67	27.8	27.85	19.36	20.94	21.27	20.48	21.62	22.38	20.91	23.63	23.19	24.2
25% Percentile	13.77	28.29	28.45	20.26	21.52	22.2	20.54	22.62	23.24	21.21	24.15	23.48	24.7
Median	14.12	30.18	28.98	20.69	22.18	22.7	20.73	22.94	23.57	21.6	24.56	23.73	25.34
75% Percentile	14.87	30.95	30.35	21.19	22.58	22.85	21.47	23.32	25.2	22.25	25.24	24.69	25.55
Maximum	15.85	33.25	30.8	21.63	24.08	24.32	23.75	24.76	27.74	23.33	27.27	25.99	26.44
Mean	14.29	29.95	29.26	20.65	22.23	22.69	21.18	23.09	24.17	21.75	24.92	24.12	25.25
Std. Deviation	0.6865	1.668	1.015	0.6311	0.9222	0.8309	0.953	0.882	1.591	0.7312	1.167	0.8813	0.6845
Std. Error of Mean	0.207	0.5029	0.3059	0.1903	0.2781	0.2505	0.2873	0.2659	0.4797	0.2205	0.352	0.2657	0.2064
Lower 95% CI of mean	13.83	28.83	28.57	20.23	21.61	22.14	20.54	22.5	23.1	21.26	24.13	23.52	24.79
Upper 95% CI of mean	14.75	31.07	29.94	21.08	22.85	23.25	21.82	23.69	25.24	22.24	25.7	24.71	25.71
Geometric mean	14.28	29.9	29.24	20.64	22.21	22.68	21.16	23.08	24.12	21.74	24.89	24.1	25.24
Geometric SD factor	1.048	1.057	1.035	1.031	1.042	1.037	1.044	1.039	1.066	1.034	1.047	1.037	1.027

(b)

	Candidate Reference Gene												
	**ACTB**	**Smap2**	**Sec22b**	**Bet1**	**Grk2**	**Hmg20a**	**Nckap1l**	**Ndufaf3**	**Pdap1**	**Stx12**	**Chchd2**	**Tmem97**	**GAPDH**
Minimum	13.17	28.62	27.54	20.5	20.65	21.43	20.5	22.24	22.55	21.16	24.09	23.02	24.37
25% Percentile	13.86	29.14	28.04	20.86	21.74	22.31	20.59	22.42	23.25	21.46	24.15	23.53	24.83
Median	14.53	31.04	29	21.41	22.74	23.2	22.04	23.44	24.3	21.96	24.87	24.26	25.66
75% Percentile	15.21	31.59	30.6	22.11	23.03	23.55	22.22	24.06	26	23.21	26.23	24.67	26.37
Maximum	15.37	32.82	31.28	22.35	23.89	24.42	23.02	24.62	27.11	24.13	28.22	26.94	26.99
Mean	14.48	30.72	29.24	21.43	22.46	23.01	21.65	23.36	24.53	22.3	25.36	24.38	25.63
Std. Deviation	0.7836	1.449	1.414	0.6827	1.008	0.9183	0.937	0.8611	1.597	1.03	1.45	1.183	0.8844
Std. Error of Mean	0.277	0.5122	0.4999	0.2414	0.3565	0.3247	0.3313	0.3044	0.5646	0.3643	0.5125	0.4181	0.3127
Lower 95% CI of mean	13.83	29.51	28.06	20.85	21.62	22.24	20.87	22.64	23.19	21.44	24.14	23.39	24.89
Upper 95% CI of mean	15.14	31.93	30.42	22	23.31	23.78	22.43	24.08	25.86	23.16	26.57	25.37	26.37
Geometric mean	14.46	30.69	29.21	21.42	22.44	22.99	21.63	23.35	24.48	22.28	25.32	24.36	25.62
Geometric SD factor	1.056	1.049	1.049	1.032	1.046	1.041	1.044	1.038	1.067	1.047	1.057	1.048	1.035

(c)

	Candidate Reference Gene												
	**ACTB**	**Smap2**	**Sec22b**	**Bet1**	**Grk2**	**Hmg20a**	**Nckap1l**	**Ndufaf3**	**Pdap1**	**Stx12**	**Chchd2**	**Tmem97**	**GAPDH**
Minimum	13.17	27.8	27.54	19.36	20.65	21.27	20.48	21.62	22.38	20.91	23.63	23.02	24.2
25% Percentile	13.77	28.62	28.16	20.5	21.65	22.23	20.54	22.62	23.24	21.37	24.15	23.48	24.75
Median	14.22	30.63	28.98	20.93	22.49	22.77	21.3	23.08	23.81	21.73	24.56	23.87	25.46
75% Percentile	14.99	31.24	30.35	21.63	23.02	23.48	22.07	23.9	25.2	22.61	26.14	24.69	26.11
Maximum	15.85	33.25	31.28	22.35	24.08	24.42	23.75	24.76	27.74	24.13	28.22	26.94	26.99
Mean	14.37	30.27	29.25	20.98	22.33	22.83	21.38	23.21	24.32	21.98	25.1	24.23	25.41
Std. Deviation	0.714	1.587	1.162	0.7457	0.9392	0.8586	0.9502	0.8597	1.559	0.8869	1.274	0.9966	0.7757
Std. Error of Mean	0.1638	0.364	0.2665	0.1711	0.2155	0.197	0.218	0.1972	0.3577	0.2035	0.2924	0.2286	0.178
Lower 95% CI of mean	14.03	29.51	28.69	20.62	21.88	22.41	20.92	22.79	23.57	21.55	24.49	23.75	25.04
Upper 95% CI of mean	14.72	31.04	29.81	21.34	22.78	23.24	21.84	23.62	25.07	22.41	25.72	24.71	25.79
Geometric mean	14.35	30.23	29.23	20.97	22.31	22.81	21.36	23.19	24.27	21.97	25.07	24.21	25.4
Geometric SD factor	1.051	1.054	1.04	1.036	1.043	1.038	1.045	1.038	1.065	1.04	1.051	1.041	1.031

Supplementary Table S3: Overall ranking of candidate reference genes analysed separately in Queensland, South Australia and both populations combined

		Ranking order												
**Queensland**	**Method**	1	2	3	4	5	6	7	8	9	10	11	12	13
	**Delta CT**	Tmem97	Stx12	Hmg20a	ACTB	GAPDH	Nckap1l	Ndufaf3	Grk2	Sec22b	Chchd2	Bet1	Smap	Pdap1
	**BestKeeper**	Bet1	ACTB	Stx12	GAPDH	Hmg20a	Ndufaf3	Nckap1l	Tmem97	Grk2	Sec22b	Chchd2	Pdap1	Smap
	**Normfinder**	Tmem97	Stx12	ACTB	Nckap1l	Hmg20a	GAPDH	Grk2	Ndufaf3	Sec22b	Chchd2	Bet1	Smap	Pdap1
	**Genorm**	Hmg20a | Ndufaf3		GAPDH	Grk2	Nckap1l	ACTB	Tmem97	Stx12	Sec22b	Chchd2	Bet1	Smap	Pdap1
	**Recommended comprehensive ranking**	Tmem97	Hmg20a	Stx12	ACTB	Ndufaf3	GAPDH	Nckap1l	Bet1	Grk2	Sec22b	Chchd2	Smap	Pdap1
		Ranking order												
**South Australia**	**Method**	1	2	3	4	5	6	7	8	9	10	11	12	13
	**Delta CT**	Nckap1l	Tmem97	Ndufaf3	Hmg20a	Stx12	GAPDH	Grk2	ACTB	Chchd2	Bet1	Smap	Sec22b	Pdap1
	**BestKeeper**	Bet1	ACTB	Ndufaf3	Hmg20a	GAPDH	Tmem97	Grk2	Nckap1l	Stx12	Smap	Chchd2	Sec22b	Pdap1
	**Normfinder**	Tmem97	Stx12	Nckap1l	Ndufaf3	ACTB	Hmg20a	GAPDH	Chchd2	Grk2	Bet1	Smap	Sec22b	Pdap1
	**Genorm**	Grk2 | Hmg20a		GAPDH	Ndufaf3	Nckap1l	Stx12	Tmem97	Chchd2	ACTB	Bet1	Smap	Sec22b	Pdap1
	**Recommended comprehensive ranking**	Tmem97	Hmg20a	Nckap1l	Ndufaf3	Grk2	Stx12	GAPDH	ACTB	Bet1	Chchd2	Smap	Sec22b	Pdap1

		Ranking order												
**Combination of Both populations**	**Method**	1	2	3	4	5	6	7	8	9	10	11	12	13
	**Delta CT**	Tmem97	Stx12	Nckap1l	Hmg20a	Ndufaf3	GAPDH	ACTB	Grk2	Chchd2	Bet1	Sec22b	Smap	Pdap1
	**BestKeeper**	Bet1	ACTB	GAPDH	Hmg20a	Ndufaf3	Stx12	Tmem97	Nckap1l	Grk2	Sec22b	Chchd2	Smap	Pdap1
	**Normfinder**	Tmem97	Stx12	Nckap1l	ACTB	Hmg20a	Ndufaf3	GAPDH	Grk2	Chchd2	Bet1	Sec22b	Smap	Pdap1
	**Genorm**	Hmg20a | Ndufaf3		GAPDH	Grk2	Nckap1l	Tmem97	Stx12	ACTB	Chchd2	Sec22b	Bet1	Smap	Pdap1
	**Recommended comprehensive ranking**	Tmem97	Hmg20a	Ndufaf3	Stx12	Nckap1l	GAPDH	ACTB	Bet1	Grk2	Chchd2	Sec22b	Smap	Pdap1

Supplementary Figure S1: Agarose gel electrophoresis view of corresponding reference genes (negative contrast); 100 bp ladder (bioline) used as a DNA marker. L1 = *ACTB*, L2 = *Stx12*, L3 = *Chchd2*, L4 = *Tmem97*, L5 = *Bet1*, L6 = *Ndufaf3*, L7 = *Nckap1l*, L8 = *Grk2*, L9 = *Hmg20a*, L10 = *Pdap1*, L11 = *Smap2*, L12 = *Sec22b*, L13 = *GAPDH* and L14= 100bp ladder

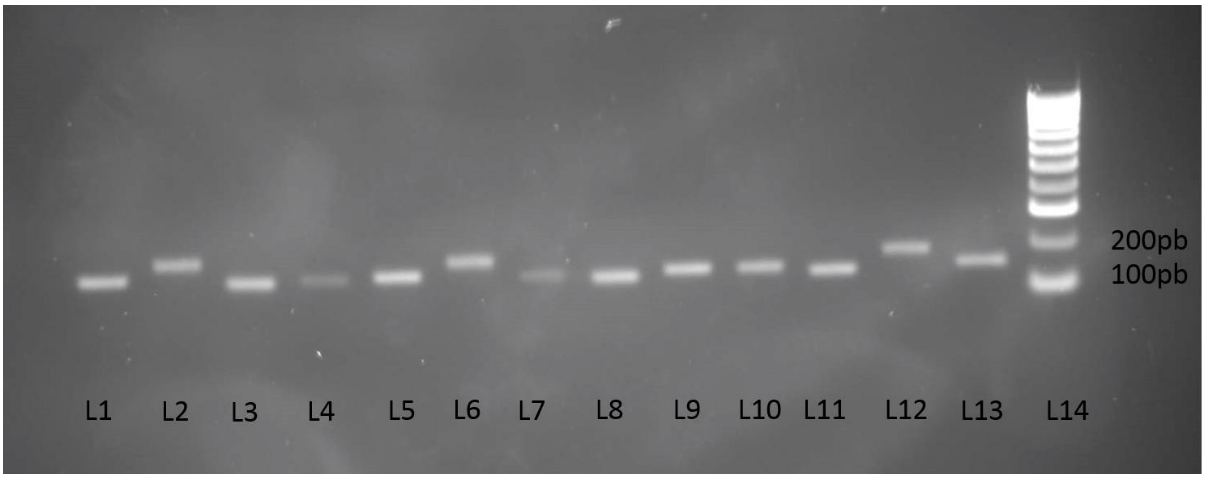

Supplementary Figure S2: Melt-curve profiles of the respective reference genes in Sybr green qPCR.

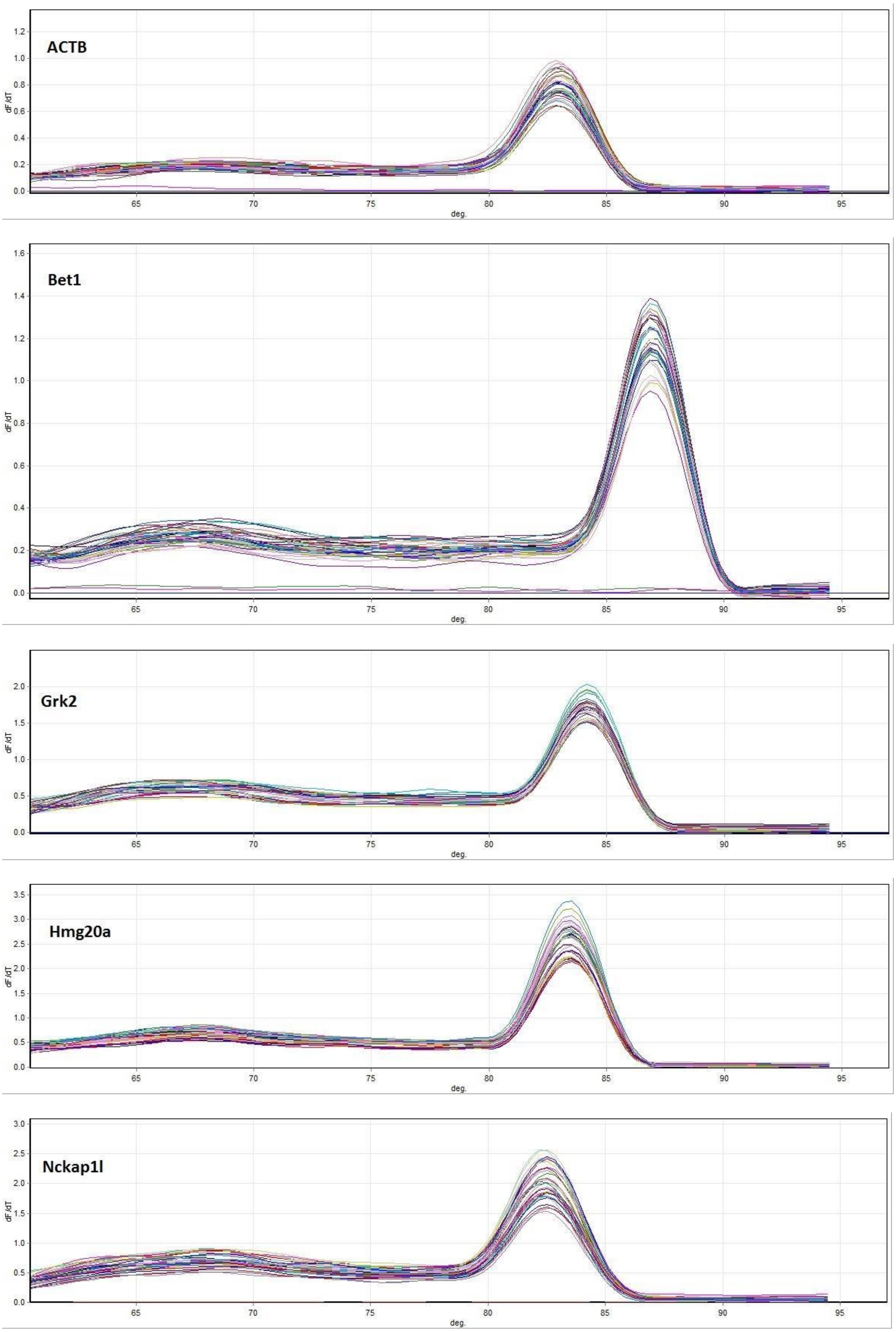

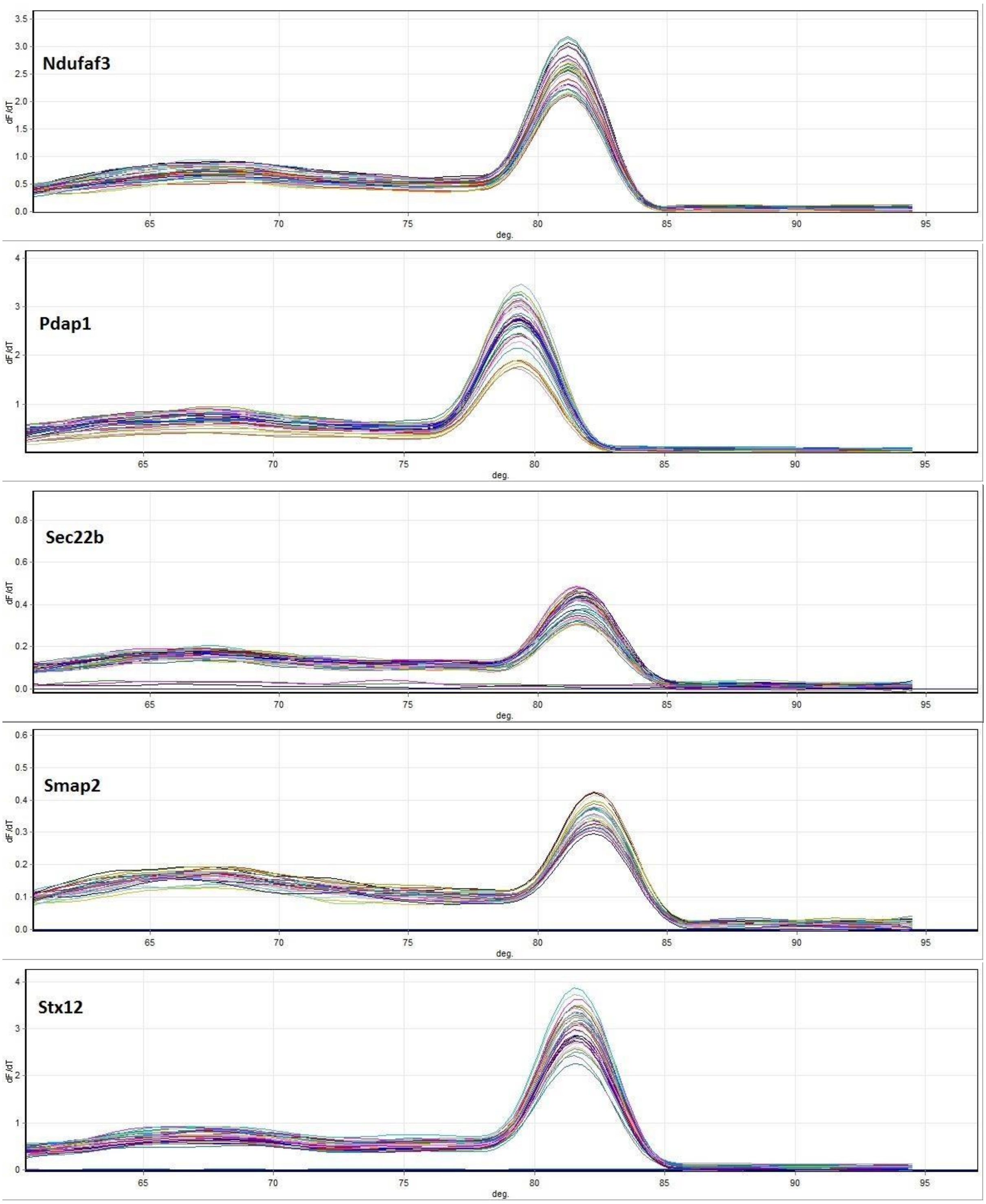
Ndufaf3
dF/dT
deg.
Pdap1
dF/dT
deg.
Sec22b
dF/dT
deg.
Smap2
dF/dT
deg.
Stx12
dF/dT
deg.

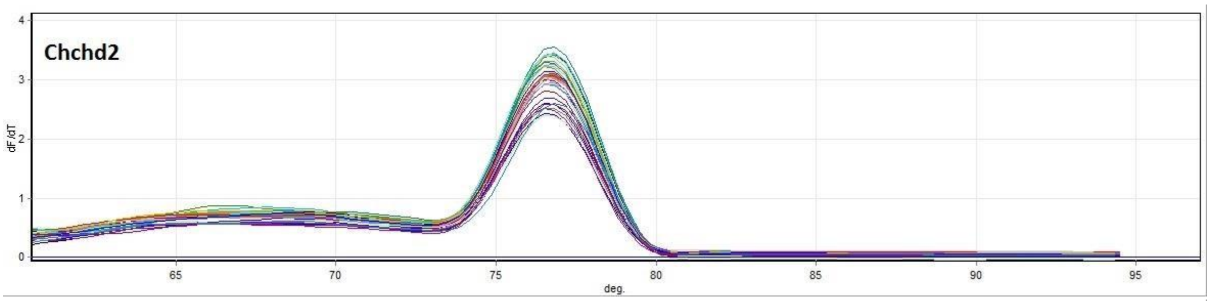
Chchd2
dF/dT
0
1
2
3
4
65
70
75
80
85
90
95
deg.

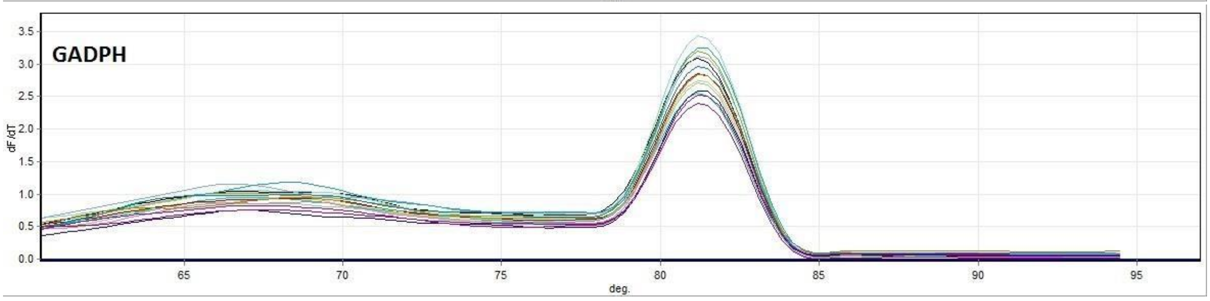
GADPH
dF/dT
0.0
0.5
1.0
1.5
2.0
2.5
3.0
3.5
65
70
75
80
85
90
95
deg.

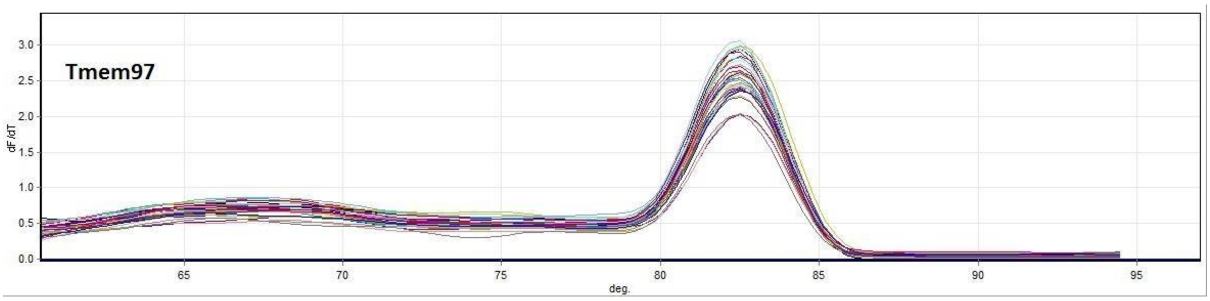
Tmem97
dF/dT
0.0
0.5
1.0
1.5
2.0
2.5
3.0
65
70
75
80
85
90
95
deg.
