# Supplementary material for: Identification of stable reference genes for quantitative PCR in koalas
Source: Sci Rep. 2018 Feb 20;8:3364. doi: 10.1038/s41598-018-21723-0 (PMC5820254; doi:10.1038/s41598-018-21723-0)
Supplement: Supplementary file 3 — Supplementary data set 2 [file 41598_2018_21723_MOESM3_ESM.docx]

>UoN.Pci.T.9055_UoN.Pci.T.9055.1

GCGCAAAGGCCTAAGGGGAAGTGCCTTCCCCTCCGGCCCCCTTGGTTACAGAACCTACCTGTTCCAGGTCCCCCCCCCCAACGTCATCACCCTGCGCCCGTCTGGTTTTCCCGGGAGGGCAGAGGAAGCTGCAGCTGTGGACTCTGTGAAGGCGATAACATCTGCCTTTTCAGTTGCGTCTCTGGAGGGCCAGACCTGGCTCAGAGTAGGGAGCTGACATGTTGGTGCTGCCCCCAGGACTGAGTGAGGAAGAAGAAGCATTGCAGAAGAAATTCAGCAAACTCAAGAAAAAGAAAAAAGCATTATTGGCTCTTAAGAAGCAGAGTAATAGTAGCACAGCCAGCCAGGGGGGCGTCAAACGCTCTCTATCAGAGCAGCCTGTGGTAGACACAGCCACAGCAACAGAGCAGGCTAAACAGCTGGTAAAATCAGGAGCTATCAGTGCCATTAAAGCGGAGACCAAGAATTCTGGCTTCAAGCGGTCACGAACTCTAGAGGGAAAATTGAAGGATCCAGAGAAGGGCCCAGCCCCTACTTTTCAGCCATTTCAGCGAAGCATCTCAGCCGATGATGACTTGCAGGAGTCATCCAGACGTCCCCAGAGGAAATCTCTATATGAGAGCTTTGTTTCATCCAGTGATCGGCTTCGGGAACTAGGACCAGAAGGGGAGGAATCAGAAGGTCCAGGGGGTGGTGATGGCCCTCCCCGAAGCTTTGACTGGGGCTATGAGGAGCGAAGTGGTACCCCCTCTTCAGCCTCCCCACCCCGAAGCCGAAGTCGAGATCGCAGCCATGAGAGGAACCGGGAAAGGGATAGGGACCGGGATCGAGACAGAGACCGGGATAGGGACAAGGACCGAGACCGGGATCGAGACCGGGACAAAGAACGTGATCGAGACCGAGACAGAGAACGTGATCGGGACAGGGACCGAGACCGGGATCGGGACAGAGACCGAGATCGGGACAGGGACCGAGACCGGGATCGAGAAGGCCCTTTCCGAAGGTCTGATTCATTCCCTGAGTACCGAGGTCCACGTAAGGGAAACACACTATATGTATCTGGAACAGATATGACACCCACCCTCCTTCGTGGAGCCTTCTCACCCTTTGGGAACATCATTGACCTTTCCATGGACCCACCCCGAAACTGTGCCTTTGTCACCTATGAGAAGATGGAATCAGCAGATCAGGCAATAGCCGAGCTCAACGGGACCAAGGTAGAATCTGTGCAACTCAAGGTTAGCATTGCTCGTAAGCAGCCCATGCTAGATGCTGCCACTGGCAATTCTGTCTGGGGCTCCCTTGCTGTACAAAGCAGCCCTAAGGGCTGCCATCGGGACAAGAGGACCCAGATTGTCTACAATGATGATGTCTATAAAGAAAACCTTGTGGATGGGTTCTAGGCAGCTGGAAGAGGGGGAAGGAAAATCCCTCCTCTCCTCCCTCTCCTCCTCTTCCCCCCACTCCCGTCCCCCATCCCCTATCCACTCCCCATCCAGGTCTCAGTAAAAACCACTGAGGTGGATATTCAGTTAAAAAAAAAA

>UoN.Pci.T.7633_UoN.Pci.T.7633.1

CACCCTCACGAGGATCACCCCCTCCTAAGCCCCTACCCAAGGGGGAGGTGATAGAAGAGAGGAGGGTGGTCACTCGCGCACGCGCAGTTCTCGAAAAAAAGCCGCAGGGCGGGTCGTTAACGCAAGAAGCCATTTTTCGTTTCCGTGAATGTGCGGGCGGTTGGCGGCTGGCGAAGGAGGGAGAGTTCGCAGCAAGGGAGAGCCAGGGGGGAATGGTGGCTGAGTCCCGGTCGCGCGACCGGAAGCTTAGAGGAAGCCGGAAACTGCTGGGGGGGCGGACCTCCCAGCCTAGCGGCCCGGGCGATCTCTCCAGGCTCCCGCCGTCCCCCGAGGCAGGAGGCAGCCCCGGGGGCGCGGGGTGAAGCAGGGCTCTGTATGGATTTTATATTGGAAGACATGGATCTTGCAGCCAATGAGCTCAGCATCTATGATAAACTTTCAGAGACCATCGATTTAGTGAGGCAGACGGGACATCAGTGTGGAATGTCAGAGAAGGCAATTGAAAAGTTTATCAAACAGCTGCTAGAGAAAAATGAACCTCAGAGGGGACCACCCCGACATCCTCTGCTCATGGTTGCTTACAAGGTCCTGATCACACTGGGGTTAACCCTGCTCACTGCCTACTTTGTGATTCAGCCCTGTAGCCCTTTACCACCTGAACCAGTGCTCTCTGGGGCTCACACCTGGCGCTCTCTGATCCATCATATCAGGCTGATGTCCCTGCCCATCGCCAAGAAATATATGGTTGAAAATAAGGAATATGTTCCTCTGTCTGATTATACTGAGCACCCAAGTGACGAAGGATATGGAGACCTAGAATGTGATGACTGGTGGAAAACCGACTGCAACCCGAACAATACCACCATCCCTTCTAACTGCACTGGCTGTGCAGTGCTTACAGGCCTCCAGGTGGTGCCACTCATGTCACAATTACCCTCCGAATTTGACAAACTCCAGCCTCTGCTGATCAAGACTGGAAACATTCTGTCATCCAAGGAGCTTCATCTTTTTCAATGCCAGTATCCAGAGTTGACAGAAGGGATCTCAGAAGGAATGTTAACCCGGTGGTGGAATTGTTTTCCTCATCAGCGTTTCCCTTTTAATTTCCCATGGAGCAAACCTCTGAATAGAACAAACATTCTACATGAGCTTTTCCCTATGTTCGATTCCTTGCCGTTTCCCAAAGATTCCTCCTTGAAAACCTGCTTTGTCGCCCACCAGCCTCCTGTACTAGGGAGTAAGATGCGTGCGGTTCACGACCTCTTTGTCATCGGCAGTGGTGAGGGTATCATGTACCTCACTCCTCCTATTGAGTGCAGAAGGCACTGTAATACTGTGGTGATGCAGCTGGAGCCAGGGGACGTTGGCTATGCCAGTGTGGATTATTGGAAGATAAGTGTTGGACCAAGAGGAACAGAGCCCTTGGTGATCTGTGATGGAACCACCAACTCAGAAATGTAGAGAAACAGGATTCTGTCCCAGGAGAACCTGGTAGAGCTGAAGAGAAGATCAG

>UoN.Pci.T.15037_UoN.Pci.T.15037.1

TTTTTTTGCATTTTCTATTTTAAAAATTTATTACATCAAAAACAAATACAACCAAACCTAAAACCCAAACCAACCCAAAGATACAAGAAGATTGTAATCGAGGCTTTCTTGCCTAACCCAGAAAAAAAATCTGAAAACAGTTTAAAAAACAAAAGAAATAGAAATGCCAGCTTCAAAAATATTTATCCAAAAAGTAAGGCATCCACTTTACAAAAAAAAAAAAACAACAATACAAGTAGAGGAACAGGTTGAAGGGGATGACCTCCTCCCCTGCACCTTGGCTGCTTGCTCTTATCAAAACCTGGGCCTTGATTAATCAATCATATGAGGCAGAGGGGTAGAGAAGGATTACCTAATGTAGGCTAGAGGTTAAAAGTAGTGGGGAGGAAAAATGGAGCTGTCTTTGGAATTCCTAGTCCCTAGAATGAATTGAGATTTCAAAAAAGGTACTTCCCTAGCTACAGATTTATGTGGGGATGAGTGGTAAGGGACAAAGTCTGGAGAACGAGGAAGGAATTAACAGATCTCAGAGAAAGCTAACTGGGGATCCAATTTCCATGGACCCACGATCTCTAACCCCACAGGTATGGCTGAAAAGGTTAAAAATTAGCCTTAAAACAAAGGACCAATATCATATTTCTATAAAACCCATGGAAAACCAACTAAGACATTCAGTATATCTGTCTCAAGACATAGTGAGGGACTTAATTTCCAACATCTTGGGTAGCCAGCTAAAGGAACTCCTTTCTTCCACAGTGAGGAAGAAAGTAGATGCAGAGTTATTATTTTCAGACATATCTCTCTAAGCTAGCCTCCATCCTCCCTCATAAAAGTCCCCTTTGTCTCTATATTAGTGGTATGTCCTATCTTTGGAAATAAGTCCTGAAGGAAGGTAGGGGGAATGGGAGGTACTGTTTGGATTGGAGTGTATTTATAGGGGAAAGAAGTCTAGAGGGTTGCTTGGAAAGAAAGGGGAAGATAAAATGGCACTGGCCCTCACCTCAGGACATACTCCTCCCCATGACCTATCTCTCAGATCCCCATATCTGAGAATGAGATCCCCTGTGAGAAGTGGGTAGCTCCAGCAGAGAAGGAAGGATGTCTTAAGGGAGAAAAATGGGAAGATTCATGCCCTGATCTTGAAGTTGTCCCAGCCTTGATTTCAAGCTGGAGACTCTCCCTGTAGGGAGAGGCTCTACCCCAGGGAAGAGATAGTGTAAATCTGCAAAATGGCAGCACTGAGAGGTGCAGTTGGGGGTCCTTGGGGACAGAGAAGGGTAAATACCAGTGATAATTACCAAATATCTCCTTCCCACTAAGGACAGCCCTGAGGCAGAGAGAGCTGCTAAACCCTGTTACTAGTAAAAAAAAAAAAGCCACTTCTGTGAAACCAACCAGGAAACAAGTTTCTCTGGGTTCAAAAAGCTTTGGCACCAAACTGGGGGGAGCATACAGGGAACACTACAGTCCCTGTAATCTGGGACTTTGCAGATCTGTTCCACAGGCTTGGGGTGTGCTTGAATGCTTGTGTGTAGGGGAGGGGGAAGGTGAGGCAGGGGAAGAAGAGGCAGTGCTCATCCTTCATTGGTCCTTAACCCTAAAGGAAATTTGTACATGTCTGACAAGGAGCTACAAGTAGTCAAGACCAAGTGTGTGTGTGAAAGCACCTATGTCCTTTCTCTGAGACTCCTGCACCCTATGACAGGATCACAAGCTGCAGCTGCCATCAGTTCCCCTTCCCTCGAAGACCTCTGGCTTCCATGTCTGAGTTGTTCCTCCTTCCCCAACTCAGGCCTCTGTTCCTTAAGCTTAGAAAGGGGAAGGAGAGAGAAGGCAATACCAAAAATGGTTGGATTCCTGAGGATGCTGCTCCAGAGGTTTTTGCCTGCTTGTGGGCACCAGGATTTGCTAAGGATAATATCTCTGGATGGCCCTCACTCTAAAAGTTGGGGTCTGTGTAGAGGAGATATCCCATGGCTAGACTTCCTCTCCCCTGCAGTACTGGTGAATGGTATTCCTACATAGACCCTACTGGTCTCTGGCTGGTTCTGTGGGTACAAATTACAGTGTCTTTATCTACCATTTCCCTCCCCTACCTCTCTGACTGCACGCACCTAAGAACAGTTTTGGGGTGAGGGTAAAGAAGGAGGCTGAGTGAAGGAGTTGAAGGGGGAATCAAAGGCTAGAAAAGCTCAAAGAAAAGCTGCTCAAAATCAGGGTCGGGATGAATCGGTTGACTCCGGCCCACTGGGGCCAGTCGATTTGTAGATGGGTTGCCTCTGTTCTTTCATGTCATCAAGAGATTCCTCCTCTTTCGATGGGCTCTCATCCACCTGGGGATACACGACCACACACAGCTTCTGGCTCAACGAGGTCAGGGATGCTATGAAGTCATTGAAACACTGGGGTTTGTGCTGCCAATACACACCGAGAAAGTAGACAGGGACACCTGTCATCATGATAGCCAGGCCAATGCCACAAACCACGGGCTCCGACCACAAACTAAAGACCAGCAGGAAGGCCCAGAACAGCAGGTAGACAACAGGAAATAGCAGGCTTATCTTGATGGGGCGGGCAATATTAGGTTGCTTCCATCGCAGGACAATTTGCCCAGCAATTGTGACGCCATAGAATAAGTAATTGATGAAGCCTACATAGTTGATGAGCGTATATATGTCACTGGTCACCAGCATCAGCAGGGTAGAGATACACGTGAAGAGGAGAGCAGGGATAGGAGTACAACGTTTTATGTGTATCATAGCTAGCACACTGGGCAGGTGGCCCTCCCGGGCTCCAGCAAAGAATAGTCTGGAAGACGTAAAGAGAGATCCGTTGACGCCACCGAATGTGGATAGCGCAACTGAAATGGGCATGATCCAGGCCATGACGCCGAGAAGCTTCTCTCCAAAGGTCACAGCAACAGCATTGGAGGCCAGCAGCTCCTGGGGTGACATCGCAGTGACATAAGCAATGTTGGCAAAGACGTACACAAAAGTGACCAGAGGGATGGAGATGAAAATAGCTCGAGGAAGGTTCTTGTAGGGATCAACCAGTTCCTCTGTCACATAATTAAGGAAGTTCCAGCCTCCATAGGCAAAGGAGCCCTGAAGGAAAGCCAGTGCGATGAGGCCGATGTCCGGCTTTTGGAAATTCTCAAAAGCGTTTTTGGGCTCTAGCCAGAAGTACTGTCCTTTGCAGATCTGCACAATCCCCATGACAATAATCAGGCCCAGGGCCAAGAGCTTCCCAGCTGTGAAGACGTCCTGCACTCGGGTGGCCCATCGCACACTTGCACAGTTGACCCATGTGAGAAGCAATAAGCAGACTGCAGCCAGGAGTCGTAGTCCAGACTCTGGAGGAAAACAGGTGGGGAAGAGTGGTTGCAGCACATAATTGGAGAAGGTGAGAGCAATGACAGCCTGGTTGGTGGGGTAGATTACTAGCACAGCAATCCACAACCTTAGGAACCCAGCCAGGCCTCCAAAAATGTCCTTGACATAAGAGTAGTCACCTCCAGATTTAGGGATAGTAACTCCAAGTTCAGCATAACACAATGCACCCACAGCTGTGATGAGCCCGGTTACAATCCATACAATCAGAGCAAGGCCCACAGAGCCAGCATTCTCCAGAACACCTTTTGGTGAGACAAAAATTCCTGAACCAATGATGTTTCCTACAATGATGCCGCAGGCACTGATCAACCCAATCTCTTTCTTCAGTGCTACTCCACCCCCTCCAGAACCAGCATCTGGACCAGGATCAGACTCGCTCCCGCCTGTGGGGCTCTTTTCAACGTTGTTTCGGTGCCTGGCCTTTTCTTCCATCTTTTTTTCAGAAAAAAAAAATCAACCACTGAAATTTCCTTCCCCTCAGCCTACTTATCTATTTGTATTTCTTCCTAAGCCTTTCAAATGAAAATGTCCTTTTCCTAAAATGACCCTTCGTCCTGGCCCTTTAAAAGAGGCACATGTAGATAAAAAAGAAAATCTCAAATTTACATTTAGTATAATTAAGACACTTTCCGGTCGCTCTAGGTTGCCACCAAAGAAAGTGGTTTAAAAAGGAAAAATAGACATGTGGACAAAAATAGAATTGAACCAGCTACTCCCTCGAGCACTCACAGGAGTGAGGGGATGTGGATCTCCCGAACCAGAGCTGAATTCTGCTCAATTTACACAGGCAGCTGCTTCATGCAGATGCACCCAGACAGTTTGGATTTCCCTTTAACACTCTCCTCAAACCAATATCCAAACTGGCTTTTAGCTCAGCTCGGTTTTTTTTTTCTATGAAGGTTAGAGCATTTTTTTTATTGACTGAGTCTTGGGGAAAAAAAAGGCAATCCTCAGGTGCTTTCTTCTCTTTTATAAGAACAAACTGCAGACAAGCCGGTTTGCTCTAGATTTTGTTCCACGGGGTGTGGATTTTCCTTTCCCTTCCTCTCTCCTCCCCTTTCCTTTCTCCTGCCTTCAGATTTTCCCCACTGTTCTCATTTATTCTTATACGCTAGTCTCTTTTCTTCCTGTCCTTGGTTTTGTTTTGCTTCGTCTTGTTTTCCTCTTTCTATCTTATAGCTCTGAGTTCTGCATCTCTCTCCGTAGCCACAGGAAGAGTAGCTTCTTCACG

>UoN.Pci.T.15037_UoN.Pci.T.15037.2

TTGCATTTTCTATTTTAAAAATTTATTACATCAAAAACAAATACAACCAAACCTAAAACCCAAACCAACCCAAAGATACAAGAAGATTGTAATCGAGGCTTTCTTGCCTAACCCAGAAAAAAAATCTGAAAACAGTTTAAAAAACAAAAGAAATAGAAATGCCAGCTTCAAAAATATTTATCCAAAAAGTAAGGCATCCACTTTACAAAAAAAAAAAAACAACAATACAAGTAGAGGAACAGGTTGAAGGGGATGACCTCCTCCCCTGCACCTTGGCTGCTTGCTCTTATCAAAACCTGGGCCTTGATTAATCAATCATATGAGGCAGAGGGGTAGAGAAGGATTACCTAATGTAGGCTAGAGGTTAAAAGTAGTGGGGAGGAAAAATGGAGCTGTCTTTGGAATTCCTAGTCCCTAGAATGAATTGAGATTTCAAAAAAGGTACTTCCCTAGCTACAGATTTATGTGGGGATGAGTGGTAAGGGACAAAGTCTGGAGAACGAGGAAGGAATTAACAGATCTCAGAGAAAGCTAACTGGGGATCCAATTTCCATGGACCCACGATCTCTAACCCCACAGGTATGGCTGAAAAGGTTAAAAATTAGCCTTAAAACAAAGGACCAATATCATATTTCTATAAAACCCATGGAAAACCAACTAAGACATTCAGTATATCTGTCTCAAGACATAGTGAGGGACTTAATTTCCAACATCTTGGGTAGCCAGCTAAAGGAACTCCTTTCTTCCACAGTGAGGAAGAAAGTAGATGCAGAGTTATTATTTTCAGACATATCTCTCTAAGCTAGCCTCCATCCTCCCTCATAAAAGTCCCCTTTGTCTCTATATTAGTGGTATGTCCTATCTTTGGAAATAAGTCCTGAAGGAAGGTAGGGGGAATGGGAGGTACTGTTTGGATTGGAGTGTATTTATAGGGGAAAGAAGTCTAGAGGGTTGCTTGGAAAGAAAGGGGAAGATAAAATGGCACTGGCCCTCACCTCAGGACATACTCCTCCCCATGACCTATCTCTCAGATCCCCATATCTGAGAATGAGATCCCCTGTGAGAAGTGGGTAGCTCCAGCAGAGAAGGAAGGATGTCTTAAGGGAGAAAAATGGGAAGATTCATGCCCTGATCTTGAAGTTGTCCCAGCCTTGATTTCAAGCTGGAGACTCTCCCTGTAGGGAGAGGCTCTACCCCAGGGAAGAGATAGTGTAAATCTGCAAAATGGCAGCACTGAGAGGTGCAGTTGGGGGTCCTTGGGGACAGAGAAGGGTAAATACCAGTGATAATTACCAAATATCTCCTTCCCACTAAGGACAGCCCTGAGGCAGAGAGAGCTGCTAAACCCTGTTACTAGTAAAAAAAAAAAAGCCACTTCTGTGAAACCAACCAGGAAACAAGTTTCTCTGGGTTCAAAAAGCTTTGGCACCAAACTGGGGGGAGCATACAGGGAACACTACAGTCCCTGTAATCTGGGACTTTGCAGATCTGTTCCACAGGCTTGGGGTGTGCTTGAATGCTTGTGTGTAGGGGAGGGGGAAGGTGAGGCAGGGGAAGAAGAGGCAGTGCTCATCCTTCATTGGTCCTTAACCCTAAAGGAAATTTGTACATGTCTGACAAGGAGCTACAAGTAGTCAAGACCAAGTGTGTGTGTGAAAGCACCTATGTCCTTTCTCTGAGACTCCTGCACCCTATGACAGGATCACAAGCTGCAGCTGCCATCAGTTCCCCTTCCCTCGAAGACCTCTGGCTTCCATGTCTGAGTTGTTCCTCCTTCCCCAACTCAGGCCTCTGTTCCTTAAGCTTAGAAAGGGGAAGGAGAGAGAAGGCAATACCAAAAATGGTTGGATTCCTGAGGATGCTGCTCCAGAGGTTTTTGCCTGCTTGTGGGCACCAGGATTTGCTAAGGATAATATCTCTGGATGGCCCTCACTCTAAAAGTTGGGGTCTGTGTAGAGGAGATATCCCATGGCTAGACTTCCTCTCCCCTGCAGTACTGGTGAATGGTATTCCTACATAGACCCTACTGGTCTCTGGCTGGTTCTGTGGGTACAAATTACAGTGTCTTTATCTACCATTTCCCTCCCCTACCTCTCTGACTGCACGCACCTAAGAACAGTTTTGGGGTGAGGGTAAAGAAGGAGGCTGAGTGAAGGAGTTGAAGGGGGAATCAAAGGCTAGAAAAGCTCAAAGAAAAGCTGCTCAAAATCAGGGTCGGGATGAATCGGTTGACTCCGGCCCACTGGGGCCAGTCGATTTGTAGATGGGTTGCCTCTGTTCTTTCATGTCATCAAGAGATTCCTCCTCTTTCGATGGGCTCTCATCCACCTGGGGATACACGACCACACACAGCTTCTGGCTCAACGAGGTCAGGGATGCTATGAAGTCATTGAAACACTGGGGTTTGTGCTGCCAATACACACCGAGAAAGTAGACAGGGACACCTGTCATCATGATAGCCAGGCCAATGCCACAAACCACGGGCTCCGACCACAAACTAAAGACCAGCAGGAAGGCCCAGAACAGCAGGTAGACAACAGGAAATAGCAGGCTTATCTTGATGGGGCGGGCAATATTAGGTTGCTTCCATCGCAGGACAATTTGCCCAGCAATTGTGACGCCATAGAATAAGTAATTGATGAAGCCTACATAGTTGATGAGCGTATATATGTCACTGGTCACCAGCATCAGCAGGGTAGAGATACACGTGAAGAGGAGAGCAGGGATAGGAGTACAACGTTTTATGTGTATCATAGCTAGCACACTGGGCAGGTGGCCCTCCCGGGCTCCAGCAAAGAATAGTCTGGAAGACGTAAAGAGAGATCCGTTGACGCCACCGAATGTGGATAGCGCAACTGAAATGGGCATGATCCAGGCCATGACGCCGAGAAGCTTCTCTCCAAAGGTCACAGCAACAGCATTGGAGGCCAGCAGCTCCTGGGGTGACATCGCAGTGACATAAGCAATGTTGGCAAAGACGTACACAAAAGTGACCAGAGGGATGGAGATGAAAATAGCTCGAGGAAGGTTCTTGTAGGGATCAACCAGTTCCTCTGTCACATAATTAAGGAAGTTCCAGCCTCCATAGGCAAAGGAGCCCTGAAGGAAAGCCAGTGCGATGAGGCCGATGTCCGGCTTTTGGAAATTCTCAAAAGCGTTTTTGGGCTCTAGCCAGAAGTACTGTCCTTTGCAGATCTGCACAATCCCCATGACAATAATCAGGCCCAGGGCCAAGAGCTTCCCAGCTGTGAAGACGTCCTGCACTCGGGTGGCCCATCGCACACTTGCACAGTTGACCCATGTGAGAAGCAATAAGCAGACTGCAGCCAGGAGTCGTAGTCCAGACTCTGGAGGAAAACAGGTGGGGAAGAGTGGTTGCAGCACATAATTGGAGAAGGTGAGAGCAATGACAGCCTGGTTGGTGGGGTAGATTACTAGCACAGCAATCCACAACCTTAGGAACCCAGCCAGGCCTCCAAAAATGTCCTTGACATAAGAGTAGTCACCTCCAGATTTAGGGATAGTAACTCCAAGTTCAGCATAACACAATGCACCCACAGCTGTGATGAGCCCGGTTACAATCCATACAATCAGAGCAAGGCCCACAGAGCCAGCATTCTCCAGAACACCTTTTGGTGAGACAAAAATTCCTGAACCAATGATGTTTCCTACAATGATGCCGCAGGCACTGATCAACCCAATCTCTTTCTTCAGTGCTACTCCACCCCCTCCAGAACCAGCATCTGGACCAGGATCAGACTCGCTCCCGCCTGTGGGGCTCTTTTCAACGTTGTTTCGGTGCCTGGCCTTTTCTTCCATCTTTTTTTCAGAAAAAAAAAATCAACCACTGAAATTTCCTTCCCCTCAGCCTACTTATCTATTTGTATTTCTTCCTAAGCCTTTCAAATGAAAATGTCCTTTTCCTAAAATGACCCTTCGTCCTGGCCCTTTAAAAGAGGCACATGTAGATAAAAAAGAAAATCTCAAATTTACATTTAGTATAATTAAGACACTTTCCGGTCGCTCTAGGTTGCCACCAAAGAAAGTGGTTTAAAAAGGAAAAATAGACATGTGGACAAAAATAGAATTGAACCAGCTACTCCCTCGAGCACTCACAGGAGTGAGGGGATGTGGATCTCCCGAACCAGAGCTGAATTCTGCTCAATTTACACAGGCAGCTGCTTCATGCAGATGCACCCAGACAGTTTGGATTTCCCTTTAACACTCTCCTCAAACCAATATCCAAACTGGCTTTTAGCTCAGCTCGGTTTTTTTTTTCTATGAAGGTTAGAGCATTTTTTTTATTGACTGAGTCTTGGGGAAAAAAAAGGCAATCCTCAGGTGCTTTCTTCTCTTTTATAAGAACAAACTGCAGACAAGCCGGTTTGCTCTAGATTTTGTTCCACGGGGTGTGGATTTTCCTTTCCCTTCCTCTCTCCTCCCCTTTCCTTTCTCCTGCCTTCAGATTTTCCCCACTGTTCTCATTTATTCTTATACGCTAGTCTCTTTTCTTCCTGTCCTTGGTTTTGTTTTGCTTCGTCTTGTTTTCCTCTTTCTATCTTATAGCTCTGAGTTCTGCATCTCTCTCCGTAGCCACAGGAAGAGTAGCTTCT

>UoN.Pci.T.15078_UoN.Pci.T.15078.1

GCTGGGGCGGTGACGCGGTCGCCTCCGCCGCCTTCCTTTCTCCGAACCCTGCGACGAGCTCAGCTCCCCTCCCCCTCCCCCTCGCGCTCCCGCCCCTCCTTCTCCTGCCCGCAGCTACTGCCTGCGCGAGGCGGACGGACAAGGGCACGCGGGGCCTGGCGGCTGAGACTGGGCTGCGGGGCCGCGGAGGTGCGGCGGGCGGGCGGGCAGACGGGCAGCCTGGACCAGCCGACGTCTGGCCGGAGGGCGCCGCTCCTGCTCTTTGCCCGGTTGCTCCAGGTTGAACTCGTACCGTGTGCATGTGGCAAAGGAAAAACTTGACTCATTGATACAGACATCAGTCCTCTTCTCGTCAGCTGTGTCTTCCCTTCTTCGCTCAGAGCCTCCATGACTTGTGGGGCAGAATTGGCTCTTTTGTCAAAGTCCAGCTGTGCTTGAAAGATTTCAAGGTGGATTTGTACCTGGCTGGCAACTTTTTTACCCCCTTTTTCTTTCAAACTTGGACGGATAAAAGATGTGCTATGGCAGGATAACACCAAAGAGCAGCTCAGCCTCACCATTTGCCTCCTTAGGACATGGATTACTTCTTCCATTCATGATTATTAACACTTTCCTTGGAAATGGAATTGCTTCAGTGTGTCCTCGACCACCAGCACCAGAAAATGGTGGCTTCATCTGCCATCCAACCCCCTGTAAAGATCCCTTGGCATCAGGCAGTGTCATCGAGTACCTTTGTGATGAAGGCTACATGCTGAAAGGGGATTACAAATACCTGACATGTAAAGATGGGGAATGGAACCCAGCCATGGAGGTCAGCTGCCATCACAGCCAAGGTCAGCCCAGCACTGATGCTAAAGGATATGAGGGAGAAAGTGCAATTCCCTTTTGGAAGTGGGAAGTCGTGTCTACTTCCTGCAGTGTTGGCCCGAGTGTAATCTGTACACAGGCTACTTGCACGTACAAATCACAGGTTCCAATGCCCCCCTCATCCCCCTTCTCCTTGTAAACTCTGTCTTTGTGAAAGACATCTGTCACTAGTGCACATGCCACTGCATGTAACTGAAGGGTGACCTTTAAGATGCTTGATAATTTGCCAAGATATTTCCCTGGTATAAAGATTAACTCTTCTGATTCTCAAAGTCTCTTGTTTTCATAGCCAACTGCTGGTGACCTTTGAGCCTCATCTTCTGGACACAGTGATTATTGTTCTCTACTTCTTTCCCTACCCTATCCCCTCAGTGGAAATTGACTCTTTTGTTATGTGTACAACAGTCCAGCTGGAGAGATCTCTCTCCCCTTGTATTTGACCTAACAGGAAAACACATGCAGCCTGTAGGAAAGTGTGTGTCATATGCCCTGTTTGTGCTTCATGCATGGATTTCTAATCCAAACCATGTTTAGCCCATGCAGCCTTAGGGGAAGCCCTGAGGGGCAAAGAAAAGTGAGTGTTTCTTACAGAAGCTGGAACATGAGTCGTTCTCCGTCCAGCTGTCACAGGCTGTTGTTCTGCAGGAAAACACAGGTGAAATCTAAGACATCACAGTGACACTCACTAGTTCACAATTGATTTTTAAAACTTCCTCCTGCTATCAGAGAGAGAGGTAATTTGAGTAGATCCAATGACCTAATCGTGGTAGTTATATATAGGTATAGGGAGCCAAAAGTGAGGAGAATTCGGTATTTAGGAATCAAGATGAGGAAAGTTTCGCAGTGATGTGTATGACAGAGCTTGACATGTATTTGTTGGCCCATCCTCTATTTGGACTGGTAGGTTGGGTTTTGTGGGTATGGACAGCTACTGATGGCTGCTTGTTTCTGTAGACAAAGACATTCGCACAACGATCGGTGTGCCTGCCCTGTCCATAGTGGCCTCTACAGCAAGCTCAGTAGCACTCATCCTTCTCCTGGTTGTGCTTTTTGTTCTGCTGCAGCCAAAGTTGAAGTCCTTCCATCATAGCAGGCGTGACCAAGGGGTATCTGGTGACCAGGTCTCCATTATGGTGGATGGTGTTCAGGTGGCCTTGCCGTCATATGAAGAAGCAGTGTATGGCAGCTCTGGGAATTCTGTGCTGCCTTCTAACTCCCGAGTGCAAATTGTGTTGTCTGAAGGGGCCGGGCCAAGCAGCACAAGGGAAGTGCAGCAGCAAGACCAAGGAAGCCATGTCTGCTCTTCCTCAGGGGGAGAGGATGATGCCCCAGAGCACTCCAGCTTAGGTGATGCCCGCAGTGCTCAGCGCGCAGAGACTGTGATGGTGCACCAGGCCACCACCTCCTCCTGGGTGGCCGGCCCGGGAAACAGCCATCCAGCACACAAAGACACTGCAGACTCAGAAAACAGCGACATACAAAGCCTTTTATCTCTCACTTCTGAGGAATATACAGATGATATCCCGTTGTTGAAAGAAGCATGAAGTTTGCTGCCGTCTTCATGTGGCCTCCTTCCCCTTTACGTTGTTCCTTCCTGCCCCCTTCCATTTCCCCGGGGGTTTTGAGCACTCTGTGCAGCCTCTATGCCCAGTCCTATGCCCTCCTGCCCTGGTGATTAGAGCAGCGCCCCAAAGTCACTCCTGGCCGACCATCCAAAGGAAACCTTTGCTGGGCAGCCAACGGGGCTGGTCCCGCGCTTGGCCTCTCCTGCCTCTCTCTTTCTGGAACTTATTTGAGTGAGCTGAGATTGGGCCCTTCCTTCCTTCCACCTCCTCAGACCTCTTCAGCCCTCCCCTCAGCCAGCTGTTTAGCAGCGGCCCCCCGCAGGCCATAGCACGGCTGTCTTTACTTGTGCCTTCCCCCGGCCCCCAGGCCTGTTGGCTCCACACTGGCCTGAGTGGTTGCGCTGTTGCTGCTGCTGTTGCTGCACAGGATGCAAAAGGTCTATGGGGCCAGTGCCCCCACCATTACTATTAGTAGCAGTAAGCCCAAGGTTGGGAGCTTGCTGGTGAGAAAAGGCTTCAGGGTAGAGGCAGTGATCATGGGGGGATTGGAGGTGGAAGAAGAGGGGAATCACCCCCCAGTGGCTGCCTTGTGATGAGCAGCTGTATACTTGGATGACACCTCTGTGACTTCTTTAACTATTTAATAGAGGGATTATTAAGAATCATGCCAGTTTTCAGTAGTTCTGTGTTGATCAATCCAGGGGCCAAGAATGCTGGCCCCTCTTTAATTGATATTTTTAGGTGATACATGTGAGAGATCTTGAAGGTGGGGAAGGGAGAGCATGTGGAGGAGGCAGCTGGTGTCTTTCTAATATGCAAGATCCCACTTACGAGTCTTGGCTTTCTCAGCCATGACCTTTGTCTCGCCCCCATTCTCTAGATTCTAAAGGAAAGCGACATGTGGCCTCCGGGGTTTGTTTTCCTTTTCCCAGGCCCCTGTGCCAGAAATGGCTCATCTGTAGAAAGAAGAAGAGAAGTCAGAAACCATCTCGTTCCCGTCGTCATTCCCTCCTGCACATGCTGGTTTCCCAGGCTTGCAACTAGCATTTTGGGCCAGTTGCATCTTTCTGGGTGTGTGGTAATATTATGGAGCTGGAGGAGCTGACGTGCACTCCTCTCCCCGACACAGTGCCTTGGGTGTGGCTGCAGGCAGCCCAAACCCTGGGTCCTGAGCATTCCTGTCAACACAGTCACGTGCCTTAGTAACTGTGCCCAAGAAGCACCCTGCTACTTAGTGCTGCTGCTTTTCCCCCTTCACCTTTTCCTCTCCCACCTCCCCTTCCTCTTTCTCCTTTGTCACAGCTGACAGATGACTTTCTTGTCTTCCTTGGAGCTCAGGCAGAAGGGCTAAGAAAGAGCCTGAGCTACCACCCTCACTCCAGTAAGGGGACGGCCTTGCTGGGGGTGAATGGTTGCTTAGCAACTCTGCTCAAGTGGCACTTAGGTTGAGGGATAGAGAGGGAGGGGTACTCTAGAAGAGCCGTGTGTGTTTGGGGAGCAGGCATCATTCTCTAGGGCCATTGGTTTCCTAGAGAAAAGACCAGAGTGAAGGCATTTAGGCTTTTTATAAGGTTACTTGGCAGAGCCTCCCATAGACAGCTTCCATGATGGTGTACGTAGAGAGCGATTCAGTTCTTTGACCATCGTGAAAGCTCAGAAGGACGTTGGATAAAGGGGACCGAGGCTTGGGAAGCTCTTAGAAACACTGGGGCAGAAGACTTGGAAGATGCCCCTCTTTTATTCTTCTCCCCCCAGCTGCCTTTTCCAGATGGTGGTAATAACTTCCCATTATTATGTAACCTGCTCAACAGAGCGTTTGGGGACTACCTGCAGCTGTCTTAGACAATGGGAGTGACCACAAAGAACAACTAGAAAGAGAAACTTCTCTCAGCCAGCAGTGTGGCTGCCTCCCCCCTTAACCACCCGTACACATCCTTACACACCGCACTTTATTGCACTGTCATCCCCTTGTTTTAGCAACAAGAAGCAGTCTCGCTTCCTCCTAGAGTCATAATAATAGCTGACATTTATGTGGCGCTTTGTAAGGGAAGTGCCTGTTGTGTTCCATTATTTACTTCCTCCCCACTTGCCCTCCACTACCACACCCACATAACTTACCCAAAGCCATGTGAGTTGGCTGGGAATTTGGGGAGAGGCCCTGTAAGGAGACTTCACAGCAGGCACTGCTGCTTCGGAGATGAACTTAAGACTAGGAGAGTGCAATGAAGCAAAAAGGATGGCAAGTGAACCCCCTGTCTCCGCAACCACTTTTAGTATCTAGAACCACAGGCAGCTTCTGGTCCCCGGGAGGAGTAGGGGGAGGGTCCCTCATGCCCACTGCTGCCTGGCCACAAGTGGAGCGTGCCCAAGCCCTTCCTTTCAGTCTTCATAAAATGGTGAAAAATAGAAACAGTTGAGGGAGAGACGATCCTGCCTCCCTATCAAGCCTTCAGCTGGTGCTTTCCAGTGTTTGAATATCTGCATGGACCTATGTCCCAGGATACCAGAAAGAGAATATCTTAGCATGTTTTTTTGTTGGAATGATCCTGTCCTCAAAGAATACACTCCAGGACCCTATGTTTACTGAGCATCAAGTGGACTGTTTGCTGGCCCTTTAAAAAGGGAAAAAGAAAGCTTCCCCATTTCTGAAGATCCCTTCCACTGGTCTTGGTCAGACAGAGCAGCCTACCCAGGGGACTGATTGCATGCCTAGACATGCTGCCAAATTAAAAGGGAAAAGGAAAAGGTGAGGAGGAGACTAAATCCATGAGTCCTTTTCCCCCACCTGCTTTGGTTACTGGAATATGAAATAAAGTCAGGGCTGTTGAGAAACCTGTATTGGATTAAATTAGACCTCTTTCGTTAAATAGCCTATCCTGTGTAGGCTACACGTTAGCTGCTACTAACCAGAACAGCTTCAAATAGAGTAATTCCCTCCCTCCCCCAGAACAACCTTTTAATGACTCCTCAGCAGGAAAACAGCTGGCATTTAGCCCCTCAGTACTGTTTTCAGGACTCATGGACTTGACTTTGACAATTGTAAAGGGATTCAGGGAGTTTAACAGCTGCTTTGAGGTGGGTCCTGGCCAAGAGACAAAGGCATGCTCTATTGGATAGAGCAACTAGGCTGGTTGTTGGTGGTCCCTAGAAGAAAAAAAAAAGGGTACCCAAAGGAGCTTCTTTGGATGGGAATGAGACACACTGTTTGACAAAACACAAACCACTGTAGGTGAACTGAGCAAAAAGCATGTGTCAGCTTTTTAGGTCTTAGGAAAGTAATGGTCAGCACCAAAGGACTAAATTTTTTTTTCTTTTTTTTCTTTTTTGCACAAAAAATATTCTGTCTGCCTCAGTGCAAGTTTTATATCGTATTGACTTGCACCATCATAATTACTTCAAAGAGAACCAGTGTAAGGTGAAAACTCTTCTTAAAGTAATACCTGTTCTTTGCAATGCCTTAGTTTAACACTTTGCCATCTCTGCAGTTTGTTTACTTTGCCACCTCCCGTTGCACCTGGTGTCAACTTGACCACTTTGGTGCTGTGCTGTTGTTTCCCCCTAATTCTTCTGAGTTGGGGTTCCAGCACTGCCCAGGAAAGCTTCCATCTAATTCTTTTTCCAAGCAGATTTTATTAGATACAGTATTTCACTGTACTTTCTATAAATCTATTTAAAAACAAAATTTAAACTAATATCCTCATTTGCCTCCATTCTTCAGATACATCTGTCTGTCCCCCTCCTGCACATTCTAATAACCTCTAATGAGAAGCTACCTAGCAAGACAAAGGTATAGTTTTCCTGCTTTCATTCCCAGGAACACCAGAGTAGCAGTCTAGTCATCCTTTCCTGCATCCTAGCCTAGCTTTCAGGTTCTTTGCTTGCATATCTCCTGCTTCCTACATTTTTAGGAAAGCATGTGAGCACAGAGATTGGAATATACTATAAAAATGTCTGTCTTGGCTTTAAAAAAAAAAAAAAGACAGGCCCCACAGTTAGGTTCCAGATGTGGGTTGGGGATGGCAAAACTGCCTTTCCCACGGGAACCATTTGGCTTCCTTCTTTTCGTTGGATTCACTCTGCCTGTTGCCAGGAGAGGAGTTGAAGCGCTTTTTTTTTTTTTGCTTTGGACAGATGTTCTGGTTGAGCACTGTACCTGATAGCTTCCATGAAAAGGGGGAAAACAATGGATTGGACCTTACTAGGAAGCAATGCCTGCTTTGGTTTTGGCTTAAACTTGAGGTTTTCAAATGAAAGCCCCAAAGCCTGACTGAGTACTCTGCCAAGCAGAGGAGGGGAGTCCAGGAGGGACAAGTGTGGGAGCTGAGGTGTCAGACTACCAGTGAGGGAAGAGATTGGTAGTTAAGCCAGCAAGAGTTTGGTGGCCTATCACGCATTTCTTCAGTTCTTATCCAAACTGCACCACTATGGAATCCTTTGTAGAATGGTACTCATACATAATGTGGGACTTCAAACAACAAATAAGTAATTGATGCTGTGCAGGATGTCAATCATTTTTATTTTGCTTTATTTTATAAACATGTATCCTGTACATTGCAGTGGGTGTGAAGATAGTATTTTAATATTTGTACAAAGTTTAATTTAATTTTAATTGTTCTATGTATATAACTGCATTTCTAAATAATAATAATAAAAGCAAGTTATTTTAAAATGAAAAAAAA

>UoN.Pci.T.15338_UoN.Pci.T.15338.1

TTTTTTAAAGGTTTTTTTAAATTATTTAATTTTTCTTTCAATATAAACATTATCTTAAGCCAGAGAGATTCTTTTGAGGTGAAAAGTTAATGTTATGAAGAAAAGCAGACCTAAGAACATAAGGATACAATTTATGCAATGCACCTTCAAATCCATTTCCAGCAACTGCAACAATCAAATGACAAATCAGTGAAAAATTAAAACTTGAGTAAAGTCATTATCTGTTATGAAACTGTTAAATGTTTTCCCACCAACTTAATTCTGAAGACTTCTGCCACATAATCACAAAGGCTTTACCAGTGGAAAGAAATCTCAGTTCTTCTCCTGGTCCCCAGAAACTAATAGACCCTTCAAAGCTTGGCATAGTCTTTAAGAATCAGCTCTAGCTTCTGGCTCAGCATGATGTTGCCTTTGACCCGAAGTTTGCCACTAAAGAAGGCCTTTTGGGGGTTAAGCTTGCCCATAACAACGTCCATGAAGTCTTCATCTGAAAGAGTGAAGACTGTGTCAGCAGAACCTCTTGCAGGGCCTTGGTATAATTCTCCAGAGCCATTTTTTAAGTCAACAGTCCATTGTGCTGCAGTTTGTCCATGTTTAGTGATGTTCCACTCAAATACAGCATTCACCTTCTTCACCAGTTCCTTTCCAACATCTTTGATGCGACGACCTATTTCCTCAAAAACAAGGGTGCTCTGAAGCACTCCATCCTTAGAAGATACCTTAGCTGAGAGATCATCAGATGTTGGTACAAAATCCACATATGCATTTGAAAGGACAATGTCTCCAGTTTCTTGGACCTTGGTCTGAAAGTGAATTCTACTTCCTTCTTTCCACATTTCTGTTGATAGAGTCTGTCCTGGATATACTGGTTTTGCAAAACGGGCCTTAATCGCTTTGAATCTAGACACGTCGTTATTTCCAAACTGCTGTAAAACATGCCTAGCTGAAAATCCAAAAGTACAGAGGCCATGTAATATGGGTTTATCAAAGCCTCCAAAACTAGCAAAGCTAGGATCAATATGTAAAGGATTCCAATCTCCGCTGAGCCGATATAAAACAGCCTGATTGAGTGAGGTGGTGTCTGTAAGCACAGCATCAGGTGGTCTCTTTGGTGGTGGCACAGTTACCTTGGCTTTATTTGATGTCTTCTTTCCACCAAACCCTCCAGAGCCAACAATGAAGAGAGAGAACTGATTAAAGCATATAAGCTCTTTCCCACTATAGGAATAAACATCCAGAAGTATCACTAATCCTGATCCTTTATCCAAGATATCAACAATAACAGATTCACTGGTTAACTGTCCTGTTCTAGGAAGTGGTTTATACAACTCCAAGTACTGCTCACCATGAAGCAACTTTGCAAAGTTTATATTCAGTCCTGGAACTTCTGAGAATCCTCCCATCATAGATTTCTGAGCTGGGATGATGCCAAAGGTAGGTAAGCAGGAAAAATCTGAGCTTCCCTCATAAACAAATTTTAAGTTTTCTGGATTCTTAATTGATGCACCCACTCCAAGGGCATACATAATAGCTTCAAGTTCAGAGTAAGAAAAGGTCTTCTGAGGCAGCTTATAGCCAATTGCTCTATTAAATGCTGAAGTGGTCTCTGATACAGCATGACTTGTACGATTCATAGAAATGCCTTCCTGAGAGTCTATTTTACCTAGGACTTCAATCACACCTGCAGTTGACTCTTGGATAGTCTGAGGCTTGAGAGCATTGTCAAAGTTACAGATCTTCTTCCAATTAGCCTTCACATCCTCAGGTGTCATTGACTGATTTTTCTGCCTGACGATGGCACCAAGAGTTCGTTCCCAACGTAATTTCCCAATCCATCCTGCTCCAACCTCAAATAAGCTTCCGTTCTCTTCACAGCTCTCATGGCATAGCCAGAGGACAAGGGGAGCCACATAATCCGGCTTCAAAGCATCCAGTAGGTCCTGTGGCATAACAGTTTGAGTCATCCGGGATCCAGCGGTAGGTGCAATAGTATTACAATGGATGTTGAATTTCCTGCCTTCAATTGCAAGTGTATTTGAAAGACCCAGAAGACCAAGTTTAGCTGCACTATAATTTGCTTGACCAAAATTGCCATAAATTCCGGAAGCTGATGAAGTCATAATGATCCTTCCAAACTTTTGTTTCTTCATGTGATCCCATGCTGCCCGGGTCACTTGGAATGATCCCCACAAATGAACTTTATGGACTATATTCCAGTCTTCATCACTTATCCTAACAAAGGAACGATCCCGCAGGATCCCAGCATTGTTTATAACAATATCTATTTTTCCAAAAGCATCAAGTGCTGTCTTCACAACCTTCTCTCCAGCTTCCACAGAATCATAGTTAGCCACAGCTTTTCCCCCTTTCTTCCTTATTTCTTCAACAACATTATCAGCAGCTGTGGAGCTTTTTCCTATTCCCTTGAAGTCCCCTCCTAAATCATTCACAACTACCGATGCACCTCTTTCTGCAAAAGCCAGGGCATACGCTCGTCCCAATCCTCCCCCCGCACCGGTAACCAGCACCACCCGCCCGTCGAATCTCAGGTGCCCGTCCATAAGTCCTGCTCACAGCCACAAAACACCTATACAAACCCACACAGAGGCGTACAGGGCTCTGCGCTGCGGGAGAGGCGTGGCGAAGGGGGAGGTGGGGATAGGGCGGAGACCTAAAGAAG

>UoN.Pci.T.15658_UoN.Pci.T.15658.1

CCACCCCCCCGCCACCACCCCTGTTGGGTTAGCTAGTTTGCAAGGAGCCGCCTGACTCTTGAGGCCTCAGCACCAGAAGCTGCAGCAAACCCCAAGGAAGAAAAGGAACGTACTGGGAGTCTGCGGAGCTCGAAAGAGCCTGAGCCCAGTGCGGTCTGCGCCATGTCGGCTAATATGAGGGAGAGGTTCGACCAGTTCCTGCACCAGAAGAACTGCATGACCGACCTTCTGGCTAAGATCGAGAGCAAGACTGGGGTGAACAGATCCTACATAGCGCTTGGTATCATCGGCTTGGTGGCTTTGTACCTTGTGTTTGGCTATGGAGCCTCTCTGCTCTGCAACCTGATAGGATTTGGCTATCCAGCCTATGTCTCAATCAAAGCTATTGAGAGTCCCAACAAAGATGATGATACACAATGGTTGACCTACTGGGTCGTATATGGCGTCTTCAGTATAGCTGAATTCTTCTCTGATCTCTTCCTGTCATGGTTCCCTTTCTACTACATGCTGAAGTGTGGCTTCCTGTTGTGGTGTATGGCCCCCAGCCCTTCAAATGGTGCGGAGATGCTGTACAAGCGTGTCATCCGCCCTTTCTTCCTGAAGCACGAATCCCAAATGGACAGTGTGGTGAAGGATCTGAAAGACAAAGCCAAAGAGACTGCAGATGCCTTCACTAAAGAAGCCAAGAAAGCTACCGTGAACTTATTGGGTGATGAAAAGAAAAGTACCTAAACAGTGAACTGGATGAAAGCCCTCTTCTGTTACACACTTTACTCTCCTATTACAGCTCTGTTATGCTGGGGACTGTGGTATAGTAATAATAATAACATTGCCTTGAAAACCTTTTTGATCTATTAAAAAGGAATGTGTTGTAAGATTTTTGCTTACTTTCTTGCTGCTGTCTGTATAAGCAGTGAGCATTTTAATTTAATTTCATTCAATGCAGTGGACAGTGTTCAAAGTTTCTTGGGAATGTATTCTTTTCCTCTCTGAGCTAGGAAAGACATCTGTCATACCACAGTGAATACAAATAAAATTAAGTATCTCACAGGCTCTATGCTTTACTGGCTTCTCTGTGCATGTATCTTCCGTATTTATGGTTTGTGGGTTTTCATGTAATGTATGATTTTTATACTAGAATTCTTAACATTTTAAACCTTTTTGTTTGTGCACATGCAGAAAAATGTTTCAAAGGGTAGATTTAAACTATTATGCTAGAGAAGGTACAATTTAGCAATATGAATTTTGTAGCAATGACGTTACTCAGTGATTGCCTATGCTATTTGTAACTGTTCTGTGCAAAAAGTGAGAGCACTTATTTGGATAATTAGGAAAACCTAATAAAAACAGCATGATGTCACTGGTTAGTTTTTTCCTAGGATGATCTCTACTAAGAATGATGACATAGAAAGTGCTAGAAAAAATGGACAATAATGTAAGTAAATTATACAGTATAATGAAGTTTGTATAGCTTTGTCACCTTATTTGTGATTATGATGTCTAGCTTTTTCTTCCCAGAATTATAGATGCTGAATGCCAAGAAACCAAGAATAAGAATAGTGTGTTACTCATGGCAAGAATGGGAAAATTAACATGCATATCAATAACTAAGGGTCTGCTACTTCAGGATACCTAGGGTTTTCTGTATGTTTTCAAGTCTATTCTGGTTTGTGGGAATTTCACCTACTGCCATGTCTAGCCTTCCCTCAAGCTAGATGAGATTGTTTCTTAAGCTCTTCTCAGAAACTCTATGCCTGGTTTTCAATTATTCTCCTTTAGCCACTAACAAAGGACCTATAATTCTGACCAAATGCTGTATTCCTGTGTACAACTGAAATACCATATTCATTAGATCTAAAACCAATGATGGGAAGAATGGAACAAGTAGGAACTGACTCCTGCCAACTGAGCTTTAATTTTAATTATAGGTCTTTCCAAAACTTCCCTCCACTTTATTCCTCTGGCTTTTTAAATCAGATACAAGAGGCAACCTTATCCTAGAAATCATATTCCTGTAGCTTCCCATTAAATGTTCTAATTTCTCTTTTCTGTGAAATTATTTCCTAATATCAGTTCTGGCACAGACCAGAAGTTTAAAAAAATTGTCACATGATTATTGCAGGCCAAATATATTACTGACATATTGACAGCTTTTGTGGCTATAGCAAAAAAGCGCTGCAAGAAAACAAAAACTCGAAAGCTTTATAGGTGCATTTTGGCTTGGATAATGAGAAATTAACTACTCCTAAAACACCAATCAGTAAATTATTTTAAAAGTTATTGTGCTTAAGACTTTATATATATATGTATATATATATATATATATATATACATATATATATAAAGTCTTAAGCACAATAACTTTTAAAATAATTTACTGATTGGTGTTTTAGGAGTAGTTAATTTCTCATTATCCAAGCCAAAATGCACCTATAAAGCTTTCGAGTTTTTGTTTTCTTGCAGCGCTTTTTTGCTATAGCCACAAAAGCTGTCAATATGTCAGTAATATATTTGGCCTGCAATAATCATGTGACAATTTTTTTAAACTTCTGGTCTGTGCCAGAACTGATATTAGGAAATAATTTCACAGAAAAGAGAAATTAGAACATTTAATGGGAAGCTACAGGAATATGATTTCTAGGATAAGGTTGCCTCTTGTATCTGATTTAAAAAGCCAGAGGAATAAAGTGGAGGGAAGTTTTGGAAAGACCTATAATTAAAATTAAAGCTCAGTTGGCAGGAGTCAGTTCCTACTTGTTCCATTCTTCCCATCATTGGTTTTAGATCTAATGAATATGGTATTTCAGTTGTACACAGGAATACAGCATTTGGTCAGAATTATAGGTCCTTTGTTAGTGGCTAAAGGAGAATAATTGAAAACCAGGCATAGAGTTTCTGAGAAGAGCTTAAGAAACAATCTCATCTAGCTTGAGGGAAGGCTAGACATGGCAGTAGGTGAAATTCCCACAAACCAGAATAGACTTGAAAACATACAGAAAACCCTAGGTATCCTGAAGTAGCAGACCCTTAGTTATTGATATGCATGTTAATTTTCCCATTCTTGCCATGAGTAACACACTATTCTTATTCTTGGTTTCTTGGCATTCAGCATCTATAATTCTGGGAAGAAAAAGCTAGACATCATAATCACAAATAAGGTGACAAAGCTATACAAACTTCATTATACTGTATAATTTACTTACATTATTGTCCATTTTTTCTAGCACTTTCTATGTCATCATTCTTAGTAGAGATCATCCTAGGAAAAAACTAACCAGTGACATCATGCTGTTTTTATTAGGTTTTCCTAATTATCCAAATAAGTGCTCTCACTTTTTGCACAGAACAGTTACAAATAGCATAGGCAATCACTGAGTAACGTCATTGCTACAAAATTCATATTGCTAAATTGTACCTTCTCTAGCATAATAGTTTAAATCTACCCTTTGAAACATTTTTCTGCATGTGCACAAACAAAAAGGTTTAAAATGTTAAGAATTCTAGTATAAAAATCATACATTACATGAAAACCCACAAACCATAAATACGGAAGATACATGCACAGAGAAGCCAGTAAAGCATAGAGCCTGTGAGATACTTAATTTTATTTGTATTCACTGTGGTATGACAGATGTCTTTCCTAGCTCAGAGAGGAAAAGAATACATTCCCAAGAAACTTTGAACACTGTCCACTGCATTGAATGAAATTAAATTAAAATGCTCACTGCTTATACAGACAGCAGCAAGAAAGTAAGCAAAAATCTTACAACACATTCCTTTTTAATAGATCAAAAAGGTTTTCAAGGCAATGTTATTATTATTACTATACCACAGTCCCCAGCATAACAGAGCTGTAATAGGAGAGTAAAGTGTGTAACAGAAGAGGGCTTTCATCCAGTTCACTGTTTAGGTACTTTTCTTTTCATCACCCAATAAGTTCACGGTAGCTTTCTTGGCTTCTTTAGTGAAGGCATCTGCAGTCTCTTTGGCTTTGTCTTTCAGATCCTTCACCACACTGTCCATTTGGGATTCGTGCTTCAGGAAGAAAGGGCGGATGACACGCTTGTACAGCATCTCCGCACCATTTGAAGGGCTGGGGGCCATACACCACAACAGGAAGCCACACTTCAGCATGTAGTAGAAAGGGAACCATGACAGGAAGAGATCAGAGAAGAATTCAGCTATACTGAAGACGCCATATACGACCCAGTAGGTCAACCATTGTGTATCATCATCTTTGTTGGGACTCTCAATAGCTTTGATTGAGACATAGGCTGGATAGCCAAATCCTATCAGGTTGCAGAGCAGAGAGGCTCCATAGCCAAACACAAGGTACAAAGCCACCAAGCCGATGATACCAAGCGCTATGTAGGATCTGTTCACCCCAGTCTTGCTCTCGATCTTAGCCAGAAGGTCGGTCATGCAGTTCTTCTGGTGCAGGAACTGGTCGAACCTCTCCCTCATATTAGCCGACATGGCGCAGACCGCACTGGGCTCAGGCTCTTTCGAGCTCCGCAGACTCCCAGTACGTTCCTTTTCTTCCTTGGGGTTTGCTGCAGCTTCTGGTGCTGAGGCCTCAAGAGTCAGGCGGCTCCTTGCAAACTAGCTAACCCAACAGGGGTGGTGGCGGGGGGGTGG

>UoN.Pci.T.9515_UoN.Pci.T.9515.1

CTCCACGTGGCCTCCAGCAGAGCCTGCTCCCGGGGCCTCTTTAACTGGGCAGCGGAAGTAGCCGCTCCAGCGTCTCCCGCCAGCGCCCAATGGGCTGGTGCAAAGCGTCCTAAGGCCCCGTCACTTCCCGGCTAGGAAACCGGCAAGGCTTGAAGAGGAACGCCCGGGGGAGGGGGCGGGCGCCGAACCCACGCATCCCCAGCCCGGCCGTGGTCCCCTGCAGTCTCGGGGCCGGCACCAACATGCCCTTTGACTTCAGGAGATTTGACATCTACCGGAAGGTTCCAAAGGACCTCACGCAGCCAACGTACACTGGGGCTATCATCTCTGTCTGCTGTTGCCTCTTTATTCTGTTCCTCTTCCTCTCGGAGCTCACTGGATTCATAGCCACTGAAGTAGTGAACGAGCTATATGTCGACGATCCCGATAAAGACAGTGGAGGCAAAATAGATGTCAGTCTGAACATCAGTTTGCCGAATTTACACTGTGAATTGGTTGGACTGGACATCCAGGATGAGATGGGGAGACATGAAGTCGGACATATTGACAACTCGATGAAAATCCCTCTGAACAATGGTGAAGGCTGCCGGTTCGAGGGCCAGTTCAGCATCAACAAGGTCCCTGGCAACTTTCACGTATCAACACACAGTGCCACTGCACAGCCCCAGAATCCAGACATGACTCATGTCATCCACAAGCTGTCCTTTGGAGATACATTGCAGGTTCAGAACATTCATGGAGCATTCAACGCTCTGGGAGGAGAAGATAAACTCACTTCTAACCCCCTTGCATCCCATGATTACATCTTGAAGATCGTCCCCACGGTGTACGAAGATAAGAGTGGAAAACAGCGGTACTCATACCAATACACGGTAGCCAATAAGGAATATGTTGCCTACAGTCACACTGGTCGCATCATCCCTGCCATTTGGTTTCGCTATGACCTGAGCCCCATCACGGTGAAGTACACAGAGCGGCGACAGCCACTGTACAGGTTCATCACCACGATCTGTGCCATCATTGGAGGCACTTTCACGGTGGCCGGAATCCTGGACTCCTGCATTTTCACAGCATCTGAGGCCTGGAAGAAGATTCAACTCGGAAAGATGCAGTAACCCTGTGACTCCGACTCATTTTGCCAGAGACAGCAATGCAGGATCTCAGACTCATCACTCTAACAAATATATTTCCTTTTTCCCTCTATCAGAGTTGATATTCAGTTTTGTTGGTTGAAGTTACAGAGTGGGTAGTTGCTACACCAAGGTTTCTCTTCCCACACCATACTCCCATTTCAGGGGGAATTCCAACTTACACATTCTCTTCTTTTGTTTTTGAAAAGAAAATACTGACTATTTTTAGGAAGAAAGTCGACTGCCAGTGCCAAGAATCGTGGTGCTCCCTGCCCTGGGGGGTAGGGGGTAAGGGTAGAAATAATTTCCTAAGAAGCAAACAGGAGGGCTCAAGGGTTGTTGTGATAGAGTTTCATTGGGAAGTAACAAGAAAGTCCAGGAGTGACAGGCTCTCAGTATCTTTCGGACAGTTACCTGGCTCACTTCTCCTGAGCATCAATCTGAAGTAAGTGGAAAATGAGGAAAGCCCTTTTGGAGAGAGAAAACATTTGGTTTCTTGCCCAGGGATACCATGTGAGACCTCTCCCTATGCCCATCCCTAACATCACGCTTTGGCGATATGCACTTGGCTGAACTATATTAAGGTACCAATTGAACTTATGTTGTATAGGGCTCGAGACCCCTGTTTCTCTCTCTCTCTCTCTCTCTCCCTTCCTTTTTAAAAGTGGGTTTCAGGCTGCCAAGAACCACAGCCCCCAAGAGACTGAAGTCTGTTCACCACTGGTGCAGCCTAGCCAACGGCTGCAAAATTAAGTTTTCCTCCCATTTTCCCCATCACTCTTAACCTCCCACCAATGTGCCTCAGTCCTAACCAAAGGACAACACCTCTTAGCTAAGGAAACAGCCTAGCCTCTGGACCCACCCCACCTTTCCATTGGCACCTCAGCCCTTTGTGTTGACAAAGTGGTGGTGAGTGGTTGGGCGTCTTACAGGGTTTAGATCTGGAAGAAGCCTTAGGGACCACCTAGTTCAACCTCCCTCATTTTACAGAGGAAGAAACTGAGGCCTGGAAATGGGAAATGCCATGCCCAAGGGCATACAGCTAATAAACTGTAGGAGAGAGAAAACTCATTTCCTCGTCCTTTAAATCCAGTGTTCTTTCCACCGCTTGACAATATTCTCTATGTAGGCCCAGTGGCTTTGCTCAGGCCCAGAGAGTGACCAGTAAGCATCATTCCCAATGACATGGAGACTTTTCCCCATTGGTCCTAGTTTTGTGTGACATCTCGGGGGCAACAAGCTCATTGCTAATATGAAAGTAGAGTCAGCGATTTTCCCTGGTGACATCCTAGTTCAAGCTGTTTTTGTTTGCTTTGTTCAGGTGTTATTTTGGTTTGGTTTGTTTTCACCATCCCCGCCAATGGCAAACAGAAAAGGGACTGTCCCGGCATGGGCCCGCAGCTCCTGCCTGCCTCCCTTATCAGACGCTTGCTCTGTGGTTTCTTTCACCAGCCCCTCCAGCCACTAAGACTAACTCGAGACAGTCATGCAGCTGGCGAGGCAGCAGCAGTTCAGAGCTGTCTCTGCTGCTGCTGCTGCTGCTGCTGTTTATACTTCCTTTCATCAGAAGATTCTCCTTTTAGAGCAGAACAAGGAAGGACGAGCAAGCCAGGCCAGGGAGGGATGCAGTGAAGGGAATTTCAAATATTCTATGTTGATCCCAGAGAGAGCACCACCCTTCCATTTTCCATGCACACCAAGAATTCTTCGCGTGCTTTGGTGATTCCGGATAATGTCCAGTTGGATTTATTTTTTAACAAGGCTTTTGCCATTTATTATTTTAAGCCCCTAGTGCCAATTAGAAAAACTCCCCCCCCTTTTTTTATTATGAGTTCATTAATATTGTAAGTGTAACTGGATTTTGTCATTTCTTGTATCTGTCACTGTTTGGATGGATTTTATATATATATTTTTTTTCCATTTACAAAATTCAGAAATGGAAATGATCCCTTGTGTATGGGGGAATGAGGGGGTTGGGGTGTGTGTGTGTGTGTGTGTGTGTAGACCTGGGGAGGGAATAATTTAGTGGGGACTGAGCCCTGGCTGAAGGGTGGGGGCTAGGGCTGGGGTCTCTGCCTCACCTGTCACAATCAGCATCAGAGGGTCACTGGACTCTGTCCAGCCAGATGAGGTCCAGTAGTGACAGCGGTAAAGACCAGCAGCTTCTACTCTCATTTTCCCCAGAAAGAATGAAGCTTTATTCCCATTAGGGTAGGACAACATAAACCAAAAGGTTCCCACTTTCTCCAGCTTGTAACCTACAGCCCCAGAAGGGCCCTGACACCAGACAGTCACAAGTTGCCCCTCAGGCATCAGATGGTCTGGCTTTGCCCAGAGGGAGGGTTTGGGGAGTAGATCTGGAAGGAAGTTAGAGCCTGGAGTTCCAGAGGGTTCCAATGCCTCCTGCCCCCAGGCCCCTCCCAGAGTCACTCCAGTAGGTTAGCCCAGACCCTCCAGTCTGTCCTCCCTTGGGGGGACAGGTGAGTAGAGAGAGAGAGAGAAAGACAGACAGACAGACAGAAAGAGAGACAGAGAGAGAGAGAGAGAGAGACAGAAACAGAGACAGAGACAGACAGAGAGATAGAGACATGGAGACACAGATACAGACAAAAAGAGAGAATGGGAGGGGGTTGGTTGGGGGGAGAGAACTCACGGTCCTGTGCCTTGATCCTCCCACTGAGACACAGTCCTGTCAGAGAGGGTCCCATCAGCCCTGCCCAGAATCCTCAGCTTCCCTACCAAAGCTCTGGGTGGGGGATGGGAAGTGGGGGGAGGCATGCTGCTGGACATCAGGGCACCTGGGTTGGGGTTCTAGCCTTGGGTGAACTCAGGGGAGTCATTTCTTCACTCTGGGCTTCTTTCTTTCATTGTCAAATGAGGGAGAGAAGAGAAGGCTGCTCTTGCCCTTGACAGCCCTGTATCAGGAACCTGAAATTCTCCCTGCCCAGACTCACCGAGGTACAGCAGGGCAGGGAG

>UoN.Pci.T.9515_UoN.Pci.T.9515.2

CTCCACGTGGCCTCCAGCAGAGCCTGCTCCCGGGGCCTCTTTAACTGGGCAGCGGAAGTAGCCGCTCCAGCGTCTCCCGCCAGCGCCCAATGGGCTGGTGCAAAGCGTCCTAAGGCCCCGTCACTTCCCGGCTAGGAAACCGGCAAGGCTTGAAGAGGAACGCCCGGGGGAGGGGGCGGGCGCCGAACCCACGCATCCCCAGCCCGGCCGTGGTCCCCTGCAGTCTCGGGGCCGGCACCAACATGCCCTTTGACTTCAGGAGATTTGACATCTACCGGAAGGTTCCAAAGGACCTCACGCAGCCAACGTACACTGGGGCTATCATCTCTGTCTGCTGTTGCCTCTTTATTCTGTTCCTCTTCCTCTCGGAGCTCACTGGATTCATAGCCACTGAAGTAGTGAACGAGCTATATGTCGACGATCCCGATAAAGACAGTGGAGGCAAAATAGATGTCAGTCTGAACATCAGTTTGCCGAATTTACACTGTGAATTGGTTGGACTGGACATCCAGGATGAGATGGGGAGACATGAAGTCGGACATATTGACAACTCGATGAAAATCCCTCTGAACAATGGTGAAGGCTGCCGGTTCGAGGGCCAGTTCAGCATCAACAAGGTCCCTGGCAACTTTCACGTATCAACACACAGTGCCACTGCACAGCCCCAGAATCCAGACATGACTCATGTCATCCACAAGCTGTCCTTTGGAGATACATTGCAGGTTCAGAACATTCATGGAGCATTCAACGCTCTGGGAGGAGAAGATAAACTCACTTCTAACCCCCTTGCATCCCATGATTACATCTTGAAGATCGTCCCCACGGTGTACGAAGATAAGAGTGGAAAACAGCGGTACTCATACCAATACACGGTAGCCAATAAGGAATATGTTGCCTACAGTCACACTGGTCGCATCATCCCTGCCATTTGGTTTCGCTATGACCTGAGCCCCATCACGGTGAAGTACACAGAGCGGCGACAGCCACTGTACAGGTTCATCACCACGATCTGTGCCATCATTGGAGGCACTTTCACGGTGGCCGGAATCCTGGACTCCTGCATTTTCACAGCATCTGAGGCCTGGAAGAAGATTCAACTCGGAAAGATGCAGTAACCCTGTGACTCCGACTCATTTTGCCAGAGACAGCAATGCAGGATCTCAGACTCATCACTCTAACAAATATATTTCCTTTTTCCCTCTATCAGAGTTGATATTCAGTTTTGTTGGTTGAAGTTACAGAGTGGGTAGTTGCTACACCAAGGTTTCTCTTCCCACACCATACTCCCATTTCAGGGGGAATTCCAACTTACACATTCTCTTCTTTTGTTTTTGAAAAGAAAATACTGACTATTTTTAGGAAGAAAGTCGACTGCCAGTGCCAAGAATCGTGGTGCTCCCTGCCCTGGGGGGTAGGGGGTAAGGGTAGAAATAATTTCCTAAGAAGCAAACAGGAGGGCTCAAGGGTTGTTGTGATAGAGTTTCATTGGGAAGTAACAAGAAAGTCCAGGAGTGACAGGCTCTCAGTATCTTTCGGACAGTTACCTGGCTCACTTCTCCTGAGCATCAATCTGAAGTAAGTGGAAAATGAGGAAAGCCCTTTTGGAGAGAGAAAACATTTGGTTTCTTGCCCAGGGATACCATGTGAGACCTCTCCCTATGCCCATCCCTAACATCACGCTTTGGCGATATGCACTTGGCTGAACTATATTAAGGTACCAATTGAACTTATGTTGTATAGGGCTCGAGACCCCTGTTTCTCTCTCTCTCTCTCTCTCTCCCTTCCTTTTTAAAAGTGGGTTTCAGGCTGCCAAGAACCACAGCCCCCAAGAGACTGAAGTCTGTTCACCACTGGTGCAGCCTAGCCAACGGCTGCAAAATTAAGTTTTCCTCCCATTTTCCCCATCACTCTTAACCTCCCACCAATGTGCCTCAGTCCTAACCAAAGGACAACACCTCTTAGCTAAGGAAACAGCCTAGCCTCTGGACCCACCCCACCTTTCCATTGGCACCTCAGCCCTTTGTGTTGACAAAGTGGTGGTGAGTGGTTGGGCGTCTTACAGGGTTTAGATCTGGAAGAAGCCTTAGGGACCACCTAGTTCAACCTCCCTCATTTTACAGAGGAAGAAACTGAGGCCTGGAAATGGGAAATGCCATGCCCAAGGGCATACAGCTAATAAACTGTAGGAGAGAGAAAACTCATTTCCTCGTCCTTTAAATCCAGTGTTCTTTCCACCGCTTGACAATATTCTCTATGTAGGCCCAGTGGCTTTGCTCAGGCCCAGAGAGTGACCAGTAAGCATCATTCCCAATGACATGGAGACTTTTCCCCATTGGTCCTAGTTTTGTGTGACATCTCGGGGGCAACAAGCTCATTGCTAATATGAAAGTAGAGTCAGCGATTTTCCCTGGTGACATCCTAGTTCAAGCTGTTTTTGTTTGCTTTGTTCAGGTGTTATTTTGGTTTGGTTTGTTTTCACCATCCCCGCCAATGGCAAACAGAAAAGGGACTGTCCCGGCATGGGCCCGCAGCTCCTGCCTGCCTCCCTTATCAGACGCTTGCTCTGTGGTTTCTTTCACCAGCCCCTCCAGCCACTAAGACTAACTCGAGACAGTCATGCAGCTGGCGAGGCAGCAGCAGTTCAGAGCTGTCTCTGCTGCTGCTGCTGCTGCTGCTGTTTATACTTCCTTTCATCAGAAGATTCTCCTTTTAGAGCAGAACAAGGAAGGACGAGCAAGCCAGGCCAGGGAGGGATGCAGTGAAGGGAATTTCAAATATTCTATGTTGATCCCAGAGAGAGCACCACCCTTCCATTTTCCATGCACACCAAGAATTCTTCGCGTGCTTTGGTGATTCCGGATAATGTCCAGTTGGATTTATTTTTTAACAAGGCTTTTGCCATTTATTATTTTAAGCCCCTAGTGCCAATTAGAAAAACTCCCCCCCCTTT

>UoN.Pci.T.9877_UoN.Pci.T.9877.1

GGGTGCTGCGGCAGGAGGGAAGATGGCGGACGAAGAGAAGCTGCCGTCAGGCTGGGAGAAGCGCATGAGCCGCAGCTCAGGACGAGTCTATTATTTCAACCACATCACCAATGCCAGCCAGTGGGAGCGTCCAAGTAGTGTGGGCAAGAATGGTCAAGGTGAACCTACTAAGGTCCGATGCTCACATCTGCTGGTGAAACACAGCCAGTCTCGAAGGCCCTCATCCTGGCGGCAGGAAAAGATCACCAGGAGCAAAGAGGAGGCACTGGAGCTGATCAATGGATACATCCAGAAGATTAAATCAGGGGAAGAAGATTTTGAGACTCTGGCATCGCAGTTCAGTGACTGCAGTTCAGCCAAAGCCAGGGGGGACCTGGGCACCTTTGGAAGAGGTCAGATGCAGAAACCATTTGAAGATGCCTCCTTCGCACTGAGGACGGGTGAGATGAGCGGCCCAGTCTTCACAGACTCAGGGATCCACATCATCCTTCGTACAGAGTAGGAGTGCCTTGGGTTGGGGATCAAGGGCAGGGGAAGACTGACCTCTGTTCACCTGCCCCCAGACTCTCTCCATCCAGTCTACAGCTGCCTCTAAGAGCTGAGGTGGCCCCAAGGCATGGGGTGGGGGGCAGGCCAGGGTTGGGGAACCCTTTACTTCCTATTTGGGGGCATCTTTTCCATTTTGTTTCAGCCTTGATGGTTCCTCCCTCTGAAAGGTCAGTGCTGGGACCCCTAGTGTTGCTGGTGTCCCTGCTCCAGCTGAAAATACACAATATTTATTGTCCCCAGACTCTCAGATGCAGAGATCGGGGTGAGGATGAGCCCTAGTTTAGGTCTCCTCACTGCCCTCTCACCTCCACTACCTGAGGGCCTCTGTTAAATCACTCCACTGTGGTCAACAATGGCCCCTACCTAGCTCCACCTAACAGCCACTCTGGCTTCCGTTCAGTCTGATCCTGCCTTCTGCATCTTAAGGAATTATGGGTCAGTAGCTGGAAGGAAACCACTTGGAAATGAGAGGGGACCCTCAGCTGGGAGGGGAAAGGGCCTGGGTTATCTGAACTCCTGTTGCAGCCAGAGCATCTTGGAGGGAGACTAAATTGCTGTTTCTGCTGCATTGCTTTGTTTAGTTAAAAGAAAATCAAAATCCTCAGCTTTAAGGCCAATGCAAGGCAGTTGTATAGTTCCTGTTCTTATCCCCTGGCTGTCCAGATGTCCCCTATTAAACCTTGTAAACAAGTTTCTACTGTGTCTCATTACCTCCTCCCCACTCCAGGTTCACAAAAACCAGAATTCATGACCAAAACACACAGCAGCACCATCATTGAAGTTATTTTATTAGCTTTTAAAAAGGGAAGAGAGGTGCAGATTTGGGGGATGGGGTGTTGCTACCCTTATTGGTAATAAAGCTCCAAGTTCATTCCATCGTGGATCTCATAGTCCCCTAGAGTCACATGGTCCTTGAAGATTGTGTACCATTTCTTCAGAACAATCTTGTTCCAACGGGTGCCTGTCTGAGCCGCAATCAGTTTCTTCAGATCTCCTATCGTGTCATCCGTGTTGCACTTCACACGGACCTTCTTGCCCAGCCGGTCGTTACACACAACCTCGATCATGGTTCCCTTCGCCTTCAGACACAGCCCCTGCCTCACTTCGCACGACAGCGCCGCCCGCACTTTACGGCCA

>UoN.Pci.T.9877_UoN.Pci.T.9877.1

GGGTGCTGCGGCAGGAGGGAAGATGGCGGACGAAGAGAAGCTGCCGTCAGGCTGGGAGAAGCGCATGAGCCGCAGCTCAGGACGAGTCTATTATTTCAACCACATCACCAATGCCAGCCAGTGGGAGCGTCCAAGTAGTGTGGGCAAGAATGGTCAAGGTGAACCTACTAAGGTCCGATGCTCACATCTGCTGGTGAAACACAGCCAGTCTCGAAGGCCCTCATCCTGGCGGCAGGAAAAGATCACCAGGAGCAAAGAGGAGGCACTGGAGCTGATCAATGGATACATCCAGAAGATTAAATCAGGGGAAGAAGATTTTGAGACTCTGGCATCGCAGTTCAGTGACTGCAGTTCAGCCAAAGCCAGGGGGGACCTGGGCACCTTTGGAAGAGGTCAGATGCAGAAACCATTTGAAGATGCCTCCTTCGCACTGAGGACGGGTGAGATGAGCGGCCCAGTCTTCACAGACTCAGGGATCCACATCATCCTTCGTACAGAGTAGGAGTGCCTTGGGTTGGGGATCAAGGGCAGGGGAAGACTGACCTCTGTTCACCTGCCCCCAGACTCTCTCCATCCAGTCTACAGCTGCCTCTAAGAGCTGAGGTGGCCCCAAGGCATGGGGTGGGGGGCAGGCCAGGGTTGGGGAACCCTTTACTTCCTATTTGGGGGCATCTTTTCCATTTTGTTTCAGCCTTGATGGTTCCTCCCTCTGAAAGGTCAGTGCTGGGACCCCTAGTGTTGCTGGTGTCCCTGCTCCAGCTGAAAATACACAATATTTATTGTCCCCAGACTCTCAGATGCAGAGATCGGGGTGAGGATGAGCCCTAGTTTAGGTCTCCTCACTGCCCTCTCACCTCCACTACCTGAGGGCCTCTGTTAAATCACTCCACTGTGGTCAACAATGGCCCCTACCTAGCTCCACCTAACAGCCACTCTGGCTTCCGTTCAGTCTGATCCTGCCTTCTGCATCTTAAGGAATTATGGGTCAGTAGCTGGAAGGAAACCACTTGGAAATGAGAGGGGACCCTCAGCTGGGAGGGGAAAGGGCCTGGGTTATCTGAACTCCTGTTGCAGCCAGAGCATCTTGGAGGGAGACTAAATTGCTGTTTCTGCTGCATTGCTTTGTTTAGTTAAAAGAAAATCAAAATCCTCAGCTTTAAGGCCAATGCAAGGCAGTTGTATAGTTCCTGTTCTTATCCCCTGGCTGTCCAGATGTCCCCTATTAAACCTTGTAAACAAGTTTCTACTGTGTCTCATTACCTCCTCCCCACTCCAGGTTCACAAAAACCAGAATTCATGACCAAAACACACAGCAGCACCATCATTGAAGTTATTTTATTAGCTTTTAAAAAGGGAAGAGAGGTGCAGATTTGGGGGATGGGGTGTTGCTACCCTTATTGGTAATAAAGCTCCAAGTTCATTCCATCGTGGATCTCATAGTCCCCTAGAGTCACATGGTCCTTGAAGATTGTGTACCATTTCTTCAGAACAATCTTGTTCCAACGGGTGCCTGTCTGAGCCGCAATCAGTTTCTTCAGATCTCCTATCGTGTCATCCGTGTTGCACTTCACACGGACCTTCTTGCCCAGCCGGTCGTTACACACAACCTCGATCATGGTTCCCTTCGCCTTCAGACACAGCCCCTGCCTCACTTCGCACGACAGCGCCGCCCGCACTTTACGGCCA

>UoN.Pci.T.9945_UoN.Pci.T.9945.1

GGCGGGAGTCCCCCGCGCTGCAGGCGATTGGACGATGCTGCGGGAGGCGGTGACTGATTGGCTGAGGCCCGCCCCTTCCGTAAGTGCCTAGCAGCGGCCTGGATCCGGCAGCGGCGGTCGCGGCGGCTCGGAGCGCTCTAGTTACCTCTCCTCACCAAACCTCGGCCGGGTTAGGTCCGCTTGAGTGTAGGCGCCATGAACATTCGCAACGCACGGCCAGAAGATCTGATGAATATGCAGCATTGTAACCTGCTGTGCCTTCCAGAAAACTATCAGATGAAATACTATTTCTATCACGGTCTATCATGGCCTCAGCTCTCTTACATTGCTGAAGATGAGAATGGAAAGATTGTGGGCTACGTCCTGGCCAAGATGGAGGAAGATCCAGATGATGTCCCCCATGGACATATCACATCCTTGGCTGTAAAGCGGTCCCACCGCCGGCTAGGTCTAGCACAGAAGCTTATGGACCAGGCCTCAAGGGCCATGATCGAAAACTTCAATGCAAAGTATGTCTCCCTGCATGTCAGAAAAAGTAACCGCGCAGCTCTGCATCTGTATTCCAACACCCTTAACTTTCAGATTAGTGAGGTGGAGCCCAAATACTATGCTGACGGAGAAGATGCCTATGCTATGAAGCGTGATCTGACTCAGATGGCAGAAGAGCTGAGGAAGCAGCTGGAAGTGAAGGAGAAGGGCAAACATGTGGTGCTGGCCTCCCTGGAGAACAAGATGGATCCCAAAGTGAACCATGTGGGAGACTGCTGCCGTGAGGAGAAGGGCCTAGGCCCCTCCACCGAGGACAGCAGCGGCGGCGACAGCAAAGACCTGAGCGAGGTCAGCGAGACCACGGAGAGCACCGATGTCAAGGACAGCTCGGAGGCCTCTGACTCAGCCTCCTAGAGTCCCCAGGCTGGGATTCTGCCGTTCATAAGCAACCCCCATCTCTCTGTCTCTCTTCCCCTTATTCCCCATCAGTTCTTCCAATTAAATAATTTGGGGCCGTGGGGGTTGTGGATTTCTGTGAAAAAAAAAAAAAA

>UoN.Pci.T.9945_UoN.Pci.T.9945.1

GGCGGGAGTCCCCCGCGCTGCAGGCGATTGGACGATGCTGCGGGAGGCGGTGACTGATTGGCTGAGGCCCGCCCCTTCCGTAAGTGCCTAGCAGCGGCCTGGATCCGGCAGCGGCGGTCGCGGCGGCTCGGAGCGCTCTAGTTACCTCTCCTCACCAAACCTCGGCCGGGTTAGGTCCGCTTGAGTGTAGGCGCCATGAACATTCGCAACGCACGGCCAGAAGATCTGATGAATATGCAGCATTGTAACCTGCTGTGCCTTCCAGAAAACTATCAGATGAAATACTATTTCTATCACGGTCTATCATGGCCTCAGCTCTCTTACATTGCTGAAGATGAGAATGGAAAGATTGTGGGCTACGTCCTGGCCAAGATGGAGGAAGATCCAGATGATGTCCCCCATGGACATATCACATCCTTGGCTGTAAAGCGGTCCCACCGCCGGCTAGGTCTAGCACAGAAGCTTATGGACCAGGCCTCAAGGGCCATGATCGAAAACTTCAATGCAAAGTATGTCTCCCTGCATGTCAGAAAAAGTAACCGCGCAGCTCTGCATCTGTATTCCAACACCCTTAACTTTCAGATTAGTGAGGTGGAGCCCAAATACTATGCTGACGGAGAAGATGCCTATGCTATGAAGCGTGATCTGACTCAGATGGCAGAAGAGCTGAGGAAGCAGCTGGAAGTGAAGGAGAAGGGCAAACATGTGGTGCTGGCCTCCCTGGAGAACAAGATGGATCCCAAAGTGAACCATGTGGGAGACTGCTGCCGTGAGGAGAAGGGCCTAGGCCCCTCCACCGAGGACAGCAGCGGCGGCGACAGCAAAGACCTGAGCGAGGTCAGCGAGACCACGGAGAGCACCGATGTCAAGGACAGCTCGGAGGCCTCTGACTCAGCCTCCTAGAGTCCCCAGGCTGGGATTCTGCCGTTCATAAGCAACCCCCATCTCTCTGTCTCTCTTCCCCTTATTCCCCATCAGTTCTTCCAATTAAATAATTTGGGGCCGTGGGGGTTGTGGATTTCTGTGAAAAAAAAAAAAAA

>UoN.Pci.T.9945_UoN.Pci.T.9945.1

GGCGGGAGTCCCCCGCGCTGCAGGCGATTGGACGATGCTGCGGGAGGCGGTGACTGATTGGCTGAGGCCCGCCCCTTCCGTAAGTGCCTAGCAGCGGCCTGGATCCGGCAGCGGCGGTCGCGGCGGCTCGGAGCGCTCTAGTTACCTCTCCTCACCAAACCTCGGCCGGGTTAGGTCCGCTTGAGTGTAGGCGCCATGAACATTCGCAACGCACGGCCAGAAGATCTGATGAATATGCAGCATTGTAACCTGCTGTGCCTTCCAGAAAACTATCAGATGAAATACTATTTCTATCACGGTCTATCATGGCCTCAGCTCTCTTACATTGCTGAAGATGAGAATGGAAAGATTGTGGGCTACGTCCTGGCCAAGATGGAGGAAGATCCAGATGATGTCCCCCATGGACATATCACATCCTTGGCTGTAAAGCGGTCCCACCGCCGGCTAGGTCTAGCACAGAAGCTTATGGACCAGGCCTCAAGGGCCATGATCGAAAACTTCAATGCAAAGTATGTCTCCCTGCATGTCAGAAAAAGTAACCGCGCAGCTCTGCATCTGTATTCCAACACCCTTAACTTTCAGATTAGTGAGGTGGAGCCCAAATACTATGCTGACGGAGAAGATGCCTATGCTATGAAGCGTGATCTGACTCAGATGGCAGAAGAGCTGAGGAAGCAGCTGGAAGTGAAGGAGAAGGGCAAACATGTGGTGCTGGCCTCCCTGGAGAACAAGATGGATCCCAAAGTGAACCATGTGGGAGACTGCTGCCGTGAGGAGAAGGGCCTAGGCCCCTCCACCGAGGACAGCAGCGGCGGCGACAGCAAAGACCTGAGCGAGGTCAGCGAGACCACGGAGAGCACCGATGTCAAGGACAGCTCGGAGGCCTCTGACTCAGCCTCCTAGAGTCCCCAGGCTGGGATTCTGCCGTTCATAAGCAACCCCCATCTCTCTGTCTCTCTTCCCCTTATTCCCCATCAGTTCTTCCAATTAAATAATTTGGGGCCGTGGGGGTTGTGGATTTCTGTGAAAAAAAAAAAAAA

>UoN.Pci.T.9914_UoN.Pci.T.9914.1

TTTTTTTTTGCCACACAAAAGATTTCACCATTTATTTCCACAACCCATATGTTACATAACATACATTCCCAACTGGGAGCTCTCCCTGAGTCACACAGCGACCAGGGAACACTGGGCATGGCACGAGGCACACATCAATGCAGCTAGGCCTGAGGGCCTAGTCCCGCCAGCGATGTCCACACTGCGCATTGCAGCACTTGTAGAAAGTGGTCATGGGCTCATCTGCAGAGCGAGTCTGAAGCTGCATGAAGTAGGCTCGAGGGTGTTCACACTTGGGGCACGGCTCTGCAGTGGAGTCAACGTTCTCCCAGGCAGCGGCTCCCCCTAGTACGTCATCCACTTCTTTCAGCTTTGGATACTTCCGATTCGTTACCTTCCTGGTGATGTTGTGCACGTATGGGCACGTGTTGCAAGCGAAGCGGTGGCAGCGCTGCCCCTCCTCCACGATCAGCCCGTTCCCGCAGCCCGGGCAGAAGAGCAGCATTTTGACAACGGTTCACCAGCCTCCACCATCGGACGGCAATTCGACCTCATTAATCCGCGACAG

>UoN.Pci.T.9948_UoN.Pci.T.9948.1

GCGAGGGAGGAGGAAGGAGCGGCGTCCCCGGGCCCGGCACCTACGCGGAGGGAGGACCCCAGGCCGGCTCGGGAGTCTCAGAGGAGCGGAGCGCAGCGCAGCCTTAAGCCCCCAGGCTGGGCTCGCCTTCGCCGGCACCATGATCGCCGCGCAGCTGCTCGCCTATTACTTTACCGAATTAAAGGATGACCGCATCAAGAAGATCGATAAGTATCTTTATTCCATGCGGCTCTCAGATGAAATGCTTAATGAGATCACAAAACGATTTAAGAAAGCTATGATAAACGGCCTCTCCAGAAATTATAATATAACTGCTTCTGTGAAGATGTTGCCAACATTTGTGAGATCCATTCCTGATGGCTCAGAAAAAGGAGATTTCATTGCTCTGGATCTTGGTGGGTCTTCCTTTCGAATTCTACGAGTGCAAGTGTCTCATGAGAAAAATCAGACTGTTCAGATGGAGTCAGAGGTTTATGAAACACCGGAGAATATAATGAATGGCAGTGGAACCCAGCTTTTTGATCATGTTGCTGAATGCCTGGGTGACTTCATGGAGAAAAAGAATATCAAAGACAAGAATTTACCTGTTGGATTTACCTTTTCCTTCCCCTGCCGACAGACGAAAATTGATGAGGCTGTCCTGATCACCTGGACCAAGCGCTTTAAAGCCAGCGGTGTGGAGGGATCCGATGTGGTCCAGCTGCTCAGCAAAGCTATCAAGAAACGAGGGGATTACGAAGCCAACATCGTGGCTGTCGTGAATGACACCGTCGGGACGATGATGACCTGCGCCTTTGACGACCAGCAGTGCGAAGTGGGCCTGATCATCGGTACTGGCACCAACGCCTGCTACATGGAAGAGATGCGGCACATCGACGTGGTGGAAGGGGATGAGGGGCGCATGTGCATCAACACGGAGTGGGGGGCCTTCGGGGACGACGGCAGCCTCAACGACTTCCGCACAGAGTTTGACAAGGAGATCGACCGGGGATCCCTCAACCCTGGGAAGCAGCTGTTTGAGAAGATGGTCAGTGGCATGTACATGGGGGAGCTCGTCAGACTGATTATTGTGAAGATGGCAAAGGGCGGCCTCCTGTTTGAAGGGCGGATCACGCCTGAGCTGCTCACCAGAGGGAAATTTGAAACCAAGAATGTGTCAGCCATTGAGAAAACTAAGGAAGGCCTCCAAAGAGCCCAAGAAATCCTGACCAACCTGGGCTTGGAGCCATCCCCTGAAGATTGCATCTCTGTCCAGCATGTGTGTGCCATTGTCTCACACCGATCGGCCAACCTGGTGGCTGCCTTGCTGGCCGGAATCTTGGCTCGCCTGAAAGAAAACAAGGGCACGACTCGGCTGCGGACCACCGTTGGTGTCGATGGGACGCTTTACAAGTTTCATCCACAGTATTCCCGTCGATTCCACAAGACCCTGCGGCGCTTGGTCCCCGACTGCGATGTGCGCTTCATGCTCTCCGAGAGCGGGAGCGGTAAGGGGGCCGCCATGGTGACCGCCGTGGCCTATCGGCTGGCCGAGCAGCACAGGCAGATGGAGGAGATACTGGCCGCCTTCAAACTCAGCACTGAGCAGCTGATGCAGGTGAAGGATAGGATGAGAATAGAGATGGATCGTGGGTTACAGAAGAAGACCAATGAATCGGCCACGGTGAAGATGCTGCCGTCCTTCGTCTGCGGCACCCCTGATGGGACCGAGCATGGAGACTTCTTGGCCCTGGACCTTGGCGGCACAAACTTCCGGGTCCTGCTGGTGAAGATCCGCAGCGGGAAAAAGAGAAGTGTGGAAATGCACAACAAGATCTACGCCATCCCTGAGGAAGCCATGCAGGGCACCGGGGAGGAGCTCTTTGACCACATCGTCTCCTGTATCTCTGACTTCCTGGATTACATGGGCATCAAAGGTGCCCGGCTGCCTCTAGGCTTCACCTTTTCCTTCCCTTGCCGACAGACAAGCCTGGATGGGGGCGTCTTGGTCACGTGGACTAAGGGATTTAATGCGTCCAACTGTGAAGGACACGATGTGCCCACCTTGCTGAGAGAAGCAATTAAAAGGAGAGAGGAATTTGACCTGGATGTTGTGGCCTTGGTCAATGACACGGTGGGCACCATGATGACCTGTGCCTATGAAGAGCCGACTTGCGAGATTGGACTCATCGTAGGGACCGGAAGCAATGCCTGTTACATGGAAGAGGCGAAGAATATTGAGATGGTGGAGGGCGACGTGGGCCGGATGTGTGTCAACATGGAGTGGGGCGCCTTTGGGGACAACGGATGCCTGGACGACATCAGGACCGAATTTGACAAAATCGTGGATGATTATTCCCTCAATCCTAACAAGCAAAGGTTTGAGAAGATGATTAGTGGTATGTACCTGGGAGAGATCGTCCGAAACATTTTAATCGACTTCACTCGAAAGGGTTTCCTGTTCCGAGGCCAGATCTCTGAAACCTTGAAAACCCGGGGAATCTTTAAGACCAAATTCCTATCACAGATTGAAAGCGATAGGTTAGCCCTGCTCCAGGTCCGAACAATCCTGCAGGATCTGGGCCTGAACAGCACCTGTGACGACAGTATCCTCGTCAAAATGGTCTGCGGGATCGTGTCCAGGAGGGCTGCCCAGCTCTGCGGGGCTGGGATGGCGGCCGTCGTGGAAAAGATCAAGGAGAACAGGGGCCTGAAAACACTGAATGTGGCCGTGGGCGTCGACGGGACGCTCTACAAACTCCATCCTCACTTCTCCAGGGTCATGCATCAGACAGTAAGCGAGTTGGCCCCCAACTGTAAGGTGAAATTCCTCCTCTCAGAAGATGGCAGTGGGAAGGGAGCAGCGCTCATCTCAGCAGTCGGGGTGAGGCTGCGGGAGGCCAAGGCCAAGGCCAGCTAAGAGCCCTGCCCTCGGAGGAGCCCCTGCCCCCTCCTCTCTCGTTCTCTCTTTCTCTCTGTCTCTCTCTGCACTTCTCCATCTTCCTTCCTCGGGTAGCCTGAACTGAACGCACACCAGACCCTGCCGATGTATGGAGCCCTGTACACCTCAAGGCATAAAGAACTTGGGTGCAAAATTGAGTGTGTGCTGTGGATAATATCTCTCTCCCCGGGAGCCCCATCCTTCCCACCCCTCCCTGCTGCCTTCTCCCCTCCCCTGTCACTGTTCTGCATGTTTTCATTTCCATTGGGTTGTCCAATTTCCACATGTGAGTTAGTGAAGTCAGTTCCTAATTTATATAGTCATCTAAGTTATTTATTGATTTAACGTGAAAAGGTCCAGCCGCCCAGTAGACTATTCCCCTGCTGCCCTCGGGAACATGTCCTCTCAGTGAAATGTATTACCACGGCAAATTGTGTCTAGACACCCACCGCTGCCCCAGAGTTACCCAGAAACCAGAGGGCTCCTTCCGTCCCAATAGCAAAAGCCAGCTGTCTCACTTCCGAAGGTCCCTGTTGAGAAGGGGCTGTGCTGGGCTCTGAGGGTCCGGTGATTACCTGCTGCATGTGATGGTCTAGCTCCGTGTGTGTGTGTGTGTGTGTGTGTGTGTGTGTGTGTGTGTGTGTGTGTTTGTGTGTGTGAGAGGGAGGGTCCAGAAAGGGCCTTACCTCAGTTCTTCTCCCCCATCCCCTGAAGGATCTCTCTTGCCTCCCGTCTCATCCTTGGACTGCCTCGTTGTTTTCATGGGCTGTGTCTTGTGTGACTGTAAATTGTACCATTCCAGCCATTGGAGCCTTGCTTGGGCTGATCAAGGGCGTGGGAGGGGGGCTACTTCTCAAAGTGTGACCAAACCAAAATGTTTTATGGATTCCCATGAATTTGGGGGGTTCTGGTTTTTTGGGCACAGATATAGGGGTCATGTCCCTAAGCCCACCCTTCTCCCTGCCCTGCCCAGAAACACTTGACTTGGTGCGACTGTGTGTTGTCTACCCTTCTATGGTATGCGTGATGAGGGTCCCTTCCTTTCTGTGTCTAGTACTGTATCTCAGCAGTCATCCTGCCAGTAATCTCCAACCAGAGTGTCCCACCGTCCCATCCCTGAGGTGGGGTAACCAGATGCTAGGGAACGAGATGAAGAGTCACTCTCACTGGCCTCCTCACCTCCCCTAGCTGGGGTCCGGGGGAATCCGAGGATGATAGATGTGAAACCAGAAGGGACTCAGAAGTGACAGTGACTTGCCCTAGGTCACCCATCTCAGAGGCAGAAGCAGGATTTGAACCCAGAGCCGCTGTTCTTTACGCTGGGCCATAGAATTGAAGATGGGAGGGCCTGGTTTGAATTTCACGAGGAGACGGGGATAACCGCTCTTCCACCTGCCATTTCCAAACCAAGGCCCTTCTCTCTCTTTGGCCCAAGCTAAGTAGATTCTCTCCTGCCCAGTTGCTCCTATCTAGACACCAACAATCCTTTGAACCGGCGTGCGCCCTGCCAGGGCCAGGTGAAGTTGGCAGGCTATATTTTTACCTCTTCCTATTCATGAATTACTCCCTCCCATGGGAGGAATTCACCTGGCCCCACCCACCGACTCCACTCTGCCCCCAAGACAGTCTCAGCCTCTGGCTTCCTTGTCCCCAGCCGCCATCTTGAATTCCCTCCCCTCCTCCTCCATGGATGCCATGTCCACCCATGTCCTGACGCACTGTGATTGACTGAATGTTGTGAGGTGCCGTGTTTGTCTGCTTTGCTGTTTTCGGTGTGTCCACCTTCAGGGACTGCATGCCCACTTGGTTAGGACAGGGAGGAAGTCCTTACATTTGTCGCATCGGCAGTGACCATGCTTGCGGCACAGTGATATTTGTGGAGAAAACAGGAAAACATCATTAAAATTTTTTGCTGTTGGCTCAAAAAAAAAAAA

>UoN.Pci.T.9972_UoN.Pci.T.9972.1

GGTTGCTCAATTGCCATTTTTCTGCTCACTCAAAACAGCTTTACAAAGTTTTCCTGCAACTTACCTCTCAAGGGAACTTGCTCACTATTATTGTGGGACAGCTACCAGTCTTGGCTGTCTTGTTGGTAAATTCCTTGAGGGCAGGTACCTTGCCTTCTCTGAACTTTGTATCTTCCCTCACAAGGCGCTCTGAATGTTTGTTGAATGAAGTGAGGAGAGACTAAGTGCAAAGGACTGGGCTAAGAACAGGGTCCCTTCCCTCAAGGAGTTCACATTCATTCCTACAAGGAAGAGTCAATATAAGAAATATGCACATCTAAGACATATACAATGTAAAATGAAATTAATTTCAGAACAGAGGCATTAGCTGGGGGGAAGAAAGTTCAAGGGACAGGACAAGGCCTCTCGAAGAGGGTGGGATCCGAGGCGAGTCTCCAAGGCAGCTGGTGAGGTGAGGAGGGGCAGCATTCTCCAGGAACCAAGAGGAGGCTTAGTGCAGGGGTGGGGAACCTTCGGCCTTGAGGCCACACGTGGCCCTTTGAATCCAAACTTCACAGAACAACTCCATTTATTAAGGGGATTTGTTCTGTAAAATTTGGACTCAGTCAAAAGGCTGCACCCAGCACCTAGAAGGCCACATGGGCCTCAAGGCTGCAGGTTTCCCATCCCTGCCCACTAGAAGTATCTTTGACAACACAAGGTCAGTGTCAGAAACTTACGTGAAGGTTATCATTTTTTCTCTCAATTTAGCAAATAAATGTTTACAGGCTAAGGGCTCCAGTCGCTGTGTCCCTGGGTCCTCAGGCCGTGTTCTCAATGAGAACAGCAATACCTTGGCCACCACCAATACAAGCTGATCCCATGGCATACTTTCCACCCCGACGTCTCAATTCATGAACCAGATGAGCGGTGATTCGTGTTCCAGAGGCTCCCAGAGGGTGGCCCAGGGCAATGGCTCCTCCATTCACATTGGTTTTACTTGGGTCAAGATGCAAAGACTCTTCCACTGCTAGATACTGTGGAGCAAAAGCTTCATTCACTTCGACCAAATCCATGTCTTTCAGGCTCAGCCCTGACTTTTTTAGTGCTCCATTAATGGCAGAAACAGGACCAATGCCCATGATACTGGGATCACATCCAGATGCAAAGTAGCTCACAATTCTGGCGAGTGGGGACAGGTTATGCTTTTTAACAGCATCTTCACTCGCTAAGATCACTGCTGCAGCTCCGTCACACACCCCCGAAGCATTTCCAGCAGTGACGGTTCCCTCTTTCTTGAAGACTGGAGAAAGTTTGGCCAGCTGTTCTAGTGTTGTTTGAGGTCGGGGATGTTCATCCTTTTGCATCATCTCTTTTCCTTTCTTTGCTTTCACTTCAATTGGTACCATTTCATCATTAAAGTAGCCAGCATCATTAGCAGCTTTCCACCTCTGTTGCGACTGCAAAGCATATTTATCACAGTTTTCTCTGCTTATTTTATGCTTTACAGCAAGATTCTCAGCAGTAATTGCCATGGGGAGTTTAACATGTAGATCAGTCAGCGCTGTCCACAAACTGTCTTCCAGCTTGATGTCTACTCCCAGCCTGGTACCGAAGCGCACGTTTCTGACACAGTAGGGAGCCTGGCTCATACTTTCTGTTCCTCCACACAGTACCACTTCAGCATCTTTGACACAAATGTCCTGACATCCGCTGACAATAGACTGGAAGCCAGAGCCACACAGTCTATTCACAGTGAGAGCTGGGGTGGCCACAGGGACCCCTACACGCAAACCAACATGCCTCGCCAGGTATGCAGCGTCTGAAGAACTCTGCAGGACATTGCCCACGATCACGCTGTCAATAATTTCTGGAGGTACTTTGCTAGAAGATAGGCTAGCCCTGGCAGCCAATTCGGCCAGGTCAGTGGCTGTGAAATCCTTCAGTAAACCTCCAAATGCCCCAAAGGGTGTTCGCTTGGCAGCAACTATGTACACACCAAGTGCATGGCCTCTGTAGGGAGAGGCGTCAGAGCTCATCGCTCAAGGCTCAGGCTGTAGCACACAAGCCGCCCGATCCGGGAAGGGGAGAAACGAACGATCAAGGTCAGCGGAGCGTGGGGGCTGCAGCGACGCCGGCCCCGGGGCCCCTCAAAGCGGCCGAAGCAGAGCGGGCAGAGCGCGCACAGCGGGAGGGCTTCCAAGTGCTCGGAGCCGGCCGGGGCGCAGGGGCTTCCTCGCCTTACCTCGGAGCAGGGACATGGCTGGGCAGGAAGGCTGCTTCTTCCGCGTCCGGAGGACTCGCT

>UoN.Pci.T.9972_UoN.Pci.T.9972.2

GGTTGCTCAATTGCCATTTTTCTGCTCACTCAAAACAGCTTTACAAAGTTTTCCTGCAACTTACCTCTCAAGGGAACTTGCTCACTATTATTGTGGGACAGCTACCAGTCTTGGCTGTCTTGTTGGTAAATTCCTTGAGGGCAGGTACCTTGCCTTCTCTGAACTTTGTATCTTCCCTCACAAGGCGCTCTGAATGTTTGTTGAATGAAGTGAGGAGAGACTAAGTGCAAAGGACTGGGCTAAGAACAGGGTCCCTTCCCTCAAGGAGTTCACATTCATTCCTACAAGGAAGAGTCAATATAAGAAATATGCACATCTAAGACATATACAATGTAAAATGAAATTAATTTCAGAACAGAGGCATTAGCTGGGGGGAAGAAAGTTCAAGGGACAGGACAAGGCCTCTCGAAGAGGGTGGGATCCGAGGCGAGTCTCCAAGGCAGCTGGTGAGGTGAGGAGGGGCAGCATTCTCCAGGAACCAAGAGGAGGCTTAGTGCAGGGGTGGGGAACCTTCGGCCTTGAGGCCACACGTGGCCCTTTGAATCCAAACTTCACAGAACAACTCCATTTATTAAGGGGATTTGTTCTGTAAAATTTGGACTCAGTCAAAAGGCTGCACCCAGCACCTAGAAGGCCACATGGGCCTCAAGGCTGCAGGTTTCCCATCCCTGCCCACTAGAAGTATCTTTGACAACACAAGGTCAGTGTCAGAAACTTACGTGAAGGTTATCATTTTTTCTCTCAATTTAGCAAATAAATGTTTACAGGCTAAGGGCTCCAGTCGCTGTGTCCCTGGGTCCTCAGGCCGTGTTCTCAATGAGAACAGCAATACCTTGGCCACCACCAATACAAGCTGATCCCATGGCATACTTTCCACCCCGACGTCTCAATTCATGAACCAGATGAGCGGTGATTCGTGTTCCAGAGGCTCCCAGAGGGTGGCCCAGGGCAATGGCTCCTCCATTCACATTGGTTTTACTTGGGTCAAGATGCAAAGACTCTTCCACTGCTAGATACTGTGGAGCAAAAGCTTCATTCACTTCGACCAAATCCATGTCTTTCAGGCTCAGCCCTGACTTTTTTAGTGCTCCATTAATGGCAGAAACAGGACCAATGCCCATGATACTGGGATCACATCCAGATGCAAAGTAGCTCACAATTCTGGCGAGTGGGGACAGGTTATGCTTTTTAACAGCATCTTCACTCGCTAAGATCACTGCTGCAGCTCCGTCACACACCCCCGAAGCATTTCCAGCAGTGACGGTTCCCTCTTTCTTGAAGACTGGAGAAAGTTTGGCCAGCTGTTCTAGTGTTGTTTGAGGTCGGGGATGTTCATCCTTTTGCATCATCTCTTTTCCTTTCTTTGCTTTCACTTCAATTGGTACCATTTCATCATTAAAGTAGCCAGCATCATTAGCAGCTTTCCACCTCTGTTGCGACTGCAAAGCATATTTATCACAGTTTTCTCTGCTTATTTTATGCTTTACAGCAAGATTCTCAGCAGTAATTGCCATGGGGAGTTTAACATGTAGATCAGTCAGCGCTGTCCACAAACTGTCTTCCAGCTTGATGTCTACTCCCAGCCTGGTACCGAAGCGCACGTTTCTGACACAGTAGGGAGCCTGGCTCATACTTTCTGTTCCTCCACACAGTACCACTTCAGCATCTTTGACACAAATGTCCTGACATCCGCTGACAATAGACTGGAAGCCAGAGCCACACAGTCTATTCACAGTGAGAGCTGGGGTGGCCACAGGGACCCCTACACGCAAACCAACATGCCTCGCCAGGTATGCAGCGTCTGAAGAACTCTGCAGGACATTGCCCACGATCACGCTGTCAATAATTTCTGGAGGTACTTTGCTAGAAGATAGGCTAGCCCTGGCAGCCAATTCGGCCAGGTCAGTGGCTGTGAAATCCTTCAGTAAACCTCCAAATGCCCCAAAGGGTGTTCGCTTGGCAGCAACTATGTACACACCAAGTGCATGGCCTCTGTAGGGAGAGGCGTCAGAGCTCATCGCTCAAGGCTCAGGCTGTAGCACACAAGCCGCCCGATCCGGGAAGGGGAGAAACGAACGATCAAGGTCAGCGGAGCGTGGGGGCTGCAGCGACGCCGGCCCCGGGGCCCCTCAAAGCGGCCGAAGCAGAGCGGGCAGAGCGCGCACAGCGGGAGGGCTTCCAAGTGCTCGGAGCCGGCCGGGGCGCAGGGGCTTCCTCGCCTTACCTCGGAGCAGGGACATGGCTGGGCAGGAAGGCTGCTTCTTCCGCGTCCGGAGGACTCGCT

>UoN.Pci.T.9972_UoN.Pci.T.9972.3

GTTGCTCAATTGCCATTTTTCTGCTCACTCAAAACAGCTTTACAAAGTTTTCCTGCAACTTACCTCTCAAGGGAACTTGCTCACTATTATTGTGGGACAGCTACCAGTCTTGGCTGTCTTGTTGGTAAATTCCTTGAGGGCAGGTACCTTGCCTTCTCTGAACTTTGTATCTTCCCTCACAAGGCGCTCTGAATGTTTGTTGAATGAAGTGAGGAGAGACTAAGTGCAAAGGACTGGGCTAAGAACAGGGTCCCTTCCCTCAAGGAGTTCACATTCATTCCTACAAGGAAGAGTCAATATAAGAAATATGCACATCTAAGACATATACAATGTAAAATGAAATTAATTTCAGAACAGAGGCATTAGCTGGGGGGAAGAAAGTTCAAGGGACAGGACAAGGCCTCTCGAAGAGGGTGGGATCCGAGGCGAGTCTCCAAGGCAGCTGGTGAGGTGAGGAGGGGCAGCATTCTCCAGGAACCAAGAGGAGGCTTAGTGCAGGGGTGGGGAACCTTCGGCCTTGAGGCCACACGTGGCCCTTTGAATCCAAACTTCACAGAACAACTCCATTTATTAAGGGGATTTGTTCTGTAAAATTTGGACTCAGTCAAAAGGCTGCACCCAGCACCTAGAAGGCCACATGGGCCTCAAGGCTGCAGGTTTCCCATCCCTGCCCACTAGAAGTATCTTTGACAACACAAGGTCAGTGTCAGAAACTTACGTGAAGGTTATCATTTTTTCTCTCAATTTAGCAAATAAATGTTTACAGGCTAAGGGCTCCAGTCGCTGTGTCCCTGGGTCCTCAGGCCGTGTTCTCAATGAGAACAGCAATACCTTGGCCACCACCAATACAAGCTGATCCCATGGCATACTTTCCACCCCGACGTCTCAATTCATGAACCAGATGAGCGGTGATTCGTGTTCCAGAGGCTCCCAGAGGGTGGCCCAGGGCAATGGCTCCTCCATTCACATTGGTTTTACTTGGGTCAAGATGCAAAGACTCTTCCACTGCTAGATACTGTGGAGCAAAAGCTTCATTCACTTCGACCAAATCCATGTCTTTCAGGCTCAGCCCTGACTTTTTTAGTGCTCCATTAATGGCAGAAACAGGACCAATGCCCATGATACTGGGATCACATCCAGATGCAAAGTAGCTCACAATTCTGGCGAGTGGGGACAGGTTATGCTTTTTAACAGCATCTTCACTCGCTAAGATCACTGCTGCAGCTCCGTCACACACCCCCGAAGCATTTCCAGCAGTGACGGTTCCCTCTTTCTTGAAGACTGGAGAAAGTTTGGCCAGCTGTTCTAGTGTTGTTTGAGGTCGGGGATGTTCATCCTTTTGCATCATCTCTTTTCCTTTCTTTGCTTTCACTTCAATTGGTACCATTTCATCATTAAAGTAGCCAGCATCATTAGCAGCTTTCCACCTCTGTTGCGACTGCAAAGCATATTTATCACAGTTTTCTCTGCTTATTTTATGCTTTACAGCAAGATTCTCAGCAGTAATTGCCATGGGGAGTTTAACATGTAGATCAGTCAGCGCTGTCCACAAACTGTCTTCCAGCTTGATGTCTACTCCCAGCCTGGTACCGAAGCGCACGTTTCTGACACAGTAGGGAGCCTGGCTCATACTTTCTGTTCCTCCACACAGTACCACTTCAGCATCTTTGACACAAATGTCCTGACATCCGCTGACAATAGACTGGAAGCCAGAGCCACACAGTCTATTCACAGTGAGAGCTGGGGTGGCCACAGGGACCCCTACACGCAAACCAACATGCCTCGCCAGGTATGCAGCGTCTGAAGAACTCTGCAGGACATTGCCCACGATCACGCTGTCAATAATTTCTGGAGGTACTTTGCTAGAAGATAGGCTAGCCCTGGCAGCCAATTCGGCCAGGTCAGTGGCTGTGAAATCCTTCAGTAAACCTCCAAATGCCCCAAAGGGTGTTCGCTTGGCAGCAACTATGTACACACCAAGTGCATGGCCTCTGTAGGGAGAGGCGTCAGAGCTCATCGCTCAAGGCTCAGGCTGTAGCACACAAGCCGCCCGATCCGGGAAGGGGAGAAACGAACGATCAAGGTCAGCGGAGCGTGGGGGCTGCAGCGACGCCGGCCCCGGGGCCCCTCAAAGCGGCCGAAGCAGAGCGGGCAGAGCGCGCACAGCGGGAGGGCTTCCAAGTGCTCGGAGCCGGCCGGGGCGCAGGGGCTTCCTCGCCTTACCTCGGAGCAGGGACATGGCTGGGCAGGAAGGCTGCTTCTTCCGCGTCCGGAGGACTCGCT

>UoN.Pci.T.9972_UoN.Pci.T.9972.4

GGGATTTGTTCTGTAAAATTTGGACTCAGTCAAAAGGCTGCACCCAGCACCTAGAAGGCCACATGGGCCTCAAGGCTGCAGGTTTCCCATCCCTGCCCACTAGAAGTATCTTTGACAACACAAGGTCAGTGTCAGAAACTTACGTGAAGGTTATCATTTTTTCTCTCAATTTAGCAAATAAATGTTTACAGGCTAAGGGCTCCAGTCGCTGTGTCCCTGGGTCCTCAGGCCGTGTTCTCAATGAGAACAGCAATACCTTGGCCACCACCAATACAAGCTGATCCCATGGCATACTTTCCACCCCGACGTCTCAATTCATGAACCAGATGAGCGGTGATTCGTGTTCCAGAGGCTCCCAGAGGGTGGCCCAGGGCAATGGCTCCTCCATTCACATTGGTTTTACTTGGGTCAAGATGCAAAGACTCTTCCACTGCTAGATACTGTGGAGCAAAAGCTTCATTCACTTCGACCAAATCCATGTCTTTCAGGCTCAGCCCTGACTTTTTTAGTGCTCCATTAATGGCAGAAACAGGACCAATGCCCATGATACTGGGATCACATCCAGATGCAAAGTAGCTCACAATTCTGGCGAGTGGGGACAGGTTATGCTTTTTAACAGCATCTTCACTCGCTAAGATCACTGCTGCAGCTCCGTCACACACCCCCGAAGCATTTCCAGCAGTGACGGTTCCCTCTTTCTTGAAGACTGGAGAAAGTTTGGCCAGCTGTTCTAGTGTTGTTTGAGGTCGGGGATGTTCATCCTTTTGCATCATCTCTTTTCCTTTCTTTGCTTTCACTTCAATTGGTACCATTTCATCATTAAAGTAGCCAGCATCATTAGCAGCTTTCCACCTCTGTTGCGACTGCAAAGCATATTTATCACAGTTTTCTCTGCTTATTTTATGCTTTACAGCAAGATTCTCAGCAGTAATTGCCATGGGGAGTTTAACATGTAGATCAGTCAGCGCTGTCCACAAACTGTCTTCCAGCTTGATGTCTACTCCCAGCCTGGTACCGAAGCGCACGTTTCTGACACAGTAGGGAGCCTGGCTCATACTTTCTGTTCCTCCACACAGTACCACTTCAGCATCTTTGACACAAATGTCCTGACATCCGCTGACAATAGACTGGAAGCCAGAGCCACACAGTCTATTCACAGTGAGAGCTGGGGTGGCCACAGGGACCCCTACACGCAAACCAACATGCCTCGCCAGGTATGCAGCGTCTGAAGAACTCTGCAGGACATTGCCCACGATCACGCTGTCAATAATTTCTGGAGGTACTTTGCTAGAAGATAGGCTAGCCCTGGCAGCCAATTCGGCCAGGTCAGTGGCTGTGAAATCCTTCAGTAAACCTCCAAATGCCCCAAAGGGTGTTCGCTTGGCAGCAACTATGTACACACCAAGTGCATGGCCTCTGTAGGGAGAGGCGTCAGAGCTCATCGCTCAAGGCTCAGGCTGTAGCACACAAGCCGCCCGATCCGGGAAGGGGAGAAACGAACGATCAAGGTCAGCGGAGCGTGGGGGCTGCAGCGACGCCGGCCCCGGGGCCCCTCAAAGCGGCCGAAGCAGAGCGGGCAGAGCGCGCACAGCGGGAGGGCTTCCAAGTGCTCGGAGCCGGCCGGGGCGCAGGGGCTTCCTCGCCTTACCTCGGAGCAGGGACATGGCTGGGCAGGAAGGCTGCTTCTTCCGCGTCCGGAGGAC

>UoN.Pci.T.11154_UoN.Pci.T.11154.1

CGGAGCTCCCCCCCATTACGGGGTACCCCCCAGGAGGAGGGGGAGCTCCGCTCTATTAGGGGGTGCCCTCCAGGAGGAAGGGGAGCCCCGCTATATTACGGGATGGCCCCAAGAGAACGGGGAGCTCGGCTCTATTATGAGGTGCCCCCCAAGAGGAGCCAGAGCTCTCCTCTCTGTCATGGAGCATTCCCAGGGTGAGGGCAAGCTTCCCTCTATTACGGGGTGCCCCCAGGAGAATGGGGAGCTCCCCTCTCTGTCAAGGGGTGCTCCCCGGGGGGGTTCTACAAGGAATAACTCTCCAGACATACTACAAGTTGTGTACAGTTTAGAGGGAAGGGGTGGGGGTGGAGATCGCCCCCCAGAAGGACCTGCATCCCTAGCGGATGGACAAGGCAGGACTCCATACACCCGCCAACAATCGGGGGTCCTCCCCACCACCCAAGGGTGGCCCCTTCCTTGCCCCCTCCCCAGGGTCCCCGGGGTGGGGGCAGAGAAGGGGCCGGTCCCCACTGCAATGCGTCTGGAGAGCGGCAGCTGGAGACGCCCCCTGGAGGCCACTGTAGGAAGTCCCGGCCGAGCTCCAGGCAGGACCCTCACTGGGGGGCAACCCCCCCCCAGGCTGTCAGAGCCCGAGCATCCTCGTCCTCCCCCCCCCCCGCAGGGGCCTCCTCCCAAATCCAAGCCCTCCAGCCATGGCCACCCTGGTGATGTCCCGGGAGGAGAAGCTGAGTCAGGATGAGATCGTGCTGGGTACCAAGGCAGTCATCCAGGGGCTGGAGACCCTGCGGGGCGAGCACCGGGCCCTGCTGGCCTCCCTGCTGGAGAATGTGGCCGGGCGGGAGGCAGGAGAGGCTGAGCCCGCAACCCAGGAGCGCTCGGGGCTCCTGCGCCGATCCCTGGAAGCCATCGAGCTGGGACTTGGGGAAGCTCAGGTACCGGGGGGTCTGGGAGCAGGAGCATTTGAGGTCTAGGGGAGGCAAGCGTGGGAGAGGGGACCTGGAGTCCTGTCCTCCCACCCCGGCCGCCCCCTTCAGCCTTCTCTCCTGGCCGAGGGGCCATAGGTGATCCTGGCACTGTCAGGCCACCTTGGGGCCGTAGAGTCAGAGAAACAGAAGCTCCGGGCCCAGGTGCGCCGCTTGGTGCAGGAGAACCAGTGGCTCCGGGAGGAGCTGGCCGGGACCCAGCAGAAGCTGCAGCGCAGCGAGCAGGCCGTGGTCCAGCTGGAGGAGGAGAAGCAGCACCTGCTGTTTATGAGCCAGATCCGGAAGCTGGACGAGGACCCTTCTCCCAACGAGGAGAAGGGGGATGTTTCCAAGGATTCTGTGGATGACTTGTTCCCTAACGAGGAGGAACAGAACCCAGGTAGGGAGGTGGGGAGAAGGGGGTCTCTTAACTGTGGGCTCTGTGACTGGTCGGGAGGAAGGTGACCATCTATATGCCTCTCAGAAGGGGGGTGATTGCATATCCTGAGGACCCAGAAGTATGCAACAGATGAAGGAAGCTCCCAGGACTCCCCAGCATGCAGTTGCACTGCCCCAGCCGGTAGACAGCCCTTATTTCTGGGCCCTGGGGCCCCCTCTGACCTCCTGCCTTCCTCCAGCTCAGGGCCCTGGGGGTGGAGATGCTGCTGCCCAGCATGGAGGCTACGAGATTCCCGCCAGGCTAAGGACCTTGCACAATCTGGTGATTCAGTACGCCTCCCAGGGGCGCTATGAGGTAGCTGTGCCCCTCTGTAAGCAAGCCCTGGAGGACCTGGAAAAGACCTCAGGACATGACCACCCCGATGTGGCCACCATGCTGAACATCTTAGCTCTCGTGTATCGGTAAGGGGACGCCTGGAGCTCAGTGCTGAGGGGCACAGGGTGCGGACCTGCAGGCAGGAAGACACACTTAGGAGCTGAGTGACCCTAGGCAAGTCATTTCTCCCTGTGTGCCTCAGTTTCCTCATCTGTGAAACGAGCTAGAGAAGGAAATGGCAAACCCCTCCGGTATCTTTGCCAAAAAAATCCTAAGTGGGGTCACAAAGGGTCAGGCAAGACTGGACAATGATTGAATGAGAAGCAGCCTTGGATTCTGACCCTACTCAGGTGAAGGGAGGGGCCGGTGTGGGGTAATGAGCACCTCCTCCCCACCCCAAGAAAACCTTCACTGATGCCTGAAAGCCCCTCATTAGCCCATCACTCCTCTGAGTTGGCCCTCCTCCCCTTCCCCTGCCCCAAGCCCCTTATGGAAACCGTAAATCTCCTGAAGAAAGGGAGCATGTGTGTGCCCCGACGGCCCGGGCGAGGTCCTGGCTCTAGAACTGGCAGGAGTCTCATTTTACAGATGGGGAGCAGGCAGTCCAGGGAGGGCAGCGACTTAACCGAGGTCCCGCGAGTAGCCGTTGTGCTCTTCTCGGGGTCAGGTGGTCCTTCCCCTGCCCCCCATGCCATCTCTTTGTCCCCTCAGGGATCAGAATAAATACAAAGAAGCCGCCCACCTGCTCAACGATGCCCTGGCCATCCGGGAGAAGACCTTGGGCAAGGACCACCCAGCTGTGAGCAGGCCTGGCCTCCCTGGGCTGGGGGTGGGGGGTGAATGAGGGCCCTCTCTCCCCAGGGCCCTGCAGCTTTCTTCCTTCCTTCGCCCTGGTCCCAGGTGGCAGCTACCTTGAACAACCTGGCAGTCCTGTATGGCAAAAGGGGCAAATACAAGGAAGCAGAGCCCTTGTGTAAGCGAGCCCTGGAGATCCGAGAGAAGGTAGAGCCCGCTGGGCCTGTTTCCCCCCGGGGGCTGTAGGAGCAGCGGGGCGGCCGGGGTGGCGGCTGCTGGGTCAGAGCACAAGCGAAGGGAGCGTCCACCTGCTCCTAGGTGCTGGGCAGATTCCACCCGGACGTGGCCAAGCAGCTGAATAACCTGGCCCTGCTGTGCCAGAACCAGGGCAAGGCCGAGGAGGTGGAGTACTACTACCGGCGTGCTCTGGAGATCTACGAGGCCCGGCTGGGCCCAGACGACCCCAATGTGGCCAAGACCAAGAACAACCTGGCCTCTTGCTACCTGAAGCAGGGCAAGTACCAAGACGCAGAAACCCTGTATAAAGAGATCCTGACTCGTGCCCATGAGAAGGAGTTCGGCTCAGTCAATGGAGACAACAAGCCTATCTGGATGCACGCCGAAGAGCGGGAGGAGAGCAAGGATAAGCGTCGGGACACCACTCCCTATGGAGAATACGGTGGCTGGTACAAGGCTTGTAAAGTGGACAGCCCCACGGTGAACACCACGCTGCGGAGCCTCGGGGCTCTCTACCGCCGGCAAGGGAAGATGGAGGCGGCTCACACGCTGGAGGACTGTGCCAGCCGCAGTCGGAAGCAGGGCCTGGATGCCGTGAGCCAGACCAAGGTTGTGGAGCTGCTGAAAGATGGCAGCGGGCGAGGGGAGCGCCGGAGTGGCCGAGAGGGCGTTGGAGGCACGGGATCTCAGGCTGACCCACAGCCTGAGGATTCGGGGCCTGCAGCCGAGTGGGCCGGGGATGGCAGCGGTGCTCTCCGGAGAAGCGGCTCCTTTGGGAAGCTTCGCGATGCCTTGAGGCGCAGCAGTGAGATGCTGGTGAAGAAGCTGCAGGGGGGCGGACCCCAAGAGCCTCCCAACCCTCGGATGAAGCGAGCCAGTTCCCTTAACTTCCTGCACAAGAGCACAGAAGAGGCCATCCAGCCCGGCGACACCAACCTGTCCGACAGCCGCACGCTCAGCTCCAGCTCCCTTGACCTCTCTCGACGGAGCTCTCTCCTTGGCTAAGAGGGAGGGGTGGGAGGGCCTGGGCCCTCCTTGACTCCTCGTCATCTCCCGCAGGGACGGCAGGCGCAGGGGGCCACCTGATCCTCAGCCTCGCTCCCGCCCTGCGTAGGCCACCCTTCCCCCAAATGCTGTTTCCCACAGGTCCCCTCCCTCAGTGTTTTTTGTTTGATCTCAGGGTAACCTCTCCCCCTGTCATTTCAGGCTTAGCAGCTCAAAGCTTATTCCCCTCCCGGTCTAGCACTGTTAGTCACAAAACCGAGAAGCGTCACATTGGTGACAGTGCAGAGAGGAGCCAGGATTCCCCCCGCCCTTGTATTTATTTCCCTCTTCCCTTCCTCCCAGTGGGAGCCAGGAAGGTGAAAAGCGGGTGCCTGGCAGAGCTTCCCAGAGCAGGCTGGGCCACCTGGCCTTGGAGAAAGAGAATACCACCAGGCCTCCCTCTCCTCCCTTACCCAGGCCTCCGCTTCTGTATATAGAGAAATAAGTTATTGGCCACTCCCTTCCCCACTGCCTGTCCGTGGTACCTCCTCTAGCCCTTTCCCTGTCCAGGGATGCCACCCAGGCCTTAACCCCTTGCAGACAAGGTCTTGGACGCACACAAGTGCAGAAATATGCCCAAATGATGGAGCCTGCCTTCTCACCCCCTGAAACCCCATTAGTGTAGGATGGAAGTGCCATGCCCCCTACGGTACCTTCTCGTCTACAGTAGCTGACACCGTCTTCACTTTTCTCATCTTTGTTCCTTCCGGGGCATGGTGCCTGGGCCTAAAGCACTCCCTTTGAGCCACATTCCAGGGACTTCCGAAACCCTTCCCTGCCGCCCCCTCCCTCCCAAGGTACCGCCCGCTGAGCCATTCCTGGGGCTCCTACTGCTCCCAGTTCTCCCCTCCAGACTCTGAGGGAGGGGGCTGCACTCCTCTCCCGGAGCTGGGAGGGGGCAGGGTTGGGGTGGGCTGGGGAACCCATTTGCTTTGTTCAGATGTTGCTGT

>UoN.Pci.T.11154_UoN.Pci.T.11154.2

CGGAGCTCCCCCCCATTACGGGGTACCCCCCAGGAGGAGGGGGAGCTCCGCTCTATTAGGGGGTGCCCTCCAGGAGGAAGGGGAGCCCCGCTATATTACGGGATGGCCCCAAGAGAACGGGGAGCTCGGCTCTATTATGAGGTGCCCCCCAAGAGGAGCCAGAGCTCTCCTCTCTGTCATGGAGCATTCCCAGGGTGAGGGCAAGCTTCCCTCTATTACGGGGTGCCCCCAGGAGAATGGGGAGCTCCCCTCTCTGTCAAGGGGTGCTCCCCGGGGGGGTTCTACAAGGAATAACTCTCCAGACATACTACAAGTTGTGTACAGTTTAGAGGGAAGGGGTGGGGGTGGAGATCGCCCCCCAGAAGGACCTGCATCCCTAGCGGATGGACAAGGCAGGACTCCATACACCCGCCAACAATCGGGGGTCCTCCCCACCACCCAAGGGTGGCCCCTTCCTTGCCCCCTCCCCAGGGTCCCCGGGGTGGGGGCAGAGAAGGGGCCGGTCCCCACTGCAATGCGTCTGGAGAGCGGCAGCTGGAGACGCCCCCTGGAGGCCACTGTAGGAAGTCCCGGCCGAGCTCCAGGCAGGACCCTCACTGGGGGGCAACCCCCCCCCAGGCTGTCAGAGCCCGAGCATCCTCGTCCTCCCCCCCCCCCGCAGGGGCCTCCTCCCAAATCCAAGCCCTCCAGCCATGGCCACCCTGGTGATGTCCCGGGAGGAGAAGCTGAGTCAGGATGAGATCGTGCTGGGTACCAAGGCAGTCATCCAGGGGCTGGAGACCCTGCGGGGCGAGCACCGGGCCCTGCTGGCCTCCCTGCTGGAGAATGTGGCCGGGCGGGAGGCAGGAGAGGCTGAGCCCGCAACCCAGGAGCGCTCGGGGCTCCTGCGCCGATCCCTGGAAGCCATCGAGCTGGGACTTGGGGAAGCTCAGGTACCGGGGGGTCTGGGAGCAGGAGCATTTGAGGTCTAGGGGAGGCAAGCGTGGGAGAGGGGACCTGGAGTCCTGTCCTCCCACCCCGGCCGCCCCCTTCAGCCTTCTCTCCTGGCCGAGGGGCCATAGGTGATCCTGGCACTGTCAGGCCACCTTGGGGCCGTAGAGTCAGAGAAACAGAAGCTCCGGGCCCAGGTGCGCCGCTTGGTGCAGGAGAACCAGTGGCTCCGGGAGGAGCTGGCCGGGACCCAGCAGAAGCTGCAGCGCAGCGAGCAGGCCGTGGTCCAGCTGGAGGAGGAGAAGCAGCACCTGCTGTTTATGAGCCAGATCCGGAAGCTGGACGAGGACCCTTCTCCCAACGAGGAGAAGGGGGATGTTTCCAAGGATTCTGTGGATGACTTGTTCCCTAACGAGGAGGAACAGAACCCAGGTAGGGAGGTGGGGAGAAGGGGGTCTCTTAACTGTGGGCTCTGTGACTGGTCGGGAGGAAGGTGACCATCTATATGCCTCTCAGAAGGGGGGTGATTGCATATCCTGAGGACCCAGAAGTATGCAACAGATGAAGGAAGCTCCCAGGACTCCCCAGCATGCAGTTGCACTGCCCCAGCCGGTAGACAGCCCTTATTTCTGGGCCCTGGGGCCCCCTCTGACCTCCTGCCTTCCTCCAGCTCAGGGCCCTGGGGGTGGAGATGCTGCTGCCCAGCATGGAGGCTACGAGATTCCCGCCAGGCTAAGGACCTTGCACAATCTGGTGATTCAGTACGCCTCCCAGGGGCGCTATGAGGTAGCTGTGCCCCTCTGTAAGCAAGCCCTGGAGGACCTGGAAAAGACCTCAGGACATGACCACCCCGATGTGGCCACCATGCTGAACATCTTAGCTCTCGTGTATCGGTAAGGGGACGCCTGGAGCTCAGTGCTGAGGGGCACAGGGTGCGGACCTGCAGGCAGGAAGACACACTTAGGAGCTGAGTGACCCTAGGCAAGTCATTTCTCCCTGTGTGCCTCAGTTTCCTCATCTGTGAAACGAGCTAGAGAAGGAAATGGCAAACCCCTCCGGTATCTTTGCCAAAAAAATCCTAAGTGGGGTCACAAAGGGTCAGGCAAGACTGGACAATGATTGAATGAGAAGCAGCCTTGGATTCTGACCCTACTCAGGTGAAGGGAGGGGCCGGTGTGGGGTAATGAGCACCTCCTCCCCACCCCAAGAAAACCTTCACTGATGCCTGAAAGCCCCTCATTAGCCCATCACTCCTCTGAGTTGGCCCTCCTCCCCTTCCCCTGCCCCAAGCCCCTTATGGAAACCGTAAATCTCCTGAAGAAAGGGAGCATGTGTGTGCCCCGACGGCCCGGGCGAGGTCCTGGCTCTAGAACTGGCAGGAGTCTCATTTTACAGATGGGGAGCAGGCAGTCCAGGGAGGGCAGCGACTTAACCGAGGTCCCGCGAGTAGCCGTTGTGCTCTTCTCGGGGTCAGGTGGTCCTTCCCCTGCCCCCCATGCCATCTCTTTGTCCCCTCAGGGATCAGAATAAATACAAAGAAGCCGCCCACCTGCTCAACGATGCCCTGGCCATCCGGGAGAAGACCTTGGGCAAGGACCACCCAGCTGTGAGCAGGCCTGGCCTCCCTGGGCTGGGGGTGGGGGGTGAATGAGGGCCCTCTCTCCCCAGGGCCCTGCAGCTTTCTTCCTTCCTTCGCCCTGGTCCCAGGTGGCAGCTACCTTGAACAACCTGGCAGTCCTGTATGGCAAAAGGGGCAAATACAAGGAAGCAGAGCCCTTGTGTAAGCGAGCCCTGGAGATCCGAGAGAAGGTAGAGCCCGCTGGGCCTGTTTCCCCCCGGGGGCTGTAGGAGCAGCGGGGCGGCCGGGGTGGCGGCTGCTGGGTCAGAGCACAAGCGAAGGGAGCGTCCACCTGCTCCTAGGTGCTGGGCAGATTCCACCCGGACGTGGCCAAGCAGCTGAATAACCTGGCCCTGCTGTGCCAGAACCAGGGCAAGGCCGAGGAGGTGGAGTACTACTACCGGCGTGCTCTGGAGATCTACGAGGCCCGGCTGGGCCCAGACGACCCCAATGTGGCCAAGACCAAGAACAACCTGGCCTCTTGCTACCTGAAGCAGGGCAAGTACCAAGACGCAGAAACCCTGTATAAAGAGATCCTGACTCGTGCCCATGAGAAGGAGTTCGGCTCAGTCAATGGAGACAACAAGCCTATCTGGATGCACGCCGAAGAGCGGGAGGAGAGCAAGGATAAGCGTCGGGACACCACTCCCTATGGAGAATACGGTGGCTGGTACAAGGCTTGTAAAGTGGACAGCCCCACGGTGAACACCACGCTGCGGAGCCTCGGGGCTCTCTACCGCCGGCAAGGGAAGATGGAGGCGGCTCACACGCTGGAGGACTGTGCCAGCCGCAGTCGGAAGCAGGGCCTGGATGCCGTGAGCCAGACCAAGGTTGTGGAGCTGCTGAAAGATGGCAGCGGGCGAGGGGAGCGCCGGAGTGGCCGAGAGGGCGTTGGAGGCACGGGATCTCAGGCTGACCCACAGCCTGAGGATTCGGGGCCTGCAGCCGAGTGGGCCGGGGATGGCAGCGGTGCTCTCCGGAGAAGCGGCTCCTTTGGGAAGCTTCGCGATGCCTTGAGGCGCAGCAGTGAGATGCTGGTGAAGAAGCTGCAGGGGGGCGGACCCCAAGAGCCTCCCAACCCTCGGATGAAGCGAGCCAGTTCCCTTAACTTCCTGCACAAGAGCACAGAAGAGGCCATCCAGCCCGGCGACACCAACCTGTCCGACAGCCGCACGCTCAGCTCCAGCTCCCTTGACCTCTCTCGACGGAGCTCTCTCCTTGGCTAAGAGGGAGGGGTGGGAGGGCCTGGGCCCTCCTTGACTCCTCGTCATCTCCCGCAGGGACGGCAGGCGCAGGGGGCCACCTGATCCTCAGCCTCGCTCCCGCCCTGCGTAGGCCACCCTTCCCCCAAATGCTGTTTCCCACAGGTCCCCTCCCTCAGTGTTTTTTGTTTGATCTCAGGGTAACCTCTCCCCCTGTCATTTCAGGCTTAGCAGCTCAAAGCTTATTCCCCTCCCGGTCTAGCACTGTTAGTCACAAAACCGAGAAGCGTCACATTGGTGACAGTGCAGAGAGGAGCCAGGATTCCCCCCGCCCTTGTATTTATTTCCCTCTTCCCTTCCTCCCAGTGGGAGCCAGGAAGGTGAAAAGCGGGTGCCTGGCAGAGCTTCCCAGAGCAGGCTGGGCCACCTGGCCTTGGAGAAAGAGAATACCACCAGGCCTCCCTCTCCTCCCTTACCCAGGCCTCCGCTTCTGTATATAGAGAAATAAGTTATTGGCCACTCCCTTCCCCACTGCCTGTCCGTGGTACCTCCTCTAGCCCTTTCCCTGTCCAGGGATGCCACCCAGGCCTTAACCCCTTGCAGACAAGGTCTTGGACGCACACAAGTGCAGAAATATGCCCAAATGATGGAGCCTGCCTTCTCACCCCCTGAAACCCCATTAGTGTAGGATGGAAGTGCCATGCCCCCTACGGTACCTTCTCGTCTACAGTAGCTGACACCGTCTTCACTTTTCTCATCTTTGTTCCTTCCGGGGCATGGTGCCTGGGCCTAAAGCACTCCCTTTGAGCCACATTCCAGGGACTTCCGAAACCCTTCCCTGCCGCCCCCTCCCTCCCAAGGTACCGCCCGCTGAGCCATTCCTGGGGCTCCTACTGCTCCCAGTTCTCCCCTCCAGACTCTGAGGGAGGGGGCTGCACTCCTCTCCCGGAGCTGGGAGGGGGCAGGGTTGGGGTGGGCTGGGGAACCCATTTGCTTTGTTCAGATGTTGCTGT

>UoN.Pci.T.11154_UoN.Pci.T.11154.3

CGGAGCTCCCCCCCATTACGGGGTACCCCCCAGGAGGAGGGGGAGCTCCGCTCTATTAGGGGGTGCCCTCCAGGAGGAAGGGGAGCCCCGCTATATTACGGGATGGCCCCAAGAGAACGGGGAGCTCGGCTCTATTATGAGGTGCCCCCCAAGAGGAGCCAGAGCTCTCCTCTCTGTCATGGAGCATTCCCAGGGTGAGGGCAAGCTTCCCTCTATTACGGGGTGCCCCCAGGAGAATGGGGAGCTCCCCTCTCTGTCAAGGGGTGCTCCCCGGGGGGGTTCTACAAGGAATAACTCTCCAGACATACTACAAGTTGTGTACAGTTTAGAGGGAAGGGGTGGGGGTGGAGATCGCCCCCCAGAAGGACCTGCATCCCTAGCGGATGGACAAGGCAGGACTCCATACACCCGCCAACAATCGGGGGTCCTCCCCACCACCCAAGGGTGGCCCCTTCCTTGCCCCCTCCCCAGGGTCCCCGGGGTGGGGGCAGAGAAGGGGCCGGTCCCCACTGCAATGCGTCTGGAGAGCGGCAGCTGGAGACGCCCCCTGGAGGCCACTGTAGGAAGTCCCGGCCGAGCTCCAGGCAGGACCCTCACTGGGGGGCAACCCCCCCCCAGGCTGTCAGAGCCCGAGCATCCTCGTCCTCCCCCCCCCCCGCAGGGGCCTCCTCCCAAATCCAAGCCCTCCAGCCATGGCCACCCTGGTGATGTCCCGGGAGGAGAAGCTGAGTCAGGATGAGATCGTGCTGGGTACCAAGGCAGTCATCCAGGGGCTGGAGACCCTGCGGGGCGAGCACCGGGCCCTGCTGGCCTCCCTGCTGGAGAATGTGGCCGGGCGGGAGGCAGGAGAGGCTGAGCCCGCAACCCAGGAGCGCTCGGGGCTCCTGCGCCGATCCCTGGAAGCCATCGAGCTGGGACTTGGGGAAGCTCAGGTACCGGGGGGTCTGGGAGCAGGAGCATTTGAGGTCTAGGGGAGGCAAGCGTGGGAGAGGGGACCTGGAGTCCTGTCCTCCCACCCCGGCCGCCCCCTTCAGCCTTCTCTCCTGGCCGAGGGGCCATAGGTGATCCTGGCACTGTCAGGCCACCTTGGGGCCGTAGAGTCAGAGAAACAGAAGCTCCGGGCCCAGGTGCGCCGCTTGGTGCAGGAGAACCAGTGGCTCCGGGAGGAGCTGGCCGGGACCCAGCAGAAGCTGCAGCGCAGCGAGCAGGCCGTGGTCCAGCTGGAGGAGGAGAAGCAGCACCTGCTGTTTATGAGCCAGATCCGGAAGCTGGACGAGGACCCTTCTCCCAACGAGGAGAAGGGGGATGTTTCCAAGGATTCTGTGGATGACTTGTTCCCTAACGAGGAGGAACAGAACCCAGGTAGGGAGGTGGGGAGAAGGGGGTCTCTTAACTGTGGGCTCTGTGACTGGTCGGGAGGAAGGTGACCATCTATATGCCTCTCAGAAGGGGGGTGATTGCATATCCTGAGGACCCAGAAGTATGCAACAGATGAAGGAAGCTCCCAGGACTCCCCAGCATGCAGTTGCACTGCCCCAGCCGGTAGACAGCCCTTATTTCTGGGCCCTGGGGCCCCCTCTGACCTCCTGCCTTCCTCCAGCTCAGGGCCCTGGGGGTGGAGATGCTGCTGCCCAGCATGGAGGCTACGAGATTCCCGCCAGGCTAAGGACCTTGCACAATCTGGTGATTCAGTACGCCTCCCAGGGGCGCTATGAGGTAGCTGTGCCCCTCTGTAAGCAAGCCCTGGAGGACCTGGAAAAGACCTCAGGACATGACCACCCCGATGTGGCCACCATGCTGAACATCTTAGCTCTCGTGTATCGGTAAGGGGACGCCTGGAGCTCAGTGCTGAGGGGCACAGGGTGCGGACCTGCAGGCAGGAAGACACACTTAGGAGCTGAGTGACCCTAGGCAAGTCATTTCTCCCTGTGTGCCTCAGTTTCCTCATCTGTGAAACGAGCTAGAGAAGGAAATGGCAAACCCCTCCGGTATCTTTGCCAAAAAAATCCTAAGTGGGGTCACAAAGGGTCAGGCAAGACTGGACAATGATTGAATGAGAAGCAGCCTTGGATTCTGACCCTACTCAGGTGAAGGGAGGGGCCGGTGTGGGGTAATGAGCACCTCCTCCCCACCCCAAGAAAACCTTCACTGATGCCTGAAAGCCCCTCATTAGCCCATCACTCCTCTGAGTTGGCCCTCCTCCCCTTCCCCTGCCCCAAGCCCCTTATGGAAACCGTAAATCTCCTGAAGAAAGGGAGCATGTGTGTGCCCCGACGGCCCGGGCGAGGTCCTGGCTCTAGAACTGGCAGGAGTCTCATTTTACAGATGGGGAGCAGGCAGTCCAGGGAGGGCAGCGACTTAACCGAGGTCCCGCGAGTAGCCGTTGTGCTCTTCTCGGGGTCAGGTGGTCCTTCCCCTGCCCCCCATGCCATCTCTTTGTCCCCTCAGGGATCAGAATAAATACAAAGAAGCCGCCCACCTGCTCAACGATGCCCTGGCCATCCGGGAGAAGACCTTGGGCAAGGACCACCCAGCTGTGAGCAGGCCTGGCCTCCCTGGGCTGGGGGTGGGGGGTGAATGAGGGCCCTCTCTCCCCAGGGCCCTGCAGCTTTCTTCCTTCCTTCGCCCTGGTCCCAGGTGGCAGCTACCTTGAACAACCTGGCAGTCCTGTATGGCAAAAGGGGCAAATACAAGGAAGCAGAGCCCTTGTGTAAGCGAGCCCTGGAGATCCGAGAGAAGGTAGAGCCCGCTGGGCCTGTTTCCCCCCGGGGGCTGTAGGAGCAGCGGGGCGGCCGGGGTGGCGGCTGCTGGGTCAGAGCACAAGCGAAGGGAGCGTCCACCTGCTCCTAGGTGCTGGGCAGATTCCACCCGGACGTGGCCAAGCAGCTGAATAACCTGGCCCTGCTGTGCCAGAACCAGGGCAAGGCCGAGGAGGTGGAGTACTACTACCGGCGTGCTCTGGAGATCTACGAGGCCCGGCTGGGCCCAGACGACCCCAATGTGGCCAAGACCAAGAACAACCTGGCCTCTTGCTACCTGAAGCAGGGCAAGTACCAAGACGCAGAAACCCTGTATAAAGAGATCCTGACTCGTGCCCATGAGAAGGAGTTCGGCTCAGTCAATGGAGACAACAAGCCTATCTGGATGCACGCCGAAGAGCGGGAGGAGAGCAAGGATAAGCGTCGGGACACCACTCCCTATGGAGAATACGGTGGCTGGTACAAGGCTTGTAAAGTGGACAGCCCCACGGTGAACACCACGCTGCGGAGCCTCGGGGCTCTCTACCGCCGGCAAGGGAAGATGGAGGCGGCTCACACGCTGGAGGACTGTGCCAGCCGCAGTCGGAAGCAGGGCCTGGATGCCGTGAGCCAGACCAAGGTTGTGGAGCTGCTGAAAGATGGCAGCGGGCGAGGGGAGCGCCGGAGTGGCCGAGAGGGCGTTGGAGGCACGGGATCTCAGGCTGACCCACAGCCTGAGGATTCGGGGCCTGCAGCCGAGTGGGCCGGGGATGGCAGCGGTGCTCTCCGGAGAAGCGGCTCCTTTGGGAAGCTTCGCGATGCCTTGAGGCGCAGCAGTGAGATGCTGGTGAAGAAGCTGCAGGGGGGCGGACCCCAAGAGCCTCCCAACCCTCGGATGAAGCGAGCCAGTTCCCTTAACTTCCTGCACAAGAGCACAGAAGAGGCCATCCAGCCCGGCGACACCAACCTGTCCGACAGCCGCACGCTCAGCTCCAGCTCCCTTGACCTCTCTCGACGGAGCTCTCTCCTTGGCTAAGAGGGAGGGGTGGGAGGGCCTGGGCCCTCCTTGACTCCTCGTCATCTCCCGCAGGGACGGCAGGCGCAGGGGGCCACCTGATCCTCAGCCTCGCTCCCGCCCTGCGTAGGCCACCCTTCCCCCAAATGCTGTTTCCCACAGGTCCCCTCCCTCAGTGTTTTTTGTTTGATCTCAGGGTAACCTCTCCCCCTGTCATTTCAGGCTTAGCAGCTCAAAGCTTATTCCCCTCCCGGTCTAGCACTGTTAGTCACAAAACCGAGAAGCGTCACATTGGTGACAGTGCAGAGAGGAGCCAGGATTCCCCCCGCCCTTGTATTTATTTCCCTCTTCCCTTCCTCCCAGTGGGAGCCAGGAAGGTGAAAAGCGGGTGCCTGGCAGAGCTTCCCAGAGCAGGCTGGGCCACCTGGCCTTGGAGAAAGAGAATACCACCAGGCCTCCCTCTCCTCCCTTACCCAGGCCTCCGCTTCTGTATATAGAGAAATAAGTTATTGGCCACTCCCTTCCCCACTGCCTGTCCGTGGTACCTCCTCTAGCCCTTTCCCTGTCCAGGGATGCCACCCAGGCCTTAACCCCTTGCAGACAAGGTCTTGGACGCACACAAGTGCAGAAATATGCCCAAATGATGGAGCCTGCCTTCTCACCCCCTGAAACCCCATTAGTGTAGGATGGAAGTGCCATGCCCCCTACGGTACCTTCTCGTCTACAGTAGCTGACACCGTCTTCACTTTTCTCATCTTTGTTCCTTCCGGGGCATGGTGCCTGGGCCTAAAGCACTCCCTTTGAGCCACATTCCAGGGACTTCCGAAACCCTTCCCTGCCGCCCCCTCCCTCCCAAGGTACCGCCCGCTGAGCCATTCCTGGGGCTCCTACTGCTCCCAGTTCTCCCCTCCAGACTCTGAGGGAGGGGGCTGCACTCCTCTCCCGGAGCTGGGAGGGGGCAGGGTTGGGGTGGGCTGGGGAACCCATTTGCTTTGTTCAGATGTTGCTGT

>UoN.Pci.T.11154_UoN.Pci.T.11154.4

CCCCCCCATTACGGGGTACCCCCCAGGAGGAGGGGGAGCTCCGCTCTATTAGGGGGTGCCCTCCAGGAGGAAGGGGAGCCCCGCTATATTACGGGATGGCCCCAAGAGAACGGGGAGCTCGGCTCTATTATGAGGTGCCCCCCAAGAGGAGCCAGAGCTCTCCTCTCTGTCATGGAGCATTCCCAGGGTGAGGGCAAGCTTCCCTCTATTACGGGGTGCCCCCAGGAGAATGGGGAGCTCCCCTCTCTGTCAAGGGGTGCTCCCCGGGGGGGTTCTACAAGGAATAACTCTCCAGACATACTACAAGTTGTGTACAGTTTAGAGGGAAGGGGTGGGGGTGGAGATCGCCCCCCAGAAGGACCTGCATCCCTAGCGGATGGACAAGGCAGGACTCCATACACCCGCCAACAATCGGGGGTCCTCCCCACCACCCAAGGGTGGCCCCTTCCTTGCCCCCTCCCCAGGGTCCCCGGGGTGGGGGCAGAGAAGGGGCCGGTCCCCACTGCAATGCGTCTGGAGAGCGGCAGCTGGAGACGCCCCCTGGAGGCCACTGTAGGAAGTCCCGGCCGAGCTCCAGGCAGGACCCTCACTGGGGGGCAACCCCCCCCCAGGCTGTCAGAGCCCGAGCATCCTCGTCCTCCCCCCCCCCCGCAGGGGCCTCCTCCCAAATCCAAGCCCTCCAGCCATGGCCACCCTGGTGATGTCCCGGGAGGAGAAGCTGAGTCAGGATGAGATCGTGCTGGGTACCAAGGCAGTCATCCAGGGGCTGGAGACCCTGCGGGGCGAGCACCGGGCCCTGCTGGCCTCCCTGCTGGAGAATGTGGCCGGGCGGGAGGCAGGAGAGGCTGAGCCCGCAACCCAGGAGCGCTCGGGGCTCCTGCGCCGATCCCTGGAAGCCATCGAGCTGGGACTTGGGGAAGCTCAGGTACCGGGGGGTCTGGGAGCAGGAGCATTTGAGGTCTAGGGGAGGCAAGCGTGGGAGAGGGGACCTGGAGTCCTGTCCTCCCACCCCGGCCGCCCCCTTCAGCCTTCTCTCCTGGCCGAGGGGCCATAGGTGATCCTGGCACTGTCAGGCCACCTTGGGGCCGTAGAGTCAGAGAAACAGAAGCTCCGGGCCCAGGTGCGCCGCTTGGTGCAGGAGAACCAGTGGCTCCGGGAGGAGCTGGCCGGGACCCAGCAGAAGCTGCAGCGCAGCGAGCAGGCCGTGGTCCAGCTGGAGGAGGAGAAGCAGCACCTGCTGTTTATGAGCCAGATCCGGAAGCTGGACGAGGACCCTTCTCCCAACGAGGAGAAGGGGGATGTTTCCAAGGATTCTGTGGATGACTTGTTCCCTAACGAGGAGGAACAGAACCCAGGTAGGGAGGTGGGGAGAAGGGGGTCTCTTAACTGTGGGCTCTGTGACTGGTCGGGAGGAAGGTGACCATCTATATGCCTCTCAGAAGGGGGGTGATTGCATATCCTGAGGACCCAGAAGTATGCAACAGATGAAGGAAGCTCCCAGGACTCCCCAGCATGCAGTTGCACTGCCCCAGCCGGTAGACAGCCCTTATTTCTGGGCCCTGGGGCCCCCTCTGACCTCCTGCCTTCCTCCAGCTCAGGGCCCTGGGGGTGGAGATGCTGCTGCCCAGCATGGAGGCTACGAGATTCCCGCCAGGCTAAGGACCTTGCACAATCTGGTGATTCAGTACGCCTCCCAGGGGCGCTATGAGGTAGCTGTGCCCCTCTGTAAGCAAGCCCTGGAGGACCTGGAAAAGACCTCAGGACATGACCACCCCGATGTGGCCACCATGCTGAACATCTTAGCTCTCGTGTATCGGTAAGGGGACGCCTGGAGCTCAGTGCTGAGGGGCACAGGGTGCGGACCTGCAGGCAGGAAGACACACTTAGGAGCTGAGTGACCCTAGGCAAGTCATTTCTCCCTGTGTGCCTCAGTTTCCTCATCTGTGAAACGAGCTAGAGAAGGAAATGGCAAACCCCTCCGGTATCTTTGCCAAAAAAATCCTAAGTGGGGTCACAAAGGGTCAGGCAAGACTGGACAATGATTGAATGAGAAGCAGCCTTGGATTCTGACCCTACTCAGGTGAAGGGAGGGGCCGGTGTGGGGTAATGAGCACCTCCTCCCCACCCCAAGAAAACCTTCACTGATGCCTGAAAGCCCCTCATTAGCCCATCACTCCTCTGAGTTGGCCCTCCTCCCCTTCCCCTGCCCCAAGCCCCTTATGGAAACCGTAAATCTCCTGAAGAAAGGGAGCATGTGTGTGCCCCGACGGCCCGGGCGAGGTCCTGGCTCTAGAACTGGCAGGAGTCTCATTTTACAGATGGGGAGCAGGCAGTCCAGGGAGGGCAGCGACTTAACCGAGGTCCCGCGAGTAGCCGTTGTGCTCTTCTCGGGGTCAGGTGGTCCTTCCCCTGCCCCCCATGCCATCTCTTTGTCCCCTCAGGGATCAGAATAAATACAAAGAAGCCGCCCACCTGCTCAACGATGCCCTGGCCATCCGGGAGAAGACCTTGGGCAAGGACCACCCAGCTGTGAGCAGGCCTGGCCTCCCTGGGCTGGGGGTGGGGGGTGAATGAGGGCCCTCTCTCCCCAGGGCCCTGCAGCTTTCTTCCTTCCTTCGCCCTGGTCCCAGGTGGCAGCTACCTTGAACAACCTGGCAGTCCTGTATGGCAAAAGGGGCAAATACAAGGAAGCAGAGCCCTTGTGTAAGCGAGCCCTGGAGATCCGAGAGAAGGTAGAGCCCGCTGGGCCTGTTTCCCCCCGGGGGCTGTAGGAGCAGCGGGGCGGCCGGGGTGGCGGCTGCTGGGTCAGAGCACAAGCGAAGGGAGCGTCCACCTGCTCCTAGGTGCTGGGCAGATTCCACCCGGACGTGGCCAAGCAGCTGAATAACCTGGCCCTGCTGTGCCAGAACCAGGGCAAGGCCGAGGAGGTGGAGTACTACTACCGGCGTGCTCTGGAGATCTACGAGGCCCGGCTGGGCCCAGACGACCCCAATGTGGCCAAGACCAAGAACAACCTGGCCTCTTGCTACCTGAAGCAGGGCAAGTACCAAGACGCAGAAACCCTGTATAAAGAGATCCTGACTCGTGCCCATGAGAAGGAGTTCGGCTCAGTCAATGGAGACAACAAGCCTATCTGGATGCACGCCGAAGAGCGGGAGGAGAGCAAGGATAAGCGTCGGGACACCACTCCCTATGGAGAATACGGTGGCTGGTACAAGGCTTGTAAAGTGGACAGCCCCACGGTGAACACCACGCTGCGGAGCCTCGGGGCTCTCTACCGCCGGCAAGGGAAGATGGAGGCGGCTCACACGCTGGAGGACTGTGCCAGCCGCAGTCGGAAGCAGGGCCTGGATGCCGTGAGCCAGACCAAGGTTGTGGAGCTGCTGAAAGATGGCAGCGGGCGAGGGGAGCGCCGGAGTGGCCGAGAGGGCGTTGGAGGCACGGGATCTCAGGCTGACCCACAGCCTGAGGATTCGGGGCCTGCAGCCGAGTGGGCCGGGGATGGCAGCGGTGCTCTCCGGAGAAGCGGCTCCTTTGGGAAGCTTCGCGATGCCTTGAGGCGCAGCAGTGAGATGCTGGTGAAGAAGCTGCAGGGGGGCGGACCCCAAGAGCCTCCCAACCCTCGGATGAAGCGAGCCAGTTCCCTTAACTTCCTGCACAAGAGCACAGAAGAGGCCATCCAGCCCGGCGACACCAACCTGTCCGACAGCCGCACGCTCAGCTCCAGCTCCCTTGACCTCTCTCGACGGAGCTCTCTCCTTGGCTAAGAGGGAGGGGTGGGAGGGCCTGGGCCCTCCTTGACTCCTCGTCATCTCCCGCAGGGACGGCAGGCGCAGGGGGCCACCTGATCCTCAGCCTCGCTCCCGCCCTGCGTAGGCCACCCTTCCCCCAAATGCTGTTTCCCACAGGTCCCCTCCCTCAGTGTTTTTTGTTTGATCTCAGGGTAACCTCTCCCCCTGTCATTTCAGGCTTAGCAGCTCAAAGCTTATTCCCCTCCCGGTCTAGCACTGTTAGTCACAAAACCGAGAAGCGTCACATTGGTGACAGTGCAGAGAGGAGCCAGGATTCCCCCCGCCCTTGTATTTATTTCCCTCTTCCCTTCCTCCCAGTGGGAGCCAGGAAGGTGAAAAGCGGGTGCCTGGCAGAGCTTCCCAGAGCAGGCTGGGCCACCTGGCCTTGGAGAAAGAGAATACCACCAGGCCTCCCTCTCCTCCCTTACCCAGGCCTCCGCTTCTGTATATAGAGAAATAAGTTATTGGCCACTCCCTTCCCCACTGCCTGTCCGTGGTACCTCCTCTAGCCCTTTCCCTGTCCAGGGATGCCACCCAGGCCTTAACCCCTTGCAGACAAGGTCTTGGACGCACACAAGTGCAGAAATATGCCCAAATGATGGAGCCTGCCTTCTCACCCCCTGAAACCCCATTAGTGTAGGATGGAAGTGCCATGCCCCCTACGGTACCTTCTCGTCTACAGTAGCTGACACCGTCTTCACTTTTCTCATCTTTGTTCCTTCCGGGGCATGGTGCCTGGGCCTAAAGCACTCCCTTTGAGCCACATTCCAGGGACTTCCGAAACCCTTCCCTGCCGCCCCCTCCCTCCCAAGGTACCGCCCGCTGAGCCATTCCTGGGGCTCCTACTGCTCCCAGTTCTCCCCTCCAGACTCTGAGGGAGGGGGCTGCACTCCTCTCCCGGAGCTGGGAGGGGGCAGGGTTGGGGTGGGCTGGGGAACCCATTTGCTTTGTTCAGATGTTGCTGT

>UoN.Pci.T.11154_UoN.Pci.T.11154.5

CGGCTCTATTATGAGGTGCCCCCCAAGAGGAGCCAGAGCTCTCCTCTCTGTCATGGAGCATTCCCAGGGTGAGGGCAAGCTTCCCTCTATTACGGGGTGCCCCCAGGAGAATGGGGAGCTCCCCTCTCTGTCAAGGGGTGCTCCCCGGGGGGGTTCTACAAGGAATAACTCTCCAGACATACTACAAGTTGTGTACAGTTTAGAGGGAAGGGGTGGGGGTGGAGATCGCCCCCCAGAAGGACCTGCATCCCTAGCGGATGGACAAGGCAGGACTCCATACACCCGCCAACAATCGGGGGTCCTCCCCACCACCCAAGGGTGGCCCCTTCCTTGCCCCCTCCCCAGGGTCCCCGGGGTGGGGGCAGAGAAGGGGCCGGTCCCCACTGCAATGCGTCTGGAGAGCGGCAGCTGGAGACGCCCCCTGGAGGCCACTGTAGGAAGTCCCGGCCGAGCTCCAGGCAGGACCCTCACTGGGGGGCAACCCCCCCCCAGGCTGTCAGAGCCCGAGCATCCTCGTCCTCCCCCCCCCCCGCAGGGGCCTCCTCCCAAATCCAAGCCCTCCAGCCATGGCCACCCTGGTGATGTCCCGGGAGGAGAAGCTGAGTCAGGATGAGATCGTGCTGGGTACCAAGGCAGTCATCCAGGGGCTGGAGACCCTGCGGGGCGAGCACCGGGCCCTGCTGGCCTCCCTGCTGGAGAATGTGGCCGGGCGGGAGGCAGGAGAGGCTGAGCCCGCAACCCAGGAGCGCTCGGGGCTCCTGCGCCGATCCCTGGAAGCCATCGAGCTGGGACTTGGGGAAGCTCAGGTACCGGGGGGTCTGGGAGCAGGAGCATTTGAGGTCTAGGGGAGGCAAGCGTGGGAGAGGGGACCTGGAGTCCTGTCCTCCCACCCCGGCCGCCCCCTTCAGCCTTCTCTCCTGGCCGAGGGGCCATAGGTGATCCTGGCACTGTCAGGCCACCTTGGGGCCGTAGAGTCAGAGAAACAGAAGCTCCGGGCCCAGGTGCGCCGCTTGGTGCAGGAGAACCAGTGGCTCCGGGAGGAGCTGGCCGGGACCCAGCAGAAGCTGCAGCGCAGCGAGCAGGCCGTGGTCCAGCTGGAGGAGGAGAAGCAGCACCTGCTGTTTATGAGCCAGATCCGGAAGCTGGACGAGGACCCTTCTCCCAACGAGGAGAAGGGGGATGTTTCCAAGGATTCTGTGGATGACTTGTTCCCTAACGAGGAGGAACAGAACCCAGGTAGGGAGGTGGGGAGAAGGGGGTCTCTTAACTGTGGGCTCTGTGACTGGTCGGGAGGAAGGTGACCATCTATATGCCTCTCAGAAGGGGGGTGATTGCATATCCTGAGGACCCAGAAGTATGCAACAGATGAAGGAAGCTCCCAGGACTCCCCAGCATGCAGTTGCACTGCCCCAGCCGGTAGACAGCCCTTATTTCTGGGCCCTGGGGCCCCCTCTGACCTCCTGCCTTCCTCCAGCTCAGGGCCCTGGGGGTGGAGATGCTGCTGCCCAGCATGGAGGCTACGAGATTCCCGCCAGGCTAAGGACCTTGCACAATCTGGTGATTCAGTACGCCTCCCAGGGGCGCTATGAGGTAGCTGTGCCCCTCTGTAAGCAAGCCCTGGAGGACCTGGAAAAGACCTCAGGACATGACCACCCCGATGTGGCCACCATGCTGAACATCTTAGCTCTCGTGTATCGGTAAGGGGACGCCTGGAGCTCAGTGCTGAGGGGCACAGGGTGCGGACCTGCAGGCAGGAAGACACACTTAGGAGCTGAGTGACCCTAGGCAAGTCATTTCTCCCTGTGTGCCTCAGTTTCCTCATCTGTGAAACGAGCTAGAGAAGGAAATGGCAAACCCCTCCGGTATCTTTGCCAAAAAAATCCTAAGTGGGGTCACAAAGGGTCAGGCAAGACTGGACAATGATTGAATGAGAAGCAGCCTTGGATTCTGACCCTACTCAGGTGAAGGGAGGGGCCGGTGTGGGGTAATGAGCACCTCCTCCCCACCCCAAGAAAACCTTCACTGATGCCTGAAAGCCCCTCATTAGCCCATCACTCCTCTGAGTTGGCCCTCCTCCCCTTCCCCTGCCCCAAGCCCCTTATGGAAACCGTAAATCTCCTGAAGAAAGGGAGCATGTGTGTGCCCCGACGGCCCGGGCGAGGTCCTGGCTCTAGAACTGGCAGGAGTCTCATTTTACAGATGGGGAGCAGGCAGTCCAGGGAGGGCAGCGACTTAACCGAGGTCCCGCGAGTAGCCGTTGTGCTCTTCTCGGGGTCAGGTGGTCCTTCCCCTGCCCCCCATGCCATCTCTTTGTCCCCTCAGGGATCAGAATAAATACAAAGAAGCCGCCCACCTGCTCAACGATGCCCTGGCCATCCGGGAGAAGACCTTGGGCAAGGACCACCCAGCTGTGAGCAGGCCTGGCCTCCCTGGGCTGGGGGTGGGGGGTGAATGAGGGCCCTCTCTCCCCAGGGCCCTGCAGCTTTCTTCCTTCCTTCGCCCTGGTCCCAGGTGGCAGCTACCTTGAACAACCTGGCAGTCCTGTATGGCAAAAGGGGCAAATACAAGGAAGCAGAGCCCTTGTGTAAGCGAGCCCTGGAGATCCGAGAGAAGGTAGAGCCCGCTGGGCCTGTTTCCCCCCGGGGGCTGTAGGAGCAGCGGGGCGGCCGGGGTGGCGGCTGCTGGGTCAGAGCACAAGCGAAGGGAGCGTCCACCTGCTCCTAGGTGCTGGGCAGATTCCACCCGGACGTGGCCAAGCAGCTGAATAACCTGGCCCTGCTGTGCCAGAACCAGGGCAAGGCCGAGGAGGTGGAGTACTACTACCGGCGTGCTCTGGAGATCTACGAGGCCCGGCTGGGCCCAGACGACCCCAATGTGGCCAAGACCAAGAACAACCTGGCCTCTTGCTACCTGAAGCAGGGCAAGTACCAAGACGCAGAAACCCTGTATAAAGAGATCCTGACTCGTGCCCATGAGAAGGAGTTCGGCTCAGTCAATGGAGACAACAAGCCTATCTGGATGCACGCCGAAGAGCGGGAGGAGAGCAAGGATAAGCGTCGGGACACCACTCCCTATGGAGAATACGGTGGCTGGTACAAGGCTTGTAAAGTGGACAGCCCCACGGTGAACACCACGCTGCGGAGCCTCGGGGCTCTCTACCGCCGGCAAGGGAAGATGGAGGCGGCTCACACGCTGGAGGACTGTGCCAGCCGCAGTCGGAAGCAGGGCCTGGATGCCGTGAGCCAGACCAAGGTTGTGGAGCTGCTGAAAGATGGCAGCGGGCGAGGGGAGCGCCGGAGTGGCCGAGAGGGCGTTGGAGGCACGGGATCTCAGGCTGACCCACAGCCTGAGGATTCGGGGCCTGCAGCCGAGTGGGCCGGGGATGGCAGCGGTGCTCTCCGGAGAAGCGGCTCCTTTGGGAAGCTTCGCGATGCCTTGAGGCGCAGCAGTGAGATGCTGGTGAAGAAGCTGCAGGGGGGCGGACCCCAAGAGCCTCCCAACCCTCGGATGAAGCGAGCCAGTTCCCTTAACTTCCTGCACAAGAGCACAGAAGAGGCCATCCAGCCCGGCGACACCAACCTGTCCGACAGCCGCACGCTCAGCTCCAGCTCCCTTGACCTCTCTCGACGGAGCTCTCTCCTTGGCTAAGAGGGAGGGGTGGGAGGGCCTGGGCCCTCCTTGACTCCTCGTCATCTCCCGCAGGGACGGCAGGCGCAGGGGGCCACCTGATCCTCAGCCTCGCTCCCGCCCTGCGTAGGCCACCCTTCCCCCAAATGCTGTTTCCCACAGGTCCCCTCCCTCAGTGTTTTTTGTTTGATCTCAGGGTAACCTCTCCCCCTGTCATTTCAGGCTTAGCAGCTCAAAGCTTATTCCCCTCCCGGTCTAGCACTGTTAGTCACAAAACCGAGAAGCGTCACATTGGTGACAGTGCAGAGAGGAGCCAGGATTCCCCCCGCCCTTGTATTTATTTCCCTCTTCCCTTCCTCCCAGTGGGAGCCAGGAAGGTGAAAAGCGGGTGCCTGGCAGAGCTTCCCAGAGCAGGCTGGGCCACCTGGCCTTGGAGAAAGAGAATACCACCAGGCCTCCCTCTCCTCCCTTACCCAGGCCTCCGCTTCTGTATATAGAGAAATAAGTTATTGGCCACTCCCTTCCCCACTGCCTGTCCGTGGTACCTCCTCTAGCCCTTTCCCTGTCCAGGGATGCCACCCAGGCCTTAACCCCTTGCAGACAAGGTCTTGGACGCACACAAGTGCAGAAATATGCCCAAATGATGGAGCCTGCCTTCTCACCCCCTGAAACCCCATTAGTGTAGGATGGAAGTGCCATGCCCCCTACGGTACCTTCTCGTCTACAGTAGCTGACACCGTCTTCACTTTTCTCATCTTTGTTCCTTCCGGGGCATGGTGCCTGGGCCTAAAGCACTCCCTTTGAGCCACATTCCAGGGACTTCCGAAACCCTTCCCTGCCGCCCCCTCCCTCCCAAGGTACCGCCCGCTGAGCCATTCCTGGGGCTCCTACTGCTCCCAGTTCTCCCCTCCAGACTCTGAGGGAGGGGGCTGCACTCCTCTCCCGGAGCTGGGAGGGGGCAGGGTTGGGGTGGGCTGGGGAACCCATTTGCTTTGTTCAGATGTTGCTGT

>UoN.Pci.T.11154_UoN.Pci.T.11154.6

CCCAGAAGGACCTGCATCCCTAGCGGATGGACAAGGCAGGACTCCATACACCCGCCAACAATCGGGGGTCCTCCCCACCACCCAAGGGTGGCCCCTTCCTTGCCCCCTCCCCAGGGTCCCCGGGGTGGGGGCAGAGAAGGGGCCGGTCCCCACTGCAATGCGTCTGGAGAGCGGCAGCTGGAGACGCCCCCTGGAGGCCACTGTAGGAAGTCCCGGCCGAGCTCCAGGCAGGACCCTCACTGGGGGGCAACCCCCCCCCAGGCTGTCAGAGCCCGAGCATCCTCGTCCTCCCCCCCCCCCGCAGGGGCCTCCTCCCAAATCCAAGCCCTCCAGCCATGGCCACCCTGGTGATGTCCCGGGAGGAGAAGCTGAGTCAGGATGAGATCGTGCTGGGTACCAAGGCAGTCATCCAGGGGCTGGAGACCCTGCGGGGCGAGCACCGGGCCCTGCTGGCCTCCCTGCTGGAGAATGTGGCCGGGCGGGAGGCAGGAGAGGCTGAGCCCGCAACCCAGGAGCGCTCGGGGCTCCTGCGCCGATCCCTGGAAGCCATCGAGCTGGGACTTGGGGAAGCTCAGGTACCGGGGGGTCTGGGAGCAGGAGCATTTGAGGTCTAGGGGAGGCAAGCGTGGGAGAGGGGACCTGGAGTCCTGTCCTCCCACCCCGGCCGCCCCCTTCAGCCTTCTCTCCTGGCCGAGGGGCCATAGGTGATCCTGGCACTGTCAGGCCACCTTGGGGCCGTAGAGTCAGAGAAACAGAAGCTCCGGGCCCAGGTGCGCCGCTTGGTGCAGGAGAACCAGTGGCTCCGGGAGGAGCTGGCCGGGACCCAGCAGAAGCTGCAGCGCAGCGAGCAGGCCGTGGTCCAGCTGGAGGAGGAGAAGCAGCACCTGCTGTTTATGAGCCAGATCCGGAAGCTGGACGAGGACCCTTCTCCCAACGAGGAGAAGGGGGATGTTTCCAAGGATTCTGTGGATGACTTGTTCCCTAACGAGGAGGAACAGAACCCAGGTAGGGAGGTGGGGAGAAGGGGGTCTCTTAACTGTGGGCTCTGTGACTGGTCGGGAGGAAGGTGACCATCTATATGCCTCTCAGAAGGGGGGTGATTGCATATCCTGAGGACCCAGAAGTATGCAACAGATGAAGGAAGCTCCCAGGACTCCCCAGCATGCAGTTGCACTGCCCCAGCCGGTAGACAGCCCTTATTTCTGGGCCCTGGGGCCCCCTCTGACCTCCTGCCTTCCTCCAGCTCAGGGCCCTGGGGGTGGAGATGCTGCTGCCCAGCATGGAGGCTACGAGATTCCCGCCAGGCTAAGGACCTTGCACAATCTGGTGATTCAGTACGCCTCCCAGGGGCGCTATGAGGTAGCTGTGCCCCTCTGTAAGCAAGCCCTGGAGGACCTGGAAAAGACCTCAGGACATGACCACCCCGATGTGGCCACCATGCTGAACATCTTAGCTCTCGTGTATCGGTAAGGGGACGCCTGGAGCTCAGTGCTGAGGGGCACAGGGTGCGGACCTGCAGGCAGGAAGACACACTTAGGAGCTGAGTGACCCTAGGCAAGTCATTTCTCCCTGTGTGCCTCAGTTTCCTCATCTGTGAAACGAGCTAGAGAAGGAAATGGCAAACCCCTCCGGTATCTTTGCCAAAAAAATCCTAAGTGGGGTCACAAAGGGTCAGGCAAGACTGGACAATGATTGAATGAGAAGCAGCCTTGGATTCTGACCCTACTCAGGTGAAGGGAGGGGCCGGTGTGGGGTAATGAGCACCTCCTCCCCACCCCAAGAAAACCTTCACTGATGCCTGAAAGCCCCTCATTAGCCCATCACTCCTCTGAGTTGGCCCTCCTCCCCTTCCCCTGCCCCAAGCCCCTTATGGAAACCGTAAATCTCCTGAAGAAAGGGAGCATGTGTGTGCCCCGACGGCCCGGGCGAGGTCCTGGCTCTAGAACTGGCAGGAGTCTCATTTTACAGATGGGGAGCAGGCAGTCCAGGGAGGGCAGCGACTTAACCGAGGTCCCGCGAGTAGCCGTTGTGCTCTTCTCGGGGTCAGGTGGTCCTTCCCCTGCCCCCCATGCCATCTCTTTGTCCCCTCAGGGATCAGAATAAATACAAAGAAGCCGCCCACCTGCTCAACGATGCCCTGGCCATCCGGGAGAAGACCTTGGGCAAGGACCACCCAGCTGTGAGCAGGCCTGGCCTCCCTGGGCTGGGGGTGGGGGGTGAATGAGGGCCCTCTCTCCCCAGGGCCCTGCAGCTTTCTTCCTTCCTTCGCCCTGGTCCCAGGTGGCAGCTACCTTGAACAACCTGGCAGTCCTGTATGGCAAAAGGGGCAAATACAAGGAAGCAGAGCCCTTGTGTAAGCGAGCCCTGGAGATCCGAGAGAAGGTAGAGCCCGCTGGGCCTGTTTCCCCCCGGGGGCTGTAGGAGCAGCGGGGCGGCCGGGGTGGCGGCTGCTGGGTCAGAGCACAAGCGAAGGGAGCGTCCACCTGCTCCTAGGTGCTGGGCAGATTCCACCCGGACGTGGCCAAGCAGCTGAATAACCTGGCCCTGCTGTGCCAGAACCAGGGCAAGGCCGAGGAGGTGGAGTACTACTACCGGCGTGCTCTGGAGATCTACGAGGCCCGGCTGGGCCCAGACGACCCCAATGTGGCCAAGACCAAGAACAACCTGGCCTCTTGCTACCTGAAGCAGGGCAAGTACCAAGACGCAGAAACCCTGTATAAAGAGATCCTGACTCGTGCCCATGAGAAGGAGTTCGGCTCAGTCAATGGAGACAACAAGCCTATCTGGATGCACGCCGAAGAGCGGGAGGAGAGCAAGGATAAGCGTCGGGACACCACTCCCTATGGAGAATACGGTGGCTGGTACAAGGCTTGTAAAGTGGACAGCCCCACGGTGAACACCACGCTGCGGAGCCTCGGGGCTCTCTACCGCCGGCAAGGGAAGATGGAGGCGGCTCACACGCTGGAGGACTGTGCCAGCCGCAGTCGGAAGCAGGGCCTGGATGCCGTGAGCCAGACCAAGGTTGTGGAGCTGCTGAAAGATGGCAGCGGGCGAGGGGAGCGCCGGAGTGGCCGAGAGGGCGTTGGAGGCACGGGATCTCAGGCTGACCCACAGCCTGAGGATTCGGGGCCTGCAGCCGAGTGGGCCGGGGATGGCAGCGGTGCTCTCCGGAGAAGCGGCTCCTTTGGGAAGCTTCGCGATGCCTTGAGGCGCAGCAGTGAGATGCTGGTGAAGAAGCTGCAGGGGGGCGGACCCCAAGAGCCTCCCAACCCTCGGATGAAGCGAGCCAGTTCCCTTAACTTCCTGCACAAGAGCACAGAAGAGGCCATCCAGCCCGGCGACACCAACCTGTCCGACAGCCGCACGCTCAGCTCCAGCTCCCTTGACCTCTCTCGACGGAGCTCTCTCCTTGGCTAAGAGGGAGGGGTGGGAGGGCCTGGGCCCTCCTTGACTCCTCGTCATCTCCCGCAGGGACGGCAGGCGCAGGGGGCCACCTGATCCTCAGCCTCGCTCCCGCCCTGCGTAGGCCACCCTTCCCCCAAATGCTGTTTCCCACAGGTCCCCTCCCTCAGTGTTTTTTGTTTGATCTCAGGGTAACCTCTCCCCCTGTCATTTCAGGCTTAGCAGCTCAAAGCTTATTCCCCTCCCGGTCTAGCACTGTTAGTCACAAAACCGAGAAGCGTCACATTGGTGACAGTGCAGAGAGGAGCCAGGATTCCCCCCGCCCTTGTATTTATTTCCCTCTTCCCTTCCTCCCAGTGGGAGCCAGGAAGGTGAAAAGCGGGTGCCTGGCAGAGCTTCCCAGAGCAGGCTGGGCCACCTGGCCTTGGAGAAAGAGAATACCACCAGGCCTCCCTCTCCTCCCTTACCCAGGCCTCCGCTTCTGTATATAGAGAAATAAGTTATTGGCCACTCCCTTCCCCACTGCCTGTCCGTGGTACCTCCTCTAGCCCTTTCCCTGTCCAGGGATGCCACCCAGGCCTTAACCCCTTGCAGACAAGGTCTTGGACGCACACAAGTGCAGAAATATGCCCAAATGATGGAGCCTGCCTTCTCACCCCCTGAAACCCCATTAGTGTAGGATGGAAGTGCCATGCCCCCTACGGTACCTTCTCGTCTACAGTAGCTGACACCGTCTTCACTTTTCTCATCTTTGTTCCTTCCGGGGCATGGTGCCTGGGCCTAAAGCACTCCCTTTGAGCCACATTCCAGGGACTTCCGAAACCCTTCCCTGCCGCCCCCTCCCTCCCAAGGTACCGCCCGCTGAGCCATTCCTGGGGCTCCTACTGCTCCCAGTTCTCCCCTCCAGACTCTGAGGGAGGGGGCTGCACTCCTCTCCCGGAGCTGGGAGGGGGCAGGGTTGGGGTGGGCTGGGGAACCCATTTGCTTTGTTCAGATGTTGCTGT

>UoN.Pci.T.11697_UoN.Pci.T.11697.1

TTTTTTTTTTTGCTTTGCCTTTTTTTTATTTAATCAAAGTTAAGACCGGTGGATAAACATAAATACAGCACAATTACTTCAGATCATTCTTCATATTTGTGTACATACAGACTGATGAACTTAATCCCAGTGAAATCTGCAGTCAGCAATGAAGTGCATACAGCCTTTCCTCCCCTCACTCAAAAAGAATTTTTAAAAACCACACAAACTTATAATCTCATTGGCACACACTCTCTCATTACAAATGATTCATACATAAATAGGTTGTGGGTGCAAAGAAGCTAATATGCTAACATATGTATAACCTTTGTTAATGAAAAACAGAAGCAGCCATAATGATTAAAATACATTCAGTTACTGATAACTAGTTGTGCCATAAGGGGCATTATCCAAAGAGAAATCGTGCTGCAGAGAAAAGCTATAGATACACACACAATGAGAAAATAAACTGCTGCAAGGTTAATGCATGCATGTTTAATACTGGAAAAGCACAGCCCTCCAATATTTCTCCATGTTTGTTATAAAAGCCCTCTGCACTCAACTAAAATTAAAATGATCTTAAAAGGATAATACTTGTAAAGTTTACTTAAGTCTTTTCAGCCACAGAAACTGCAATGGAAATAAATGTTCAGAGTCATTTTCAAAACAAAAACAAAAATCTATTCTTCTGATGACATGCATGATTAAAAGGTCTATGCAAGATACATTGCTCTACATTCCCAATGACTAGGAAAAAAAAGCTCAAGTCAATTTAGTTTGCAGATGGTCAAAAGGATTTTTGTATTCCAGCAGGAGTTCAAATCTTCTGGATCTTTTCCCCAACAACTACTGGATTTTCTTTTCTGATTTTCTCAGCAGCATCAGCTTTGTAATTGTAAGAGCAATTGTGTACATCTGAATAACGGTGCACACCACAGTAAACATTTCCACACCGGCATTCAAACCCAGTCAGTCCCACTTTCTTCCTGCACATGAAACAGCGATTCTTTTTTTGTTTTGGTTTGTCAAGTGACTTGTTTTGCTCTTCAGAGGGTTGCTGTGCAGTATCCGATACTGAAGCTTGCAGATCGTCTGTTTCAGGTATTGCTTTGTCCACAGATGTACTTTCCACTTGGGTAGGTGCTACAGAGTCTGATAAAAGTGACTGATTTGATACAGGGCTTGGCTGCATAGATGGGGAAGATGTGGAGTCTAATGATTGAGCTTCTGGGACATTGCCTTCTGTGCACTGAACTGGTAAAGATTCAGACAGACTACTGACAGAAGTTGCAGGTGGGCTAATTCTACCATTACTACTATTCTGTCTTTGAAGATGTTCTTTATAGCATACTGAACACATGCCATTTGTACGAGGGTTTCCATAAAATCCACAGCCAGTGGAACAAAGCATAGGCACTTGGCTATGGTTAGTTTCTTGAGCCATGTTCCTCTTCTGTGCACTCTGGTGAAAAAGTATAGGGATTTTGGATCTGGAGTACAGGCCTTCTACTGTACTGAGGATGTCTTGCAGTTCTCTCTCTGTGTAGCTGTCCCCACTTCCCTATGTCAGGCTGTCTTTCCTTCTGCAACTTGGGCTGGCGGCGGCTCCCTCCGCCGGACACGCCCGCCACAACCGCTGCCGCCGCCGCCGCCGCCCCCGCCGCGGGTGAATGGGAACAGGTGAGGCCGTCCAACCCCCCGCTCGCTCCGGCTCCGGCTCCGGCCCCGCGCCCCCTCCTCCCGCCGGCCCCGCGTCTTCGGGAGTTTGCGGTGCCTTGTTGACATCCTGGAACTTCGGAAAAGGACGAAATCTGCTCTTAGGGGATAATGCTAAGGGAAGCTATGAAGTCTTGGAGTGATAGCCAGTCAGACCTCTACAGCAGTGACCAAGAAGAGGAGGAAGAGATGATTTTTGGTGAAAATGAGGATGATTTGGAAGAGATGATGGATTTAAGTGATCTGCCTACCTCACTTTTTGCATGTTGTGTACATGAAACTGTGTTTGAGGTGCAAGAGCAGAAGGAGAGATTTGAAGCACTTTTTACAATCTATGATGACCAAGTTACATTTCAGTTATTTAAAAGCTTTAGAAGAGTCAGAATAAACTTCAGCAAACCTGAAGCAGCAGCAAGAGCCCGGATAGAGCTTCACGAGACGGACTTTGATGGGAAGAAGCTGAAACTGTATTTTGCACAGGTCCAGATGACTAGCGAGCCAGGAGACAAAGCGTATCTGCTTCCCCCGCAGCCCGTCAAGCAGTTCCTGATCTCCCCGCCGGCCTCTCCTCCTGTGGGGTGGAAGCAGGGTGAAGACGCGATGCCTGTCATAAACTATGACTTACTTTGTGCCGTTTCCAAGCTGGGTCCTGGAGAAAAATACGAGCTTCACGCAGGGACCGAGTCTACCCCAAGCGTGGTTGTTCACGTCTGTGAAAGCGAGACTGAAGAGGAGGAAGAAACGAAAAATCCCAAACGGAAAATCGCCCCAACCAGGCGTCCTGAAGCGCCTTCGATGATCCGAAGCGATCCTCCGGCTTTTGGCTGTACACTGTGAAGGTAGCAGTGGCAGGAGGGACCTCACATGGTCTTGGTCGTGAAGAGCAGCTTTCTGCCCCCTGCTGCTGGCGTGGGTCAGTCAGAGTGTCAGGGGATGGACGTGCTGTGCTCAGCAGTCCTGTGCTCTTCTGAAAGTCGTGTACATTTTACATAGTACAGGGTAACAGCAGATTTCACTAGTGATTGTCTTTCATGTTCGAATTTTAGATGATATTCTAAACAGCACTTTAATTTAAAGGATAGTACCGCCCCCCAGTGTAGCATCTCTGACATATTTTACTAGTGGAAAATTAGGAAGTTGGTTTTATTTTTTCCAGCAATGTTAACTTCTCTAATATCATGTAGCATGGGGTACCAAAAACTGGAACTATATTCACCCAGTACAGTACCATTTTATGGATTTAAAAATCTAGTCTCTAGCTTAACAGTTCAGCGTGGGTATTTTTGTACTGGTATACTAGCTTCTAACTAATGTATTGATCAAATGTTAATGCTATTCAGTATATACTAACATTTTTGCACGGCTAGAAGTCTAATGTCTAGGAGAATTAATAGAAAATGAATCGGCCCATTTCAAAGTATTTTTTCTTCAACTTTTTGGAAAGAAATGCCAAATTCACCTTGATTGATGAAAACTTTGGCATCTTTCCATGTGCATATTGTGTAGTGCTCTACCCTTAACAGAAAGTCTGGAGGGAAGAGATACACAGAACTCCCTTCCTGATGCCTATGCCTTCTTAGAGGCCGGCTCCCTGCGGGACACCTTGCCAGTAGGCATGATTGACTGTGGTGTCAGATGGCTGATCATAGATTGCCATTTGCAGCTAACTGTGTTCCTGTCTAGAGTAAAAACTTGCTTTCAGCCGTTCAGTGTTAGTCTGTTAACTTCTCTATTGTTGTACTGATCACTGGTCCTTGAGTCTGTTTGTTTTGTTATGTGTGTTTGGTTGTGTCTGTCCCATAATCCAGAGAAAAAGAGATGACCGATTTTCTGAAGGTGGCTTGTAATTTGAAAATAAGTCTACACACTCTCAGTTTTCTGTTTCTAAGAGTAGTGGGGAAGAAAGAAATCGGCTGACTCATCACATTTGCTTTGGGGATGAAGCATGTCTGCTTGTGTTCACTAAAGTACAGGACGGCTTCTCTATTGTGCACGGGATACTTCTGAGAGGAAAAGCACCTTTGGAAGTGCCCTTCAGTGGGCTCCAGATATTTCTGGTACCTGTTACGGAGCATTACCTGTTCTAATGACAGTAAGATGGATTTCTGTGGGCTTATGTTTCACTGCAGATACTTTTTTTTTTAATCTTCCAAATGTCCCTGGTCCTTCAGCATTCTGAAATCTTCCCAGTTGCATGGGGACACACACACACACACACACACACACACACACACACACACACACACACACACACACACACA

>UoN.Pci.T.10351_UoN.Pci.T.10351.1

TTTTTTTTTGCATTGTAAACTTTGTTTATATTTCAAAAGAAAAAAGATATGATTGCAAAGACACTTCCAACATTTTTGGCTGTACAACTAACAAAATTTTTATTTGTCATCCTTCCCCTTTGTCTTTCTATATACCATTTCTCGAAAACTGGCTAGAATCTTGTTTTCTCTCTTCCTTCTCTCTTCTTGGTTAAAGGATGCTAAAGCTCGCTTCTCATCAGCACTGTAGATCTGGTTTTCTTTTCGAAGTCGCACAGCTTCCATTCTTCGGTGTCTACTACCACTCATTACATAGCCTGAACATTCAAATGATGCAATCTCTTCACTAGTCAAGCCAATTTCTCCTCTTCGAGGGATACGCTTTCCAGCTTTGACATACTCAGCCATGGCTGCACCTTCCCCCGGCAACAGAGCATGCCCATAGTTCAAAGGTCTATCATCTTGAGAGGCATGAGTCACAGGTGCTTCTGGTCCAATTAAATCCATAGCTTCTGCATTCTTTGATCTCTCAATCCAAAGATCATCTTCTGGAAACTCTTCACCAGAGACATCAGAACTTGACTCAGTAGATTCTTTTTTATATTTCTTCTTCTTGGACCTTTTCACTTTGTGTTTCTTTTTCTTTTTCTTCTTGGCTTTCTTTGCCTTTTTTCTCTCTTCTTCACTGCTAGAGTCAGAGTCAGAATCAGAGTCACTGTCACTATCAGAATATTTTTTATGTTTTCTTTTGGATGCTTTTTTCTTTCTTTTCTTCTGAGCATTTTCTTTGGAGCGATTCACCTTCTTCTTTCTCCTCTTCTTATCTTCCTCAGAACTGGAACTTGAAGCACTTTTCTTAGACTTTGAATCCTCCTCCTCTACAGGGGTATGTTCATCAGAATCAGGCTCGGGGATCTTGGGCGAAAGTCCCCAGACCTCCGGGGCTCCAAGCTCCCCGATCCGCTCCCGCTCATTTAACCTCCTCTGGCGCAGGCTCTCCTCCTTTTCCTTCTCGTAGTAATCCGGCCATTGCCGGTCGCCCCCATGGTGATGACAGTGGTGATGGTGATGGTAGTGGGCGGAAGATGAGGACGAGGAGCCGCGCAGATCAGGGAGCCGCTCTCGCTCGCGGCCGTGGCTCCGGCTGGGGCTACGGCGGCTGCGGCCTGGGCCTCCCGGCTCCGTGAAGGACGACGGCTGCCGGAAGCCGTTGCGCTCCCGGGAACGAGAACGAGAGCGGCCCCGCGGTGAGCGGATGGCTTTGGTGTTGGGGGGGCTCTCGGAGGAGCTTCGATGGCCGCCGCCGCTTCCTCCCGATGCTGCAGCTGATAGTCCGCTACCGCCGCCTCCGTGACGCCGCCGCCGGGAACCTGAGGCATCCTCCGGGCTGCGAGAGCTGGAGGCCGGCGCCATAACACCGAGCTGCTCCGCTGAGAGCTCCGCCGGACGCTCTCGCGAGAAGAAAAATCGGAAGCACCCTTTACCTTAACTCCGGTACGAGAGACCGCCTCAGAGGGGCGTGGCCCGTTTCTAAAGCGAGGGATAAGCAAACTCCGGCGTCTTTCCTAAGGTGTCGCACAAGGATTCAGTTTTCATTGGAAAGACCGTCCCAGAAGGACGTGGCCCCGCTTGAGGCAGGAATTAGCGAATTACAGCCTGTTAGCACACAGATATTGTTTCCATTGGAGAGAACTTCCTGGAAGGGCGTGGCCCAACTTCGGG

>UoN.Pci.T.10431_UoN.Pci.T.10431.1

TTTTTTTTTTTGCTTTTTAAAATGTTTATTGATATTAAGTGGATGGAATGGTGGTGCGATAGTGCAAAGAAATGTGTGCAATTCTGGGGCCTTGTTTTTCCTAATTTTCACAGCTGAACGAGTCAAAGAGGATTAAAAAAACACCAAGGGAAGCTGAGGGTGGGCAGAGGATGTAAATACACTTGGACTGGCCACTTGCTAGCCAGCTCAGGTGGTGTGGTTCACGGTTGGGTCAGAGCTGGAAAGGTCTTCCTCACTCCATTCACTCCAGGTCAACACATAGGGGAAGTAGACCACACGTGTGTGGAATGAGGGTGGGTTTATTTCAAACATGTTCAGCCAGTCTAAACTATATGGAGAGAGCTGAGTCTCCTGTTCATAAATTAGCTTCACGAGTTAGCATTACTATTCCAAACCAGCATTATTTACAAAACGAACCTTTTACCTGCTCTAGCCTCAGGTTCATAGGACAAGTTATTAAAGAAAACTTCTGGCTAATTTAAAAAATCTCTCTTCCATTCAACACCCAGCAACTTTTGAGGGGCAGGGCCTAGGGGGGGTGTTCGACTTTGTGACAGCAACAATCACCATTCACCAGGAATGCAGAGTCACAAACTAAATGACAGTAAATTTAAGTCACTACAGATTGTTTGCTCCCAGTTAGCTTGTTATAAGAATGCCACTTTGAAACAGGAGTGATCACTCAGTCATCCTCTTTCTACAGATACATGAGAGAAGGGCCCCAGGGAGTGCTAATAAATATATTGGTTAACAAGCTCATTTGAAAACCAAAAAGTGCTGTCTGAACACAATCCAAATGAGGCGACCATGGACTGACCTTAGCAGAAAACTGAAGGATGAAACTCGAAATAGGAGGTGTGCCCTTAATTAAGACTGACCAGTTTGCACTTTCAGCTGAAAACACAGGGCTGAAATCTTCCAAAGCAGATGGAGGATATTTTTCTTGGAAATTCCAAATCAAAATGGCAAGTAAGAAAAATTGCTGGCTTATAAAAGGTTCCTGATGGCCTCCATTACACTGGGTGCCAGAAAATCCTTCTGTTCCGGTTGCTGGTAAAAAGGCCGTTCTGGCACACACATAAAAAGACGATAGCACAAGAATATAGTGTATTACCTGTCAAACAAGAATTTCAAGAACGGAATCTTCGAGGACTCACTCATCAGTGAACATTTGGGACAGAGAGCAATCAGAATACAAAAGAAAGCAAAAACCCACATAAGTTAAATTCCTAATTTGCAAAACTGATACAATTTGTACATAAACTGATAAACACCATCTTCATGGCTCACCAATCCCAAGTCCTAGTCACCAGCGGTTAGTTTCTACCTACATTACTAACAGGGAGCTCAGGGATGCTCTCTTGAGGGGATTGGTTTGCTCTGGAGATGCCGACACACTCTTGCTTGGAAGCTGTTCGGCAGGAGTGGACTCTGAGGGAAACAGCCACTTCATTTGGATAACCACACGATAATGAATATCAAGATTCCCATAGCCACTGCTAGCACAAGCACCAGGATGCAGATCTTCTTCCGGGATTTTTTCTGATAGTACGCGGCTCGCTGTAGCTGGTCTGTGGCTCTTTCAACGTGCACCTCAGAGCTCTCCACATTGGCTTCTATGCTATCTGAAAAAGCAAGAGACATTGGCTGTTAACACACACAGAGTCACACAGAGTCATACACACACACACACACACACACACACACACACACACACACACTGAGAGCCCTCCCTGCTGTTCCTCTGGTGCTCCTCTCTCTGCTGCTTGTATATACTGACAGTGCAGAGGAGTTAGGAGCCGAGTTTAGGGAGCTGAAGCTTAGGCACAACAACCGTGGCAAAAAACAGCTTGGCAGACACTTAGGTGACAGAGTTAAGACAGAAATAGGCGAATGGAGTTTAGAATGTAAGAAAGTCTGCCATGATTCTTACTTTTAAAACGAGAGGATATTTATTATGGGAATTCTGTTCAATATGTCCAACAATACACAAGGCTCTTGCACACAGTATAACTGTATTCTTTTTCAGCGCAAATGCAAACACACAGTGAGGCATTTTCAGGGCCCCCCCCTTTCTGTTTTTTGCTAAATCAACCACAGTACATACCAATCAAATCTCCCTGATCATGGATCATCATGGCCAAATCTTTAAATATCTGATTGACGTCCAAAATGTCGGCCTGTAGGACACAACAATCTTAAGCAATGTTTTGATTCATTTTCTCCATTCCTTCCTCACGCCTGTTCCATGCTTCAGTTGTTAACATGAGAGGAATGCATATGTTTGCAAGTATTTATAAACATAACCTTAGGTACTCAGGACTATTTCTCCACAAATTGTGAGCAAAGAACATGAAGTAATTAATGATGGTCAAACAGTTGAATGATGAGGACTGAAGCCACAGGCAGAGGGTTTAGAAAAGAGCAAGAGGACAGGATGTGGAGGGCACTGTAGATGACTATCTCAAGGAATTTAGCCATGAAAGAGAGGAGAGATGTAGGATGATAGTAAAGATGGTCTGGTTGGGTCAACTGAAGATTTTTTTCTTTTTAAAGGAAGGAGATGTATGTTTGCAAGTAGCAGGGAGGCAGCCAGCAGATGGGGAGGGATTGAAGATTAGTCAAAGAGCAAGGATGATAGAGATGGCACTATGCTGGAGAAGACAGGATACAATGGGACTAAGGGTGCCTGGAGAGCAGGCCTGTACCACATATGACCACAGGGTGATATGTTGTACAAGCCTGTTTTACATCCCCTACATTTTTCACGGGTTGCAGGCCAAATGTTGAGCAGGCCTGCTGTAGAGGGTTTTGCCCTTCTAGAGTACAGCTGTCCTTGCCATGATTCCCACAAACAGACACTTATGGAGCACAGCGACTTCTGCTAAAAAACTAGTTTTGAAAAAGTGAATTTGTTCCAACGTGACTGATATATTAGGACGTGCAATTTCACTTGCAAGAAACGGCAAGACTATAGAAACCCAAACCTAGCTGAACGAATCACCAAAGAATGCACAAAACGCACACGCTTCAAACACATACCAGCTACCTCAGTTCACATGCACATTATTGTATAAGTTGTGTTTTGCTACTCTTTTGCATTTGTGAACTTTTCTATATAAAAAAACATTCTTTCTACAGCTTAAGCTGAATGAGGCTCAGACTCAGGTCTTTAAGGCCGCCCTCTCACCTGTACCCACTGACTTAGCCAGCTTGGGCCCCACCGGAACTGGGTGGCAGACGGAGGACTTATGGCTCTATTTCATAAACTCATGTGTTTTGAGAGAAATTCCTGGGGGAATAATACAACAAAAGGACTTCAATAGCTTCATGGACAGTGCAAAATGCAAAGGCCAGGACCCTCCTCTTACCTCCAGTTGCCTGATCGCAGTTTCTCGCTCCTTGATAAGTTCAAGGTCTTGCTCTGTAATGGCCAACTCATCTTCCTGTGACTGCATCTGATTCCACTCTTCATTGCTGTCAAATGAAACAAGCTGCTCCTCTCTCTGCCTCTCCTCCGCAGAGAGACGAGATCCCGCTCTGGCTCGGGCGACAGTCTCCTTCTCCTTCTCAGACACTTTCCTCTGAACCGCCTGGAAGTTGTTTAAGGCTATAGAGAAGTCATTCATCAAACGCTCCTTCTGAAGTTTCTGCAGGCGCTGTTCTGAAGCCGACAGAGGGAGCGGCAATGATCCCAGCTCTTTCAGGCATTCATTTGTCTCCTTAGCGAGCTGATTTGTAGAATGTTGCAGTTGTTGTAGATCTTCCTGCAGTTTGCTAGAATCTTGCTTAGTCCCCAGCTGGCTCATCAAATTCTTGATCTGAGCAGTGGCGTAGCTGATCCTCTGGATGTGGCCGCAGCAAGTATTGAAGATGCTGTCAAAGTCCCGGGGCTGGGGGCCCTGCGGGCCGGAGGGCCCCGCCGCCCGGTACATGTCTAGGGGCCCGTACGACATGGCGAGGGCTGCCTGGCCGGGCCGGGGTCGATCTGGACCGATCTCCAGAAGGAAGAGGACCCGCCGACCAGTGACAGGGAGGAGGAGGAGGAGGAGGAAGATAGAGCCGCGGTGGGGGGGAGGACAGCGGTGGG

>UoN.Pci.T.12486_UoN.Pci.T.12486.1

ATTCCTTTTTATTAATATACACATCAGGCTACTGAACGTGAATTCAAGTAGCTTTTCCCTCAAAGATGGCACTTGCAGATAGAAGATGCAGATGACTACAACCTTCAGAAGTTTACTTGATAAGATTCTTCAACAGCAGTTTGATATTGTGTTTCCTCATCTAACTGAAGATTCACAAATTCTCCATAATCAAATTGATGTCTTTGATTTAGATGGCTAACAAAATTTCTAGTAATCTGGCTAGGATCTCCCCATGGAAGAGACACACAAATAGGACACGTCACAGGAACTATCTGAAAGAGGTGACTATTATTACAATGATCCAGTAAACGCTGTCTTGTAAAGTTTGCTTCTTGACACAAGGGACACTTAAATGTGGGATGCCCAGTAGAACTTCCATCTCCTTGATAGGTCTCTGTATTATCCGAGGCAGATGTCTCACTCCTATTACTATTCCCTACTGAATCTTGAGAGATCTGAAAGTTTGGAATGATGGAGGACACGCCATACTCATCCTGATACTTCTTACAAGATTTGTAATGATGTCTCATGCGATAAAATTTGATCTGTTTTGCACAGCATCTGCAGCTACCAGAAAACTTCCTCATGACATTTTCAATGTCTAAGGCCCGTTCAGGACATGCTCGCTCTCTTCTCGTTACATTTCCACGACAGAGGGGACAATGTATTCCACTTTCTCTCATAGCAGTCAGGAAACATTTTCTACAAAAAACGTGCTGACAGGCCGCGGTCCGCACTGGGGTCTTGAACACCTCCTGACAGACCGGGCAGTAGAAGTCGTCTTCGGTGTAGGACGTGGCGGTGGAGAGCTTCTCAGCCATGGTGCGGGGCTGGCGGGAGGTGGCGATGGCGACTGCGGCGGCCCCGGGCCTGGCTCCCTGCGTGCACCGAGGGGAGGGGGAGGGGGAGAGGAAAGCGGCGGCGGCGTCAGGAAGCGGCTCGCGCCAGGAAGGGGGGGGCGGGGAAGGGGGGAAAGGAGTAGGAGGAAGAGGAGGAGGAAGAAGATGAGGAGTCTGGCAATCAGTCCGATAGGAGTGGTTCTAGTGGTCGACGAAAAGCCAAGAAAAAATGGCGGAAGGACAGTCCTTGGGTGAAGCCCACCAGAAAACGTCGAAAGCGGGAAACACCACGTGCCAAGGAGCAGCGGGGGGTGAATGGTGTTGGTCCCTCTGGCCCCAGTGAGTATATAGAGGTGCCTCTGGGGTCTCTGGAGCTACCCAGCGAGGGGAACCTTTCTCCCAACCATGCTGGGGTGTCAAATGACACATCATCCCTGGAGACGGAGCGGGGGTTTGAGGAGTTACCCCTGTGCAGCTGCCGCATGGAGGCTCCAAAGATTGACCGGATCAGCGAGAGGGCTGGGCACAAGTGCATGGCCACAGAGAGCGTGGATGGAGAGCTGTCGGGCTGTAGTGCAGGAATCCAAAAGCGGGAAACAATGAGGCCTTCGAGCAGAGTAGCTCTGATGGTCCTCTGCGAGACACATCGAGCCCGGATGGTCAAGCATCACTGCTGCCCAGGCTGTGGCTATTTTTGTACAGCGGGCACATTCTTGGAGTGTCACCCTGATTTCCGAGTGGCCCATCGATTCCACAAAGCCTGCGTGTCCCAGCTGAATGGGATGGTTTTCTGCCCCCACTGTGGGGAGGACGCTTCTGAGGCCCAGGAGGTGACAATTGCCCGGAGTGATGGGGTGGCCCCACCTCCTGGCACTGCTGCTCCTGTCCCCCCACCCCCTGCCCAGGATGCCCCTGGACGGGCCGACACTTCCCAGCCCAGCGCCAGGATGCGAGGTCATGGAGAACCAAGACGCCCCCTCTGCGACCCACTGGCTGATACCATCGACAGCTCAGGCCCTTCCCTGGCATTGCCCAATGGCAATATCCTCTCAGCTGTAGGTCTAGGGGCTGGCCCAGGCAGAGAAGCCCTGGAAAAGGCACTAGTCATCCAGGAGTCCGAGCGAAGGAAGAAGCTTCGCTTTCATCCCCGGCAGTTGTACCTGTCTGTGAAGCAGGGGGAGCTGCAAAAGGTCGTCCTAATGCTCTTGGACAACCTGGACCCCAACTTCCAAAGTGACCAGCAGAGCAAGCGCACACCCCTGCATGCAGCCGCACAAAAGGGCTCTGTTGAGATCTGCCATGTGCTCCTGCAGGCTGGAGCAAATATCAATGCTGTGGATAAGCTGCAACGGACCCCTCTGATGGAGGCTGTGGCCAACAACCACGTGGAAGCCGCTCGATACATGGTACAGCGAGGAGGCTGTGTCTATAGCAAGGAGGAAGATGGTTCTACTTGTCTTCACCACGCAGCTAAGATTGGCAACCTGGAGATGGTCAGCCTGCTGCTCAGCACTGGCCAGGTGGATGTAAATGCTCAGGATAATGGGGGCTGGACACCCATTATTTGGGCAGCTGAACATAAACATATTGAAGTTATTCGGATGCTGTTGACCCGAGGAGCAGATGTTACCCTCACTGACAATGAAGAGAACATCTGTTTGCACTGGGCATCCTTCACAGGCAGTGCTGCTATTGCCGAGGTCCTACTGAATGCCCGCTGTGACCTCCATGCAGTCAACTATCATGGCGACACACCCTTACATATCGCTGCTCGAGAGAGTTACCACGACTGTGTGCTGCTTTTCCTGTCCCGGGGTGCAGATCCAGAGTTACGGAACAAAGAAGGAGATACGGCATGGGACCTGACCCCTGAGCGCTCAGATGTCTGGGTGGCCCTGCAGCTCAACCGAAAGCTTCGCCTTGGTGTGGGTAACCGAGTACTGCGGACAGAAAAGATCATTTGCAGAGACGTAGCTCGAGGTTATGAGAACGTGCCAATCCCCTGTGTCAATGGAGTGGATGGGGAGCCGTGCCCAGAAGATTACAAATACATTTCTGAGAACTGTGAAACTTCAACTATGAATATTGACCGAAACATCACACACCTGCAGCACTGCACCTGCGTGGATGACTGCTCAAGTTCCAACTGTCTCTGTGGCCAGCTCAGTATACGGTGTTGGTACGACAAGGATGGACGTCTGCTTCAGGAGTTTAATAAGATTGAGCCCCCCCTGATCTTTGAATGTAACCAGGCCTGCGCCTGCTGGAGAAGCTGCAAGAATCGAGTGGTACAGAGTGGTATCAAGGTTCGGCTACAGCTCTACCGTACCGCCAAGATGGGTTGGGGTGTCCGTGCATTGCAGACCATTCCCCAGGGTACCTTCATCTGCGAGTATGTTGGGGAACTGATCTCAGATGCTGAGGCAGATGTTCGAGAAGATGATTCTTACCTCTTTGACCTGGACAACAAAGATGGAGAGGTGTACTGCATTGATGCCCGATACTATGGCAACATCAGCCGTTTTATCAATCATTTGTGTGACCCTAACATCATCCCAGTTCGAGTCTTCATGCTGCACCAAGACCTCAGGTTCCCCCGAATTGCCTTCTTCAGTTCTCGAGACATCCGGACGGGCGAGGAGCTAGGGTTTGACTATGGTGACCGCTTCTGGGACATTAAGAGCAAGTATTTCACCTGTCAGTGTGGCTCTGAAAAATGCAAGCACTCAGCAGAGGCCATTGCCCTGGAGCAGAGCCGCCTGGCCCGTCTGGATCCCCACCCAGAACTGATTGCTGACCTTGGGTCCTTGCCCTCCCTCAGTTCCTGACCCCAGATCCAGTGACTTCTGGGGGGCTTCCTCACAGAGTTTCTTCTCCTGGGAATTGTTCTCACCTCTCAGAACTAGCTGCCTTTTTCCTGCCCACACACTGTCCCAAGCCATTCCTGTTCATTTGTATCACCTGTTTTGAAACTGGGATACTCTTCCCCATCAGCAAGGAATGATGGTGTCCTGTTCTGCCATAATGATTGATTCCCAGACCCAGTTCCCCACTTTGAGACAAAAGTGACCTCCTTTGTATTCCCCACTACCTATCAGAGAGAGACAGACAGACACACACACACACCCGCAATTGGATTAAAGAAAGGGCATCTTTGGGGATCTGAGCACCCAACTGGGGTGTGTCTCCAGCAGCAGCCTCACCTCCCCAGAAAGTCAAGGGCAATCTGTGCTCTCCCACCCTGAACATTGCTTCCTCCCCCATATCACTTGACCGTCATCACCCCACTCCCTGACAATTTTTTTTTTCTCAATAAATGTTTTGGTTTCGTTAAAAAAAAAA

>UoN.Pci.T.12486_UoN.Pci.T.12486.2

GGGCGGGGAAGGGGGGAAAGGAGTAGGAGGAAGAGGAGGAGGAAGAAGATGAGGAGTCTGGCAATCAGTCCGATAGGAGTGGTTCTAGTGGTCGACGAAAAGCCAAGAAAAAATGGCGGAAGGACAGTCCTTGGGTGAAGCCCACCAGAAAACGTCGAAAGCGGGAAACACCACGTGCCAAGGAGCAGCGGGGGGTGAATGGTGTTGGTCCCTCTGGCCCCAGTGAGTATATAGAGGTGCCTCTGGGGTCTCTGGAGCTACCCAGCGAGGGGAACCTTTCTCCCAACCATGCTGGGGTGTCAAATGACACATCATCCCTGGAGACGGAGCGGGGGTTTGAGGAGTTACCCCTGTGCAGCTGCCGCATGGAGGCTCCAAAGATTGACCGGATCAGCGAGAGGGCTGGGCACAAGTGCATGGCCACAGAGAGCGTGGATGGAGAGCTGTCGGGCTGTAGTGCAGGAATCCAAAAGCGGGAAACAATGAGGCCTTCGAGCAGAGTAGCTCTGATGGTCCTCTGCGAGACACATCGAGCCCGGATGGTCAAGCATCACTGCTGCCCAGGCTGTGGCTATTTTTGTACAGCGGGCACATTCTTGGAGTGTCACCCTGATTTCCGAGTGGCCCATCGATTCCACAAAGCCTGCGTGTCCCAGCTGAATGGGATGGTTTTCTGCCCCCACTGTGGGGAGGACGCTTCTGAGGCCCAGGAGGTGACAATTGCCCGGAGTGATGGGGTGGCCCCACCTCCTGGCACTGCTGCTCCTGTCCCCCCACCCCCTGCCCAGGATGCCCCTGGACGGGCCGACACTTCCCAGCCCAGCGCCAGGATGCGAGGTCATGGAGAACCAAGACGCCCCCTCTGCGACCCACTGGCTGATACCATCGACAGCTCAGGCCCTTCCCTGGCATTGCCCAATGGCAATATCCTCTCAGCTGTAGGTCTAGGGGCTGGCCCAGGCAGAGAAGCCCTGGAAAAGGCACTAGTCATCCAGGAGTCCGAGCGAAGGAAGAAGCTTCGCTTTCATCCCCGGCAGTTGTACCTGTCTGTGAAGCAGGGGGAGCTGCAAAAGGTCGTCCTAATGCTCTTGGACAACCTGGACCCCAACTTCCAAAGTGACCAGCAGAGCAAGCGCACACCCCTGCATGCAGCCGCACAAAAGGGCTCTGTTGAGATCTGCCATGTGCTCCTGCAGGCTGGAGCAAATATCAATGCTGTGGATAAGCTGCAACGGACCCCTCTGATGGAGGCTGTGGCCAACAACCACGTGGAAGCCGCTCGATACATGGTACAGCGAGGAGGCTGTGTCTATAGCAAGGAGGAAGATGGTTCTACTTGTCTTCACCACGCAGCTAAGATTGGCAACCTGGAGATGGTCAGCCTGCTGCTCAGCACTGGCCAGGTGGATGTAAATGCTCAGGATAATGGGGGCTGGACACCCATTATTTGGGCAGCTGAACATAAACATATTGAAGTTATTCGGATGCTGTTGACCCGAGGAGCAGATGTTACCCTCACTGACAATGAAGAGAACATCTGTTTGCACTGGGCATCCTTCACAGGCAGTGCTGCTATTGCCGAGGTCCTACTGAATGCCCGCTGTGACCTCCATGCAGTCAACTATCATGGCGACACACCCTTACATATCGCTGCTCGAGAGAGTTACCACGACTGTGTGCTGCTTTTCCTGTCCCGGGGTGCAGATCCAGAGTTACGGAACAAAGAAGGAGATACGGCATGGGACCTGACCCCTGAGCGCTCAGATGTCTGGGTGGCCCTGCAGCTCAACCGAAAGCTTCGCCTTGGTGTGGGTAACCGAGTACTGCGGACAGAAAAGATCATTTGCAGAGACGTAGCTCGAGGTTATGAGAACGTGCCAATCCCCTGTGTCAATGGAGTGGATGGGGAGCCGTGCCCAGAAGATTACAAATACATTTCTGAGAACTGTGAAACTTCAACTATGAATATTGACCGAAACATCACACACCTGCAGCACTGCACCTGCGTGGATGACTGCTCAAGTTCCAACTGTCTCTGTGGCCAGCTCAGTATACGGTGTTGGTACGACAAGGATGGACGTCTGCTTCAGGAGTTTAATAAGATTGAGCCCCCCCTGATCTTTGAATGTAACCAGGCCTGCGCCTGCTGGAGAAGCTGCAAGAATCGAGTGGTACAGAGTGGTATCAAGGTTCGGCTACAGCTCTACCGTACCGCCAAGATGGGTTGGGGTGTCCGTGCATTGCAGACCATTCCCCAGGGTACCTTCATCTGCGAGTATGTTGGGGAACTGATCTCAGATGCTGAGGCAGATGTTCGAGAAGATGATTCTTACCTCTTTGACCTGGACAACAAAGATGGAGAGGTGTACTGCATTGATGCCCGATACTATGGCAACATCAGCCGTTTTATCAATCATTTGTGTGACCCTAACATCATCCCAGTTCGAGTCTTCATGCTGCACCAAGACCTCAGGTTCCCCCGAATTGCCTTCTTCAGTTCTCGAGACATCCGGACGGGCGAGGAGCTAGGGTTTGACTATGGTGACCGCTTCTGGGACATTAAGAGCAAGTATTTCACCTGTCAGTGTGGCTCTGAAAAATGCAAGCACTCAGCAGAGGCCATTGCCCTGGAGCAGAGCCGCCTGGCCCGTCTGGATCCCCACCCAGAACTGATTGCTGACCTTGGGTCCTTGCCCTCCCTCAGTTCCTGACCCCAGATCCAGTGACTTCTGGGGGGCTTCCTCACAGAGTTTCTTCTCCTGGGAATTGTTCTCACCTCTCAGAACTAGCTGCCTTTTTCCTGCCCACACACTGTCCCAAGCCATTCCTGTTCATTTGTATCACCTGTTTTGAAACTGGGATACTCTTCCCCATCAGCAAGGAATGATGGTGTCCTGTTCTGCCATAATGATTGATTCCCAGACCCAGTTCCCCACTTTGAGACAAAAGTGACCTCCTTTGTATTCCCCACTACCTATCAGAGAGAGACAGACAGACACACACACACACCCGCAATTGGATTAAAGAAAGGGCATCTTTGGGGATCTGAGCACCCAACTGGGGTGTGTCTCCAGCAGCAGCCTCACCTCCCCAGAAAGTCAAGGGCAATCTGTGCTCTCCCACCCTGAACATTGCTTCCTCCCCCATATCACTTGACCGTCATCACCCCACTCCCTGACAATTTTTTTTTTCTCAATAAATGTTTTGGTTTCGTTAAAAAAAAAA

>UoN.Pci.T.12547_UoN.Pci.T.12547.1

GAAGGAGGAAGGCGGAGGGAGTGGAGGTGCCCCCGCCCCCGCCGCCCAGCCCGCCAGTGGCTGCCTCCTTTTCCGTGTCTCCACATCCTCTGCCCATCTGACCGTCGGTGGGCCCCGGAGCCCCAGCCCAGCCGCACCCCGGCAGCCGCTGCCCCTGGCCAGGGATGGAGATCCCCCCGACCCACTTCCCCGCAGCCCGGGCGGCCTCGGTGGCGGAGAACTGCATCAACTACCAGCAGGGGACCCCGCACAAGGTGTTCCTGGTTCAGACGGTCACCCAGGCCAGCCTGGAGGACATTCCAGGAAGTGGACATAAGTACCACCTGAAATTTTCTGTGGAAGAAATCATCCAGAAGCAAATTACCGTGAACTGCCGAGCACAAATACTCTACCCAACAGCTGGACAAAGCACCGCACCAGAAGTGGACTTCACGTTTGAAGGGGAGATTGGGAAGAACCCAGATGAGGAAGACAATAAATTTTATGAGCGACTCAAATCCATGAAGGAGCCACTGGAAGCAAAGAATATTCCAGACAGCTTTGGGAACGTCTCGCCAGAAATGAAACCAGTCCGACACCTCGCTTGGGTGGCCTGTGGTTACATAATATGGCAGAATTCCACTGAAGACACGTGGTATAAAATGGCAAAAATACAAACTGTCAAGCAAGTGAAAAGAAATGATGATTTTATTGAATTAGACTATACCATCCTACTGCATGACATTGCATCTCAGGAAATGATTCCTTGGCAAATGCAAGTCCTCTGGCATCCCCAGTATGGAGTTAAAGTGAAACACAACAGCCGTCAACCCAAGCAGGCCCACCTGGAATAAGCAGCAGCACGCAGTCACCAACACCCCTGCTCATGCTGTTGACCCACCCCCCTTCCACTTGGGAACCAACCCTTCCCAAGACAGTGACATACCAGTGTCTAGCCTGCAGATTACTATTGCGCAGACTCCATAATAAACACTTCGATTCACGTCTAAAAAAAAA

>UoN.Pci.T.13188_UoN.Pci.T.13188.1

GCGCTTGCGCTCCGGGCGCGGCCTGAGGCACCATGGTGGCCCGCGCGGCTTGGCTGCTGTTAGCCGCCCCGGCCTTGCCCCTGCTCTGCCTGCAGCTACGGAGCGCGGGCTTCAACCCAGGATGGAAGGATATCGCTTTGCTGGGCGGCCGGGGTTTGCTGTTTCTTGCTATCCTGACTCTGATTATTTTGATGACCACCCCATGGCTCACATCTCAAGCTCCATCCAAAGTGTGTTTGACAGTAACCGAAGATGAGAACAAAAGAAGGCAGAAACGAGTGAGGGAAGAGCAGCAGGAGACCCTGAGCATCCAGAGCAGTGCATACCTGAAGAATGTCTTGAAACCTCGCCAGGAAGTGAAACTGAGAAAACAGGAAGAACGCTTCTATCAAATGACCGGGGAAAGCTGGAAATTACTTGCCGGCTACAAGCTTGGGGCCCTCGGTTTACCTGAAGAACCTCCGGAGACAGCCGAGGAGGTGGTTACGGTCGCCCTCCGCTGTCCTGATGGACGAGTGCTCAAACGGAGGTTTTACAAGACGTGCAGCTCACAGGTACTGTTGGACTGGATGATGAAAGTGGGGTATCACAGGTCCATTTATACCCTCTCCACTTCCTATCCCAGAAGACCTTTGGAAGTGGGAGAAGACCAGACATTAGAAGACACTGGTCTCACTACAGACACTGTGCTGAATGTGGAGGAGAAAGAGCCCTACCTCTCGCTGTGAAGTGGGGCTCGTGGGACCTGTCACAGGATGGATACGCAAGCTGTGAGCGGCATGAGGGCTCCACGTGACAATCGAGCTAAACAGTAAATGGGCCTCCTGCAGGCAAACACACTGGCCTACCTTGGTAATAAATGGATATGCTGTCAGAGTGGGGAACCTCTGGAATGAATGAGTCACTGCCTCTCGTGGTCATTCAGGGGACAAAAATCTACCTCCCAGGAACAGGTGTGACCTCACCTGTTGGAAGGCACTGCTCCTTGGGTGGTCAGCTTGTGTTGAG

>UoN.Pci.T.13440_UoN.Pci.T.13440.1

GCTTCCCTGGGACGATGCCTAGTTCCCCGTCCGGGTAAGGCGGAAGGAGGAGACGTCAGGTGGGCGTGTACTTCCGCTATCTGCGTCTTCCCCAAGATGGCTGCCCCCGTGTCAGTGCAGGTGGAGTTCGGAGGTGGGGCAGAACTCTTATTTGATGGTGTAAAGAAGCACCAGGTCACCTTGCCCGGTCAAGAGGAGCCCTGGGATATCCGAAACCTTCTCATCTGGATAAAGAAGAACTTACTGAAGGAGCGGCCAGAGTTATTCATCCAGGGAGATAGCGTGCGACCAGGGATTCTGGTACTGATCAATGATGCTGACTGGGAGCTGCAGGGTGAATTGGACTACCAGCTACAGGACCAGGACAGCATCCTCTTCATCTCCACACTGCATGGTGGTTAGAGCATCTGTCCTCCTCTGTCCTTCAGTTCCTCCAGCTCCTAGGGACCATCAGGGCAGAAAACATCCAAAACAGGGAACAAAAGAGACAGAAATCCTCCCCCTCCCCCTCCCCCTTCCCCCACAGCCAGAGCATGTGGGACACCAAGCTGCTCATGACTTTGTCTTCATGGGGAAGGACCAGGAGCAAAAGAACCAGGCAGTCCTGCTCTCCCGCCTCTTCCCTTTGCTGCATCCTCCCCACTCCCTCTCTAGCCTTAGCAGCTTTCCCTCCGGGAAGGGAAATGAGGCCAGGTACTAAAAATGTGCTGCTTACAGCTTGTGCGATAGTGGAAGAAGCTACTACCACCTCCTTCCCCAAAACAGGGAAGGGGAGGGCAGCTTTGCCTAGAAACCTACCACAGATTGCCCCATCCATAATCTGTGCCTTGAAAGGAGGTAGTGGTCCTTCTGCCCCCACTACTCTGCAGGCGACTTTGTGAGCTCTGAATCCCTGTCGTCCTGCCTCTTAAGGAGCTGTTCCGACGTTCTAAACTGTGTCACCCTGGGCAAGTCATTCATCACCCTGGGTTTCAGTCTCCTCATCTGTGAAATTGTCTTTTGACAGTTTAAGGACCCTTTCAAGCTCTAGAATTCTGTGACTGAGATTTTTTGCCTCAGTTTCCTCATCTCAGAAACCAAGGGCTCCACCTGCTTCTTCCAGCATCATTACTGAGCCTCAGCTGGGGGCCCCTATTCACTGCTGCCTCTTCCTCCTCCTTCTGCCTGGATGCCCCCAGCTGCTCCAGCCTTGCTCCTCTGACTCCCCTAGCCCTTGACCTAGGCCTGCCCCACAGTAGACAGGCCACCAGATGGACTCCCAGGCCCCCTCCCCCAGAGACAAAAAGCTGTCGTCCGTCATATAACTGTTTGAAGACACCTGGGCTCTGAGCCCCACTGGGGAGAGCTGTGGGTCATTTCATTGTGCCTCAGTTTCCTCTTTGATGCTTAGGATTTTACCATTGTGAACTTAAAAGGGTTACCAGAAAACCAGGGCACAAGAAACTGCTTTGTGACATATTTGGGACCTTCCAGCTTTTCTCCCCACAGCATACAGACCCATCTGGAAGTCAACATGGCGGTGCCTGTAACAGCCGGCATTGGGCAAATAAAGGCCTTCATGCCTGTCGTTTTTTTCTTTATTGCCTGGAGCCTCATCCTAGATACAGCTGGGCTGGGTCAGTTGTTGGTATCCCAGTGACCTCATGGCACAGCTCCTGGCTTGATGCAAATATCCCAAGGATTCTGAGAATCATCCTCTTATTTCCCTGGAGCAGGTTTAAGGGCCCAACCAGAATTGTCTGGACCAGATCTGGGGATGGGGATTCCCCAACAATGATGTTGGGGAAATTTGGGGGGACCAGGAAAGGTTGAATCCAGCTTGAGCTGGATCTCCAAGTTCACAGTAAAGCTGGGAGCTGGAACCCTTGCTCATCTGCCTCTCCCTTGTATACATAATGTTGAGTATTACACCATCTACCACAGGATTCATTTCTAGTCTATCACTTAGCCTGTCAGACTGAGTTTTCAGGAACCCTTCCTAATATATTCCCAATTCACTCAGTCTTCAGAGAAGCCCCCTCTTCATCCATCAGAAGAGATTGAGAATTCTCAGTACATGCTAGGTATCCCTGTTCCCAATCTTGGGTGTTTGTTTACCTTGTCTCTTTATATATGTAGGAAGGAGGAGGGATGTGTTACTTGTCTGAAAGTCTAGAACTATTGGCATAGCTAGGCCCCAAAGATCCTTTGTACATCCACAAGGAGAATTCAATCCCCTCAGTCCCAAAACTAGAGATGAGGAGTAAGGGATGGGGGGGGCAGGGTTTTGCCACGGACAGAGAAAATATTTTCCCCAGATACTTAGGTTTTCCAGAAAAGCTGAAAGAGGGGAGAGGATAAAAATACCTCCCACCCATTACCCACCTCCAGTCATCCTCTAAATGAGACTAATGCCAAGCTCATCTCCCTCATGTTGATCCATGTGAGGGACTTAGATCTTCCTGGCTTTGGGGTGTCGAGTGAAAGATGAGGTGTTCATTGTAAAAACTCCTGGACTGGTTGTGCTCTCCTTATCTTAGTATCAATTAAAATTGGTAATGTGTTTGGAAAAAAAAAAA

>UoN.Pci.T.15753_UoN.Pci.T.15753.1

GAATGGTGGATGGTTTTCCAATATGCTGATGATTACCTCCTCCGAAGGGATGTTCCACGGGCTATCGGGAAAGAAGGTTCTCACATCACCAAGCACGCAAGGGAACATGAATTTCTTCCCTTTAAATCCTGCTGACTCAACTCAGGGAAAGGGACAAACACCCCACTCTACCCCCTCCTCAATGTACTGAACTGCTCCTCCCTTTTTACTCCTTGATATAAAGGATGCATCTGAAAATGATACAAAACCAGAAAAACCGAAAATCTTAAAGGGAATTAACACTTATATTTGATTCCATAGCTAAGTTCCACCCTCTCAACAATCCTATCCTCACTTAGCTCCTGAAGTTCAGCAGTAATTTCAGAGATGTAAATGGCCAACCAACTAACTGACCCGTGTCTCAAAGAATAACACTTGCAATCTCAATCCAGGGCAGCCTCAACTGAATGCTGTTATGCCCTCTTTCACCTCCCCAGGTAGCCAGCCACACAGCGTGGCAGTCTGAAAAACTAATGACTAGAACTACACTGAAGTATCCATTGAGACATCCCCACGAGTTACCATCGAACAGAAGCAACAGATGAAACACTCACATTCATGGCCACACCCCGGACACGAGGCCAGCAGTTTCTCTTGGCCTTGTATTTGTGGT

>UoN.Pci.T.16730_UoN.Pci.T.16730.1

GCCTCGCTCCGTCATTCAGGCCGGGGGCAGCGGGGCGGCTGAGGCGGGAGCGGCGGCGGCGGCTACACAGGCGGCGACGCCGGCGACACCGAGCCCTCTGAGCCCAGCGATCCCGGCGACCCTGACCCCACCCCCCGGCGACGCGCGGCGGACGGAGCAGCGCCGCCACCATGATCACCTCAGCCGCTGGAATCATTTCTCTTCTGGATGAAGAAGAACCACAGCTTAAGGAGTTTGCACTACAGAAATTGAATGCAGTGGTCAATGACTTCTGGGCAGAAATTTCAGAGTCTGTAGACAAAATAGAAGTCCTGTATGAAGATGAAGGCTTTCGTAGTCGTCACTTTGCTGCCCTCGTGGCATCCAAAGTGTTTTATCACCTGGGGGCTTTTGAGGAGTCTCTGAACTATGCTCTGGGAGCCGGTGACCTCTTCAATGTCAATGACAATTCGGAATATGTGGAGACCATCATAGCCAAATGCATTGACCATTACACCAAGCAGTGTGTGGAGAATGCCGAGTTGCCCGAGGGGGAAAGGAAGCCTGTCGACCAGAGACTAGAAGGTATTGTGAACAAGATGTTTCAGAGGTGTCTGGACGATCACAAGTACAAACAGGCCATCGGCATTGCTCTGGAGACCCGCAGGCTAGACGTCTTTGAGAAGACCATATTGGAGTCGAATGATGTCCCTGGGATGCTGGCTTACAGCCTCAAACTCTGCATGTCTCTGATGCAGAATAAACAGTTCCGTAATAGAGTGCTGAGAGTTCTGGTTAAAATTTACATGAATTTGGAGAAGCCGGACTTCATCAACGTTTGCCAGTGCTTGATTTTCTTAGATGATCCTCAGGCTGTGAGTGACATCTTGGAGAAGCTGGTCAAGGAGGACAACCTCCTCATGGCCTATCAGATCTGCTTTGACCTGTATGAGAGTGCCAGCCAGCAGTTCCTGTCCTCCGTGATCCAGAACCTCCGTACTGTGGGCACCCCCATCGCTTCTGTGCCCGGTTCAACCAACACGGGTACCGTGCCCGGGGCCGAGAAGGAGAGTGAGAGTATGGAAACAGAAGAAAAGACAGGCCACACATTTGCTGGGAAGTCCCCAGAAGTGAACCCAGAGCCCAAGGACCAGATATCAAAAATGATTAAAATTTTAAGTGGTGAAATGGCAATTGAATTACATCTTCAGTTTTTAATACGAAATAATAACACAGACCTCATGATTCTAAAAAACACAAAGGATGCGGTGCGGAACTCTGTATGTCACACGGCCACAGTCATAGCCAACTCGTTCATGCACTGTGGGACGACGAGTGACCAGTTCCTTAGAGATAATTTGGAATGGCTTGCCAGAGCCACGAACTGGGCAAAGTTTACTGCAACTGCCAGTCTGGGTGTAATTCATAAGGGTCACGAGAAGGAAGCATTACAATTAATGGCAACCTATCTCCCCAAAGACACCTCTCCCGGGTCAGCCTATCAGGAAGGTGGAGGTCTCTACGCTTTGGGTCTCATTCATGCCAATCATGGTGGCGACATCATTGACTATCTGCTTAACCAGCTCAAGAATGCCAGCAATGACATTGTCCGACATGGTGGTAGTTTGGGCCTCGGATTGGCTGCCATGGGCACAGCCCGCCAGGATGTCTACGATCTGCTGAAAACAAATCTGTACCAGGATGACGCCGTGACAGGAGAAGCAGCTGGCCTTGCCCTGGGCCTGGTTATGTTAGGTTCTAAAAATGCCCAGGCTATCGAGGACATGGTTGGCTATGCCCAGGAGACCCAGCATGAGAAGATCTTACGGGGCCTTGCTGTTGGCATCGCTTTGGTAATGTACGGAAGGATGGAGGAAGCCGACGCCCTCATTGAGTCTCTGTGCCGGGATAAGGACCCAATCCTCCGTCGATCTGGAATGTACACTGTAGCCATGGCTTACTGTGGTTCAGGGAACAACAAGGCCATTCGACGCCTGCTGCACGTGGCAGTCAGTGATGTCAATGATGATGTCAGAAGGGCTGCAGTAGAATCCCTTGGGTTCATTCTATTCAGGACGCCTGAACAGTGCCCGAGCGTGGTTTCCTTGTTGTCAGAGAGTTATAACCCTCACGTTCGTTATGGAGCCGCCATGGCGCTGGGTATCTGCTGCGCCGGCACAGGAAACAAGGAAGCCATCAATCTGCTGGAACCCATGACCAACGACCCAGTGAACTACGTGCGACAAGGTGCGCTCATTGCCTCGGCTCTCATCATGATTCAGCAGACAGAGATCACTTGTCCAAAGGTCAACCAGTTCAGACAGCTGTATTCCAAAGTCATCAATGATAAGCATGATGATGTCATGGCAAAGTTTGGTGCTATCCTGGCCCAGGGCATCTTGGATGCAGGGGGCCACAACGTCACCATCTCCTTGCAGTCTCGGACTGGGCACACCCACATGCCTTCCGTGGTGGGCGTCCTGGTCTTCACCCAGTTCTGGTTCTGGTTCCCTCTGTCCCACTTCCTGTCCCTGGCTTACACTCCTACCTGTGTCATCGGCCTTAACAAGGACCTCAAGATGCCAAAAGTCCAGTACAAGTCCAACTGCAAACCGTCCACATTTGCATATCCCGCGCCCCTCGAGGTGCCGAAAGAGAAAGAGAAGGAAAAGGTTTCTACTGCTGTGCTCTCGATCACCGCCAAGGCCAAAAAGAAGGAAAAAGAGAAAGAAAAGAAAGAGGAAGAGAAAATGGAAGTGGATGAAACAGAGAAGAAGGATGAAAAAGAAAAGAAAAAGGAGCCTGAGCCGAGCTTCCAGCTACTGGATAATCCAGCCCGAGTTATGCCCGCACAGCTGAAGGTCCTCTCCATGACTGAGACCTGTCGATACCAGCCTTTTAAACCTCTCTCCATCGGAGGCATCATTATCATGAAGGACACCAGCGAGGATCTGGAAGAATTGGTGGAGCCCGTGGCTGCTCACGGCCCCAAAATCGAGGAGGAGGAGCAGGAGCCAGAGCCGCCAGAGCCCTTTGAGTACATCGACGACTGAGGCCTGGAGAATCTCATTCACCCATTTCCAGAAGATTGCCGAGTCTGATATTGGAAATAACATCCACGAGGAAGTTCCTGCCGAGACCTGCCATTCAATGCATGCATGGTTGCCTCTGTGCTAACCTTGGGAAGCCAGTCTGAGTCTTCGGTGGAAGAAAAGATGGATGGCTGTTTAGTATACTCTTACACAAACAAAACGTGGTTTTCAAATAAACATATTAAAATCTCCAAAAAAAAAAAAAA

>UoN.Pci.T.16730_UoN.Pci.T.16730.1

GCCTCGCTCCGTCATTCAGGCCGGGGGCAGCGGGGCGGCTGAGGCGGGAGCGGCGGCGGCGGCTACACAGGCGGCGACGCCGGCGACACCGAGCCCTCTGAGCCCAGCGATCCCGGCGACCCTGACCCCACCCCCCGGCGACGCGCGGCGGACGGAGCAGCGCCGCCACCATGATCACCTCAGCCGCTGGAATCATTTCTCTTCTGGATGAAGAAGAACCACAGCTTAAGGAGTTTGCACTACAGAAATTGAATGCAGTGGTCAATGACTTCTGGGCAGAAATTTCAGAGTCTGTAGACAAAATAGAAGTCCTGTATGAAGATGAAGGCTTTCGTAGTCGTCACTTTGCTGCCCTCGTGGCATCCAAAGTGTTTTATCACCTGGGGGCTTTTGAGGAGTCTCTGAACTATGCTCTGGGAGCCGGTGACCTCTTCAATGTCAATGACAATTCGGAATATGTGGAGACCATCATAGCCAAATGCATTGACCATTACACCAAGCAGTGTGTGGAGAATGCCGAGTTGCCCGAGGGGGAAAGGAAGCCTGTCGACCAGAGACTAGAAGGTATTGTGAACAAGATGTTTCAGAGGTGTCTGGACGATCACAAGTACAAACAGGCCATCGGCATTGCTCTGGAGACCCGCAGGCTAGACGTCTTTGAGAAGACCATATTGGAGTCGAATGATGTCCCTGGGATGCTGGCTTACAGCCTCAAACTCTGCATGTCTCTGATGCAGAATAAACAGTTCCGTAATAGAGTGCTGAGAGTTCTGGTTAAAATTTACATGAATTTGGAGAAGCCGGACTTCATCAACGTTTGCCAGTGCTTGATTTTCTTAGATGATCCTCAGGCTGTGAGTGACATCTTGGAGAAGCTGGTCAAGGAGGACAACCTCCTCATGGCCTATCAGATCTGCTTTGACCTGTATGAGAGTGCCAGCCAGCAGTTCCTGTCCTCCGTGATCCAGAACCTCCGTACTGTGGGCACCCCCATCGCTTCTGTGCCCGGTTCAACCAACACGGGTACCGTGCCCGGGGCCGAGAAGGAGAGTGAGAGTATGGAAACAGAAGAAAAGACAGGCCACACATTTGCTGGGAAGTCCCCAGAAGTGAACCCAGAGCCCAAGGACCAGATATCAAAAATGATTAAAATTTTAAGTGGTGAAATGGCAATTGAATTACATCTTCAGTTTTTAATACGAAATAATAACACAGACCTCATGATTCTAAAAAACACAAAGGATGCGGTGCGGAACTCTGTATGTCACACGGCCACAGTCATAGCCAACTCGTTCATGCACTGTGGGACGACGAGTGACCAGTTCCTTAGAGATAATTTGGAATGGCTTGCCAGAGCCACGAACTGGGCAAAGTTTACTGCAACTGCCAGTCTGGGTGTAATTCATAAGGGTCACGAGAAGGAAGCATTACAATTAATGGCAACCTATCTCCCCAAAGACACCTCTCCCGGGTCAGCCTATCAGGAAGGTGGAGGTCTCTACGCTTTGGGTCTCATTCATGCCAATCATGGTGGCGACATCATTGACTATCTGCTTAACCAGCTCAAGAATGCCAGCAATGACATTGTCCGACATGGTGGTAGTTTGGGCCTCGGATTGGCTGCCATGGGCACAGCCCGCCAGGATGTCTACGATCTGCTGAAAACAAATCTGTACCAGGATGACGCCGTGACAGGAGAAGCAGCTGGCCTTGCCCTGGGCCTGGTTATGTTAGGTTCTAAAAATGCCCAGGCTATCGAGGACATGGTTGGCTATGCCCAGGAGACCCAGCATGAGAAGATCTTACGGGGCCTTGCTGTTGGCATCGCTTTGGTAATGTACGGAAGGATGGAGGAAGCCGACGCCCTCATTGAGTCTCTGTGCCGGGATAAGGACCCAATCCTCCGTCGATCTGGAATGTACACTGTAGCCATGGCTTACTGTGGTTCAGGGAACAACAAGGCCATTCGACGCCTGCTGCACGTGGCAGTCAGTGATGTCAATGATGATGTCAGAAGGGCTGCAGTAGAATCCCTTGGGTTCATTCTATTCAGGACGCCTGAACAGTGCCCGAGCGTGGTTTCCTTGTTGTCAGAGAGTTATAACCCTCACGTTCGTTATGGAGCCGCCATGGCGCTGGGTATCTGCTGCGCCGGCACAGGAAACAAGGAAGCCATCAATCTGCTGGAACCCATGACCAACGACCCAGTGAACTACGTGCGACAAGGTGCGCTCATTGCCTCGGCTCTCATCATGATTCAGCAGACAGAGATCACTTGTCCAAAGGTCAACCAGTTCAGACAGCTGTATTCCAAAGTCATCAATGATAAGCATGATGATGTCATGGCAAAGTTTGGTGCTATCCTGGCCCAGGGCATCTTGGATGCAGGGGGCCACAACGTCACCATCTCCTTGCAGTCTCGGACTGGGCACACCCACATGCCTTCCGTGGTGGGCGTCCTGGTCTTCACCCAGTTCTGGTTCTGGTTCCCTCTGTCCCACTTCCTGTCCCTGGCTTACACTCCTACCTGTGTCATCGGCCTTAACAAGGACCTCAAGATGCCAAAAGTCCAGTACAAGTCCAACTGCAAACCGTCCACATTTGCATATCCCGCGCCCCTCGAGGTGCCGAAAGAGAAAGAGAAGGAAAAGGTTTCTACTGCTGTGCTCTCGATCACCGCCAAGGCCAAAAAGAAGGAAAAAGAGAAAGAAAAGAAAGAGGAAGAGAAAATGGAAGTGGATGAAACAGAGAAGAAGGATGAAAAAGAAAAGAAAAAGGAGCCTGAGCCGAGCTTCCAGCTACTGGATAATCCAGCCCGAGTTATGCCCGCACAGCTGAAGGTCCTCTCCATGACTGAGACCTGTCGATACCAGCCTTTTAAACCTCTCTCCATCGGAGGCATCATTATCATGAAGGACACCAGCGAGGATCTGGAAGAATTGGTGGAGCCCGTGGCTGCTCACGGCCCCAAAATCGAGGAGGAGGAGCAGGAGCCAGAGCCGCCAGAGCCCTTTGAGTACATCGACGACTGAGGCCTGGAGAATCTCATTCACCCATTTCCAGAAGATTGCCGAGTCTGATATTGGAAATAACATCCACGAGGAAGTTCCTGCCGAGACCTGCCATTCAATGCATGCATGGTTGCCTCTGTGCTAACCTTGGGAAGCCAGTCTGAGTCTTCGGTGGAAGAAAAGATGGATGGCTGTTTAGTATACTCTTACACAAACAAAACGTGGTTTTCAAATAAACATATTAAAATCTCCAAAAAAAAAAAAAA

>UoN.Pci.T.16949_UoN.Pci.T.16949.1

CCGGAAGCGGCGCCGAGGCCGCTTCCGGTCTCCCGCCGCTGAGGCCGAGGCTGAGGGTGTTCGGGCTGCGATGGCGGCCGCGGTGGCGCCGGGGGCGGAGCCGGGCCCGGGGGCGGGGTCGGTGCCGAGCTTGGAGAGCCAGCGGCGGCTGGTGGAGGACGGGGAGAGCGGCCGGGAGCGGCCCCTGCGGCCAGGGGAGAGCTGGTTTCTGGTGGAACAACACTGGTACCAGCAATGGGAGGCCTATGTCCGAGCTGGAGACCAGGACTCTAGCGCCTTCCCTGGGCCTGTCAACAATGCTGAGCTCTTCGAAGACCAAGAAACCTGGCGCCTGAAGGAACGGCTGGTGGAAGGTGAGGACTATGTGCTGCTGCCAGCCATGGCCTGGCATCTTCTGGTTAACTGGTATGGGCTGGAGCCCGGGCAGCCACCCATCGAGCGGAAGGTGGTAGAGCTGCCCAGCACTCACAAGGTAGAAGTATATCTGGTGGAGCTGCTCCTGTGTCAACACAATGATATGGAGATATCTCGGCCTGCCCAATTCAGCCGAGCAGACCCTGTTGAGTTGGTCCTTCATACAGCCAGGGAACAATTCTTAGTGGGGCCAGAGGAGGAGACACGCCTGTGGGTGAAGAACGCAGATGGCTCCTGTGAGAGACTAAGGAACATACATATGTCTGTGTTAGATGCCTGCCTTGGACCTGGTCAGGTTGTCATCATGGAGACTCGGAATAAAGATGGCACATGGCCTAGCTCCCGGCCTCACATCATGTGAGTGGAGATAGGGCCTGAAGAGGCTCAGGGGCAAGCCAAGGGTGGTTTGGGAGACCTAAATAGTGTCTTTTGAGGGCCTACGAAGAAAGGAATTTTGGGAGTGCTGGAAGGGACCTGAAGGTGAGGGAAAGAGCTCTGGGGATACTTAAGGGGACTTGAGAGCCAAGAAAACTATCCTTTGGGTGTTCTGTTAAACTTTAGATAAAAGGTCATAGGAGTGTCTGAGGGAACCCAAGGGTGAAAGAAGTGTTTTGGGGGGTTGAAAATGGGGATCTTAGGGGTGTTTGTAAACCCTGAGGATAATTGGGAGGTAGGAACACTTGAAGTTGACTGAAGGAGTCTTGGGGTTGTCTGGGAATTCATGAGGCAGGTGTTGACTGTCCTTGGGGCCCCTAGGAAGAGCGTATCAGATGAGGAGGACAGTTTCCGGGGCCAGCCAGGCGTCTGTGGCCTCACCAACCTGGGGAACACGTGCTTCATGAACTCAGCCCTGCAGTGCCTGAGCAATGTCCCCCCGCTTACGGAATACTTCCTCCACAACCACTACCTAAAGGAGCTGAATTTCTGCAATCCCCTCGGCATGAAGGGCGAGATTGCTGAGGCCTATGCTGACCTGGTCAAACAGGCCTGGTCCGGCCATCACCGCTCTGTGGTCCCCCGGATGTTCAAGACCAAGGTTGGCCACTTTGCCTCCCAATTCCTTGGGTACCAGCAACACGACTCCCAGGAGCTACTGTCTTTCCTGCTTGATGGGCTGCATGAGGACCTGAACCGGGTCAAGAAGAAGGAGTACGTGGAGCTTCGAGATGCTGCTGGACGGCCTGATCAGGAGGTAGCAGATGAAGCATGGCGAAACCACAAGCGTCGGAATGATTCTGTCATTGTGGACACATTCCATGGGCTCTTCAAGTCAACGCTGGTCTGCCCTGAGTGTGGAAAAGTGTCTGTAACCTTTGACCCCTTCTGCTACCTGAGTGTCCCTCTGCCTGTGAGCAAAGAGCGTGGCATGGAGGTCTTCTTCGTTTCTATGGACCCTCGGCGAAAGCCTGAGCAGCATCGTCTGGTTGTGCCAAAGGCGGGGAAGGTGCTGGACCTCTGTGTCGCACTAGCTAAACACACAGGCGTGTCTCCAGACAGGATGATGGTAGCTGATGTGTTCAGCCACCGCTTCTATAAGATCTACCAGCTGGAGGAGTCTCTGAGCTGTATCCTGGACCGGGATGACATCTTTGTGTATGAGGTGTCTGGTGGAGCTGGAAGTGGAGAGGGTACAGGAGAGGACGTAGTACTGCCTGTCTACCTTCGGGAGCGGACCCCGGCCCGGGACTATAGTGACTCCTATTATGGTCTCATGCTTTTTGGACACCCACTACTGGTGTCTGTGCCTCGTGACCGGCTCTCCTGGGATGCCCTCTACCACATTCTGCTGCATCGCCTTTCGCGGTATGTTACTCGACCTGAATCAGATGAGGAG

>UoN.Pci.T.16133_UoN.Pci.T.16133.1

TCAAAGTGACCCCGTTTAATGGGCAGAAAGAAAGTGCCCTCCCAGGATGGGAGGGCAAGATCCGCCCCCATCCACACCAAGTCTGGTGAGGATGTGACCCCCAGCCCCCTCGCCCCAGGTCAGGAATAAGAGGAGAAAGGGCAGAGCTTTAGACAAGAAGGAGGGAGGGGTAAAGTGCGTGTGCGGGAAGAATGGGAGAGGCAGAGTCAGTGAGGACCACATCAAGGTATAACACTCAGGGATCTGGCCCCGAGTCCTCAGGCAGGATCTCCAGGCTGCTGTCAGCCTGGGGAGTGGGTGAAGGACCCCCAGAAACAGAGGATGGGGCTTCAGGGGCTGGGGTGGGTGACAGAGGCAGGGGTGAGCCTGAGGGGGAAGGGCTGCCTGGTGTGGAAGCTGGGGCCAGGCTCAGGCCCAGGATCTCCTGCACGTTGTGGGGCAGCTCCTGGGGGGAGAGGCAGAGGCCAGAGTGAGGGGGGAAGGGTCCATGGCGGAGTATGGAGCCCTTCTTAGGGGAGGGCTAAGAACAGTGGATTAAGGAAAAGCTAGGAAATTGGGTGGGAAGAGATGAGGGCAAGGGCAAGGAGTGTCTAGGTCTCACCTGACAGGCTGTCAGCTGCGCATACAATGCCTGGGCCCGGGTCAGGACATCTTCCACACTCAGCTTCATAGTAAGTTCATTAATGTGTTTGAGGATCTCATTGGAGCCAAAGCCAGAGAGCATGAGCGCGTCCCTCTCCATGTCAAGGATGGCGCAGGCCACCAACAGGTGCAGGTTGGGGCCTGGGAGCCCAGTCCACAGCACCTCCCACAAACGGAGAACATCAGGGAAGGAGAATTCACGCTTGAACCAGATGAGCAGCCAGCGGAAACAGAAGCAGAGAGAGCCAGAGTCCTGGGAGTCGAGGAAATCACAGAGTGGAGGATCTAGAACCCGCAACAGCAGCAGAAGCTGGCCAAGCTGACGCTTCATCGTCTCCTGACTCTCCTCAAAGTTTCCGTGCACAAGCTCCATGAAGCCACAGAAGCACCAAAAGGCATCGACCTCATTCTGAGTGACAAAGAGGATTGGAGAGAGAAGATCGCTCATGCCCTGGACGTAACCAAGGTCAAAGTGATACATGCAGTAGGTCAGAAGGATGTCATTCAGCAAGCCCAGGCCCGGATTCCCAGGCCCCTCGTAGAACTTGTTGTTGCGGTCAGTACGGCTCACATCTCGCTCAATGAGGCTGCGATATCCATGGAGCAATGAGTTCCTCCTCTCCTGCTCGGGGCTCACTGACTTCCATTGCAGCTTCATACGAAAATACTCATCCCTACACCCACCAGGAGAGCGGGGGTGGGGGTGGGGGGAGAAAGAATGGGGCAGAAGACAGGTGACCTAGCACTTCAGTTTTTTTGTAACACTGGGTTAAACAGTCCAGTAACGTGGTCCTTAAGTGGGACCTGACAGGCACCCAAAGTCACTGCCTATGGTGTGAAAGTTTCATTCCATCTAACTTCAGGTTCTGTTTCATCCTTGTTAGTTTTGTCATTTTGATTCAGTACAAATATTGCATAGTGGTATGTGACCACTGGATGCACAGAGTCTGGGTTTCTCTCTATTACATTTCAATGCCAAATTCAGGAGTTGCAGACTTGCCACTGATGAGAGTGAAGCAAGTAGGTGAGTAAAACAATCTCCCTGATGTGAAAGGAAGCAGCAGATTCCCTCATAGGGGTTCAGATGCCGAAATCCCCTCTGACTCCTGGAAGGGCTCCTGACAGTGTCTCTTGGCAGCCCCTGCCAGTTGCTTCTTGGCATAGTTGCTGGCAGGCCAGCTCCCTGGCCTCTTAGAGAGGTGTGCCTGGACAAGTTGCTGAGCCCAGAAGGAGCTGGGTGTTCAAAAGAGGAACTGACGACTTCATTTGGGAACCAAAAACCTTAGTAAAAGAATTAAGTAGAGGACTTCGAGCCATCTATTTTCCCTCAAAAGTATGCATCTTTGCCAGCAGTGTCTTGTTGGATTATATTACTGAGGAATGAACTGAGATGATTATGTGGAAGTGTTATTTAATGGCATATTGTACTAGAAATTTGAAGACCTGCAGTAGATTTTAAATCCCAACTCTTGTTATAACATTTTTAAGAGATTGTGAAATTGTCAAAATGCACATTAATCAAGTTTTAATATACTGTATGATGGGTGGATGAGACTGTCCATTGTACCTTATTTCGTTATTTGAATTCTCAGGCATGGTTTTGGCAGTGCAAGAACCCTGTAACATTAACAAATTCAATAAAATGGAAATGTATATGGAAAAAAAAAAAA

>UoN.Pci.T.16285_UoN.Pci.T.16285.1

GTTCGGAAGGTTGCCGCAGGCTCCTCCCCCGTGCGGGGCTACAGCTTCCGGTTCTGAGCCCGGAGCCCCATAATCCTCTGTTCCTTCCATCTCCAACCGGGACCCGTACAGATCTGTGGCGAAGATGGCGGTGGCCGTGGTTGGAATCCTGGGCGGCCGGCTCCGGCCCCGAGCTGGCCAGCTGCCCACCATCCAGACTGTTCGCCATGGCTCCAAGGCTGTTACCCGACATTTTCGAGTTATGCACTTTGAGCGACAGAAACTGATGGCACTGACTCAGTATATCCCCCCAAAACCTGTCATTCCCCCATGTTGTCTGTCCCCCCCCCCCAAGCCACCCTCGGAGGAGTTGGGCTTGAATCGATTGCTCCGGAGGGAGGTGACAGAAGTTTTCAGAAAAAACCGAATGATTGCTGTATGTCAGAATGTGGCTCTGAGTGCTGAGGACAAGCTCCTTGTCAGGCACCAGCTTCGGAAACACAACATCTTTGTGAAAGTCTTTCCCAATTCAATCCTGAAGCCATTCCTGGCGGAATCCAAGTACCAAAACCTGCTACCTCTCTTCATTGGGCATAACATGTTGCTGGTGAGCCCAGAACCCAAGGCCAAGGAGATGTTTCGGGTCTTGAAGAGCATGCCTGTCCTACCACTACTTGGTGGCTGCATTGATGACACCATCCTCAGCTATCAAGGTTTCCTGAACTACTCCAAACTGCCCTCACAGTCTTTGATGCAAGGGGAGCTGGTAGGGGGCCTGACCATCCTGCCATCTCAGACTCACTCCCTCCTACAGCACCAGCCCCTTCAGTTGACTGCCTTGTTGGATCAGTACATCAGACAGCAGCACATAGAGGACAGCGAAGCGCCCAGCACTGGACAGCCGGCTTCCGCTGACCCTGTAACAGACACTTAGCTGGGTGGGGCTCCGCGTCCATAAATGTCCTTTGACCCAAAAAAAAAA

>UoN.Pci.T.16285_UoN.Pci.T.16285.2

GTTCGGAAGGTTGCCGCAGGCTCCTCCCCCGTGCGGGGCTACAGCTTCCGGTTCTGAGCCCGGAGCCCCATAATCCTCTGTTCCTTCCATCTCCAACCGGGACCCGTACAGATCTGTGGCGAAGATGGCGGTGGCCGTGGTTGGAATCCTGGGCGGCCGGCTCCGGCCCCGAGCTGGCCAGCTGCCCACCATCCAGACTGTTCGCCATGGCTCCAAGGCTGTTACCCGACATTTTCGAGTTATGCACTTTGAGCGACAGAAACTGATGGCACTGACTCAGTATATCCCCCCAAAACCTGTCATTCCCCCATGTTGTCTGTCCCCCCCCCCCAAGCCACCCTCGGAGGAGTTGGGCTTGAATCGATTGCTCCGGAGGGAGGTGACAGAAGTTTTCAGAAAAAACCGAATGATTGCTGTATGTCAGAATGTGGCTCTGAGTGCTGAGGACAAGCTCCTTGTCAGGCACCAGCTTCGGAAACACAACATCTTTGTGAAAGTCTTTCCCAATTCAATCCTGAAGCCATTCCTGGCGGAATCCAAGTACCAAAACCTGCTACCTCTCTTCATTGGGCATAACATGTTGCTGGTGAGCCCAGAACCCAAGGCCAAGGAGATGTTTCGGGTCTTGAAGAGCATGCCTGTCCTACCACTACTTGGTGGCTGCATTGATGACACCATCCTCAGCTATCAAGGTTTCCTGAACTACTCCAAACTGCCCTCACAGTCTTTGATGCAAGGGGAGCTGGTAGGGGGCCTGACCATCCTGCCATCTCAGACTCACTCCCTCCTACAGCACCAGCCCCTTCAGTTGACTGCCTTGTTGGATCAGTACATCAGACAGCAGCACATAGAGGACAGCGAAGCGCCCAGCACTGGACAGCCGGCTTCCGCTGACCCTGTAACAGACACTTAGCTGGGTGGGGCTCCGCGTCCATAAATGTCCTTTGACCCAAAAAAAAAA

>UoN.Pci.T.16285_UoN.Pci.T.16285.3

GTTCGGAAGGTTGCCGCAGGCTCCTCCCCCGTGCGGGGCTACAGCTTCCGGTTCTGAGCCCGGAGCCCCATAATCCTCTGTTCCTTCCATCTCCAACCGGGACCCGTACAGATCTGTGGCGAAGATGGCGGTGGCCGTGGTTGGAATCCTGGGCGGCCGGCTCCGGCCCCGAGCTGGCCAGCTGCCCACCATCCAGACTGTTCGCCATGGCTCCAAGGCTGTTACCCGACATTTTCGAGTTATGCACTTTGAGCGACAGAAACTGATGGCACTGACTCAGTATATCCCCCCAAAACCTGTCATTCCCCCATGTTGTCTGTCCCCCCCCCCCAAGCCACCCTCGGAGGAGTTGGGCTTGAATCGATTGCTCCGGAGGGAGGTGACAGAAGTTTTCAGAAAAAACCGAATGATTGCTGTATGTCAGAATGTGGCTCTGAGTGCTGAGGACAAGCTCCTTGTCAGGCACCAGCTTCGGAAACACAACATCTTTGTGAAAGTCTTTCCCAATTCAATCCTGAAGCCATTCCTGGCGGAATCCAAGTACCAAAACCTGCTACCTCTCTTCATTGGGCATAACATGTTGCTGGTGAGCCCAGAACCCAAGGCCAAGGAGATGTTTCGGGTCTTGAAGAGCATGCCTGTCCTACCACTACTTGGTGGCTGCATTGATGACACCATCCTCAGCTATCAAGGTTTCCTGAACTACTCCAAACTGCCCTCACAGTCTTTGATGCAAGGGGAGCTGGTAGGGGGCCTGACCATCCTGCCATCTCAGACTCACTCCCTCCTACAGCACCAGCCCCTTCAGTTGACTGCCTTGTTGGATCAGTACATCAGACAGCAGCACATAGAGGACAGCGAAGCGCCCAGCACTGGACAGCCGGCTTCCGCTGACCCTGTAACAGACACTTAGCTGGGTGGGGCTCCGCGTCCATAAATGTCCTTTGACCCAAAAAAAAAA

>UoN.Pci.T.17178_UoN.Pci.T.17178.1

AGTGGATGTACCATAGCCTGAGACGACGGCGGCGGCGGGAGCTGGGTGACGTTGGCCGGGTTCCCGGGCCGGTGGAGGCGGTGCGCGCTGCGGCGATCCAGTGTAGTTGGCTTCCCGCCCCCGCCCCCAGCCCCTATCCCAAGGTCCCGCGGCTTTCCGCGTGCGCGCGCGCGTGCGCGGTACCTAGCCTATCCGTGCCCGGCGCCGGCTCGCGCTGCTAGAAGCTGCTTCCTGCTCTAGGCGACCACTCTTCTTCCTCCCGTAGTTCTCCCCGCTCCCCCGTCCAGCAACCGCCGGGATGCAGTGGACAAGGAGCTCGCGTGCCCTGCGAATGGCCGTGGGGCTGGCCATCGTGGCGGTGCTGGTGCAGGGCATCCGCATCTGGCTGGACTCCAAGAGCTTCGTCTTCCAGCGCGAGGAGATCGCGCAGCTGGCGCGCCAGTACGCAGGCCTTGATCATGAGCTGGCTTTCTCTCGCCTCATTGTGGAGCTCCGGCGGCTGCACCCTGGCCACATCCTGGCAGATGAGGACCTACAGTGGATTTTTGTGAACGCCGGCGGGTGGATGGGCTCCATGTGCATCTTGCATGCTTCACTCACCGAGTACGTGCTGCTCTTCGGCACAGCCATCGACACTGGGGGCCACTCTGGACGATACTGGGCTGAAATCTCAGACACAGTCATCTCTGGGACCTTCCGCCAGTGGAGAGAGGGGACAACCAAGAGTGAAGTTTACTACCCAGGAGACACCATTGTTCACGGGCCTGGGGAGGCCACAGCAGTGCAGTGGGGGGCTGGCACGTGGATGGTGGAGTACGGCCGGGGCTTCATCCCTTCCACCTTGGGCTTTGCACTGGCCGACACATTCTTCAGCACCCAGGACTTTCTCACACTCTTCTACACTGTCCGGGTCTATGCCAGGGCACTGTATCTGGAGCTCACCACCTACCTCTCCAGCCAGGGACATTGATCTCCCCCTCCCATTGACACCCCTTTCCCTGCCCAGAACTGATGAGGGTACCTGAGTCAGTGTGAGATTGAGATGGTCAGACTCTCATGGACGCAGAGACAGGCCAGACCCTCAGCGGGTTTGTATTCAGGCACACACTTAAACACAGGGATACAGACACAGTATAGGGGGTTTGGGTCTGCAGTGAACCTAAATCTACTTCCTGGGCAGCAGTCTGGGGGAGTAGTGGAGGTGGTGTTCTTGAGTGCCCTAGCCCCCAGTTTGGAACTGTGATACCTCCCACACTCACCCTCACCAGACGCCTTATATGTCTAGCTCCCTGTCCTGGCTATGCAAAGGGGACCAGTGGCCTGTTCTGTACCCCCTACTTTTTCCCCCTCCCCCAATATCTCCTCTCCTCCTGCCTTCCAGGTAGGTGCTGCCAATCATGTGATTTTCCTCCCCTTCCATTGTGGACTCCCCCAAACCCCTTCACCTTTCTTTTCAGAGCCCTTGAGGGGTTGGGACCCCAAAGTGCTAACTAACAAAAACTTCATCTGTCTCCCAGGCTGTGTGACTGACTGGGGGGAAGGCAGAGAAGGGGCTGAACCTTTTCATTAAAGAGAGTTCTGACTGAATGTTCCACAGGACGAACATCTCCAGGGCTCTATGTTAAAGGTATATCAATGAGCAGGTCTCTCCTGGGTGCACCCCATCCTCTTTGCGCCCAGTATGGAGACAGAAGGGAGCTCACAGGTAGATTAAGAACCTACAACCCACCAGGACAACCAAGGACTGATTAATTGATAGACTTCAGAGTCCTAACTACAGGATGCATAGAAGTTCAGAGAACCCTAGAGCTCTAAGGGACCTTGAAAATTCAGTTCATCTGACCTCTCTTTGTCCATGGGGAGACCAAAGCCAGAGAACTGGTGACTTGCCCATTTTAATATATATGCACCAAACGGTTTAAGTACACACATGGTTCAGGCTCAAAGCAGCCTAGAAAGGATAGTTCATTGTGAACTTGGGAAATCAGGGTGGGGCTCCTGGAGCATGTGGGACTGATAGGGAAGGCTGGAGGAACTCTAGCCACCTCCCCACCCCTCCCAAAGCATCTGGTGCCATATGTCCCTTCTCAGATGTGATTTACAGGTGGGAAATGGAGTGGCAAAGTCCATTTCAGATGATATTAATGCCAAACATTTTATCGAGTGCTTAAAGACTTGCAAAATGCTTTCCTCAATATTCCTGTCAGGTAGGTAGCATAAGTACAAAGTGTCCCAAAAATCTTAGCGTGTTTTTAAGCTATTGTAGTTTAAAAGCACATGCAGATTTTTCGGACCCCTTGTATTATCTCCATTTTTCAGGTGAAACAGGTTCAGACATGAAATGACTTTTATCTTTGGGCACCCAGCTAGTTAAGTATCTACCACTTTCCTGAGGTCCCAATCCAGGTCTTCTGACTCAGAGTCTGGTGCTGTGCTTGAATCCTCCCACTGAGCAACAAGGCTTCTCTTTGGCTCTCCCCACCCTATTCCTTGGGACAATTGGGCTTCCCTCTCAATCATTAGGAAATAAGGAATCCTAGAGTTTCTCTCCCTTTGGACCTGGTCTCCCTGGGCCACCACTTCAGGATGGTGGAGTGTAGTCTTTCCTCCAAGATTCCTAGGGATTCAAAGGTCTTTGGCTTCAGCTGTTATCTGAAGGAGGGTAGTATCAGATGCCCCCTCAGATCCTTCCTACTGCCCTTAACAAAGCAGCTTCTCCCCAGAATCCCTCTGTGTTGCTGTCTGTCTCAATAAATATTTCTTGAATATCAAAAAAAAA

>UoN.Pci.T.17469_UoN.Pci.T.17469.1

TTTTTTTTTTCACTGAGAAGGAAATTTATTTGATAATTCAAGGTCTATGTAAACAATTCCTTTACCCACAGAAAATGTAATCTAATCATTAAGCAGAAGAAATAACAAAGCATGAAAGAAGAAAATGTACAATCTGTTCTCCTGGAAAAAACCGGATTTCTCAACTGCTTTCCAAATGCTCAGATACCTTCAAGATTACAATTGTGTAATTCATTTGAAATGTACATTAGGAGTTGCTGGGATGAGGGAAAATCTTCCCAAGGCTATTAAAATTCAGAAGCAGAGGTGAGGTGGTTATTTGGTCAGGGTGTTCTCTTCAAATCCCAGCTCTGGGTGGGGGATAAGGGCTTACAGTTTAAATTTTAACCTGTGCTTCTGGGATTAGTCAGCCTCCCTAGCCCCATTCTAGCCTCTTTACTAAAGTAATTAAGCCATCACTGCTTTCATTCCTGAGCCTAAGAGTCAGATGTACTTATTATAAGCACACTATATTTTGGGGGAATTTTTTCCTTTTCTGAAATGGACAAAGCCAATCCCAGTCAACCAAGCAATTAGTGAAGTAACATCTCCATGCTCAGTTGTACCAAACAGAGGTGTAAACACCGCCACAAATCACACGGAAACTGAATTTAAAGTCTGACGCCACTTAAATCATTTCAAGCAATCCAATAAGGACATTCTGTTTTCCTTTGGCCTCTCCACAAGTATGAAAAGTGAAGAGGAAAATAGACCAAATCACTTGACCCTTAGGTTGAAGCTCCAAAAGTCTTCATCCCTTCTCCACTTACCACTTTTGAATCTCCTACTTAACAGGGGCTTATTATTTTTGGGGTGGCTAACCTGCATCCTTCATTGCTTAAGATTCTGACCTGACATTTTAGTAAATGTGATAGAGTTCTAGCAGCAGGCCCTAGCACCTTGCCTTGGTCAGAGTATCTGCCCAGATGGGCATCTTCTAGTTCTTGGGGAAAGCCACTATTTCTAAAATACTAGCAAATCCTGAGCAGAACCTGTCCCACCAGCAATACACACAGACCAGTTGGTAAAGCAGCTTGAATCTGTGATCAAGATGGTGGACAAATGAAAGGAGGAGAAGACTTTAAAGATCCAGAATTTAAATTTCAGTAAGACTATTTCCTCTTCCCCAAAAGGCAACTGAAGGCTATAATGAATGGGAGCACTTGAAAACACTTTATCTCTAGTGCTGGATGGGAATCGGGATGAACAGCATAAATCTTTGTTCAAATGATGTCTTCCATTATCCAGAAGGCTTCTGTATTTTGTGTTTAGCAAACCATTCGTCACTTTTGTCTGCTGCCATTGCATCATCCAACAATTTGCTCCCATTTGGATCCATCCTGGAAAGTTCTCGAAATGCTTCATCTCCCACGAAACAAATCTCATGTCCATCCGGGTCTGCCAGAATAACCACTTGGACCGCTGCTTTCCCAGGTGTGTCCAGACTAACCAGGGGGGTCAGAATCTTCTGGTTCTCCTTTTTCATCAATGCTTCTATGTCTGGTAACTCTTTCTTTGGGCAGGAGAAAGCAATCCTCCCAAAGGCTGTGCCATGATCCACTGCTCCCTCGATGCCCTGCAATTCCAGCTTGCACTGGCTCTCTGCATAGCCCAGCACAGCCCTCTGCTGCTCTTCATCCTTCTCATAAACTGTCATCCCCAGCAGGTCTGACCAGTAGTGGAGGGACGTGCACAGGTCAGACACGGCCAAGCTCACTTTCAGCACTGGGTCTGATGGGGGCTGACTGTGGTTCTTTAAATAGAACTTATATCCTCCTGGGGCTTCAGTTTCGAAAACTCCCTCAGCAACCTCATGGAGCGGCCACTCCAACTTCTTGGCGTTAGTGACAGCCTGGCTGGAGACCAGTGTGAGGCCCAGGAAGTCATTGCCAAGCCGGTAGTCACCAATGCCATAATTGTAGGTCAGTTCTGCGACAAAGTGATTGTCTTCAGACCCATAGCCCACCATGGTTTTACTCCACTTCCCGTCATAAGGCCCATTACAGGAAGCCTTGCAGCCTTCCTCAAATTCCTCATGCCGTAGGATCTGTAAGGAAATCAATGTTAGCTGCCAGGCTGTGTGTGCTCCATATGGCCGAAGGCCCCCCGTACACACTGCCCACATTACCACTCACCAGGAGACTTGCTCCACGGTGGGTAATCCAAACTTTCCTGCTTGGACCACTAGTGGCCCTTTCTTGGTTGGATCTTTATCTGGGCAGGCAGTGGCCGAGGGGCAGGGGAAATACTGCAGGTTCATGGACACCCAGAGTTGAGAGGGAGAAAAGTGGGTGGGGATCATTCTCTGGATAAGTATGGGGGGGGGAGGGAGAGAGG

>UoN.Pci.T.17469_UoN.Pci.T.17469.2

TTTTTTTTTCACTGAGAAGGAAATTTATTTGATAATTCAAGGTCTATGTAAACAATTCCTTTACCCACAGAAAATGTAATCTAATCATTAAGCAGAAGAAATAACAAAGCATGAAAGAAGAAAATGTACAATCTGTTCTCCTGGAAAAAACCGGATTTCTCAACTGCTTTCCAAATGCTCAGATACCTTCAAGATTACAATTGTGTAATTCATTTGAAATGTACATTAGGAGTTGCTGGGATGAGGGAAAATCTTCCCAAGGCTATTAAAATTCAGAAGCAGAGGTGAGGTGGTTATTTGGTCAGGGTGTTCTCTTCAAATCCCAGCTCTGGGTGGGGGATAAGGGCTTACAGTTTAAATTTTAACCTGTGCTTCTGGGATTAGTCAGCCTCCCTAGCCCCATTCTAGCCTCTTTACTAAAGTAATTAAGCCATCACTGCTTTCATTCCTGAGCCTAAGAGTCAGATGTACTTATTATAAGCACACTATATTTTGGGGGAATTTTTTCCTTTTCTGAAATGGACAAAGCCAATCCCAGTCAACCAAGCAATTAGTGAAGTAACATCTCCATGCTCAGTTGTACCAAACAGAGGTGTAAACACCGCCACAAATCACACGGAAACTGAATTTAAAGTCTGACGCCACTTAAATCATTTCAAGCAATCCAATAAGGACATTCTGTTTTCCTTTGGCCTCTCCACAAGTATGAAAAGTGAAGAGGAAAATAGACCAAATCACTTGACCCTTAGGTTGAAGCTCCAAAAGTCTTCATCCCTTCTCCACTTACCACTTTTGAATCTCCTACTTAACAGGGGCTTATTATTTTTGGGGTGGCTAACCTGCATCCTTCATTGCTTAAGATTCTGACCTGACATTTTAGTAAATGTGATAGAGTTCTAGCAGCAGGCCCTAGCACCTTGCCTTGGTCAGAGTATCTGCCCAGATGGGCATCTTCTAGTTCTTGGGGAAAGCCACTATTTCTAAAATACTAGCAAATCCTGAGCAGAACCTGTCCCACCAGCAATACACACAGACCAGTTGGTAAAGCAGCTTGAATCTGTGATCAAGATGGTGGACAAATGAAAGGAGGAGAAGACTTTAAAGATCCAGAATTTAAATTTCAGTAAGACTATTTCCTCTTCCCCAAAAGGCAACTGAAGGCTATAATGAATGGGAGCACTTGAAAACACTTTATCTCTAGTGCTGGATGGGAATCGGGATGAACAGCATAAATCTTTGTTCAAATGATGTCTTCCATTATCCAGAAGGCTTCTGTATTTTGTGTTTAGCAAACCATTCGTCACTTTTGTCTGCTGCCATTGCATCATCCAACAATTTGCTCCCATTTGGATCCATCCTGGAAAGTTCTCGAAATGCTTCATCTCCCACGAAACAAATCTCATGTCCATCCGGGTCTGCCAGAATAACCACTTGGACCGCTGCTTTCCCAGGTGTGTCCAGACTAACCAGGGGGGTCAGAATCTTCTGGTTCTCCTTTTTCATCAATGCTTCTATGTCTGGTAACTCTTTCTTTGGGCAGGAGAAAGCAATCCTCCCAAAGGCTGTGCCATGATCCACTGCTCCCTCGATGCCCTGCAATTCCAGCTTGCACTGGCTCTCTGCATAGCCCAGCACAGCCCTCTGCTGCTCTTCATCCTTCTCATAAACTGTCATCCCCAGCAGGTCTGACCAGTAGTGGAGGGACGTGCACAGGTCAGACACGGCCAAGCTCACTTTCAGCACTGGGTCTGATGGGGGCTGACTGTGGTTCTTTAAATAGAACTTATATCCTCCTGGGGCTTCAGTTTCGAAAACTCCCTCAGCAACCTCATGGAGCGGCCACTCCAACTTCTTGGCGTTAGTGACAGCCTGGCTGGAGACCAGTGTGAGGCCCAGGAAGTCATTGCCAAGCCGGTAGTCACCAATGCCATAATTGTAGGTCAGTTCTGCGACAAAGTGATTGTCTTCAGACCCATAGCCCACCATGGTTTTACTCCACTTCCCGTCATAAGGCCCATTACAGGAAGCCTTGCAGCCTTCCTCAAATTCCTCATGCCGTAGGATCTGTAAGGAAATCAATGTTAGCTGCCAGGCTGTGTGTGCTCCATATGGCCGAAGGCCCCCCGTACACACTGCCCACATTACCACTCACCAGGAGACTTGCTCCACGGTGGGTAATCCAAACTTTCCTGCTTGGACCACTAGTGGCCCTTTCTTGGTTGGATCTTTATCTGGGCAGGCAGTGGCCGAGGGGCAGGGGAAATACTGCAGGTTCATGGACACCCAGAGTTGAGAGGGAGAAAAGTGGGTGGGGATCATTCTCTGGATAAGTATGGGGGGGGGAGGGAGAGAGG

>UoN.Pci.T.17572_UoN.Pci.T.17572.1

CTCCCTCCCTCCCCCATCCAGAGGGGATGTGGGCAGTCACATGAAACATTTAGAACAACAACCGGGCAAGAGCCATCCTTCACCTACCCTGCTAATGGACATTTAAGGCAATTTAGTCAGGACCACAACAATCCATCCCATTACACAAATACTGGATCAAACAGTATCTTGACAATAAAACACCATGACCAGAAGACACCAACGCACACATCACTCAGGCAGTCAGGAAGAGAAGTCACTGCAAGGGACTGGACAGAAGAGACACAAAGCACACGCTACGATGAGGGCGGCAGAGGACACCAAACAACGTCCCCGGATGACATCTCTGGCACGCCCCTACCTAGGGGTCTTCACCCTTATGATCCTCCTTTTATTTATTTCAAATGAACATCTGACACTTTTCAAAAGGAGGCAGAAACACATTAAATGAAAGAGAAATACTGATCTCTACCTGGCACTGTGAAGGGGGACTACAGATTTGCTGGGAAAACATGGGGCAGTAGGAAAACTACACTGAGCATTAGGATTCTGTGTCAGTTCTTTCTTCTGGTGCTGCAACCTCAGATGACTTATTAAAAGTAAGCTGGTGTCAGCTCAGTCTTCCCTCCTGTGGTTACCACCTGGATTAGGCTTCCGGAGCTTGGTCTGTTTATTCCTGTGATCCAGTTGTGAAAGGTTTCTACAAGGAATTCTAATGGGGTCCAGGCACCAAAGTCTGCATCAGGCATTGATTTCCTGTTCATTGGCGTATCCAGGGTAACCGGAAGAACGGCGATGGCAGCGGAACCCGGCGGCATGCCACTGTTGTCCCCGGCCAGGCTCTGACACAGCTGATGGACTGCTCCCTTGGCCATGCCGTAGCCGATCATTCCTGGCGTTCCATCTAAAGCAGCCTTAGCCCCAGCCAAGGTCAGCAGGCCTCCATCCTTAAGGTGTTTGGTTGCAAGGTGACTAGAAATGGTTGAGGTCCACATGCTCTGTTTCCACATCAAGTCACAGTTTTTATAGAGAGATTTAGCTTTGGCACTGCCTCCTGCCCATCCTCCAGCCACACAGAGAATGGCATCCACCTTCTTATCGCCCAAAAGCTTCCCAACATCAGCAGTCACCTGGTCAGCTTGCTCAGTGAAGGAGTCAGTCATTTTAACAACCACACTGGCGTTGGCTTCTTCATTTTCTACCACGTCAATACTGGCAACCCACCAGTCTTTGGCCCGGAAAGCGTGCACGCATTTGGTGCCCAGTGCGCCCCTGCCTCCGTACACCAGCACCCGGCGCGCGTCCCCAGCCGCGGCTGCCATCATCCCCGTCCGCCCCCGCCGCTCCTCCGGCCGGCCCCGCCTCCACCCCGCACGACACGTGCTAAGCGAACAAGACAGACGGGAGGTGCGCTCCGCTCTGCGCGTCGGGAAACGCTGCTGCCGCCTAGTCTGGCTGACCGCGGGGGGTGCGCCGCGTCGCACCCAGGCCCAGGGCAGAAAGGGGCAGCACGGGCTCACGCCTCCGCCCAAGGGGGGGGAGACGCCGTGCACACAACTAGGCACAAGGGGTATGTGCAAGTTACCGTGACGATGATCCGCGGCGGGGGGGGGGGAGCGCTACA

>UoN.Pci.T.17761_UoN.Pci.T.17761.1

GTGCGGAGCCGGAATAGCCAGGCAGCGGGAGGAGCCTGGGACTGAGGGGGAGGGGCTTCCAGTGTTTGGGGTCGAGCAGGCTGACTGGAGAGCGACCCCCGCCAGGAATATACTTGGCCAGAAGAGACCTTACTGTTCTGCTGAGTTTGAGCCAGATCTATCGGAACTATAAGAATTTGCTGTGTGCCTGGAGCTGACCCTGCCTGGGAAAAAGTCATAGTTAGATCCTCTTCTTCTTGCTGTGGCCTGTAGTATCTAGCCCTGAATGGCCAAGTGGGAGATCTTGCAGGGGCTAGACCGGCCTTTCCAAGAGCGGCTCCATGAACTTTATTCAAACAGTCTCTTGCCTATGGATGTACGGAAACACCTAGCTTCTTGGATTGAGGACCAAAACTGGTACCAAGCAGTCATGAGCCCTGATACATCTCATGCTTACTTGCTCTTCCATCATCTCTTGGACCAACTGGGCTCCCAATCTAATGGTTATAGTCAGGATTCTGACCTGCTACTACAGCATAACTTAAGGAAGTTCTGCCGGGATATTCAGGCTCTCTTCCAGGATAATCCCATGCAGCTGGCCGGGCTAATTTATAATCTGCTGCTAGAGGAAAGAAATATCCTGAACCAGGCTCAAAGGACCCAGCAGGACCATGTAGACACAAGCCCTAAGGTATCCAAGGAGACTAGACAGCAACAGGAGATTGAGTCTCGGATCCAGGAATTGAAGGATATGATACAGAAACTGGTCAAAGATGTTTACCAACTGAGTGACCTACAGGATGTTTTCTCTTTTCGGTATAAGACACAGACCCCTCCAGTGAAGACATCTTCCATGGACTCTAATCAGGCCAAGCAACAACAGCTCCTTCAGGAAACACTCAATGAATTGGACCGAAAAAGGAAGGAGGTGCTGGATACCTCCAGAGCACTACATGGCCGATGTGCCACTCTGATTGAGCTCCTGCTGCAGGAGCTAGAGAAGTGGAAAGATCAGCAGCGAAAGGCCTGCATTGGGGCCCCTCTGGACCTCCACTTGGATCAGTTAGAGACTTGGTTCACAGCTGGAGCAAAGATATTGTTCCAGCTAAAGCAACAACTGAAGGAACTTGAGGATTTGACCCACTTCGTGAGCTATGAGAATGATCCCCTGAAAACTAAGCTGCCTTTACAGGGGGTCACAGAATTGCTGCAGCTCCTGCTCCACAGAGCCTTTATAGTGGAATCTCAACCTCAGATGCTCCTGCCTGTCAAACGACCTCTTGTCCTTAAGACTGGGAATAAGTTTTCTGTTCAAACACGGCTCTTGGTGAGGCTGCAGGATGCGAATGAGCCCCTGACTGTGCACGTCACCATTGACAAGAATTCTCCCCAGATGAAAGGGTAAGAGCCAGGGGACTCATGGTGGAATGGGATATGACCAGGACAAAAGAGAATCATTTTGTCTCCTATCCCTGAGACTCCAGAGGGGGGCTGGGGGGCCAGATCCTGGCTACTTATCAGATTTCCTGTATGTCCCCTCCCTCGTGGAAGTTGGCTTCAGAAATGGGAACCCAGAGGCAAGTTCAACTGTGTCTTTGATGCTGTCTGATCTTCCAATTGAGGTTATCATGAGGAAAGCATTTTGTAAACCTGAAAAAAGGACCATATAAATGCCAGTTATTGATGCTATTGGTTTTATTATTGTTTCAATTGGAGCAGCTTTCGAATGTTCAACATCCTGACCTCAGAACAGAAAACTTTGACCCCAGAAAAGGGAAGGAGCCAAGGCTTGATCTGGGACTTCCGCTGCCTGGTATGTAGAGTCAGAACCCTAGAGTTAGGTAATCTCTTTAACATGAGTGAATTGGAGAAATCTGGTGACACAGCAGCATTGCAAGAGGAGGCGGCGTGGTGTAGTGGCCAGAGTGCTGGACAAAGAACCTGAGACACTTGGATTTGAATCCCACCTCAGACAATTACTAGCCGGATGACCCTGGACAAGTCACTTAACTTTTCTGCAACTGTTTCCTTATCTGTAAAATGGGATAATAATACCTACCTGTCAGGTTTGCTGTGAAAATCAAATGAAATAATGTCTATGAAATTCTTTGCAAACCATAAAATGCTACACCCCGTTCTAACCCCTTCCCATCCTCTTCCCTTTCTGTTCACAGACACTGAAGGAGCAGAAGACAAGCAGCTCTGGGAAGGGCTGCAGCGAGGGACTGCTCATTGTGACGGAGGAACTTCACATCATTAACTTCACTGTCAAATACTCCTACCAGGGTCTGAATCTAGAGTTGAAGACCAATAGTCTTCCTGTGGTGATCATCTCCAACATGAACCAGCTTTCCAGTGCTTGGGCCTCCATACTCTGGTTTAATCTGCTCAGCTCCGATCCCCAGGACCAGCAGTTCTTTTCCAGTCCCCCCAAGGCCCCATGGACAGTGTTGGGCCTAGCCCTCAGCTGGCAGTTCTCTTCCTGTACTGGCCGAAGTCTTGACCAAGAGCAGCTGAACATGTTGAAGAAAAAGCTGTTTGTTCCCAGAACTGAACACAGCATTCTATCTGTCCCTCCATTTGAAGCCCCTGTGTCCTTCTGTCCTTCAGAGCAAGAGCACAAGAAAGAATCACAATTGTTGTTGTCCTGGGCTGCCTTCTCCAAGCAGGACAGCCCTCCTGGGAAGATGCCTTTCTGGACATGGTTGGACAAGATCCTGGACCTGGTGCACAGCCACCTGAAGGATATCTGGAAGGATGGGCTCATTAAGGGCTTTGTGACCCGTGCCCAGGCACTGCGATTGTTGAAGAAGAAGTTGCCAGGCACTTTTCTGCTGAGATTCAGTGAGACCTCCGCAGAGGGAGGCATCACCTGCTCATGGGTGGAACATCAGGATGATGACAAGGTGGTCCTCCGATCAGTAGAGCCGTACACTAAAGATATTCTGCAGTTGGTCCCGATTACAGAGATCATTCGTAATTACCAGTTACTGGCAGAGGAGAATGTGCCTGAGAACCCACTATGCTTCCTTTACCCTGGCATACCCAGGGATGAAGCTTTTGGACCCTATTACCGTGAGAAAGTTGATCTTGCGAAACACAGGAAATACTTAAGCCGGGAACTCATTGTAGTGTCCAACAGGCAGGTCGATGATGTGCAGTCACCACATCCGTTGCAGCAGCCAGAAGTGCTGGACATTGATCTGGATCTTCATCCAGAGTGGGAACAGACAGAGCCTGAGCTTCCCTTGGACCTGCGGGACCTAGGGAAGGACCTGCTAACTCTAAATATGAATGATATTGAAGCTCTGAAAAATGACGAGTTGACCCATGATGCTATCCTGCTGAGAGATGACCCGGTGTTACCTATCCTGTCCCCTGGCGATGAGGCCCCATCTTCTGTGAGCCACTTCTTCATGGATGGTGCCACTACTTTTGATTTCCCTAATTACTAGGAATTAAATTTCTCCCTGTTTCCTTCCTTCCCCCTCTACCCCTCTCCATCGTGATATCGATGTTTGGTGGAAATCAAGAGCCTGGTGTTTGTTGTGGGGGGAGGGAGATGGAATTAGGAAAAGGAAGAAACAGAACTTTACAGCTTCCTGTTCTCCCATGCCCAGTGCCAAATTGTCTCTTCTTTCTCCCCTTGCACTTTGTGAAGTCCTGTGTACCCAGTTTTTCCTTGCAAGGGATAAGCAATGTCTGCCTCATTTTACTTGGGGAGGCTCTCTCCAGAAGTGGGAGTCTAGTAAATAGATCAATAATAACTGGGTTATCTAATCATTTGAAGTTTATAAAGCCCTTTTCTCCCTAAAAGTAGCCATCCTATGAAGCAAGTACAAGTTCTACTGTATTCATTTTACAAATAGAGATTTATCCATGGTCATGCAGCTATGTGAGCTCAACCCCAGAACTCCTGACCCTGTTCTGTGCTCTTCCTGTGCTGCTTTCCTGTCCTGCCTTTGCCCTCATTACCCCTGGCGAAAGTTTCACATGTGCACTTTGATATCTGGAACCACTTAGTGATGAGCAGCTGGTTGTCTAAAGCTCTGTCTGATAACAAATGATGCTGGGAGCCAAGCAAGTACTGCTATTATGATAGTTAACCAGTAGAACTGGACAAGAGCAAAGCCAGTACAATAAACTGTGTTCTCTAACAAAAAAAAAAAAA

>UoN.Pci.T.18164_UoN.Pci.T.18164.1

GGAGCTGGGGGCGGGGAGGGAGCGCACAGTGGAGAGATCCCCGCCAGGACAGAGTGTGCCTGGGGAGGGGAGAAGGGGCAGGCCGGGGAGCGGGGATCGTCGCCGGCAGGAAGCCATCCCTCGCAGTTCGAGCGCGGCCAGGCTCGGCCAGCCCTCGGTGACGTTACTCATCTTCCAGGGATCTCTCCTCTCCTCTCTTCCCCTCCCCTCCCTCTTACCCTCTTTCCTCTCTCCCTCTCCACCCCTCCTCCCTGTCCCCTCCCCCTCTCTCCCTCGAATCCCTGTCTCTGCACATCTCTCTCCATCAGCTTCCTCCGGACCTTCGCCGCCACCACCAGCAGCAGCAGGAGCAGCAGCACTTCGGCGGCCGAGGCAGCAGGAGAGGGGCTGCCAGCGGCTCCCCCCCCCCCCGGGGCTCCCATCCCCCCTCCTTCTCCACCTCCTCCCTGCCCTGCCCTGCCCTTCCCAGCTCGCCTTGAAGGGGATCCCCCTTCTTTCTCTCTGCTCAGTCCCCCCCTGCTGCGCCCCAGGATCCCGGCCCCCCTCCCCCTGCCTAACCCCCTCCTGAGCTCGAGGAGCCGCTCGGCGGAGACAAGGATGTGACTGCACCCGGCGGGCGGAGGGTGCACTCGGCGGCGGCGGCGGTCGGAGCGAAGCTGCGGGCGCCCGGGGCTCCCTCTCTCCTCGCTCGCCCTCTCTTCTCCCGCTTCCCCTGTCCCCAGTCCGCGGCCCCCGGCGGTCCGGCTGCCCCGACCCCGCGCCCCAGACCTCGCCGCCTCGGGGCACCCTTCCCCTGCCCCGGACCGCCACTCTCCGGCCGCCCCCGTGCTCCAGCGCTGACCCTGTCCGCCGGCGGGGAAGGAGGAGGAGGAGGAGGCGGCGCCGCCGAACCACCGGGCGAGAACATGTCGGAAGTGCTGCCGTTTAACGAGGAAAAGCTGGGGCACTATGGAGATGGGGGGGACGTGGGCCAGATCTTCACGTGCCGCCTGCAGGATACCAACAACTTCTTCGGGGCGGCTCAGAACAAGAGACCGCCCAAACTGGGCCAGATCGGCAGGAGCAAGAGAGTTGTTATTGAAGATGATAGGATTGATGACGTGCTGAAAAATATGACAGACAAGGCACCTCCTGGTGTTTAAAGCCCCCAAAGACACTGAGTTAAGGGAGAAAATGATGATAACAAACACAGCAACTTCACACAGTTTATTGGCAAAAAAAAAAAAGCACTTTGAAAAAAATCTTCATTACTCTAGCCTGCAGTCCACAATGCGGACACACCACCAACCTGTATCTTGTGTCAGGCCTGGGGACAGAAGAGGTGTGAGCATAGCAAGGACAGAAAGGCTCTAGGGTCTTCTGCAGAAATAAAAACAAAAAGCAAAAAAAAAAAGATTATTCAATAAATTAGTTGATTTACACACACTCATATGGAAATTGACATCAGCTGCAGCTGTTTGTCTAAATTAATATGCGTTTCCTTTTATATACAGTGAATATTGCGCAATTCTAGACCTGGATTTCCAGCCACTTTAAATAATGCAGCAGCAGCAGCAGCAACCGCCTGCCCAGCAAAATGCCTCTGGGTTCCTGGTGGGAGGGGGGGGGCTGGCATCCCCTTTGCTGATCTGGCTGCCCTGAAAGTCAGCCCTTTGTTTACATTATTCCCATCTGGCAAAAATGATTTCTGTGCACTTAGATGTGAAATGCTGTCCGTTTCATTTTTACAAAAATATTCATATCTTTGGATGTGTGTAAAACAAAACAAAACAAGAAAACAAAAAAAACCCCAGAATGACTTATATATAGATATATAGATAGTTATTCTATTTCGTTTTCCCGCCACCTTGGAAGGTCTTCCTTGGTGGGATGTGTGTGCGCTCCACAGAACAAAACCAACCCACTTTCCCTAAGGAATGCCTCTAGGAAGAGTTTTCTTCTGTTTTCAATGTGCTGTGAAGCTTGTGTTCTCTGGACTGCTTGCAAGCAATCCAGGCTCCAGTTCACACGGACTGAATTTTATATGGGGGGGGAGGAGGAAGGGGAAAAGAAGCCTTCTTCTCTGCGTTGTATGAGCCCCCATCCCGGTTCTTTCCTCCTCCCGGTGTTGTGGCGACTCATCTTCTTCAATGAATGGACTGTACAAAAATGTGATCATGAGAGTGCCAAATATCTGGAAACGCCAAAAGGCAGTTCAGTTTGGTTCCCCTTCTCTGTGCTCCCTGAGCCCACGTGAAGCAGTGCTTTGTCAGAGCCTCTCTTTTCTGGGCTGTTTATCGGGCTTTCTCTTAAGGCAGAACCGCTCTCCTTTTTGCATGATGTTTGCAGAGATGCCTCTTGTGCAGGGGCTGGGGGTGGGGGGTCTTTTCACCATTTTTGTTTTCTTTCTTCTTTTGGGTTTGCAACTTTGGATGACAGACCTCAAAGAGGTTTCTTTCACCTTCCCTTCTGCTACCCCCTCCCCTTTCTCCTTCCCCCCCTCCCTTTTCTTAATTCAATATTGTGTATATGGTGCACTGTTAACCACTACACAGGGCTATTTCTGCAGTGTACAATAAAAGGGACTGTTCCTGTGTCTAATCTCAAAAAAAAA

>UoN.Pci.T.18164_UoN.Pci.T.18164.2

GGAGCTGGGGGCGGGGAGGGAGCGCACAGTGGAGAGATCCCCGCCAGGACAGAGTGTGCCTGGGGAGGGGAGAAGGGGCAGGCCGGGGAGCGGGGATCGTCGCCGGCAGGAAGCCATCCCTCGCAGTTCGAGCGCGGCCAGGCTCGGCCAGCCCTCGGTGACGTTACTCATCTTCCAGGGATCTCTCCTCTCCTCTCTTCCCCTCCCCTCCCTCTTACCCTCTTTCCTCTCTCCCTCTCCACCCCTCCTCCCTGTCCCCTCCCCCTCTCTCCCTCGAATCCCTGTCTCTGCACATCTCTCTCCATCAGCTTCCTCCGGACCTTCGCCGCCACCACCAGCAGCAGCAGGAGCAGCAGCACTTCGGCGGCCGAGGCAGCAGGAGAGGGGCTGCCAGCGGCTCCCCCCCCCCCCGGGGCTCCCATCCCCCCTCCTTCTCCACCTCCTCCCTGCCCTGCCCTGCCCTTCCCAGCTCGCCTTGAAGGGGATCCCCCTTCTTTCTCTCTGCTCAGTCCCCCCCTGCTGCGCCCCAGGATCCCGGCCCCCCTCCCCCTGCCTAACCCCCTCCTGAGCTCGAGGAGCCGCTCGGCGGAGACAAGGATGTGACTGCACCCGGCGGGCGGAGGGTGCACTCGGCGGCGGCGGCGGTCGGAGCGAAGCTGCGGGCGCCCGGGGCTCCCTCTCTCCTCGCTCGCCCTCTCTTCTCCCGCTTCCCCTGTCCCCAGTCCGCGGCCCCCGGCGGTCCGGCTGCCCCGACCCCGCGCCCCAGACCTCGCCGCCTCGGGGCACCCTTCCCCTGCCCCGGACCGCCACTCTCCGGCCGCCCCCGTGCTCCAGCGCTGACCCTGTCCGCCGGCGGGGAAGGAGGAGGAGGAGGAGGCGGCGCCGCCGAACCACCGGGCGAGAACATGTCGGAAGTGCTGCCGTTTAACGAGGAAAAGCTGGGGCACTATGGAGATGGGGGGGACGTGGGCCAGATCTTCACGTGCCGCCTGCAGGATACCAACAACTTCTTCGGGGCGGCTCAGAACAAGAGACCGCCCAAACTGGGCCAGATCGGCAGGAGCAAGAGAGTTGTTATTGAAGATGATAGGATTGATGACGTGCTGAAAAATATGACAGACAAGGCACCTCCTGGTGTTTAAAGCCCCCAAAGACACTGAGTTAAGGGAGAAAATGATGATAACAAACACAGCAACTTCACACAGTTTATTGGCAAAAAAAAAAAAGCACTTTGAAAAAAATCTTCATTACTCTAGCCTGCAGTCCACAATGCGGACACACCACCAACCTGTATCTTGTGTCAGGCCTGGGGACAGAAGAGGTGTGAGCATAGCAAGGACAGAAAGGCTCTAGGGTCTTCTGCAGAAATAAAAACAAAAAGCAAAAAAAAAAAGATTATTCAATAAATTAGTTGATTTACACACACTCATATGGAAATTGACATCAGCTGCAGCTGTTTGTCTAAATTAATATGCGTTTCCTTTTATATACAGTGAATATTGCGCAATTCTAGACCTGGATTTCCAGCCACTTTAAATAATGCAGCAGCAGCAGCAGCAACCGCCTGCCCAGCAAAATGCCTCTGGGTTCCTGGTGGGAGGGGGGGGGCTGGCATCCCCTTTGCTGATCTGGCTGCCCTGAAAGTCAGCCCTTTGTTTACATTATTCCCATCTGGCAAAAATGATTTCTGTGCACTTAGATGTGAAATGCTGTCCGTTTCATTTTTACAAAAATATTCATATCTTTGGATGTGTGTAAAACAAAACAAAACAAGAAAACAAAAAAAACCCCAGAATGACTTATATATAGATATATAGATAGTTATTCTATTTCGTTTTCCCGCCACCTTGGAAGGTCTTCCTTGGTGGGATGTGTGTGCGCTCCACAGAACAAAACCAACCCACTTTCCCTAAGGAATGCCTCTAGGAAGAGTTTTCTTCTGTTTTCAATGTGCTGTGAAGCTTGTGTTCTCTGGACTGCTTGCAAGCAATCCAGGCTCCAGTTCACACGGACTGAATTTTATATGGGGGGGGAGGAGGAAGGGGAAAAGAAGCCTTCTTCTCTGCGTTGTATGAGCCCCCATCCCGGTTCTTTCCTCCTCCCGGTGTTGTGGCGACTCATCTTCTTCAATGAATGGACTGTACAAAAATGTGATCATGAGAGTGCCAAATATCTGGAAACGCCAAAAGGCAGTTCAGTTTGGTTCCCCTTCTCTGTGCTCCCTGAGCCCACGTGAAGCAGTGCTTTGTCAGAGCCTCTCTTTTCTGGGCTGTTTATCGGGCTTTCTCTTAAGGCAGAACCGCTCTCCTTTTTGCATGATGTTTGCAGAGATGCCTCTTGTGCAGGGGCTGGGGGTGGGGGGTCTTTTCACCATTTTTGTTTTCTTTCTTCTTTTGGGTTTGCAACTTTGGATGACAGACCTCAAAGAGGTTTCTTTCACCTTCCCTTCTGCTACCCCCTCCCCTTTCTCCTTCCCCCCCTCCCTTTTCTTAATTCAATATTGTGTATATGGTGCACTGTTAACCACTACACAGGGCTATTTCTGCAGTGTACAATAAAAGGGACTGTTCCTGTGTCTAATCTCAAAAAAAAA

>UoN.Pci.T.19069_UoN.Pci.T.19069.1

GGCGGGGGCATCAATCCTGCCTCCCCACTCTTTTCACTGGAGCCTTTGGTGGGTGGGTGGGCAGGTTGGTGGCAGGCAGACCTGCAGCTACTGGAGATAAGGCCAAAGAGCACCCTGGACAGCGCCTTCCTTTCATCCCCTTCCCTCCCCCCCATCTTGGGCTTCTGTTAGTTTAGAAGCCTCTTTGACACCCCCTTTCCCCTGTTTACCTGAGGGAGTGGGAGAAGAACCCTAAAGAAAGGACAGCAGCCTTGGCCAGTCAGGACAATGGTGCTCCCTTCTTCCGCCAAAGCTTTGTCGCTGTTCCCTCCTATCCCATATCACCACTCAGCTTGGTAGATAGTTGACTTTCCCCACAAACGTCATGTGCCCGCTGGACTTGATCAGATCTCCTCTTACCCACTGACCCCATCTGTCCCTAGGCCTGCTTTGCTATTTCACTGTAACCATGCCCAGAAGATATCCCAGAAGATCTGTCTGCCTGTCTAGGAGGCCCCTCAGCTCTCACATTTCTTTCCTCTCCACCCTACCCACCCCCAACCCCCAGTATGACCAGCTCATTACCTTCCCTAACATTCTGGGACACTCTGGCACTGTTTCTTTTCCTCAGACTTCCTGCTGTACCCATTCCTGGGAGGACTTTCTTTCTCCCAGATATCCTAATTATTAAAAGCCTGTTTTTAGTGAGATTCCTCAGCCCATCTTTTTCATGTCCGGTTCATTGTTGTGCTTCTAACCCTGATCCCTCCTCCCTCTCTCTGGGCCAGTTCTGACAACCCTCCTTCCTATCTCCTAGATGTCCAGCTCGCCGCTGTCCAAGAAGCGTCGCGTGTCCGGGCCTGATCCAAAACCGGGTTCTAACTGCTCACCTGCCCACCCTGTTCCCTCTGAAGTGCCCACTGCACCTGCTAACGGAATGGCAAAGAACGGGAACGAGGCAGATATCGACGAGAGCCTATACTCCAGGCAGCTGTATGTACTGGGCCATGAGGCCATGAAGCGACTTCAGACTTCCAGCGTCCTAGTGTCTGGGCTCCGGGGCCTGGGAGTTGAGATTGCTAAGAACATAATCCTTGGTGGAGTCAAAGCTGTTACCCTCCATGATCAGGGTGCTGCCCAGTGGGCTGATCTCTCTTCCCAGTTCTACCTTCGAGAAGAAGATGTAGGCAAGAACCGGGCAGAGGTTTCACAACCCCGTCTGGCTGAGCTCAATGCCTATGTTCCCGTCTGTTCCTACACAGGGCCTCTCACTGAGGACTTCCTTAGCAACTTCCAGGTGGTTGTCCTCACCAACTCTCCCCTGGAGGAGCAGCTGCGTGTGGGCGAGTTCTGCCATGGTCATGGTATCAAGCTGGTGGTGGCTGACACCCGGGGCCTCTTTGGACAGCTGTTCTGTGACTTTGGTGAGGAAATGGTCCTGACAGACGCGAATGGGGAGCAGCCACTTAGCGCCATGGTCTCCATGGTGACCAAGGACAGCCCCGGTGTGGTGACCTGCCTGGATGAGGCGCGGCATGGCTTTGAGAGTGGCGACTTCGTCACCTTCACTGAGGTACAAGGGATGAGTGAGCTTAATGGCATATCGCCCGTGGAGATCAGAGTCCTCGGTCCATATACCTTCAGCATCTGTGACACGGCCAGATTCTCTGACTATGTCCGTGGTGGAATCGTCACCCAGGTCAAAGTGCCCAAGAAGATCAGCTTTGTGAGTCTCTCCAGGGCAGGTGGTGGGCAGGGGACTGGTGCCGTGGAGAGGTGAGATGGGAGACCTCAGTTTCCTCATCTGTAAAACGGGGATCATAATGAACATGTACCTCCTAGGACCGCGAGATCATATAGTGCCTGGCACATAGTAGGCTCTTAAGAAATGCTTGTTTCCTTTTTTCCTGGCTTGATCCTCCTTGTCCTTTAGAAATCACTACCTGTATCCCTGGCTAAGCCAGAATTTGTGATGACGGATTTTGCCAAGTTTTCTCGCCCTGCCCATCTCCATGTGGCCTTTCAGGCCCTACACCAGTTCCACAGCCAGCGAGGGCGGCTGCCCCACCCTCAAAATCAGCTGGATGCAGCAGAGATGGTGAGCCTGGCGCAGGCGATTAAGGAAGCTGCCTCGCCCCGAGTGCTGCAGGAGGATTTGGATGAGGAGCTGGTACGGCAGTTGGCCTACATGGCAGCTGGAGACCTGGCTCCTATAAATGCTTTCATTGGGGGCCTGGCTGCCCAGGAAGTTATGAAGGCGTGCTCGGGAAAGTTTATGCCCATCATGCAGTGGTTGTACTTTGATGCCCTGGAGTGCCTGCCTGAGGACAGAGAGGCCCTAACAGAGGACAGTTGCCGCCCGCGCCAAACACGCTATGATGGGCAGGTGGCAGTGTTTGGCTCTCATCTGCAGGAAAAGCTTGGCAGGCAGAAGTACTTCCTGGTTGGGGCAGGGGCCATTGGTTGTGAGCTGCTCAAGAACTTTGCCATGATTGGCCTGGGCTGTGGGGATGGTGGAGAGGTCATCGTCACAGACATGGACACTATTGAGAAGTCCAATCTCAACCGGCAGTTCCTCTTCCGGCCCTGGGATGTCACGAAGCTCAAGTCTGACACAGCAGCTGCTGCTGCCCGTCAAATGAACCCACAGATGCACGTGACGAGTCACCAGAACCGCGTGGGGCCTGACACTGAGCGCATTTATGATGATGATTTCTTCCAGGCCCTGGATGGCGTGACCAACGCATTGGACAACGTTGACGCACGCATGTATATGGACCGTCGCTGTGTGTACTACCGGAAGCCGCTGCTGGAGTCGGGCACCCTGGGTACCAAGGGCAATGTCCAGGTGGTCATCCCTTTCCTTACTGAGTCCTACAGCTCCAGTCAAGACCCCCCGGAAAAATCCATCCCCATCTGTACTCTCAAGAACTTTCCCAATGCAATTGAGCATACGCTGCAGTGGGCCCGGGATGAGTTTGAAGGTCTCTTCAAGCAGCCGGCAGAGAGTGTCAATCAGTATCTCACGTGAGTGGCCCCAGCCCCTTTTCCCACACTCCTGGCTTGTGGCCCCAATGCCCAGCCTGGATTTTCTGTTGGCTTACTTGTGAGCTTCTTGAGCTAGCCCTAAATCCTCCTGCTGTCCCGCACTGCAGCCCTTGACACCCCTGTCCTAATGCCATTGGTCTCCTCCCCCTTGCCCACAGAGACCCAAAGTTTGTGGAGCGAACACTGCGGCTGGCAGGGACCCAGCCCCTGGAGGTGCTGGAGGCCGTACAGCGGAGCTTGGTGCTACAGCGCCCCCGTACCTGGGCCGACTGTGTGGCGTGGGCCTGCCTTCACTGGCACGCCCAGTATGCCAACAACATCCGTCAGCTGTTGCACAACTTCCCTCCTGAGCAGCTGACAAGCTCTGGTGCCCCCTTTTGGTCTGGACCCAAGCGATGTCCTCATCCCCTCACCTTTGATGTCCACAACCCCTTGCATTTGGATTACATCATGGCGGCCGCCAACCTCTTTGCTCAGACCTATGGGCTGGTGGGCTCTAGGGACCGGACGGCTGTGGCCACGCTTCTCCAGACCGTGCACGTCCCGGAATTCACTCCCAAGTCTGGAGTCAAGATCCACGTCTCTGACCAGGAGCTGCAGAGTGCCAACACTTCAGTTGGTAAGCACCAATCCCCCTTGGCCTCGGGCCTCCAGTTCTTACTAGCTCCCAGGAGTCTCCGTCCTTTCCCTGCTCTGCCCTCATCTCCTCAGCCTGGTCTTCTTCCTTGTGCCCCCTGCTTCTGGTGGGGCCACAGATGACAGCCGGCTGGAGGAGCTGAAGGCAACACTGCCCAGTCCAGAGAAGCTGTCTGGATTCAAGATGTATCCCATTGATTTTGAGAAGGATGATGACAATAACTTCCACATGGATTTCATCGTGGCCGCCTCCAACCTTCGGGCTGAGAACTATGACATTGCCCCTGCCGACCGGCACAAGAGTAAGCTGATCGCAGGGAAGATCATCCCAGCCATCGCCACGACTACTGCAGCTGTGGTTGGCCTCGTGTGCCTGGAGCTGTACAAAGTGGTGCAGGGCCACCAGCGGCTTGAGGCCTACAAGAATGGCTTTCTCAACCTTGCCCTGCCCTTCTTTGGCTTCTCAGAGCCCATCGCGGCTCCCCGTCACAAGGTGAGGTGCTAACAGACCGTTGGGCCCAAGTGGGGAGCAGGGAGGGTAGAGGGCTGTTGACTTGCTTGGTCTTTCTCACTTGCAGTATTATGACAATGAGTGGACACTTTGGGATCGCTTTGAGGTCAAGGGGCTCCAACCTGGGGGCGAGGAGATGACTCTGAAGCAATTTCTGGACTACTTCAAGACAGAGCACAAGCTGGAGATCACAATGCTGTCCCAGGGTGTATCCATGCTGTACTCCTTCTTCATGCCAGCTGCTAAGCTCAAGGAGCGGCTGGACCAACCTATGACGGAGATTGTGAGCCGGGTGTCAAAAAGGAAGCTGGGCCGCCACGTGCAAGCACTGGTTCTAGAGCTCTGCTGCAATGACGACAGTGGCGAGGACGTGGAGGTGCCCTACGTGCGTTATACCATCCGCTGACTGGGTGGCAGGCGGGTGCCTTTGCCTTCCCTCCCCCTTCTGCTGCCCTCTCCCGTGCCCGCCCCCTCAGGGGGGCTGGGCCCCCCCTTCCCCCCACCTTTGCCTAGGGCACTGGAGGGCATGACTTTGTACTGAACCCATAATAAAGTTATTGATGGAAACCAAAAAAAAA

>UoN.Pci.T.19069_UoN.Pci.T.19069.1

GGCGGGGGCATCAATCCTGCCTCCCCACTCTTTTCACTGGAGCCTTTGGTGGGTGGGTGGGCAGGTTGGTGGCAGGCAGACCTGCAGCTACTGGAGATAAGGCCAAAGAGCACCCTGGACAGCGCCTTCCTTTCATCCCCTTCCCTCCCCCCCATCTTGGGCTTCTGTTAGTTTAGAAGCCTCTTTGACACCCCCTTTCCCCTGTTTACCTGAGGGAGTGGGAGAAGAACCCTAAAGAAAGGACAGCAGCCTTGGCCAGTCAGGACAATGGTGCTCCCTTCTTCCGCCAAAGCTTTGTCGCTGTTCCCTCCTATCCCATATCACCACTCAGCTTGGTAGATAGTTGACTTTCCCCACAAACGTCATGTGCCCGCTGGACTTGATCAGATCTCCTCTTACCCACTGACCCCATCTGTCCCTAGGCCTGCTTTGCTATTTCACTGTAACCATGCCCAGAAGATATCCCAGAAGATCTGTCTGCCTGTCTAGGAGGCCCCTCAGCTCTCACATTTCTTTCCTCTCCACCCTACCCACCCCCAACCCCCAGTATGACCAGCTCATTACCTTCCCTAACATTCTGGGACACTCTGGCACTGTTTCTTTTCCTCAGACTTCCTGCTGTACCCATTCCTGGGAGGACTTTCTTTCTCCCAGATATCCTAATTATTAAAAGCCTGTTTTTAGTGAGATTCCTCAGCCCATCTTTTTCATGTCCGGTTCATTGTTGTGCTTCTAACCCTGATCCCTCCTCCCTCTCTCTGGGCCAGTTCTGACAACCCTCCTTCCTATCTCCTAGATGTCCAGCTCGCCGCTGTCCAAGAAGCGTCGCGTGTCCGGGCCTGATCCAAAACCGGGTTCTAACTGCTCACCTGCCCACCCTGTTCCCTCTGAAGTGCCCACTGCACCTGCTAACGGAATGGCAAAGAACGGGAACGAGGCAGATATCGACGAGAGCCTATACTCCAGGCAGCTGTATGTACTGGGCCATGAGGCCATGAAGCGACTTCAGACTTCCAGCGTCCTAGTGTCTGGGCTCCGGGGCCTGGGAGTTGAGATTGCTAAGAACATAATCCTTGGTGGAGTCAAAGCTGTTACCCTCCATGATCAGGGTGCTGCCCAGTGGGCTGATCTCTCTTCCCAGTTCTACCTTCGAGAAGAAGATGTAGGCAAGAACCGGGCAGAGGTTTCACAACCCCGTCTGGCTGAGCTCAATGCCTATGTTCCCGTCTGTTCCTACACAGGGCCTCTCACTGAGGACTTCCTTAGCAACTTCCAGGTGGTTGTCCTCACCAACTCTCCCCTGGAGGAGCAGCTGCGTGTGGGCGAGTTCTGCCATGGTCATGGTATCAAGCTGGTGGTGGCTGACACCCGGGGCCTCTTTGGACAGCTGTTCTGTGACTTTGGTGAGGAAATGGTCCTGACAGACGCGAATGGGGAGCAGCCACTTAGCGCCATGGTCTCCATGGTGACCAAGGACAGCCCCGGTGTGGTGACCTGCCTGGATGAGGCGCGGCATGGCTTTGAGAGTGGCGACTTCGTCACCTTCACTGAGGTACAAGGGATGAGTGAGCTTAATGGCATATCGCCCGTGGAGATCAGAGTCCTCGGTCCATATACCTTCAGCATCTGTGACACGGCCAGATTCTCTGACTATGTCCGTGGTGGAATCGTCACCCAGGTCAAAGTGCCCAAGAAGATCAGCTTTGTGAGTCTCTCCAGGGCAGGTGGTGGGCAGGGGACTGGTGCCGTGGAGAGGTGAGATGGGAGACCTCAGTTTCCTCATCTGTAAAACGGGGATCATAATGAACATGTACCTCCTAGGACCGCGAGATCATATAGTGCCTGGCACATAGTAGGCTCTTAAGAAATGCTTGTTTCCTTTTTTCCTGGCTTGATCCTCCTTGTCCTTTAGAAATCACTACCTGTATCCCTGGCTAAGCCAGAATTTGTGATGACGGATTTTGCCAAGTTTTCTCGCCCTGCCCATCTCCATGTGGCCTTTCAGGCCCTACACCAGTTCCACAGCCAGCGAGGGCGGCTGCCCCACCCTCAAAATCAGCTGGATGCAGCAGAGATGGTGAGCCTGGCGCAGGCGATTAAGGAAGCTGCCTCGCCCCGAGTGCTGCAGGAGGATTTGGATGAGGAGCTGGTACGGCAGTTGGCCTACATGGCAGCTGGAGACCTGGCTCCTATAAATGCTTTCATTGGGGGCCTGGCTGCCCAGGAAGTTATGAAGGCGTGCTCGGGAAAGTTTATGCCCATCATGCAGTGGTTGTACTTTGATGCCCTGGAGTGCCTGCCTGAGGACAGAGAGGCCCTAACAGAGGACAGTTGCCGCCCGCGCCAAACACGCTATGATGGGCAGGTGGCAGTGTTTGGCTCTCATCTGCAGGAAAAGCTTGGCAGGCAGAAGTACTTCCTGGTTGGGGCAGGGGCCATTGGTTGTGAGCTGCTCAAGAACTTTGCCATGATTGGCCTGGGCTGTGGGGATGGTGGAGAGGTCATCGTCACAGACATGGACACTATTGAGAAGTCCAATCTCAACCGGCAGTTCCTCTTCCGGCCCTGGGATGTCACGAAGCTCAAGTCTGACACAGCAGCTGCTGCTGCCCGTCAAATGAACCCACAGATGCACGTGACGAGTCACCAGAACCGCGTGGGGCCTGACACTGAGCGCATTTATGATGATGATTTCTTCCAGGCCCTGGATGGCGTGACCAACGCATTGGACAACGTTGACGCACGCATGTATATGGACCGTCGCTGTGTGTACTACCGGAAGCCGCTGCTGGAGTCGGGCACCCTGGGTACCAAGGGCAATGTCCAGGTGGTCATCCCTTTCCTTACTGAGTCCTACAGCTCCAGTCAAGACCCCCCGGAAAAATCCATCCCCATCTGTACTCTCAAGAACTTTCCCAATGCAATTGAGCATACGCTGCAGTGGGCCCGGGATGAGTTTGAAGGTCTCTTCAAGCAGCCGGCAGAGAGTGTCAATCAGTATCTCACGTGAGTGGCCCCAGCCCCTTTTCCCACACTCCTGGCTTGTGGCCCCAATGCCCAGCCTGGATTTTCTGTTGGCTTACTTGTGAGCTTCTTGAGCTAGCCCTAAATCCTCCTGCTGTCCCGCACTGCAGCCCTTGACACCCCTGTCCTAATGCCATTGGTCTCCTCCCCCTTGCCCACAGAGACCCAAAGTTTGTGGAGCGAACACTGCGGCTGGCAGGGACCCAGCCCCTGGAGGTGCTGGAGGCCGTACAGCGGAGCTTGGTGCTACAGCGCCCCCGTACCTGGGCCGACTGTGTGGCGTGGGCCTGCCTTCACTGGCACGCCCAGTATGCCAACAACATCCGTCAGCTGTTGCACAACTTCCCTCCTGAGCAGCTGACAAGCTCTGGTGCCCCCTTTTGGTCTGGACCCAAGCGATGTCCTCATCCCCTCACCTTTGATGTCCACAACCCCTTGCATTTGGATTACATCATGGCGGCCGCCAACCTCTTTGCTCAGACCTATGGGCTGGTGGGCTCTAGGGACCGGACGGCTGTGGCCACGCTTCTCCAGACCGTGCACGTCCCGGAATTCACTCCCAAGTCTGGAGTCAAGATCCACGTCTCTGACCAGGAGCTGCAGAGTGCCAACACTTCAGTTGGTAAGCACCAATCCCCCTTGGCCTCGGGCCTCCAGTTCTTACTAGCTCCCAGGAGTCTCCGTCCTTTCCCTGCTCTGCCCTCATCTCCTCAGCCTGGTCTTCTTCCTTGTGCCCCCTGCTTCTGGTGGGGCCACAGATGACAGCCGGCTGGAGGAGCTGAAGGCAACACTGCCCAGTCCAGAGAAGCTGTCTGGATTCAAGATGTATCCCATTGATTTTGAGAAGGATGATGACAATAACTTCCACATGGATTTCATCGTGGCCGCCTCCAACCTTCGGGCTGAGAACTATGACATTGCCCCTGCCGACCGGCACAAGAGTAAGCTGATCGCAGGGAAGATCATCCCAGCCATCGCCACGACTACTGCAGCTGTGGTTGGCCTCGTGTGCCTGGAGCTGTACAAAGTGGTGCAGGGCCACCAGCGGCTTGAGGCCTACAAGAATGGCTTTCTCAACCTTGCCCTGCCCTTCTTTGGCTTCTCAGAGCCCATCGCGGCTCCCCGTCACAAGGTGAGGTGCTAACAGACCGTTGGGCCCAAGTGGGGAGCAGGGAGGGTAGAGGGCTGTTGACTTGCTTGGTCTTTCTCACTTGCAGTATTATGACAATGAGTGGACACTTTGGGATCGCTTTGAGGTCAAGGGGCTCCAACCTGGGGGCGAGGAGATGACTCTGAAGCAATTTCTGGACTACTTCAAGACAGAGCACAAGCTGGAGATCACAATGCTGTCCCAGGGTGTATCCATGCTGTACTCCTTCTTCATGCCAGCTGCTAAGCTCAAGGAGCGGCTGGACCAACCTATGACGGAGATTGTGAGCCGGGTGTCAAAAAGGAAGCTGGGCCGCCACGTGCAAGCACTGGTTCTAGAGCTCTGCTGCAATGACGACAGTGGCGAGGACGTGGAGGTGCCCTACGTGCGTTATACCATCCGCTGACTGGGTGGCAGGCGGGTGCCTTTGCCTTCCCTCCCCCTTCTGCTGCCCTCTCCCGTGCCCGCCCCCTCAGGGGGGCTGGGCCCCCCCTTCCCCCCACCTTTGCCTAGGGCACTGGAGGGCATGACTTTGTACTGAACCCATAATAAAGTTATTGATGGAAACCAAAAAAAAA

>UoN.Pci.T.19875_UoN.Pci.T.19875.1

GGGGCAGTGAGTGCAAAGCCGCCCTGGGCCTGCAGGTGGCGCTACAGTCTCCCCCGCCCTCTCGGCCGCCGTTGCTTACAACCGGGTGCTCAGCAGGCGAGAAGCGGAACGAGCTGTCGCTATGGCGGGCGAGCTCGAGAGTTCCAAGTCCCTGAGCGGCCTGTTAAGCGGCCTGGCCCAAAGCACCTTCCATGGGCACCCAGGTATCACGGAGGAGCTACTGCGGAGCCAACTTTACCCGGAGGTACCCCCAGAGGAATTCCGCCCTTTCCTGGAGAAGATGCGGGGGCTCTTGAAGTCTATTGCATCTGCAGACATGGATTTCAACCAATTAGAGGCATTCCTGACTGCTCAGACCAAAAAACAAGGTGGAATTACAGCTGATCAAGCAGCAGTCATTTCCAAATTCTGGAAAAACCACAAGACAAAAATCAAGGAGAGTCTAATCAATCAAAGTCGATGGGAAAACAGTCTGAAAGCCATGAATTGGAGAGTAGATTTGAAGTCTCAGTCAAAACATCTTGACCAAATTAATGCTCCTGTTGCTATTGTGGAACTAGAACTGGGGAAAAGTGGACAGGAATCTGAATGTCTGTGTTTGGAGTTTGATGAAGTCAAGCTTAACCAAATCCTGAAGAAGCTCACTGAGGTGGAAGACAGTATCAACACCCTGACAAACCCAGCCTAAACATGAAGGTGATTTCGGAAGAAGTTAGGAAATTCACAGCAGGAGCCACCACTGAGTTCTAACTATTATCTTTTTCTGTATGACATTCCACTGCATTAAATTCTCAAAATAGAA

>UoN.Pci.T.19875_UoN.Pci.T.19875.1

GGGGCAGTGAGTGCAAAGCCGCCCTGGGCCTGCAGGTGGCGCTACAGTCTCCCCCGCCCTCTCGGCCGCCGTTGCTTACAACCGGGTGCTCAGCAGGCGAGAAGCGGAACGAGCTGTCGCTATGGCGGGCGAGCTCGAGAGTTCCAAGTCCCTGAGCGGCCTGTTAAGCGGCCTGGCCCAAAGCACCTTCCATGGGCACCCAGGTATCACGGAGGAGCTACTGCGGAGCCAACTTTACCCGGAGGTACCCCCAGAGGAATTCCGCCCTTTCCTGGAGAAGATGCGGGGGCTCTTGAAGTCTATTGCATCTGCAGACATGGATTTCAACCAATTAGAGGCATTCCTGACTGCTCAGACCAAAAAACAAGGTGGAATTACAGCTGATCAAGCAGCAGTCATTTCCAAATTCTGGAAAAACCACAAGACAAAAATCAAGGAGAGTCTAATCAATCAAAGTCGATGGGAAAACAGTCTGAAAGCCATGAATTGGAGAGTAGATTTGAAGTCTCAGTCAAAACATCTTGACCAAATTAATGCTCCTGTTGCTATTGTGGAACTAGAACTGGGGAAAAGTGGACAGGAATCTGAATGTCTGTGTTTGGAGTTTGATGAAGTCAAGCTTAACCAAATCCTGAAGAAGCTCACTGAGGTGGAAGACAGTATCAACACCCTGACAAACCCAGCCTAAACATGAAGGTGATTTCGGAAGAAGTTAGGAAATTCACAGCAGGAGCCACCACTGAGTTCTAACTATTATCTTTTTCTGTATGACATTCCACTGCATTAAATTCTCAAAATAGAA

>UoN.Pci.T.23154_UoN.Pci.T.23154.1

CGCCAGCGGGAGGACGCTAGGCCTGCTCTCCGCCCTTCTCGCATTTGACCCCCCCCCTCCACCTCGGATAAGATCGGGCGCACAGCCTGCTTAGTTGAAGGGCGCCTGGACACGGAGCCGGCTGGCCGAGGGCGGGCTTCTGGGCGCTGTGAGAGGGAGGCTCTGCTCCCTGACTCCAGGCCCAGCCCAGCGGTAGGACAGAAGGACGGACGGACTGACGGAAGGACGGCCGGGGGGAGGATGAGCGGCGGGAGGCGGAAAGAGGAGCCGCCGCAGCCGCACCTGGCCAACGGTGCCCCGAAGGGCTCGGCCTGGAGCAAAGCGCTGCGGAGCGACGCGGCCTGGGAGGACAAGGATGAGTTTTTAGATGTGATCTACTGGTTCCGACAGATCATTGCGGTTGTCCTGGGAGTCATTTGGGGAGTGGTGCCACTGAAGGGCTTCGTGGGAATAGCAGTATTCTGCCTGATCAATGCTGGCATCTTGTACCTCTACTTCAGCAGCTTCCAACAGATCGATGAGGAAGATTATGGTGGGACGTGGGAGCTCACAAAAGAAGGTTTTATGACGTCTTTTGCACTGTTTATGGTTGTTTGGATCATCTTTTACACTGCCATCCACTATGACTGACAGGCTGCAGCCCACACCTGCTAGCACTGTTCATTCAAAAGGACATTCTAATCACAGGCCAGAAATGTGCCAGTGGTCAGGGGCATTTCAGCCAGAAGGAGACTGTGATGCCCCATTCTCCCAAACTGAGAATCAGCGTGTTGGTCATCAGTGTTTTATACCAGTGTCGTGTCTTGGAACTTTTAAACAAGCCATCCCTATTTTGAAAACATTACTGAACTGTTCCATCACGACCATATTCCATGCCTTTATTGACTACCCATCACTAATACCTGTTGGCTGAATGGATCTAGTAATTTCATTTACCTTAGGATCTGAATGAAACATTAAATTGTCTCGTCTTCCTCAGCTGTCCATCAGGCACACAATTTTAAAGATATCAATGGCATTTTATGTTTTGAGTTCTTGGACAAAGCATGGCAGAATGTGCGTGGTTCATAATACAGTACATGTCTAATAATAGTTTCCATTGTAGAATGTTTTAAGATAACTTGAATAAATTGAATCTTGTTAACAGATCTCCTAAGCCTTTGATTGAAAGTTTGAGGGGCTATCAGTGTAGCTCTTTGCTTTGTGTCTGCAGACAGCATTTACCTGGCCCAGGGTTGTCCAGCTGACTCAGCCTTTGCCTTCACCTACTGTGCCTCAGGCCTTCCTCTTTACACTTAGCACTCAGCCAGGTCTAGGCTATATCTCTTAACCTCTG

>UoN.Pci.T.20889_UoN.Pci.T.20889.1

GTCACTGGTTGGTGGGGGTAGGGGGGGGGAGCGGGAGCCGCCGCTGCTGCCGCCGGAGCAAACCGCGGGGACCGAGATGCAGGTGGGACCGGAACCGGAACCCCCCTTCTCATTCTTCTTTCCCCACCCGAACCCTCCCCCCTTTCCCGGGCCTGGGACTGGAGGGAGGGCCGAAGGTCCTGGAGCGGGGCCGGGCTCGGAAGCTGGGGGCTGGGGGAATATCCCCAGGGCAGCGCACCGCCGGAGGCCGGTGGAGCGGGGCGCAATGGGCGGAGGTGAAGGAGGCCGCCCACGGGCGGAGGGAAAGCCGGCGTTCTCCGGAGCCCCCCACCCCCGGAGGAGCGCTGGGGCTGGGCAAGGGAGCAAGGCTGCCCGCGGCCTCATCACTGCAGGGCCAGGGGGTCGGGGCGAGTGGGCAGCAGCAGCCGGGCTAAAGGAGCTACGGCGTTTGGGGGGGAGCGACCTCAAGGGACCCACACTGGGTGAGGGGTCCCCCCTGTTGTTGGTGTGGAACGTACGAGCTCTGGGGTGGAGGGAGTAGGCATGTCATGCTTGGGTGTCTCCCCCAGCATGCACCAAACCTCCCTCTTTCCCAGGCTCTCTTCATTGGCATTTGAGACTATTTCATGTGTTTCTGTCTCCTCGAGGCAGGGTCCGGGGTAGATGTAAAAGGCGGCTGAAAGGATAGGGAATGAAGGGAGGGTGAAGATGTTGGTCCGGAAGTGACCAGCAGAATTGGCGTGGGTTGCCTGTCAGGAAGGAGTTCTTGAGAAAATAAGAAACACAGGCCAGACTTCTTTAAGGCCACTCTCATTCCAGCCCTTTCCATGGAGGTCTGGCCTTAGGAAATTATTTCCTTTGTTCATTCCAGGTTCTGATGTCTCTCTGTGGAAATTCATTTTGTTGCTGAACCTAGGCCTGCGCAGAATTTCCCATCTTAGGGAACTGGAATAAGGAATCAGGGTTCTTAGGTTCTGTGCCTGACTAGAACTCGAAGCCACCACTTTTGCGACCTTAGGCAGTCATTCAACTTCTGAGTCTGTTTTCTTACCCATAAAATGGGCACAATAGCCCCTGGTTTACCTCTCTAATAGGGTTGCTGTGAAGTAGGAGAATGTGTGTGAAAATGCTTTGAAATCATAAAGTGCTATGTGAATGCTGTTATTGGTCAAGCAGAAATTTTTCTAGAAACTCTTCCTAACCTGGTCAACTAGTGATGCTACAAATAGTGGCTATGAGTCTGGTCTGAAGAAGCACAGGATTAGTGGCCCTCAGTTCTGAGCCGGGTTTTTCCCGGAGTCCAGTTGCTCTGGGCTGTGGGACTAAGGGCCTGTGTGCTCATTTTCACAGCTTGTCTGACCTTTAGCCCCTTTTGCTTTCTTTTATTCTTTGACATTAGAAAATACTTCATCCCAGCCTTCTTATAGTTTCTTTTATTCTTGAGACTGAGGGGAAGAAGTAAGGTAGCAGTACTCAATATTTGGATGGTGAGGAGCTACTGAGGATGGAAGGGGGTGGGTGGGAATGATGCCTTCAAGGCAGTACCCTCATACCATAATGCTTAAAATGCCAATGAGCCTTAGGGAGAGAAGCAAGGTGGCAGTCACCTACCTATTGCTGACCTGTTAGGGACCTTTAGAGTAAGATATTGGATATATTTATTTCTGAAAGAGAAGAGATTGGAAAGGAAGCTTTCTGTCTCTGAAGGGTTTCTTTTTTGGAGGATGTCGACCAGCTTACCTTTAACCCTTCTGGGATCAAAGCCAAAGGATGTGGGCTGAGATTACAGCAGGAAAGATAGAGGCTAGATATCAGAAAAAAACCTACCAGTGCTTGAGAGGTGTGAGAACCTGGAATATGGGAAGGAAGCTAACATATTTTGAAGGACTTTTCTTGAATCTGTCTGAATCAGAGAGGAACAAGGTGAACAGAAGGCAGTGTGGAAAAGTGGATGGCTTCTCAGTGAGGGTGGAACAATTGGGGAATCTCTGGTATTTGGGGAAAGGTCTAGAACTTATTCTCTATAAGGTATAATTCTAATACTGACTCCTATATATGTGCCTCTTTTTCCCTTCCACAGTTGCTGCAGATGACCCTGAGTCTTCTGGCCTCCCCCCCCACTTCCTTCCCTCCCCTGGGTCCCCCACTCTCCATTTCATCTGGCTCATCATGAATGGTGCCCCTTCCCCTGAGGATGGGACTTCCCCTTCTCCTCCCCCTCTGCCACCACCACTTCCACCACCTCCTCCCCCAAGTTGGCGGGAGTTCTGTGAATCCCACGCTCGGGCAGCAGCCCTAGACTTTGCCCGACGCTTCCGCCTCTACTTGGCTTCCCATCCCCAGTATGCAGGGCCTGGGGCTGAGGCAGCCTTCTCCCGACGTTTTGCTGAGCTCTTCTTGCAGCACTTTGAGGCTGAGGTGGCCCGGGCCTCAGGTTCCCTTTTATCATCTGGTCCGATCCCCCTGAGTCCCAGTACGGAAACCCCATCACGTGATCTTTCCCTGGAGAGCTGCAGGAACAGTGGGCCTCTGACTGTGTTGGGTCCTTCTCGATCCTCTGAAGACCTGGCTGGCCCTCTCCCTTCTTCAATCTCTTCATCCTCCTCAAAGCCTAAGCTGAAGAAACGTTTCTCCCTCCGCTCTGTGGGCCGATCAGTGCGAGGCTCGGTCCGAGGGATCTTGCAGTGGCGGGGAGCTGGTGATCCCCCCTCTCCAGTGGGACCCTTGGAGACCTCTGCCTCTGGACCACCTGTCCTGGGTGGAAACAGCAACTCCAACTCCTCTGGTGGGGGTGGTAGCACCAATGTGGGGCCAACTAGCGGTGGGGCATCCCCTGGGGAAAGATGGACACACCGCTTTGAGAGGCTGAGACTAAGTAGGGGAGGGGGACCCTTAAAGGATGGGGCAGGGACAGTGCAAAGGGAGGGGCTACTGAGCTTCATGGGAACAGAGGAGGCAGCTGTTGGTGGAGGAGAAGGTCGAGGGGGAGGAGGAACTGGAACCTCATCTGGGGGTGGGGGACCACCAAGGTGGCAGAAGTGTCGCCTGCTGCTTCGGAGTGAGGGGGAAGGAGGAGGAAGTCGGCTGGAGTTCTATATCCCACCTAAGGTGAGAGAGAAGGGGAAGTGTGACTGGGGATGAATAATGTAGAAGCCTTGCAGATAGAACCCCAGAAGTTTACAAGCCTACAGGCTTTTACTTTTTTTTTTCCTTTGACACTTGGACCCATCTTGCTAACTCTTGACACTTGTTGAGTATGCAGGACTAGGTTGGTGATATCAACAGTCTTTCTGCCACCTCCACATTACTTACAAGGTAGAGCAGGGAGCCATTGGGCTTGTGTTGACACCCCTTTGGTGAAGTGGGAAGAGTTGGGAGGCCAGGTTTCCCCTGGGAAGTGGAGGAGTGTTTTTGAGCCTGAATATTGGGACTCCTACTGCCTCTTCTACCGCAGGCATCCCGGCCCCGGATCAACATCCTGTGTTCTACCGTCACAGATGTCCGGACGACCACAGCCCTGGAGATGCCCGACAGGGAGAACACGTTTGTGGTTAAGGTGGAGGGTCCTATGGAATACATCCTGGAGACAGCTGATGCCCTGAGTATGAAGGCCTGGGTGTCTGATCTACAGGATTGCCTGAGTCCAGGGGAGAACACAGACAGCCTGGACCTGTCCTGCCTCAACCACTCAGAGAGTCTGCCCAGCCAAGAGCTGCCCTTGGGACCCAGTGAGAGCAATGAGCATCTGTCTCAGGGGGCGTATGGGGGCCTCTCGGATCGTCCCTCAGCTTCTGTCTCCCCCAGCTCTGCCTCCATTGCTGCCTCCCATTTTGATTCAATGGAACTACTGCCCCCAGAGTTGCCCCCCCGAGTCCCTATTGAAGAGGGGCCCCCAGTGGGGACAACTTACCCCCTCCCAGCCCCTTACCCTCCTCCGGACACTCCAGACACCACAGGTTCGTTCCTGTTCCAGGGGGAATCAGATGGAGGTGATGGGGACCATCCCCTTTCAGAGTACCCTTGGTTCCATGGGACCCTTTCCCGACTCAAAGCTGCCCAGCTAGTGTTGGCAGGGGGTGCTGGCAGCCATGGTGTCTTCCTGGTACGTCAAAGCGAGACAAGGAGGGGGGAGTATGTCCTCACGTTCAACTTCCAGGGCAAGGCCAAGCACCTTCGTCTGTCCCTGAATGAGGATGGTCAGTGCCGGGTGCAGCACCTCTGGTTCCAGACGGTCTTTGACATGCTGGAGCATTTCCGGGTCCACCCCATTCCCTTGGAGTCTGGGGGCTCCAGTGATGTTACCCTCGTCAGCTATGTTGTGGCCTCTCAGAGGCTACAAGGCAGGGAACGGGCAGGGAGCCGAGCTGGGGTGTGTGAGGGAGATCGCTGCTACCCCGACGCCTCTTCCACCCTCATGCCCATCGGAGCGAGTGACTGTGTAACAGAACATCTCCCATGACCCATCCCAATCTCCTGATCCCGATCTGAGGGCAGACCCTGCACACCCTGGGGAAGAGGAGGAGATGGTGGAGGCATCAGAAGCTTCAGAGGAAGAGAAGGCAGGCAGTGGAGGGACCCCAGGAGAGCCGGGCCCTGTGGCTGAGCTTGAGGAGACCACAGCCCTAAACAGGGAGTCCTCAGGGCTAGAGGGGCCTGGTGGGGAGGTGGGAGTGACCCCTTTTGTGCATTTCTTGCAGTTGCAGCCAGGGAATGCCGGGGAGGAAGGGGTCTGCTCCAGAGCCATCAATAACCAGTACTCCTTCCTATAAGGATGAATACCCCCACACAACCAATTCCCCCTTTCTCCCTGGCCCTGGGCCAGGACCCGTGACAGATCCTCCTCAGAGAGGTACAAATGCTTCCCTTGCCCCTATTTGTCTAGGGGGTAGGAATAGTACAGGACAGAGGGACAGAGCCATTAGTAACTTCCCAGAGGACACACTCCAAGCTGCCCCTTTCCCTGGGCAGGGGACTTGGATCCGTTACCTCCTTTTGGGGGTGGGCCAATTGTGCTCAGCCTCGCCCCCTGTGGGCCATTTCTCCAATACCTCTCACAGCTCTCTGTGATGTATTGGGGAGAGGCTGTATCAGCTACATTAAGGATTTTTTTTTTAAGTGAAATGGAGAAACAAATAAAAAGGTTTATCTCAGCTCTCTTCTGGTCATTGGCCCAATGTTTGTATCACAGAATGGAATTGGAGATGGGGACATAATTGGCTCAGGAGGTGCACATAGATATGCTTTTGAGTCCTGGATCAGCCACTTTCTGGGTGACTTTGTGCCTGTCACTTCCCATGTCAGACTAGTCAGCTCTTAAGCCCCTTCCAACTCTTAACGTTCTGGTTCTGGAATTCCAACCCCTGTGGGAGAGCTGTACATAGAGCCAGGGCATGAGGGTGGCTTCTTTTTTCCTAAATAGGTTGTTCTGGGAGAGGTCAAAGGTAATGGCCATTGATCTGGGCTGGAAAGGCCAGACTTGGGAGCAGGGTTTCTGGGTTCTTGAGAAAGGAGGCATCTGGCCTGGGACTGGGAAACTTGTAGTAGCTCTGCTACTCAGTTACATGTGTTACCTTGGAGAAGTCACTCAATTTATTTGGGCCTCAGTTTACTC

>UoN.Pci.T.20889_UoN.Pci.T.20889.2

GTCACTGGTTGGTGGGGGTAGGGGGGGGGAGCGGGAGCCGCCGCTGCTGCCGCCGGAGCAAACCGCGGGGACCGAGATGCAGGTGGGACCGGAACCGGAACCCCCCTTCTCATTCTTCTTTCCCCACCCGAACCCTCCCCCCTTTCCCGGGCCTGGGACTGGAGGGAGGGCCGAAGGTCCTGGAGCGGGGCCGGGCTCGGAAGCTGGGGGCTGGGGGAATATCCCCAGGGCAGCGCACCGCCGGAGGCCGGTGGAGCGGGGCGCAATGGGCGGAGGTGAAGGAGGCCGCCCACGGGCGGAGGGAAAGCCGGCGTTCTCCGGAGCCCCCCACCCCCGGAGGAGCGCTGGGGCTGGGCAAGGGAGCAAGGCTGCCCGCGGCCTCATCACTGCAGGGCCAGGGGGTCGGGGCGAGTGGGCAGCAGCAGCCGGGCTAAAGGAGCTACGGCGTTTGGGGGGGAGCGACCTCAAGGGACCCACACTGGGTGAGGGGTCCCCCCTGTTGTTGGTGTGGAACGTACGAGCTCTGGGGTGGAGGGAGTAGGCATGTCATGCTTGGGTGTCTCCCCCAGCATGCACCAAACCTCCCTCTTTCCCAGGCTCTCTTCATTGGCATTTGAGACTATTTCATGTGTTTCTGTCTCCTCGAGGCAGGGTCCGGGGTAGATGTAAAAGGCGGCTGAAAGGATAGGGAATGAAGGGAGGGTGAAGATGTTGGTCCGGAAGTGACCAGCAGAATTGGCGTGGGTTGCCTGTCAGGAAGGAGTTCTTGAGAAAATAAGAAACACAGGCCAGACTTCTTTAAGGCCACTCTCATTCCAGCCCTTTCCATGGAGGTCTGGCCTTAGGAAATTATTTCCTTTGTTCATTCCAGGTTCTGATGTCTCTCTGTGGAAATTCATTTTGTTGCTGAACCTAGGCCTGCGCAGAATTTCCCATCTTAGGGAACTGGAATAAGGAATCAGGGTTCTTAGGTTCTGTGCCTGACTAGAACTCGAAGCCACCACTTTTGCGACCTTAGGCAGTCATTCAACTTCTGAGTCTGTTTTCTTACCCATAAAATGGGCACAATAGCCCCTGGTTTACCTCTCTAATAGGGTTGCTGTGAAGTAGGAGAATGTGTGTGAAAATGCTTTGAAATCATAAAGTGCTATGTGAATGCTGTTATTGGTCAAGCAGAAATTTTTCTAGAAACTCTTCCTAACCTGGTCAACTAGTGATGCTACAAATAGTGGCTATGAGTCTGGTCTGAAGAAGCACAGGATTAGTGGCCCTCAGTTCTGAGCCGGGTTTTTCCCGGAGTCCAGTTGCTCTGGGCTGTGGGACTAAGGGCCTGTGTGCTCATTTTCACAGCTTGTCTGACCTTTAGCCCCTTTTGCTTTCTTTTATTCTTTGACATTAGAAAATACTTCATCCCAGCCTTCTTATAGTTTCTTTTATTCTTGAGACTGAGGGGAAGAAGTAAGGTAGCAGTACTCAATATTTGGATGGTGAGGAGCTACTGAGGATGGAAGGGGGTGGGTGGGAATGATGCCTTCAAGGCAGTACCCTCATACCATAATGCTTAAAATGCCAATGAGCCTTAGGGAGAGAAGCAAGGTGGCAGTCACCTACCTATTGCTGACCTGTTAGGGACCTTTAGAGTAAGATATTGGATATATTTATTTCTGAAAGAGAAGAGATTGGAAAGGAAGCTTTCTGTCTCTGAAGGGTTTCTTTTTTGGAGGATGTCGACCAGCTTACCTTTAACCCTTCTGGGATCAAAGCCAAAGGATGTGGGCTGAGATTACAGCAGGAAAGATAGAGGCTAGATATCAGAAAAAAACCTACCAGTGCTTGAGAGGTGTGAGAACCTGGAATATGGGAAGGAAGCTAACATATTTTGAAGGACTTTTCTTGAATCTGTCTGAATCAGAGAGGAACAAGGTGAACAGAAGGCAGTGTGGAAAAGTGGATGGCTTCTCAGTGAGGGTGGAACAATTGGGGAATCTCTGGTATTTGGGGAAAGGTCTAGAACTTATTCTCTATAAGGTATAATTCTAATACTGACTCCTATATATGTGCCTCTTTTTCCCTTCCACAGTTGCTGCAGATGACCCTGAGTCTTCTGGCCTCCCCCCCCACTTCCTTCCCTCCCCTGGGTCCCCCACTCTCCATTTCATCTGGCTCATCATGAATGGTGCCCCTTCCCCTGAGGATGGGACTTCCCCTTCTCCTCCCCCTCTGCCACCACCACTTCCACCACCTCCTCCCCCAAGTTGGCGGGAGTTCTGTGAATCCCACGCTCGGGCAGCAGCCCTAGACTTTGCCCGACGCTTCCGCCTCTACTTGGCTTCCCATCCCCAGTATGCAGGGCCTGGGGCTGAGGCAGCCTTCTCCCGACGTTTTGCTGAGCTCTTCTTGCAGCACTTTGAGGCTGAGGTGGCCCGGGCCTCAGGTTCCCTTTTATCATCTGGTCCGATCCCCCTGAGTCCCAGTACGGAAACCCCATCACGTGATCTTTCCCTGGAGAGCTGCAGGAACAGTGGGCCTCTGACTGTGTTGGGTCCTTCTCGATCCTCTGAAGACCTGGCTGGCCCTCTCCCTTCTTCAATCTCTTCATCCTCCTCAAAGCCTAAGCTGAAGAAACGTTTCTCCCTCCGCTCTGTGGGCCGATCAGTGCGAGGCTCGGTCCGAGGGATCTTGCAGTGGCGGGGAGCTGGTGATCCCCCCTCTCCAGTGGGACCCTTGGAGACCTCTGCCTCTGGACCACCTGTCCTGGGTGGAAACAGCAACTCCAACTCCTCTGGTGGGGGTGGTAGCACCAATGTGGGGCCAACTAGCGGTGGGGCATCCCCTGGGGAAAGATGGACACACCGCTTTGAGAGGCTGAGACTAAGTAGGGGAGGGGGACCCTTAAAGGATGGGGCAGGGACAGTGCAAAGGGAGGGGCTACTGAGCTTCATGGGAACAGAGGAGGCAGCTGTTGGTGGAGGAGAAGGTCGAGGGGGAGGAGGAACTGGAACCTCATCTGGGGGTGGGGGACCACCAAGGTGGCAGAAGTGTCGCCTGCTGCTTCGGAGTGAGGGGGAAGGAGGAGGAAGTCGGCTGGAGTTCTATATCCCACCTAAGGTGAGAGAGAAGGGGAAGTGTGACTGGGGATGAATAATGTAGAAGCCTTGCAGATAGAACCCCAGAAGTTTACAAGCCTACAGGCTTTTACTTTTTTTTTTCCTTTGACACTTGGACCCATCTTGCTAACTCTTGACACTTGTTGAGTATGCAGGACTAGGTTGGTGATATCAACAGTCTTTCTGCCACCTCCACATTACTTACAAGGTAGAGCAGGGAGCCATTGGGCTTGTGTTGACACCCCTTTGGTGAAGTGGGAAGAGTTGGGAGGCCAGGTTTCCCCTGGGAAGTGGAGGAGTGTTTTTGAGCCTGAATATTGGGACTCCTACTGCCTCTTCTACCGCAGGCATCCCGGCCCCGGATCAACATCCTGTGTTCTACCGTCACAGATGTCCGGACGACCACAGCCCTGGAGATGCCCGACAGGGAGAACACGTTTGTGGTTAAGGTGGAGGGTCCTATGGAATACATCCTGGAGACAGCTGATGCCCTGAGTATGAAGGCCTGGGTGTCTGATCTACAGGATTGCCTGAGTCCAGGGGAGAACACAGACAGCCTGGACCTGTCCTGCCTCAACCACTCAGAGAGTCTGCCCAGCCAAGAGCTGCCCTTGGGACCCAGTGAGAGCAATGAGCATCTGTCTCAGGGGGCGTATGGGGGCCTCTCGGATCGTCCCTCAGCTTCTGTCTCCCCCAGCTCTGCCTCCATTGCTGCCTCCCATTTTGATTCAATGGAACTACTGCCCCCAGAGTTGCCCCCCCGAGTCCCTATTGAAGAGGGGCCCCCAGTGGGGACAACTTACCCCCTCCCAGCCCCTTACCCTCCTCCGGACACTCCAGACACCACAGGTTCGTTCCTGTTCCAGGGGGAATCAGATGGAGGTGATGGGGACCATCCCCTTTCAGAGTACCCTTGGTTCCATGGGACCCTTTCCCGACTCAAAGCTGCCCAGCTAGTGTTGGCAGGGGGTGCTGGCAGCCATGGTGTCTTCCTGGTACGTCAAAGCGAGACAAGGAGGGGGGAGTATGTCCTCACGTTCAACTTCCAGGGCAAGGCCAAGCACCTTCGTCTGTCCCTGAATGAGGATGGTCAGTGCCGGGTGCAGCACCTCTGGTTCCAGACGGTCTTTGACATGCTGGAGCATTTCCGGGTCCACCCCATTCCCTTGGAGTCTGGGGGCTCCAGTGATGTTACCCTCGTCAGCTATGTTGTGGCCTCTCAGAGGCTACAAGGCAGGGAACGGGCAGGGAGCCGAGCTGGGGTGTGTGAGGGAGATCGCTGCTACCCCGACGCCTCTTCCACCCTCATGCCCATCGGAGCGAGTGACTGTGTAACAGAACATCTCCCATGACCCATCCCAATCTCCTGATCCCGATCTGAGGGCAGACCCTGCACACCCTGGGGAAGAGGAGGAGATGGTGGAGGCATCAGAAGCTTCAGAGGAAGAGAAGGCAGGCAGTGGAGGGACCCCAGGAGAGCCGGGCCCTGTGGCTGAGCTTGAGGAGACCACAGCCCTAAACAGGGAGTCCTCAGGGCTAGAGGGGCCTGGTGGGGAGGTGGGAGTGACCCCTTTTGTGCATTTCTTGCAGTTGCAGCCAGGGAATGCCGGGGAGGAAGGGGTCTGCTCCAGAGCCATCAATAACCAGTACTCCTTCCTATAAGGATGAATACCCCCACACAACCAATTCCCCCTTTCTCCCTGGCCCTGGGCCAGGACCCGTGACAGATCCTCCTCAGAGAGGTACAAATGCTTCCCTTGCCCCTATTTGTCTAGGGGGTAGGAATAGTACAGGACAGAGGGACAGAGCCATTAGTAACTTCCCAGAGGACACACTCCAAGCTGCCCCTTTCCCTGGGCAGGGGACTTGGATCCGTTACCTCCTTTTGGGGGTGGGCCAATTGTGCTCAGCCTCGCCCCCTGTGGGCCATTTCTCCAATACCTCTCACAGCTCTCTGTGATGTATTGGGGAGAGGCTGTATCAGCTACATTAAGGATTTTTTTTTTAAGTGAAATGGAGAAACAAATAAAAAGGTTTATCTCAGCTCTCTTCTGGTCATTGGCCCAATGTTTGTATCACAGAATGGAATTGGAGATGGGGACATAATTGGCTCAGGAGGTGCACATAGATATGCTTTTGAGTCCTGGATCAGCCACTTTCTGGGTGACTTTGTGCCTGTCACTTCCCATGTCAGACTAGTCAGCTCTTAAGCCCCTTCCAACTCTTAACGTTCTGGTTCTGGAATTCCAACCCCTGTGGGAGAGCTGTACATAGAGCCAGGGCATGAGGGTGGCTTCTTTTTTCCTAAATAGGTTGTTCTGGGAGAGGTCAAAGGTAATGGCCATTGATCTGGGCTGGAAAGGCCAGACTTGGGAGCAGGGTTTCTGGGTTCTTGAGAAAGGAGGCATCTGGCCTGGGACTGGGAAACTTGTAGTAGCTCTGCTACTCAGTTACATGTGTTACCTTGGAGAAGTCACTCAATTTATTTGGGCCTCAGTTTACTC

>UoN.Pci.T.20889_UoN.Pci.T.20889.3

GTCACTGGTTGGTGGGGGTAGGGGGGGGGAGCGGGAGCCGCCGCTGCTGCCGCCGGAGCAAACCGCGGGGACCGAGATGCAGGTGGGACCGGAACCGGAACCCCCCTTCTCATTCTTCTTTCCCCACCCGAACCCTCCCCCCTTTCCCGGGCCTGGGACTGGAGGGAGGGCCGAAGGTCCTGGAGCGGGGCCGGGCTCGGAAGCTGGGGGCTGGGGGAATATCCCCAGGGCAGCGCACCGCCGGAGGCCGGTGGAGCGGGGCGCAATGGGCGGAGGTGAAGGAGGCCGCCCACGGGCGGAGGGAAAGCCGGCGTTCTCCGGAGCCCCCCACCCCCGGAGGAGCGCTGGGGCTGGGCAAGGGAGCAAGGCTGCCCGCGGCCTCATCACTGCAGGGCCAGGGGGTCGGGGCGAGTGGGCAGCAGCAGCCGGGCTAAAGGAGCTACGGCGTTTGGGGGGGAGCGACCTCAAGGGACCCACACTGGGTGAGGGGTCCCCCCTGTTGTTGGTGTGGAACGTACGAGCTCTGGGGTGGAGGGAGTAGGCATGTCATGCTTGGGTGTCTCCCCCAGCATGCACCAAACCTCCCTCTTTCCCAGGCTCTCTTCATTGGCATTTGAGACTATTTCATGTGTTTCTGTCTCCTCGAGGCAGGGTCCGGGGTAGATGTAAAAGGCGGCTGAAAGGATAGGGAATGAAGGGAGGGTGAAGATGTTGGTCCGGAAGTGACCAGCAGAATTGGCGTGGGTTGCCTGTCAGGAAGGAGTTCTTGAGAAAATAAGAAACACAGGCCAGACTTCTTTAAGGCCACTCTCATTCCAGCCCTTTCCATGGAGGTCTGGCCTTAGGAAATTATTTCCTTTGTTCATTCCAGGTTCTGATGTCTCTCTGTGGAAATTCATTTTGTTGCTGAACCTAGGCCTGCGCAGAATTTCCCATCTTAGGGAACTGGAATAAGGAATCAGGGTTCTTAGGTTCTGTGCCTGACTAGAACTCGAAGCCACCACTTTTGCGACCTTAGGCAGTCATTCAACTTCTGAGTCTGTTTTCTTACCCATAAAATGGGCACAATAGCCCCTGGTTTACCTCTCTAATAGGGTTGCTGTGAAGTAGGAGAATGTGTGTGAAAATGCTTTGAAATCATAAAGTGCTATGTGAATGCTGTTATTGGTCAAGCAGAAATTTTTCTAGAAACTCTTCCTAACCTGGTCAACTAGTGATGCTACAAATAGTGGCTATGAGTCTGGTCTGAAGAAGCACAGGATTAGTGGCCCTCAGTTCTGAGCCGGGTTTTTCCCGGAGTCCAGTTGCTCTGGGCTGTGGGACTAAGGGCCTGTGTGCTCATTTTCACAGCTTGTCTGACCTTTAGCCCCTTTTGCTTTCTTTTATTCTTTGACATTAGAAAATACTTCATCCCAGCCTTCTTATAGTTTCTTTTATTCTTGAGACTGAGGGGAAGAAGTAAGGTAGCAGTACTCAATATTTGGATGGTGAGGAGCTACTGAGGATGGAAGGGGGTGGGTGGGAATGATGCCTTCAAGGCAGTACCCTCATACCATAATGCTTAAAATGCCAATGAGCCTTAGGGAGAGAAGCAAGGTGGCAGTCACCTACCTATTGCTGACCTGTTAGGGACCTTTAGAGTAAGATATTGGATATATTTATTTCTGAAAGAGAAGAGATTGGAAAGGAAGCTTTCTGTCTCTGAAGGGTTTCTTTTTTGGAGGATGTCGACCAGCTTACCTTTAACCCTTCTGGGATCAAAGCCAAAGGATGTGGGCTGAGATTACAGCAGGAAAGATAGAGGCTAGATATCAGAAAAAAACCTACCAGTGCTTGAGAGGTGTGAGAACCTGGAATATGGGAAGGAAGCTAACATATTTTGAAGGACTTTTCTTGAATCTGTCTGAATCAGAGAGGAACAAGGTGAACAGAAGGCAGTGTGGAAAAGTGGATGGCTTCTCAGTGAGGGTGGAACAATTGGGGAATCTCTGGTATTTGGGGAAAGGTCTAGAACTTATTCTCTATAAGGTATAATTCTAATACTGACTCCTATATATGTGCCTCTTTTTCCCTTCCACAGTTGCTGCAGATGACCCTGAGTCTTCTGGCCTCCCCCCCCACTTCCTTCCCTCCCCTGGGTCCCCCACTCTCCATTTCATCTGGCTCATCATGAATGGTGCCCCTTCCCCTGAGGATGGGACTTCCCCTTCTCCTCCCCCTCTGCCACCACCACTTCCACCACCTCCTCCCCCAAGTTGGCGGGAGTTCTGTGAATCCCACGCTCGGGCAGCAGCCCTAGACTTTGCCCGACGCTTCCGCCTCTACTTGGCTTCCCATCCCCAGTATGCAGGGCCTGGGGCTGAGGCAGCCTTCTCCCGACGTTTTGCTGAGCTCTTCTTGCAGCACTTTGAGGCTGAGGTGGCCCGGGCCTCAGGTTCCCTTTTATCATCTGGTCCGATCCCCCTGAGTCCCAGTACGGAAACCCCATCACGTGATCTTTCCCTGGAGAGCTGCAGGAACAGTGGGCCTCTGACTGTGTTGGGTCCTTCTCGATCCTCTGAAGACCTGGCTGGCCCTCTCCCTTCTTCAATCTCTTCATCCTCCTCAAAGCCTAAGCTGAAGAAACGTTTCTCCCTCCGCTCTGTGGGCCGATCAGTGCGAGGCTCGGTCCGAGGGATCTTGCAGTGGCGGGGAGCTGGTGATCCCCCCTCTCCAGTGGGACCCTTGGAGACCTCTGCCTCTGGACCACCTGTCCTGGGTGGAAACAGCAACTCCAACTCCTCTGGTGGGGGTGGTAGCACCAATGTGGGGCCAACTAGCGGTGGGGCATCCCCTGGGGAAAGATGGACACACCGCTTTGAGAGGCTGAGACTAAGTAGGGGAGGGGGACCCTTAAAGGATGGGGCAGGGACAGTGCAAAGGGAGGGGCTACTGAGCTTCATGGGAACAGAGGAGGCAGCTGTTGGTGGAGGAGAAGGTCGAGGGGGAGGAGGAACTGGAACCTCATCTGGGGGTGGGGGACCACCAAGGTGGCAGAAGTGTCGCCTGCTGCTTCGGAGTGAGGGGGAAGGAGGAGGAAGTCGGCTGGAGTTCTATATCCCACCTAAGGTGAGAGAGAAGGGGAAGTGTGACTGGGGATGAATAATGTAGAAGCCTTGCAGATAGAACCCCAGAAGTTTACAAGCCTACAGGCTTTTACTTTTTTTTTTCCTTTGACACTTGGACCCATCTTGCTAACTCTTGACACTTGTTGAGTATGCAGGACTAGGTTGGTGATATCAACAGTCTTTCTGCCACCTCCACATTACTTACAAGGTAGAGCAGGGAGCCATTGGGCTTGTGTTGACACCCCTTTGGTGAAGTGGGAAGAGTTGGGAGGCCAGGTTTCCCCTGGGAAGTGGAGGAGTGTTTTTGAGCCTGAATATTGGGACTCCTACTGCCTCTTCTACCGCAGGCATCCCGGCCCCGGATCAACATCCTGTGTTCTACCGTCACAGATGTCCGGACGACCACAGCCCTGGAGATGCCCGACAGGGAGAACACGTTTGTGGTTAAGGTGGAGGGTCCTATGGAATACATCCTGGAGACAGCTGATGCCCTGAGTATGAAGGCCTGGGTGTCTGATCTACAGGATTGCCTGAGTCCAGGGGAGAACACAGACAGCCTGGACCTGTCCTGCCTCAACCACTCAGAGAGTCTGCCCAGCCAAGAGCTGCCCTTGGGACCCAGTGAGAGCAATGAGCATCTGTCTCAGGGGGCGTATGGGGGCCTCTCGGATCGTCCCTCAGCTTCTGTCTCCCCCAGCTCTGCCTCCATTGCTGCCTCCCATTTTGATTCAATGGAACTACTGCCCCCAGAGTTGCCCCCCCGAGTCCCTATTGAAGAGGGGCCCCCAGTGGGGACAACTTACCCCCTCCCAGCCCCTTACCCTCCTCCGGACACTCCAGACACCACAGGTTCGTTCCTGTTCCAGGGGGAATCAGATGGAGGTGATGGGGACCATCCCCTTTCAGAGTACCCTTGGTTCCATGGGACCCTTTCCCGACTCAAAGCTGCCCAGCTAGTGTTGGCAGGGGGTGCTGGCAGCCATGGTGTCTTCCTGGTACGTCAAAGCGAGACAAGGAGGGGGGAGTATGTCCTCACGTTCAACTTCCAGGGCAAGGCCAAGCACCTTCGTCTGTCCCTGAATGAGGATGGTCAGTGCCGGGTGCAGCACCTCTGGTTCCAGACGGTCTTTGACATGCTGGAGCATTTCCGGGTCCACCCCATTCCCTTGGAGTCTGGGGGCTCCAGTGATGTTACCCTCGTCAGCTATGTTGTGGCCTCTCAGAGGCTACAAGGCAGGGAACGGGCAGGGAGCCGAGCTGGGGTGTGTGAGGGAGATCGCTGCTACCCCGACGCCTCTTCCACCCTCATGCCCATCGGAGCGAGTGACTGTGTAACAGAACATCTCCCATGACCCATCCCAATCTCCTGATCCCGATCTGAGGGCAGACCCTGCACACCCTGGGGAAGAGGAGGAGATGGTGGAGGCATCAGAAGCTTCAGAGGAAGAGAAGGCAGGCAGTGGAGGGACCCCAGGAGAGCCGGGCCCTGTGGCTGAGCTTGAGGAGACCACAGCCCTAAACAGGGAGTCCTCAGGGCTAGAGGGGCCTGGTGGGGAGGTGGGAGTGACCCCTTTTGTGCATTTCTTGCAGTTGCAGCCAGGGAATGCCGGGGAGGAAGGGGTCTGCTCCAGAGCCATCAATAACCAGTACTCCTTCCTATAAGGATGAATACCCCCACACAACCAATTCCCCCTTTCTCCCTGGCCCTGGGCCAGGACCCGTGACAGATCCTCCTCAGAGAGGTACAAATGCTTCCCTTGCCCCTATTTGTCTAGGGGGTAGGAATAGTACAGGACAGAGGGACAGAGCCATTAGTAACTTCCCAGAGGACACACTCCAAGCTGCCCCTTTCCCTGGGCAGGGGACTTGGATCCGTTACCTCCTTTTGGGGGTGGGCCAATTGTGCTCAGCCTCGCCCCCTGTGGGCCATTTCTCCAATACCTCTCACAGCTCTCTGTGATGTATTGGGGAGAGGCTGTATCAGCTACATTAAGGATTTTTTTTTTAAGTGAAATGGAGAAACAAATAAAAAGGTTTATCTCAGCTCTCTTCTGGTCATTGGCCCAATGTTTGTATCACAGAATGGAATTGGAGATGGGGACATAATTGGCTCAGGAGGTGCACATAGATATGCTTTTGAGTCCTGGATCAGCCACTTTCTGGGTGACTTTGTGCCTGTCACTTCCCATGTCAGACTAGTCAGCTCTTAAGCCCCTTCCAACTCTTAACGTTCTGGTTCTGGAATTCCAAC

>UoN.Pci.T.20889_UoN.Pci.T.20889.4

GTCACTGGTTGGTGGGGGTAGGGGGGGGGAGCGGGAGCCGCCGCTGCTGCCGCCGGAGCAAACCGCGGGGACCGAGATGCAGGTGGGACCGGAACCGGAACCCCCCTTCTCATTCTTCTTTCCCCACCCGAACCCTCCCCCCTTTCCCGGGCCTGGGACTGGAGGGAGGGCCGAAGGTCCTGGAGCGGGGCCGGGCTCGGAAGCTGGGGGCTGGGGGAATATCCCCAGGGCAGCGCACCGCCGGAGGCCGGTGGAGCGGGGCGCAATGGGCGGAGGTGAAGGAGGCCGCCCACGGGCGGAGGGAAAGCCGGCGTTCTCCGGAGCCCCCCACCCCCGGAGGAGCGCTGGGGCTGGGCAAGGGAGCAAGGCTGCCCGCGGCCTCATCACTGCAGGGCCAGGGGGTCGGGGCGAGTGGGCAGCAGCAGCCGGGCTAAAGGAGCTACGGCGTTTGGGGGGGAGCGACCTCAAGGGACCCACACTGGGTGAGGGGTCCCCCCTGTTGTTGGTGTGGAACGTACGAGCTCTGGGGTGGAGGGAGTAGGCATGTCATGCTTGGGTGTCTCCCCCAGCATGCACCAAACCTCCCTCTTTCCCAGGCTCTCTTCATTGGCATTTGAGACTATTTCATGTGTTTCTGTCTCCTCGAGGCAGGGTCCGGGGTAGATGTAAAAGGCGGCTGAAAGGATAGGGAATGAAGGGAGGGTGAAGATGTTGGTCCGGAAGTGACCAGCAGAATTGGCGTGGGTTGCCTGTCAGGAAGGAGTTCTTGAGAAAATAAGAAACACAGGCCAGACTTCTTTAAGGCCACTCTCATTCCAGCCCTTTCCATGGAGGTCTGGCCTTAGGAAATTATTTCCTTTGTTCATTCCAGGTTCTGATGTCTCTCTGTGGAAATTCATTTTGTTGCTGAACCTAGGCCTGCGCAGAATTTCCCATCTTAGGGAACTGGAATAAGGAATCAGGGTTCTTAGGTTCTGTGCCTGACTAGAACTCGAAGCCACCACTTTTGCGACCTTAGGCAGTCATTCAACTTCTGAGTCTGTTTTCTTACCCATAAAATGGGCACAATAGCCCCTGGTTTACCTCTCTAATAGGGTTGCTGTGAAGTAGGAGAATGTGTGTGAAAATGCTTTGAAATCATAAAGTGCTATGTGAATGCTGTTATTGGTCAAGCAGAAATTTTTCTAGAAACTCTTCCTAACCTGGTCAACTAGTGATGCTACAAATAGTGGCTATGAGTCTGGTCTGAAGAAGCACAGGATTAGTGGCCCTCAGTTCTGAGCCGGGTTTTTCCCGGAGTCCAGTTGCTCTGGGCTGTGGGACTAAGGGCCTGTGTGCTCATTTTCACAGCTTGTCTGACCTTTAGCCCCTTTTGCTTTCTTTTATTCTTTGACATTAGAAAATACTTCATCCCAGCCTTCTTATAGTTTCTTTTATTCTTGAGACTGAGGGGAAGAAGTAAGGTAGCAGTACTCAATATTTGGATGGTGAGGAGCTACTGAGGATGGAAGGGGGTGGGTGGGAATGATGCCTTCAAGGCAGTACCCTCATACCATAATGCTTAAAATGCCAATGAGCCTTAGGGAGAGAAGCAAGGTGGCAGTCACCTACCTATTGCTGACCTGTTAGGGACCTTTAGAGTAAGATATTGGATATATTTATTTCTGAAAGAGAAGAGATTGGAAAGGAAGCTTTCTGTCTCTGAAGGGTTTCTTTTTTGGAGGATGTCGACCAGCTTACCTTTAACCCTTCTGGGATCAAAGCCAAAGGATGTGGGCTGAGATTACAGCAGGAAAGATAGAGGCTAGATATCAGAAAAAAACCTACCAGTGCTTGAGAGGTGTGAGAACCTGGAATATGGGAAGGAAGCTAACATATTTTGAAGGACTTTTCTTGAATCTGTCTGAATCAGAGAGGAACAAGGTGAACAGAAGGCAGTGTGGAAAAGTGGATGGCTTCTCAGTGAGGGTGGAACAATTGGGGAATCTCTGGTATTTGGGGAAAGGTCTAGAACTTATTCTCTATAAGGTATAATTCTAATACTGACTCCTATATATGTGCCTCTTTTTCCCTTCCACAGTTGCTGCAGATGACCCTGAGTCTTCTGGCCTCCCCCCCCACTTCCTTCCCTCCCCTGGGTCCCCCACTCTCCATTTCATCTGGCTCATCATGAATGGTGCCCCTTCCCCTGAGGATGGGACTTCCCCTTCTCCTCCCCCTCTGCCACCACCACTTCCACCACCTCCTCCCCCAAGTTGGCGGGAGTTCTGTGAATCCCACGCTCGGGCAGCAGCCCTAGACTTTGCCCGACGCTTCCGCCTCTACTTGGCTTCCCATCCCCAGTATGCAGGGCCTGGGGCTGAGGCAGCCTTCTCCCGACGTTTTGCTGAGCTCTTCTTGCAGCACTTTGAGGCTGAGGTGGCCCGGGCCTCAGGTTCCCTTTTATCATCTGGTCCGATCCCCCTGAGTCCCAGTACGGAAACCCCATCACGTGATCTTTCCCTGGAGAGCTGCAGGAACAGTGGGCCTCTGACTGTGTTGGGTCCTTCTCGATCCTCTGAAGACCTGGCTGGCCCTCTCCCTTCTTCAATCTCTTCATCCTCCTCAAAGCCTAAGCTGAAGAAACGTTTCTCCCTCCGCTCTGTGGGCCGATCAGTGCGAGGCTCGGTCCGAGGGATCTTGCAGTGGCGGGGAGCTGGTGATCCCCCCTCTCCAGTGGGACCCTTGGAGACCTCTGCCTCTGGACCACCTGTCCTGGGTGGAAACAGCAACTCCAACTCCTCTGGTGGGGGTGGTAGCACCAATGTGGGGCCAACTAGCGGTGGGGCATCCCCTGGGGAAAGATGGACACACCGCTTTGAGAGGCTGAGACTAAGTAGGGGAGGGGGACCCTTAAAGGATGGGGCAGGGACAGTGCAAAGGGAGGGGCTACTGAGCTTCATGGGAACAGAGGAGGCAGCTGTTGGTGGAGGAGAAGGTCGAGGGGGAGGAGGAACTGGAACCTCATCTGGGGGTGGGGGACCACCAAGGTGGCAGAAGTGTCGCCTGCTGCTTCGGAGTGAGGGGGAAGGAGGAGGAAGTCGGCTGGAGTTCTATATCCCACCTAAGGTGAGAGAGAAGGGGAAGTGTGACTGGGGATGAATAATGTAGAAGCCTTGCAGATAGAACCCCAGAAGTTTACAAGCCTACAGGCTTTTACTTTTTTTTTTCCTTTGACACTTGGACCCATCTTGCTAACTCTTGACACTTGTTGAGTATGCAGGACTAGGTTGGTGATATCAACAGTCTTTCTGCCACCTCCACATTACTTACAAGGTAGAGCAGGGAGCCATTGGGCTTGTGTTGACACCCCTTTGGTGAAGTGGGAAGAGTTGGGAGGCCAGGTTTCCCCTGGGAAGTGGAGGAGTGTTTTTGAGCCTGAATATTGGGACTCCTACTGCCTCTTCTACCGCAGGCATCCCGGCCCCGGATCAACATCCTGTGTTCTACCGTCACAGATGTCCGGACGACCACAGCCCTGGAGATGCCCGACAGGGAGAACACGTTTGTGGTTAAGGTGGAGGGTCCTATGGAATACATCCTGGAGACAGCTGATGCCCTGAGTATGAAGGCCTGGGTGTCTGATCTACAGGATTGCCTGAGTCCAGGGGAGAACACAGACAGCCTGGACCTGTCCTGCCTCAACCACTCAGAGAGTCTGCCCAGCCAAGAGCTGCCCTTGGGACCCAGTGAGAGCAATGAGCATCTGTCTCAGGGGGCGTATGGGGGCCTCTCGGATCGTCCCTCAGCTTCTGTCTCCCCCAGCTCTGCCTCCATTGCTGCCTCCCATTTTGATTCAATGGAACTACTGCCCCCAGAGTTGCCCCCCCGAGTCCCTATTGAAGAGGGGCCCCCAGTGGGGACAACTTACCCCCTCCCAGCCCCTTACCCTCCTCCGGACACTCCAGACACCACAGGTTCGTTCCTGTTCCAGGGGGAATCAGATGGAGGTGATGGGGACCATCCCCTTTCAGAGTACCCTTGGTTCCATGGGACCCTTTCCCGACTCAAAGCTGCCCAGCTAGTGTTGGCAGGGGGTGCTGGCAGCCATGGTGTCTTCCTGGTACGTCAAAGCGAGACAAGGAGGGGGGAGTATGTCCTCACGTTCAACTTCCAGGGCAAGGCCAAGCACCTTCGTCTGTCCCTGAATGAGGATGGTCAGTGCCGGGTGCAGCACCTCTGGTTCCAGACGGTCTTTGACATGCTGGAGCATTTCCGGGTCCACCCCATTCCCTTGGAGTCTGGGGGCTCCAGTGATGTTACCCTCGTCAGCTATGTTGTGGCCTCTCAGAGGCTACAAGGCAGGGAACGGGCAGGGAGCCGAGCTGGGGTGTGTGAGGGAGATCGCTGCTACCCCGACGCCTCTTCCACCCTCATGCCCATCGGAGCGAGTGACTGTGTAACAGAACATCTCCCATGACCCATCCCAATCTCCTGATCCCGATCTGAGGGCAGACCCTGCACACCCTGGGGAAGAGGAGGAGATGGTGGAGGCATCAGAAGCTTCAGAGGAAGAGAAGGCAGGCAGTGGAGGGACCCCAGGAGAGCCGGGCCCTGTGGCTGAGCTTGAGGAGACCACAGCCCTAAACAGGGAGTCCTCAGGGCTAGAGGGGCCTGGTGGGGAGGTGGGAGTGACCCCTTTTGTGCATTTCTTGCAGTTGCAGCCAGGGAATGCCGGGGAGGAAGGGGTCTGCTCCAGAGCCATCAATAACCAGTACTCCTTCCTATAAGGATGAATACCCCCACACAACCAATTCCCCCTTTCTCCCTGGCCCTGGGCCAGGACCCGTGACAGATCCTCCTCAGAGAGGTACAAATGCTTCCCTTGCCCCTATTTGTCTAGGGGGTAGGAATAGTACAGGACAGAGGGACAGAGCCATTAGTAACTTCCCAGAGGACACACTCCAAGCTGCCCCTTTCCCTGGGCAGGGGACTTGGATCCGTTACCTCCTTTTGGGGGTGGGCCAATTGTGCTCAGCCTCGCCCCCTGTGGGCCATTTCTCCAATACCTCTCACAGCTCTCTGTGATGTATTGGGGAGAGGCTGTATCAGCTACATTAAGGATTTTTTTTTTAAGTGAAATGGAGAAACAAATAAAAAGGTTTATCTCAGCTCTCTTCTGGTCATTGGCCCAATGTTTGTATCACAGAATGGAATTGGAGATGGGGACATAATTGGCTCAGGAGGTGCACATAGATATGCTTTTGAGTCCTGGATCAGCCACTTTCTGGGTGACTTTGTGCCTGTCACTTCCCATGTCAGACTAGTCAGCTCTTAAGCCCCTTCCAACTCTTAACGTTCTGGTTCTGGAATTCCAACCCCTGTGGGAGAGCTGTACATAGAGCCAGGGCATGAGGGTGGCTTCTTTTTTCCTAAATAGGTTGTTCTGGGAGAGGTCAAAGGTAATGGCCATTGATCTGGGCTGGAAAGGCCAGACTTGGGAGCAGGGTTTCTGGGTTCTTGAGAAAGGAGGCATCTGGCCTGGGACTGGGAAACTTGTAGTAGCTCTGCTACTCAGTTACATGTGTTACCTTGGAGAAGTCACTCAATTTATTTGGGCCTCAGTTTACTCCTCTGTAAATGAGGGG

>UoN.Pci.T.20889_UoN.Pci.T.20889.5

GTCACTGGTTGGTGGGGGTAGGGGGGGGGAGCGGGAGCCGCCGCTGCTGCCGCCGGAGCAAACCGCGGGGACCGAGATGCAGGTGGGACCGGAACCGGAACCCCCCTTCTCATTCTTCTTTCCCCACCCGAACCCTCCCCCCTTTCCCGGGCCTGGGACTGGAGGGAGGGCCGAAGGTCCTGGAGCGGGGCCGGGCTCGGAAGCTGGGGGCTGGGGGAATATCCCCAGGGCAGCGCACCGCCGGAGGCCGGTGGAGCGGGGCGCAATGGGCGGAGGTGAAGGAGGCCGCCCACGGGCGGAGGGAAAGCCGGCGTTCTCCGGAGCCCCCCACCCCCGGAGGAGCGCTGGGGCTGGGCAAGGGAGCAAGGCTGCCCGCGGCCTCATCACTGCAGGGCCAGGGGGTCGGGGCGAGTGGGCAGCAGCAGCCGGGCTAAAGGAGCTACGGCGTTTGGGGGGGAGCGACCTCAAGGGACCCACACTGGGTGAGGGGTCCCCCCTGTTGTTGGTGTGGAACGTACGAGCTCTGGGGTGGAGGGAGTAGGCATGTCATGCTTGGGTGTCTCCCCCAGCATGCACCAAACCTCCCTCTTTCCCAGGCTCTCTTCATTGGCATTTGAGACTATTTCATGTGTTTCTGTCTCCTCGAGGCAGGGTCCGGGGTAGATGTAAAAGGCGGCTGAAAGGATAGGGAATGAAGGGAGGGTGAAGATGTTGGTCCGGAAGTGACCAGCAGAATTGGCGTGGGTTGCCTGTCAGGAAGGAGTTCTTGAGAAAATAAGAAACACAGGCCAGACTTCTTTAAGGCCACTCTCATTCCAGCCCTTTCCATGGAGGTCTGGCCTTAGGAAATTATTTCCTTTGTTCATTCCAGGTTCTGATGTCTCTCTGTGGAAATTCATTTTGTTGCTGAACCTAGGCCTGCGCAGAATTTCCCATCTTAGGGAACTGGAATAAGGAATCAGGGTTCTTAGGTTCTGTGCCTGACTAGAACTCGAAGCCACCACTTTTGCGACCTTAGGCAGTCATTCAACTTCTGAGTCTGTTTTCTTACCCATAAAATGGGCACAATAGCCCCTGGTTTACCTCTCTAATAGGGTTGCTGTGAAGTAGGAGAATGTGTGTGAAAATGCTTTGAAATCATAAAGTGCTATGTGAATGCTGTTATTGGTCAAGCAGAAATTTTTCTAGAAACTCTTCCTAACCTGGTCAACTAGTGATGCTACAAATAGTGGCTATGAGTCTGGTCTGAAGAAGCACAGGATTAGTGGCCCTCAGTTCTGAGCCGGGTTTTTCCCGGAGTCCAGTTGCTCTGGGCTGTGGGACTAAGGGCCTGTGTGCTCATTTTCACAGCTTGTCTGACCTTTAGCCCCTTTTGCTTTCTTTTATTCTTTGACATTAGAAAATACTTCATCCCAGCCTTCTTATAGTTTCTTTTATTCTTGAGACTGAGGGGAAGAAGTAAGGTAGCAGTACTCAATATTTGGATGGTGAGGAGCTACTGAGGATGGAAGGGGGTGGGTGGGAATGATGCCTTCAAGGCAGTACCCTCATACCATAATGCTTAAAATGCCAATGAGCCTTAGGGAGAGAAGCAAGGTGGCAGTCACCTACCTATTGCTGACCTGTTAGGGACCTTTAGAGTAAGATATTGGATATATTTATTTCTGAAAGAGAAGAGATTGGAAAGGAAGCTTTCTGTCTCTGAAGGGTTTCTTTTTTGGAGGATGTCGACCAGCTTACCTTTAACCCTTCTGGGATCAAAGCCAAAGGATGTGGGCTGAGATTACAGCAGGAAAGATAGAGGCTAGATATCAGAAAAAAACCTACCAGTGCTTGAGAGGTGTGAGAACCTGGAATATGGGAAGGAAGCTAACATATTTTGAAGGACTTTTCTTGAATCTGTCTGAATCAGAGAGGAACAAGGTGAACAGAAGGCAGTGTGGAAAAGTGGATGGCTTCTCAGTGAGGGTGGAACAATTGGGGAATCTCTGGTATTTGGGGAAAGGTCTAGAACTTATTCTCTATAAGGTATAATTCTAATACTGACTCCTATATATGTGCCTCTTTTTCCCTTCCACAGTTGCTGCAGATGACCCTGAGTCTTCTGGCCTCCCCCCCCACTTCCTTCCCTCCCCTGGGTCCCCCACTCTCCATTTCATCTGGCTCATCATGAATGGTGCCCCTTCCCCTGAGGATGGGACTTCCCCTTCTCCTCCCCCTCTGCCACCACCACTTCCACCACCTCCTCCCCCAAGTTGGCGGGAGTTCTGTGAATCCCACGCTCGGGCAGCAGCCCTAGACTTTGCCCGACGCTTCCGCCTCTACTTGGCTTCCCATCCCCAGTATGCAGGGCCTGGGGCTGAGGCAGCCTTCTCCCGACGTTTTGCTGAGCTCTTCTTGCAGCACTTTGAGGCTGAGGTGGCCCGGGCCTCAGGTTCCCTTTTATCATCTGGTCCGATCCCCCTGAGTCCCAGTACGGAAACCCCATCACGTGATCTTTCCCTGGAGAGCTGCAGGAACAGTGGGCCTCTGACTGTGTTGGGTCCTTCTCGATCCTCTGAAGACCTGGCTGGCCCTCTCCCTTCTTCAATCTCTTCATCCTCCTCAAAGCCTAAGCTGAAGAAACGTTTCTCCCTCCGCTCTGTGGGCCGATCAGTGCGAGGCTCGGTCCGAGGGATCTTGCAGTGGCGGGGAGCTGGTGATCCCCCCTCTCCAGTGGGACCCTTGGAGACCTCTGCCTCTGGACCACCTGTCCTGGGTGGAAACAGCAACTCCAACTCCTCTGGTGGGGGTGGTAGCACCAATGTGGGGCCAACTAGCGGTGGGGCATCCCCTGGGGAAAGATGGACACACCGCTTTGAGAGGCTGAGACTAAGTAGGGGAGGGGGACCCTTAAAGGATGGGGCAGGGACAGTGCAAAGGGAGGGGCTACTGAGCTTCATGGGAACAGAGGAGGCAGCTGTTGGTGGAGGAGAAGGTCGAGGGGGAGGAGGAACTGGAACCTCATCTGGGGGTGGGGGACCACCAAGGTGGCAGAAGTGTCGCCTGCTGCTTCGGAGTGAGGGGGAAGGAGGAGGAAGTCGGCTGGAGTTCTATATCCCACCTAAGGTGAGAGAGAAGGGGAAGTGTGACTGGGGATGAATAATGTAGAAGCCTTGCAGATAGAACCCCAGAAGTTTACAAGCCTACAGGCTTTTACTTTTTTTTTTCCTTTGACACTTGGACCCATCTTGCTAACTCTTGACACTTGTTGAGTATGCAGGACTAGGTTGGTGATATCAACAGTCTTTCTGCCACCTCCACATTACTTACAAGGTAGAGCAGGGAGCCATTGGGCTTGTGTTGACACCCCTTTGGTGAAGTGGGAAGAGTTGGGAGGCCAGGTTTCCCCTGGGAAGTGGAGGAGTGTTTTTGAGCCTGAATATTGGGACTCCTACTGCCTCTTCTACCGCAGGCATCCCGGCCCCGGATCAACATCCTGTGTTCTACCGTCACAGATGTCCGGACGACCACAGCCCTGGAGATGCCCGACAGGGAGAACACGTTTGTGGTTAAGGTGGAGGGTCCTATGGAATACATCCTGGAGACAGCTGATGCCCTGAGTATGAAGGCCTGGGTGTCTGATCTACAGGATTGCCTGAGTCCAGGGGAGAACACAGACAGCCTGGACCTGTCCTGCCTCAACCACTCAGAGAGTCTGCCCAGCCAAGAGCTGCCCTTGGGACCCAGTGAGAGCAATGAGCATCTGTCTCAGGGGGCGTATGGGGGCCTCTCGGATCGTCCCTCAGCTTCTGTCTCCCCCAGCTCTGCCTCCATTGCTGCCTCCCATTTTGATTCAATGGAACTACTGCCCCCAGAGTTGCCCCCCCGAGTCCCTATTGAAGAGGGGCCCCCAGTGGGGACAACTTACCCCCTCCCAGCCCCTTACCCTCCTCCGGACACTCCAGACACCACAGGTTCGTTCCTGTTCCAGGGGGAATCAGATGGAGGTGATGGGGACCATCCCCTTTCAGAGTACCCTTGGTTCCATGGGACCCTTTCCCGACTCAAAGCTGCCCAGCTAGTGTTGGCAGGGGGTGCTGGCAGCCATGGTGTCTTCCTGGTACGTCAAAGCGAGACAAGGAGGGGGGAGTATGTCCTCACGTTCAACTTCCAGGGCAAGGCCAAGCACCTTCGTCTGTCCCTGAATGAGGATGGTCAGTGCCGGGTGCAGCACCTCTGGTTCCAGACGGTCTTTGACATGCTGGAGCATTTCCGGGTCCACCCCATTCCCTTGGAGTCTGGGGGCTCCAGTGATGTTACCCTCGTCAGCTATGTTGTGGCCTCTCAGAGGCTACAAGGCAGGGAACGGGCAGGGAGCCGAGCTGGGGTGTGTGAGGGAGATCGCTGCTACCCCGACGCCTCTTCCACCCTCATGCCCATCGGAGCGAGTGACTGTGTAACAGAACATCTCCCATGACCCATCCCAATCTCCTGATCCCGATCTGAGGGCAGACCCTGCACACCCTGGGGAAGAGGAGGAGATGGTGGAGGCATCAGAAGCTTCAGAGGAAGAGAAGGCAGGCAGTGGAGGGACCCCAGGAGAGCCGGGCCCTGTGGCTGAGCTTGAGGAGACCACAGCCCTAAACAGGGAGTCCTCAGGGCTAGAGGGGCCTGGTGGGGAGGTGGGAGTGACCCCTTTTGTGCATTTCTTGCAGTTGCAGCCAGGGAATGCCGGGGAGGAAGGGGTCTGCTCCAGAGCCATCAATAACCAGTACTCCTTCCTATAAGGATGAATACCCCCACACAACCAATTCCCCCTTTCTCCCTGGCCCTGGGCCAGGACCCGTGACAGATCCTCCTCAGAGAGGTACAAATGCTTCCCTTGCCCCTATTTGTCTAGGGGGTAGGAATAGTACAGGACAGAGGGACAGAGCCATTAGTAACTTCCCAGAGGACACACTCCAAGCTGCCCCTTTCCCTGGGCAGGGGACTTGGATCCGTTACCTCCTTTTGGGGGTGGGCCAATTGTGCTCAGCCTCGCCCCCTGTGGGCCATTTCTCCAATACCTCTCACAGCTCTCTGTGATGTATTGGGGAGAGGCTGTATCAGCTACATTAAGGATTTTTTTTTTAAGTGAAATGGAGAAACAAATAAAAAGGTTTATCTCAGCTCTCTT

>UoN.Pci.T.20889_UoN.Pci.T.20889.6

GTCACTGGTTGGTGGGGGTAGGGGGGGGGAGCGGGAGCCGCCGCTGCTGCCGCCGGAGCAAACCGCGGGGACCGAGATGCAGGTGGGACCGGAACCGGAACCCCCCTTCTCATTCTTCTTTCCCCACCCGAACCCTCCCCCCTTTCCCGGGCCTGGGACTGGAGGGAGGGCCGAAGGTCCTGGAGCGGGGCCGGGCTCGGAAGCTGGGGGCTGGGGGAATATCCCCAGGGCAGCGCACCGCCGGAGGCCGGTGGAGCGGGGCGCAATGGGCGGAGGTGAAGGAGGCCGCCCACGGGCGGAGGGAAAGCCGGCGTTCTCCGGAGCCCCCCACCCCCGGAGGAGCGCTGGGGCTGGGCAAGGGAGCAAGGCTGCCCGCGGCCTCATCACTGCAGGGCCAGGGGGTCGGGGCGAGTGGGCAGCAGCAGCCGGGCTAAAGGAGCTACGGCGTTTGGGGGGGAGCGACCTCAAGGGACCCACACTGGGTGAGGGGTCCCCCCTGTTGTTGGTGTGGAACGTACGAGCTCTGGGGTGGAGGGAGTAGGCATGTCATGCTTGGGTGTCTCCCCCAGCATGCACCAAACCTCCCTCTTTCCCAGGCTCTCTTCATTGGCATTTGAGACTATTTCATGTGTTTCTGTCTCCTCGAGGCAGGGTCCGGGGTAGATGTAAAAGGCGGCTGAAAGGATAGGGAATGAAGGGAGGGTGAAGATGTTGGTCCGGAAGTGACCAGCAGAATTGGCGTGGGTTGCCTGTCAGGAAGGAGTTCTTGAGAAAATAAGAAACACAGGCCAGACTTCTTTAAGGCCACTCTCATTCCAGCCCTTTCCATGGAGGTCTGGCCTTAGGAAATTATTTCCTTTGTTCATTCCAGGTTCTGATGTCTCTCTGTGGAAATTCATTTTGTTGCTGAACCTAGGCCTGCGCAGAATTTCCCATCTTAGGGAACTGGAATAAGGAATCAGGGTTCTTAGGTTCTGTGCCTGACTAGAACTCGAAGCCACCACTTTTGCGACCTTAGGCAGTCATTCAACTTCTGAGTCTGTTTTCTTACCCATAAAATGGGCACAATAGCCCCTGGTTTACCTCTCTAATAGGGTTGCTGTGAAGTAGGAGAATGTGTGTGAAAATGCTTTGAAATCATAAAGTGCTATGTGAATGCTGTTATTGGTCAAGCAGAAATTTTTCTAGAAACTCTTCCTAACCTGGTCAACTAGTGATGCTACAAATAGTGGCTATGAGTCTGGTCTGAAGAAGCACAGGATTAGTGGCCCTCAGTTCTGAGCCGGGTTTTTCCCGGAGTCCAGTTGCTCTGGGCTGTGGGACTAAGGGCCTGTGTGCTCATTTTCACAGCTTGTCTGACCTTTAGCCCCTTTTGCTTTCTTTTATTCTTTGACATTAGAAAATACTTCATCCCAGCCTTCTTATAGTTTCTTTTATTCTTGAGACTGAGGGGAAGAAGTAAGGTAGCAGTACTCAATATTTGGATGGTGAGGAGCTACTGAGGATGGAAGGGGGTGGGTGGGAATGATGCCTTCAAGGCAGTACCCTCATACCATAATGCTTAAAATGCCAATGAGCCTTAGGGAGAGAAGCAAGGTGGCAGTCACCTACCTATTGCTGACCTGTTAGGGACCTTTAGAGTAAGATATTGGATATATTTATTTCTGAAAGAGAAGAGATTGGAAAGGAAGCTTTCTGTCTCTGAAGGGTTTCTTTTTTGGAGGATGTCGACCAGCTTACCTTTAACCCTTCTGGGATCAAAGCCAAAGGATGTGGGCTGAGATTACAGCAGGAAAGATAGAGGCTAGATATCAGAAAAAAACCTACCAGTGCTTGAGAGGTGTGAGAACCTGGAATATGGGAAGGAAGCTAACATATTTTGAAGGACTTTTCTTGAATCTGTCTGAATCAGAGAGGAACAAGGTGAACAGAAGGCAGTGTGGAAAAGTGGATGGCTTCTCAGTGAGGGTGGAACAATTGGGGAATCTCTGGTATTTGGGGAAAGGTCTAGAACTTATTCTCTATAAGGTATAATTCTAATACTGACTCCTATATATGTGCCTCTTTTTCCCTTCCACAGTTGCTGCAGATGACCCTGAGTCTTCTGGCCTCCCCCCCCACTTCCTTCCCTCCCCTGGGTCCCCCACTCTCCATTTCATCTGGCTCATCATGAATGGTGCCCCTTCCCCTGAGGATGGGACTTCCCCTTCTCCTCCCCCTCTGCCACCACCACTTCCACCACCTCCTCCCCCAAGTTGGCGGGAGTTCTGTGAATCCCACGCTCGGGCAGCAGCCCTAGACTTTGCCCGACGCTTCCGCCTCTACTTGGCTTCCCATCCCCAGTATGCAGGGCCTGGGGCTGAGGCAGCCTTCTCCCGACGTTTTGCTGAGCTCTTCTTGCAGCACTTTGAGGCTGAGGTGGCCCGGGCCTCAGGTTCCCTTTTATCATCTGGTCCGATCCCCCTGAGTCCCAGTACGGAAACCCCATCACGTGATCTTTCCCTGGAGAGCTGCAGGAACAGTGGGCCTCTGACTGTGTTGGGTCCTTCTCGATCCTCTGAAGACCTGGCTGGCCCTCTCCCTTCTTCAATCTCTTCATCCTCCTCAAAGCCTAAGCTGAAGAAACGTTTCTCCCTCCGCTCTGTGGGCCGATCAGTGCGAGGCTCGGTCCGAGGGATCTTGCAGTGGCGGGGAGCTGGTGATCCCCCCTCTCCAGTGGGACCCTTGGAGACCTCTGCCTCTGGACCACCTGTCCTGGGTGGAAACAGCAACTCCAACTCCTCTGGTGGGGGTGGTAGCACCAATGTGGGGCCAACTAGCGGTGGGGCATCCCCTGGGGAAAGATGGACACACCGCTTTGAGAGGCTGAGACTAAGTAGGGGAGGGGGACCCTTAAAGGATGGGGCAGGGACAGTGCAAAGGGAGGGGCTACTGAGCTTCATGGGAACAGAGGAGGCAGCTGTTGGTGGAGGAGAAGGTCGAGGGGGAGGAGGAACTGGAACCTCATCTGGGGGTGGGGGACCACCAAGGTGGCAGAAGTGTCGCCTGCTGCTTCGGAGTGAGGGGGAAGGAGGAGGAAGTCGGCTGGAGTTCTATATCCCACCTAAGGTGAGAGAGAAGGGGAAGTGTGACTGGGGATGAATAATGTAGAAGCCTTGCAGATAGAACCCCAGAAGTTTACAAGCCTACAGGCTTTTACTTTTTTTTTTCCTTTGACACTTGGACCCATCTTGCTAACTCTTGACACTTGTTGAGTATGCAGGACTAGGTTGGTGATATCAACAGTCTTTCTGCCACCTCCACATTACTTACAAGGTAGAGCAGGGAGCCATTGGGCTTGTGTTGACACCCCTTTGGTGAAGTGGGAAGAGTTGGGAGGCCAGGTTTCCCCTGGGAAGTGGAGGAGTGTTTTTGAGCCTGAATATTGGGACTCCTACTGCCTCTTCTACCGCAGGCATCCCGGCCCCGGATCAACATCCTGTGTTCTACCGTCACAGATGTCCGGACGACCACAGCCCTGGAGATGCCCGACAGGGAGAACACGTTTGTGGTTAAGGTGGAGGGTCCTATGGAATACATCCTGGAGACAGCTGATGCCCTGAGTATGAAGGCCTGGGTGTCTGATCTACAGGATTGCCTGAGTCCAGGGGAGAACACAGACAGCCTGGACCTGTCCTGCCTCAACCACTCAGAGAGTCTGCCCAGCCAAGAGCTGCCCTTGGGACCCAGTGAGAGCAATGAGCATCTGTCTCAGGGGGCGTATGGGGGCCTCTCGGATCGTCCCTCAGCTTCTGTCTCCCCCAGCTCTGCCTCCATTGCTGCCTCCCATTTTGATTCAATGGAACTACTGCCCCCAGAGTTGCCCCCCCGAGTCCCTATTGAAGAGGGGCCCCCAGTGGGGACAACTTACCCCCTCCCAGCCCCTTACCCTCCTCCGGACACTCCAGACACCACAGGTTCGTTCCTGTTCCAGGGGGAATCAGATGGAGGTGATGGGGACCATCCCCTTTCAGAGTACCCTTGGTTCCATGGGACCCTTTCCCGACTCAAAGCTGCCCAGCTAGTGTTGGCAGGGGGTGCTGGCAGCCATGGTGTCTTCCTGGTACGTCAAAGCGAGACAAGGAGGGGGGAGTATGTCCTCACGTTCAACTTCCAGGGCAAGGCCAAGCACCTTCGTCTGTCCCTGAATGAGGATGGTCAGTGCCGGGTGCAGCACCTCTGGTTCCAGACGGTCTTTGACATGCTGGAGCATTTCCGGGTCCACCCCATTCCCTTGGAGTCTGGGGGCTCCAGTGATGTTACCCTCGTCAGCTATGTTGTGGCCTCTCAGAGGCTACAAGGCAGGGAACGGGCAGGGAGCCGAGCTGGGGTGTGTGAGGGAGATCGCTGCTACCCCGACGCCTCTTCCACCCTCATGCCCATCGGAGCGAGTGACTGTGTAACAGAACATCTCCCATGACCCATCCCAATCTCCTGATCCCGATCTGAGGGCAGACCCTGCACACCCTGGGGAAGAGGAGGAGATGGTGGAGGCATCAGAAGCTTCAGAGGAAGAGAAGGCAGGCAGTGGAGGGACCCCAGGAGAGCCGGGCCCTGTGGCTGAGCTTGAGGAGACCACAGCCCTAAACAGGGAGTCCTCAGGGCTAGAGGGGCCTGGTGGGGAGGTGGGAGTGACCCCTTTTGTGCATTTCTTGCAGTTGCAGCCAGGGAATGCCGGGGAGGAAGGGGTCTGCTCCAGAGCCATCAATAACCAGTACTCCTTCCTATAAGGATGAATACCCCCACACAACCAATTCCCCCTTTCTCCCTGGCCCTGGGCCAGGACCCGTGACAGATCCTCCTCAGAGAGGTACAAATGCTTCCCTTGCCCCTATTTGTCTAGGGGGTAGGAATAGTACAGGACAGAGGGACAGAGCCATTAGTAACTTCCCAGAGGACACACTCCAAGCTGCCCCTTTCCCTGGGCAGGGGACTTGGATCCGTTACCTCCTTTTGGGGGTGGGCCAATTGTGCTCAGCCTCGCCCCCTGTGGGCCATTTCTCCAATACCTCTCACAGCTCTCTGTGATGTATTGGGGAGAGGCTGTATCAGCTACATTAAGGATTTTTTTTTTAAGTGAAATGGAGAAACAAATAAAAAGGTTTATCTCAGCTCTCTTCTGGTCATTGGCCCAATGTTTGTATCACAGAATGGAATTGGAGATGGGGACATAATTGGCTCAGGAGGTGCACATAGATATGCTTTTGAGTCCTGGATCAGCCACTTTCTGGGTGACTTTGTGCCTGTCACTTCCCATGTCAGACTAGTCAGCTCTTAAGCCCCTTCCAACTCTTAACGTTCTGGTTCTGGAATTCCAACCCCTGTGGGAGAGCTGTACATAGAGCCAGGGCATGAGGGTGGCTTCTTTTTTCCTAAATAGGTTGTTCTGGGAGAGGTCAAAGGTAATGGCCATTGATCTGGGCTGGAAAGGCCAGACTTGGGAGCAGGGTTTCTGGGTTCTTGAGAAAGGAGGCATCTGGCCTGGGACTGGGAAACTTGTAGTAGCTCTGCTACTCAGTTACATGTGTTACCTTGGAGAAGTCACTCAATTTATTTGGG

>UoN.Pci.T.20889_UoN.Pci.T.20889.7

GTCACTGGTTGGTGGGGGTAGGGGGGGGGAGCGGGAGCCGCCGCTGCTGCCGCCGGAGCAAACCGCGGGGACCGAGATGCAGGTGGGACCGGAACCGGAACCCCCCTTCTCATTCTTCTTTCCCCACCCGAACCCTCCCCCCTTTCCCGGGCCTGGGACTGGAGGGAGGGCCGAAGGTCCTGGAGCGGGGCCGGGCTCGGAAGCTGGGGGCTGGGGGAATATCCCCAGGGCAGCGCACCGCCGGAGGCCGGTGGAGCGGGGCGCAATGGGCGGAGGTGAAGGAGGCCGCCCACGGGCGGAGGGAAAGCCGGCGTTCTCCGGAGCCCCCCACCCCCGGAGGAGCGCTGGGGCTGGGCAAGGGAGCAAGGCTGCCCGCGGCCTCATCACTGCAGGGCCAGGGGGTCGGGGCGAGTGGGCAGCAGCAGCCGGGCTAAAGGAGCTACGGCGTTTGGGGGGGAGCGACCTCAAGGGACCCACACTGGGTGAGGGGTCCCCCCTGTTGTTGGTGTGGAACGTACGAGCTCTGGGGTGGAGGGAGTAGGCATGTCATGCTTGGGTGTCTCCCCCAGCATGCACCAAACCTCCCTCTTTCCCAGGCTCTCTTCATTGGCATTTGAGACTATTTCATGTGTTTCTGTCTCCTCGAGGCAGGGTCCGGGGTAGATGTAAAAGGCGGCTGAAAGGATAGGGAATGAAGGGAGGGTGAAGATGTTGGTCCGGAAGTGACCAGCAGAATTGGCGTGGGTTGCCTGTCAGGAAGGAGTTCTTGAGAAAATAAGAAACACAGGCCAGACTTCTTTAAGGCCACTCTCATTCCAGCCCTTTCCATGGAGGTCTGGCCTTAGGAAATTATTTCCTTTGTTCATTCCAGGTTCTGATGTCTCTCTGTGGAAATTCATTTTGTTGCTGAACCTAGGCCTGCGCAGAATTTCCCATCTTAGGGAACTGGAATAAGGAATCAGGGTTCTTAGGTTCTGTGCCTGACTAGAACTCGAAGCCACCACTTTTGCGACCTTAGGCAGTCATTCAACTTCTGAGTCTGTTTTCTTACCCATAAAATGGGCACAATAGCCCCTGGTTTACCTCTCTAATAGGGTTGCTGTGAAGTAGGAGAATGTGTGTGAAAATGCTTTGAAATCATAAAGTGCTATGTGAATGCTGTTATTGGTCAAGCAGAAATTTTTCTAGAAACTCTTCCTAACCTGGTCAACTAGTGATGCTACAAATAGTGGCTATGAGTCTGGTCTGAAGAAGCACAGGATTAGTGGCCCTCAGTTCTGAGCCGGGTTTTTCCCGGAGTCCAGTTGCTCTGGGCTGTGGGACTAAGGGCCTGTGTGCTCATTTTCACAGCTTGTCTGACCTTTAGCCCCTTTTGCTTTCTTTTATTCTTTGACATTAGAAAATACTTCATCCCAGCCTTCTTATAGTTTCTTTTATTCTTGAGACTGAGGGGAAGAAGTAAGGTAGCAGTACTCAATATTTGGATGGTGAGGAGCTACTGAGGATGGAAGGGGGTGGGTGGGAATGATGCCTTCAAGGCAGTACCCTCATACCATAATGCTTAAAATGCCAATGAGCCTTAGGGAGAGAAGCAAGGTGGCAGTCACCTACCTATTGCTGACCTGTTAGGGACCTTTAGAGTAAGATATTGGATATATTTATTTCTGAAAGAGAAGAGATTGGAAAGGAAGCTTTCTGTCTCTGAAGGGTTTCTTTTTTGGAGGATGTCGACCAGCTTACCTTTAACCCTTCTGGGATCAAAGCCAAAGGATGTGGGCTGAGATTACAGCAGGAAAGATAGAGGCTAGATATCAGAAAAAAACCTACCAGTGCTTGAGAGGTGTGAGAACCTGGAATATGGGAAGGAAGCTAACATATTTTGAAGGACTTTTCTTGAATCTGTCTGAATCAGAGAGGAACAAGGTGAACAGAAGGCAGTGTGGAAAAGTGGATGGCTTCTCAGTGAGGGTGGAACAATTGGGGAATCTCTGGTATTTGGGGAAAGGTCTAGAACTTATTCTCTATAAGGTATAATTCTAATACTGACTCCTATATATGTGCCTCTTTTTCCCTTCCACAGTTGCTGCAGATGACCCTGAGTCTTCTGGCCTCCCCCCCCACTTCCTTCCCTCCCCTGGGTCCCCCACTCTCCATTTCATCTGGCTCATCATGAATGGTGCCCCTTCCCCTGAGGATGGGACTTCCCCTTCTCCTCCCCCTCTGCCACCACCACTTCCACCACCTCCTCCCCCAAGTTGGCGGGAGTTCTGTGAATCCCACGCTCGGGCAGCAGCCCTAGACTTTGCCCGACGCTTCCGCCTCTACTTGGCTTCCCATCCCCAGTATGCAGGGCCTGGGGCTGAGGCAGCCTTCTCCCGACGTTTTGCTGAGCTCTTCTTGCAGCACTTTGAGGCTGAGGTGGCCCGGGCCTCAGGTTCCCTTTTATCATCTGGTCCGATCCCCCTGAGTCCCAGTACGGAAACCCCATCACGTGATCTTTCCCTGGAGAGCTGCAGGAACAGTGGGCCTCTGACTGTGTTGGGTCCTTCTCGATCCTCTGAAGACCTGGCTGGCCCTCTCCCTTCTTCAATCTCTTCATCCTCCTCAAAGCCTAAGCTGAAGAAACGTTTCTCCCTCCGCTCTGTGGGCCGATCAGTGCGAGGCTCGGTCCGAGGGATCTTGCAGTGGCGGGGAGCTGGTGATCCCCCCTCTCCAGTGGGACCCTTGGAGACCTCTGCCTCTGGACCACCTGTCCTGGGTGGAAACAGCAACTCCAACTCCTCTGGTGGGGGTGGTAGCACCAATGTGGGGCCAACTAGCGGTGGGGCATCCCCTGGGGAAAGATGGACACACCGCTTTGAGAGGCTGAGACTAAGTAGGGGAGGGGGACCCTTAAAGGATGGGGCAGGGACAGTGCAAAGGGAGGGGCTACTGAGCTTCATGGGAACAGAGGAGGCAGCTGTTGGTGGAGGAGAAGGTCGAGGGGGAGGAGGAACTGGAACCTCATCTGGGGGTGGGGGACCACCAAGGTGGCAGAAGTGTCGCCTGCTGCTTCGGAGTGAGGGGGAAGGAGGAGGAAGTCGGCTGGAGTTCTATATCCCACCTAAGGTGAGAGAGAAGGGGAAGTGTGACTGGGGATGAATAATGTAGAAGCCTTGCAGATAGAACCCCAGAAGTTTACAAGCCTACAGGCTTTTACTTTTTTTTTTCCTTTGACACTTGGACCCATCTTGCTAACTCTTGACACTTGTTGAGTATGCAGGACTAGGTTGGTGATATCAACAGTCTTTCTGCCACCTCCACATTACTTACAAGGTAGAGCAGGGAGCCATTGGGCTTGTGTTGACACCCCTTTGGTGAAGTGGGAAGAGTTGGGAGGCCAGGTTTCCCCTGGGAAGTGGAGGAGTGTTTTTGAGCCTGAATATTGGGACTCCTACTGCCTCTTCTACCGCAGGCATCCCGGCCCCGGATCAACATCCTGTGTTCTACCGTCACAGATGTCCGGACGACCACAGCCCTGGAGATGCCCGACAGGGAGAACACGTTTGTGGTTAAGGTGGAGGGTCCTATGGAATACATCCTGGAGACAGCTGATGCCCTGAGTATGAAGGCCTGGGTGTCTGATCTACAGGATTGCCTGAGTCCAGGGGAGAACACAGACAGCCTGGACCTGTCCTGCCTCAACCACTCAGAGAGTCTGCCCAGCCAAGAGCTGCCCTTGGGACCCAGTGAGAGCAATGAGCATCTGTCTCAGGGGGCGTATGGGGGCCTCTCGGATCGTCCCTCAGCTTCTGTCTCCCCCAGCTCTGCCTCCATTGCTGCCTCCCATTTTGATTCAATGGAACTACTGCCCCCAGAGTTGCCCCCCCGAGTCCCTATTGAAGAGGGGCCCCCAGTGGGGACAACTTACCCCCTCCCAGCCCCTTACCCTCCTCCGGACACTCCAGACACCACAGGTTCGTTCCTGTTCCAGGGGGAATCAGATGGAGGTGATGGGGACCATCCCCTTTCAGAGTACCCTTGGTTCCATGGGACCCTTTCCCGACTCAAAGCTGCCCAGCTAGTGTTGGCAGGGGGTGCTGGCAGCCATGGTGTCTTCCTGGTACGTCAAAGCGAGACAAGGAGGGGGGAGTATGTCCTCACGTTCAACTTCCAGGGCAAGGCCAAGCACCTTCGTCTGTCCCTGAATGAGGATGGTCAGTGCCGGGTGCAGCACCTCTGGTTCCAGACGGTCTTTGACATGCTGGAGCATTTCCGGGTCCACCCCATTCCCTTGGAGTCTGGGGGCTCCAGTGATGTTACCCTCGTCAGCTATGTTGTGGCCTCTCAGAGGCTACAAGGCAGGGAACGGGCAGGGAGCCGAGCTGGGGTGTGTGAGGGAGATCGCTGCTACCCCGACGCCTCTTCCACCCTCATGCCCATCGGAGCGAGTGACTGTGTAACAGAACATCTCCCATGACCCATCCCAATCTCCTGATCCCGATCTGAGGGCAGACCCTGCACACCCTGGGGAAGAGGAGGAGATGGTGGAGGCATCAGAAGCTTCAGAGGAAGAGAAGGCAGGCAGTGGAGGGACCCCAGGAGAGCCGGGCCCTGTGGCTGAGCTTGAGGAGACCACAGCCCTAAACAGGGAGTCCTCAGGGCTAGAGGGGCCTGGTGGGGAGGTGGGAGTGACCCCTTTTGTGCATTTCTTGCAGTTGCAGCCAGGGAATGCCGGGGAGGAAGGGGTCTGCTCCAGAGCCATCAATAACCAGTACTCCTTCCTATAAGGATGAATACCCCCACACAACCAATTCCCCCTTTCTCCCTGGCCCTGGGCCAGGACCCGTGACAGATCCTCCTCAGAGAGGTACAAATGCTTCCCTTGCCCCTATTTGTCTAGGGGGTAGGAATAGTACAGGACAGAGGGACAGAGCCATTAGTAACTTCCCAGAGGACACACTCCAAGCTGCCCCTTTCCCTGGGCAGGGGACTTGGATCCGTTACCTCCTTTTGGGGGTGGGCCAATTGTGCTCAGCCTCGCCCCCTGTGGGCCATTTCTCCAATACCTCTCACAGCTCTCTGTGATGTATTGGGGAGAGGCTGTATCAGCTACATTAAGGATTTTTTTTTTAAGTGAAATGGAGAAACAAATAA

>UoN.Pci.T.20889_UoN.Pci.T.20889.8

GTCACTGGTTGGTGGGGGTAGGGGGGGGGAGCGGGAGCCGCCGCTGCTGCCGCCGGAGCAAACCGCGGGGACCGAGATGCAGGTGGGACCGGAACCGGAACCCCCCTTCTCATTCTTCTTTCCCCACCCGAACCCTCCCCCCTTTCCCGGGCCTGGGACTGGAGGGAGGGCCGAAGGTCCTGGAGCGGGGCCGGGCTCGGAAGCTGGGGGCTGGGGGAATATCCCCAGGGCAGCGCACCGCCGGAGGCCGGTGGAGCGGGGCGCAATGGGCGGAGGTGAAGGAGGCCGCCCACGGGCGGAGGGAAAGCCGGCGTTCTCCGGAGCCCCCCACCCCCGGAGGAGCGCTGGGGCTGGGCAAGGGAGCAAGGCTGCCCGCGGCCTCATCACTGCAGGGCCAGGGGGTCGGGGCGAGTGGGCAGCAGCAGCCGGGCTAAAGGAGCTACGGCGTTTGGGGGGGAGCGACCTCAAGGGACCCACACTGGGTGAGGGGTCCCCCCTGTTGTTGGTGTGGAACGTACGAGCTCTGGGGTGGAGGGAGTAGGCATGTCATGCTTGGGTGTCTCCCCCAGCATGCACCAAACCTCCCTCTTTCCCAGGCTCTCTTCATTGGCATTTGAGACTATTTCATGTGTTTCTGTCTCCTCGAGGCAGGGTCCGGGGTAGATGTAAAAGGCGGCTGAAAGGATAGGGAATGAAGGGAGGGTGAAGATGTTGGTCCGGAAGTGACCAGCAGAATTGGCGTGGGTTGCCTGTCAGGAAGGAGTTCTTGAGAAAATAAGAAACACAGGCCAGACTTCTTTAAGGCCACTCTCATTCCAGCCCTTTCCATGGAGGTCTGGCCTTAGGAAATTATTTCCTTTGTTCATTCCAGGTTCTGATGTCTCTCTGTGGAAATTCATTTTGTTGCTGAACCTAGGCCTGCGCAGAATTTCCCATCTTAGGGAACTGGAATAAGGAATCAGGGTTCTTAGGTTCTGTGCCTGACTAGAACTCGAAGCCACCACTTTTGCGACCTTAGGCAGTCATTCAACTTCTGAGTCTGTTTTCTTACCCATAAAATGGGCACAATAGCCCCTGGTTTACCTCTCTAATAGGGTTGCTGTGAAGTAGGAGAATGTGTGTGAAAATGCTTTGAAATCATAAAGTGCTATGTGAATGCTGTTATTGGTCAAGCAGAAATTTTTCTAGAAACTCTTCCTAACCTGGTCAACTAGTGATGCTACAAATAGTGGCTATGAGTCTGGTCTGAAGAAGCACAGGATTAGTGGCCCTCAGTTCTGAGCCGGGTTTTTCCCGGAGTCCAGTTGCTCTGGGCTGTGGGACTAAGGGCCTGTGTGCTCATTTTCACAGCTTGTCTGACCTTTAGCCCCTTTTGCTTTCTTTTATTCTTTGACATTAGAAAATACTTCATCCCAGCCTTCTTATAGTTTCTTTTATTCTTGAGACTGAGGGGAAGAAGTAAGGTAGCAGTACTCAATATTTGGATGGTGAGGAGCTACTGAGGATGGAAGGGGGTGGGTGGGAATGATGCCTTCAAGGCAGTACCCTCATACCATAATGCTTAAAATGCCAATGAGCCTTAGGGAGAGAAGCAAGGTGGCAGTCACCTACCTATTGCTGACCTGTTAGGGACCTTTAGAGTAAGATATTGGATATATTTATTTCTGAAAGAGAAGAGATTGGAAAGGAAGCTTTCTGTCTCTGAAGGGTTTCTTTTTTGGAGGATGTCGACCAGCTTACCTTTAACCCTTCTGGGATCAAAGCCAAAGGATGTGGGCTGAGATTACAGCAGGAAAGATAGAGGCTAGATATCAGAAAAAAACCTACCAGTGCTTGAGAGGTGTGAGAACCTGGAATATGGGAAGGAAGCTAACATATTTTGAAGGACTTTTCTTGAATCTGTCTGAATCAGAGAGGAACAAGGTGAACAGAAGGCAGTGTGGAAAAGTGGATGGCTTCTCAGTGAGGGTGGAACAATTGGGGAATCTCTGGTATTTGGGGAAAGGTCTAGAACTTATTCTCTATAAGGTATAATTCTAATACTGACTCCTATATATGTGCCTCTTTTTCCCTTCCACAGTTGCTGCAGATGACCCTGAGTCTTCTGGCCTCCCCCCCCACTTCCTTCCCTCCCCTGGGTCCCCCACTCTCCATTTCATCTGGCTCATCATGAATGGTGCCCCTTCCCCTGAGGATGGGACTTCCCCTTCTCCTCCCCCTCTGCCACCACCACTTCCACCACCTCCTCCCCCAAGTTGGCGGGAGTTCTGTGAATCCCACGCTCGGGCAGCAGCCCTAGACTTTGCCCGACGCTTCCGCCTCTACTTGGCTTCCCATCCCCAGTATGCAGGGCCTGGGGCTGAGGCAGCCTTCTCCCGACGTTTTGCTGAGCTCTTCTTGCAGCACTTTGAGGCTGAGGTGGCCCGGGCCTCAGGTTCCCTTTTATCATCTGGTCCGATCCCCCTGAGTCCCAGTACGGAAACCCCATCACGTGATCTTTCCCTGGAGAGCTGCAGGAACAGTGGGCCTCTGACTGTGTTGGGTCCTTCTCGATCCTCTGAAGACCTGGCTGGCCCTCTCCCTTCTTCAATCTCTTCATCCTCCTCAAAGCCTAAGCTGAAGAAACGTTTCTCCCTCCGCTCTGTGGGCCGATCAGTGCGAGGCTCGGTCCGAGGGATCTTGCAGTGGCGGGGAGCTGGTGATCCCCCCTCTCCAGTGGGACCCTTGGAGACCTCTGCCTCTGGACCACCTGTCCTGGGTGGAAACAGCAACTCCAACTCCTCTGGTGGGGGTGGTAGCACCAATGTGGGGCCAACTAGCGGTGGGGCATCCCCTGGGGAAAGATGGACACACCGCTTTGAGAGGCTGAGACTAAGTAGGGGAGGGGGACCCTTAAAGGATGGGGCAGGGACAGTGCAAAGGGAGGGGCTACTGAGCTTCATGGGAACAGAGGAGGCAGCTGTTGGTGGAGGAGAAGGTCGAGGGGGAGGAGGAACTGGAACCTCATCTGGGGGTGGGGGACCACCAAGGTGGCAGAAGTGTCGCCTGCTGCTTCGGAGTGAGGGGGAAGGAGGAGGAAGTCGGCTGGAGTTCTATATCCCACCTAAGGTGAGAGAGAAGGGGAAGTGTGACTGGGGATGAATAATGTAGAAGCCTTGCAGATAGAACCCCAGAAGTTTACAAGCCTACAGGCTTTTACTTTTTTTTTTCCTTTGACACTTGGACCCATCTTGCTAACTCTTGACACTTGTTGAGTATGCAGGACTAGGTTGGTGATATCAACAGTCTTTCTGCCACCTCCACATTACTTACAAGGTAGAGCAGGGAGCCATTGGGCTTGTGTTGACACCCCTTTGGTGAAGTGGGAAGAGTTGGGAGGCCAGGTTTCCCCTGGGAAGTGGAGGAGTGTTTTTGAGCCTGAATATTGGGACTCCTACTGCCTCTTCTACCGCAGGCATCCCGGCCCCGGATCAACATCCTGTGTTCTACCGTCACAGATGTCCGGACGACCACAGCCCTGGAGATGCCCGACAGGGAGAACACGTTTGTGGTTAAGGTGGAGGGTCCTATGGAATACATCCTGGAGACAGCTGATGCCCTGAGTATGAAGGCCTGGGTGTCTGATCTACAGGATTGCCTGAGTCCAGGGGAGAACACAGACAGCCTGGACCTGTCCTGCCTCAACCACTCAGAGAGTCTGCCCAGCCAAGAGCTGCCCTTGGGACCCAGTGAGAGCAATGAGCATCTGTCTCAGGGGGCGTATGGGGGCCTCTCGGATCGTCCCTCAGCTTCTGTCTCCCCCAGCTCTGCCTCCATTGCTGCCTCCCATTTTGATTCAATGGAACTACTGCCCCCAGAGTTGCCCCCCCGAGTCCCTATTGAAGAGGGGCCCCCAGTGGGGACAACTTACCCCCTCCCAGCCCCTTACCCTCCTCCGGACACTCCAGACACCACAGGTTCGTTCCTGTTCCAGGGGGAATCAGATGGAGGTGATGGGGACCATCCCCTTTCAGAGTACCCTTGGTTCCATGGGACCCTTTCCCGACTCAAAGCTGCCCAGCTAGTGTTGGCAGGGGGTGCTGGCAGCCATGGTGTCTTCCTGGTACGTCAAAGCGAGACAAGGAGGGGGGAGTATGTCCTCACGTTCAACTTCCAGGGCAAGGCCAAGCACCTTCGTCTGTCCCTGAATGAGGATGGTCAGTGCCGGGTGCAGCACCTCTGGTTCCAGACGGTCTTTGACATGCTGGAGCATTTCCGGGTCCACCCCATTCCCTTGGAGTCTGGGGGCTCCAGTGATGTTACCCTCGTCAGCTATGTTGTGGCCTCTCAGAGGCTACAAGGCAGGGAACGGGCAGGGAGCCGAGCTGGGGTGTGTGAGGGAGATCGCTGCTACCCCGACGCCTCTTCCACCCTCATGCCCATCGGAGCGAGTGACTGTGTAACAGAACATCTCCCATGACCCATCCCAATCTCCTGATCCCGATCTGAGGGCAGACCCTGCACACCCTGGGGAAGAGGAGGAGATGGTGGAGGCATCAGAAGCTTCAGAGGAAGAGAAGGCAGGCAGTGGAGGGACCCCAGGAGAGCCGGGCCCTGTGGCTGAGCTTGAGGAGACCACAGCCCTAAACAGGGAGTCCTCAGGGCTAGAGGGGCCTGGTGGGGAGGTGGGAGTGACCCCTTTTGTGCATTTCTTGCAGTTGCAGCCAGGGAATGCCGGGGAGGAAGGGGTCTGCTCCAGAGCCATCAATAACCAGTACTCCTTCCTATAAGGATGAATACCCCCACACAACCAATTCCCCCTTTCTCCCTGGCCCTGGGCCAGGACCCGTGACAGATCCTCCTCAGAGAGGTACAAATGCTTCCCTTGCCCCTATTTGTCTAGGGGGTAGGAATAGTACAGGACAGAGGGACAGAGCCATTAGTAACTTCCCAGAGGACACACTCCAAGCTGCCCCTTTCCCTGGGCAGGGGACTTGGATCCGTTACCTCCTTTTGGGGGTGGGCCAATTGTGCTCAGCCTCGCCCCCTGTGGGCCATTTCTCCAATACCTCTCACAGCTCTCTGTGATGTATTGGGGAGAGGCTGTATCAGCTACATTAAGGATTTTTTTTTTAAGTGAAATGGAGAAACAAATAAAAAGGTTTATCTCA

>UoN.Pci.T.20889_UoN.Pci.T.20889.9

GTCACTGGTTGGTGGGGGTAGGGGGGGGGAGCGGGAGCCGCCGCTGCTGCCGCCGGAGCAAACCGCGGGGACCGAGATGCAGGTGGGACCGGAACCGGAACCCCCCTTCTCATTCTTCTTTCCCCACCCGAACCCTCCCCCCTTTCCCGGGCCTGGGACTGGAGGGAGGGCCGAAGGTCCTGGAGCGGGGCCGGGCTCGGAAGCTGGGGGCTGGGGGAATATCCCCAGGGCAGCGCACCGCCGGAGGCCGGTGGAGCGGGGCGCAATGGGCGGAGGTGAAGGAGGCCGCCCACGGGCGGAGGGAAAGCCGGCGTTCTCCGGAGCCCCCCACCCCCGGAGGAGCGCTGGGGCTGGGCAAGGGAGCAAGGCTGCCCGCGGCCTCATCACTGCAGGGCCAGGGGGTCGGGGCGAGTGGGCAGCAGCAGCCGGGCTAAAGGAGCTACGGCGTTTGGGGGGGAGCGACCTCAAGGGACCCACACTGGGTGAGGGGTCCCCCCTGTTGTTGGTGTGGAACGTACGAGCTCTGGGGTGGAGGGAGTAGGCATGTCATGCTTGGGTGTCTCCCCCAGCATGCACCAAACCTCCCTCTTTCCCAGGCTCTCTTCATTGGCATTTGAGACTATTTCATGTGTTTCTGTCTCCTCGAGGCAGGGTCCGGGGTAGATGTAAAAGGCGGCTGAAAGGATAGGGAATGAAGGGAGGGTGAAGATGTTGGTCCGGAAGTGACCAGCAGAATTGGCGTGGGTTGCCTGTCAGGAAGGAGTTCTTGAGAAAATAAGAAACACAGGCCAGACTTCTTTAAGGCCACTCTCATTCCAGCCCTTTCCATGGAGGTCTGGCCTTAGGAAATTATTTCCTTTGTTCATTCCAGGTTCTGATGTCTCTCTGTGGAAATTCATTTTGTTGCTGAACCTAGGCCTGCGCAGAATTTCCCATCTTAGGGAACTGGAATAAGGAATCAGGGTTCTTAGGTTCTGTGCCTGACTAGAACTCGAAGCCACCACTTTTGCGACCTTAGGCAGTCATTCAACTTCTGAGTCTGTTTTCTTACCCATAAAATGGGCACAATAGCCCCTGGTTTACCTCTCTAATAGGGTTGCTGTGAAGTAGGAGAATGTGTGTGAAAATGCTTTGAAATCATAAAGTGCTATGTGAATGCTGTTATTGGTCAAGCAGAAATTTTTCTAGAAACTCTTCCTAACCTGGTCAACTAGTGATGCTACAAATAGTGGCTATGAGTCTGGTCTGAAGAAGCACAGGATTAGTGGCCCTCAGTTCTGAGCCGGGTTTTTCCCGGAGTCCAGTTGCTCTGGGCTGTGGGACTAAGGGCCTGTGTGCTCATTTTCACAGCTTGTCTGACCTTTAGCCCCTTTTGCTTTCTTTTATTCTTTGACATTAGAAAATACTTCATCCCAGCCTTCTTATAGTTTCTTTTATTCTTGAGACTGAGGGGAAGAAGTAAGGTAGCAGTACTCAATATTTGGATGGTGAGGAGCTACTGAGGATGGAAGGGGGTGGGTGGGAATGATGCCTTCAAGGCAGTACCCTCATACCATAATGCTTAAAATGCCAATGAGCCTTAGGGAGAGAAGCAAGGTGGCAGTCACCTACCTATTGCTGACCTGTTAGGGACCTTTAGAGTAAGATATTGGATATATTTATTTCTGAAAGAGAAGAGATTGGAAAGGAAGCTTTCTGTCTCTGAAGGGTTTCTTTTTTGGAGGATGTCGACCAGCTTACCTTTAACCCTTCTGGGATCAAAGCCAAAGGATGTGGGCTGAGATTACAGCAGGAAAGATAGAGGCTAGATATCAGAAAAAAACCTACCAGTGCTTGAGAGGTGTGAGAACCTGGAATATGGGAAGGAAGCTAACATATTTTGAAGGACTTTTCTTGAATCTGTCTGAATCAGAGAGGAACAAGGTGAACAGAAGGCAGTGTGGAAAAGTGGATGGCTTCTCAGTGAGGGTGGAACAATTGGGGAATCTCTGGTATTTGGGGAAAGGTCTAGAACTTATTCTCTATAAGGTATAATTCTAATACTGACTCCTATATATGTGCCTCTTTTTCCCTTCCACAGTTGCTGCAGATGACCCTGAGTCTTCTGGCCTCCCCCCCCACTTCCTTCCCTCCCCTGGGTCCCCCACTCTCCATTTCATCTGGCTCATCATGAATGGTGCCCCTTCCCCTGAGGATGGGACTTCCCCTTCTCCTCCCCCTCTGCCACCACCACTTCCACCACCTCCTCCCCCAAGTTGGCGGGAGTTCTGTGAATCCCACGCTCGGGCAGCAGCCCTAGACTTTGCCCGACGCTTCCGCCTCTACTTGGCTTCCCATCCCCAGTATGCAGGGCCTGGGGCTGAGGCAGCCTTCTCCCGACGTTTTGCTGAGCTCTTCTTGCAGCACTTTGAGGCTGAGGTGGCCCGGGCCTCAGGTTCCCTTTTATCATCTGGTCCGATCCCCCTGAGTCCCAGTACGGAAACCCCATCACGTGATCTTTCCCTGGAGAGCTGCAGGAACAGTGGGCCTCTGACTGTGTTGGGTCCTTCTCGATCCTCTGAAGACCTGGCTGGCCCTCTCCCTTCTTCAATCTCTTCATCCTCCTCAAAGCCTAAGCTGAAGAAACGTTTCTCCCTCCGCTCTGTGGGCCGATCAGTGCGAGGCTCGGTCCGAGGGATCTTGCAGTGGCGGGGAGCTGGTGATCCCCCCTCTCCAGTGGGACCCTTGGAGACCTCTGCCTCTGGACCACCTGTCCTGGGTGGAAACAGCAACTCCAACTCCTCTGGTGGGGGTGGTAGCACCAATGTGGGGCCAACTAGCGGTGGGGCATCCCCTGGGGAAAGATGGACACACCGCTTTGAGAGGCTGAGACTAAGTAGGGGAGGGGGACCCTTAAAGGATGGGGCAGGGACAGTGCAAAGGGAGGGGCTACTGAGCTTCATGGGAACAGAGGAGGCAGCTGTTGGTGGAGGAGAAGGTCGAGGGGGAGGAGGAACTGGAACCTCATCTGGGGGTGGGGGACCACCAAGGTGGCAGAAGTGTCGCCTGCTGCTTCGGAGTGAGGGGGAAGGAGGAGGAAGTCGGCTGGAGTTCTATATCCCACCTAAGGTGAGAGAGAAGGGGAAGTGTGACTGGGGATGAATAATGTAGAAGCCTTGCAGATAGAACCCCAGAAGTTTACAAGCCTACAGGCTTTTACTTTTTTTTTTCCTTTGACACTTGGACCCATCTTGCTAACTCTTGACACTTGTTGAGTATGCAGGACTAGGTTGGTGATATCAACAGTCTTTCTGCCACCTCCACATTACTTACAAGGTAGAGCAGGGAGCCATTGGGCTTGTGTTGACACCCCTTTGGTGAAGTGGGAAGAGTTGGGAGGCCAGGTTTCCCCTGGGAAGTGGAGGAGTGTTTTTGAGCCTGAATATTGGGACTCCTACTGCCTCTTCTACCGCAGGCATCCCGGCCCCGGATCAACATCCTGTGTTCTACCGTCACAGATGTCCGGACGACCACAGCCCTGGAGATGCCCGACAGGGAGAACACGTTTGTGGTTAAGGTGGAGGGTCCTATGGAATACATCCTGGAGACAGCTGATGCCCTGAGTATGAAGGCCTGGGTGTCTGATCTACAGGATTGCCTGAGTCCAGGGGAGAACACAGACAGCCTGGACCTGTCCTGCCTCAACCACTCAGAGAGTCTGCCCAGCCAAGAGCTGCCCTTGGGACCCAGTGAGAGCAATGAGCATCTGTCTCAGGGGGCGTATGGGGGCCTCTCGGATCGTCCCTCAGCTTCTGTCTCCCCCAGCTCTGCCTCCATTGCTGCCTCCCATTTTGATTCAATGGAACTACTGCCCCCAGAGTTGCCCCCCCGAGTCCCTATTGAAGAGGGGCCCCCAGTGGGGACAACTTACCCCCTCCCAGCCCCTTACCCTCCTCCGGACACTCCAGACACCACAGGTTCGTTCCTGTTCCAGGGGGAATCAGATGGAGGTGATGGGGACCATCCCCTTTCAGAGTACCCTTGGTTCCATGGGACCCTTTCCCGACTCAAAGCTGCCCAGCTAGTGTTGGCAGGGGGTGCTGGCAGCCATGGTGTCTTCCTGGTACGTCAAAGCGAGACAAGGAGGGGGGAGTATGTCCTCACGTTCAACTTCCAGGGCAAGGCCAAGCACCTTCGTCTGTCCCTGAATGAGGATGGTCAGTGCCGGGTGCAGCACCTCTGGTTCCAGACGGTCTTTGACATGCTGGAGCATTTCCGGGTCCACCCCATTCCCTTGGAGTCTGGGGGCTCCAGTGATGTTACCCTCGTCAGCTATGTTGTGGCCTCTCAGAGGCTACAAGGCAGGGAACGGGCAGGGAGCCGAGCTGGGGTGTGTGAGGGAGATCGCTGCTACCCCGACGCCTCTTCCACCCTCATGCCCATCGGAGCGAGTGACTGTGTAACAGAACATCTCCCATGACCCATCCCAATCTCCTGATCCCGATCTGAGGGCAGACCCTGCACACCCTGGGGAAGAGGAGGAGATGGTGGAGGCATCAGAAGCTTCAGAGGAAGAGAAGGCAGGCAGTGGAGGGACCCCAGGAGAGCCGGGCCCTGTGGCTGAGCTTGAGGAGACCACAGCCCTAAACAGGGAGTCCTCAGGGCTAGAGGGGCCTGGTGGGGAGGTGGGAGTGACCCCTTTTGTGCATTTCTTGCAGTTGCAGCCAGGGAATGCCGGGGAGGAAGGGGTCTGCTCCAGAGCCATCAATAACCAGTACTCCTTCCTATAAGGATGAATACCCCCACACAACCAATTCCCCCTTTCTCCCTGGCCCTGGGCCAGGACCCGTGACAGATCCTCCTCAGAGAGGTACAAATGCTTCCCTTGCCCCTATTTGTCTAGGGGGTAGGAATAGTACAGGACAGAGGGACAGAGCCATTAGTAACTTCCCAGAGGACACACTCCAAGCTGCCCCTTTCCCTGGGCAGGGGACTTGGATCCGTTACCTCCTTTTGGGGGTGGGCCAATTGTGCTCAGCCTCGCCCCCTGTGGGCCATTTCTCCAATACCTCTCACAGCTCTCTGTGATGTATTGGGGAGAGGCTGTATCAGCTACATTAAGGATTTTTTTTTTAAGTGAAATGGAGAAACAAATAAAAAGGTTTATCTCA

>UoN.Pci.T.20889_UoN.Pci.T.20889.10

GTCACTGGTTGGTGGGGGTAGGGGGGGGGAGCGGGAGCCGCCGCTGCTGCCGCCGGAGCAAACCGCGGGGACCGAGATGCAGGTGGGACCGGAACCGGAACCCCCCTTCTCATTCTTCTTTCCCCACCCGAACCCTCCCCCCTTTCCCGGGCCTGGGACTGGAGGGAGGGCCGAAGGTCCTGGAGCGGGGCCGGGCTCGGAAGCTGGGGGCTGGGGGAATATCCCCAGGGCAGCGCACCGCCGGAGGCCGGTGGAGCGGGGCGCAATGGGCGGAGGTGAAGGAGGCCGCCCACGGGCGGAGGGAAAGCCGGCGTTCTCCGGAGCCCCCCACCCCCGGAGGAGCGCTGGGGCTGGGCAAGGGAGCAAGGCTGCCCGCGGCCTCATCACTGCAGGGCCAGGGGGTCGGGGCGAGTGGGCAGCAGCAGCCGGGCTAAAGGAGCTACGGCGTTTGGGGGGGAGCGACCTCAAGGGACCCACACTGGGTGAGGGGTCCCCCCTGTTGTTGGTGTGGAACGTACGAGCTCTGGGGTGGAGGGAGTAGGCATGTCATGCTTGGGTGTCTCCCCCAGCATGCACCAAACCTCCCTCTTTCCCAGGCTCTCTTCATTGGCATTTGAGACTATTTCATGTGTTTCTGTCTCCTCGAGGCAGGGTCCGGGGTAGATGTAAAAGGCGGCTGAAAGGATAGGGAATGAAGGGAGGGTGAAGATGTTGGTCCGGAAGTGACCAGCAGAATTGGCGTGGGTTGCCTGTCAGGAAGGAGTTCTTGAGAAAATAAGAAACACAGGCCAGACTTCTTTAAGGCCACTCTCATTCCAGCCCTTTCCATGGAGGTCTGGCCTTAGGAAATTATTTCCTTTGTTCATTCCAGGTTCTGATGTCTCTCTGTGGAAATTCATTTTGTTGCTGAACCTAGGCCTGCGCAGAATTTCCCATCTTAGGGAACTGGAATAAGGAATCAGGGTTCTTAGGTTCTGTGCCTGACTAGAACTCGAAGCCACCACTTTTGCGACCTTAGGCAGTCATTCAACTTCTGAGTCTGTTTTCTTACCCATAAAATGGGCACAATAGCCCCTGGTTTACCTCTCTAATAGGGTTGCTGTGAAGTAGGAGAATGTGTGTGAAAATGCTTTGAAATCATAAAGTGCTATGTGAATGCTGTTATTGGTCAAGCAGAAATTTTTCTAGAAACTCTTCCTAACCTGGTCAACTAGTGATGCTACAAATAGTGGCTATGAGTCTGGTCTGAAGAAGCACAGGATTAGTGGCCCTCAGTTCTGAGCCGGGTTTTTCCCGGAGTCCAGTTGCTCTGGGCTGTGGGACTAAGGGCCTGTGTGCTCATTTTCACAGCTTGTCTGACCTTTAGCCCCTTTTGCTTTCTTTTATTCTTTGACATTAGAAAATACTTCATCCCAGCCTTCTTATAGTTTCTTTTATTCTTGAGACTGAGGGGAAGAAGTAAGGTAGCAGTACTCAATATTTGGATGGTGAGGAGCTACTGAGGATGGAAGGGGGTGGGTGGGAATGATGCCTTCAAGGCAGTACCCTCATACCATAATGCTTAAAATGCCAATGAGCCTTAGGGAGAGAAGCAAGGTGGCAGTCACCTACCTATTGCTGACCTGTTAGGGACCTTTAGAGTAAGATATTGGATATATTTATTTCTGAAAGAGAAGAGATTGGAAAGGAAGCTTTCTGTCTCTGAAGGGTTTCTTTTTTGGAGGATGTCGACCAGCTTACCTTTAACCCTTCTGGGATCAAAGCCAAAGGATGTGGGCTGAGATTACAGCAGGAAAGATAGAGGCTAGATATCAGAAAAAAACCTACCAGTGCTTGAGAGGTGTGAGAACCTGGAATATGGGAAGGAAGCTAACATATTTTGAAGGACTTTTCTTGAATCTGTCTGAATCAGAGAGGAACAAGGTGAACAGAAGGCAGTGTGGAAAAGTGGATGGCTTCTCAGTGAGGGTGGAACAATTGGGGAATCTCTGGTATTTGGGGAAAGGTCTAGAACTTATTCTCTATAAGGTATAATTCTAATACTGACTCCTATATATGTGCCTCTTTTTCCCTTCCACAGTTGCTGCAGATGACCCTGAGTCTTCTGGCCTCCCCCCCCACTTCCTTCCCTCCCCTGGGTCCCCCACTCTCCATTTCATCTGGCTCATCATGAATGGTGCCCCTTCCCCTGAGGATGGGACTTCCCCTTCTCCTCCCCCTCTGCCACCACCACTTCCACCACCTCCTCCCCCAAGTTGGCGGGAGTTCTGTGAATCCCACGCTCGGGCAGCAGCCCTAGACTTTGCCCGACGCTTCCGCCTCTACTTGGCTTCCCATCCCCAGTATGCAGGGCCTGGGGCTGAGGCAGCCTTCTCCCGACGTTTTGCTGAGCTCTTCTTGCAGCACTTTGAGGCTGAGGTGGCCCGGGCCTCAGGTTCCCTTTTATCATCTGGTCCGATCCCCCTGAGTCCCAGTACGGAAACCCCATCACGTGATCTTTCCCTGGAGAGCTGCAGGAACAGTGGGCCTCTGACTGTGTTGGGTCCTTCTCGATCCTCTGAAGACCTGGCTGGCCCTCTCCCTTCTTCAATCTCTTCATCCTCCTCAAAGCCTAAGCTGAAGAAACGTTTCTCCCTCCGCTCTGTGGGCCGATCAGTGCGAGGCTCGGTCCGAGGGATCTTGCAGTGGCGGGGAGCTGGTGATCCCCCCTCTCCAGTGGGACCCTTGGAGACCTCTGCCTCTGGACCACCTGTCCTGGGTGGAAACAGCAACTCCAACTCCTCTGGTGGGGGTGGTAGCACCAATGTGGGGCCAACTAGCGGTGGGGCATCCCCTGGGGAAAGATGGACACACCGCTTTGAGAGGCTGAGACTAAGTAGGGGAGGGGGACCCTTAAAGGATGGGGCAGGGACAGTGCAAAGGGAGGGGCTACTGAGCTTCATGGGAACAGAGGAGGCAGCTGTTGGTGGAGGAGAAGGTCGAGGGGGAGGAGGAACTGGAACCTCATCTGGGGGTGGGGGACCACCAAGGTGGCAGAAGTGTCGCCTGCTGCTTCGGAGTGAGGGGGAAGGAGGAGGAAGTCGGCTGGAGTTCTATATCCCACCTAAGGTGAGAGAGAAGGGGAAGTGTGACTGGGGATGAATAATGTAGAAGCCTTGCAGATAGAACCCCAGAAGTTTACAAGCCTACAGGCTTTTACTTTTTTTTTTCCTTTGACACTTGGACCCATCTTGCTAACTCTTGACACTTGTTGAGTATGCAGGACTAGGTTGGTGATATCAACAGTCTTTCTGCCACCTCCACATTACTTACAAGGTAGAGCAGGGAGCCATTGGGCTTGTGTTGACACCCCTTTGGTGAAGTGGGAAGAGTTGGGAGGCCAGGTTTCCCCTGGGAAGTGGAGGAGTGTTTTTGAGCCTGAATATTGGGACTCCTACTGCCTCTTCTACCGCAGGCATCCCGGCCCCGGATCAACATCCTGTGTTCTACCGTCACAGATGTCCGGACGACCACAGCCCTGGAGATGCCCGACAGGGAGAACACGTTTGTGGTTAAGGTGGAGGGTCCTATGGAATACATCCTGGAGACAGCTGATGCCCTGAGTATGAAGGCCTGGGTGTCTGATCTACAGGATTGCCTGAGTCCAGGGGAGAACACAGACAGCCTGGACCTGTCCTGCCTCAACCACTCAGAGAGTCTGCCCAGCCAAGAGCTGCCCTTGGGACCCAGTGAGAGCAATGAGCATCTGTCTCAGGGGGCGTATGGGGGCCTCTCGGATCGTCCCTCAGCTTCTGTCTCCCCCAGCTCTGCCTCCATTGCTGCCTCCCATTTTGATTCAATGGAACTACTGCCCCCAGAGTTGCCCCCCCGAGTCCCTATTGAAGAGGGGCCCCCAGTGGGGACAACTTACCCCCTCCCAGCCCCTTACCCTCCTCCGGACACTCCAGACACCACAGGTTCGTTCCTGTTCCAGGGGGAATCAGATGGAGGTGATGGGGACCATCCCCTTTCAGAGTACCCTTGGTTCCATGGGACCCTTTCCCGACTCAAAGCTGCCCAGCTAGTGTTGGCAGGGGGTGCTGGCAGCCATGGTGTCTTCCTGGTACGTCAAAGCGAGACAAGGAGGGGGGAGTATGTCCTCACGTTCAACTTCCAGGGCAAGGCCAAGCACCTTCGTCTGTCCCTGAATGAGGATGGTCAGTGCCGGGTGCAGCACCTCTGGTTCCAGACGGTCTTTGACATGCTGGAGCATTTCCGGGTCCACCCCATTCCCTTGGAGTCTGGGGGCTCCAGTGATGTTACCCTCGTCAGCTATGTTGTGGCCTCTCAGAGGCTACAAGGCAGGGAACGGGCAGGGAGCCGAGCTGGGGTGTGTGAGGGAGATCGCTGCTACCCCGACGCCTCTTCCACCCTCATGCCCATCGGAGCGAGTGACTGTGTAACAGAACATCTCCCATGACCCATCCCAATCTCCTGATCCCGATCTGAGGGCAGACCCTGCACACCCTGGGGAAGAGGAGGAGATGGTGGAGGCATCAGAAGCTTCAGAGGAAGAGAAGGCAGGCAGTGGAGGGACCCCAGGAGAGCCGGGCCCTGTGGCTGAGCTTGAGGAGACCACAGCCCTAAACAGGGAGTCCTCAGGGCTAGAGGGGCCTGGTGGGGAGGTGGGAGTGACCCCTTTTGTGCATTTCTTGCAGTTGCAGCCAGGGAATGCCGGGGAGGAAGGGGTCTGCTCCAGAGCCATCAATAACCAGTACTCCTTCCTATAAGGATGAATACCCCCACACAACCAATTCCCCCTTTCTCCCTGGCCCTGGGCCAGGACCCGTGACAGATCCTCCTCAGAGAGGTACAAATGCTTCCCTTGCCCCTATTTGTCTAGGGGGTAGGAATAGTACAGGACAGAGGGACAGAGCCATTAGTAACTTCCCAGAGGACACACTCCAAGCTGCCCCTTTCCCTGGGCAGGGGACTTGGATCCGTTACCTCCTTTTGGGGGTGGGCCAATTGTGCTCAGCCTCGCCCCCTGTGGGCCATTTCTCCAATACCTCTCACAGCTCTCTGTGATGTATTGGGGAGAGGCTGTATCAGCTACATTAAGGATTTTTTTTTTAAGTGAAATGGAGAAACAAA

>UoN.Pci.T.22728_UoN.Pci.T.22728.1

GGCCGAGTCATATGACCGCTCTCTCGGCTTCCTGGCTCCGGCCGCCGCCGCCGCCGCCGCCTCTGCGCGTAGCCCTGTGTGTGTCCGTGTCCCCGTCCCCGTCCGCGTCGCTTCCCCGGGCCGGCCGAGCCGTTGGCGTGGGGGATCCGTGTCGCTCGCGTGCAAGCCGCCCGCCCCCGCCATCATGCTGGCGCTCATCTCCCGGCTCCTGGACTGGTTCCGCTCGCTCTTCTGGAAGGAGGAGATGGAGCTGACGTTGGTGGGGCTGCAGTACTCGGGCAAGACCACCTTCGTCAATGTCATCGCGTCAGGTCAATTCAGTGAAGATATGATCCCAACAGTGGGCTTTAACATGAGGAAGGTAACTAAAGGTAACGTCACAATAAAGATCTGGGACATAGGAGGGCAGCCACGATTCCGAAGCATGTGGGAACGATACTGCAGAGGAGTCAATGCGATTGTGTAAGTGGGGTGTGGCCACGGGGATCTTTCCAGTTTGTTCTCTTAGAAAAATTCTTGTAGCACTGGATTCAGTCAGCCAGCATTTAGTAAGTACTTACTCTGTGCCCAAGTACTTACTGTATGCTGGGTCAGGCCCTGGAGATGCAGGCCCTTGAGACGTTTACAAGTCCAGTGGAAAGAGGGATTTTTTTTCGTTTTTTCTTTTTCCTGTCCAAGGTGGGGACCACCAAGTTAAAAGTCTTTTAAAAATTCATTTTAATTGAATTTTGAAAATTTAAAATAAAAGCATGACTTTTTATCATATTTAACTTTATCATATTTTACTTAAAAGAAAACTATGTATTAAAAACTTTAAATGACAACTATAACCTTAACTGCCACTTAATTGGGTGCATATAAGTATCTCTGAGGGTGCTAAATCTGACAGGCATCCAGGTGTTGCAGTAGATAGAGTGCAAGGCCTGGAGTCAAGAAGACTCCAGTTCCAGTCCAGCCCCACACACTTCCTAGCTGTGGAACCTTGGGCAAGTTACTTAACCTGTCTACCTCAGTTTCCTCAGCTGTTAAAAAGGGATAATGATAGTACCCATCTCACAAAGTTGTTGTGAAGAAGAGATGAGATAATATTTGTAAAGCACTTAGCACAGTACCTGACACATAGTAGGTACTATAAAAATGCTAGCTATTATTACTGTTATTAAATTAATTAAATTAAATCTTCAAGATCAGATTTTTGCCCTGGCTCTTCCCTTCTCTGGGCAGTCCTGATCTCGTCTATTCCCCCTCTCCACCCTCTTCCTCCTTTGGTCTAAGGTTGAACTGTGGGACCATTTTTTAAGGAAGTGAGACTCTTAGATGTTATTATTAAGAAATTTTGCTCTCAACTTTTTAAGTTACATGGTGGATGCTGCAGATCGTGAAAAGATCGAAGCTTCTCGGAATGAGCTACATAATCTTCTGGACAAACCACAGCTACAAGGCATTCCAGTGCTAGTACTTGGAAACAAGAGAGACCTGCCTAATGCCTTGGATGAGAAACAACTAATTGAAAAAATGAATCTCTCTGCTATTCAGGACAGAGAAATCTGCTGCTATTCAATTTCTTGCAAAGAAAAGGATAATATAGACATCACGCTTCAGTGGCTTATTCAGCATTCGAAATCTAGAAGAAGCTGAGGTCTTCAGCCCCTGGAGTCCCTCCCCCCACCCCGCTGGCTGTGATCCCAGACTTGTCGTCCGTTCCTCTGACGTGCTACCCAGAATATGGTCCTTCCTACATCCAAGAAATGGCCTTTTGTCTGAGTTAATTTCTCCTGTGCGCTGCTGGAGATATGTATCCTTCACCTGTCACAAAGCATCACCTAGAGTTGTCATGATAAAGTCAGCACAAAAAGGCTTGTCACAAACAGCACACAGTGCTGTTGAAGCAGTCTTGACCACCTTAAATCAAGGAAATTGCATATTTCAATTTGGGTCATTCTGGCCAGATTTTTATATTGATTTTCAGCAAGTGTTTACGATATTTAATTAGATTTCAGTAGCTTGATACTGAGACAGCATGAGGATTCTAGCACCACCTACAGTATTTAGAAACCCTTTAAGCCTGACTCCTGTGGGATAGAAGCCTATTGTTTCTTTGTCTTATCTCACACATGCATGTGAAATGTATATTTTTAAGCCATAGAAATGGAAATTGTCTGAAAAGGGGGCACAAGGCCCAATGAGGTCAATGTCACCGTTTCTCCTCTCTACTGGTGAGATCTTACCCAGGGACTTTTAGAAATGAACTCCTTTCTGGTGATGCTTAATAGCCACGTACATAGTGGTGGGATGATGTGGGAGAAACTAAGGCATGCTGTTTTGTAGCTGTCCAGATCATTACTTGTATCTTTTTTTTTTGGTTTAACACTTTTTTAAGGGGATGGGAATTGACTTTCTCAAAAACTTTCAAGGGTTTAGAACATTCCAGTTAAATAATAAAACTTATGAAGAATGTTACTTTGTTCAAAGCCCTGTATGTTTTTCTATCTACATGTCATTAAGTCAGTGCTGCTGCATGCCTCTCAAACTATTGAATTTTTATATCCAGTCAGAATAGGACTTTCAGCACAAAAGATACCTATAAAAACTGTCATGTTTTGTCTCTCTTACTGATTTCAGACCTGGCACCACTCTGCCTGGTTCCCCCAGACATCTTGGTTATTGTGACAACTCTAGACCTGCTTGCTTTTGTAATGTAATCATTCGCCACCTGGACAGGTTGTAGGACATCTGAGACAGAGGTGTCTAAGCACAAGAGAGCTGATTAATTGATCCTGTTAATTGATTCACTTTTAAAAGATAGAGGTGGGTGGGTTAAAACTAAAAATGGACTTGTTTTTGTAGCTTAGTGATCTCTGTATATTTAAAGGGACACATATGTTAATTCTGGTTCATTCGTAAAATATTGATGTAGACCACTGTAGGGGATAACTCTCTGGTACACCTGCTTATGTGTTGCCTCACACTGTGTGTGATTTTTTTTTTAGCAGCACTTAAATATAGACAGTGCATTTCCAAAATCGTCGAGCACTCAAAGTGTGGTCAGCAATGACTTCTTTTAATAAGTACCCGTTTTAATTTATAGACTGCCATGGCCTTAATATTCTGTACGAACTACTGCACCATGTTGCACAGACACTACTTGGTTTCTCTCCATTTACTTTTTTTTTTAAGAGAAAAATTGGGGCTTTATGAAATCGTCCTCTTTTTTTTCCCCTTAAAGCATGTTGCATATTTCTTGGATGATTTTGGAGGGGTGGGGTGGGAGGGTTCTGTTAGATATGGGG

>UoN.Pci.T.23023_UoN.Pci.T.23023.1

GCCTCAGCAGTGGTGCAGCCTCCTCCTCCTCCTCCTCTGTCTCCCCTCTGGGGGCACTTAGGTTGGTATCGGCCCTCCCTGTTCTGGGCCGGGGGCTCCAGCGCTCTAGTCGGAGTCCGACCCTGCGCCTGCTGCGTCTCTATTCGGTCCTCCTCCTCCTCCTCCCCCTCCTCCCCCTGCCCCTCCTCCTCTTCTAGCCACCGCCTCATCCTCCTCCTCCTCCTGCTGCCCGGCCGCTGGTCTATGCCCCACCCCTGAGCCCGCAGCAGTCCGCGACATGTCCACCCCGGCTCGGCGGCGCCTCATGCGGGACTTCAAGAGGCTGCAAGAGGACCCCCCCGCCGGGGTCAGCGGAGCCCCGTCGGAGAACAACATTATGGTGTGGAACGCGGTCATTTTCGGGCCCGAGGGGACCCCATTTGAAGATGGCACATTTAAACTTACAATAGAATTCACAGAAGAATATCCAAACAAACCACCTACAGTAAGATTTGTCTCTAAAATGTTTCATCCAAATGGTAAGTATAGTGATCATTTTACCTGTCTTTCTTGTGGGAGGGTACTTATCTGTACAATGCATTCCAGCTGGTTTTGCTTGCTTCCAGTTCAGTAAAAGCAGAGTATTTTGTTGAAGGGGTGGGTAGGGGGAAGTGAAATCACTTCCTAGACCAGCAGTCCTCAGACCCTAAATTCTCCCTTAAAGGACTTGAGGTTTTGCAGGCCAAAGTAAGGTTTTGTCTCCAGAGTAAGAGCAGTTAAATGGCTCTTGGATGAGGCTTTCCTAGTAGTTGGGCAAGCTCCCTCTTAGGCACAGTGGGATCAATATTTATTCTCTATAAGCTGAGTCACAATCCCTGCAGTTTGAGTGTGGCTATGGCAACAGCAGCAGAACAGTTGAGGGATTATGTTATTGGGGGGAAGGAGGGGGGAGAGTTAAATTGGGGAAAGACCTGAAATGGCTTAAGAGCAACCCTTCTTTTTCACAGCACAGCCAGCTTTGGTTTGAAAAATGTTTTTCTATTATGATCGTCCTTTAAGGACATCTTGTGAGTTCTTAGTTCTGACACTTTACTCTTTTATCATAATTATCCGTTGGCATATTTTAACATCATTTGCCCTTCTGGCCTTTTCCTTTTCGGAGCGAGGTAGATGATATCTGCTTTCTCCTGGAGACAGAGATGTTTTCTCTTACCTGACCAGTATCCCACTGCTTAGTCAAACATCCCACATGAGGGAGGCCTGCTAGAATCACAGAGGAGCTGAGCTGTGATAAAATGCTGAGCTTGTTGGCCAAGGCCACACAGGCGAGTGCCTTTTAACATGGCCTATTAAAGAACTACACCCAACTTTGTGTACACTGTCAGGGTCATAATAGCAGTGGGTACAGTATGTGTACTGATACAGCTATGCTTTTAGTCTATGCAGATGGTAGTATATGCCTGGATATACTTCAGAATCGTTGGAGTCCAACCTATGATGTTTCCTCCATTTTGACATCCATACAGTCTTTACTGGATGAGCCTAATCCCAACAGTCCAGCAAACAGCCAGGCTGCTCAGCTATACCAGGAGAACAAGCGAGAATATGAAAAGCGAGTTTCGGCAATAGTAGAACAGAGCTGGCGTGATTGTTGACCCAGGATGATACAAACGAACAGTCTGGTCATAAGGAAAAAATCCTGTCTGTCCCTTGCCTTCTTTTCCCATTAGCATCATTACCTTAATTTTGTTGAAAACAAAACAGCTGTTACGCTGTTGCCACCCTTCCTTGCTGAAGCTTTCTTTCCTCCCTCCCCTTCTGACATCTGAGAACACCTACTACAAAGTCAAATGTATTGTACTTGGGTTACTTGTAAAAGAATTACTAATACTGAATTCCTTTTCTGTGGTACTTCAACTCCAATTTTAGGATAGTATCGTACATTAACCGTACTTTCCGCTAAGCGTTTAAAATAATGGTTATACACAAGGGTACTTACTTATGTAGCTTCATAACATATTTCTATGCACACATGCACATTAAAAAAAAAACTCAATTGATTAAAGTGATGGAAAATTAGTCCAGGCTGGTCTTGGGAGCATGAGTTTGAAGCACTTACTCTACCTAGAGTTTTCCCACTTGGGAAAACTTGATTCCATGTAAAGTAATCCATTTATAAAGTTGGTCTGGGCTATTCCTGGGAGATGGTACGTTAATGACAGGAAGGAAAAATATTGTTAAATAGGAGCATTGCTTAAATAAGAAAAACAAACCCCACCCACTCACATTTTCAGGTATTAACTAGAGCTATAACTGTGGTCTAGTAACTAGGTATGACACTGAAGGGAAAATCTGTCGGTTTTGAGGTCGGGAGGTAAAGAAACTCCTACTCTGGTTTTTATGTTGGTTGAGAACAAGAGATCCATTATTGACTTTGGAGGATCGAAGCTGTCCCTACAATTTTCTCTCTCTCTTCTGAGTTTAAATTACCCAAAGAATGAAGTACCTCTTGGCAAAAAGCATCTTGCTTCTTCATATTTGCCCAATCCAGAGCTCCGTATCAAGCAGTAAGTATGTGTTAGCCCCTTCAGATTCACGACAGCCTCCTCTTTTTGGTTCAAAGTCCAAAAACCCTTCAGGCTAAGGCTGTCTGGACTTTGTGACAAATGATACCTTTATTAGGACAGTGTGGGACACAGCTTATAAATGTTCTGTTTATGTCCATTGTCATCTTTTGTGCAGCTCAGTCTGTTCAAGTAATTTTGCACAAGTAGCCCATGTAAAAAGTGTACATTTTCCCAGAAATTGTAAATAAAAAGTAACCTTAAATTTAAGGCAAGCTCTCCCAGTCCTACTTTGTTAGCTTAATATGACTTCACAGTGATCAGAAAGTTAGCAGAATCTTGGTACCTTCCCAAGAGCCTAGTTATCGCTTAAAATCAATACCAACATCAAAATTTGACATATTTCTTTTGGAGAGGGGAAATAACATCAGCATAAGTAATAGCAATTTAAAAAAATTAATATGTACAGATGAATCTTAGAAAATATAAACTGTCAGCATAAAATTATATTGGGAGGCGGGGAGAGGGTATGTAAAATCAATCTACTAGGCTAAAAATATGGGCAAACTCTAATCTTACATAAAGCTGGGACAACAGGAACATTATTCCAGGAAAGTAAGACCTCTTTCTCGGTGTCTTTACGTCTTGTGGCCTTTTGGTGGATACTGCCATTAAAGAACATGTGGAGTGTCTTAAAAGGTCTTAGTGCAGTTTGAAGCTTTAGTAACGTAACATACTGTATTTTATTTTCACAATCAAAGGCATAAATGTCATCATTTCAGTGAAATTGAAGGCCTGGAGAGTTCTCTGGAAACCATGAGAGCACCAGTACCTCTAAGAAGTCATATAAACAGCTAGGTGTGTAGTGGATAGAGCACTTGGCTTGGACTGAGGAAAAATCATGTTTTTGAGTTCAGATCTGGCCTCAAATGTTTACTAGCTGTGTGATCCTGGGCAAGTCACTTAACCCTGTTTGCCTGTTTCCTCATCTGTAAAATGAGCT

>UoN.Pci.T.23059_UoN.Pci.T.23059.1

GGAGAGGCCCTGAGCAAATCAGATCGCAGGAAAGAAACTGCCGGCTTTCAAAAATTCTCCCTCGCCGCCATCATCGATCGGCGGCCTGGAGGCGCAGGTGCTGTTGGCGGAGACTGGGGCGAGACGCGTCCTGGGGAGGACGTTTGACAAAGCGGCAGAGACCATGGCATCTGAGGATATTGCCAAGCTCGCCGAAAGCCTGGCCAGAACCCAGGTGGGCGGAGGACAGCTGAGCTTCAAAGGCGAAAGCCTGAAACTGAACACGGCAGAAGATGCTCAGGAGGTGATTAAACAGATAGAGGCGTTCGATGGGCTGGAGGCCCTGCGTTTTGAAGGCAACACAGTGGGTGTGGAGGCGGCCAAAGTCATCGCCAAGGCCCTGGAGAAGAAATCGGAGCTGAAGCGATGCCACTGGAGCGACATGTTCACCGGGAGGCTGAGGTCAGAGATCCCTCCTGCCCTGATCTCCCTGGGGGAGGCCCTCATCACAGCGGGGGCGCAGTTGGTGGAACTGGACCTGAGCGACAACGCCTTTGGGCCGGACGGCGTGCGAGGCTTCGAGGCCCTACTCAAGAGCTCAGCTTGTTTCACGCTTCAGGAGCTGAAGCTCAACAACTGTGGCATGGGCATCGGTGGTGGCAAGATCTTGGCAGCAGCTCTGACTGAATGCCACCGGAAATCCAGCACCCTGGGCAAGCCGCTGGCCCTGAAGGTCTTCGTGGCCGGCAGAAACCGCCTAGAGAACGATGGTGCCACCTCCTTGGCCGAAGCATTTGGGATCATCGGGACCCTGGAGGAAATCCACATGCCTCAGAATGGGATCAACCACCCCGGGGTCACGGCGCTCGCTCAGGCCTTTGCCACCAACTCCCTGCTGAGAGTCATCAACCTGAACGACAACACCTTCACTGAGAAGGGGGCAGTGGCCATGGCCGAGACCCTGAAGATCCTGAGACAGGTGGAGGTGATCAATTTTGGGGACTGCCTGGTACGTTCCCGAGGGGCCCTGGCCATCGCCGAAGCCGTGAAGGAGGGGCTGCACAGGCTGAAGGAGCTGAACTTGTCCTTCTGTGAAATCAAACGAGACTCCGCACTGGCCATCGCAGAGGCCGTTGAGGACAAATCAGAACTGGAAAAATTGGATCTCAATGGGAATGCTTTAGGAGAGGAAGGCTGTGAGCAGCTTCAGGAGATCTTGGAAGGCTTCAACATGGCCCACACACTGGCCTCACTCAGTGATGATGAAGGAGATGATGAGGAAGTGGAGGAGGAAGAGGAAGTGGAGGAGGAAGAGGAGGAGGAAGAGGAAGTTGAAGTTGAGGAAGAGGAGGAGGAAGAGGAGGAAGAAGAGGAGGAAGAGCTTCAGCAAGGCCAAGGAGAAGTGACCACGACCCCAAAGAAGATTCTGGATACACACGTTGGGGAACCTGCTTCCGTGACGTGTTCTCCATCCGACATCTCCACATTCCTCGCTTTCCCGTCTCCGGAAAAGCTTCTGCACCTTGGGCCCAAGAGCTCCATGTTGATAGCCCAGCAGACAGATACATCTGATCCAGAAAAGGTGGTTTCGGCTTTTCTGAAGGTCTCCTCTGTGTTCAAGGATGAGCTGGCAGTGAAGACAGCCGTTCAGGAGGCAGTGGGTGAGTGCGCCCCCAGCCTGGCGCAGGCTGGCAGGGGAAGGGAGAGCTTCTGGAAGGTGTCTGACTGCCTGGCGTGGCTCAGGCTGTGCCCGTGGGCACAGAACAGCTCCCGGCTCCGGTGCTTTGGCTACAACCCTGATCCTTACGCCCATTCTCTGGATGAGGAAGTTGAGGTGTGAGTCCAGGAATAGCAAGTGTGGAAGCCACAATTTGAGCCTGGGTCTTGGCTGCCTCTCGCTCTAGTGATCTTTCCACAAAGCCAGGACAGAAGCCTGGCTGGAGAAATAAGGCCCATGCCTTATTCCCAACGTCATGTGGTCTGAGGGTGGGCCCTGTGTGCCCCTGTCCTCCCCACAGTGCCCCGCAGCCAGGAATTCCGTGGTTCCCTAGCCAGAGAGTGCGTGGCCTTCTGGGCCGGGCTAGGCCTGGAGCGAGCTGCTCCATCAGTGGAGAGGAGCCAGGCCCGCGGGCCTGAGGAAGAGGTGAAGTGGTGGAAGGCAGGGGTCTTGGGGTAAGGTGTCGGCAGCAACTGTTAGACCTCGGGCAAGGCCTTTCTGCACCTTCCAGCTGCTAGAGCAATGGGCTCCCGAGCTGCTCCTTGGAGAAGAGGCCAAGCACTCGCTGGATGGCTTCCTGACTCCCAGCACCTCATCCCGCCTGCCTCAAAGCTGAGTGGGGTAGCTTTTGGGGGGGATGCAGGAGACGGCCGTGTCGAGACTAACGTGATTTAAATGCCAAGTGTTTGTAGAGTGGGAGATAAGGGGGGAGGATTTGAAAGGTAGACAGGGACTATGAGAGGAGCCCATGGGCCCAGTAGGCAGGGCCTCACCAGCAGCCACCAGGATGGCTCCAAGCCTACTGTATGCCTTGGGAGGGGCGGGATAGGAACTGTGACCCTGGAGCAGCCTGTGCCCAGGGCCCCTTTCTCTTCTCCGTGTCCTCCCCTTTGCCCATGTGGCCCTTAGGCGGCTCCTCTCCCTCTCCTGTGAGCTGCCCTCAGTGCTGCTGGAGGCTTTCGGGGCCAAGACTCCCCTGTGCTAGCTAGAGTGTGGCCCCTCCCGGGGGCCCCCACCCAAGGCCCCAGGATGCCTCTCTGTTCCCCTCTTCCAGATGCCCTGATGAAGAAAGCCTTCGATTCAGCCGCCTTCAACTCCAATGTGTTCATCACCAGGCTCTTAATACACATGGGGCTTCTCAAGAGCGAGGATAAGATCAAGGCAATCCCCAATCTCTACGGCCCCCTCATGGCCCTCAACCACATGGCCCAGCAGGACTACTTCCCCAAGACCCTTACACCCATCCTCCTGGCCTTTGTGACCAAGCCCAATCGGGCCCTGGAGTCCTGCTCCCTTGCTCGCCACAACCTCCTGCAAACCCTCCACCACGTCTAGTCGCTAAGAGGCCGGGCCTCTGCTGTCGTTCCCCATGAACGTTATGACCTGAGAGCCCCTGCCCAGGCCCAGAGGACGAGTGTGTGTGTCTGGCGTCACTTCCACTGAAGATACCCTTCCCCTCCTCCCCCCGGGACGCCGAGGGGACGCTGCTTCTGTTTGGCCTGCCGGCCCTGGCCGGGCCGGGCTTGTGGGATTTTTAATGACGTCTCTGTTGTGAATCACCTCGGCCTGCCTCAGGGGAGCTGGCCTCGGACACTTGCTTCCACCCTGCCTGCTCGCGGAGTCAGTCAGTGGCCCCTTCATTAAACGCTGTTCCCTGCCCTCACCTCACGCCGGCCTGCCTGGATCTCCCCTGGCCATCGCTGCCTGTTCTGGGTGCAGCGTGGTATCCTTAGCTGGCTCGGGGAGCTCCTTGGCCACTTCCTCTCACTAGGAATCTCGGGAAAGGGGAGCAGCCTTATCTCTGCCTCCCAGGCTCTGCTCCCGGGAGGACCCCGGAGCACAGTGTGGGAGGCAGACCTGGTGACCCCAGGGCCTGGCCCTTCCTAGTCAGTGACTCGGGGGCCTGTGCCCTCCCAGAGGAGCAGAAGTAGGCAGCACCTGGGTGCCTACTGTCTCCTGTGGTAGCACCAGCCTGGCCTCAGACCTTCTGCTCCGTGGAAGGAGCTGACGGGCTGGGCGCCCTCTGCTGTCCTGGGGCCTGAGCCCCGGCCCTTCGGCGGGGTGCTCAGGAAGGAGCCCTGCCCCACCTGGCTTCTTGGGACTCTGGACTGGAGCATTCTCAACGGCTCCTCCCCCTCCCCTATTGGAGACAAGAGACTTGAGCGAGGAAAGGTGTCCACTGTTACTTGTCTCCCTTCTAGCCCAGGCCTCGGTCCAGTCACCTTGTTAGCTGGTCACAGGGCAGCTGGGCCTGTTGGGAGTGCAGTACCCCCAGGGCCCTGAGCAGCCGTTTTCTTCTGGGCCTCAGTGGGTTCCAAGATGGGCCTGGGTCCCCGTGGCTCTTAACCTTTTCTCTCAGCTTGCTGTGCCTGGGCCCTGCCCAGGGCTTCACTAATGGACCAAAATGCTGAGGAGGGAGTTCAGGGATCCCCCAGGTTGCCCACTCTTCCCTTCACCATAACTTAGGTTTATCTTCTGTTTGGCACAGGGGGGTCCTGGGAGCAGGCACTGGGCTCAGAGCATGTCTGCAGGGTCCTGAGCTGGTCTCTGGAGGAGCTGCCGTGCCCTGCCTGTGGTGGTCCCCCAGCTAGCACTGGCTCTGGGGGCCATGAGATGCCCATCCTGCTCTCACCTGTCCCTGCTGTCCTGTGAGCGCTGGCAAAGGTTTGGAGTAGGTCTTTGTCCAGCTTCTCTGGCCTTTACCTGGTTTTAGCCAGTGTCCATAGGAGTGCTTGTGCCAGGGGGTGGGGGGGTGGCCCTCCTGTCATCATGGTGAAGGTCCGGGAGGCCTCGGTCTCCCGCTGTTCTTCCCCACGGTGGCAGAAGGAGTGGGCCCTACCCTGGTTGGCGTAACCCGGAACAGGGCCAGCGAGAGGAATGGGGAGGAGGCAGGGCTGGGCCCCGTTGGCCTCTTCAGCTGCCTGCTTGACCGTAGGTGCTCGTGTCACGTCAGTCCTCTGCCTGCTCAAAGGGGGTGTTAGGGCAGAGAGGCCTTGGGCCTGTCTAGAAACCCCAAGGAGAGGCTCTGCAGCTGCCCAGCTGTGTGGGCCCAGGGGCTGTCTTCAGGGCTCCGGTGGGTCAGAGGAGGAGGGAAGGCTACAGTAACTCCCCCCAGTGGGCCTAGGCTAGTGTGGCTTTGGAGCTGAGGAGGCCAGCTCTGGTAGAGCCTGCCCCCAGGGTGCCTTTCGCCCCGGTACACCCGCCCTCATCCATGTTGGTACTTGTGCTGATGTGTGAGTAACTGCCCTGCCTGGGCCGGGGCTTAGGGTAGTGTGCAGAATAAAGTGCTG

>UoN.Pci.T.23059_UoN.Pci.T.23059.2

GGAGAGGCCCTGAGCAAATCAGATCGCAGGAAAGAAACTGCCGGCTTTCAAAAATTCTCCCTCGCCGCCATCATCGATCGGCGGCCTGGAGGCGCAGGTGCTGTTGGCGGAGACTGGGGCGAGACGCGTCCTGGGGAGGACGTTTGACAAAGCGGCAGAGACCATGGCATCTGAGGATATTGCCAAGCTCGCCGAAAGCCTGGCCAGAACCCAGGTGGGCGGAGGACAGCTGAGCTTCAAAGGCGAAAGCCTGAAACTGAACACGGCAGAAGATGCTCAGGAGGTGATTAAACAGATAGAGGCGTTCGATGGGCTGGAGGCCCTGCGTTTTGAAGGCAACACAGTGGGTGTGGAGGCGGCCAAAGTCATCGCCAAGGCCCTGGAGAAGAAATCGGAGCTGAAGCGATGCCACTGGAGCGACATGTTCACCGGGAGGCTGAGGTCAGAGATCCCTCCTGCCCTGATCTCCCTGGGGGAGGCCCTCATCACAGCGGGGGCGCAGTTGGTGGAACTGGACCTGAGCGACAACGCCTTTGGGCCGGACGGCGTGCGAGGCTTCGAGGCCCTACTCAAGAGCTCAGCTTGTTTCACGCTTCAGGAGCTGAAGCTCAACAACTGTGGCATGGGCATCGGTGGTGGCAAGATCTTGGCAGCAGCTCTGACTGAATGCCACCGGAAATCCAGCACCCTGGGCAAGCCGCTGGCCCTGAAGGTCTTCGTGGCCGGCAGAAACCGCCTAGAGAACGATGGTGCCACCTCCTTGGCCGAAGCATTTGGGATCATCGGGACCCTGGAGGAAATCCACATGCCTCAGAATGGGATCAACCACCCCGGGGTCACGGCGCTCGCTCAGGCCTTTGCCACCAACTCCCTGCTGAGAGTCATCAACCTGAACGACAACACCTTCACTGAGAAGGGGGCAGTGGCCATGGCCGAGACCCTGAAGATCCTGAGACAGGTGGAGGTGATCAATTTTGGGGACTGCCTGGTACGTTCCCGAGGGGCCCTGGCCATCGCCGAAGCCGTGAAGGAGGGGCTGCACAGGCTGAAGGAGCTGAACTTGTCCTTCTGTGAAATCAAACGAGACTCCGCACTGGCCATCGCAGAGGCCGTTGAGGACAAATCAGAACTGGAAAAATTGGATCTCAATGGGAATGCTTTAGGAGAGGAAGGCTGTGAGCAGCTTCAGGAGATCTTGGAAGGCTTCAACATGGCCCACACACTGGCCTCACTCAGTGATGATGAAGGAGATGATGAGGAAGTGGAGGAGGAAGAGGAAGTGGAGGAGGAAGAGGAGGAGGAAGAGGAAGTTGAAGTTGAGGAAGAGGAGGAGGAAGAGGAGGAAGAAGAGGAGGAAGAGCTTCAGCAAGGCCAAGGAGAAGTGACCACGACCCCAAAGAAGATTCTGGATACACACGTTGGGGAACCTGCTTCCGTGACGTGTTCTCCATCCGACATCTCCACATTCCTCGCTTTCCCGTCTCCGGAAAAGCTTCTGCACCTTGGGCCCAAGAGCTCCATGTTGATAGCCCAGCAGACAGATACATCTGATCCAGAAAAGGTGGTTTCGGCTTTTCTGAAGGTCTCCTCTGTGTTCAAGGATGAGCTGGCAGTGAAGACAGCCGTTCAGGAGGCAGTGGGTGAGTGCGCCCCCAGCCTGGCGCAGGCTGGCAGGGGAAGGGAGAGCTTCTGGAAGGTGTCTGACTGCCTGGCGTGGCTCAGGCTGTGCCCGTGGGCACAGAACAGCTCCCGGCTCCGGTGCTTTGGCTACAACCCTGATCCTTACGCCCATTCTCTGGATGAGGAAGTTGAGGTGTGAGTCCAGGAATAGCAAGTGTGGAAGCCACAATTTGAGCCTGGGTCTTGGCTGCCTCTCGCTCTAGTGATCTTTCCACAAAGCCAGGACAGAAGCCTGGCTGGAGAAATAAGGCCCATGCCTTATTCCCAACGTCATGTGGTCTGAGGGTGGGCCCTGTGTGCCCCTGTCCTCCCCACAGTGCCCCGCAGCCAGGAATTCCGTGGTTCCCTAGCCAGAGAGTGCGTGGCCTTCTGGGCCGGGCTAGGCCTGGAGCGAGCTGCTCCATCAGTGGAGAGGAGCCAGGCCCGCGGGCCTGAGGAAGAGGTGAAGTGGTGGAAGGCAGGGGTCTTGGGGTAAGGTGTCGGCAGCAACTGTTAGACCTCGGGCAAGGCCTTTCTGCACCTTCCAGCTGCTAGAGCAATGGGCTCCCGAGCTGCTCCTTGGAGAAGAGGCCAAGCACTCGCTGGATGGCTTCCTGACTCCCAGCACCTCATCCCGCCTGCCTCAAAGCTGAGTGGGGTAGCTTTTGGGGGGGATGCAGGAGACGGCCGTGTCGAGACTAACGTGATTTAAATGCCAAGTGTTTGTAGAGTGGGAGATAAGGGGGGAGGATTTGAAAGGTAGACAGGGACTATGAGAGGAGCCCATGGGCCCAGTAGGCAGGGCCTCACCAGCAGCCACCAGGATGGCTCCAAGCCTACTGTATGCCTTGGGAGGGGCGGGATAGGAACTGTGACCCTGGAGCAGCCTGTGCCCAGGGCCCCTTTCTCTTCTCCGTGTCCTCCCCTTTGCCCATGTGGCCCTTAGGCGGCTCCTCTCCCTCTCCTGTGAGCTGCCCTCAGTGCTGCTGGAGGCTTTCGGGGCCAAGACTCCCCTGTGCTAGCTAGAGTGTGGCCCCTCCCGGGGGCCCCCACCCAAGGCCCCAGGATGCCTCTCTGTTCCCCTCTTCCAGATGCCCTGATGAAGAAAGCCTTCGATTCAGCCGCCTTCAACTCCAATGTGTTCATCACCAGGCTCTTAATACACATGGGGCTTCTCAAGAGCGAGGATAAGATCAAGGCAATCCCCAATCTCTACGGCCCCCTCATGGCCCTCAACCACATGGCCCAGCAGGACTACTTCCCCAAGACCCTTACACCCATCCTCCTGGCCTTTGTGACCAAGCCCAATCGGGCCCTGGAGTCCTGCTCCCTTGCTCGCCACAACCTCCTGCAAACCCTCCACCACGTCTAGTCGCTAAGAGGCCGGGCCTCTGCTGTCGTTCCCCATGAACGTTATGACCTGAGAGCCCCTGCCCAGGCCCAGAGGACGAGTGTGTGTGTCTGGCGTCACTTCCACTGAAGATACCCTTCCCCTCCTCCCCCCGGGACGCCGAGGGGACGCTGCTTCTGTTTGGCCTGCCGGCCCTGGCCGGGCCGGGCTTGTGGGATTTTTAATGACGTCTCTGTTGTGAATCACCTCGGCCTGCCTCAGGGGAGCTGGCCTCGGACACTTGCTTCCACCCTGCCTGCTCGCGGAGTCAGTCAGTGGCCCCTTCATTAAACGCTGTTCCCTGCCCTCACCTCACGCCGGCCTGCCTGGATCTCCCCTGGCCATCGCTGCCTGTTCTGGGTGCAGCGTGGTATCCTTAGCTGGCTCGGGGAGCTCCTTGGCCACTTCCTCTCACTAGGAATCTCGGGAAAGGGGAGCAGCCTTATCTCTGCCTCCCAGGCTCTGCTCCCGGGAGGACCCCGGAGCACAGTGTGGGAGGCAGACCTGGTGACCCCAGGGCCTGGCCCTTCCTAGTCAGTGACTCGGGGGCCTGTGCCCTCCCAGAGGAGCAGAAGTAGGCAGCACCTGGGTGCCTACTGTCTCCTGTGGTAGCACCAGCCTGGCCTCAGACCTTCTGCTCCGTGGAAGGAGCTGACGGGCTGGGCGCCCTCTGCTGTCCTGGGGCCTGAGCCCCGGCCCTTCGGCGGGGTGCTCAGGAAGGAGCCCTGCCCCACCTGGCTTCTTGGGACTCTGGACTGGAGCATTCTCAACGGCTCCTCCCCCTCCCCTATTGGAGACAAGAGACTTGAGCGAGGAAAGGTGTCCACTGTTACTTGTCTCCCTTCTAGCCCAGGCCTCGGTCCAGTCACCTTGTTAGCTGGTCACAGGGCAGCTGGGCCTGTTGGGAGTGCAGTACCCCCAGGGCCCTGAGCAGCCGTTTTCTTCTGGGCCTCAGTGGGTTCCAAGATGGGCCTGGGTCCCCGTGGCTCTTAACCTTTTCTCTCAGCTTGCTGTGCCTGGGCCCTGCCCAGGGCTTCACTAATGGACCAAAATGCTGAGGAGGGAGTTCAGGGATCCCCCAGGTTGCCCACTCTTCCCTTCACCATAACTTAGGTTTATCTTCTGTTTGGCACAGGGGGGTCCTGGGAGCAGGCACTGGGCTCAGAGCATGTCTGCAGGGTCCTGAGCTGGTCTCTGGAGGAGCTGCCGTGCCCTGCCTGTGGTGGTCCCCCAGCTAGCACTGGCTCTGGGGGCCATGAGATGCCCATCCTGCTCTCACCTGTCCCTGCTGTCCTGTGAGCGCTGGCAAAGGTTTGGAGTAGGTCTTTGTCCAGCTTCTCTGGCCTTTACCTGGTTTTAGCCAGTGTCCATAGGAGTGCTTGTGCCAGGGGGTGGGGGGGTGGCCCTCCTGTCATCATGGTGAAGGTCCGGGAGGCCTCGGTCTCCCGCTGTTCTTCCCCACGGTGGCAGAAGGAGTGGGCCCTACCCTGGTTGGCGTAACCCGGAACAGGGCCAGCGAGAGGAATGGGGAGGAGGCAGGGCTGGGCCCCGTTGGCCTCTTCAGCTGCCTGCTTGACCGTAGGTGCTCGTGTCACGTCAGTCCTCTGCCTGCTCAAAGGGGGTGTTAGGGCAGAGAGGCCTTGGGCCTGTCTAGAAACCCCAAGGAGAGGCTCTGCAGCTGCCCAGCTGTGTGGGCCCAGGGGCTGTCTTCAGGGCTCCGGTGGGTCAGAGGAGGAGGGAAGGCTACAGTAACTCCCCCCAGTGGGCCTAGGCTAGTGTGGCTTTGGAGCTGAGGAGGCCAGCTCTGGTAGAGCCTGCCCCCAGGGTGCCTTTCGCCCCGGTACACCCGCCCTCATCCATGTTGGTACTTGTGCTGATGTGTGAGTAACTGCCCTGCCTGGGCCGGGGCTTAGGGTAGTGTGCAGAATAAAGTGCTG

>UoN.Pci.T.23059_UoN.Pci.T.23059.3

GGAGAGGCCCTGAGCAAATCAGATCGCAGGAAAGAAACTGCCGGCTTTCAAAAATTCTCCCTCGCCGCCATCATCGATCGGCGGCCTGGAGGCGCAGGTGCTGTTGGCGGAGACTGGGGCGAGACGCGTCCTGGGGAGGACGTTTGACAAAGCGGCAGAGACCATGGCATCTGAGGATATTGCCAAGCTCGCCGAAAGCCTGGCCAGAACCCAGGTGGGCGGAGGACAGCTGAGCTTCAAAGGCGAAAGCCTGAAACTGAACACGGCAGAAGATGCTCAGGAGGTGATTAAACAGATAGAGGCGTTCGATGGGCTGGAGGCCCTGCGTTTTGAAGGCAACACAGTGGGTGTGGAGGCGGCCAAAGTCATCGCCAAGGCCCTGGAGAAGAAATCGGAGCTGAAGCGATGCCACTGGAGCGACATGTTCACCGGGAGGCTGAGGTCAGAGATCCCTCCTGCCCTGATCTCCCTGGGGGAGGCCCTCATCACAGCGGGGGCGCAGTTGGTGGAACTGGACCTGAGCGACAACGCCTTTGGGCCGGACGGCGTGCGAGGCTTCGAGGCCCTACTCAAGAGCTCAGCTTGTTTCACGCTTCAGGAGCTGAAGCTCAACAACTGTGGCATGGGCATCGGTGGTGGCAAGATCTTGGCAGCAGCTCTGACTGAATGCCACCGGAAATCCAGCACCCTGGGCAAGCCGCTGGCCCTGAAGGTCTTCGTGGCCGGCAGAAACCGCCTAGAGAACGATGGTGCCACCTCCTTGGCCGAAGCATTTGGGATCATCGGGACCCTGGAGGAAATCCACATGCCTCAGAATGGGATCAACCACCCCGGGGTCACGGCGCTCGCTCAGGCCTTTGCCACCAACTCCCTGCTGAGAGTCATCAACCTGAACGACAACACCTTCACTGAGAAGGGGGCAGTGGCCATGGCCGAGACCCTGAAGATCCTGAGACAGGTGGAGGTGATCAATTTTGGGGACTGCCTGGTACGTTCCCGAGGGGCCCTGGCCATCGCCGAAGCCGTGAAGGAGGGGCTGCACAGGCTGAAGGAGCTGAACTTGTCCTTCTGTGAAATCAAACGAGACTCCGCACTGGCCATCGCAGAGGCCGTTGAGGACAAATCAGAACTGGAAAAATTGGATCTCAATGGGAATGCTTTAGGAGAGGAAGGCTGTGAGCAGCTTCAGGAGATCTTGGAAGGCTTCAACATGGCCCACACACTGGCCTCACTCAGTGATGATGAAGGAGATGATGAGGAAGTGGAGGAGGAAGAGGAAGTGGAGGAGGAAGAGGAGGAGGAAGAGGAAGTTGAAGTTGAGGAAGAGGAGGAGGAAGAGGAGGAAGAAGAGGAGGAAGAGCTTCAGCAAGGCCAAGGAGAAGTGACCACGACCCCAAAGAAGATTCTGGATACACACGTTGGGGAACCTGCTTCCGTGACGTGTTCTCCATCCGACATCTCCACATTCCTCGCTTTCCCGTCTCCGGAAAAGCTTCTGCACCTTGGGCCCAAGAGCTCCATGTTGATAGCCCAGCAGACAGATACATCTGATCCAGAAAAGGTGGTTTCGGCTTTTCTGAAGGTCTCCTCTGTGTTCAAGGATGAGCTGGCAGTGAAGACAGCCGTTCAGGAGGCAGTGGGTGAGTGCGCCCCCAGCCTGGCGCAGGCTGGCAGGGGAAGGGAGAGCTTCTGGAAGGTGTCTGACTGCCTGGCGTGGCTCAGGCTGTGCCCGTGGGCACAGAACAGCTCCCGGCTCCGGTGCTTTGGCTACAACCCTGATCCTTACGCCCATTCTCTGGATGAGGAAGTTGAGGTGTGAGTCCAGGAATAGCAAGTGTGGAAGCCACAATTTGAGCCTGGGTCTTGGCTGCCTCTCGCTCTAGTGATCTTTCCACAAAGCCAGGACAGAAGCCTGGCTGGAGAAATAAGGCCCATGCCTTATTCCCAACGTCATGTGGTCTGAGGGTGGGCCCTGTGTGCCCCTGTCCTCCCCACAGTGCCCCGCAGCCAGGAATTCCGTGGTTCCCTAGCCAGAGAGTGCGTGGCCTTCTGGGCCGGGCTAGGCCTGGAGCGAGCTGCTCCATCAGTGGAGAGGAGCCAGGCCCGCGGGCCTGAGGAAGAGGTGAAGTGGTGGAAGGCAGGGGTCTTGGGGTAAGGTGTCGGCAGCAACTGTTAGACCTCGGGCAAGGCCTTTCTGCACCTTCCAGCTGCTAGAGCAATGGGCTCCCGAGCTGCTCCTTGGAGAAGAGGCCAAGCACTCGCTGGATGGCTTCCTGACTCCCAGCACCTCATCCCGCCTGCCTCAAAGCTGAGTGGGGTAGCTTTTGGGGGGGATGCAGGAGACGGCCGTGTCGAGACTAACGTGATTTAAATGCCAAGTGTTTGTAGAGTGGGAGATAAGGGGGGAGGATTTGAAAGGTAGACAGGGACTATGAGAGGAGCCCATGGGCCCAGTAGGCAGGGCCTCACCAGCAGCCACCAGGATGGCTCCAAGCCTACTGTATGCCTTGGGAGGGGCGGGATAGGAACTGTGACCCTGGAGCAGCCTGTGCCCAGGGCCCCTTTCTCTTCTCCGTGTCCTCCCCTTTGCCCATGTGGCCCTTAGGCGGCTCCTCTCCCTCTCCTGTGAGCTGCCCTCAGTGCTGCTGGAGGCTTTCGGGGCCAAGACTCCCCTGTGCTAGCTAGAGTGTGGCCCCTCCCGGGGGCCCCCACCCAAGGCCCCAGGATGCCTCTCTGTTCCCCTCTTCCAGATGCCCTGATGAAGAAAGCCTTCGATTCAGCCGCCTTCAACTCCAATGTGTTCATCACCAGGCTCTTAATACACATGGGGCTTCTCAAGAGCGAGGATAAGATCAAGGCAATCCCCAATCTCTACGGCCCCCTCATGGCCCTCAACCACATGGCCCAGCAGGACTACTTCCCCAAGACCCTTACACCCATCCTCCTGGCCTTTGTGACCAAGCCCAATCGGGCCCTGGAGTCCTGCTCCCTTGCTCGCCACAACCTCCTGCAAACCCTCCACCACGTCTAGTCGCTAAGAGGCCGGGCCTCTGCTGTCGTTCCCCATGAACGTTATGACCTGAGAGCCCCTGCCCAGGCCCAGAGGACGAGTGTGTGTGTCTGGCGTCACTTCCACTGAAGATACCCTTCCCCTCCTCCCCCCGGGACGCCGAGGGGACGCTGCTTCTGTTTGGCCTGCCGGCCCTGGCCGGGCCGGGCTTGTGGGATTTTTAATGACGTCTCTGTTGTGAATCACCTCGGCCTGCCTCAGGGGAGCTGGCCTCGGACACTTGCTTCCACCCTGCCTGCTCGCGGAGTCAGTCAGTGGCCCCTTCATTAAACGCTGTTCCCTGCCCTCACCTCACGCCGGCCTGCCTGGATCTCCCCTGGCCATCGCTGCCTGTTCTGGGTGCAGCGTGGTATCCTTAGCTGGCTCGGGGAGCTCCTTGGCCACTTCCTCTCACTAGGAATCTCGGGAAAGGGGAGCAGCCTTATCTCTGCCTCCCAGGCTCTGCTCCCGGGAGGACCCCGGAGCACAGTGTGGGAGGCAGACCTGGTGACCCCAGGGCCTGGCCCTTCCTAGTCAGTGACTCGGGGGCCTGTGCCCTCCCAGAGGAGCAGAAGTAGGCAGCACCTGGGTGCCTACTGTCTCCTGTGGTAGCACCAGCCTGGCCTCAGACCTTCTGCTCCGTGGAAGGAGCTGACGGGCTGGGCGCCCTCTGCTGTCCTGGGGCCTGAGCCCCGGCCCTTCGGCGGGGTGCTCAGGAAGGAGCCCTGCCCCACCTGGCTTCTTGGGACTCTGGACTGGAGCATTCTCAACGGCTCCTCCCCCTCCCCTATTGGAGACAAGAGACTTGAGCGAGGAAAGGTGTCCACTGTTACTTGTCTCCCTTCTAGCCCAGGCCTCGGTCCAGTCACCTTGTTAGCTGGTCACAGGGCAGCTGGGCCTGTTGGGAGTGCAGTACCCCCAGGGCCCTGAGCAGCCGTTTTCTTCTGGGCCTCAGTGGGTTCCAAGATGGGCCTGGGTCCCCGTGGCTCTTAACCTTTTCTCTCAGCTTGCTGTGCCTGGGCCCTGCCCAGGGCTTCACTAATGGACCAAAATGCTGAGGAGGGAGTTCAGGGATCCCCCAGGTTGCCCACTCTTCCCTTCACCATAACTTAGGTTTATCTTCTGTTTGGCACAGGGGGGTCCTGGGAGCAGGCACTGGGCTCAGAGCATGTCTGCAGGGTCCTGAGCTGGTCTCTGGAGGAGCTGCCGTGCCCTGCCTGTGGTGGTCCCCCAGCTAGCACTGGCTCTGGGGGCCATGAGATGCCCATCCTGCTCTCACCTGTCCCTGCTGTCCTGTGAGCGCTGGCAAAGGTTTGGAGTAGGTCTTTGTCCAGCTTCTCTGGCCTTTACCTGGTTTTAGCCAGTGTCCATAGGAGTGCTTGTGCCAGGGGGTGGGGGGGTGGCCCTCCTGTCATCATGGTGAAGGTCCGGGAGGCCTCGGTCTCCCGCTGTTCTTCCCCACGGTGGCAGAAGGAGTGGGCCCTACCCTGGTTGGCGTAACCCGGAACAGGGCCAGCGAGAGGAATGGGGAGGAGGCAGGGCTGGGCCCCGTTGGCCTCTTCAGCTGCCTGCTTGACCGTAGGTGCTCGTGTCACGTCAGTCCTCTGCCTGCTCAAAGGGGGTGTTAGGGCAGAGAGGCCTTGGGCCTGTCTAGAAACCCCAAGGAGAGGCTCTGCAGCTGCCCAGCTGTGTGGGCCCAGGGGCTGTCTTCAGGGCTCCGGTGGGTCAGAGGAGGAGGGAAGGCTACAGTAACTCCCCCCAGTGGGCCTAGGCTAGTGTGGCTTTGGAGCTGAGGAGGCCAGCTCTGGTAGAGCCTGCCCCCAGGGTGCCTTTCGCCCCGGTACACCCGCCCTCATCCATGTTGGTACTTGTGCTGATGTGTGAGTAACTGCCCTGCCTGGGCCGGGGCTTAGGGTAGTGTGCAGAATAAAGTGCTG

>UoN.Pci.T.23059_UoN.Pci.T.23059.4

CCCTGAGCAAATCAGATCGCAGGAAAGAAACTGCCGGCTTTCAAAAATTCTCCCTCGCCGCCATCATCGATCGGCGGCCTGGAGGCGCAGGTGCTGTTGGCGGAGACTGGGGCGAGACGCGTCCTGGGGAGGACGTTTGACAAAGCGGCAGAGACCATGGCATCTGAGGATATTGCCAAGCTCGCCGAAAGCCTGGCCAGAACCCAGGTGGGCGGAGGACAGCTGAGCTTCAAAGGCGAAAGCCTGAAACTGAACACGGCAGAAGATGCTCAGGAGGTGATTAAACAGATAGAGGCGTTCGATGGGCTGGAGGCCCTGCGTTTTGAAGGCAACACAGTGGGTGTGGAGGCGGCCAAAGTCATCGCCAAGGCCCTGGAGAAGAAATCGGAGCTGAAGCGATGCCACTGGAGCGACATGTTCACCGGGAGGCTGAGGTCAGAGATCCCTCCTGCCCTGATCTCCCTGGGGGAGGCCCTCATCACAGCGGGGGCGCAGTTGGTGGAACTGGACCTGAGCGACAACGCCTTTGGGCCGGACGGCGTGCGAGGCTTCGAGGCCCTACTCAAGAGCTCAGCTTGTTTCACGCTTCAGGAGCTGAAGCTCAACAACTGTGGCATGGGCATCGGTGGTGGCAAGATCTTGGCAGCAGCTCTGACTGAATGCCACCGGAAATCCAGCACCCTGGGCAAGCCGCTGGCCCTGAAGGTCTTCGTGGCCGGCAGAAACCGCCTAGAGAACGATGGTGCCACCTCCTTGGCCGAAGCATTTGGGATCATCGGGACCCTGGAGGAAATCCACATGCCTCAGAATGGGATCAACCACCCCGGGGTCACGGCGCTCGCTCAGGCCTTTGCCACCAACTCCCTGCTGAGAGTCATCAACCTGAACGACAACACCTTCACTGAGAAGGGGGCAGTGGCCATGGCCGAGACCCTGAAGATCCTGAGACAGGTGGAGGTGATCAATTTTGGGGACTGCCTGGTACGTTCCCGAGGGGCCCTGGCCATCGCCGAAGCCGTGAAGGAGGGGCTGCACAGGCTGAAGGAGCTGAACTTGTCCTTCTGTGAAATCAAACGAGACTCCGCACTGGCCATCGCAGAGGCCGTTGAGGACAAATCAGAACTGGAAAAATTGGATCTCAATGGGAATGCTTTAGGAGAGGAAGGCTGTGAGCAGCTTCAGGAGATCTTGGAAGGCTTCAACATGGCCCACACACTGGCCTCACTCAGTGATGATGAAGGAGATGATGAGGAAGTGGAGGAGGAAGAGGAAGTGGAGGAGGAAGAGGAGGAGGAAGAGGAAGTTGAAGTTGAGGAAGAGGAGGAGGAAGAGGAGGAAGAAGAGGAGGAAGAGCTTCAGCAAGGCCAAGGAGAAGTGACCACGACCCCAAAGAAGATTCTGGATACACACGTTGGGGAACCTGCTTCCGTGACGTGTTCTCCATCCGACATCTCCACATTCCTCGCTTTCCCGTCTCCGGAAAAGCTTCTGCACCTTGGGCCCAAGAGCTCCATGTTGATAGCCCAGCAGACAGATACATCTGATCCAGAAAAGGTGGTTTCGGCTTTTCTGAAGGTCTCCTCTGTGTTCAAGGATGAGCTGGCAGTGAAGACAGCCGTTCAGGAGGCAGTGGGTGAGTGCGCCCCCAGCCTGGCGCAGGCTGGCAGGGGAAGGGAGAGCTTCTGGAAGGTGTCTGACTGCCTGGCGTGGCTCAGGCTGTGCCCGTGGGCACAGAACAGCTCCCGGCTCCGGTGCTTTGGCTACAACCCTGATCCTTACGCCCATTCTCTGGATGAGGAAGTTGAGGTGTGAGTCCAGGAATAGCAAGTGTGGAAGCCACAATTTGAGCCTGGGTCTTGGCTGCCTCTCGCTCTAGTGATCTTTCCACAAAGCCAGGACAGAAGCCTGGCTGGAGAAATAAGGCCCATGCCTTATTCCCAACGTCATGTGGTCTGAGGGTGGGCCCTGTGTGCCCCTGTCCTCCCCACAGTGCCCCGCAGCCAGGAATTCCGTGGTTCCCTAGCCAGAGAGTGCGTGGCCTTCTGGGCCGGGCTAGGCCTGGAGCGAGCTGCTCCATCAGTGGAGAGGAGCCAGGCCCGCGGGCCTGAGGAAGAGGTGAAGTGGTGGAAGGCAGGGGTCTTGGGGTAAGGTGTCGGCAGCAACTGTTAGACCTCGGGCAAGGCCTTTCTGCACCTTCCAGCTGCTAGAGCAATGGGCTCCCGAGCTGCTCCTTGGAGAAGAGGCCAAGCACTCGCTGGATGGCTTCCTGACTCCCAGCACCTCATCCCGCCTGCCTCAAAGCTGAGTGGGGTAGCTTTTGGGGGGGATGCAGGAGACGGCCGTGTCGAGACTAACGTGATTTAAATGCCAAGTGTTTGTAGAGTGGGAGATAAGGGGGGAGGATTTGAAAGGTAGACAGGGACTATGAGAGGAGCCCATGGGCCCAGTAGGCAGGGCCTCACCAGCAGCCACCAGGATGGCTCCAAGCCTACTGTATGCCTTGGGAGGGGCGGGATAGGAACTGTGACCCTGGAGCAGCCTGTGCCCAGGGCCCCTTTCTCTTCTCCGTGTCCTCCCCTTTGCCCATGTGGCCCTTAGGCGGCTCCTCTCCCTCTCCTGTGAGCTGCCCTCAGTGCTGCTGGAGGCTTTCGGGGCCAAGACTCCCCTGTGCTAGCTAGAGTGTGGCCCCTCCCGGGGGCCCCCACCCAAGGCCCCAGGATGCCTCTCTGTTCCCCTCTTCCAGATGCCCTGATGAAGAAAGCCTTCGATTCAGCCGCCTTCAACTCCAATGTGTTCATCACCAGGCTCTTAATACACATGGGGCTTCTCAAGAGCGAGGATAAGATCAAGGCAATCCCCAATCTCTACGGCCCCCTCATGGCCCTCAACCACATGGCCCAGCAGGACTACTTCCCCAAGACCCTTACACCCATCCTCCTGGCCTTTGTGACCAAGCCCAATCGGGCCCTGGAGTCCTGCTCCCTTGCTCGCCACAACCTCCTGCAAACCCTCCACCACGTCTAGTCGCTAAGAGGCCGGGCCTCTGCTGTCGTTCCCCATGAACGTTATGACCTGAGAGCCCCTGCCCAGGCCCAGAGGACGAGTGTGTGTGTCTGGCGTCACTTCCACTGAAGATACCCTTCCCCTCCTCCCCCCGGGACGCCGAGGGGACGCTGCTTCTGTTTGGCCTGCCGGCCCTGGCCGGGCCGGGCTTGTGGGATTTTTAATGACGTCTCTGTTGTGAATCACCTCGGCCTGCCTCAGGGGAGCTGGCCTCGGACACTTGCTTCCACCCTGCCTGCTCGCGGAGTCAGTCAGTGGCCCCTTCATTAAACGCTGTTCCCTGCCCTCACCTCACGCCGGCCTGCCTGGATCTCCCCTGGCCATCGCTGCCTGTTCTGGGTGCAGCGTGGTATCCTTAGCTGGCTCGGGGAGCTCCTTGGCCACTTCCTCTCACTAGGAATCTCGGGAAAGGGGAGCAGCCTTATCTCTGCCTCCCAGGCTCTGCTCCCGGGAGGACCCCGGAGCACAGTGTGGGAGGCAGACCTGGTGACCCCAGGGCCTGGCCCTTCCTAGTCAGTGACTCGGGGGCCTGTGCCCTCCCAGAGGAGCAGAAGTAGGCAGCACCTGGGTGCCTACTGTCTCCTGTGGTAGCACCAGCCTGGCCTCAGACCTTCTGCTCCGTGGAAGGAGCTGACGGGCTGGGCGCCCTCTGCTGTCCTGGGGCCTGAGCCCCGGCCCTTCGGCGGGGTGCTCAGGAAGGAGCCCTGCCCCACCTGGCTTCTTGGGACTCTGGACTGGAGCATTCTCAACGGCTCCTCCCCCTCCCCTATTGGAGACAAGAGACTTGAGCGAGGAAAGGTGTCCACTGTTACTTGTCTCCCTTCTAGCCCAGGCCTCGGTCCAGTCACCTTGTTAGCTGGTCACAGGGCAGCTGGGCCTGTTGGGAGTGCAGTACCCCCAGGGCCCTGAGCAGCCGTTTTCTTCTGGGCCTCAGTGGGTTCCAAGATGGGCCTGGGTCCCCGTGGCTCTTAACCTTTTCTCTCAGCTTGCTGTGCCTGGGCCCTGCCCAGGGCTTCACTAATGGACCAAAATGCTGAGGAGGGAGTTCAGGGATCCCCCAGGTTGCCCACTCTTCCCTTCACCATAACTTAGGTTTATCTTCTGTTTGGCACAGGGGGGTCCTGGGAGCAGGCACTGGGCTCAGAGCATGTCTGCAGGGTCCTGAGCTGGTCTCTGGAGGAGCTGCCGTGCCCTGCCTGTGGTGGTCCCCCAGCTAGCACTGGCTCTGGGGGCCATGAGATGCCCATCCTGCTCTCACCTGTCCCTGCTGTCCTGTGAGCGCTGGCAAAGGTTTGGAGTAGGTCTTTGTCCAGCTTCTCTGGCCTTTACCTGGTTTTAGCCAGTGTCCATAGGAGTGCTTGTGCCAGGGGGTGGGGGGGTGGCCCTCCTGTCATCATGGTGAAGGTCCGGGAGGCCTCGGTCTCCCGCTGTTCTTCCCCACGGTGGCAGAAGGAGTGGGCCCTACCCTGGTTGGCGTAACCCGGAACAGGGCCAGCGAGAGGAATGGGGAGGAGGCAGGGCTGGGCCCCGTTGGCCTCTTCAGCTGCCTGCTTGACCGTAGGTGCTCGTGTCACGTCAGTCCTCTGCCTGCTCAAAGGGGGTGTTAGGGCAGAGAGGCCTTGGGCCTGTCTAGAAACCCCAAGGAGAGGCTCTGCAGCTGCCCAGCTGTGTGGGCCCAGGGGCTGTCTTCAGGGCTCCGGTGGGTCAGAGGAGGAGGGAAGGCTACAGTAACTCCCCCCAGTGGGCCTAGGCTAGTGTGGCTTTGGAGCTGAGGAGGCCAGCTCTGGTAGAGCCTGCCCCCAGGGTGCCTTTCGCCCCGGTACACCCGCCCTCATCCATGTTGGTACTTGTGCTGATGTGTGAGTAACTGCCCTGCCTGGGCCGGGGCTTAGGGTAGTGTGCAGAATAAAGTGCTG

>UoN.Pci.T.24059_UoN.Pci.T.24059.1

GTATGAGGCACTGTGTTAGGATATGGAGATATAAAGACAAGAACAATCCCTGCCCTCAAAGAGCTTCCATGCTATTAGGGAACAATGGCAGGACAGGCAAGCAAATGCAAAATACATACAAGTAATTAAAAGTAATTTCAGGAAAGAGAAAGAACAACTGAAATGGGAAAATGGCCTCATGTAGGCCAAAGAGTTGTAGACATGACTAAAAGTGACCTTAGAGGCTACCAACCCTAACTCCTTCATTATACAGATGAAGAAACTGAGGACCAGAGAGTAAGCTGACAAGTTCACAGAAATAATAAGGGCCACCAAATAGGATTATGTAGTGAAGAATTTGTAATAGAATTTGTCCCATATACATAACTTATAAACTTTCATCTTGTGATAATGGCAAAAGAACCATGATCAACTTTCACAAAAATATCAGAGAACATACCTAGTAGTATATACTACCTTAAGCACTCTGATCCAAACTGATATAGATTTGTATGTTATATATTTGGATACCATGACAAAACCAGGCATATAGGTTCTTTTACCAAGTGAAATTTTAATATTAATCTTTAACATGGCAAGGAAGCAAACTACAGAACAGCAAACATTCACTCTAAATAACTAGCTCTGATTCCTCTACGTCTTAACTTTAGTGTTTAGAAGCATGTTCCTCCTCATTCTTTGGTGCAGTTAGGAAATATTCCACTCCGACAGCTAATAAAAATGCTCCAAAACCCCATTTGAATCCTCTTGTCAAAGCAGCCCACAAAGTAACAGGTTTTCCATAATGACCCATGTATCGCCAAGCTTCATTGCGGGCCCATGGATCCCTGAGACCTTGCTTAGCCAACCTCTCCTGGACTCGCTGTAGTGGTGTTCCTTCTATCTTCCACTGTTTGTAATCAGGGAGTTCAATTTTTCCATGACCATGTCCATGCTCATGTCCATGTGCCATGCCTGACAGGAATCCTGACCTCCCCGGGACACTGGAATCTCTATACCGGCCCCCCCCCACCCCGTCCCCCAAG

>UoN.Pci.T.24992_UoN.Pci.T.24992.1

CCTCCCTCGGCGCACAGGACTAGGGGAGGTGTGGGCTTGTCCCTGCTGTCTAACCGTCGCGCCTCAGACTCTCAGACGCAGCTTCCCAGCAGGCACTTCGAAGAGGAAGGGGAACGGTTTCCGGCGGCGAGTTCGCGGCGCGAGAAGTGACGTGTACGGTAAACGGAGCTGTAGCCGTGGCAGCGGCGGAGCCAGCTACGGACCGGAGAGCGGAGCTGCCGTCTCCCGCGCCCGCCCTGCATCGCGGCGGGCCCCGGCGTCAGCCATGGCTAAAGACATCCTGAGTGATGCAGGCCTGCACTTTGATGAGCTGAACAAGCTTCGAGTCTTGGACCCTGAGGTGACTCAGCAGACCATAGAACTCAAGGAGGAATGCAAGGACTTTGTGGACAGTGAGTAGCTCTTGGCCCTTTCTCCTTCAGCTGCCTCCCCCAGTCCCAGAAACCCAGGCTTATGCCCTCCTCCTTATAAGGCAGTCAGGGGGCTACTGCCCACGCTCATGCTACCTCCCCCTACCCATCCTTCTCCTCTCTTTCTTAAAGACTGAGCCCCCTTTGATTGCAGGGCTACTTATGGGTCTGATCCTGTTATTGAGAGGCACAGGAGTCTGGACCTGTTCTGTTTCTGCCCTGGGCTGGTTTACTCTCTTTCCCCTCCTCGTTCATATTAATGCCAAGCTTAGTGCAGGCACCTTATTGGCATAGTCCACTTTACCTGAGGCTCCTAGGTAGTCAGCATCACCATGACATGCCCGTCCTTTCTTATTTCTTAATGCATGGGAGGGGGCTGGGTTTTCATTTTATCTTACACTGGCCTCTTCCAGGCTGATGGAGAGATCAGAAATTTAACCTTGACTGAAGATAGATCTTTCTTTCCCACAAGCTTAGTTTGAATGGTGTTTCCTCCAAACATAAACCATGGCCTTATGCTGTTTTTCAGTTAACAGCCAGGCTTGACTGAAAAACTGGATTTCCTTCAGATTTATCTTAATATTTGATTTCTTAATCTGAAGACGTGTGTACTAGGCTTATCTCTGCCCTCGTGTGCCTTCTCAGCATGACCTTGGCTAAGTCACGTAAACTCACTTGGACCTCAGGTTCCTTCCCCTCCCCCACCAAACGGTAATCTTTGCCTTTCCCTGCTCCTCCTGCCAAAATCTAAAAGCCAAATGGAGGAATGCACAAAGAAAAGCCTTGGGTTCTTTGGTGTGAGGGTTAACGTAATTCTCTGTGGTCATGTGGGATGCTGACGTTCAGCTGATCCTCGGGCCACAGTTTGCAGTCTCCCACAAGAAACCTCTGATGGACTGGAACAGATGGTTGCTACCCCAGCAGGTAGGGCTCAGCAGAAAGAACGTACCCGCAAGCTGAAGGGATCGCTGAAAACTTCTGTCCCAGGGCATTCAACATTGAGGCGGCTGCCCAGGCAAACCGCGCTCTGCAGGGCTCTAATGGGGAGGAAGTAAAACGTCTCACTTTTCTTTCCCTGAAAGAAATTGGCCAATTTCAGAAAATAGTTGGCGGCTTCATTGAGCTCGTTGACCAGCTCGCGAAAGAAGCTGAGAACGAAAAGATGAAGGTAAGATGAGGTCAGACAGAGGCATCTTAATGAAACCTTGGGCCCCTGACCTTCCTGTCCTCTCCTTCCTGTCCACCTCATGCCATCTGTTTGAGAGAGCCCTCATGAGGCAGGCACAGTGCCTGCTTTCAAAGCTAACCTTCCTTTCCTTGCTGATAGGTCCGACCAAAGGTAGGCCACCCTCACCCTCCCGTCCTCCGATTCTCTGAATGGCTTTTCCATCACGGCGAGTTTTTCGTCGAGTAGCTGTGTCACGACCCCCACTTTCTGTGTTCATCCTGTTTTCACGGTTTACTTTGGGGAGGCTGAATACAGCTGGAAATTGTTGTTCAGTTTTCTCAGTCATCTGTCTCCACAGGCAATTGGGGCACGCAACTTGCTCAAATCCATAGCAAAGCAAAGGGAGGCCCAGGAGCAGCAGCTGCAGGCTCTAATAGCAGAGAAGAAGATGCAGCTAGAAAGGTAAGAATTATGAGAGGAACTGACCTCGGCTCATTCGCCAGCTGTCCCCAGTTTGGCTGTTAGGCTCTGCAGCACCCACTCTAGTGCCCTTCCCTCTTCCCTCCACTGGAGTCTCAGAACTCTTGGCTAGTTAAAGATGGGTTTTGATGGGAAACATTATTTTTGTTACTTTTCTTGCTTATATTGAGACTTTTTTTTTTGTAGTTTCTGAATCTCTTTCTCTTATCCCTCTCCTTTCCAGTGAGCTATCACTTGCAGCAAATAAGAGAAAAAAAATGCATCAGTAAACCCAAAGTTATCACCTGACTCTGGTGTATGCAGGTGTCCCACATCCATAATTCCCCTTTCTCAAAAAGAAGGGAGTGTACTCTCAGAGTCTCCTTTTGACCATTATTAGTGTTAGGTTTGGGAAAACTTATTTTTAACTTTTGTCAAAGAGTGAGGAGGGGGAAAAGCTTAGAAGGAAAGATTAGTTTGGTGTGATTGTGGCTGTCTGTACTGTAGATTGAAAGTTGTCCTAGCATGTCCTGGAAAGAAATGTGCTTAAGGTAGATAGATTCTGGGATCGTGATGTATTTCTTTTCCATCCTTTACTTAATTTCTTCTGTTTTCTTTTTTAAAACTGCTGAACCTTCTTGCTTCCTCAGAATCATGTGACCACCCTGCTTCCATAACATGTGCACACAGTAGACAAGTGGTTTGTATACCATCATTTCATCCCTCTTTAGGATTTCTAACAATTTTCTGTCTTTCAGGTATCGGGTTGAATATGAAGCTTTGTGTAAAGTAGAAGCAGAACAAAATGAATTTATTGACCAATTTATTTTTCAGAAATAAGCTGATTTTTACAGTAAAAACCCTATTTTAAAAAAACCCCCCACCAAAAATCCCCCCTCAACCGTATCATTCCAATAAACTTGATGACCACATGCACATATATGCAATTTGGGATTGTAACAAATAGCATGTCTTCTTGAAAGGCAATAACAAACCTTTTTTTAGAATTCTGCCCAATTGTAGCCAAGTGGCAGTAACCAGGACCTGCCTATCCAGAAGGCAGGACAGCAGGGCTAACCATCTTTTGCTGTGGACTCTCCTGTGTTGTCAGTCTCTGACCTCTAACCACATCTGTAAACACCAGTCCTTTTTTGAAGCGCCTGCCACTTTGAAATAAGGCAATAAATTATTATTTCTAGATTTTTGTTATCTGCTTGTTGTACTTTGAATACCTATTTGCAGTTCTCACTGGAAAATTCTTTCTCCCCGAGTAATCCACAAAAGAAGTCATGGTCAGCAGCCATCTTTATTTGAATTAGGTACACAATCAGTTTTCAACAATTAAAGCAAATACCAATTTGTGAGGAGCATTTTATGAGGAAGTCTTTATTAAGTGCTTACTCAGGCCCTGGAATAAGCACTAGGGATACAAATACAAAAACAAAAAACAGGCTGCCCTCAAGGAACTTACATTCTAACATAGGAAGACACACACATATCAGGTAGTAGTGGCCAGGGAAGAGTATATTGGTCTGGAAAGTCACAGGGATGGTGAATGGAATCAGAGTAACTGACTGACATCATCTTTCCAGGAATGGTGGTTGATTTGCTTACTGATCCCAAAGAAAAAGGACCACAGGAGAGGTAGGAGGTTAGGGGGAATCCATCCTGTGGCAGCATGACAAGAAGACCGCCAGGGTGCCTTCCAGGAAGGGAGCTGGACAAATGTCCAAGAGTCATGAAGAGTGGAAAGCCCCACAGCAGAGGCAGTGTTGCCTGGAGCCTGCTCCACTCTGTTATGGACAAGGGCAAACTTAGTTTTGGGGAGCAAATTTCAGGGTAACAGTAGGTCACTACCTCCAGGTACATGCAGGAATGACAGAACATGATGAGCAGCTGCTATTCATCTCCATTACCATGTGGAGACTTAAATTGTGATAGAAAAAATGACAGTTTCTTGGTGAGAGGCAAGACACTGGACCACAGCAGGTTATAATGAACAGGTTTTGGAGAATGAACAAGATCATACTGCTTGGAAAGCAGGGTTTAAGTCAGATGACCCCTAAAGAGTCGAAAGCCCTAGGATTCAAATCAGTAAGGCTCAATAGAAGCACTAGTCAGAATAATTCTAATTGAAGAATTTGATGGGAATGGATCTCGATTAAAGAAAAAATTTAAAGCCCTTTCCATTGAATGTTAATTCCCTTCAAAAAAAAACAAACAAAAAACAAAAAAAGGAAACCTAGAGAATCCCATTCCCACTGGAGAAATGCCTGGGGTGGGGTGGTGGGGGTAGTTCACAACCCTTTAAAACTAGCTCTAAAAGTGCAAGTGTGATGGACGAGGACATTGGGGCACTCAAGTTTATTGGGTCCGTGGGCCTACCAGGCTAGCAAGAGAGCAGGTGTTGTGCACCCGTTTGGTCTGATTTGGGTTGGAAAAAACTTAATGAAGAATTGCAAAGTAGCCCAAGAAATGTGTTTCTTTGAGCAGTCCCCTTATATGGCTTGGAGAGAAGAGTACCAAATGGCTGGTCATTCCGATTATTGGTCAAACTAACCTGAGAACCTGACTGATTAGACAGTTTCAAAGAATCTTCTTGTGGGGATTATGGCAGTGTGAATCCTGAGATGGTACTTGAGGCACACGCTGAGTATTCAGCACTGCTTCCCTGCAGCTCCCCCTGCCATCTTCAGGGGATCAGTCATTCCCAATTCCCCTGGCAAATGGCTTCTCCCACCTGCAGCCTTGTTCTGGGAGGTTGCTGCTGATGTGTTTCAAGGGGGGCCTGGGGAAACTGAGCACACACAAGGAGCTCTCTCTCCCTGGCCAGCTCTTCTTTACCTTGTTTTCCTTTTCTTTTTCTCGTCGCTGCTGTAGTAAGGACTCCAAAGCATAAAAAACAGGAGTATGAGTGGAATGAAGAAGTATGGAGCATAGATGGATAGAAGGCTGAGGCGTTCTTGGAGTGTCCTAGGTCCTTGGGTGAAACTCTCAAACAGGATGTGGGCGCTAATTGGAATTAATGTTGTCATTGTATGAACTGAGTAGATAATCGCAGGGATGCGGATCCACTTGCAACTTCCTTTGAAGAAGGCATACGCTGCAAAGGGAAAGAGAGGCAGCTGGAACACAAGCTCACAAAAAAGAAAGGACTTGAACCAGCTTGGAGGGTTCAGCATCAGAGGGTCTTTAAATGCCTGCACATACCACTTGAGCATGTCTGTCACCTCGGTCGGGAACAAGTTAGCCGACAGTAACGCCTGCATGTCGAAGAGCAGCGTGATGGGGATGTGCATCACGAAGTAGAGGCCCAGCAGGTACTCCAGCGCTCGCGCCCCGCCCCGCGAGCCCATCGTGCCTCAGACTCCGGCCGGGCCGCCAGAAGAGCTGCGAAAGAGGTTGGCTCGCCGGGACCGAACGGCGCACTAGTGAGCGAGCGCTGGGCGGACGCCGCCGGGACGCCTCGGCCCCGCCCCCG

>UoN.Pci.T.24992_UoN.Pci.T.24992.2

GCGCACAGGACTAGGGGAGGTGTGGGCTTGTCCCTGCTGTCTAACCGTCGCGCCTCAGACTCTCAGACGCAGCTTCCCAGCAGGCACTTCGAAGAGGAAGGGGAACGGTTTCCGGCGGCGAGTTCGCGGCGCGAGAAGTGACGTGTACGGTAAACGGAGCTGTAGCCGTGGCAGCGGCGGAGCCAGCTACGGACCGGAGAGCGGAGCTGCCGTCTCCCGCGCCCGCCCTGCATCGCGGCGGGCCCCGGCGTCAGCCATGGCTAAAGACATCCTGAGTGATGCAGGCCTGCACTTTGATGAGCTGAACAAGCTTCGAGTCTTGGACCCTGAGGTGACTCAGCAGACCATAGAACTCAAGGAGGAATGCAAGGACTTTGTGGACAGTGAGTAGCTCTTGGCCCTTTCTCCTTCAGCTGCCTCCCCCAGTCCCAGAAACCCAGGCTTATGCCCTCCTCCTTATAAGGCAGTCAGGGGGCTACTGCCCACGCTCATGCTACCTCCCCCTACCCATCCTTCTCCTCTCTTTCTTAAAGACTGAGCCCCCTTTGATTGCAGGGCTACTTATGGGTCTGATCCTGTTATTGAGAGGCACAGGAGTCTGGACCTGTTCTGTTTCTGCCCTGGGCTGGTTTACTCTCTTTCCCCTCCTCGTTCATATTAATGCCAAGCTTAGTGCAGGCACCTTATTGGCATAGTCCACTTTACCTGAGGCTCCTAGGTAGTCAGCATCACCATGACATGCCCGTCCTTTCTTATTTCTTAATGCATGGGAGGGGGCTGGGTTTTCATTTTATCTTACACTGGCCTCTTCCAGGCTGATGGAGAGATCAGAAATTTAACCTTGACTGAAGATAGATCTTTCTTTCCCACAAGCTTAGTTTGAATGGTGTTTCCTCCAAACATAAACCATGGCCTTATGCTGTTTTTCAGTTAACAGCCAGGCTTGACTGAAAAACTGGATTTCCTTCAGATTTATCTTAATATTTGATTTCTTAATCTGAAGACGTGTGTACTAGGCTTATCTCTGCCCTCGTGTGCCTTCTCAGCATGACCTTGGCTAAGTCACGTAAACTCACTTGGACCTCAGGTTCCTTCCCCTCCCCCACCAAACGGTAATCTTTGCCTTTCCCTGCTCCTCCTGCCAAAATCTAAAAGCCAAATGGAGGAATGCACAAAGAAAAGCCTTGGGTTCTTTGGTGTGAGGGTTAACGTAATTCTCTGTGGTCATGTGGGATGCTGACGTTCAGCTGATCCTCGGGCCACAGTTTGCAGTCTCCCACAAGAAACCTCTGATGGACTGGAACAGATGGTTGCTACCCCAGCAGGTAGGGCTCAGCAGAAAGAACGTACCCGCAAGCTGAAGGGATCGCTGAAAACTTCTGTCCCAGGGCATTCAACATTGAGGCGGCTGCCCAGGCAAACCGCGCTCTGCAGGGCTCTAATGGGGAGGAAGTAAAACGTCTCACTTTTCTTTCCCTGAAAGAAATTGGCCAATTTCAGAAAATAGTTGGCGGCTTCATTGAGCTCGTTGACCAGCTCGCGAAAGAAGCTGAGAACGAAAAGATGAAGGTAAGATGAGGTCAGACAGAGGCATCTTAATGAAACCTTGGGCCCCTGACCTTCCTGTCCTCTCCTTCCTGTCCACCTCATGCCATCTGTTTGAGAGAGCCCTCATGAGGCAGGCACAGTGCCTGCTTTCAAAGCTAACCTTCCTTTCCTTGCTGATAGGTCCGACCAAAGGTAGGCCACCCTCACCCTCCCGTCCTCCGATTCTCTGAATGGCTTTTCCATCACGGCGAGTTTTTCGTCGAGTAGCTGTGTCACGACCCCCACTTTCTGTGTTCATCCTGTTTTCACGGTTTACTTTGGGGAGGCTGAATACAGCTGGAAATTGTTGTTCAGTTTTCTCAGTCATCTGTCTCCACAGGCAATTGGGGCACGCAACTTGCTCAAATCCATAGCAAAGCAAAGGGAGGCCCAGGAGCAGCAGCTGCAGGCTCTAATAGCAGAGAAGAAGATGCAGCTAGAAAGGTAAGAATTATGAGAGGAACTGACCTCGGCTCATTCGCCAGCTGTCCCCAGTTTGGCTGTTAGGCTCTGCAGCACCCACTCTAGTGCCCTTCCCTCTTCCCTCCACTGGAGTCTCAGAACTCTTGGCTAGTTAAAGATGGGTTTTGATGGGAAACATTATTTTTGTTACTTTTCTTGCTTATATTGAGACTTTTTTTTTTGTAGTTTCTGAATCTCTTTCTCTTATCCCTCTCCTTTCCAGTGAGCTATCACTTGCAGCAAATAAGAGAAAAAAAATGCATCAGTAAACCCAAAGTTATCACCTGACTCTGGTGTATGCAGGTGTCCCACATCCATAATTCCCCTTTCTCAAAAAGAAGGGAGTGTACTCTCAGAGTCTCCTTTTGACCATTATTAGTGTTAGGTTTGGGAAAACTTATTTTTAACTTTTGTCAAAGAGTGAGGAGGGGGAAAAGCTTAGAAGGAAAGATTAGTTTGGTGTGATTGTGGCTGTCTGTACTGTAGATTGAAAGTTGTCCTAGCATGTCCTGGAAAGAAATGTGCTTAAGGTAGATAGATTCTGGGATCGTGATGTATTTCTTTTCCATCCTTTACTTAATTTCTTCTGTTTTCTTTTTTAAAACTGCTGAACCTTCTTGCTTCCTCAGAATCATGTGACCACCCTGCTTCCATAACATGTGCACACAGTAGACAAGTGGTTTGTATACCATCATTTCATCCCTCTTTAGGATTTCTAACAATTTTCTGTCTTTCAGGTATCGGGTTGAATATGAAGCTTTGTGTAAAGTAGAAGCAGAACAAAATGAATTTATTGACCAATTTATTTTTCAGAAATAAGCTGATTTTTACAGTAAAAACCCTATTTTAAAAAAACCCCCCACCAAAAATCCCCCCTCAACCGTATCATTCCAATAAACTTGATGACCACATGCACATATATGCAATTTGGGATTGTAACAAATAGCATGTCTTCTTGAAAGGCAATAACAAACCTTTTTTTAGAATTCTGCCCAATTGTAGCCAAGTGGCAGTAACCAGGACCTGCCTATCCAGAAGGCAGGACAGCAGGGCTAACCATCTTTTGCTGTGGACTCTCCTGTGTTGTCAGTCTCTGACCTCTAACCACATCTGTAAACACCAGTCCTTTTTTGAAGCGCCTGCCACTTTGAAATAAGGCAATAAATTATTATTTCTAGATTTTTGTTATCTGCTTGTTGTACTTTGAATACCTATTTGCAGTTCTCACTGGAAAATTCTTTCTCCCCGAGTAATCCACAAAAGAAGTCATGGTCAGCAGCCATCTTTATTTGAATTAGGTACACAATCAGTTTTCAACAATTAAAGCAAATACCAATTTGTGAGGAGCATTTTATGAGGAAGTCTTTATTAAGTGCTTACTCAGGCCCTGGAATAAGCACTAGGGATACAAATACAAAAACAAAAAACAGGCTGCCCTCAAGGAACTTACATTCTAACATAGGAAGACACACACATATCAGGTAGTAGTGGCCAGGGAAGAGTATATTGGTCTGGAAAGTCACAGGGATGGTGAATGGAATCAGAGTAACTGACTGACATCATCTTTCCAGGAATGGTGGTTGATTTGCTTACTGATCCCAAAGAAAAAGGACCACAGGAGAGGTAGGAGGTTAGGGGGAATCCATCCTGTGGCAGCATGACAAGAAGACCGCCAGGGTGCCTTCCAGGAAGGGAGCTGGACAAATGTCCAAGAGTCATGAAGAGTGGAAAGCCCCACAGCAGAGGCAGTGTTGCCTGGAGCCTGCTCCACTCTGTTATGGACAAGGGCAAACTTAGTTTTGGGGAGCAAATTTCAGGGTAACAGTAGGTCACTACCTCCAGGTACATGCAGGAATGACAGAACATGATGAGCAGCTGCTATTCATCTCCATTACCATGTGGAGACTTAAATTGTGATAGAAAAAATGACAGTTTCTTGGTGAGAGGCAAGACACTGGACCACAGCAGGTTATAATGAACAGGTTTTGGAGAATGAACAAGATCATACTGCTTGGAAAGCAGGGTTTAAGTCAGATGACCCCTAAAGAGTCGAAAGCCCTAGGATTCAAATCAGTAAGGCTCAATAGAAGCACTAGTCAGAATAATTCTAATTGAAGAATTTGATGGGAATGGATCTCGATTAAAGAAAAAATTTAAAGCCCTTTCCATTGAATGTTAATTCCCTTCAAAAAAAAACAAACAAAAAACAAAAAAAGGAAACCTAGAGAATCCCATTCCCACTGGAGAAATGCCTGGGGTGGGGTGGTGGGGGTAGTTCACAACCCTTTAAAACTAGCTCTAAAAGTGCAAGTGTGATGGACGAGGACATTGGGGCACTCAAGTTTATTGGGTCCGTGGGCCTACCAGGCTAGCAAGAGAGCAGGTGTTGTGCACCCGTTTGGTCTGATTTGGGTTGGAAAAAACTTAATGAAGAATTGCAAAGTAGCCCAAGAAATGTGTTTCTTTGAGCAGTCCCCTTATATGGCTTGGAGAGAAGAGTACCAAATGGCTGGTCATTCCGATTATTGGTCAAACTAACCTGAGAACCTGACTGATTAGACAGTTTCAAAGAATCTTCTTGTGGGGATTATGGCAGTGTGAATCCTGAGATGGTACTTGAGGCACACGCTGAGTATTCAGCACTGCTTCCCTGCAGCTCCCCCTGCCATCTTCAGGGGATCAGTCATTCCCAATTCCCCTGGCAAATGGCTTCTCCCACCTGCAGCCTTGTTCTGGGAGGTTGCTGCTGATGTGTTTCAAGGGGGGCCTGGGGAAACTGAGCACACACAAGGAGCTCTCTCTCCCTGGCCAGCTCTTCTTTACCTTGTTTTCCTTTTCTTTTTCTCGTCGCTGCTGTAGTAAGGACTCCAAAGCATAAAAAACAGGAGTATGAGTGGAATGAAGAAGTATGGAGCATAGATGGATAGAAGGCTGAGGCGTTCTTGGAGTGTCCTAGGTCCTTGGGTGAAACTCTCAAACAGGATGTGGGCGCTAATTGGAATTAATGTTGTCATTGTATGAACTGAGTAGATAATCGCAGGGATGCGGATCCACTTGCAACTTCCTTTGAAGAAGGCATACGCTGCAAAGGGAAAGAGAGGCAGCTGGAACACAAGCTCACAAAAAAGAAAGGACTTGAACCAGCTTGGAGGGTTCAGCATCAGAGGGTCTTTAAATGCCTGCACATACCACTTGAGCATGTCTGTCACCTCGGTCGGGAACAAGTTAGCCGACAGTAACGCCTGCATGTCGAAGAGCAGCGTGATGGGGATGTGCATCACGAAGTAGAGGCCCAGCAGGTACTCCAGCGCTCGCGCCCCGCCCCGCGAGCCCATCGTGCCTCAGACTCCGGCCGGGCCGCCAGAAGAGCTGCGAAAGAGGTTGGCTCGCCGGGACCGAACGGCGCACTAGTGAGCGAGCGCTGGGCGGACGCCGCCGGGACGCC

>UoN.Pci.T.25124_UoN.Pci.T.25124.1

CCCTACCCCAGGCCAAGGGCAAAAAGTGGGGAGGCATTTCATAATTTATAGATGCCGGCCCCACCCTTCTGGGATCGGAGCATGGCTTACTTGATGAAAGGTAAAAGGGAGGCAGCACCTGATATCTTGGGGGATGGTGGTGGAACATTTCCTCATTGTGATGCTAAGATCTGGACTGCTGCCCCTCTGTGGTAAGAAGAAGAAGTGCATCTATAGAAGGCTGTCCCCAGGAGGCAACCTGGGGGGATCCTGGCAAGCTGGAAAATGAGCATGATTTCCAGCCCTTTCCTCATGATTGTCATTTGCTCTCCTAAGCAACGGGAAAGAGGGCTCCCCGTGAACGAGCTGACATGTCCCTGCTCGTACAGGTGACTCTGACCTAGGGTGGCCCCATTTCCAGGAGTCCATGGAAACTGGAGAAGAGGAAGGGAGGGGTTCCAGCAGCCCAGGGGCTACAGTGCAAGGTCAGGGGTCATCTGCAGGGAGGTGCCAGGGGACTTTCGGCGGGCACTCCGACGACCTGTATCTGACCTTCGGTATGGCTGACTCCGAAGATCAGTGATAATGTCCTCTATGGCCCCATCACGGTCACTCTCATAAATGAGCGGCTCTTTCTGCTGCTTCCTCTTCAGCTCCGAAATGAGGTCCATTTGTTGCCTTCGGGCCTTGGTAGGGGACTGGATCAGAGGTGATGGGGAGGCATGCTTTGACCATCTGGGCCTCCTCCTCTCCTTCCAATCCCCAGCCACCCCAGACACCCCATTTCTCAGGGACACAGCTGGGAAGGTGGTGACCACTGGAATGCCCAGGGGGATCCAGTGAGGGGCTGGGATGGGTGGGAGGGAGGGAGGCTCAGGGAAGAGACACTAGACAGCCTCATCCCCACAGCAAATACTTGAAGACCTTCCTGGGCAATGGAAAACCACTTTGCAGGTGTAGACTTTGGTCTCCTTAGGCTACATAAGGGCACATTGCCCGCCCCCCCCAATAGACAAGCAGTGAAATGATGTCCCCACGGAGACCAGCTGTGGTTATGGGGGGCAGGCAGGGAGGAAAGAAAAGAAATCCAGCATCTCAACACCCAAGCAGCATGGAATCATGCCTTACCTTGGGCACTGCAGGCTCCCCTTCGCCTGCGATGGGAGCCCCGGGAGCCCCGGCCTCCTGGGCTGCAGCCTCCTGTTTCTTCCACTGTTCCACCTCCTGCTCAGCTTTCTTATAGGCTTTGACGAAGCGACTGAATACAGGGAAGAACATAGACGGAGGGGTGGTCTTGGGATTCTCCCCGAAGTATTCCACCACAGATTCATATGCCTCCTGGGCTGTCTTTCCATCGGCCAACAGCTTCTCCATACTGGGACTGTTGACCTTCAGGAACTCCTTCAACACGGCATTATCATCTTGGCGCATGAACTCCCGCCTGGTCAGCTCCATGCCCCGCTGGAGAGCCCGGGTGTCCTGGAGGACACTGTCTAGGGACACAGCCCCAGCCTTGTCCAGAAAGTGCAGGTCAGCGTGGAAACAGGTGAGTTTGGGATACTTCTCTCCGATCACCTTCACCAGGTAGTGCAGCAGTGTCTGCTTCCGGTCCGTCGACTTCATCTCCAGCAGTGCATCAAGGCTTTGCAGCCTGAAACCATAGGCTGCCCCGCGTTTACTGCTGTTCATGTAATTCCCAAAGGCCAGGACAATCTCTAAGATATTTCGGAGCTTGTCAGAAGACTTGAGAGATATGGAAGCAGCGATAATAGCGTTCAGTTGCGGCATGAGTAGCTGAGCAGTGTCCGGGAAGTTGCCCAGGAAAGCCAGAGTGGCCATTCTCTCAGACAGGCGAGGGATGCGGCTGAAACGCAGCATGAACCGGTCCTCGTCAGACAGCTCCTCCACTGGCCGCTGCTCCTTCTCGTAGCGTCCAATGAGGCTCAGCTCATACTCAGTGGGCAGGAATCGAGTCAGGAGCTCCAAAAAGTCCAGGCTGAGGGCCTGCAAGTCATACGTCTCGATGGCCTGGCAGATACGATCGGTGCTCAGATTGCCTTTGCGTAAGGTGATGGCCAGATTCTTGGCCCGGTTGGCCTCAATGAGCGTCACCTTGCTTGGGGCCTTTTGTGTAGTCTTGGCTTTGAGGGCATTCAAGTCCAAGGAAGGACCCTGAGACTTGGTCTTGAACTGTTCCTCAAAGTCACTCATATCCAGCTCCTGTAGGACTTTCTCATCATTGATCTCAGTAAAGACAGTGCCCGTGATCTGGTTGGGCTTTAGGGCCACCCAGTTGAAAACTGGCATCCGGAACTTGGTCTGAATTGGTTTCTTGGCCTTCACTGTACTAGTTTCTGAAGCAGGCCCCCCAAAGGCATCAGGAAGTCCTCCAGGAGGAGGAGGAGGAGGAGGTGGCACTGTTCCATTCATCCCTGGTGGTGGGGGTGGTGGAGGTGGGGGTGCTGGGGATGCCCCAGGTCCAGGCAATGGTGGGGCAGGGGGTAGTTCCAGGTTACCAGGAAGGGGTGGAGGTGGAGGGGGTACTGAAGGTGGGGTTTCTTGATGGGTAGGTGAGGCGAAATCAGAGATTGGGGGAGCTGGTGGTGGTGGCGGTGGTGACGGTGGACACACAGATGCAGTGGGGGGAATTGCTGGAGGTGGGAGATCTGTGCTGGAGGAGTCGGGGGCCACAAGCGTCATCGTTTTGGGCGTCATCACGTCAGATGGAGTTGCCACGACAACTGGGATGATCTCAATGGTGATGTCTTCCCCAGGTCCTCTTAGGATTCGGATTAACCCTTTCCTTTCCAGCTCCTCCAACTTAAGCTCCAAGGCAGAGGGCTGGGCTGGGACTGGGGTGAGTACAGGCACCTTCTTGGGGTCAGGGGGCAGCTGGGAGGTACCCATGGTGGCACACTCACTGTGGCGCTCCCGGAGTACGTCCAAGTCCTTTCGGGTATAGCTCAGCTGCTTCTCCAGCTCAGCGATCTTGGCCATGGACTCGTTCTCTGCCTCCCGGAGCCGCTCTGTCAGCTGTGGCAGCAGCACATGGTGAGCACACCCACCCCTGGCTGCTGACATCGGGCGAGGGGGTGGGTACCAGGCAGGCCTGATACCCAGGAGGGGGCGGGAAGAGGGACAAGAATGCCTCTTGCTTCAGCGCCCTTCCCTTTTATTCTCCAATGTGTGGAGCGGGGACAGGTACAGGGAATGGGGACAAGGAAACCAGCTGGCAGTGAGGTGAGCTCCCCATCACTGACCCTGGCACAGACCCCACTGGGGGCTCCACATTCCCCAGGCCCCTTCCTTTTTCTGGACCAAGAGCTAGAACTACTCAGTTCAGAGATCCTAGAATGTATTCTCTTGCCACCCATTCAGCCTTTAGCTGTGAAGAATAACCTTCATCCCCCTAGCTCTACTATTTTATTCTTTGGGGCATATTCCTTTTCTGCCTGGCACCAGTAGGGGGTACCATGCCTAGTCTTCAAGCAAACAAACACCAAGGCCCTGTGTAGTCTCAGCCTATGGGAGCACCAGTCTGCCCGAGAGGGTGGTAGGAAGGGCAGGATCAGAGTGGGAGACAGAAGCTCTGGGAAACAGGAAGACCCCAATGGAGAAAATATAAATTGACTCATCCATTCCCCCAATCTTCCTCCTTCTAGAGGAGGTCAAGAAGATATCTCAAAGCACTCAATGTTTTCTCCCACTGGTCAGGGGAGAATCCTTGATGAAGGAAGTGGGGGTGAAGGCGTAAGGTACCAGAGCAGGAGTTCTGACTCTTGGGCTCCCAGGGTCCCCACTAGATGTCCTGGGGTCCTTCCTGGGGCTAGTTCCTTCTGTCCCCAGAATCATACCTGCTTTGGAGGAGGCTGCATAAAAGGGGGTCTCCTCCTGGTCCCACCCTCTTACCAAGGCCACCTGCTCCTGCAGCTCCTCCATGTGCTCCAGAACTGCATTCTTCGTCTCTGTGTCCTCCAGGAGGGCACCCACATCAAACACGTTATCCAGATATGCCTGGATCTGAACCTGGAGCTTATCACTCTCTGTAAGCCGCAGCCTCTGCCACCGGTGAAAGGGTAAGAGGAAAGGGGGTGAGGGCCAGGAGAGGCCCCCTCCCCTAGTGTTCTCCCACAGCGAGTAAAGGAACAAGCTCTACTAATCCCTCTCCCATTAAAAAGAAAATACCCCAAAAGTCCCGCTCCTCCAGGAAGTCTTCCTGAATTGATCAGAAAAATTAGACCTAATAGTTTATTTTGTTATGCTCTACTCCATTTGGCCATGATGTCCTCCACAGATGGTCTCCTCCAGGCACCCTGGCTCTTTCCTCAAAAGGCACACACAATTTTCATGCTCTAGGCTTTCATGCATTTATTTACAATTAGCAAGCATCGCATGTCTGCACGCCCTGCCTTCATATGGGCAGTCTGTTTGAGACCATCTCATGCTCAGGCTATAGGGTACAGGACAAAGTATAGGGTATGGGGCTTGGGGGCCTGGAATTCTTAAATTGCAGTCCAGTTTCTGATGCTAACTCACTGTGTAATCTCAGGCCAGTCACTTCTTCCTTGGACCCTCTATAAAATGGCAGGCTTGGACTGGACGGTCTCTTCCCACTCTGAATTCTAGGATCCCATGAGCCAAAGGCAGGGGCCAGGGCTTGGCCTGTGTGGCCATCTGTGCCTCCAGCCCAAAGCCATGCCTTCACACAACAGGATGTTTGGACAGGGTCTCTTGCTGCGGATGCTGCTAAGAGTGTCACTGGGAGAAGGAGGGACTCCCACACCAGGGCTTACCTCCAGGTAAACATCCAGGCCCAGGTGGGTGAACTCATACTGCAAGAAGACTCGAAAATTCATGTTCTCCACTGAATGTACCACAATGTTGATAAACTGCATACAAGCCACCATGAAGTCAATGTTGCTATCTTCATTCCGGAAATACTCCATCAGTCTCTCAAACCTGTTCTTCTCACCACACGCCTCTTTGAAGTTGTCAAAGGCAGCCAAAATGATTTCGTGTCCCCCCCGCACCAGACACACCGCAGCCAGTAGCTCCAGCACAAGAGCCTTGGTTCTGGGACTCTTGTTGTTGAGACTCAGGGTAATCTCATTGACACAGGCCGGATGATTCATGACAAGGCTGAAGCCAGACTGGTAATTCATGATTGCCCGGAGACACATGATGCACACGTGTACATCGTCCTTCTGGCTGACGATACGGGAGTTCCTCAGTGTCTTCCGGCTGTGAGCTGGGTTTAGCCTGACAGTCAGGTGGCGGCCCCTGCCCTGGGATAGGGAGGAGGAGGAGGAGGAAGAAGGTGGAGAGCCCTTGCCGAGATCTTCAACTGACTGGTCCAGAGGCTTGCTCTTCTCTGGGCTGGGGGTGCCATTATCTGTGCTCTCCATGTCATACGTGACTGAACACTGGGCAAAGGCCAGGTACTCCAGCAGCACGTCCAAGCCCTGGTTCTCTTCATTTAGGAACTCTTGTACCCACCCAATGTGGTTGGTCCTCAGTGAAATCTCCAGCTCCCGCAAGACCTGTGTGGACTCCTGAACACGCCTCTTAAACCCCAGGTTTGTCATCCAGTCAGCTGTCACCTTTCTGCTGACTCCACCCGTCTCCAGGTAGCTCTTAAGTTTTTGGATATAGGCAGCTGGGGGGTTCTTGACTTGAAACCGCTCCTGGTCACAGATCAGCTCCCACTTTTTCTCATTGTCATATTGGCTCAGAAGTTGTATCTTGTCTGGGGGCAGGTTCATGCAGTTCAGGACGCGGTTAAACCTCTCCTCCAGCTCTCCGGCCCCGGGCATCGGCTGCTTCGGGGCTGCAGGCTGCTTGTGGGGCAGGGCGGCCGGAGCCGGGGGGTGATCCCCGCTGCCCGCCGCGTTGCCCATGGTGCTGCCCACCCAGCCCGCCAGACGCCTCCAGGCTCCGAAATCCAGCTTTCCAGGAGGTGGGGGCTCCAGACCTGCAGGGGTGCCCGGGGGCAGCAGCTCTGGCGGATGGGTGTCTCGGCGGCCCCGGGCGTTCAGTCCATGGTGCCCGGGCAGGCGGCTGGCAGCTGGGGGACCCTGGGCGCCGCGGGCGTCCCGTCCAGGCTGGCCCCCTGGGAGCTGCGGAGCTCAGCCGGGCGGGCGGGGCGGAGGAAGTCAGGCTGCGGGCGGCCAGGCCGGGGCCCGAGGGCTGGGGTTGGGGAGGGGGTGGGGGCGGCAGGAAGCGGCGGAGGCTGCCGGCGCCGGCTCCCCCCTCCGCGATCTTCTAACTTCTGGTCTCGTTCCCTTTACGCGCAAAGCCAAACCGCATTTCACTGCGCACCCAGC

>UoN.Pci.T.25720_UoN.Pci.T.25720.1

GGGAGCCCGAGCAGAGCCCAGCCAGGCGGGACCGGTGCACCAGGGCTGATTAGGCCCGAGCAGGTCAGCTTCACGGGGCCGGTTGAGGTCCGGTGTAGTGGAGCCACCCCCACCCACCATGAACTCGAGAAAGAGAGGGAGCCCATCTCGGACTCACTCGATGATGTCGCTGTCTGTTCGGCCCCAACGTCGCATTCTCAGCGCCAGGATCAGTAGGAGCCAGTCCTTCGCAGGGGTCTTGAGTGGCCAAGACCGGGGGGGCAGGAGCCTAACAGCCTTCAGCCCCCCTGTGGCTCCCCGAAAAACACCTGCCATCGTCCGAGTCTCCAGAATGTTTTCCATGGCACACCCACCTCCAAAGGTGCCTCAGCCAGAGCGGCTGGACCAGGTGTATGAAGCCCTGAAGAGGGGTCTAACGGCCTATCTGGAGGTGCATCAGTTAGAACAGGAGAAACTCCAAGTTCAAATCAGAGAATCCAAGAGAAACTCTAGGCTGGGCTTTCTCTATGAGTTGGACAAGCAAGTGAAATCTATCGAGCGTTTCCTCCGGCGCCTAGAGTTCCATGTCAGCAAGATTGATGAGCTGTATGACGGATATTGCATCCAGCGGCGGTTACGGGATGGAGCACACAACATGGTTCGGGCTTATACTTCAGGGACTCCTGGGAGTCGGGAGGCCAGGGACAGCCTGGCTGAGGCTACTCGTGGGCACCGGGAATACACAGAGAGCATGTGTTTGCTGGAAAGTGAACTTGAAGGACAATTAGGTGAATTCCACCTCAAAATGAAAGGGCTAGCTGGTTTTGCCCGGCTGTGTGTTGGGGACCAATACGAGATCTATATGAAATATGGGCGTCAGCGCTGGAAGCTACGTGGACGAATCGAAAGCAGTGGGAAGCAAGTCTGGGACAGCGAGGACATGGTCTTCCTGCCACTGCTCACAGAGTTCCTCTCCATCAAGGTCACAGAACTAAAGAGCTTGGCGAACCATGTGGTTGTGGGCAGTGTCTCTTGTGAGACCAAAGACCTGTTTGCTGCTCTGCCTCAGGTTGTCGCTGTGGATATCAATGACCTGGGCACTATCAAACTCAGCCTTGAGGTCACTTGGAGTCCCTTCGACAAGGATGACCAACCTTCAGCGGCGTCCACGGTCACCAAGACCTCTACTGTCAGCAAACGCTTTTCCACCTATAGCCAGAGTCCTCCAGATACCCCTTCACTGCGGGAACAGGCTTTCTATAACATGCTCCGTCGTCAGGAGGAGCTAGAGAACGGAACGGCTTGGTCTCTGTCCTCAGAATCTTCAGATGACTCCTCCAGCCCCCAGCTGTCAGGGGCTGCCCGCCTCACCCCACCCTCTCGGCCCCTGGTGCAACAACCCGAGGCTCCCCCGATCCACATTGCCTTCTCTCGACCAGAGATTCCCCATCCCTCAGGGGAGGCCCTGGAGGAGGAGGGGGCCAAAGCCCCAGTCTTGGCCAATGGGCACATCCCCTATAGCCGAACTCTGAGCCATATCAGTGAGGCCAGTGTAGATGCTGTGCTTGAGGCTACCAATGAATCTGTGGCCTCAGAGAGCCGTGCCCAGGGCCTCACCTCCTCTGTACACCTAGACTCTACTGACACTACCCCACTCCCTGTTCTAGAGGCACATCCAGACCCCTTCAACCCAAGCCCCACCCTCTCTTGTTTAGACCAGACCACTTTGCATGAAGGCCTTACCCCCTCACCCTCAGAACCAGTCACTTTAAATCTGAAGTCCTTTCCCTCACATCCAAGACCGTCTGAGTCTGATATTACCCAACTAGGCCCCCTCCCCCCCACCTCAGATCCCATATCCTCTTGCCCAGACTCCATCGCCTCCCATCCAGCGCCTGAAACCTTGCACTCTGACCATGGCTGTATCCACCCAGACTCTTCCCCCTCACACTCAGACCTTGCCCCTGTGTTCCCACTTACCAGTCCTGAACTCACAGACCATGTTTCTCCTTCCCCAGCCCCAACCCTACTGCACTCAGACCCTGCATCCCGACACTCAGATTCATCTTACCCCCATTCATACCCTGCCTCGTCACATACAGATCCCACCCCCACACAGTCACCCCTCACAGACCATGCCCCCAGTCCCCCTGTGGTGCCCCAGGTTCCAGTCCAGGAGGCAGCTGGTATTTCCCTAACAGAGGCCAAAGTTCTTCCTCAGAAGGGGCTGCTGGAGCAGGGAGCAGGGCTGAGGGACACAGGGCTGGAAGAAGCCCTGGGGGCCTTGAGCTCTGCCCTGGATGACTATCGGGGCCAATTTCCAGAGCTGCAAGGACTGGAGCAGGAGGTGACTCGGCTCGAGAGTCTACTTATGCAGAGACAAGGCATGTCCCGAAGCCGGGCATCCAGCCTCAGCCTCACTGTGGAGCATGCACTGGAGAGCTTTAGCTTCCTCAATGATGATGAAGATGAGGATGACGGGCCTGAGGACAGGTGAAGAAGGGGCTGGGGTACAGGTGAGGGAGGGACCGTTGAGAGATTAAGAACACAGAAGAGTGAAAGTGGAGGGGGAGCTGAGGGGAGGACAGTTAGGATTTACTGGGAAGGGGACCTGTATCTAACCTTTCTCTCCAGCTCTCCCCTGTGTTTCCATTTTCCCCAGTTACCCCCTTCCTGGGTCGTTGCCCCCACCCCGCCTTCAAGTGTCTTCCTTAGAGTCTTCAGGACACCCCCTCCCAGTACCCCTGATCTGTCTACCTCTAGCTCTGCCTCTCTCCCTCAGTCTCATTTTGGACCTGCTCTCTGGACATGCCCCCCCATCATCTCCTCACTCACCCATGGCTGGAAACTGGCCTCATCATCTTCATCTCTCCCCTTCGGCCCTCACTCCCTCAGCCCAACATAGGCGGAGGCTTTGGTCCCTTGATGAACTCCCTCGGAGCAGCTTTCGTACTGTCCACCTCACTGACCGAGTTTTGCCCCTTCTCCCCTCGCCGCTGCTCCAGCCCAAAGCCTCAGCACCTCTGTCCTCCCGCCAGAGCCCCTGTCCCTTCCTTTGGTGCTCTGTCAGACGGTCACTTCACCGACGGCCTTCCCATTGCCAACCTGGTGACCTCTCTTCCCAATACTCAAAATGCCAGCACCAGTCTTCTGCCCCTCTGACTACATTTACTCTCTGCCCTCCCTGAGATCCATTTCATCCCCACTCAAATACACTCCCATCACTACAACCCATGGTGGCCCCTGCTCAGAGGTCTCCACAATCCAGGAAGTCCAGTTATCGCAGCCATGTATGTGTATGTGAAGTCATCCTCCATGTGATGATTCATGTGCTGTTCTACACCAGCTATCCATCCTGTCTGTATCTAGAGGTGGGAGAGGGGGCTGAGGATGGGTATACATCTCAATAAATGTACACTGGGCACAGTGAGGGTGATACTCTGCCTCTGATGATCTGGATAGCAGGTGCTCACCACTCAGGTCTCTCCTGCAGGATGTTGAGCAGCCCAGGGCCTGGGTCTGAGGCTGGGGTTAGTGGTGTGTCTGAGGATGCCCTGGACTCCTCTACGCCTGGCCCCCTCAGCACTGGCTGCCCTGCCCTGGACACAGCCCTGGTCTTGCACCTACAGCACTGTGGCCACCTCTTGCTGGTGAGGCCAGCAGCCCTCCCCTCCAATACATTAACCCATTACTGACCCCAAGAGTCTAGCACACTAGGACCTTTAGCCTCCATTACCCCGTTGCACGCTTGTAATCTCCACCAGCATTTTTTTTAACCTCAGATGGACACACCTGCCCTTTGCATGCTGGGAGTTGCAGTCCCTGGGCTCCACACCATATTTCTAGGTCCCCTGTGCTACCCACCACCCCCAACCCTGAATAGGGGATGAGTAGAACTCCTACTCCCTGTGTCTGAGGTGGCCCTGGCTGGTCTCTGCTCTTCAGAAACTAGGCACCTTTGGGCCCCTACGATGCCAGGAAGCATCAGCATTGGAGCGGCTGCTTCGGGAGGCCCTGGTGCTGGAGGTGGTGTGCCAACTTTGTGGGGGGCAAGTGGGCAAGGCCACCTCTGCTCAGGAAGGTAAAGGTACCCACTCCCTTCCACCCTGGAGCAAAATCGGGAAATCTGCGTCTGTCCTTTCTCCAGAATTCTGCCTTTTGTCGTCGTGTCCCCCCCCCCCGAGACCTGCCCCCACTCTCCACCCCGCCCAACAAGCCCTCCATTCACCTACCCGCTGCAGCCCCTCATCGGCCCTCCTGGTCCCCCTGACTGTTTCCCCCCACGCCCCTCCCCCACCAGTGCTGCAGTTCTCTGTTCCCCGGCCGGGCTTCCTGCCTTTCTGGGACCTGTGCACTGAGGGAGGCAGCCTCTTGGTTTGTCCTGTGGAGCGCATCCTCTTCACCTTCTGCAGCCAGTATGGAGCCCGCCTCTCGCTGCGCCAGCCAGGCCTGGCCGAGACTGGTGAGTGACAGTCCCCGTCTTCCCCACGTGCTAGCCAAGCCCTCGCACAACCCCCTCACAAACATGTCCTTAGCGCGTACCGTGTTCAAGGCGGAGAATTAACTGAGACGCACCCAAAATGTATGGCATACTGGCCAAGCCCAGCGAGTGCGTGTGGAGATGGCGCGGCATGTCAGGCACAAAGATCTGGAGCTGGAAGGAACAGAAGAGGCCACCTTTGCCAGCTTCCCCATTTTATTAGAGGACCAAACTCAGCCCCTGAGAGGCCTTGTGACTCGCCGAGTTCACCCAGGGAGTGGGTGACAGATCTGGTACTCGAGCCCAGATCTCCTACGAATCACAGAGTTCTTGCTCTGATACCAGGCAGCTTACGTGTTTGGATGAGGGGTAGAGAGAGTCAGGGCTGCTGCAGGTCAGACAAGGGATCGAGCACTCCGGGATGGGTCTGGTCAGGAATCTGCAGCGTGGCTGGGGCGGCTGAGGCACACCTTCACCTGGGCACATCCAGAGGCTTCCATGACATCCCTCTTGAGTACCCCGTCCTTCCTGTCTCCTTTCCCAGTGTGTGTTAAGCTCCTGGAGGACGCTATGGGACAGAGGCTGCCCCGGAGGCCTCGGCCGGGGCCTGGGGAGCAGCTCACAATCTTCCAGTTCTGGAGCCACATCGAGGCCTTGAATAGCCCCTCCATGGAGGCTTATGTCACAGAGACAGCCGAAGAGGTGTTGATCGTGAAGAACCTGAATTCAGACGACCAGGCCGTGGTGCTCCGGGCACTGAGACTGGCCCCTGAGGGGCGGCTCCACCGGGATGGGCTCCGGGCCCTTAGCTCCCTTCTCATCCACGGTAACAGCAAAGTGATGGCTGCTGTCAGCACCCAGCTTCGAAGTTTAGCCCTGGGCTCGGACTTCCGGAAGAAGGCCCTGCTTTGTTTTTTGGAACAGCTAGAGGATGAGGATATGCAGACACGGGTGGCAGGGTGCGTGGCTCTGGGCTGTCTCAAGGCTCCAGAGGGCATTGAACCACTGGTGTATCTGTGCCAAACAGACAAGGAGGCTGTGAGGGAAGCTGCCCGGCAGAGCCTCCTGCAGTGTGGGGAGGATGGGCAGTCAGCTCACCGACGGCTGGAAGAGTCTCTGGAAGCTCTGCCCCGGATCTTCGGGCCAGGAAGCATGGCCAGCACCGCGTTCTGAGCTCCCTATCTGCTCTCTGTCCCAGGAACACAGGGGACTCGGGAGTCACTGCCTATTCCCTCAAGCCCCTCCCCCTACTACCACCTTTCAGGGCTGGCCCTGCCCTGGGAGCCCCCAACCTGATGGGCCCAACTGACATTGGCAGGGGTAGGCTGGGTGTCAGGACCCAGGCTCCAGGGTTCCCTCCCATCCTACAGATTCCTATAAATATAAATCTCTTTAATATATTCTTCTGCTACCCCTAGCAGAGACCGCCACCTGCAGTCAAGTGCCTCCCCAGCCTCCATCAGGCTTCCCTCACCACACGGTGTCTGGGATCCCTTCTAATCCCTGGTCACCCCGCCTCTCCCCTTCATGTCTGATTTTGTATTTTATTTAGAGTTTTGCAGAAAATAAAAGGCAAAATCTCTTTCCTATGCTGGGCAGCACCACCATTGTGGAGCACCCTGTCTTGCCTATCCTCCAACTCTAAACTCATCAAGGAAGAGAGGAGACTTGGAGAATCTGTGTCTTTTACTTCACCCATACTATTGTCACTGTTTCCCTGGGGACCCTGGTGTGTATTTCTGCCTATGCACCAAAAAGTGTGTTGATATAAGCAGAAGAATGTGTACCCAATAAGAGCTCACAGTAACATTTACAAAGCATCACACACACACACACACAAAACCTTATAGGTTAGTTTAAATTGGTCTTTTGAAGACCAAATACCCTCTTTTTCATCTTCTTAGAGCTTCAGATTAGATATTAAAACACTTCCTTATATTATTCATGCAGTACGACCAGAATGAAACACTGAATGTTCATTAGTATTACATGATAGTATAGTTATACTCAACAGTACTTTAAGTAAAAATCTTATGTCACAAAATATTTTCTTCAGGACTCCTAACTAGGTTTCTATGTTTTTATTACACATCACATTTGTTTAAACTTCTTTTGAGCTCCAGATTTTAAAAATATATTTATATACATGTTGTATATGTTTTTAATAATTCAATGAGTGGTCAGTCCTCATAATTATCAAAAGCTTATTTATACTGGGTCTAACAGCTAAACCAGCCCTTTGTTCTGTGGACAAGAAATTATGTTTAAAAAACATTTTTCTTGAAATGCTTGATTTTGATTTTATTCATTTCTCATAATATTTAAAAATAGGTTTGTCTGATACCCAGCAGTATAATTGAGTGGAAAATTTTAAGGTTCTGGCTATTTTCATTCAGAACATTTTAAATTTAGAGGTTATTTAACAAATCTGTGAATGCTTGGTAGTTAAAATAACTACAATGCATTATCATAAACTCTGTAAAAACAATGGCTTGCATGCATGCAGTTTAAAAAAAAAAGGACAGACAAGTATAAACACACTGCTGACTCTCTCTCCAGCTCATCTAATGCGTGTTTCCACAAGGGTTGTCTGAGATCTGGCAGCCCATGTTTTGGCCTGTCACCTGGACTTTGAACAGAGTTCCGTCTGCACACATACACAGCTTGTAGCGTACCCAGTCACCATTATTTAACTGATCTCCAAAGGAAGAACTTGGTAGGCGAGAGGTGCTGACCATCACAGATCCATTTAGGATGATGCTGTGTTCAGCTTTTAATTCCATATTACCACCCACGTGAAATTCAATGGTTTTGCCCATAATGAATACACCTTCATTTCCACGGACAATGGCACGTCCATCAACTTTTATATTTAAATCACTTGTAGCATTACTGGTAATCCTTTCAGTAGATGCTTTTTGTACATTCAAGCTCTTTACTCCACTTGGCAAATGAAATTCATGAGTTTCATAGTCTGTACTGAATAAAATATTTTGAGTCCTTGGGTCAAAAAACTGCATGCCAATGTCACTTGTAATAGAAGTTTTGTTCTTTTCTACACTGAGTTTCGTTGTTCCTTGCTGAAAAACAATTGGTTGGTTATTGCCAGTGATAACCAAATTTTCATTACGTCTGCCTCCTACTGTGCTCTTATAAAGAGGATGTATAACTCCCATATCAGATACTTGTTTAAATCGCAGCAAACCACTCTCATGGAACTCCATACTATCACAGCCATTGGGTCCAATACGGATCACAGCCCAGATAACAAGCGTTATAATTAAATTGATGACAGCCAAGATAAAAAGGAGAACAATCACACAGATGGCTAAATTGCCCTTTCTCCCTCTGAGGCCAGTTTTATGCAGCCGATCTTCATCAATTGGAATATAGCCAGCTTTAAAGTTACTGTTATGCTCTTTATTAACATTTCTTCGCTCAACAGCCTTCTCCCGCATAGACTTCTTTACAGGACCATTAGAACTTTGCTGCTCCGCTGCTGCAGCAGTCGCCGCCGTCGCCGCCATGTTCCCGCTCCCG

>UoN.Pci.T.25720_UoN.Pci.T.25720.2

GGGAGCCCGAGCAGAGCCCAGCCAGGCGGGACCGGTGCACCAGGGCTGATTAGGCCCGAGCAGGTCAGCTTCACGGGGCCGGTTGAGGTCCGGTGTAGTGGAGCCACCCCCACCCACCATGAACTCGAGAAAGAGAGGGAGCCCATCTCGGACTCACTCGATGATGTCGCTGTCTGTTCGGCCCCAACGTCGCATTCTCAGCGCCAGGATCAGTAGGAGCCAGTCCTTCGCAGGGGTCTTGAGTGGCCAAGACCGGGGGGGCAGGAGCCTAACAGCCTTCAGCCCCCCTGTGGCTCCCCGAAAAACACCTGCCATCGTCCGAGTCTCCAGAATGTTTTCCATGGCACACCCACCTCCAAAGGTGCCTCAGCCAGAGCGGCTGGACCAGGTGTATGAAGCCCTGAAGAGGGGTCTAACGGCCTATCTGGAGGTGCATCAGTTAGAACAGGAGAAACTCCAAGTTCAAATCAGAGAATCCAAGAGAAACTCTAGGCTGGGCTTTCTCTATGAGTTGGACAAGCAAGTGAAATCTATCGAGCGTTTCCTCCGGCGCCTAGAGTTCCATGTCAGCAAGATTGATGAGCTGTATGACGGATATTGCATCCAGCGGCGGTTACGGGATGGAGCACACAACATGGTTCGGGCTTATACTTCAGGGACTCCTGGGAGTCGGGAGGCCAGGGACAGCCTGGCTGAGGCTACTCGTGGGCACCGGGAATACACAGAGAGCATGTGTTTGCTGGAAAGTGAACTTGAAGGACAATTAGGTGAATTCCACCTCAAAATGAAAGGGCTAGCTGGTTTTGCCCGGCTGTGTGTTGGGGACCAATACGAGATCTATATGAAATATGGGCGTCAGCGCTGGAAGCTACGTGGACGAATCGAAAGCAGTGGGAAGCAAGTCTGGGACAGCGAGGACATGGTCTTCCTGCCACTGCTCACAGAGTTCCTCTCCATCAAGGTCACAGAACTAAAGAGCTTGGCGAACCATGTGGTTGTGGGCAGTGTCTCTTGTGAGACCAAAGACCTGTTTGCTGCTCTGCCTCAGGTTGTCGCTGTGGATATCAATGACCTGGGCACTATCAAACTCAGCCTTGAGGTCACTTGGAGTCCCTTCGACAAGGATGACCAACCTTCAGCGGCGTCCACGGTCACCAAGACCTCTACTGTCAGCAAACGCTTTTCCACCTATAGCCAGAGTCCTCCAGATACCCCTTCACTGCGGGAACAGGCTTTCTATAACATGCTCCGTCGTCAGGAGGAGCTAGAGAACGGAACGGCTTGGTCTCTGTCCTCAGAATCTTCAGATGACTCCTCCAGCCCCCAGCTGTCAGGGGCTGCCCGCCTCACCCCACCCTCTCGGCCCCTGGTGCAACAACCCGAGGCTCCCCCGATCCACATTGCCTTCTCTCGACCAGAGATTCCCCATCCCTCAGGGGAGGCCCTGGAGGAGGAGGGGGCCAAAGCCCCAGTCTTGGCCAATGGGCACATCCCCTATAGCCGAACTCTGAGCCATATCAGTGAGGCCAGTGTAGATGCTGTGCTTGAGGCTACCAATGAATCTGTGGCCTCAGAGAGCCGTGCCCAGGGCCTCACCTCCTCTGTACACCTAGACTCTACTGACACTACCCCACTCCCTGTTCTAGAGGCACATCCAGACCCCTTCAACCCAAGCCCCACCCTCTCTTGTTTAGACCAGACCACTTTGCATGAAGGCCTTACCCCCTCACCCTCAGAACCAGTCACTTTAAATCTGAAGTCCTTTCCCTCACATCCAAGACCGTCTGAGTCTGATATTACCCAACTAGGCCCCCTCCCCCCCACCTCAGATCCCATATCCTCTTGCCCAGACTCCATCGCCTCCCATCCAGCGCCTGAAACCTTGCACTCTGACCATGGCTGTATCCACCCAGACTCTTCCCCCTCACACTCAGACCTTGCCCCTGTGTTCCCACTTACCAGTCCTGAACTCACAGACCATGTTTCTCCTTCCCCAGCCCCAACCCTACTGCACTCAGACCCTGCATCCCGACACTCAGATTCATCTTACCCCCATTCATACCCTGCCTCGTCACATACAGATCCCACCCCCACACAGTCACCCCTCACAGACCATGCCCCCAGTCCCCCTGTGGTGCCCCAGGTTCCAGTCCAGGAGGCAGCTGGTATTTCCCTAACAGAGGCCAAAGTTCTTCCTCAGAAGGGGCTGCTGGAGCAGGGAGCAGGGCTGAGGGACACAGGGCTGGAAGAAGCCCTGGGGGCCTTGAGCTCTGCCCTGGATGACTATCGGGGCCAATTTCCAGAGCTGCAAGGACTGGAGCAGGAGGTGACTCGGCTCGAGAGTCTACTTATGCAGAGACAAGGCATGTCCCGAAGCCGGGCATCCAGCCTCAGCCTCACTGTGGAGCATGCACTGGAGAGCTTTAGCTTCCTCAATGATGATGAAGATGAGGATGACGGGCCTGAGGACAGGTGAAGAAGGGGCTGGGGTACAGGTGAGGGAGGGACCGTTGAGAGATTAAGAACACAGAAGAGTGAAAGTGGAGGGGGAGCTGAGGGGAGGACAGTTAGGATTTACTGGGAAGGGGACCTGTATCTAACCTTTCTCTCCAGCTCTCCCCTGTGTTTCCATTTTCCCCAGTTACCCCCTTCCTGGGTCGTTGCCCCCACCCCGCCTTCAAGTGTCTTCCTTAGAGTCTTCAGGACACCCCCTCCCAGTACCCCTGATCTGTCTACCTCTAGCTCTGCCTCTCTCCCTCAGTCTCATTTTGGACCTGCTCTCTGGACATGCCCCCCCATCATCTCCTCACTCACCCATGGCTGGAAACTGGCCTCATCATCTTCATCTCTCCCCTTCGGCCCTCACTCCCTCAGCCCAACATAGGCGGAGGCTTTGGTCCCTTGATGAACTCCCTCGGAGCAGCTTTCGTACTGTCCACCTCACTGACCGAGTTTTGCCCCTTCTCCCCTCGCCGCTGCTCCAGCCCAAAGCCTCAGCACCTCTGTCCTCCCGCCAGAGCCCCTGTCCCTTCCTTTGGTGCTCTGTCAGACGGTCACTTCACCGACGGCCTTCCCATTGCCAACCTGGTGACCTCTCTTCCCAATACTCAAAATGCCAGCACCAGTCTTCTGCCCCTCTGACTACATTTACTCTCTGCCCTCCCTGAGATCCATTTCATCCCCACTCAAATACACTCCCATCACTACAACCCATGGTGGCCCCTGCTCAGAGGTCTCCACAATCCAGGAAGTCCAGTTATCGCAGCCATGTATGTGTATGTGAAGTCATCCTCCATGTGATGATTCATGTGCTGTTCTACACCAGCTATCCATCCTGTCTGTATCTAGAGGTGGGAGAGGGGGCTGAGGATGGGTATACATCTCAATAAATGTACACTGGGCACAGTGAGGGTGATACTCTGCCTCTGATGATCTGGATAGCAGGTGCTCACCACTCAGGTCTCTCCTGCAGGATGTTGAGCAGCCCAGGGCCTGGGTCTGAGGCTGGGGTTAGTGGTGTGTCTGAGGATGCCCTGGACTCCTCTACGCCTGGCCCCCTCAGCACTGGCTGCCCTGCCCTGGACACAGCCCTGGTCTTGCACCTACAGCACTGTGGCCACCTCTTGCTGGTGAGGCCAGCAGCCCTCCCCTCCAATACATTAACCCATTACTGACCCCAAGAGTCTAGCACACTAGGACCTTTAGCCTCCATTACCCCGTTGCACGCTTGTAATCTCCACCAGCATTTTTTTTAACCTCAGATGGACACACCTGCCCTTTGCATGCTGGGAGTTGCAGTCCCTGGGCTCCACACCATATTTCTAGGTCCCCTGTGCTACCCACCACCCCCAACCCTGAATAGGGGATGAGTAGAACTCCTACTCCCTGTGTCTGAGGTGGCCCTGGCTGGTCTCTGCTCTTCAGAAACTAGGCACCTTTGGGCCCCTACGATGCCAGGAAGCATCAGCATTGGAGCGGCTGCTTCGGGAGGCCCTGGTGCTGGAGGTGGTGTGCCAACTTTGTGGGGGGCAAGTGGGCAAGGCCACCTCTGCTCAGGAAGGTAAAGGTACCCACTCCCTTCCACCCTGGAGCAAAATCGGGAAATCTGCGTCTGTCCTTTCTCCAGAATTCTGCCTTTTGTCGTCGTGTCCCCCCCCCCCGAGACCTGCCCCCACTCTCCACCCCGCCCAACAAGCCCTCCATTCACCTACCCGCTGCAGCCCCTCATCGGCCCTCCTGGTCCCCCTGACTGTTTCCCCCCACGCCCCTCCCCCACCAGTGCTGCAGTTCTCTGTTCCCCGGCCGGGCTTCCTGCCTTTCTGGGACCTGTGCACTGAGGGAGGCAGCCTCTTGGTTTGTCCTGTGGAGCGCATCCTCTTCACCTTCTGCAGCCAGTATGGAGCCCGCCTCTCGCTGCGCCAGCCAGGCCTGGCCGAGACTGGTGAGTGACAGTCCCCGTCTTCCCCACGTGCTAGCCAAGCCCTCGCACAACCCCCTCACAAACATGTCCTTAGCGCGTACCGTGTTCAAGGCGGAGAATTAACTGAGACGCACCCAAAATGTATGGCATACTGGCCAAGCCCAGCGAGTGCGTGTGGAGATGGCGCGGCATGTCAGGCACAAAGATCTGGAGCTGGAAGGAACAGAAGAGGCCACCTTTGCCAGCTTCCCCATTTTATTAGAGGACCAAACTCAGCCCCTGAGAGGCCTTGTGACTCGCCGAGTTCACCCAGGGAGTGGGTGACAGATCTGGTACTCGAGCCCAGATCTCCTACGAATCACAGAGTTCTTGCTCTGATACCAGGCAGCTTACGTGTTTGGATGAGGGGTAGAGAGAGTCAGGGCTGCTGCAGGTCAGACAAGGGATCGAGCACTCCGGGATGGGTCTGGTCAGGAATCTGCAGCGTGGCTGGGGCGGCTGAGGCACACCTTCACCTGGGCACATCCAGAGGCTTCCATGACATCCCTCTTGAGTACCCCGTCCTTCCTGTCTCCTTTCCCAGTGTGTGTTAAGCTCCTGGAGGACGCTATGGGACAGAGGCTGCCCCGGAGGCCTCGGCCGGGGCCTGGGGAGCAGCTCACAATCTTCCAGTTCTGGAGCCACATCGAGGCCTTGAATAGCCCCTCCATGGAGGCTTATGTCACAGAGACAGCCGAAGAGGTGTTGATCGTGAAGAACCTGAATTCAGACGACCAGGCCGTGGTGCTCCGGGCACTGAGACTGGCCCCTGAGGGGCGGCTCCACCGGGATGGGCTCCGGGCCCTTAGCTCCCTTCTCATCCACGGTAACAGCAAAGTGATGGCTGCTGTCAGCACCCAGCTTCGAAGTTTAGCCCTGGGCTCGGACTTCCGGAAGAAGGCCCTGCTTTGTTTTTTGGAACAGCTAGAGGATGAGGATATGCAGACACGGGTGGCAGGGTGCGTGGCTCTGGGCTGTCTCAAGGCTCCAGAGGGCATTGAACCACTGGTGTATCTGTGCCAAACAGACAAGGAGGCTGTGAGGGAAGCTGCCCGGCAGAGCCTCCTGCAGTGTGGGGAGGATGGGCAGTCAGCTCACCGACGGCTGGAAGAGTCTCTGGAAGCTCTGCCCCGGATCTTCGGGCCAGGAAGCATGGCCAGCACCGCGTTCTGAGCTCCCTATCTGCTCTCTGTCCCAGGAACACAGGGGACTCGGGAGTCACTGCCTATTCCCTCAAGCCCCTCCCCCTACTACCACCTTTCAGGGCTGGCCCTGCCCTGGGAGCCCCCAACCTGATGGGCCCAACTGACATTGGCAGGGGTAGGCTGGGTGTCAGGACCCAGGCTCCAGGGTTCCCTCCCATCCTACAGATTCCTATAAATATAAATCTCTTTAATATATTCTTCTGCTACCCCTAGCAGAGACCGCCACCTGCAGTCAAGTGCCTCCCCAGCCTCCATCAGGCTTCCCTCACCACACGGTGTCTGGGATCCCTTCTAATCCCTGGTCACCCCGCCTCTCCCCTTCATGTCTGATTTTGTATTTTATTTAGAGTTTTGCAGAAAATAAAAGGCAAAATCTCTTTCCTATGCTGGGCAGCACCACCATTGTGGAGCACCCTGTCTTGCCTATCCTCCAACTCTAAACTCATCAAGGAAGAGAGGAGACTTGGAGAATCTGTGTCTTTTACTTCACCCATACTATTGTCACTGTTTCCCTGGGGACCCTGGTGTGTATTTCTGCCTATGCACCAAAAAGTGTGTTGATATAAGCAGAAGAATGTGTACCCAATAAGAGCTCACAGTAACATTTACAAAGCATCACACACACACACACACAAAACCTTATAGGTTAGTTTAAATTGGTCTTTTGAAGACCAAATACCCTCTTTTTCATCTTCTTAGAGCTTCAGATTAGATATTAAAACACTTCCTTATATTATTCATGCAGTACGACCAGAATGAAACACTGAATGTTCATTAGTATTACATGATAGTATAGTTATACTCAACAGTACTTTAAGTAAAAATCTTATGTCACAAAATATTTTCTTCAGGACTCCTAACTAGGTTTCTATGTTTTTATTACACATCACATTTGTTTAAACTTCTTTTGAGCTCCAGATTTTAAAAATATATTTATATACATGTTGTATATGTTTTTAATAATTCAATGAGTGGTCAGTCCTCATAATTATCAAAAGCTTATTTATACTGGGTCTAACAGCTAAACCAGCCCTTTGTTCTGTGGACAAGAAATTATGTTTAAAAAACATTTTTCTTGAAATGCTTGATTTTGATTTTATTCATTTCTCATAATATTTAAAAATAGGTTTGTCTGATACCCAGCAGTATAATTGAGTGGAAAATTTTAAGGTTCTGGCTATTTTCATTCAGAACATTTTAAATTTAGAGGTTATTTAACAAATCTGTGAATGCTTGGTAGTTAAAATAACTACAATGCATTATCATAAACTCTGTAAAAACAATGGCTTGCATGCATGCAGTTTAAAAAAAAAAGGACAGACAAGTATAAACACACTGCTGACTCTCTCTCCAGCTCATCTAATGCGTGTTTCCACAAGGGTTGTCTGAGATCTGGCAGCCCATGTTTTGGCCTGTCACCTGGACTTTGAACAGAGTTCCGTCTGCACACATACACAGCTTGTAGCGTACCCAGTCACCATTATTTAACTGATCTCCAAAGGAAGAACTTGGTAGGCGAGAGGTGCTGACCATCACAGATCCATTTAGGATGATGCTGTGTTCAGCTTTTAATTCCATATTACCACCCACGTGAAATTCAATGGTTTTGCCCATAATGAATACACCTTCATTTCCACGGACAATGGCACGTCCATCAACTTTTATATTTAAATCACTTGTAGCATTACTGGTAATCCTTTCAGTAGATGCTTTTTGTACATTCAAGCTCTTTACTCCACTTGGCAAATGAAATTCATGAGTTTCATAGTCTGTACTGAATAAAATATTTTGAGTCCTTGGGTCAAAAAACTGCATGCCAATGTCACTTGTAATAGAAGTTTTGTTCTTTTCTACACTGAGTTTCGTTGTTCCTTGCTGAAAAACAATTGGTTGGTTATTGCCAGTGATAACCAAATTTTCATTACGTCTGCCTCCTACTGTGCTCTTATAAAGAGGATGTATAACTCCCATATCAGATACTTGTTTAAATCGCAGCAAACCACTCTCATGGAACTCCATACTATCACAGCCATTGGGTCCAATACGGATCACAGCCCAGATAACAAGCGTTATAATTAAATTGATGACAGCCAAGATAAAAAGGAGAACAATCACACAGATGGCTAAATTGCCCTTTCTCCCTCTGAGGCCAGTTTTATGCAGCCGATCTTCATCAATTGGAATATAGCCAGCTTTAAAGTTACTGTTATGCTCTTTATTAACATTTCTTCGCTCAACAGCCTTCTCCCGCATAGACTTCTTTACAGGACCATTAGAACTTTGCTGCTCCGCTGCTGCAGCAGTCGCCGCCGTCGCCGCCATGTTCCCGCTCCC

>UoN.Pci.T.25720_UoN.Pci.T.25720.3

GGGAGCCCGAGCAGAGCCCAGCCAGGCGGGACCGGTGCACCAGGGCTGATTAGGCCCGAGCAGGTCAGCTTCACGGGGCCGGTTGAGGTCCGGTGTAGTGGAGCCACCCCCACCCACCATGAACTCGAGAAAGAGAGGGAGCCCATCTCGGACTCACTCGATGATGTCGCTGTCTGTTCGGCCCCAACGTCGCATTCTCAGCGCCAGGATCAGTAGGAGCCAGTCCTTCGCAGGGGTCTTGAGTGGCCAAGACCGGGGGGGCAGGAGCCTAACAGCCTTCAGCCCCCCTGTGGCTCCCCGAAAAACACCTGCCATCGTCCGAGTCTCCAGAATGTTTTCCATGGCACACCCACCTCCAAAGGTGCCTCAGCCAGAGCGGCTGGACCAGGTGTATGAAGCCCTGAAGAGGGGTCTAACGGCCTATCTGGAGGTGCATCAGTTAGAACAGGAGAAACTCCAAGTTCAAATCAGAGAATCCAAGAGAAACTCTAGGCTGGGCTTTCTCTATGAGTTGGACAAGCAAGTGAAATCTATCGAGCGTTTCCTCCGGCGCCTAGAGTTCCATGTCAGCAAGATTGATGAGCTGTATGACGGATATTGCATCCAGCGGCGGTTACGGGATGGAGCACACAACATGGTTCGGGCTTATACTTCAGGGACTCCTGGGAGTCGGGAGGCCAGGGACAGCCTGGCTGAGGCTACTCGTGGGCACCGGGAATACACAGAGAGCATGTGTTTGCTGGAAAGTGAACTTGAAGGACAATTAGGTGAATTCCACCTCAAAATGAAAGGGCTAGCTGGTTTTGCCCGGCTGTGTGTTGGGGACCAATACGAGATCTATATGAAATATGGGCGTCAGCGCTGGAAGCTACGTGGACGAATCGAAAGCAGTGGGAAGCAAGTCTGGGACAGCGAGGACATGGTCTTCCTGCCACTGCTCACAGAGTTCCTCTCCATCAAGGTCACAGAACTAAAGAGCTTGGCGAACCATGTGGTTGTGGGCAGTGTCTCTTGTGAGACCAAAGACCTGTTTGCTGCTCTGCCTCAGGTTGTCGCTGTGGATATCAATGACCTGGGCACTATCAAACTCAGCCTTGAGGTCACTTGGAGTCCCTTCGACAAGGATGACCAACCTTCAGCGGCGTCCACGGTCACCAAGACCTCTACTGTCAGCAAACGCTTTTCCACCTATAGCCAGAGTCCTCCAGATACCCCTTCACTGCGGGAACAGGCTTTCTATAACATGCTCCGTCGTCAGGAGGAGCTAGAGAACGGAACGGCTTGGTCTCTGTCCTCAGAATCTTCAGATGACTCCTCCAGCCCCCAGCTGTCAGGGGCTGCCCGCCTCACCCCACCCTCTCGGCCCCTGGTGCAACAACCCGAGGCTCCCCCGATCCACATTGCCTTCTCTCGACCAGAGATTCCCCATCCCTCAGGGGAGGCCCTGGAGGAGGAGGGGGCCAAAGCCCCAGTCTTGGCCAATGGGCACATCCCCTATAGCCGAACTCTGAGCCATATCAGTGAGGCCAGTGTAGATGCTGTGCTTGAGGCTACCAATGAATCTGTGGCCTCAGAGAGCCGTGCCCAGGGCCTCACCTCCTCTGTACACCTAGACTCTACTGACACTACCCCACTCCCTGTTCTAGAGGCACATCCAGACCCCTTCAACCCAAGCCCCACCCTCTCTTGTTTAGACCAGACCACTTTGCATGAAGGCCTTACCCCCTCACCCTCAGAACCAGTCACTTTAAATCTGAAGTCCTTTCCCTCACATCCAAGACCGTCTGAGTCTGATATTACCCAACTAGGCCCCCTCCCCCCCACCTCAGATCCCATATCCTCTTGCCCAGACTCCATCGCCTCCCATCCAGCGCCTGAAACCTTGCACTCTGACCATGGCTGTATCCACCCAGACTCTTCCCCCTCACACTCAGACCTTGCCCCTGTGTTCCCACTTACCAGTCCTGAACTCACAGACCATGTTTCTCCTTCCCCAGCCCCAACCCTACTGCACTCAGACCCTGCATCCCGACACTCAGATTCATCTTACCCCCATTCATACCCTGCCTCGTCACATACAGATCCCACCCCCACACAGTCACCCCTCACAGACCATGCCCCCAGTCCCCCTGTGGTGCCCCAGGTTCCAGTCCAGGAGGCAGCTGGTATTTCCCTAACAGAGGCCAAAGTTCTTCCTCAGAAGGGGCTGCTGGAGCAGGGAGCAGGGCTGAGGGACACAGGGCTGGAAGAAGCCCTGGGGGCCTTGAGCTCTGCCCTGGATGACTATCGGGGCCAATTTCCAGAGCTGCAAGGACTGGAGCAGGAGGTGACTCGGCTCGAGAGTCTACTTATGCAGAGACAAGGCATGTCCCGAAGCCGGGCATCCAGCCTCAGCCTCACTGTGGAGCATGCACTGGAGAGCTTTAGCTTCCTCAATGATGATGAAGATGAGGATGACGGGCCTGAGGACAGGTGAAGAAGGGGCTGGGGTACAGGTGAGGGAGGGACCGTTGAGAGATTAAGAACACAGAAGAGTGAAAGTGGAGGGGGAGCTGAGGGGAGGACAGTTAGGATTTACTGGGAAGGGGACCTGTATCTAACCTTTCTCTCCAGCTCTCCCCTGTGTTTCCATTTTCCCCAGTTACCCCCTTCCTGGGTCGTTGCCCCCACCCCGCCTTCAAGTGTCTTCCTTAGAGTCTTCAGGACACCCCCTCCCAGTACCCCTGATCTGTCTACCTCTAGCTCTGCCTCTCTCCCTCAGTCTCATTTTGGACCTGCTCTCTGGACATGCCCCCCCATCATCTCCTCACTCACCCATGGCTGGAAACTGGCCTCATCATCTTCATCTCTCCCCTTCGGCCCTCACTCCCTCAGCCCAACATAGGCGGAGGCTTTGGTCCCTTGATGAACTCCCTCGGAGCAGCTTTCGTACTGTCCACCTCACTGACCGAGTTTTGCCCCTTCTCCCCTCGCCGCTGCTCCAGCCCAAAGCCTCAGCACCTCTGTCCTCCCGCCAGAGCCCCTGTCCCTTCCTTTGGTGCTCTGTCAGACGGTCACTTCACCGACGGCCTTCCCATTGCCAACCTGGTGACCTCTCTTCCCAATACTCAAAATGCCAGCACCAGTCTTCTGCCCCTCTGACTACATTTACTCTCTGCCCTCCCTGAGATCCATTTCATCCCCACTCAAATACACTCCCATCACTACAACCCATGGTGGCCCCTGCTCAGAGGTCTCCACAATCCAGGAAGTCCAGTTATCGCAGCCATGTATGTGTATGTGAAGTCATCCTCCATGTGATGATTCATGTGCTGTTCTACACCAGCTATCCATCCTGTCTGTATCTAGAGGTGGGAGAGGGGGCTGAGGATGGGTATACATCTCAATAAATGTACACTGGGCACAGTGAGGGTGATACTCTGCCTCTGATGATCTGGATAGCAGGTGCTCACCACTCAGGTCTCTCCTGCAGGATGTTGAGCAGCCCAGGGCCTGGGTCTGAGGCTGGGGTTAGTGGTGTGTCTGAGGATGCCCTGGACTCCTCTACGCCTGGCCCCCTCAGCACTGGCTGCCCTGCCCTGGACACAGCCCTGGTCTTGCACCTACAGCACTGTGGCCACCTCTTGCTGGTGAGGCCAGCAGCCCTCCCCTCCAATACATTAACCCATTACTGACCCCAAGAGTCTAGCACACTAGGACCTTTAGCCTCCATTACCCCGTTGCACGCTTGTAATCTCCACCAGCATTTTTTTTAACCTCAGATGGACACACCTGCCCTTTGCATGCTGGGAGTTGCAGTCCCTGGGCTCCACACCATATTTCTAGGTCCCCTGTGCTACCCACCACCCCCAACCCTGAATAGGGGATGAGTAGAACTCCTACTCCCTGTGTCTGAGGTGGCCCTGGCTGGTCTCTGCTCTTCAGAAACTAGGCACCTTTGGGCCCCTACGATGCCAGGAAGCATCAGCATTGGAGCGGCTGCTTCGGGAGGCCCTGGTGCTGGAGGTGGTGTGCCAACTTTGTGGGGGGCAAGTGGGCAAGGCCACCTCTGCTCAGGAAGGTAAAGGTACCCACTCCCTTCCACCCTGGAGCAAAATCGGGAAATCTGCGTCTGTCCTTTCTCCAGAATTCTGCCTTTTGTCGTCGTGTCCCCCCCCCCCGAGACCTGCCCCCACTCTCCACCCCGCCCAACAAGCCCTCCATTCACCTACCCGCTGCAGCCCCTCATCGGCCCTCCTGGTCCCCCTGACTGTTTCCCCCCACGCCCCTCCCCCACCAGTGCTGCAGTTCTCTGTTCCCCGGCCGGGCTTCCTGCCTTTCTGGGACCTGTGCACTGAGGGAGGCAGCCTCTTGGTTTGTCCTGTGGAGCGCATCCTCTTCACCTTCTGCAGCCAGTATGGAGCCCGCCTCTCGCTGCGCCAGCCAGGCCTGGCCGAGACTGGTGAGTGACAGTCCCCGTCTTCCCCACGTGCTAGCCAAGCCCTCGCACAACCCCCTCACAAACATGTCCTTAGCGCGTACCGTGTTCAAGGCGGAGAATTAACTGAGACGCACCCAAAATGTATGGCATACTGGCCAAGCCCAGCGAGTGCGTGTGGAGATGGCGCGGCATGTCAGGCACAAAGATCTGGAGCTGGAAGGAACAGAAGAGGCCACCTTTGCCAGCTTCCCCATTTTATTAGAGGACCAAACTCAGCCCCTGAGAGGCCTTGTGACTCGCCGAGTTCACCCAGGGAGTGGGTGACAGATCTGGTACTCGAGCCCAGATCTCCTACGAATCACAGAGTTCTTGCTCTGATACCAGGCAGCTTACGTGTTTGGATGAGGGGTAGAGAGAGTCAGGGCTGCTGCAGGTCAGACAAGGGATCGAGCACTCCGGGATGGGTCTGGTCAGGAATCTGCAGCGTGGCTGGGGCGGCTGAGGCACACCTTCACCTGGGCACATCCAGAGGCTTCCATGACATCCCTCTTGAGTACCCCGTCCTTCCTGTCTCCTTTCCCAGTGTGTGTTAAGCTCCTGGAGGACGCTATGGGACAGAGGCTGCCCCGGAGGCCTCGGCCGGGGCCTGGGGAGCAGCTCACAATCTTCCAGTTCTGGAGCCACATCGAGGCCTTGAATAGCCCCTCCATGGAGGCTTATGTCACAGAGACAGCCGAAGAGGTGTTGATCGTGAAGAACCTGAATTCAGACGACCAGGCCGTGGTGCTCCGGGCACTGAGACTGGCCCCTGAGGGGCGGCTCCACCGGGATGGGCTCCGGGCCCTTAGCTCCCTTCTCATCCACGGTAACAGCAAAGTGATGGCTGCTGTCAGCACCCAGCTTCGAAGTTTAGCCCTGGGCTCGGACTTCCGGAAGAAGGCCCTGCTTTGTTTTTTGGAACAGCTAGAGGATGAGGATATGCAGACACGGGTGGCAGGGTGCGTGGCTCTGGGCTGTCTCAAGGCTCCAGAGGGCATTGAACCACTGGTGTATCTGTGCCAAACAGACAAGGAGGCTGTGAGGGAAGCTGCCCGGCAGAGCCTCCTGCAGTGTGGGGAGGATGGGCAGTCAGCTCACCGACGGCTGGAAGAGTCTCTGGAAGCTCTGCCCCGGATCTTCGGGCCAGGAAGCATGGCCAGCACCGCGTTCTGAGCTCCCTATCTGCTCTCTGTCCCAGGAACACAGGGGACTCGGGAGTCACTGCCTATTCCCTCAAGCCCCTCCCCCTACTACCACCTTTCAGGGCTGGCCCTGCCCTGGGAGCCCCCAACCTGATGGGCCCAACTGACATTGGCAGGGGTAGGCTGGGTGTCAGGACCCAGGCTCCAGGGTTCCCTCCCATCCTACAGATTCCTATAAATATAAATCTCTTTAATATATTCTTCTGCTACCCCTAGCAGAGACCGCCACCTGCAGTCAAGTGCCTCCCCAGCCTCCATCAGGCTTCCCTCACCACACGGTGTCTGGGATCCCTTCTAATCCCTGGTCACCCCGCCTCTCCCCTTCATGTCTGATTTTGTATTTTATTTAGAGTTTTGCAGAAAATAAAAGGCAAAATCTCTTTCCTATGCTGGGCAGCACCACCATTGTGGAGCACCCTGTCTTGCCTATCCTCCAACTCTAAACTCATCAAGGAAGAGAGGAGACTTGGAGAATCTGTGTCTTTTACTTCACCCATACTATTGTCACTGTTTCCCTGGGGACCCTGGTGTGTATTTCTGCCTATGCACCAAAAAGTGTGTTGATATAAGCAGAAGAATGTGTACCCAATAAGAGCTCACAGTAACATTTACAAAGCATCACACACACACACACACAAAACCTTATAGGTTAGTTTAAATTGGTCTTTTGAAGACCAAATACCCTCTTTTTCATCTTCTTAGAGCTTCAGATTAGATATTAAAACACTTCCTTATATTATTCATGCAGTACGACCAGAATGAAACACTGAATGTTCATTAGTATTACATGATAGTATAGTTATACTCAACAGTACTTTAAGTAAAAATCTTATGTCACAAAATATTTTCTTCAGGACTCCTAACTAGGTTTCTATGTTTTTATTACACATCACATTTGTTTAAACTTCTTTTGAGCTCCAGATTTTAAAAATATATTTATATACATGTTGTATATGTTTTTAATAATTCAATGAGTGGTCAGTCCTCATAATTATCAAAAGCTTATTTATACTGGGTCTAACAGCTAAACCAGCCCTTTGTTCTGTGGACAAGAAATTATGTTTAAAAAACATTTTTCTTGAAATGCTTGATTTTGATTTTATTCATTTCTCATAATATTTAAAAATAGGTTTGTCTGATACCCAGCAGTATAATTGAGTGGAAAATTTTAAGGTTCTGGCTATTTTCATTCAGAACATTTTAAATTTAGAGGTTATTTAACAAATCTGTGAATGCTTGGTAGTTAAAATAACTACAATGCATTATCATAAACTCTGTAAAAACAATGGCTTGCATGCATGCAGTTTAAAAAAAAAAGGACAGACAAGTATAAACACACTGCTGACTCTCTCTCCAGCTCATCTAATGCGTGTTTCCACAAGGGTTGTCTGAGATCTGGCAGCCCATGTTTTGGCCTGTCACCTGGACTTTGAACAGAGTTCCGTCTGCACACATACACAGCTTGTAGCGTACCCAGTCACCATTATTTAACTGATCTCCAAAGGAAGAACTTGGTAGGCGAGAGGTGCTGACCATCACAGATCCATTTAGGATGATGCTGTGTTCAGCTTTTAATTCCATATTACCACCCACGTGAAATTCAATGGTTTTGCCCATAATGAATACACCTTCATTTCCACGGACAATGGCACGTCCATCAACTTTTATATTTAAATCACTTGTAGCATTACTGGTAATCCTTTCAGTAGATGCTTTTTGTACATTCAAGCTCTTTACTCCACTTGGCAAATGAAATTCATGAGTTTCATAGTCTGTACTGAATAAAATATTTTGAGTCCTTGGGTCAAAAAACTGCATGCCAATGTCACTTGTAATAGAAGTTTTGTTCTTTTCTACACTGAGTTTCGTTGTTCCTTGCTGAAAAACAATTGGTTGGTTATTGCCAGTGATAACCAAATTTTCATTACGTCTGCCTCCTACTGTGCTCTTATAAAGAGGATGTATAACTCCCATATCAGATACTTGTTTAAATCGCAGCAAACCACTCTCATGGAACTCCATACTATCACAGCCATTGGGTCCAATACGGATCACAGCCCAGATAACAAGCGTTATAATTAAATTGATGACAGCCAAGATAAAAAGGAGAACAATCACACAGATGGCTAAATTGCCCTTTCTCCCTCTGAGGCCAGTTTTATGCAGCCGATCTTCATCAATTGGAATATAGCCAGCTTTAAAGTTACTGTTATGCTCTTTATTAACATTTCTTCGCTCAACAGCCTTCTCCCGCATAGACTTCTTTACAGGACCATTAGAACTTTGCTGCTCCGCTGCTGCAGCAGTCGCCGCCGTCGCCGCCATGTTCCCGCTCC

>UoN.Pci.T.25720_UoN.Pci.T.25720.4

GGGAGCCCGAGCAGAGCCCAGCCAGGCGGGACCGGTGCACCAGGGCTGATTAGGCCCGAGCAGGTCAGCTTCACGGGGCCGGTTGAGGTCCGGTGTAGTGGAGCCACCCCCACCCACCATGAACTCGAGAAAGAGAGGGAGCCCATCTCGGACTCACTCGATGATGTCGCTGTCTGTTCGGCCCCAACGTCGCATTCTCAGCGCCAGGATCAGTAGGAGCCAGTCCTTCGCAGGGGTCTTGAGTGGCCAAGACCGGGGGGGCAGGAGCCTAACAGCCTTCAGCCCCCCTGTGGCTCCCCGAAAAACACCTGCCATCGTCCGAGTCTCCAGAATGTTTTCCATGGCACACCCACCTCCAAAGGTGCCTCAGCCAGAGCGGCTGGACCAGGTGTATGAAGCCCTGAAGAGGGGTCTAACGGCCTATCTGGAGGTGCATCAGTTAGAACAGGAGAAACTCCAAGTTCAAATCAGAGAATCCAAGAGAAACTCTAGGCTGGGCTTTCTCTATGAGTTGGACAAGCAAGTGAAATCTATCGAGCGTTTCCTCCGGCGCCTAGAGTTCCATGTCAGCAAGATTGATGAGCTGTATGACGGATATTGCATCCAGCGGCGGTTACGGGATGGAGCACACAACATGGTTCGGGCTTATACTTCAGGGACTCCTGGGAGTCGGGAGGCCAGGGACAGCCTGGCTGAGGCTACTCGTGGGCACCGGGAATACACAGAGAGCATGTGTTTGCTGGAAAGTGAACTTGAAGGACAATTAGGTGAATTCCACCTCAAAATGAAAGGGCTAGCTGGTTTTGCCCGGCTGTGTGTTGGGGACCAATACGAGATCTATATGAAATATGGGCGTCAGCGCTGGAAGCTACGTGGACGAATCGAAAGCAGTGGGAAGCAAGTCTGGGACAGCGAGGACATGGTCTTCCTGCCACTGCTCACAGAGTTCCTCTCCATCAAGGTCACAGAACTAAAGAGCTTGGCGAACCATGTGGTTGTGGGCAGTGTCTCTTGTGAGACCAAAGACCTGTTTGCTGCTCTGCCTCAGGTTGTCGCTGTGGATATCAATGACCTGGGCACTATCAAACTCAGCCTTGAGGTCACTTGGAGTCCCTTCGACAAGGATGACCAACCTTCAGCGGCGTCCACGGTCACCAAGACCTCTACTGTCAGCAAACGCTTTTCCACCTATAGCCAGAGTCCTCCAGATACCCCTTCACTGCGGGAACAGGCTTTCTATAACATGCTCCGTCGTCAGGAGGAGCTAGAGAACGGAACGGCTTGGTCTCTGTCCTCAGAATCTTCAGATGACTCCTCCAGCCCCCAGCTGTCAGGGGCTGCCCGCCTCACCCCACCCTCTCGGCCCCTGGTGCAACAACCCGAGGCTCCCCCGATCCACATTGCCTTCTCTCGACCAGAGATTCCCCATCCCTCAGGGGAGGCCCTGGAGGAGGAGGGGGCCAAAGCCCCAGTCTTGGCCAATGGGCACATCCCCTATAGCCGAACTCTGAGCCATATCAGTGAGGCCAGTGTAGATGCTGTGCTTGAGGCTACCAATGAATCTGTGGCCTCAGAGAGCCGTGCCCAGGGCCTCACCTCCTCTGTACACCTAGACTCTACTGACACTACCCCACTCCCTGTTCTAGAGGCACATCCAGACCCCTTCAACCCAAGCCCCACCCTCTCTTGTTTAGACCAGACCACTTTGCATGAAGGCCTTACCCCCTCACCCTCAGAACCAGTCACTTTAAATCTGAAGTCCTTTCCCTCACATCCAAGACCGTCTGAGTCTGATATTACCCAACTAGGCCCCCTCCCCCCCACCTCAGATCCCATATCCTCTTGCCCAGACTCCATCGCCTCCCATCCAGCGCCTGAAACCTTGCACTCTGACCATGGCTGTATCCACCCAGACTCTTCCCCCTCACACTCAGACCTTGCCCCTGTGTTCCCACTTACCAGTCCTGAACTCACAGACCATGTTTCTCCTTCCCCAGCCCCAACCCTACTGCACTCAGACCCTGCATCCCGACACTCAGATTCATCTTACCCCCATTCATACCCTGCCTCGTCACATACAGATCCCACCCCCACACAGTCACCCCTCACAGACCATGCCCCCAGTCCCCCTGTGGTGCCCCAGGTTCCAGTCCAGGAGGCAGCTGGTATTTCCCTAACAGAGGCCAAAGTTCTTCCTCAGAAGGGGCTGCTGGAGCAGGGAGCAGGGCTGAGGGACACAGGGCTGGAAGAAGCCCTGGGGGCCTTGAGCTCTGCCCTGGATGACTATCGGGGCCAATTTCCAGAGCTGCAAGGACTGGAGCAGGAGGTGACTCGGCTCGAGAGTCTACTTATGCAGAGACAAGGCATGTCCCGAAGCCGGGCATCCAGCCTCAGCCTCACTGTGGAGCATGCACTGGAGAGCTTTAGCTTCCTCAATGATGATGAAGATGAGGATGACGGGCCTGAGGACAGGTGAAGAAGGGGCTGGGGTACAGGTGAGGGAGGGACCGTTGAGAGATTAAGAACACAGAAGAGTGAAAGTGGAGGGGGAGCTGAGGGGAGGACAGTTAGGATTTACTGGGAAGGGGACCTGTATCTAACCTTTCTCTCCAGCTCTCCCCTGTGTTTCCATTTTCCCCAGTTACCCCCTTCCTGGGTCGTTGCCCCCACCCCGCCTTCAAGTGTCTTCCTTAGAGTCTTCAGGACACCCCCTCCCAGTACCCCTGATCTGTCTACCTCTAGCTCTGCCTCTCTCCCTCAGTCTCATTTTGGACCTGCTCTCTGGACATGCCCCCCCATCATCTCCTCACTCACCCATGGCTGGAAACTGGCCTCATCATCTTCATCTCTCCCCTTCGGCCCTCACTCCCTCAGCCCAACATAGGCGGAGGCTTTGGTCCCTTGATGAACTCCCTCGGAGCAGCTTTCGTACTGTCCACCTCACTGACCGAGTTTTGCCCCTTCTCCCCTCGCCGCTGCTCCAGCCCAAAGCCTCAGCACCTCTGTCCTCCCGCCAGAGCCCCTGTCCCTTCCTTTGGTGCTCTGTCAGACGGTCACTTCACCGACGGCCTTCCCATTGCCAACCTGGTGACCTCTCTTCCCAATACTCAAAATGCCAGCACCAGTCTTCTGCCCCTCTGACTACATTTACTCTCTGCCCTCCCTGAGATCCATTTCATCCCCACTCAAATACACTCCCATCACTACAACCCATGGTGGCCCCTGCTCAGAGGTCTCCACAATCCAGGAAGTCCAGTTATCGCAGCCATGTATGTGTATGTGAAGTCATCCTCCATGTGATGATTCATGTGCTGTTCTACACCAGCTATCCATCCTGTCTGTATCTAGAGGTGGGAGAGGGGGCTGAGGATGGGTATACATCTCAATAAATGTACACTGGGCACAGTGAGGGTGATACTCTGCCTCTGATGATCTGGATAGCAGGTGCTCACCACTCAGGTCTCTCCTGCAGGATGTTGAGCAGCCCAGGGCCTGGGTCTGAGGCTGGGGTTAGTGGTGTGTCTGAGGATGCCCTGGACTCCTCTACGCCTGGCCCCCTCAGCACTGGCTGCCCTGCCCTGGACACAGCCCTGGTCTTGCACCTACAGCACTGTGGCCACCTCTTGCTGGTGAGGCCAGCAGCCCTCCCCTCCAATACATTAACCCATTACTGACCCCAAGAGTCTAGCACACTAGGACCTTTAGCCTCCATTACCCCGTTGCACGCTTGTAATCTCCACCAGCATTTTTTTTAACCTCAGATGGACACACCTGCCCTTTGCATGCTGGGAGTTGCAGTCCCTGGGCTCCACACCATATTTCTAGGTCCCCTGTGCTACCCACCACCCCCAACCCTGAATAGGGGATGAGTAGAACTCCTACTCCCTGTGTCTGAGGTGGCCCTGGCTGGTCTCTGCTCTTCAGAAACTAGGCACCTTTGGGCCCCTACGATGCCAGGAAGCATCAGCATTGGAGCGGCTGCTTCGGGAGGCCCTGGTGCTGGAGGTGGTGTGCCAACTTTGTGGGGGGCAAGTGGGCAAGGCCACCTCTGCTCAGGAAGGTAAAGGTACCCACTCCCTTCCACCCTGGAGCAAAATCGGGAAATCTGCGTCTGTCCTTTCTCCAGAATTCTGCCTTTTGTCGTCGTGTCCCCCCCCCCCGAGACCTGCCCCCACTCTCCACCCCGCCCAACAAGCCCTCCATTCACCTACCCGCTGCAGCCCCTCATCGGCCCTCCTGGTCCCCCTGACTGTTTCCCCCCACGCCCCTCCCCCACCAGTGCTGCAGTTCTCTGTTCCCCGGCCGGGCTTCCTGCCTTTCTGGGACCTGTGCACTGAGGGAGGCAGCCTCTTGGTTTGTCCTGTGGAGCGCATCCTCTTCACCTTCTGCAGCCAGTATGGAGCCCGCCTCTCGCTGCGCCAGCCAGGCCTGGCCGAGACTGGTGAGTGACAGTCCCCGTCTTCCCCACGTGCTAGCCAAGCCCTCGCACAACCCCCTCACAAACATGTCCTTAGCGCGTACCGTGTTCAAGGCGGAGAATTAACTGAGACGCACCCAAAATGTATGGCATACTGGCCAAGCCCAGCGAGTGCGTGTGGAGATGGCGCGGCATGTCAGGCACAAAGATCTGGAGCTGGAAGGAACAGAAGAGGCCACCTTTGCCAGCTTCCCCATTTTATTAGAGGACCAAACTCAGCCCCTGAGAGGCCTTGTGACTCGCCGAGTTCACCCAGGGAGTGGGTGACAGATCTGGTACTCGAGCCCAGATCTCCTACGAATCACAGAGTTCTTGCTCTGATACCAGGCAGCTTACGTGTTTGGATGAGGGGTAGAGAGAGTCAGGGCTGCTGCAGGTCAGACAAGGGATCGAGCACTCCGGGATGGGTCTGGTCAGGAATCTGCAGCGTGGCTGGGGCGGCTGAGGCACACCTTCACCTGGGCACATCCAGAGGCTTCCATGACATCCCTCTTGAGTACCCCGTCCTTCCTGTCTCCTTTCCCAGTGTGTGTTAAGCTCCTGGAGGACGCTATGGGACAGAGGCTGCCCCGGAGGCCTCGGCCGGGGCCTGGGGAGCAGCTCACAATCTTCCAGTTCTGGAGCCACATCGAGGCCTTGAATAGCCCCTCCATGGAGGCTTATGTCACAGAGACAGCCGAAGAGGTGTTGATCGTGAAGAACCTGAATTCAGACGACCAGGCCGTGGTGCTCCGGGCACTGAGACTGGCCCCTGAGGGGCGGCTCCACCGGGATGGGCTCCGGGCCCTTAGCTCCCTTCTCATCCACGGTAACAGCAAAGTGATGGCTGCTGTCAGCACCCAGCTTCGAAGTTTAGCCCTGGGCTCGGACTTCCGGAAGAAGGCCCTGCTTTGTTTTTTGGAACAGCTAGAGGATGAGGATATGCAGACACGGGTGGCAGGGTGCGTGGCTCTGGGCTGTCTCAAGGCTCCAGAGGGCATTGAACCACTGGTGTATCTGTGCCAAACAGACAAGGAGGCTGTGAGGGAAGCTGCCCGGCAGAGCCTCCTGCAGTGTGGGGAGGATGGGCAGTCAGCTCACCGACGGCTGGAAGAGTCTCTGGAAGCTCTGCCCCGGATCTTCGGGCCAGGAAGCATGGCCAGCACCGCGTTCTGAGCTCCCTATCTGCTCTCTGTCCCAGGAACACAGGGGACTCGGGAGTCACTGCCTATTCCCTCAAGCCCCTCCCCCTACTACCACCTTTCAGGGCTGGCCCTGCCCTGGGAGCCCCCAACCTGATGGGCCCAACTGACATTGGCAGGGGTAGGCTGGGTGTCAGGACCCAGGCTCCAGGGTTCCCTCCCATCCTACAGATTCCTATAAATATAAATCTCTTTAATATATTCTTCTGCTACCCCTAGCAGAGACCGCCACCTGCAGTCAAGTGCCTCCCCAGCCTCCATCAGGCTTCCCTCACCACACGGTGTCTGGGATCCCTTCTAATCCCTGGTCACCCCGCCTCTCCCCTTCATGTCTGATTTTGTATTTTATTTAGAGTTTTGCAGAAAATAAAAGGCAAAATCTCTTTCCTATGCTGGGCAGCACCACCATTGTGGAGCACCCTGTCTTGCCTATCCTCCAACTCTAAACTCATCAAGGAAGAGAGGAGACTTGGAGAATCTGTGTCTTTTACTTCACCCATACTATTGTCACTGTTTCCCTGGGGACCCTGGTGTGTATTTCTGCCTATGCACCAAAAAGTGTGTTGATATAAGCAGAAGAATGTGTACCCAATAAGAGCTCACAGTAACATTTACAAAGCATCACACACACACACACACAAAACCTTATAGGTTAGTTTAAATTGGTCTTTTGAAGACCAAATACCCTCTTTTTCATCTTCTTAGAGCTTCAGATTAGATATTAAAACACTTCCTTATATTATTCATGCAGTACGACCAGAATGAAACACTGAATGTTCATTAGTATTACATGATAGTATAGTTATACTCAACAGTACTTTAAGTAAAAATCTTATGTCACAAAATATTTTCTTCAGGACTCCTAACTAGGTTTCTATGTTTTTATTACACATCACATTTGTTTAAACTTCTTTTGAGCTCCAGATTTTAAAAATATATTTATATACATGTTGTATATGTTTTTAATAATTCAATGAGTGGTCAGTCCTCATAATTATCAAAAGCTTATTTATACTGGGTCTAACAGCTAAACCAGCCCTTTGTTCTGTGGACAAGAAATTATGTTTAAAAAACATTTTTCTTGAAATGCTTGATTTTGATTTTATTCATTTCTCATAATATTTAAAAATAGGTTTGTCTGATACCCAGCAGTATAATTGAGTGGAAAATTTTAAGGTTCTGGCTATTTTCATTCAGAACATTTTAAATTTAGAGGTTATTTAACAAATCTGTGAATGCTTGGTAGTTAAAATAACTACAATGCATTATCATAAACTCTGTAAAAACAATGGCTTGCATGCATGCAGTTTAAAAAAAAAAGGACAGACAAGTATAAACACACTGCTGACTCTCTCTCCAGCTCATCTAATGCGTGTTTCCACAAGGGTTGTCTGAGATCTGGCAGCCCATGTTTTGGCCTGTCACCTGGACTTTGAACAGAGTTCCGTCTGCACACATACACAGCTTGTAGCGTACCCAGTCACCATTATTTAACTGATCTCCAAAGGAAGAACTTGGTAGGCGAGAGGTGCTGACCATCACAGATCCATTTAGGATGATGCTGTGTTCAGCTTTTAATTCCATATTACCACCCACGTGAAATTCAATGGTTTTGCCCATAATGAATACACCTTCATTTCCACGGACAATGGCACGTCCATCAACTTTTATATTTAAATCACTTGTAGCATTACTGGTAATCCTTTCAGTAGATGCTTTTTGTACATTCAAGCTCTTTACTCCACTTGGCAAATGAAATTCATGAGTTTCATAGTCTGTACTGAATAAAATATTTTGAGTCCTTGGGTCAAAAAACTGCATGCCAATGTCACTTGTAATAGAAGTTTTGTTCTTTTCTACACTGAGTTTCGTTGTTCCTTGCTGAAAAACAATTGGTTGGTTATTGCCAGTGATAACCAAATTTTCATTACGTCTGCCTCCTACTGTGCTCTTATAAAGAGGATGTATAACTCCCATATCAGATACTTGTTTAAATCGCAGCAAACCACTCTCATGGAACTCCATACTATCACAGCCATTGGGTCCAATACGGATCACAGCCCAGATAACAAGCGTTATAATTAAATTGATGACAGCCAAGATAAAAAGGAGAACAATCACACAGATGGCTAAATTGCCCTTTCTCCCTCTGAGGCCAGTTTTATGCAGCCGATCTTCATCAATTGGAATATAGCCAGCTTTAAAGTTACTGTTATGCTCTTTATTAACATTTCTTCGCTCAACAGCCTTCTCCCGCATAGACTTCTTTACAGGACCATTAGAACTTTGCTGCTCCGCTGCTGCAGCAGTCGCCGCCGTCGCCGCCATGTTCCCGCTCCCG

>UoN.Pci.T.25720_UoN.Pci.T.25720.5

GGGAGCCCGAGCAGAGCCCAGCCAGGCGGGACCGGTGCACCAGGGCTGATTAGGCCCGAGCAGGTCAGCTTCACGGGGCCGGTTGAGGTCCGGTGTAGTGGAGCCACCCCCACCCACCATGAACTCGAGAAAGAGAGGGAGCCCATCTCGGACTCACTCGATGATGTCGCTGTCTGTTCGGCCCCAACGTCGCATTCTCAGCGCCAGGATCAGTAGGAGCCAGTCCTTCGCAGGGGTCTTGAGTGGCCAAGACCGGGGGGGCAGGAGCCTAACAGCCTTCAGCCCCCCTGTGGCTCCCCGAAAAACACCTGCCATCGTCCGAGTCTCCAGAATGTTTTCCATGGCACACCCACCTCCAAAGGTGCCTCAGCCAGAGCGGCTGGACCAGGTGTATGAAGCCCTGAAGAGGGGTCTAACGGCCTATCTGGAGGTGCATCAGTTAGAACAGGAGAAACTCCAAGTTCAAATCAGAGAATCCAAGAGAAACTCTAGGCTGGGCTTTCTCTATGAGTTGGACAAGCAAGTGAAATCTATCGAGCGTTTCCTCCGGCGCCTAGAGTTCCATGTCAGCAAGATTGATGAGCTGTATGACGGATATTGCATCCAGCGGCGGTTACGGGATGGAGCACACAACATGGTTCGGGCTTATACTTCAGGGACTCCTGGGAGTCGGGAGGCCAGGGACAGCCTGGCTGAGGCTACTCGTGGGCACCGGGAATACACAGAGAGCATGTGTTTGCTGGAAAGTGAACTTGAAGGACAATTAGGTGAATTCCACCTCAAAATGAAAGGGCTAGCTGGTTTTGCCCGGCTGTGTGTTGGGGACCAATACGAGATCTATATGAAATATGGGCGTCAGCGCTGGAAGCTACGTGGACGAATCGAAAGCAGTGGGAAGCAAGTCTGGGACAGCGAGGACATGGTCTTCCTGCCACTGCTCACAGAGTTCCTCTCCATCAAGGTCACAGAACTAAAGAGCTTGGCGAACCATGTGGTTGTGGGCAGTGTCTCTTGTGAGACCAAAGACCTGTTTGCTGCTCTGCCTCAGGTTGTCGCTGTGGATATCAATGACCTGGGCACTATCAAACTCAGCCTTGAGGTCACTTGGAGTCCCTTCGACAAGGATGACCAACCTTCAGCGGCGTCCACGGTCACCAAGACCTCTACTGTCAGCAAACGCTTTTCCACCTATAGCCAGAGTCCTCCAGATACCCCTTCACTGCGGGAACAGGCTTTCTATAACATGCTCCGTCGTCAGGAGGAGCTAGAGAACGGAACGGCTTGGTCTCTGTCCTCAGAATCTTCAGATGACTCCTCCAGCCCCCAGCTGTCAGGGGCTGCCCGCCTCACCCCACCCTCTCGGCCCCTGGTGCAACAACCCGAGGCTCCCCCGATCCACATTGCCTTCTCTCGACCAGAGATTCCCCATCCCTCAGGGGAGGCCCTGGAGGAGGAGGGGGCCAAAGCCCCAGTCTTGGCCAATGGGCACATCCCCTATAGCCGAACTCTGAGCCATATCAGTGAGGCCAGTGTAGATGCTGTGCTTGAGGCTACCAATGAATCTGTGGCCTCAGAGAGCCGTGCCCAGGGCCTCACCTCCTCTGTACACCTAGACTCTACTGACACTACCCCACTCCCTGTTCTAGAGGCACATCCAGACCCCTTCAACCCAAGCCCCACCCTCTCTTGTTTAGACCAGACCACTTTGCATGAAGGCCTTACCCCCTCACCCTCAGAACCAGTCACTTTAAATCTGAAGTCCTTTCCCTCACATCCAAGACCGTCTGAGTCTGATATTACCCAACTAGGCCCCCTCCCCCCCACCTCAGATCCCATATCCTCTTGCCCAGACTCCATCGCCTCCCATCCAGCGCCTGAAACCTTGCACTCTGACCATGGCTGTATCCACCCAGACTCTTCCCCCTCACACTCAGACCTTGCCCCTGTGTTCCCACTTACCAGTCCTGAACTCACAGACCATGTTTCTCCTTCCCCAGCCCCAACCCTACTGCACTCAGACCCTGCATCCCGACACTCAGATTCATCTTACCCCCATTCATACCCTGCCTCGTCACATACAGATCCCACCCCCACACAGTCACCCCTCACAGACCATGCCCCCAGTCCCCCTGTGGTGCCCCAGGTTCCAGTCCAGGAGGCAGCTGGTATTTCCCTAACAGAGGCCAAAGTTCTTCCTCAGAAGGGGCTGCTGGAGCAGGGAGCAGGGCTGAGGGACACAGGGCTGGAAGAAGCCCTGGGGGCCTTGAGCTCTGCCCTGGATGACTATCGGGGCCAATTTCCAGAGCTGCAAGGACTGGAGCAGGAGGTGACTCGGCTCGAGAGTCTACTTATGCAGAGACAAGGCATGTCCCGAAGCCGGGCATCCAGCCTCAGCCTCACTGTGGAGCATGCACTGGAGAGCTTTAGCTTCCTCAATGATGATGAAGATGAGGATGACGGGCCTGAGGACAGGTGAAGAAGGGGCTGGGGTACAGGTGAGGGAGGGACCGTTGAGAGATTAAGAACACAGAAGAGTGAAAGTGGAGGGGGAGCTGAGGGGAGGACAGTTAGGATTTACTGGGAAGGGGACCTGTATCTAACCTTTCTCTCCAGCTCTCCCCTGTGTTTCCATTTTCCCCAGTTACCCCCTTCCTGGGTCGTTGCCCCCACCCCGCCTTCAAGTGTCTTCCTTAGAGTCTTCAGGACACCCCCTCCCAGTACCCCTGATCTGTCTACCTCTAGCTCTGCCTCTCTCCCTCAGTCTCATTTTGGACCTGCTCTCTGGACATGCCCCCCCATCATCTCCTCACTCACCCATGGCTGGAAACTGGCCTCATCATCTTCATCTCTCCCCTTCGGCCCTCACTCCCTCAGCCCAACATAGGCGGAGGCTTTGGTCCCTTGATGAACTCCCTCGGAGCAGCTTTCGTACTGTCCACCTCACTGACCGAGTTTTGCCCCTTCTCCCCTCGCCGCTGCTCCAGCCCAAAGCCTCAGCACCTCTGTCCTCCCGCCAGAGCCCCTGTCCCTTCCTTTGGTGCTCTGTCAGACGGTCACTTCACCGACGGCCTTCCCATTGCCAACCTGGTGACCTCTCTTCCCAATACTCAAAATGCCAGCACCAGTCTTCTGCCCCTCTGACTACATTTACTCTCTGCCCTCCCTGAGATCCATTTCATCCCCACTCAAATACACTCCCATCACTACAACCCATGGTGGCCCCTGCTCAGAGGTCTCCACAATCCAGGAAGTCCAGTTATCGCAGCCATGTATGTGTATGTGAAGTCATCCTCCATGTGATGATTCATGTGCTGTTCTACACCAGCTATCCATCCTGTCTGTATCTAGAGGTGGGAGAGGGGGCTGAGGATGGGTATACATCTCAATAAATGTACACTGGGCACAGTGAGGGTGATACTCTGCCTCTGATGATCTGGATAGCAGGTGCTCACCACTCAGGTCTCTCCTGCAGGATGTTGAGCAGCCCAGGGCCTGGGTCTGAGGCTGGGGTTAGTGGTGTGTCTGAGGATGCCCTGGACTCCTCTACGCCTGGCCCCCTCAGCACTGGCTGCCCTGCCCTGGACACAGCCCTGGTCTTGCACCTACAGCACTGTGGCCACCTCTTGCTGGTGAGGCCAGCAGCCCTCCCCTCCAATACATTAACCCATTACTGACCCCAAGAGTCTAGCACACTAGGACCTTTAGCCTCCATTACCCCGTTGCACGCTTGTAATCTCCACCAGCATTTTTTTTAACCTCAGATGGACACACCTGCCCTTTGCATGCTGGGAGTTGCAGTCCCTGGGCTCCACACCATATTTCTAGGTCCCCTGTGCTACCCACCACCCCCAACCCTGAATAGGGGATGAGTAGAACTCCTACTCCCTGTGTCTGAGGTGGCCCTGGCTGGTCTCTGCTCTTCAGAAACTAGGCACCTTTGGGCCCCTACGATGCCAGGAAGCATCAGCATTGGAGCGGCTGCTTCGGGAGGCCCTGGTGCTGGAGGTGGTGTGCCAACTTTGTGGGGGGCAAGTGGGCAAGGCCACCTCTGCTCAGGAAGGTAAAGGTACCCACTCCCTTCCACCCTGGAGCAAAATCGGGAAATCTGCGTCTGTCCTTTCTCCAGAATTCTGCCTTTTGTCGTCGTGTCCCCCCCCCCCGAGACCTGCCCCCACTCTCCACCCCGCCCAACAAGCCCTCCATTCACCTACCCGCTGCAGCCCCTCATCGGCCCTCCTGGTCCCCCTGACTGTTTCCCCCCACGCCCCTCCCCCACCAGTGCTGCAGTTCTCTGTTCCCCGGCCGGGCTTCCTGCCTTTCTGGGACCTGTGCACTGAGGGAGGCAGCCTCTTGGTTTGTCCTGTGGAGCGCATCCTCTTCACCTTCTGCAGCCAGTATGGAGCCCGCCTCTCGCTGCGCCAGCCAGGCCTGGCCGAGACTGGTGAGTGACAGTCCCCGTCTTCCCCACGTGCTAGCCAAGCCCTCGCACAACCCCCTCACAAACATGTCCTTAGCGCGTACCGTGTTCAAGGCGGAGAATTAACTGAGACGCACCCAAAATGTATGGCATACTGGCCAAGCCCAGCGAGTGCGTGTGGAGATGGCGCGGCATGTCAGGCACAAAGATCTGGAGCTGGAAGGAACAGAAGAGGCCACCTTTGCCAGCTTCCCCATTTTATTAGAGGACCAAACTCAGCCCCTGAGAGGCCTTGTGACTCGCCGAGTTCACCCAGGGAGTGGGTGACAGATCTGGTACTCGAGCCCAGATCTCCTACGAATCACAGAGTTCTTGCTCTGATACCAGGCAGCTTACGTGTTTGGATGAGGGGTAGAGAGAGTCAGGGCTGCTGCAGGTCAGACAAGGGATCGAGCACTCCGGGATGGGTCTGGTCAGGAATCTGCAGCGTGGCTGGGGCGGCTGAGGCACACCTTCACCTGGGCACATCCAGAGGCTTCCATGACATCCCTCTTGAGTACCCCGTCCTTCCTGTCTCCTTTCCCAGTGTGTGTTAAGCTCCTGGAGGACGCTATGGGACAGAGGCTGCCCCGGAGGCCTCGGCCGGGGCCTGGGGAGCAGCTCACAATCTTCCAGTTCTGGAGCCACATCGAGGCCTTGAATAGCCCCTCCATGGAGGCTTATGTCACAGAGACAGCCGAAGAGGTGTTGATCGTGAAGAACCTGAATTCAGACGACCAGGCCGTGGTGCTCCGGGCACTGAGACTGGCCCCTGAGGGGCGGCTCCACCGGGATGGGCTCCGGGCCCTTAGCTCCCTTCTCATCCACGGTAACAGCAAAGTGATGGCTGCTGTCAGCACCCAGCTTCGAAGTTTAGCCCTGGGCTCGGACTTCCGGAAGAAGGCCCTGCTTTGTTTTTTGGAACAGCTAGAGGATGAGGATATGCAGACACGGGTGGCAGGGTGCGTGGCTCTGGGCTGTCTCAAGGCTCCAGAGGGCATTGAACCACTGGTGTATCTGTGCCAAACAGACAAGGAGGCTGTGAGGGAAGCTGCCCGGCAGAGCCTCCTGCAGTGTGGGGAGGATGGGCAGTCAGCTCACCGACGGCTGGAAGAGTCTCTGGAAGCTCTGCCCCGGATCTTCGGGCCAGGAAGCATGGCCAGCACCGCGTTCTGAGCTCCCTATCTGCTCTCTGTCCCAGGAACACAGGGGACTCGGGAGTCACTGCCTATTCCCTCAAGCCCCTCCCCCTACTACCACCTTTCAGGGCTGGCCCTGCCCTGGGAGCCCCCAACCTGATGGGCCCAACTGACATTGGCAGGGGTAGGCTGGGTGTCAGGACCCAGGCTCCAGGGTTCCCTCCCATCCTACAGATTCCTATAAATATAAATCTCTTTAATATATTCTTCTGCTACCCCTAGCAGAGACCGCCACCTGCAGTCAAGTGCCTCCCCAGCCTCCATCAGGCTTCCCTCACCACACGGTGTCTGGGATCCCTTCTAATCCCTGGTCACCCCGCCTCTCCCCTTCATGTCTGATTTTGTATTTTATTTAGAGTTTTGCAGAAAATAAAAGGCAAAATCTCTTTCCTATGCTGGGCAGCACCACCATTGTGGAGCACCCTGTCTTGCCTATCCTCCAACTCTAAACTCATCAAGGAAGAGAGGAGACTTGGAGAATCTGTGTCTTTTACTTCACCCATACTATTGTCACTGTTTCCCTGGGGACCCTGGTGTGTATTTCTGCCTATGCACCAAAAAGTGTGTTGATATAAGCAGAAGAATGTGTACCCAATAAGAGCTCACAGTAACATTTACAAAGCATCACACACACACACACACAAAACCTTATAGGTTAGTTTAAATTGGTCTTTTGAAGACCAAATACCCTCTTTTTCATCTTCTTAGAGCTTCAGATTAGATATTAAAACACTTCCTTATATTATTCATGCAGTACGACCAGAATGAAACACTGAATGTTCATTAGTATTACATGATAGTATAGTTATACTCAACAGTACTTTAAGTAAAAATCTTATGTCACAAAATATTTTCTTCAGGACTCCTAACTAGGTTTCTATGTTTTTATTACACATCACATTTGTTTAAACTTCTTTTGAGCTCCAGATTTTAAAAATATATTTATATACATGTTGTATATGTTTTTAATAATTCAATGAGTGGTCAGTCCTCATAATTATCAAAAGCTTATTTATACTGGGTCTAACAGCTAAACCAGCCCTTTGTTCTGTGGACAAGAAATTATGTTTAAAAAACATTTTTCTTGAAATGCTTGATTTTGATTTTATTCATTTCTCATAATATTTAAAAATAGGTTTGTCTGATACCCAGCAGTATAATTGAGTGGAAAATTTTAAGGTTCTGGCTATTTTCATTCAGAACATTTTAAATTTAGAGGTTATTTAACAAATCTGTGAATGCTTGGTAGTTAAAATAACTACAATGCATTATCATAAACTCTGTAAAAACAATGGCTTGCATGCATGCAGTTTAAAAAAAAAAGGACAGACAAGTATAAACACACTGCTGACTCTCTCTCCAGCTCATCTAATGCGTGTTTCCACAAGGGTTGTCTGAGATCTGGCAGCCCATGTTTTGGCCTGTCACCTGGACTTTGAACAGAGTTCCGTCTGCACACATACACAGCTTGTAGCGTACCCAGTCACCATTATTTAACTGATCTCCAAAGGAAGAACTTGGTAGGCGAGAGGTGCTGACCATCACAGATCCATTTAGGATGATGCTGTGTTCAGCTTTTAATTCCATATTACCACCCACGTGAAATTCAATGGTTTTGCCCATAATGAATACACCTTCATTTCCACGGACAATGGCACGTCCATCAACTTTTATATTTAAATCACTTGTAGCATTACTGGTAATCCTTTCAGTAGATGCTTTTTGTACATTCAAGCTCTTTACTCCACTTGGCAAATGAAATTCATGAGTTTCATAGTCTGTACTGAATAAAATATTTTGAGTCCTTGGGTCAAAAAACTGCATGCCAATGTCACTTGTAATAGAAGTTTTGTTCTTTTCTACACTGAGTTTCGTTGTTCCTTGCTGAAAAACAATTGGTTGGTTATTGCCAGTGATAACCAAATTTTCATTACGTCTGCCTCCTACTGTGCTCTTATAAAGAGGATGTATAACTCCCATATCAGATACTTGTTTAAATCGCAGCAAACCACTCTCATGGAACTCCATACTATCACAGCCATTGGGTCCAATACGGATCACAGCCCAGATAACAAGCGTTATAATTAAATTGATGACAGCCAAGATAAAAAGGAGAACAATCACACAGATGGCTAAATTGCCCTTTCTCCCTCTGAGGCCAGTTTTATGCAGCCGATCTTCATCAATTGGAATATAGCCAGCTTTAAAGTTACTGTTATGCTCTTTATTAACATTTCTTCGCTCAACAGCCTTCTCCCGCATAGACTTCTTTACAGGACCATTAGAACTTTGCTGCTCCGCTGCTGCAGCAGTCGCCGCCGTCGCCGCCATGTTCCCGCTCCCG

>UoN.Pci.T.25720_UoN.Pci.T.25720.6

GGGAGCCCGAGCAGAGCCCAGCCAGGCGGGACCGGTGCACCAGGGCTGATTAGGCCCGAGCAGGTCAGCTTCACGGGGCCGGTTGAGGTCCGGTGTAGTGGAGCCACCCCCACCCACCATGAACTCGAGAAAGAGAGGGAGCCCATCTCGGACTCACTCGATGATGTCGCTGTCTGTTCGGCCCCAACGTCGCATTCTCAGCGCCAGGATCAGTAGGAGCCAGTCCTTCGCAGGGGTCTTGAGTGGCCAAGACCGGGGGGGCAGGAGCCTAACAGCCTTCAGCCCCCCTGTGGCTCCCCGAAAAACACCTGCCATCGTCCGAGTCTCCAGAATGTTTTCCATGGCACACCCACCTCCAAAGGTGCCTCAGCCAGAGCGGCTGGACCAGGTGTATGAAGCCCTGAAGAGGGGTCTAACGGCCTATCTGGAGGTGCATCAGTTAGAACAGGAGAAACTCCAAGTTCAAATCAGAGAATCCAAGAGAAACTCTAGGCTGGGCTTTCTCTATGAGTTGGACAAGCAAGTGAAATCTATCGAGCGTTTCCTCCGGCGCCTAGAGTTCCATGTCAGCAAGATTGATGAGCTGTATGACGGATATTGCATCCAGCGGCGGTTACGGGATGGAGCACACAACATGGTTCGGGCTTATACTTCAGGGACTCCTGGGAGTCGGGAGGCCAGGGACAGCCTGGCTGAGGCTACTCGTGGGCACCGGGAATACACAGAGAGCATGTGTTTGCTGGAAAGTGAACTTGAAGGACAATTAGGTGAATTCCACCTCAAAATGAAAGGGCTAGCTGGTTTTGCCCGGCTGTGTGTTGGGGACCAATACGAGATCTATATGAAATATGGGCGTCAGCGCTGGAAGCTACGTGGACGAATCGAAAGCAGTGGGAAGCAAGTCTGGGACAGCGAGGACATGGTCTTCCTGCCACTGCTCACAGAGTTCCTCTCCATCAAGGTCACAGAACTAAAGAGCTTGGCGAACCATGTGGTTGTGGGCAGTGTCTCTTGTGAGACCAAAGACCTGTTTGCTGCTCTGCCTCAGGTTGTCGCTGTGGATATCAATGACCTGGGCACTATCAAACTCAGCCTTGAGGTCACTTGGAGTCCCTTCGACAAGGATGACCAACCTTCAGCGGCGTCCACGGTCACCAAGACCTCTACTGTCAGCAAACGCTTTTCCACCTATAGCCAGAGTCCTCCAGATACCCCTTCACTGCGGGAACAGGCTTTCTATAACATGCTCCGTCGTCAGGAGGAGCTAGAGAACGGAACGGCTTGGTCTCTGTCCTCAGAATCTTCAGATGACTCCTCCAGCCCCCAGCTGTCAGGGGCTGCCCGCCTCACCCCACCCTCTCGGCCCCTGGTGCAACAACCCGAGGCTCCCCCGATCCACATTGCCTTCTCTCGACCAGAGATTCCCCATCCCTCAGGGGAGGCCCTGGAGGAGGAGGGGGCCAAAGCCCCAGTCTTGGCCAATGGGCACATCCCCTATAGCCGAACTCTGAGCCATATCAGTGAGGCCAGTGTAGATGCTGTGCTTGAGGCTACCAATGAATCTGTGGCCTCAGAGAGCCGTGCCCAGGGCCTCACCTCCTCTGTACACCTAGACTCTACTGACACTACCCCACTCCCTGTTCTAGAGGCACATCCAGACCCCTTCAACCCAAGCCCCACCCTCTCTTGTTTAGACCAGACCACTTTGCATGAAGGCCTTACCCCCTCACCCTCAGAACCAGTCACTTTAAATCTGAAGTCCTTTCCCTCACATCCAAGACCGTCTGAGTCTGATATTACCCAACTAGGCCCCCTCCCCCCCACCTCAGATCCCATATCCTCTTGCCCAGACTCCATCGCCTCCCATCCAGCGCCTGAAACCTTGCACTCTGACCATGGCTGTATCCACCCAGACTCTTCCCCCTCACACTCAGACCTTGCCCCTGTGTTCCCACTTACCAGTCCTGAACTCACAGACCATGTTTCTCCTTCCCCAGCCCCAACCCTACTGCACTCAGACCCTGCATCCCGACACTCAGATTCATCTTACCCCCATTCATACCCTGCCTCGTCACATACAGATCCCACCCCCACACAGTCACCCCTCACAGACCATGCCCCCAGTCCCCCTGTGGTGCCCCAGGTTCCAGTCCAGGAGGCAGCTGGTATTTCCCTAACAGAGGCCAAAGTTCTTCCTCAGAAGGGGCTGCTGGAGCAGGGAGCAGGGCTGAGGGACACAGGGCTGGAAGAAGCCCTGGGGGCCTTGAGCTCTGCCCTGGATGACTATCGGGGCCAATTTCCAGAGCTGCAAGGACTGGAGCAGGAGGTGACTCGGCTCGAGAGTCTACTTATGCAGAGACAAGGCATGTCCCGAAGCCGGGCATCCAGCCTCAGCCTCACTGTGGAGCATGCACTGGAGAGCTTTAGCTTCCTCAATGATGATGAAGATGAGGATGACGGGCCTGAGGACAGGTGAAGAAGGGGCTGGGGTACAGGTGAGGGAGGGACCGTTGAGAGATTAAGAACACAGAAGAGTGAAAGTGGAGGGGGAGCTGAGGGGAGGACAGTTAGGATTTACTGGGAAGGGGACCTGTATCTAACCTTTCTCTCCAGCTCTCCCCTGTGTTTCCATTTTCCCCAGTTACCCCCTTCCTGGGTCGTTGCCCCCACCCCGCCTTCAAGTGTCTTCCTTAGAGTCTTCAGGACACCCCCTCCCAGTACCCCTGATCTGTCTACCTCTAGCTCTGCCTCTCTCCCTCAGTCTCATTTTGGACCTGCTCTCTGGACATGCCCCCCCATCATCTCCTCACTCACCCATGGCTGGAAACTGGCCTCATCATCTTCATCTCTCCCCTTCGGCCCTCACTCCCTCAGCCCAACATAGGCGGAGGCTTTGGTCCCTTGATGAACTCCCTCGGAGCAGCTTTCGTACTGTCCACCTCACTGACCGAGTTTTGCCCCTTCTCCCCTCGCCGCTGCTCCAGCCCAAAGCCTCAGCACCTCTGTCCTCCCGCCAGAGCCCCTGTCCCTTCCTTTGGTGCTCTGTCAGACGGTCACTTCACCGACGGCCTTCCCATTGCCAACCTGGTGACCTCTCTTCCCAATACTCAAAATGCCAGCACCAGTCTTCTGCCCCTCTGACTACATTTACTCTCTGCCCTCCCTGAGATCCATTTCATCCCCACTCAAATACACTCCCATCACTACAACCCATGGTGGCCCCTGCTCAGAGGTCTCCACAATCCAGGAAGTCCAGTTATCGCAGCCATGTATGTGTATGTGAAGTCATCCTCCATGTGATGATTCATGTGCTGTTCTACACCAGCTATCCATCCTGTCTGTATCTAGAGGTGGGAGAGGGGGCTGAGGATGGGTATACATCTCAATAAATGTACACTGGGCACAGTGAGGGTGATACTCTGCCTCTGATGATCTGGATAGCAGGTGCTCACCACTCAGGTCTCTCCTGCAGGATGTTGAGCAGCCCAGGGCCTGGGTCTGAGGCTGGGGTTAGTGGTGTGTCTGAGGATGCCCTGGACTCCTCTACGCCTGGCCCCCTCAGCACTGGCTGCCCTGCCCTGGACACAGCCCTGGTCTTGCACCTACAGCACTGTGGCCACCTCTTGCTGGTGAGGCCAGCAGCCCTCCCCTCCAATACATTAACCCATTACTGACCCCAAGAGTCTAGCACACTAGGACCTTTAGCCTCCATTACCCCGTTGCACGCTTGTAATCTCCACCAGCATTTTTTTTAACCTCAGATGGACACACCTGCCCTTTGCATGCTGGGAGTTGCAGTCCCTGGGCTCCACACCATATTTCTAGGTCCCCTGTGCTACCCACCACCCCCAACCCTGAATAGGGGATGAGTAGAACTCCTACTCCCTGTGTCTGAGGTGGCCCTGGCTGGTCTCTGCTCTTCAGAAACTAGGCACCTTTGGGCCCCTACGATGCCAGGAAGCATCAGCATTGGAGCGGCTGCTTCGGGAGGCCCTGGTGCTGGAGGTGGTGTGCCAACTTTGTGGGGGGCAAGTGGGCAAGGCCACCTCTGCTCAGGAAGGTAAAGGTACCCACTCCCTTCCACCCTGGAGCAAAATCGGGAAATCTGCGTCTGTCCTTTCTCCAGAATTCTGCCTTTTGTCGTCGTGTCCCCCCCCCCCGAGACCTGCCCCCACTCTCCACCCCGCCCAACAAGCCCTCCATTCACCTACCCGCTGCAGCCCCTCATCGGCCCTCCTGGTCCCCCTGACTGTTTCCCCCCACGCCCCTCCCCCACCAGTGCTGCAGTTCTCTGTTCCCCGGCCGGGCTTCCTGCCTTTCTGGGACCTGTGCACTGAGGGAGGCAGCCTCTTGGTTTGTCCTGTGGAGCGCATCCTCTTCACCTTCTGCAGCCAGTATGGAGCCCGCCTCTCGCTGCGCCAGCCAGGCCTGGCCGAGACTGGTGAGTGACAGTCCCCGTCTTCCCCACGTGCTAGCCAAGCCCTCGCACAACCCCCTCACAAACATGTCCTTAGCGCGTACCGTGTTCAAGGCGGAGAATTAACTGAGACGCACCCAAAATGTATGGCATACTGGCCAAGCCCAGCGAGTGCGTGTGGAGATGGCGCGGCATGTCAGGCACAAAGATCTGGAGCTGGAAGGAACAGAAGAGGCCACCTTTGCCAGCTTCCCCATTTTATTAGAGGACCAAACTCAGCCCCTGAGAGGCCTTGTGACTCGCCGAGTTCACCCAGGGAGTGGGTGACAGATCTGGTACTCGAGCCCAGATCTCCTACGAATCACAGAGTTCTTGCTCTGATACCAGGCAGCTTACGTGTTTGGATGAGGGGTAGAGAGAGTCAGGGCTGCTGCAGGTCAGACAAGGGATCGAGCACTCCGGGATGGGTCTGGTCAGGAATCTGCAGCGTGGCTGGGGCGGCTGAGGCACACCTTCACCTGGGCACATCCAGAGGCTTCCATGACATCCCTCTTGAGTACCCCGTCCTTCCTGTCTCCTTTCCCAGTGTGTGTTAAGCTCCTGGAGGACGCTATGGGACAGAGGCTGCCCCGGAGGCCTCGGCCGGGGCCTGGGGAGCAGCTCACAATCTTCCAGTTCTGGAGCCACATCGAGGCCTTGAATAGCCCCTCCATGGAGGCTTATGTCACAGAGACAGCCGAAGAGGTGTTGATCGTGAAGAACCTGAATTCAGACGACCAGGCCGTGGTGCTCCGGGCACTGAGACTGGCCCCTGAGGGGCGGCTCCACCGGGATGGGCTCCGGGCCCTTAGCTCCCTTCTCATCCACGGTAACAGCAAAGTGATGGCTGCTGTCAGCACCCAGCTTCGAAGTTTAGCCCTGGGCTCGGACTTCCGGAAGAAGGCCCTGCTTTGTTTTTTGGAACAGCTAGAGGATGAGGATATGCAGACACGGGTGGCAGGGTGCGTGGCTCTGGGCTGTCTCAAGGCTCCAGAGGGCATTGAACCACTGGTGTATCTGTGCCAAACAGACAAGGAGGCTGTGAGGGAAGCTGCCCGGCAGAGCCTCCTGCAGTGTGGGGAGGATGGGCAGTCAGCTCACCGACGGCTGGAAGAGTCTCTGGAAGCTCTGCCCCGGATCTTCGGGCCAGGAAGCATGGCCAGCACCGCGTTCTGAGCTCCCTATCTGCTCTCTGTCCCAGGAACACAGGGGACTCGGGAGTCACTGCCTATTCCCTCAAGCCCCTCCCCCTACTACCACCTTTCAGGGCTGGCCCTGCCCTGGGAGCCCCCAACCTGATGGGCCCAACTGACATTGGCAGGGGTAGGCTGGGTGTCAGGACCCAGGCTCCAGGGTTCCCTCCCATCCTACAGATTCCTATAAATATAAATCTCTTTAATATATTCTTCTGCTACCCCTAGCAGAGACCGCCACCTGCAGTCAAGTGCCTCCCCAGCCTCCATCAGGCTTCCCTCACCACACGGTGTCTGGGATCCCTTCTAATCCCTGGTCACCCCGCCTCTCCCCTTCATGTCTGATTTTGTATTTTATTTAGAGTTTTGCAGAAAATAAAAGGCAAAATCTCTTTCCTATGCTGGGCAGCACCACCATTGTGGAGCACCCTGTCTTGCCTATCCTCCAACTCTAAACTCATCAAGGAAGAGAGGAGACTTGGAGAATCTGTGTCTTTTACTTCACCCATACTATTGTCACTGTTTCCCTGGGGACCCTGGTGTGTATTTCTGCCTATGCACCAAAAAGTGTGTTGATATAAGCAGAAGAATGTGTACCCAATAAGAGCTCACAGTAACATTTACAAAGCATCACACACACACACACACAAAACCTTATAGGTTAGTTTAAATTGGTCTTTTGAAGACCAAATACCCTCTTTTTCATCTTCTTAGAGCTTCAGATTAGATATTAAAACACTTCCTTATATTATTCATGCAGTACGACCAGAATGAAACACTGAATGTTCATTAGTATTACATGATAGTATAGTTATACTCAACAGTACTTTAAGTAAAAATCTTATGTCACAAAATATTTTCTTCAGGACTCCTAACTAGGTTTCTATGTTTTTATTACACATCACATTTGTTTAAACTTCTTTTGAGCTCCAGATTTTAAAAATATATTTATATACATGTTGTATATGTTTTTAATAATTCAATGAGTGGTCAGTCCTCATAATTATCAAAAGCTTATTTATACTGGGTCTAACAGCTAAACCAGCCCTTTGTTCTGTGGACAAGAAATTATGTTTAAAAAACATTTTTCTTGAAATGCTTGATTTTGATTTTATTCATTTCTCATAATATTTAAAAATAGGTTTGTCTGATACCCAGCAGTATAATTGAGTGGAAAATTTTAAGGTTCTGGCTATTTTCATTCAGAACATTTTAAATTTAG

>UoN.Pci.T.25720_UoN.Pci.T.25720.7

GGGAGCCCGAGCAGAGCCCAGCCAGGCGGGACCGGTGCACCAGGGCTGATTAGGCCCGAGCAGGTCAGCTTCACGGGGCCGGTTGAGGTCCGGTGTAGTGGAGCCACCCCCACCCACCATGAACTCGAGAAAGAGAGGGAGCCCATCTCGGACTCACTCGATGATGTCGCTGTCTGTTCGGCCCCAACGTCGCATTCTCAGCGCCAGGATCAGTAGGAGCCAGTCCTTCGCAGGGGTCTTGAGTGGCCAAGACCGGGGGGGCAGGAGCCTAACAGCCTTCAGCCCCCCTGTGGCTCCCCGAAAAACACCTGCCATCGTCCGAGTCTCCAGAATGTTTTCCATGGCACACCCACCTCCAAAGGTGCCTCAGCCAGAGCGGCTGGACCAGGTGTATGAAGCCCTGAAGAGGGGTCTAACGGCCTATCTGGAGGTGCATCAGTTAGAACAGGAGAAACTCCAAGTTCAAATCAGAGAATCCAAGAGAAACTCTAGGCTGGGCTTTCTCTATGAGTTGGACAAGCAAGTGAAATCTATCGAGCGTTTCCTCCGGCGCCTAGAGTTCCATGTCAGCAAGATTGATGAGCTGTATGACGGATATTGCATCCAGCGGCGGTTACGGGATGGAGCACACAACATGGTTCGGGCTTATACTTCAGGGACTCCTGGGAGTCGGGAGGCCAGGGACAGCCTGGCTGAGGCTACTCGTGGGCACCGGGAATACACAGAGAGCATGTGTTTGCTGGAAAGTGAACTTGAAGGACAATTAGGTGAATTCCACCTCAAAATGAAAGGGCTAGCTGGTTTTGCCCGGCTGTGTGTTGGGGACCAATACGAGATCTATATGAAATATGGGCGTCAGCGCTGGAAGCTACGTGGACGAATCGAAAGCAGTGGGAAGCAAGTCTGGGACAGCGAGGACATGGTCTTCCTGCCACTGCTCACAGAGTTCCTCTCCATCAAGGTCACAGAACTAAAGAGCTTGGCGAACCATGTGGTTGTGGGCAGTGTCTCTTGTGAGACCAAAGACCTGTTTGCTGCTCTGCCTCAGGTTGTCGCTGTGGATATCAATGACCTGGGCACTATCAAACTCAGCCTTGAGGTCACTTGGAGTCCCTTCGACAAGGATGACCAACCTTCAGCGGCGTCCACGGTCACCAAGACCTCTACTGTCAGCAAACGCTTTTCCACCTATAGCCAGAGTCCTCCAGATACCCCTTCACTGCGGGAACAGGCTTTCTATAACATGCTCCGTCGTCAGGAGGAGCTAGAGAACGGAACGGCTTGGTCTCTGTCCTCAGAATCTTCAGATGACTCCTCCAGCCCCCAGCTGTCAGGGGCTGCCCGCCTCACCCCACCCTCTCGGCCCCTGGTGCAACAACCCGAGGCTCCCCCGATCCACATTGCCTTCTCTCGACCAGAGATTCCCCATCCCTCAGGGGAGGCCCTGGAGGAGGAGGGGGCCAAAGCCCCAGTCTTGGCCAATGGGCACATCCCCTATAGCCGAACTCTGAGCCATATCAGTGAGGCCAGTGTAGATGCTGTGCTTGAGGCTACCAATGAATCTGTGGCCTCAGAGAGCCGTGCCCAGGGCCTCACCTCCTCTGTACACCTAGACTCTACTGACACTACCCCACTCCCTGTTCTAGAGGCACATCCAGACCCCTTCAACCCAAGCCCCACCCTCTCTTGTTTAGACCAGACCACTTTGCATGAAGGCCTTACCCCCTCACCCTCAGAACCAGTCACTTTAAATCTGAAGTCCTTTCCCTCACATCCAAGACCGTCTGAGTCTGATATTACCCAACTAGGCCCCCTCCCCCCCACCTCAGATCCCATATCCTCTTGCCCAGACTCCATCGCCTCCCATCCAGCGCCTGAAACCTTGCACTCTGACCATGGCTGTATCCACCCAGACTCTTCCCCCTCACACTCAGACCTTGCCCCTGTGTTCCCACTTACCAGTCCTGAACTCACAGACCATGTTTCTCCTTCCCCAGCCCCAACCCTACTGCACTCAGACCCTGCATCCCGACACTCAGATTCATCTTACCCCCATTCATACCCTGCCTCGTCACATACAGATCCCACCCCCACACAGTCACCCCTCACAGACCATGCCCCCAGTCCCCCTGTGGTGCCCCAGGTTCCAGTCCAGGAGGCAGCTGGTATTTCCCTAACAGAGGCCAAAGTTCTTCCTCAGAAGGGGCTGCTGGAGCAGGGAGCAGGGCTGAGGGACACAGGGCTGGAAGAAGCCCTGGGGGCCTTGAGCTCTGCCCTGGATGACTATCGGGGCCAATTTCCAGAGCTGCAAGGACTGGAGCAGGAGGTGACTCGGCTCGAGAGTCTACTTATGCAGAGACAAGGCATGTCCCGAAGCCGGGCATCCAGCCTCAGCCTCACTGTGGAGCATGCACTGGAGAGCTTTAGCTTCCTCAATGATGATGAAGATGAGGATGACGGGCCTGAGGACAGGTGAAGAAGGGGCTGGGGTACAGGTGAGGGAGGGACCGTTGAGAGATTAAGAACACAGAAGAGTGAAAGTGGAGGGGGAGCTGAGGGGAGGACAGTTAGGATTTACTGGGAAGGGGACCTGTATCTAACCTTTCTCTCCAGCTCTCCCCTGTGTTTCCATTTTCCCCAGTTACCCCCTTCCTGGGTCGTTGCCCCCACCCCGCCTTCAAGTGTCTTCCTTAGAGTCTTCAGGACACCCCCTCCCAGTACCCCTGATCTGTCTACCTCTAGCTCTGCCTCTCTCCCTCAGTCTCATTTTGGACCTGCTCTCTGGACATGCCCCCCCATCATCTCCTCACTCACCCATGGCTGGAAACTGGCCTCATCATCTTCATCTCTCCCCTTCGGCCCTCACTCCCTCAGCCCAACATAGGCGGAGGCTTTGGTCCCTTGATGAACTCCCTCGGAGCAGCTTTCGTACTGTCCACCTCACTGACCGAGTTTTGCCCCTTCTCCCCTCGCCGCTGCTCCAGCCCAAAGCCTCAGCACCTCTGTCCTCCCGCCAGAGCCCCTGTCCCTTCCTTTGGTGCTCTGTCAGACGGTCACTTCACCGACGGCCTTCCCATTGCCAACCTGGTGACCTCTCTTCCCAATACTCAAAATGCCAGCACCAGTCTTCTGCCCCTCTGACTACATTTACTCTCTGCCCTCCCTGAGATCCATTTCATCCCCACTCAAATACACTCCCATCACTACAACCCATGGTGGCCCCTGCTCAGAGGTCTCCACAATCCAGGAAGTCCAGTTATCGCAGCCATGTATGTGTATGTGAAGTCATCCTCCATGTGATGATTCATGTGCTGTTCTACACCAGCTATCCATCCTGTCTGTATCTAGAGGTGGGAGAGGGGGCTGAGGATGGGTATACATCTCAATAAATGTACACTGGGCACAGTGAGGGTGATACTCTGCCTCTGATGATCTGGATAGCAGGTGCTCACCACTCAGGTCTCTCCTGCAGGATGTTGAGCAGCCCAGGGCCTGGGTCTGAGGCTGGGGTTAGTGGTGTGTCTGAGGATGCCCTGGACTCCTCTACGCCTGGCCCCCTCAGCACTGGCTGCCCTGCCCTGGACACAGCCCTGGTCTTGCACCTACAGCACTGTGGCCACCTCTTGCTGGTGAGGCCAGCAGCCCTCCCCTCCAATACATTAACCCATTACTGACCCCAAGAGTCTAGCACACTAGGACCTTTAGCCTCCATTACCCCGTTGCACGCTTGTAATCTCCACCAGCATTTTTTTTAACCTCAGATGGACACACCTGCCCTTTGCATGCTGGGAGTTGCAGTCCCTGGGCTCCACACCATATTTCTAGGTCCCCTGTGCTACCCACCACCCCCAACCCTGAATAGGGGATGAGTAGAACTCCTACTCCCTGTGTCTGAGGTGGCCCTGGCTGGTCTCTGCTCTTCAGAAACTAGGCACCTTTGGGCCCCTACGATGCCAGGAAGCATCAGCATTGGAGCGGCTGCTTCGGGAGGCCCTGGTGCTGGAGGTGGTGTGCCAACTTTGTGGGGGGCAAGTGGGCAAGGCCACCTCTGCTCAGGAAGGTAAAGGTACCCACTCCCTTCCACCCTGGAGCAAAATCGGGAAATCTGCGTCTGTCCTTTCTCCAGAATTCTGCCTTTTGTCGTCGTGTCCCCCCCCCCCGAGACCTGCCCCCACTCTCCACCCCGCCCAACAAGCCCTCCATTCACCTACCCGCTGCAGCCCCTCATCGGCCCTCCTGGTCCCCCTGACTGTTTCCCCCCACGCCCCTCCCCCACCAGTGCTGCAGTTCTCTGTTCCCCGGCCGGGCTTCCTGCCTTTCTGGGACCTGTGCACTGAGGGAGGCAGCCTCTTGGTTTGTCCTGTGGAGCGCATCCTCTTCACCTTCTGCAGCCAGTATGGAGCCCGCCTCTCGCTGCGCCAGCCAGGCCTGGCCGAGACTGGTGAGTGACAGTCCCCGTCTTCCCCACGTGCTAGCCAAGCCCTCGCACAACCCCCTCACAAACATGTCCTTAGCGCGTACCGTGTTCAAGGCGGAGAATTAACTGAGACGCACCCAAAATGTATGGCATACTGGCCAAGCCCAGCGAGTGCGTGTGGAGATGGCGCGGCATGTCAGGCACAAAGATCTGGAGCTGGAAGGAACAGAAGAGGCCACCTTTGCCAGCTTCCCCATTTTATTAGAGGACCAAACTCAGCCCCTGAGAGGCCTTGTGACTCGCCGAGTTCACCCAGGGAGTGGGTGACAGATCTGGTACTCGAGCCCAGATCTCCTACGAATCACAGAGTTCTTGCTCTGATACCAGGCAGCTTACGTGTTTGGATGAGGGGTAGAGAGAGTCAGGGCTGCTGCAGGTCAGACAAGGGATCGAGCACTCCGGGATGGGTCTGGTCAGGAATCTGCAGCGTGGCTGGGGCGGCTGAGGCACACCTTCACCTGGGCACATCCAGAGGCTTCCATGACATCCCTCTTGAGTACCCCGTCCTTCCTGTCTCCTTTCCCAGTGTGTGTTAAGCTCCTGGAGGACGCTATGGGACAGAGGCTGCCCCGGAGGCCTCGGCCGGGGCCTGGGGAGCAGCTCACAATCTTCCAGTTCTGGAGCCACATCGAGGCCTTGAATAGCCCCTCCATGGAGGCTTATGTCACAGAGACAGCCGAAGAGGTGTTGATCGTGAAGAACCTGAATTCAGACGACCAGGCCGTGGTGCTCCGGGCACTGAGACTGGCCCCTGAGGGGCGGCTCCACCGGGATGGGCTCCGGGCCCTTAGCTCCCTTCTCATCCACGGTAACAGCAAAGTGATGGCTGCTGTCAGCACCCAGCTTCGAAGTTTAGCCCTGGGCTCGGACTTCCGGAAGAAGGCCCTGCTTTGTTTTTTGGAACAGCTAGAGGATGAGGATATGCAGACACGGGTGGCAGGGTGCGTGGCTCTGGGCTGTCTCAAGGCTCCAGAGGGCATTGAACCACTGGTGTATCTGTGCCAAACAGACAAGGAGGCTGTGAGGGAAGCTGCCCGGCAGAGCCTCCTGCAGTGTGGGGAGGATGGGCAGTCAGCTCACCGACGGCTGGAAGAGTCTCTGGAAGCTCTGCCCCGGATCTTCGGGCCAGGAAGCATGGCCAGCACCGCGTTCTGAGCTCCCTATCTGCTCTCTGTCCCAGGAACACAGGGGACTCGGGAGTCACTGCCTATTCCCTCAAGCCCCTCCCCCTACTACCACCTTTCAGGGCTGGCCCTGCCCTGGGAGCCCCCAACCTGATGGGCCCAACTGACATTGGCAGGGGTAGGCTGGGTGTCAGGACCCAGGCTCCAGGGTTCCCTCCCATCCTACAGATTCCTATAAATATAAATCTCTTTAATATATTCTTCTGCTACCCCTAGCAGAGACCGCCACCTGCAGTCAAGTGCCTCCCCAGCCTCCATCAGGCTTCCCTCACCACACGGTGTCTGGGATCCCTTCTAATCCCTGGTCACCCCGCCTCTCCCCTTCATGTCTGATTTTGTATTTTATTTAGAGTTTTGCAGAAAATAAAAGGCAAAATCTCTTTCCTATGCTGGGCAGCACCACCATTGTGGAGCACCCTGTCTTGCCTATCCTCCAACTCTAAACTCATCAAGGAAGAGAGGAGACTTGGAGAATCTGTGTCTTTTACTTCACCCATACTATTGTCACTGTTTCCCTGGGGACCCTGGTGTGTATTTCTGCCTATGCACCAAAAAGTGTGTTGATATAAGCAGAAGAATGTGTACCCAATAAGAGCTCACAGTAACATTTACAAAGCATCACACACACACACACACAAAACCTTATAGGTTAGTTTAAATTGGTCTTTTGAAGACCAAATACCCTCTTTTTCATCTTCTTAGAGCTTCAGATTAGATATTAAAACACTTCCTTATATTATTCATGCAGTACGACCAGAATGAAACACTGAATGTTCATTAGTATTACATGATAGTATAGTTATACTCAACAGTACTTTAAGTAAAAATCTTATGTCACAAAATATTTTCTTCAGGACTCCTAACTAGGTTTCTATGTTTTTATTACACATCACATTTGTTTAAACTTCTTTTGAGCTCCAGATTTTAAAAATATATTTATATACATGTTGTATATGTTTTTAATAATTCAATGAGTGGTCAGTCCTCATAATTATCAAAAGCTTATTTATACTGGGTCTAACAGCTAAACCAGCCCTTTGTTCTGTGGACAAGAAATTATGTTTAAAAAACATTTTTCTTGAAATGCTTGATTTTGATTTTATTCATTTCTCATAATATTTAAAAATAGGTTTGTCTGATACCCAGCAGTATAATTGAGTGGAAAATTTTAAGGTTCTGGCTATTTTCATTCAGAACATTTTAAATTTAGAGGTTATTTAACAAATCTGTGAATGCTTGGTAGTTAAAATAACTACAATGCATTATCATAAACTCTGTAAAAACAATGGCTTGCATGCATGCAGTTTAAAAAAAAAAGGACAGACAAGTATAAACACACTGCTGACTCTCTCTCCAGCTCATCTAATGCGTGTTTCCACAAGGGTTGTCTGAGATCTGGCAGCCCATGTTTTGGCCTGTCACCTGGACTTTGAACAGAGTTCCGTCTGCACACATACACAGCTTGTAGCGTACCCAGTCACCATTATTTAACTGATCTCCAAAGGAAGAACTTGGTAGGCGAGAGGTGCTGACCATCACAGATCCATTTAGGATGATGCTGTGTTCAGCTTTTAATTCCATATTACCACCCACGTGAAATTCAATGGTTTTGCCCATAATGAATACACCTTCATTTCCACGGACAATGGCACGTCCATCAACTTTTATATTTAAATCACTTGTAGCATTACTGGTAATCCTTTCAGTAGATGCTTTTTGTACATTCAAGCTCTTTACTCCACTTGGCAAATGAAATTCATGAGTTTCATAGTCTGTACTGAATAAAATATTTTGAGTCCTTGGGTCAAAAAACTGCATGCCAATGTCACTTGTAATAGAAGTTTTGTTCTTTTCTACACTGAGTTTCGTTGTTCCTTGCTGAAAAACAATTGGTTGGTTATTGCCAGTGATAACCAAATTTTCATTACGTCTGCCTCCTACTGTGCTCTTATAAAGAGGATGTATAACTCCCATATCAGATACTTGTTTAAATCGCAGCAAACCACTCTCATGGAACTCCATACTATCACAGCCATTGGGTCCAATACGGATCACAGCCCAGATAACAAGCGTTATAATTAAATTGATGACAGCCAAGATAAAAAGGAGAACAATCACACAGATGGCTAAATTGCCCTTTCTCCCTCTGAGGCCAGTTTTATGCAGCCGATCTTCATCAATTGGAATATAGCCAGCTTTAAAGTTACTGTTATGCTCTTTATTAACATTTCTTCGCTCAACAGCCTTCTCCCGCATAGACTTCTTTACAGGACCATTAGAACTTTGCTGCTCCGCTGCTGCAGCAGTCGCCGCCGTCGCCGCCATGTTCCCG

>UoN.Pci.T.26298_UoN.Pci.T.26298.1

AAGAAAAGAAAAAGACAAAACATGGGATTTGATTAGGAAAGTAGCTGAGGGTCTTCCTCTCCCAGTTTTTTTGTTTGTTTGTTTGTTTTATTAAGAAAGCATGCAAAAAAAAATTGTGAGGCCAAACTCCTTCCCCACCCCTTTCCAGCCCACATTGATGCAAAGCATCATGAGTGTCACGAGGGAATCACCTAACCAAATACATGAGGGTTAAAGGGAGACAGGCAAGGAAGGGTGGATCTACACCAATTACTTGCTCCCCTGCTCCCTCTTCCATATCTTTTTCTGCCACCTGTGAGCTCCTGCTATCACACATCACTCAGTACAATGACAACTACATTTCACATTCAGCAAACAGTCCTCTTCAGCACACACTGGAATTTTGAACCAACAAAGGAGTAGTATTTTCTCCATTTCACTGGGGGCTGGGGAAGGTAGGATGGAACCGAGGTACAGAAAGAGACTTGTCCTATGCAACAGAATGGGCTGAAATTAACAGAGGGGACCTGGGAACCCAGGAGGCTTGACACCCAAATTCTTGTCAAGATTCATAGGGCCCACACCCTCTGACTGCTTGGAGTCCTGAAGTATGCCTATTTCCTGAAAGATGATTTGCTGTCAAGGTGGCACATTTGCTTGTGCAAGTAAGTCCACTCTTATAAATGCTGCATTTATCTAAGGGGAAAGGGATTTACAGGGAGAGGAGCTCTGATCAGCAGTAGGGACTCCTCAATGAAATCTGTGTAGGAGATGCTGAGTACAAAAAAGAGGAGGCCCCCTTCCTTGGATCCCTGAAATGGAGTTAAAAGACAGACATTATGCCCCACTTGTAGGAAAGGTTCTCCCTCCAGAATGAGAAACATTATGCCAGTTGCACCTTAGTTTTACTGCCTTGCCACACAATGGATTGATCCTCCACCCCATTTCCCTCTGACAAGCGCTTCTTCCCAGAGACTAGTTCTTCCTGTCCCTATACCTCAATTCATTTTATTTCTATTGTGCAACAATGCATACCCAACTGAAGTTCCTTCTAAGGAAGGAGGGAAAAAGCAGAGTCAAAACAGATGTCTTGGAAATCCTGAGAACAGGAAACAGGAATGGAAGGTCCCGTACGGGAGTTTCAGGAAGTGGAAAAGGGCCTCACCCTATTTCCACCCATTTCTACTTAAAAGTGAAAATATATGGGGTGGAGGTGTAAGCGAATTTGCTTTTCTCTCTGAAGTCCAGAATTAAACACTCAACACAGACTTTTCACTGGGTGTAAAGAGCACACTGAGCTAGTCAATATATGTTGATTTCCAATTGTTAAAGGACATAAGTTATCACTAAACCCTGTTCTCTTTTTCCACCTTCACTGTATTCCTCTCTTAAGCCTCCATGCTCTTCAAAAACCAATGGGAAAACCTCCAACCCCAAGAGATGAACATTTGCCTCTGCAACGAAAATGGCCAGGAATTCCCCACTTTTACTTCCAATGACACCTTCTCTCCTCTCAGCCCAAACTCTTGTACCTCAATGCCATTTGTTTGATTCTGCTGATATTTATGGTACTCAGGGGTCCTCTCCCTTCTGGGAAGGTCAAAGGGCTGACTGGATCAAAGGGAAGATCAGTATGAAAAACTGACCTTTGGACAAAAATATTAAATTTGTTGGGTGGGAGGTGGACATATAAAGATGGCAATTTCCATATATAAATGAGATGTGGATTTAAAGAGGTGATCTAGAATGAGGAAAAAAGGTGAAACAGAAACTCTCTCCTAGGAATCGAGTTAGGGCTGATTATAGTCACTCCTCACAAGAGCAGGAGTTGAGTTCACTAGTCAAGATCAATCCCAATTGCTGGTGTCGTGGGCTGGAGGCCACAATAGTGGTTGACTTCAATGGCAGTACAATCCAATTCCAGACATTCATAGATCACCACCTCTCTATAAGAGTCTCACAACAATCTGTATCTCACTTACCTAATCAAAACCCTACTTATCTCCTGAGATCCCGCCTTCCTATTTCAGTCACCAATCACTGCCTACCTCATCTTTCACTTGTCCTACTTCTTATTCACAGGGATTTTAAGCCCTGGCCTTACTGAGCATTGCTGGGGGGGAGAGGAGGGAGGGAAGATGACCAAGAACTATTTCAGGATGGGGGAGGAAATAGCACCACCTCGCAGACTCCTTTGGGATCAATGATGCCCGCATAGCCCTTCAGGCCCATCCACTTAATCTCTCTAGCCCTGAACTGAGTTTTGATAGGGGCAGTAACCAATCCAGAGCTAACAGGTGGGATCAGCACTGAATCTCCAAGATGTGGAAGTTTTGAGTTCCTGCTGCTCCCTGGCTGTTACAGCCTTGCCGCCCAACCCGGGGACGGCAATGGTCCGTGGCGCCAGGGACCGGAGGTCGGGGCCAGCAAAGTAATCAATAATATTATTAGTAATAACAATAATATAATAATAACAATAATAAAAGAAAGGGGAGAAAGACGGGGAAGGCAGGGTTATCTTCGGCAGCATCTTGCAGTGGCTTCTCTGGCAAAGAGAAAAAAAAAGAAAAAGAGGAAATAGATATATATCTATATCTATCTATCTATCTATGTACAGGCTTGGGGAAAGGGAACTGGACTGGGGTTATTTCTAGATTCTTTGCCAGGGTTCACCACCGCTGGCCCTGGGTTCTTCAGTCTGTGACAGACTAAGTGATCAAGTCCCAGTCGAAATCATCAGGGATCTCCTCATTGGAACCAGGAGGCGGGACAGGCGGCCGGGGGACCAGATGATGTGCTGGACGGTTATATCCTGGAGAGTCCTGGGTAGGTATAGGGTACATGGGCGATGGGCCAGAGTAGAGGTGGGGTGCTTGTGGGTGTCCATAAGAGTTCACTTGGTTTATTGTGGACTCCTGCTTGCCGCTGGTCAGGGCACAGGTGTCAGCAATTCTAGTGGGAGGTGGTGGCTGGTGGGGACCCCCTGGGTGGGGCAGCTGACCAGTCTGGGTAAGGAATTGGTTTAGGGAGTCACAGAGATTGGCAATGTGGTGCTGGTCCAAGCTCCAGTTTTTTCTTTCTGCCTGCCGGAGGCTCTCCATTAGTTCTGAAAAATGGGACTGCTCAATGCTGGACCAGTTAAGCCGATTCACCATCTTGAAGCTTTCTTTCAGGGAGTCAATGTTGGAGCACCAGTCAGAGGGAAATGCAAAGCCAGTATTGTTGAGAGAAGGCTGGGACTGGGCCTGCTGCTGGGGCGGTGGTGGAGGCTGCTGGGGCGGATGCGGGTGGGGGTGGTGCTGATGGGTATGATAACCCACGTGCTGCAGTGGGGGCGGATGCATGGCCATCACAGAGGGTGTTCCATAGCTGTCAGCCCCAGGCTGCTTCCCTCCCGGTGGGGGGCAGCCCTCTGGGCTGGGAGTATGCAGTGAAGGGGTACCCCCTACAGATGAAGGGCCCTGTGTTGGTGCTTGCTGCTGAGATTGCTGGGGTGGTTGCTGTTGTTGCTGGGGTGGCTGTGGTGGTTGCTGGTACATGTAGTAGTTGTGATTGGAAGGCTGCATATCACCCAGGAGAGTCGAGAAACCATGTGGGGAAGAGGAAGAGGAAGATTTCTCTAGCATGGACTTGTAAAGGTTTCGAAAGGACCAGCTCAGATCCTGAAAGTTTATCTCCGAGTAGGAAAATTTGAAGTCATGATTGGTATTATACAATGGAGGTGGCCCGTCAGCCCCATCTCGTCCTGGACCACCAGCTGCAACTACTGCACCATCCACGGGTCCTGAACCCTGATTGAAGCTGGTAATGGGTGTAGTGCTCTGTAGTGACATCTGAGTATTTCCTTCAGGAGACAATGAGGCTTCTCCACTCCCTGCGACACCTCCTCGTGGACTCTTACTTGCTTCCTGTTCTGGGGAGTCCTGGGATAGTTCATCATCTGGAGGGTGCCGTCTCTTACGAGAGATGTCAGGACAGGTGTCAATTGTCCAGTAAGAGCCCTTCCCAGGGTCATCCCGAGGTCTCGGCACCTTCCGGAAACATTTATTAAGAGAAAGATTGTGACGAATTGAGTTCTTCCAGCCAATGCCAGCATTCTTGTAATAAGGGAAGTTATCACAGATCCAGCGGTAAATTTCACTGAGGGTCATCTTCTTGGCTGGGGAGGAGTTGATGGCATAGGTAATAAGTGTAGCATAGCTGTATCGAGGCTTGCCATCCTGGTGAACAGCTGCTTCATCCTTGCTCAGGGTAGCATTGGGATCCGTGGGTGAACCAGGTGGGCATTTGCGGCTACCTCCAGGGGGCCCAGCTTGTGAGGCTCCCCCCAGCTTCTCAATGGTAGCCCGAAGGGTCAGCTGGGGAAGCCAGTCTATGGAGGTGAGGCTGCTCTCCAGGTCAGAAGCCATGGTGCTGCCAGGTTCTGCCTCAGAGGTTCTCCCGGCAAAGGAAGGGAACCACCTGGCACTGGAGTTTCGGGCTGAAGGGATATCTCTCTTTAGGGGCAGGTCATCCTCAGTAGCTCTTCGTGGGCTTTGCCGAGTTCCCCAAGCTGGGGTGGGGAGGCGAGTGCCCCGAGAAGAAGGAGCAGCAGCAGGAGGAGAAGGAGGTCCTACCTGCGGGAGGTGGTAGACCCCCCGCGCCTTGGCAGTAATCCTGGGGAGAAGGGGAGGAGCCTCACCCAACCTGGGGTTGTCTCCTCCCCTTTGGGAGAGTCCCCCTCCCCTCAAAACTGACTCCTTCAGGGGTGAGGGACTTCTTCTGTCGGCTGGAGAATTAAGGGGAGAGGCACTGCCCTGTTCCACTTTCTAGGTCCTAGGAAGAAGCCCGCGGACTTGGGGTGGGGATGTTGTTGCCCTGTCCTGAGTATGGGAGGAATCTGAATTCTCCCCTTCCCTACTATGTCCTCTCAGCCCCTGGTCCTGGCACTCTTCTCGGCTCTCGCCAAAGGAGAACGGGCACGAGATGGGGGAGGGCACCTTTCTCCTTTCCCCCCTTGCCCAGAAAGTCTGGTCCTAAGAAGTGAGTGTGTCTACGCAGGCTAGTGGGAAGGAGGGTGCCCTTGCTCTCCGGGGTGGGACGTGCTCCCCCGGGGCACGGCCTGGGCTCTAGGAGTCTCCGGTCCAGCGGAGGCCGGCCGGGCGCCCCGGGCGCGGCGTTTCGCCTCTCTTCGCGCTCCGCGGAGGCTGGCCGGGCAGGGAGGCCGCGCCTCCGGGCTCGGGCTCGGCGCGGGGGAGCAGCGGCGGCAGCCCGGCCGCCTCGTCCCCCGGGGCCCGCGGGACTGCCCGCGCTGCCACCCCGGACGCCACGCTACTCGGACTCCGCACGAGCGGGGGAACTGGCGAGCATACGCGCGGTCCCTGGGCTCGGCTCGGGCTCCGGCTGCGCTCCGCTGCAGGCCCCGGCTCCTCCCCG

>UoN.Pci.T.26298_UoN.Pci.T.26298.2

AAGAAAAGAAAAAGACAAAACATGGGATTTGATTAGGAAAGTAGCTGAGGGTCTTCCTCTCCCAGTTTTTTTGTTTGTTTGTTTGTTTTATTAAGAAAGCATGCAAAAAAAAATTGTGAGGCCAAACTCCTTCCCCACCCCTTTCCAGCCCACATTGATGCAAAGCATCATGAGTGTCACGAGGGAATCACCTAACCAAATACATGAGGGTTAAAGGGAGACAGGCAAGGAAGGGTGGATCTACACCAATTACTTGCTCCCCTGCTCCCTCTTCCATATCTTTTTCTGCCACCTGTGAGCTCCTGCTATCACACATCACTCAGTACAATGACAACTACATTTCACATTCAGCAAACAGTCCTCTTCAGCACACACTGGAATTTTGAACCAACAAAGGAGTAGTATTTTCTCCATTTCACTGGGGGCTGGGGAAGGTAGGATGGAACCGAGGTACAGAAAGAGACTTGTCCTATGCAACAGAATGGGCTGAAATTAACAGAGGGGACCTGGGAACCCAGGAGGCTTGACACCCAAATTCTTGTCAAGATTCATAGGGCCCACACCCTCTGACTGCTTGGAGTCCTGAAGTATGCCTATTTCCTGAAAGATGATTTGCTGTCAAGGTGGCACATTTGCTTGTGCAAGTAAGTCCACTCTTATAAATGCTGCATTTATCTAAGGGGAAAGGGATTTACAGGGAGAGGAGCTCTGATCAGCAGTAGGGACTCCTCAATGAAATCTGTGTAGGAGATGCTGAGTACAAAAAAGAGGAGGCCCCCTTCCTTGGATCCCTGAAATGGAGTTAAAAGACAGACATTATGCCCCACTTGTAGGAAAGGTTCTCCCTCCAGAATGAGAAACATTATGCCAGTTGCACCTTAGTTTTACTGCCTTGCCACACAATGGATTGATCCTCCACCCCATTTCCCTCTGACAAGCGCTTCTTCCCAGAGACTAGTTCTTCCTGTCCCTATACCTCAATTCATTTTATTTCTATTGTGCAACAATGCATACCCAACTGAAGTTCCTTCTAAGGAAGGAGGGAAAAAGCAGAGTCAAAACAGATGTCTTGGAAATCCTGAGAACAGGAAACAGGAATGGAAGGTCCCGTACGGGAGTTTCAGGAAGTGGAAAAGGGCCTCACCCTATTTCCACCCATTTCTACTTAAAAGTGAAAATATATGGGGTGGAGGTGTAAGCGAATTTGCTTTTCTCTCTGAAGTCCAGAATTAAACACTCAACACAGACTTTTCACTGGGTGTAAAGAGCACACTGAGCTAGTCAATATATGTTGATTTCCAATTGTTAAAGGACATAAGTTATCACTAAACCCTGTTCTCTTTTTCCACCTTCACTGTATTCCTCTCTTAAGCCTCCATGCTCTTCAAAAACCAATGGGAAAACCTCCAACCCCAAGAGATGAACATTTGCCTCTGCAACGAAAATGGCCAGGAATTCCCCACTTTTACTTCCAATGACACCTTCTCTCCTCTCAGCCCAAACTCTTGTACCTCAATGCCATTTGTTTGATTCTGCTGATATTTATGGTACTCAGGGGTCCTCTCCCTTCTGGGAAGGTCAAAGGGCTGACTGGATCAAAGGGAAGATCAGTATGAAAAACTGACCTTTGGACAAAAATATTAAATTTGTTGGGTGGGAGGTGGACATATAAAGATGGCAATTTCCATATATAAATGAGATGTGGATTTAAAGAGGTGATCTAGAATGAGGAAAAAAGGTGAAACAGAAACTCTCTCCTAGGAATCGAGTTAGGGCTGATTATAGTCACTCCTCACAAGAGCAGGAGTTGAGTTCACTAGTCAAGATCAATCCCAATTGCTGGTGTCGTGGGCTGGAGGCCACAATAGTGGTTGACTTCAATGGCAGTACAATCCAATTCCAGACATTCATAGATCACCACCTCTCTATAAGAGTCTCACAACAATCTGTATCTCACTTACCTAATCAAAACCCTACTTATCTCCTGAGATCCCGCCTTCCTATTTCAGTCACCAATCACTGCCTACCTCATCTTTCACTTGTCCTACTTCTTATTCACAGGGATTTTAAGCCCTGGCCTTACTGAGCATTGCTGGGGGGGAGAGGAGGGAGGGAAGATGACCAAGAACTATTTCAGGATGGGGGAGGAAATAGCACCACCTCGCAGACTCCTTTGGGATCAATGATGCCCGCATAGCCCTTCAGGCCCATCCACTTAATCTCTCTAGCCCTGAACTGAGTTTTGATAGGGGCAGTAACCAATCCAGAGCTAACAGGTGGGATCAGCACTGAATCTCCAAGATGTGGAAGTTTTGAGTTCCTGCTGCTCCCTGGCTGTTACAGCCTTGCCGCCCAACCCGGGGACGGCAATGGTCCGTGGCGCCAGGGACCGGAGGTCGGGGCCAGCAAAGTAATCAATAATATTATTAGTAATAACAATAATATAATAATAACAATAATAAAAGAAAGGGGAGAAAGACGGGGAAGGCAGGGTTATCTTCGGCAGCATCTTGCAGTGGCTTCTCTGGCAAAGAGAAAAAAAAAGAAAAAGAGGAAATAGATATATATCTATATCTATCTATCTATCTATGTACAGGCTTGGGGAAAGGGAACTGGACTGGGGTTATTTCTAGATTCTTTGCCAGGGTTCACCACCGCTGGCCCTGGGTTCTTCAGTCTGTGACAGACTAAGTGATCAAGTCCCAGTCGAAATCATCAGGGATCTCCTCATTGGAACCAGGAGGCGGGACAGGCGGCCGGGGGACCAGATGATGTGCTGGACGGTTATATCCTGGAGAGTCCTGGGTAGGTATAGGGTACATGGGCGATGGGCCAGAGTAGAGGTGGGGTGCTTGTGGGTGTCCATAAGAGTTCACTTGGTTTATTGTGGACTCCTGCTTGCCGCTGGTCAGGGCACAGGTGTCAGCAATTCTAGTGGGAGGTGGTGGCTGGTGGGGACCCCCTGGGTGGGGCAGCTGACCAGTCTGGGTAAGGAATTGGTTTAGGGAGTCACAGAGATTGGCAATGTGGTGCTGGTCCAAGCTCCAGTTTTTTCTTTCTGCCTGCCGGAGGCTCTCCATTAGTTCTGAAAAATGGGACTGCTCAATGCTGGACCAGTTAAGCCGATTCACCATCTTGAAGCTTTCTTTCAGGGAGTCAATGTTGGAGCACCAGTCAGAGGGAAATGCAAAGCCAGTATTGTTGAGAGAAGGCTGGGACTGGGCCTGCTGCTGGGGCGGTGGTGGAGGCTGCTGGGGCGGATGCGGGTGGGGGTGGTGCTGATGGGTATGATAACCCACGTGCTGCAGTGGGGGCGGATGCATGGCCATCACAGAGGGTGTTCCATAGCTGTCAGCCCCAGGCTGCTTCCCTCCCGGTGGGGGGCAGCCCTCTGGGCTGGGAGTATGCAGTGAAGGGGTACCCCCTACAGATGAAGGGCCCTGTGTTGGTGCTTGCTGCTGAGATTGCTGGGGTGGTTGCTGTTGTTGCTGGGGTGGCTGTGGTGGTTGCTGGTACATGTAGTAGTTGTGATTGGAAGGCTGCATATCACCCAGGAGAGTCGAGAAACCATGTGGGGAAGAGGAAGAGGAAGATTTCTCTAGCATGGACTTGTAAAGGTTTCGAAAGGACCAGCTCAGATCCTGAAAGTTTATCTCCGAGTAGGAAAATTTGAAGTCATGATTGGTATTATACAATGGAGGTGGCCCGTCAGCCCCATCTCGTCCTGGACCACCAGCTGCAACTACTGCACCATCCACGGGTCCTGAACCCTGATTGAAGCTGGTAATGGGTGTAGTGCTCTGTAGTGACATCTGAGTATTTCCTTCAGGAGACAATGAGGCTTCTCCACTCCCTGCGACACCTCCTCGTGGACTCTTACTTGCTTCCTGTTCTGGGGAGTCCTGGGATAGTTCATCATCTGGAGGGTGCCGTCTCTTACGAGAGATGTCAGGACAGGTGTCAATTGTCCAGTAAGAGCCCTTCCCAGGGTCATCCCGAGGTCTCGGCACCTTCCGGAAACATTTATTAAGAGAAAGATTGTGACGAATTGAGTTCTTCCAGCCAATGCCAGCATTCTTGTAATAAGGGAAGTTATCACAGATCCAGCGGTAAATTTCACTGAGGGTCATCTTCTTGGCTGGGGAGGAGTTGATGGCATAGGTAATAAGTGTAGCATAGCTGTATCGAGGCTTGCCATCCTGGTGAACAGCTGCTTCATCCTTGCTCAGGGTAGCATTGGGATCCGTGGGTGAACCAGGTGGGCATTTGCGGCTACCTCCAGGGGGCCCAGCTTGTGAGGCTCCCCCCAGCTTCTCAATGGTAGCCCGAAGGGTCAGCTGGGGAAGCCAGTCTATGGAGGTGAGGCTGCTCTCCAGGTCAGAAGCCATGGTGCTGCCAGGTTCTGCCTCAGAGGTTCTCCCGGCAAAGGAAGGGAACCACCTGGCACTGGAGTTTCGGGCTGAAGGGATATCTCTCTTTAGGGGCAGGTCATCCTCAGTAGCTCTTCGTGGGCTTTGCCGAGTTCCCCAAGCTGGGGTGGGGAGGCGAGTGCCCCGAGAAGAAGGAGCAGCAGCAGGAGGAGAAGGAGGTCCTACCTGCGGGAGGTGGTAGACCCCCCGCGCCTTGGCAGTAATCCTGGGGAGAAGGGGAGGAGCCTCACCCAACCTGGGGTTGTCTCCTCCCCTTTGGGAGAGTCCCCCTCCCCTCAAAACTGACTCCTTCAGGGGTGAGGGACTTCTTCTGTCGGCTGGAGAATTAAGGGGAGAGGCACTGCCCTGTTCCACTTTCTAGGTCCTAGGAAGAAGCCCGCGGACTTGGGGTGGGGATGTTGTTGCCCTGTCCTGAGTATGGGAGGAATCTGAATTCTCCCCTTCCCTACTATGTCCTCTCAGCCCCTGGTCCTGGCACTCTTCTCGGCTCTCGCCAAAGGAGAACGGGCACGAGATGGGGGAGGGCACCTTTCTCCTTTCCCCCCTTGCCCAGAAAGTCTGGTCCTAAGAAGTGAGTGTGTCTACGCAGGCTAGTGGGAAGGAGGGTGCCCTTGCTCTCCGGGGTGGGACGTGCTCCCCCGGGGCACGGCCTGGGCTCTAGGAGTCTCCGGTCCAGCGGAGGCCGGCCGGGCGCCCCGGGCGCGGCGTTTCGCCTCTCTTCGCGCTCCGCGGAGGCTGGCCGGGCAGGGAGGCCGCGCCTCCGGGCTCGGGCTCGGCGCGGGGGAGCAGCGGCGGCAGCCCGGCCGCCTCGTCCCCCGGGGCCCGCGGGACTGCCCGCGCTGCCACCCCGGACGCCACGCTACTCGGACTCCGCACGAGCGGGGGAACTGGCGAGCATACGCGCGGTCCCTGGGCTCGGCTCGGGCTCCGGCTGCGCTCCGCTGCAGGCCCCGGCT

>UoN.Pci.T.26298_UoN.Pci.T.26298.3

TTGTTTGTTTGTTTGTTTTATTAAGAAAGCATGCAAAAAAAAATTGTGAGGCCAAACTCCTTCCCCACCCCTTTCCAGCCCACATTGATGCAAAGCATCATGAGTGTCACGAGGGAATCACCTAACCAAATACATGAGGGTTAAAGGGAGACAGGCAAGGAAGGGTGGATCTACACCAATTACTTGCTCCCCTGCTCCCTCTTCCATATCTTTTTCTGCCACCTGTGAGCTCCTGCTATCACACATCACTCAGTACAATGACAACTACATTTCACATTCAGCAAACAGTCCTCTTCAGCACACACTGGAATTTTGAACCAACAAAGGAGTAGTATTTTCTCCATTTCACTGGGGGCTGGGGAAGGTAGGATGGAACCGAGGTACAGAAAGAGACTTGTCCTATGCAACAGAATGGGCTGAAATTAACAGAGGGGACCTGGGAACCCAGGAGGCTTGACACCCAAATTCTTGTCAAGATTCATAGGGCCCACACCCTCTGACTGCTTGGAGTCCTGAAGTATGCCTATTTCCTGAAAGATGATTTGCTGTCAAGGTGGCACATTTGCTTGTGCAAGTAAGTCCACTCTTATAAATGCTGCATTTATCTAAGGGGAAAGGGATTTACAGGGAGAGGAGCTCTGATCAGCAGTAGGGACTCCTCAATGAAATCTGTGTAGGAGATGCTGAGTACAAAAAAGAGGAGGCCCCCTTCCTTGGATCCCTGAAATGGAGTTAAAAGACAGACATTATGCCCCACTTGTAGGAAAGGTTCTCCCTCCAGAATGAGAAACATTATGCCAGTTGCACCTTAGTTTTACTGCCTTGCCACACAATGGATTGATCCTCCACCCCATTTCCCTCTGACAAGCGCTTCTTCCCAGAGACTAGTTCTTCCTGTCCCTATACCTCAATTCATTTTATTTCTATTGTGCAACAATGCATACCCAACTGAAGTTCCTTCTAAGGAAGGAGGGAAAAAGCAGAGTCAAAACAGATGTCTTGGAAATCCTGAGAACAGGAAACAGGAATGGAAGGTCCCGTACGGGAGTTTCAGGAAGTGGAAAAGGGCCTCACCCTATTTCCACCCATTTCTACTTAAAAGTGAAAATATATGGGGTGGAGGTGTAAGCGAATTTGCTTTTCTCTCTGAAGTCCAGAATTAAACACTCAACACAGACTTTTCACTGGGTGTAAAGAGCACACTGAGCTAGTCAATATATGTTGATTTCCAATTGTTAAAGGACATAAGTTATCACTAAACCCTGTTCTCTTTTTCCACCTTCACTGTATTCCTCTCTTAAGCCTCCATGCTCTTCAAAAACCAATGGGAAAACCTCCAACCCCAAGAGATGAACATTTGCCTCTGCAACGAAAATGGCCAGGAATTCCCCACTTTTACTTCCAATGACACCTTCTCTCCTCTCAGCCCAAACTCTTGTACCTCAATGCCATTTGTTTGATTCTGCTGATATTTATGGTACTCAGGGGTCCTCTCCCTTCTGGGAAGGTCAAAGGGCTGACTGGATCAAAGGGAAGATCAGTATGAAAAACTGACCTTTGGACAAAAATATTAAATTTGTTGGGTGGGAGGTGGACATATAAAGATGGCAATTTCCATATATAAATGAGATGTGGATTTAAAGAGGTGATCTAGAATGAGGAAAAAAGGTGAAACAGAAACTCTCTCCTAGGAATCGAGTTAGGGCTGATTATAGTCACTCCTCACAAGAGCAGGAGTTGAGTTCACTAGTCAAGATCAATCCCAATTGCTGGTGTCGTGGGCTGGAGGCCACAATAGTGGTTGACTTCAATGGCAGTACAATCCAATTCCAGACATTCATAGATCACCACCTCTCTATAAGAGTCTCACAACAATCTGTATCTCACTTACCTAATCAAAACCCTACTTATCTCCTGAGATCCCGCCTTCCTATTTCAGTCACCAATCACTGCCTACCTCATCTTTCACTTGTCCTACTTCTTATTCACAGGGATTTTAAGCCCTGGCCTTACTGAGCATTGCTGGGGGGGAGAGGAGGGAGGGAAGATGACCAAGAACTATTTCAGGATGGGGGAGGAAATAGCACCACCTCGCAGACTCCTTTGGGATCAATGATGCCCGCATAGCCCTTCAGGCCCATCCACTTAATCTCTCTAGCCCTGAACTGAGTTTTGATAGGGGCAGTAACCAATCCAGAGCTAACAGGTGGGATCAGCACTGAATCTCCAAGATGTGGAAGTTTTGAGTTCCTGCTGCTCCCTGGCTGTTACAGCCTTGCCGCCCAACCCGGGGACGGCAATGGTCCGTGGCGCCAGGGACCGGAGGTCGGGGCCAGCAAAGTAATCAATAATATTATTAGTAATAACAATAATATAATAATAACAATAATAAAAGAAAGGGGAGAAAGACGGGGAAGGCAGGGTTATCTTCGGCAGCATCTTGCAGTGGCTTCTCTGGCAAAGAGAAAAAAAAAGAAAAAGAGGAAATAGATATATATCTATATCTATCTATCTATCTATGTACAGGCTTGGGGAAAGGGAACTGGACTGGGGTTATTTCTAGATTCTTTGCCAGGGTTCACCACCGCTGGCCCTGGGTTCTTCAGTCTGTGACAGACTAAGTGATCAAGTCCCAGTCGAAATCATCAGGGATCTCCTCATTGGAACCAGGAGGCGGGACAGGCGGCCGGGGGACCAGATGATGTGCTGGACGGTTATATCCTGGAGAGTCCTGGGTAGGTATAGGGTACATGGGCGATGGGCCAGAGTAGAGGTGGGGTGCTTGTGGGTGTCCATAAGAGTTCACTTGGTTTATTGTGGACTCCTGCTTGCCGCTGGTCAGGGCACAGGTGTCAGCAATTCTAGTGGGAGGTGGTGGCTGGTGGGGACCCCCTGGGTGGGGCAGCTGACCAGTCTGGGTAAGGAATTGGTTTAGGGAGTCACAGAGATTGGCAATGTGGTGCTGGTCCAAGCTCCAGTTTTTTCTTTCTGCCTGCCGGAGGCTCTCCATTAGTTCTGAAAAATGGGACTGCTCAATGCTGGACCAGTTAAGCCGATTCACCATCTTGAAGCTTTCTTTCAGGGAGTCAATGTTGGAGCACCAGTCAGAGGGAAATGCAAAGCCAGTATTGTTGAGAGAAGGCTGGGACTGGGCCTGCTGCTGGGGCGGTGGTGGAGGCTGCTGGGGCGGATGCGGGTGGGGGTGGTGCTGATGGGTATGATAACCCACGTGCTGCAGTGGGGGCGGATGCATGGCCATCACAGAGGGTGTTCCATAGCTGTCAGCCCCAGGCTGCTTCCCTCCCGGTGGGGGGCAGCCCTCTGGGCTGGGAGTATGCAGTGAAGGGGTACCCCCTACAGATGAAGGGCCCTGTGTTGGTGCTTGCTGCTGAGATTGCTGGGGTGGTTGCTGTTGTTGCTGGGGTGGCTGTGGTGGTTGCTGGTACATGTAGTAGTTGTGATTGGAAGGCTGCATATCACCCAGGAGAGTCGAGAAACCATGTGGGGAAGAGGAAGAGGAAGATTTCTCTAGCATGGACTTGTAAAGGTTTCGAAAGGACCAGCTCAGATCCTGAAAGTTTATCTCCGAGTAGGAAAATTTGAAGTCATGATTGGTATTATACAATGGAGGTGGCCCGTCAGCCCCATCTCGTCCTGGACCACCAGCTGCAACTACTGCACCATCCACGGGTCCTGAACCCTGATTGAAGCTGGTAATGGGTGTAGTGCTCTGTAGTGACATCTGAGTATTTCCTTCAGGAGACAATGAGGCTTCTCCACTCCCTGCGACACCTCCTCGTGGACTCTTACTTGCTTCCTGTTCTGGGGAGTCCTGGGATAGTTCATCATCTGGAGGGTGCCGTCTCTTACGAGAGATGTCAGGACAGGTGTCAATTGTCCAGTAAGAGCCCTTCCCAGGGTCATCCCGAGGTCTCGGCACCTTCCGGAAACATTTATTAAGAGAAAGATTGTGACGAATTGAGTTCTTCCAGCCAATGCCAGCATTCTTGTAATAAGGGAAGTTATCACAGATCCAGCGGTAAATTTCACTGAGGGTCATCTTCTTGGCTGGGGAGGAGTTGATGGCATAGGTAATAAGTGTAGCATAGCTGTATCGAGGCTTGCCATCCTGGTGAACAGCTGCTTCATCCTTGCTCAGGGTAGCATTGGGATCCGTGGGTGAACCAGGTGGGCATTTGCGGCTACCTCCAGGGGGCCCAGCTTGTGAGGCTCCCCCCAGCTTCTCAATGGTAGCCCGAAGGGTCAGCTGGGGAAGCCAGTCTATGGAGGTGAGGCTGCTCTCCAGGTCAGAAGCCATGGTGCTGCCAGGTTCTGCCTCAGAGGTTCTCCCGGCAAAGGAAGGGAACCACCTGGCACTGGAGTTTCGGGCTGAAGGGATATCTCTCTTTAGGGGCAGGTCATCCTCAGTAGCTCTTCGTGGGCTTTGCCGAGTTCCCCAAGCTGGGGTGGGGAGGCGAGTGCCCCGAGAAGAAGGAGCAGCAGCAGGAGGAGAAGGAGGTCCTACCTGCGGGAGGTGGTAGACCCCCCGCGCCTTGGCAGTAATCCTGGGGAGAAGGGGAGGAGCCTCACCCAACCTGGGGTTGTCTCCTCCCCTTTGGGAGAGTCCCCCTCCCCTCAAAACTGACTCCTTCAGGGGTGAGGGACTTCTTCTGTCGGCTGGAGAATTAAGGGGAGAGGCACTGCCCTGTTCCACTTTCTAGGTCCTAGGAAGAAGCCCGCGGACTTGGGGTGGGGATGTTGTTGCCCTGTCCTGAGTATGGGAGGAATCTGAATTCTCCCCTTCCCTACTATGTCCTCTCAGCCCCTGGTCCTGGCACTCTTCTCGGCTCTCGCCAAAGGAGAACGGGCACGAGATGGGGGAGGGCACCTTTCTCCTTTCCCCCCTTGCCCAGAAAGTCTGGTCCTAAGAAGTGAGTGTGTCTACGCAGGCTAGTGGGAAGGAGGGTGCCCTTGCTCTCCGGGGTGGGACGTGCTCCCCCGGGGCACGGCCTGGGCTCTAGGAGTCTCCGGTCCAGCGGAGGCCGGCCGGGCGCCCCGGGCGCGGCGTTTCGCCTCTCTTCGCGCTCCGCGGAGGCTGGCCGGGCAGGGAGGCCGCGCCTCCGGGCTCGGGCTCGGCGCGGGGGAGCAGCGGCGGCAGCCCGGCCGCCTCGTCCCCCGGGGCCCGCGGGACTGCCCGCGCTGCCACCCCGGACGCCACGCTACTCGGACTCCGCACGAGCGGGGGAACTGGCGAGCATACGCGCGGTCCCTGGGCTCGGCTCGGGCTCCGGCTGCGCTCCGCTGCAGGCCCCGGCTC

>UoN.Pci.T.27025_UoN.Pci.T.27025.1

CCTGAGAAGGGGCGGGGCTTTGGCGCCTCCTCTGGCCCTCAGCGTTGGTTGTTTGCATTAGAGATTGAGGAGGCGGGGGAGGAGGGTTGCACGTGCGGACCGCGGGTCCCCGAGCTGCGGAGCGAGCTGATTGGACGACGGCTGGACCGCGCGCGTGCGCACTGGGGGAAAAGCGCCTCTGAAGTTTTCCTTTCCTCCGTCCGTTTTGTCGCTCTGCGCTGCATCTTCATTCTCAGTGCCCCAGGATCACGAGGACCATGTCCGGGTGGGAGTCGTATTACAAAAACGAGGGCGAGGACGAGGAAGAGGAGCAGGACGAGGGGGCGGACGCGGAAGGAAACTATAAGTATTCTGGCCGGGACAGTTTGATTTTCTTAGTGGATACCTCCAAGGCTATGTTTGAGCCCCAGGATGAAGATCAAGGAACTTCTTTTGACCTAACCATCCAGTGCATCCAGAGTGTGTACAGTAGCAAGATCATTAGCAGTGACCGAGACCTCCTGGCCGTGGTCTTCTATGGCACCGACAAAGACAAGAACTCGGTGAATTTCAGAAATGTTTATGTTCTCCATGAGCTGGATACCCCAGGTGCAAAGCGAATCCTTGAGCTTGACCAGTTTAAGGGAAAGCAGGGAGAGAAACGCTTCCAGGAGCTGATCGGCCAGGGCTCAGACTACTCCCTGAGCGATGCCCTCTGGGTCTGCGCCAACCTCTTCAGTGGGGTCCGGCTCAAGATGAGTCACAAGAGGATCATGTTGTTCACCAATGACGACAACCCCCATGGCAGTGACAGCGCCAAGGCCAGTCGGGCCAAGACCAAAGCCAGTGACCTCCGGGACACAGGTATCTTCCTCGACCTTATGAACCTGAAGAAGCCTGGGGGGTTTGACATTTCCTTATTCTACAGGGACATCATCACCCTAGCCGAGGACGAGAACCTCGGGGTTCACTTTGAGGAGTCGGGCAAGCTGGAGGACCTCCTGAGGAAGGTTCGGGCCAAGGAGACCCGGAAGCGCGCTCTGGCCAGGCTGAAGCTCAAGCTGGGCAAGGATGTAGCCCTGACTGTTGGCATTTTTAACATGGTCCAGAAGGCCTTCAAGCCTTCTCCTATCAGGCTTTACCGGGAAACAAATGAGCAGGTGAAGACCAAGACCCGAACGTTTAACGTAGACACTGGCAGCTTGCTTCTGCCCAGCGACACCAAGAGGGCCCAGGTCTATGGAGGCCGTCAGATTGTGATGGAGAAGGAGGAGACGGAGGAGCTGAAGAGATTTGATGAACCCGGCTTGGTTCTGATTGGCTTCAAGCCTCTGTTTATGCTGAAGCAGCACCATTTCCTAAGACCGTCTCTGTTTGTGTACCCAGAGGAATCCCTGGTTAATGGAAGTACAACACTGTTCGCTGCTTTACTCACCAAGTGCCTGGAGAAGGAGGTGTTGGCACTGTGCAGATACACCCCCCGCCAAAACAGCCCCCCATGTTTTGTGGCCCTGGTGCCCCAGGAGGAGGAGCTGGATGCCCAGAGAGTCCAGGTGACGCCTCCAGGCTTTCAGCTGGTCTTCCTGCCTTATGCGGATGACAAACGGAAGGTGCCCTTCACTGAGAAAGTGACCGCCAACCCGGAGCAGGTGGACAAGATGAAGGCGATTGTGCAGAGACTGCGCTTCAAGTACCGGAGCGACAGCTTTGAGAACCCTGTGCTGCAGCAGCATTTCAGAAACCTGGAGGCCTTGGCCCTGGACCTGATGGAGCCCGAGCACGTGCCAGACCTCACACTGCCCAAGGTTGAAGCCATGGATCTCAGACTGGGCAGCCTGGTGGAGGAGTTCAAGGAGCTGGTCTACCCCCCTGACTACAGCCCTGAGAAGAAGGCCGCGAAGAGGAAACCAGGTGAGGTCTGGGGGCCGCTCTGCTCCTGCCTTATCAGCCTTCGGAGGTCAGTGAAGGTTAAGGGACTGGCCGGAAACCAAGAGGGCAGGAAGTGATGGACGGAGGGTGCCCTGAGGGGTCAGTCTTGTTTGGCTCTGGTTGCCAGTCTTTGGTTTTCCCCTCACGTCGCAGAGAGAAGAGGTAAGTCTCCTCGTTCTCCTCCTCTTCAAGGGTCATCGGGGCCACATCCTGGGCCCACCCTCTTGGCGTTTCCCAAGGCCTCGTTTCTCTCCTGTGCTTCGCTGCTCTGGCACTGGCCGCCCGGGGTCATCTCCAGCTGCCTTAGGCTGTAATCGACTCTGCCCATCCGTGGGTTCAAAATACAGCTCTCTATTTCCTCTTCCTTCAGATTTCTGACACTTCTGTTGAAACGGGTTACCCTTGGCTCTCCCTTCTCTCTCCTCCCATGCAGGCTTCTGGTCAGTCTCCCTCTAATATAGCTTCTCCCCAACTGGACTCTGTTCTCTTCCTGCAGTCCTTCCCTCACAAAGCTGCCAAAGTCATCTTTAAAAGCACCAGTCTGATCATCTGCTTCCCTGGCTCAAAAAGTTTCATGGACTCCCTTTGCCTTCAGCCTCCTTCCTGTATTCCTTCATGCCCTCTGTCCCAGCCACACTGGCTGCCTCGAATCGCACTGATGCCCTCCCCTCGGCCTCCTTGGAGGCTCAGCCCGCTGACTGTGTGTGAGGCCGCCTGTTTGCCTGTTGTGCCTCTCACCCCAGAGTACTCCTTAGGCTGTTTTTCCGCATGACGGGTCTGTTTCGCCAGCCCCACACCTGGCACGTGGCTTTGTCCTGACCTCTGGTTTCTCATTGGGGTGGACCGTTGAGTAAGGAAACCCCTTTGCTTGTACAGATCATGGCTGCTCTGCAGAGGTTTCTGGGCCACTGAGGGCTCACCTAGGCAGTCTGGGACTGGACCCCAGGCCAGCACCACCCCCTGCGGGCATCCTCAGGTCTCCTGGATCGTGTGAACACCTGGCCCAGCGTGTTGGGCAGACCCTGCCTTCTGCAGCTGATGCCCCTGCTTCCTCCCACCCCTGCTCTCTCGTCTTTTGCAGTGTGACCCTCATTCCCACCTGAGGATCACCAGGGATCGGTCTGAGATGTGGCCTCCACTTTGCTGCAGAGGCGGTTTCAAGGCCAAATTTCCCTTTCATATTCCAGAAGAAAAACCTTGTGGAATTCTGATTTTTCTTGTGTGACCCAGTGGAGTATCATTTCCTCAAGGGCATAGGTGATTTTATCCCTGAACCTAGCACAGTGCCTGGCACGTAGTAGGTGCTGTTTTTGAAAGCTTTTAAAATTACATTTCTTGAAATATAATTCACTGTTTCCTCATATTTTTCAGGGAGACCAGTAGTTCTCAGGTTTGTGTCTGAGCTTGAAATAATTTCGTTTGAATTCACATGTAGCCCTTCAGTCTTCTCTCTGATGATTAATTAGCACACCGAAAGGTAGAGGTAAGGGTGAGAGCATAGAGAGAGGGGCACTCACTGGCAAGGGGAGCCTTTGTCTTTTTCTGGCTCTAGTACTTAGCACAGTGCCCGGCATGCACTAGGTGCTTAATGAATGATGTTGGCTGGCTTCTTGCTCTAAAGCCCACGAGCCTATACGTCTTAGCTGCCCAGCTGACATTCTGACCTAGTGAGAGACTCCCAGGTCAGTTTTATTAAATGCCTTGAGGCCTTTTTTCCCCCACAACTTTCCTCTGCTCAAAACCCCTCCTGTAATCCTACCCCTGTACCTCAGTCCTGCCCTTGTTGCCTTCTCCCCTCCCCCTATCTGGTCTGGGGGCTCCTCTAGAGGCTCAAGAACCCCACAGCTTCCTATTGCTCAAGTAAAAGGCAAACAGAACTCCTACAAGGGGCCCTGTTTGGTGTGGCCCCAGGCTTCCCCTGCAGCTGAGTGACCCCTGCCGGCCCTCATGACATTTCTGGCCAATCCCATCTGCTTCCCCCTTGCCTCCCCCCAAGCAAAGGTCAGCTGGCTCTTTCTCCTTGGCATCGCACACAGCAGTGGCTGTAATGGAGAGGCTCCTGGCCAGGCTGTGGCTCACAGCATCCTTTCATTCTTCACCCTTTTCATTAACAGATGGCGGTGGTCCTGAAGAGAAACAGCCCAAGCTCGAGCTGTCCGAGGAGGAGCTCAGGGCCCACGTTCAGAAGGGGAGCCTGGGCAAGCTCACAGTGCCCGTCCTCAAAGAGGCCTGCCGAGTGTATGGGCTGAAGGCCAGCAGCACCAAGAAGCAGGAGCTTTTGGACCTGCTGAAGCAGCATTTCCAGGAGGCCTGATGGGCCCAGGACCCTCGTCCTCACCCCTCGTTTACCAATAAAGAGAATTGCTTTTCTTTGGAACCAGGCAGTGTCAGGGTTCTCTCCTACTCTTCACAAGTCATTTGAAAGCATTTCCCTTTTCTCCCCTCACCGCCCCTGTAGACTCAAGGATCTGGACCCAGCCCTGGCCTTCAGAAAGAAATGGAGGCAGGGGAGCGGACAGAGCGGCCCCTTCTCTGCAGAGGAGAGAACTCCGTGGGTTAGGAGGCTCCTCACTGGGAGCACAGACCCAGTCCTTGACCTTTCTAGTCCCGGGGGGTGATCCCCCTTCCCGCCCCTTTGTCTCATACCCTGGGCTCACACCACACCTGCAAGGAACACTGACTGCGGGGCCCTCTGTGACACGGAGTGCTCTGGGCAGACCACACAAGAGGCCAGAAGTGGCTTCTTCAAGAAGGCTGCGGCTTCTTAGCACCAACAGTGACCCTGATCAGGGATTCTTAACTGGGAGTCTCTGAACTTCGAGAGACTCCAGGTTTGGGTAACGTCTGTATCAACGCCAGAAGGGGAACGGGCCCACAGCCGTGAGAGGGCAAAGCGGGGACTTCACATCCCTGTGACCAGGCACTTTGGTGGAAAACGGCCTGGTCTCTGTATACTCTACTTGGCCCAGTGTCTGGCACTTAGTAGCCCGCTCTTATCAATGCGCATTCCCTTCCTATACTACCTACAGTATAAGGCCAAGTGCCTCCACGAGGACTGCCATGGGAGCTG

>UoN.Pci.T.27025_UoN.Pci.T.27025.2

CCTGAGAAGGGGCGGGGCTTTGGCGCCTCCTCTGGCCCTCAGCGTTGGTTGTTTGCATTAGAGATTGAGGAGGCGGGGGAGGAGGGTTGCACGTGCGGACCGCGGGTCCCCGAGCTGCGGAGCGAGCTGATTGGACGACGGCTGGACCGCGCGCGTGCGCACTGGGGGAAAAGCGCCTCTGAAGTTTTCCTTTCCTCCGTCCGTTTTGTCGCTCTGCGCTGCATCTTCATTCTCAGTGCCCCAGGATCACGAGGACCATGTCCGGGTGGGAGTCGTATTACAAAAACGAGGGCGAGGACGAGGAAGAGGAGCAGGACGAGGGGGCGGACGCGGAAGGAAACTATAAGTATTCTGGCCGGGACAGTTTGATTTTCTTAGTGGATACCTCCAAGGCTATGTTTGAGCCCCAGGATGAAGATCAAGGAACTTCTTTTGACCTAACCATCCAGTGCATCCAGAGTGTGTACAGTAGCAAGATCATTAGCAGTGACCGAGACCTCCTGGCCGTGGTCTTCTATGGCACCGACAAAGACAAGAACTCGGTGAATTTCAGAAATGTTTATGTTCTCCATGAGCTGGATACCCCAGGTGCAAAGCGAATCCTTGAGCTTGACCAGTTTAAGGGAAAGCAGGGAGAGAAACGCTTCCAGGAGCTGATCGGCCAGGGCTCAGACTACTCCCTGAGCGATGCCCTCTGGGTCTGCGCCAACCTCTTCAGTGGGGTCCGGCTCAAGATGAGTCACAAGAGGATCATGTTGTTCACCAATGACGACAACCCCCATGGCAGTGACAGCGCCAAGGCCAGTCGGGCCAAGACCAAAGCCAGTGACCTCCGGGACACAGGTATCTTCCTCGACCTTATGAACCTGAAGAAGCCTGGGGGGTTTGACATTTCCTTATTCTACAGGGACATCATCACCCTAGCCGAGGACGAGAACCTCGGGGTTCACTTTGAGGAGTCGGGCAAGCTGGAGGACCTCCTGAGGAAGGTTCGGGCCAAGGAGACCCGGAAGCGCGCTCTGGCCAGGCTGAAGCTCAAGCTGGGCAAGGATGTAGCCCTGACTGTTGGCATTTTTAACATGGTCCAGAAGGCCTTCAAGCCTTCTCCTATCAGGCTTTACCGGGAAACAAATGAGCAGGTGAAGACCAAGACCCGAACGTTTAACGTAGACACTGGCAGCTTGCTTCTGCCCAGCGACACCAAGAGGGCCCAGGTCTATGGAGGCCGTCAGATTGTGATGGAGAAGGAGGAGACGGAGGAGCTGAAGAGATTTGATGAACCCGGCTTGGTTCTGATTGGCTTCAAGCCTCTGTTTATGCTGAAGCAGCACCATTTCCTAAGACCGTCTCTGTTTGTGTACCCAGAGGAATCCCTGGTTAATGGAAGTACAACACTGTTCGCTGCTTTACTCACCAAGTGCCTGGAGAAGGAGGTGTTGGCACTGTGCAGATACACCCCCCGCCAAAACAGCCCCCCATGTTTTGTGGCCCTGGTGCCCCAGGAGGAGGAGCTGGATGCCCAGAGAGTCCAGGTGACGCCTCCAGGCTTTCAGCTGGTCTTCCTGCCTTATGCGGATGACAAACGGAAGGTGCCCTTCACTGAGAAAGTGACCGCCAACCCGGAGCAGGTGGACAAGATGAAGGCGATTGTGCAGAGACTGCGCTTCAAGTACCGGAGCGACAGCTTTGAGAACCCTGTGCTGCAGCAGCATTTCAGAAACCTGGAGGCCTTGGCCCTGGACCTGATGGAGCCCGAGCACGTGCCAGACCTCACACTGCCCAAGGTTGAAGCCATGGATCTCAGACTGGGCAGCCTGGTGGAGGAGTTCAAGGAGCTGGTCTACCCCCCTGACTACAGCCCTGAGAAGAAGGCCGCGAAGAGGAAACCAGGTGAGGTCTGGGGGCCGCTCTGCTCCTGCCTTATCAGCCTTCGGAGGTCAGTGAAGGTTAAGGGACTGGCCGGAAACCAAGAGGGCAGGAAGTGATGGACGGAGGGTGCCCTGAGGGGTCAGTCTTGTTTGGCTCTGGTTGCCAGTCTTTGGTTTTCCCCTCACGTCGCAGAGAGAAGAGGTAAGTCTCCTCGTTCTCCTCCTCTTCAAGGGTCATCGGGGCCACATCCTGGGCCCACCCTCTTGGCGTTTCCCAAGGCCTCGTTTCTCTCCTGTGCTTCGCTGCTCTGGCACTGGCCGCCCGGGGTCATCTCCAGCTGCCTTAGGCTGTAATCGACTCTGCCCATCCGTGGGTTCAAAATACAGCTCTCTATTTCCTCTTCCTTCAGATTTCTGACACTTCTGTTGAAACGGGTTACCCTTGGCTCTCCCTTCTCTCTCCTCCCATGCAGGCTTCTGGTCAGTCTCCCTCTAATATAGCTTCTCCCCAACTGGACTCTGTTCTCTTCCTGCAGTCCTTCCCTCACAAAGCTGCCAAAGTCATCTTTAAAAGCACCAGTCTGATCATCTGCTTCCCTGGCTCAAAAAGTTTCATGGACTCCCTTTGCCTTCAGCCTCCTTCCTGTATTCCTTCATGCCCTCTGTCCCAGCCACACTGGCTGCCTCGAATCGCACTGATGCCCTCCCCTCGGCCTCCTTGGAGGCTCAGCCCGCTGACTGTGTGTGAGGCCGCCTGTTTGCCTGTTGTGCCTCTCACCCCAGAGTACTCCTTAGGCTGTTTTTCCGCATGACGGGTCTGTTTCGCCAGCCCCACACCTGGCACGTGGCTTTGTCCTGACCTCTGGTTTCTCATTGGGGTGGACCGTTGAGTAAGGAAACCCCTTTGCTTGTACAGATCATGGCTGCTCTGCAGAGGTTTCTGGGCCACTGAGGGCTCACCTAGGCAGTCTGGGACTGGACCCCAGGCCAGCACCACCCCCTGCGGGCATCCTCAGGTCTCCTGGATCGTGTGAACACCTGGCCCAGCGTGTTGGGCAGACCCTGCCTTCTGCAGCTGATGCCCCTGCTTCCTCCCACCCCTGCTCTCTCGTCTTTTGCAGTGTGACCCTCATTCCCACCTGAGGATCACCAGGGATCGGTCTGAGATGTGGCCTCCACTTTGCTGCAGAGGCGGTTTCAAGGCCAAATTTCCCTTTCATATTCCAGAAGAAAAACCTTGTGGAATTCTGATTTTTCTTGTGTGACCCAGTGGAGTATCATTTCCTCAAGGGCATAGGTGATTTTATCCCTGAACCTAGCACAGTGCCTGGCACGTAGTAGGTGCTGTTTTTGAAAGCTTTTAAAATTACATTTCTTGAAATATAATTCACTGTTTCCTCATATTTTTCAGGGAGACCAGTAGTTCTCAGGTTTGTGTCTGAGCTTGAAATAATTTCGTTTGAATTCACATGTAGCCCTTCAGTCTTCTCTCTGATGATTAATTAGCACACCGAAAGGTAGAGGTAAGGGTGAGAGCATAGAGAGAGGGGCACTCACTGGCAAGGGGAGCCTTTGTCTTTTTCTGGCTCTAGTACTTAGCACAGTGCCCGGCATGCACTAGGTGCTTAATGAATGATGTTGGCTGGCTTCTTGCTCTAAAGCCCACGAGCCTATACGTCTTAGCTGCCCAGCTGACATTCTGACCTAGTGAGAGACTCCCAGGTCAGTTTTATTAAATGCCTTGAGGCCTTTTTTCCCCCACAACTTTCCTCTGCTCAAAACCCCTCCTGTAATCCTACCCCTGTACCTCAGTCCTGCCCTTGTTGCCTTCTCCCCTCCCCCTATCTGGTCTGGGGGCTCCTCTAGAGGCTCAAGAACCCCACAGCTTCCTATTGCTCAAGTAAAAGGCAAACAGAACTCCTACAAGGGGCCCTGTTTGGTGTGGCCCCAGGCTTCCCCTGCAGCTGAGTGACCCCTGCCGGCCCTCATGACATTTCTGGCCAATCCCATCTGCTTCCCCCTTGCCTCCCCCCAAGCAAAGGTCAGCTGGCTCTTTCTCCTTGGCATCGCACACAGCAGTGGCTGTAATGGAGAGGCTCCTGGCCAGGCTGTGGCTCACAGCATCCTTTCATTCTTCACCCTTTTCATTAACAGATGGCGGTGGTCCTGAAGAGAAACAGCCCAAGCTCGAGCTGTCCGAGGAGGAGCTCAGGGCCCACGTTCAGAAGGGGAGCCTGGGCAAGCTCACAGTGCCCGTCCTCAAAGAGGCCTGCCGAGTGTATGGGCTGAAGGCCAGCAGCACCAAGAAGCAGGAGCTTTTGGACCTGCTGAAGCAGCATTTCCAGGAGGCCTGATGGGCCCAGGACCCTCGTCCTCACCCCTCGTTTACCAATAAAGAGAATTGCTTTTCTTTGGAACCAGGCAGTGTCAGGGTTCTCTCCTACTCTTCACAAGTCATTTGAAAGCATTTCCCTTTTCTCCCCTCACCGCCCCTGTAGACTCAAGGATCTGGACCCAGCCCTGGCCTTCAGAAAGAAATGGAGGCAGGGGAGCGGACAGAGCGGCCCCTTCTCTGCAGAGGAGAGAACTCCGTGGGTTAGGAGGCTCCTCACTGGGAGCACAGACCCAGTCCTTGACCTTTCTAGTCCCGGGGGGTGATCCCCCTTCCCGCCCCTTTGTCTCATACCCTGGGCTCACACCACACCTGCAAGGAACACTGACTGCGGGGCCCTCTGTGACACGGAGTGCTCTGGGCAGACCACACAAGAGGCCAGAAGTGGCTTCTTCAAGAAGGCTGCGGCTTCTTAGCACCAACAGTGACCCTGATCAGGGATTCTTAACTGGGAGTCTCTGAACTTCGAGAGACTCCAGGTTTGGGTAACGTCTGTATCAACGCCAGAAGGGGAACGGGCCCACAGCCGTGAGAGGGCAAAGCGGGGACTTCACATCCCTGTGACCAGGCACTTTGGTGGAAAACGGCCTGGTCTCTGTATACTCTACTTGGCCCAGTGTCTGGCACTTAGTAGCCCGCTCTTATCAATGCGCATTCCCTTCCTATACTACCTACAGTATAAGGCCAAGTGCCTCCACGAGGACTGCCATGGGAGCTGTGTCCCCTGGCTGCTGGG

>UoN.Pci.T.27025_UoN.Pci.T.27025.3

CCTGAGAAGGGGCGGGGCTTTGGCGCCTCCTCTGGCCCTCAGCGTTGGTTGTTTGCATTAGAGATTGAGGAGGCGGGGGAGGAGGGTTGCACGTGCGGACCGCGGGTCCCCGAGCTGCGGAGCGAGCTGATTGGACGACGGCTGGACCGCGCGCGTGCGCACTGGGGGAAAAGCGCCTCTGAAGTTTTCCTTTCCTCCGTCCGTTTTGTCGCTCTGCGCTGCATCTTCATTCTCAGTGCCCCAGGATCACGAGGACCATGTCCGGGTGGGAGTCGTATTACAAAAACGAGGGCGAGGACGAGGAAGAGGAGCAGGACGAGGGGGCGGACGCGGAAGGAAACTATAAGTATTCTGGCCGGGACAGTTTGATTTTCTTAGTGGATACCTCCAAGGCTATGTTTGAGCCCCAGGATGAAGATCAAGGAACTTCTTTTGACCTAACCATCCAGTGCATCCAGAGTGTGTACAGTAGCAAGATCATTAGCAGTGACCGAGACCTCCTGGCCGTGGTCTTCTATGGCACCGACAAAGACAAGAACTCGGTGAATTTCAGAAATGTTTATGTTCTCCATGAGCTGGATACCCCAGGTGCAAAGCGAATCCTTGAGCTTGACCAGTTTAAGGGAAAGCAGGGAGAGAAACGCTTCCAGGAGCTGATCGGCCAGGGCTCAGACTACTCCCTGAGCGATGCCCTCTGGGTCTGCGCCAACCTCTTCAGTGGGGTCCGGCTCAAGATGAGTCACAAGAGGATCATGTTGTTCACCAATGACGACAACCCCCATGGCAGTGACAGCGCCAAGGCCAGTCGGGCCAAGACCAAAGCCAGTGACCTCCGGGACACAGGTATCTTCCTCGACCTTATGAACCTGAAGAAGCCTGGGGGGTTTGACATTTCCTTATTCTACAGGGACATCATCACCCTAGCCGAGGACGAGAACCTCGGGGTTCACTTTGAGGAGTCGGGCAAGCTGGAGGACCTCCTGAGGAAGGTTCGGGCCAAGGAGACCCGGAAGCGCGCTCTGGCCAGGCTGAAGCTCAAGCTGGGCAAGGATGTAGCCCTGACTGTTGGCATTTTTAACATGGTCCAGAAGGCCTTCAAGCCTTCTCCTATCAGGCTTTACCGGGAAACAAATGAGCAGGTGAAGACCAAGACCCGAACGTTTAACGTAGACACTGGCAGCTTGCTTCTGCCCAGCGACACCAAGAGGGCCCAGGTCTATGGAGGCCGTCAGATTGTGATGGAGAAGGAGGAGACGGAGGAGCTGAAGAGATTTGATGAACCCGGCTTGGTTCTGATTGGCTTCAAGCCTCTGTTTATGCTGAAGCAGCACCATTTCCTAAGACCGTCTCTGTTTGTGTACCCAGAGGAATCCCTGGTTAATGGAAGTACAACACTGTTCGCTGCTTTACTCACCAAGTGCCTGGAGAAGGAGGTGTTGGCACTGTGCAGATACACCCCCCGCCAAAACAGCCCCCCATGTTTTGTGGCCCTGGTGCCCCAGGAGGAGGAGCTGGATGCCCAGAGAGTCCAGGTGACGCCTCCAGGCTTTCAGCTGGTCTTCCTGCCTTATGCGGATGACAAACGGAAGGTGCCCTTCACTGAGAAAGTGACCGCCAACCCGGAGCAGGTGGACAAGATGAAGGCGATTGTGCAGAGACTGCGCTTCAAGTACCGGAGCGACAGCTTTGAGAACCCTGTGCTGCAGCAGCATTTCAGAAACCTGGAGGCCTTGGCCCTGGACCTGATGGAGCCCGAGCACGTGCCAGACCTCACACTGCCCAAGGTTGAAGCCATGGATCTCAGACTGGGCAGCCTGGTGGAGGAGTTCAAGGAGCTGGTCTACCCCCCTGACTACAGCCCTGAGAAGAAGGCCGCGAAGAGGAAACCAGGTGAGGTCTGGGGGCCGCTCTGCTCCTGCCTTATCAGCCTTCGGAGGTCAGTGAAGGTTAAGGGACTGGCCGGAAACCAAGAGGGCAGGAAGTGATGGACGGAGGGTGCCCTGAGGGGTCAGTCTTGTTTGGCTCTGGTTGCCAGTCTTTGGTTTTCCCCTCACGTCGCAGAGAGAAGAGGTAAGTCTCCTCGTTCTCCTCCTCTTCAAGGGTCATCGGGGCCACATCCTGGGCCCACCCTCTTGGCGTTTCCCAAGGCCTCGTTTCTCTCCTGTGCTTCGCTGCTCTGGCACTGGCCGCCCGGGGTCATCTCCAGCTGCCTTAGGCTGTAATCGACTCTGCCCATCCGTGGGTTCAAAATACAGCTCTCTATTTCCTCTTCCTTCAGATTTCTGACACTTCTGTTGAAACGGGTTACCCTTGGCTCTCCCTTCTCTCTCCTCCCATGCAGGCTTCTGGTCAGTCTCCCTCTAATATAGCTTCTCCCCAACTGGACTCTGTTCTCTTCCTGCAGTCCTTCCCTCACAAAGCTGCCAAAGTCATCTTTAAAAGCACCAGTCTGATCATCTGCTTCCCTGGCTCAAAAAGTTTCATGGACTCCCTTTGCCTTCAGCCTCCTTCCTGTATTCCTTCATGCCCTCTGTCCCAGCCACACTGGCTGCCTCGAATCGCACTGATGCCCTCCCCTCGGCCTCCTTGGAGGCTCAGCCCGCTGACTGTGTGTGAGGCCGCCTGTTTGCCTGTTGTGCCTCTCACCCCAGAGTACTCCTTAGGCTGTTTTTCCGCATGACGGGTCTGTTTCGCCAGCCCCACACCTGGCACGTGGCTTTGTCCTGACCTCTGGTTTCTCATTGGGGTGGACCGTTGAGTAAGGAAACCCCTTTGCTTGTACAGATCATGGCTGCTCTGCAGAGGTTTCTGGGCCACTGAGGGCTCACCTAGGCAGTCTGGGACTGGACCCCAGGCCAGCACCACCCCCTGCGGGCATCCTCAGGTCTCCTGGATCGTGTGAACACCTGGCCCAGCGTGTTGGGCAGACCCTGCCTTCTGCAGCTGATGCCCCTGCTTCCTCCCACCCCTGCTCTCTCGTCTTTTGCAGTGTGACCCTCATTCCCACCTGAGGATCACCAGGGATCGGTCTGAGATGTGGCCTCCACTTTGCTGCAGAGGCGGTTTCAAGGCCAAATTTCCCTTTCATATTCCAGAAGAAAAACCTTGTGGAATTCTGATTTTTCTTGTGTGACCCAGTGGAGTATCATTTCCTCAAGGGCATAGGTGATTTTATCCCTGAACCTAGCACAGTGCCTGGCACGTAGTAGGTGCTGTTTTTGAAAGCTTTTAAAATTACATTTCTTGAAATATAATTCACTGTTTCCTCATATTTTTCAGGGAGACCAGTAGTTCTCAGGTTTGTGTCTGAGCTTGAAATAATTTCGTTTGAATTCACATGTAGCCCTTCAGTCTTCTCTCTGATGATTAATTAGCACACCGAAAGGTAGAGGTAAGGGTGAGAGCATAGAGAGAGGGGCACTCACTGGCAAGGGGAGCCTTTGTCTTTTTCTGGCTCTAGTACTTAGCACAGTGCCCGGCATGCACTAGGTGCTTAATGAATGATGTTGGCTGGCTTCTTGCTCTAAAGCCCACGAGCCTATACGTCTTAGCTGCCCAGCTGACATTCTGACCTAGTGAGAGACTCCCAGGTCAGTTTTATTAAATGCCTTGAGGCCTTTTTTCCCCCACAACTTTCCTCTGCTCAAAACCCCTCCTGTAATCCTACCCCTGTACCTCAGTCCTGCCCTTGTTGCCTTCTCCCCTCCCCCTATCTGGTCTGGGGGCTCCTCTAGAGGCTCAAGAACCCCACAGCTTCCTATTGCTCAAGTAAAAGGCAAACAGAACTCCTACAAGGGGCCCTGTTTGGTGTGGCCCCAGGCTTCCCCTGCAGCTGAGTGACCCCTGCCGGCCCTCATGACATTTCTGGCCAATCCCATCTGCTTCCCCCTTGCCTCCCCCCAAGCAAAGGTCAGCTGGCTCTTTCTCCTTGGCATCGCACACAGCAGTGGCTGTAATGGAGAGGCTCCTGGCCAGGCTGTGGCTCACAGCATCCTTTCATTCTTCACCCTTTTCATTAACAGATGGCGGTGGTCCTGAAGAGAAACAGCCCAAGCTCGAGCTGTCCGAGGAGGAGCTCAGGGCCCACGTTCAGAAGGGGAGCCTGGGCAAGCTCACAGTGCCCGTCCTCAAAGAGGCCTGCCGAGTGTATGGGCTGAAGGCCAGCAGCACCAAGAAGCAGGAGCTTTTGGACCTGCTGAAGCAGCATTTCCAGGAGGCCTGATGGGCCCAGGACCCTCGTCCTCACCCCTCGTTTACCAATAAAGAGAATTGCTTTTCTTTGGAACCAGGCAGTGTCAGGGTTCTCTCCTACTCTTCACAAGTCATTTGAAAGCATTTCCCTTTTCTCCCCTCACCGCCCCTGTAGACTCAAGGATCTGGACCCAGCCCTGGCCTTCAGAAAGAAATGGAGGCAGGGGAGCGGACAGAGCGGCCCCTTCTCTGCAGAGGAGAGAACTCCGTGGGTTAGGAGGCTCCTCACTGGGAGCACAGACCCAGTCCTTGACCTTTCTAGTCCCGGGGGGTGATCCCCCTTCCCGCCCCTTTGTCTCATACCCTGGGCTCACACCACACCTGCAAGGAACACTGACTGCGGGGCCCTCTGTGACACGGAGTGCTCTGGGCAGACCACACAAGAGGCCAGAAGTGGCTTCTTCAAGAAGGCTGCGGCTTCTTAGCACCAACAGTGACCCTGATCAGGGATTCTTAACTGGGAGTCTCTGAACTTCGAGAGACTCCAGGTTTGGGTAACGTCTGTATCAACGCCAGAAGGGGAACGGGCCCACAGCCGTGAGAGGGCAAAGCGGGGACTTCACATCCCTGTGACCAGGCACTTTGGTGGAAAACGGCCTGGTCTCTGTATACTCTACTTGGCCCAGTGTCTGGCACTTAGTAGCCCGCTCTTATCAATGCGCATTCCCTTCCTATACTACCTACAGTATAAGGCCAAGTGCCTCCACGAGGACTGCCATGGGAGCTG

>UoN.Pci.T.20995_UoN.Pci.T.20995.1

AGAGAGAGAGAGAGAGAGAGAGAGAGAGAGAGAGAGAGAGAGAGAGAGAGAGAGAGAGAGAGAGAGAAAGAGATCTTACTAATACAAATAACTGAAGTTTGGTCTTCAGTAATGAATTGTTTGGGGCCAGGACAATTTACAAAACCTAGCTTGTAAGGCATGGAGAGTCTGTGATCTTACACTGATGAAATCAGAGACGTTGGAAGTCCTGACATTGTACATTTGTGACAACATCTGAGAGGCTATGTGGCACATGACATAAAGCTCCAACCTCATAGACAGGAAATCCTGCCTCTCAGACATAATAGCTGTGTTACCATAGGTGAATCACTCAACCTCTCATTGTTCTAGAAAACTCTTTAGGACGATATGAAAGGGTTAGTGACCTATGTGAGTGAACAGATTTTGCACACCATGAGTGTTCTGCTCACAGATGAAATCACAGGTCTGGACAAAAAAAATCTTTTGTGTTTATCTGTTCTGTTCATATGCATGTTCTTTAGCATGTGCCATGGATATCATGAAAGATCAAGTTGCATTAAGTTGCATTAAGGGATGACTTTAGGACCAGAAAGATGACAGAGGCTGAGTCTATGTGGTACCAACATAATTACCATGAGATCACAGATTTGTAGCTGGAAGGGACCTCAGAGACCATCTCTGGTGCAACCACCTCATTAAAATGATCTATAAACCTTCATCTATAAATAGGTGAAAAAACTGAGGCCCAGGTAGTTTAAGTGACATCCAAGAAAAAGGTAAAATCCAACCATTTAATGTTTCATACGTAGAATGCTCCAAAAACTCCAGTAACTGGTTGGGTCAACAGAACCACCTTGATTATGTTGTATAATAGATACCCAATGCTAATGTTAAATGAAGAAGTGAAAAGGTTCCTTCTGGGAATCCTGGTACTAGTGATCACAGTGAAAAAGACCCAGACCAGAGAACTTTCACTCCATTTGCATCCCAATAGAATGGGACATCTCAGGCCTTGTTTCCTGAGAATGTCACCCCTTTTCCATCCTTGGTAAATGCTCAGCCAAGATTCACATATATTCACCCTGGTGGTTTGTGATTCTGTGATATACCAGTAACTTCCTTCTCTTAAGAGGCCACAGGAGTCCAGACAATCACTCCACCCCCCCTTTTTTTTAGGGATCAATTTTTCTTAAATTTCTGTTCCATGTTGTTGGCAGAGTATACTAGGCTTTGTAAATATCTAAAATGGAAACAGACCCACCCAATATAGTTAATGTTAAAAGTAAGAAACAAACAACACAAGAGAGATGAGAAAAGTCCCTGACAGGGCACTGGGCCACGTTTTCTGTTTTTCCATATAATCACAATGTTATCTGGTTCAGCATGTTCTGTTACCATTTAAAATTTATTTCCAATAGCTCTGCTATATATCAATGGGACATGCGCATCCTCTGCTGTTAAGGTATCTTTTTTTTTTTGCATTGGCACCTGTTGTTTTCCCTAAATGTCAAGGACTGACATTTGATATCATTATACCCTATCAGTATATTAATGATTAAATTTCCCATTCCACTCCCTCCCCTACCCCATGTCCCTGCTCCCATGACATTGAATTTAACGGTGACCATCTGTATTGCAAATGAAATCAATAACCCCCAATCTGGCATTCAGTTCTCCCTGAAAAAAGAAAATGGCCTTGAAATGGGGGTGGTGGCAGCTGCAGCTTCCCCTCAAAATGGCCATAGCATTGGGGCTTTCCTGTTTGTCAGATGTAGTATTGCAAGGTGTATCCTCCAGGATGGGGAATGTTAAGAAAATCTCTGTGTGGTGACTTTTTTTTTTGGTAGGGGAATCATTCTTCTTTCTCAAATGAAATATTCTTTTTTGCTCTCATCTCCAGGTTCCTGAAGAGTTGGGTCGTTTTTCAGGGCTTCTTCGGCACTCTCTGCAAACTCTGTCCCCTTGTCTTCATTTGTGTGGTAGGTGCCCTTGTGCCGGAACATGTAATGCAGAATTACAAGCAGGAGGCAGATGAGCACAAAGGCCACAGCAGCAATCACAACTGCAATGATGGTGATGTCATTCCCTGGACTGGTGTCTCCTGCGTCATCCACTGTTGTACTGTTTGGGTCACTCATAACTGACAGACTCCAGAGCGCCCTGGGCCACTGGTCTCGGCTGGCTCAGGGAGGGCTGGGGAGATTGAGCAGAACAGCTGGAAGGATCAGAAAAAGGCAGGAGTCTCCTGGAGAAGTCTCCTCTTGTTCTTGTCTGGTTCCTGACCCTTCGACAACTGGGAAAGGAAACAGCACATGTGTCATTCTCTTTACAGCTCACATGCTGAGGGGAGGGGCTCAAATTTGGAGATAGGCAGGGTTGTTAGCTTTTTTTTTCTTTTTATTTTCCTTTTCTCCTG

>UoN.Pci.T.28362_UoN.Pci.T.28362.1

GATGGCACCTCCGGTCCCTCTGGCCGAACGCTGGAGGCTCGCCCTCGTTCTCTCTTCCGGGAGAAAGCGTGACGTCCTTCGCCGGGGCTGGGGGCGCGGCCGGGGCCGGAGTAAAGATGGCGGCGGCGGTGAGACAGGATTTGGGCCAGCTTATGAACTCGAGCGGGTCTCACAAGGACCTGGCGGGCAAGTATCGACAGATATTGGAAAAAGCCATTCAGTTGCCCGGGGCAGAACAGCTTGAAGCTTTGAAAGCCTTTGTGGAAGCAAGTAAGTGGAACTGAAATGTAATACTTGGTTTCGTGTTTCCTGTAGTGAGCTTTGAAAATGTGAGCTAATCATATCAGTGTTGGTGCCCCCTTGTGTTGTGTTTTTTGATATCACATAGAGATTTTTAAATTATATATGCCATGCGATTTATTTTGAAACAAATGAAAGATTGCAATCCTTAATTTAGCGGCATATTATAGTAGTTAAGTGAGTTATTTTTGAATTATGAAATCTCTTATCAGAAATGCTTAATTACTTCAGGCATCATTCAGATTTTAAACATTTAATTTATGATGAAATTTGCCCTATAGTTTATAATTCAATTCAGTAATCATTTGTTACCTGCTGTGGTCACTATAGGCACTGAGGACATAAAACTAGAAGCAGGGCCTGCCCTCCGGGATCTTGCCTCCCCTGGGAGGATGAGGCCCACCCCCATGCAAGTAAACACCTGGGGATTGGAGGAAGGGGAGAGCATTGACCAAGGGAGGGCACCGGGAAGGGTATGGTGGAGGTGTAACGCCGAGCTTAGCCTCCCGGGAATTAACGAATAAGTAAAAATATTTACTGAGCACTTACTGTGTGCCTAGTACGGTGCCATGTGCTGGATACAGAATTTCCGACAGTTCCTGCCCTCAGGGATCTTAAATCCTAATGGTGACCAGGGAGGCTGTTTTGGTTTTTAAGTTATAAGGATGGACGGTGGGGCCACGGGAGGAGGATTTTAATATACCCCTTTCTAGGATCAGTGATAGAGCTGAATTAACAATAATAATAATGTAATAGTAGTAGCTATGTATTATCTCATTTAATCATAACACAACTCTGGGAGGTAAGTGGGATTACTATCTCCATTACAGATGAGGAAACTGAGGCAGCGCCAGGTTAAGTGGCTTGCCCAGGGTCACACAGCTAGTAAGCGTCTGAGGCCAGAGTCTACACTGTAGCCCAGCACTCTGCCCATCATACTTCCTCATTTGATTTGAATAGTTCTCACACTGGCAAGGGGGTAAGGCTGGTGCTGACAAGGTGGCGGTCATAGCCAGAGCGTGGGTTCCAGTCTTGTTGTTGGCAGCTGTCGCGATAGCCTGGGAAGGGAAGAATTTGAAGAAGAGGCGAGATCAGGGAACTCGTAGAAGAAGGCCTCAGTTTTCTTAGGTAAGTAGGGGGCCAGGTCATCCAAGACTCCAGAGACAAAGACATTTCATGGCCGAGGAGCTTTGGAACACTTGCTGTGAGGCTCCTGCCATTCGAGCCGAGGCCCAGGGTTTGCTGGGAAGCAGTGGCCGAGGTGAAGTGGAGTAATGTGGATTGGTGGCACACCAGCCGGCATGGCTTTGTGACTTTTTCTAGGAGTATTCCAAAGCTTGGAAGTCAAGGCTAGAAAAGGAACGACTGAGGGTTAGGATTAGAGTTAGGCAGACAGTTTTAAGTGGCATTTATTGGAAGAGCTTCTTCAGCTGCTGCTGTTATTTTATAATAAGACCAGGCCCCAGAATGTCAGAAGGACATACCTACACATGGGACATTTGAAGAGCCTCTGTATTCCGCTAAGGAAGAAGCAAGGGACTCAGGCAGAAGGAGGAGCCATGTTAGCGACCAGGTGGAGGGCTTCTGGGCTGGGGCAGAGCTGCCAGGGGCCAAGCTGTTGCTGAGTTTAGGGCTGAGGAGGGAGCCCTGGCCTCTGCCCTTAAAGCCAAGTGGCTCCTCAGGGACCTGCAGGCCAGCTAGCCCCAGTACCAGAGGGGCCCTTTCGGGCTAGGCAGGATGGGACAAGGTGACCTATTCAGGGAGCCTCTTCTGTCCCTTGAAAAGCCTGGGGCCCTTCCCATCTTCCAGATTTTTACAAAAGGATGATTAATCATCCCCGAATAAACTGCTCCTGAAATTCAAGTGGGGAGAAACCAAAAAGCACTTGGGTACCTCTGTTTGTATCTGTACTTTCTCAAGGAGTTTGGCTTTAGTCAGATGGAAAATGGGGCGGTAGTGTGTTGTGGTAGAAAGGGTGCAGAAGCCACGTTTAGGCCTCAGGACGTTCATTTTATGGACAAAGGCCAGAAATGGGGTCCTGCAAATCCCTCCCCAGCCTAGGAAGTGTCCACAGGTTCTCGGCAGTCTTCCCTGAGAGAAGCAAGATGAGGCCGGGCCCTGCCTCCAACTAGCCCTGTCCCACTTCTCCGTCTCTTTCAGCTTTTCTGGGGTGGGGTGGGGAGCTTGGAGCACCTGGGAGGAAGGAGAGCTGTTCTTTGTGCTCAGCTGCTTGGTCTGAGTTTTGACGCCCTAGGTGACGCTCCAGAAGAGCTGGTCCCCTGGTCCTTCAAAGGTGGCCAGAGAGCAAAAGCAGATTTTAGTCTCTTCTGTGACCCTAACTAGGGGTCTCTCTTTCCCACTCTTTAAAGTGAAGGGGTTGAACAGAGGTTCCCTTCCAGGCCTGACAGCCTGTCAGATTTGGGTTCCCCTCATCTCTAGTGAGACTAGTGTCACCAGTGCTGCTCTGTGACTTTAGACACGTCTTTTCTCCTTTTTGGGACCTTCCTTTTCTCTTCTGTAAAATCAAAGAGGAGCTAGATGATTCCCAAGATTCCTTCTTTCTCTGATCCCTTGAAGGTTGTTTTTTTTGGGGGGTGGGAGGAGGGGGCGGCCTCAAGTTCTCTTTCCATCCTCTCCCCGAAAGCTTCTCTGTTTGTTACAAAGCATCTACCATTCCTTGACTTCTTAAATGCCAAGGGTGAAGAAGCAAGTGATGCTTCTATAGAAGTACGTGACATGGTCAGTTTCAGTGTGAAACCTCCTCATGGCTCTCTTCCACTAAGGTGGGAGACTGACTAGCCCTTCTTGCCCTGGCCTTGTGCTGGCTGGGGTCTGTGGCTCCCCTCCAGAGAGGAGTGTCTGCTCTCCCCATCCCTTCCCCAAGTCAGGGCGCCTGGCATGCTGTTTGCCCCTCACTGCAGCAATAATTTCCTAGCTAGAGGTTCTGATCACAATTCATTCCAATTAATGTTATCAGCTAATGAACAAAAAATACAATATCAGTTCTTTGCTTAAAAAATAGGATCTTCGACCTTGTTTTTTAAAAGCAGTTTGTTTATAGCATAGAAATTAATTGACTTTAGCAGGGCTTGGTTAATTTCATTGTGGGAGGCTGTGTAACCTTTTCTGTTAAATTTTGCTTTTTGAATTGATTTGTAACTATTATACCATTATGTTTACAAACTTTTAAAAACAAATGTAGGGGGCAGGTAGGTGGCACAGTGGATAGAGCACCGGCCCTGGAGTCAGGAGCACCTGAGTTCAAATCTAGCCTCAGACATGTGATACTCAATAGCTGTGTGACCCTGGGCAAGTCACTTAACCCCAATGGCCTCACCAAAAAAAAAAAATGTAGGTAGGGGAAAGAAAGCACAATTCTCAGTTGTTTATGAAAACTATCTTTGGATATTATTTTTAAAAAATCAAGTGAGTCAATTCAGTTCAGCAAACATAAGGTGTCCCTTTTGTAAGAGTTAAGCAGATGAAAAATTTGTCTTGTGAAATATTTGAAAAGTATGCAGATTCCTCATATTTCTTTGTCTCATGTTTTCAAACCGAGTGGTAAATGAGAACGTCAGTCTAGTGATCTCTCGGCAGCTGCTGACCGACTTCTGCACACACCTGCCGAGTCTGCCTGACGGCACGGCCAAAGAAGTCTATCATTTCACCTTGGAAAAGATCCAGCCCAGAGTCATCTCATTTGAGGAGCAGGTTGCTTCAATAAGACAGCATCTTGCATCGATATATGAGAAAGAAGAAGACTGGAGAAATGCAGCCCAAGTGTTGGTGGGGATTCCCTTGGAGACAGGACAAAAGCAGTACAATGTGGATTATAAACTGGAGACCTATCTGAAAATCGCCAGGCTCTACCTGGAGGACGACGACCCTGTGCAGGCCGAGGCCTACATCAACCGAGCTTCCTTGCTGCAGAACGAGTCCGCCAATGAGCAGCTGCAGATCCACTACAAGGTGTGCTATGCTCGTGTTCTTGATTATAGAAGGAAGTTCATCGAAGCTGCCCAAAGATACAATGAGCTCTCCTATAAGACGATAGTCCATGAAAGTGAGAGACTAGAGGCCCTGAAACATGCTCTGCACTGTACCATCTTAGCGTCAGCAGGACAACAGCGCTCTCGAATGCTAGCTACTCTTTTTAAGGATGAAAGGTGTCAGCAGCTCGCAGCCTATGGAATCCTAGAGAAGATGTATCTAGATAGGATTATCAGAGGAAATCAGCTTCAAGAATTTGCTGCTATGTTGATGCCTCACCAAAAAGCAACTACAGCCGATGGTTCCAGCATCTTGGACAGAGCTGTCATTGAACACAATTTGTTGTCTGCAAGCAAGTTATATAATAATATTACATTTGAAGAGCTTGGAGCTCTATTAGAGATCCCTGCAGCTAAGGCAGAAAAGATAGCATCTCAGATGATTACCGAAGGCCGTATGAATGGATTTATCGATCAGATTGATGGAATAGTTCATTTTGAAACCCGTGAGGCTTTGCCAACGTGGGACAAACAGATTCAGTCACTTTGTTTCCAAGTGAATAACCTTTTGGAGAAGATAAGCCAGACAGCACCCGAGTGGACGGCGCAGGCCATGGAGGCCCAGATGGCCCAGTGACTCGACGCTCATTTGACGGCTTCTGCAGCGTCTCTGTGATCGTACAGATTAATTTCATTACGACTCCAAAGAATCTGTATTATGACCCTGCACATTTCCGTGTCCTCTTACAACAGAATCTATTTCAAGAAGCATTATTTCAGAGGAGATGGTATGAACGAACACTTTAAATTTCTCAGTCCTCAGTCCCCGTGTTTGGTGGGTGTCTGTCTATCAGACTGCTGCGGTTGTTTTGAAGGTGTTTGTTTAAATTGCTGGAGCGAAATCTCTGTGACTAAAAAGATTTATTATAGTTCTTCACCTTGTGCTACGTTGTACGTGTGCTTTATTCCTCTCAGATGTTCCAATAAAGAGGGGAAGGAGCCAAATACAAATGGGTTTTTGTGTTTTAATGATTCAGTATTTTTTCCTCCTCAGTTTTCTATGGTTACATGAGACAAAAATGCCTTTTCTACAGCTTCACGTCTGTTCCTAACACTTTTCTTTAATCAGAAATGAAGCCGTATTGCTACATGCCCCTTATTTATATGCATCGGTGTTTGTTATATTAAAAAAACCCATTCCTACATGTGAGATTTGGGGAGTCATTGTATTATTATTAGAGCTATATTTTAAAATAAGGACCCCAGGGAAATGGAACGAAAATATAACTAAGTCATTGATAGAAATCTCCTCAGAAGGATGCAAAACCTTAGTCTTTGTAACTCATAGACCAAAGTTCACTGGCAGTAAGCTGAAATTTCTAAGAACCAAATTTTTGTAATAATGTGAATAAAAAGTATAAAAATCAGTATTAACATAAAATATGTACACAG

>UoN.Pci.T.28362_UoN.Pci.T.28362.2

GATGGCACCTCCGGTCCCTCTGGCCGAACGCTGGAGGCTCGCCCTCGTTCTCTCTTCCGGGAGAAAGCGTGACGTCCTTCGCCGGGGCTGGGGGCGCGGCCGGGGCCGGAGTAAAGATGGCGGCGGCGGTGAGACAGGATTTGGGCCAGCTTATGAACTCGAGCGGGTCTCACAAGGACCTGGCGGGCAAGTATCGACAGATATTGGAAAAAGCCATTCAGTTGCCCGGGGCAGAACAGCTTGAAGCTTTGAAAGCCTTTGTGGAAGCAAGTAAGTGGAACTGAAATGTAATACTTGGTTTCGTGTTTCCTGTAGTGAGCTTTGAAAATGTGAGCTAATCATATCAGTGTTGGTGCCCCCTTGTGTTGTGTTTTTTGATATCACATAGAGATTTTTAAATTATATATGCCATGCGATTTATTTTGAAACAAATGAAAGATTGCAATCCTTAATTTAGCGGCATATTATAGTAGTTAAGTGAGTTATTTTTGAATTATGAAATCTCTTATCAGAAATGCTTAATTACTTCAGGCATCATTCAGATTTTAAACATTTAATTTATGATGAAATTTGCCCTATAGTTTATAATTCAATTCAGTAATCATTTGTTACCTGCTGTGGTCACTATAGGCACTGAGGACATAAAACTAGAAGCAGGGCCTGCCCTCCGGGATCTTGCCTCCCCTGGGAGGATGAGGCCCACCCCCATGCAAGTAAACACCTGGGGATTGGAGGAAGGGGAGAGCATTGACCAAGGGAGGGCACCGGGAAGGGTATGGTGGAGGTGTAACGCCGAGCTTAGCCTCCCGGGAATTAACGAATAAGTAAAAATATTTACTGAGCACTTACTGTGTGCCTAGTACGGTGCCATGTGCTGGATACAGAATTTCCGACAGTTCCTGCCCTCAGGGATCTTAAATCCTAATGGTGACCAGGGAGGCTGTTTTGGTTTTTAAGTTATAAGGATGGACGGTGGGGCCACGGGAGGAGGATTTTAATATACCCCTTTCTAGGATCAGTGATAGAGCTGAATTAACAATAATAATAATGTAATAGTAGTAGCTATGTATTATCTCATTTAATCATAACACAACTCTGGGAGGTAAGTGGGATTACTATCTCCATTACAGATGAGGAAACTGAGGCAGCGCCAGGTTAAGTGGCTTGCCCAGGGTCACACAGCTAGTAAGCGTCTGAGGCCAGAGTCTACACTGTAGCCCAGCACTCTGCCCATCATACTTCCTCATTTGATTTGAATAGTTCTCACACTGGCAAGGGGGTAAGGCTGGTGCTGACAAGGTGGCGGTCATAGCCAGAGCGTGGGTTCCAGTCTTGTTGTTGGCAGCTGTCGCGATAGCCTGGGAAGGGAAGAATTTGAAGAAGAGGCGAGATCAGGGAACTCGTAGAAGAAGGCCTCAGTTTTCTTAGGTAAGTAGGGGGCCAGGTCATCCAAGACTCCAGAGACAAAGACATTTCATGGCCGAGGAGCTTTGGAACACTTGCTGTGAGGCTCCTGCCATTCGAGCCGAGGCCCAGGGTTTGCTGGGAAGCAGTGGCCGAGGTGAAGTGGAGTAATGTGGATTGGTGGCACACCAGCCGGCATGGCTTTGTGACTTTTTCTAGGAGTATTCCAAAGCTTGGAAGTCAAGGCTAGAAAAGGAACGACTGAGGGTTAGGATTAGAGTTAGGCAGACAGTTTTAAGTGGCATTTATTGGAAGAGCTTCTTCAGCTGCTGCTGTTATTTTATAATAAGACCAGGCCCCAGAATGTCAGAAGGACATACCTACACATGGGACATTTGAAGAGCCTCTGTATTCCGCTAAGGAAGAAGCAAGGGACTCAGGCAGAAGGAGGAGCCATGTTAGCGACCAGGTGGAGGGCTTCTGGGCTGGGGCAGAGCTGCCAGGGGCCAAGCTGTTGCTGAGTTTAGGGCTGAGGAGGGAGCCCTGGCCTCTGCCCTTAAAGCCAAGTGGCTCCTCAGGGACCTGCAGGCCAGCTAGCCCCAGTACCAGAGGGGCCCTTTCGGGCTAGGCAGGATGGGACAAGGTGACCTATTCAGGGAGCCTCTTCTGTCCCTTGAAAAGCCTGGGGCCCTTCCCATCTTCCAGATTTTTACAAAAGGATGATTAATCATCCCCGAATAAACTGCTCCTGAAATTCAAGTGGGGAGAAACCAAAAAGCACTTGGGTACCTCTGTTTGTATCTGTACTTTCTCAAGGAGTTTGGCTTTAGTCAGATGGAAAATGGGGCGGTAGTGTGTTGTGGTAGAAAGGGTGCAGAAGCCACGTTTAGGCCTCAGGACGTTCATTTTATGGACAAAGGCCAGAAATGGGGTCCTGCAAATCCCTCCCCAGCCTAGGAAGTGTCCACAGGTTCTCGGCAGTCTTCCCTGAGAGAAGCAAGATGAGGCCGGGCCCTGCCTCCAACTAGCCCTGTCCCACTTCTCCGTCTCTTTCAGCTTTTCTGGGGTGGGGTGGGGAGCTTGGAGCACCTGGGAGGAAGGAGAGCTGTTCTTTGTGCTCAGCTGCTTGGTCTGAGTTTTGACGCCCTAGGTGACGCTCCAGAAGAGCTGGTCCCCTGGTCCTTCAAAGGTGGCCAGAGAGCAAAAGCAGATTTTAGTCTCTTCTGTGACCCTAACTAGGGGTCTCTCTTTCCCACTCTTTAAAGTGAAGGGGTTGAACAGAGGTTCCCTTCCAGGCCTGACAGCCTGTCAGATTTGGGTTCCCCTCATCTCTAGTGAGACTAGTGTCACCAGTGCTGCTCTGTGACTTTAGACACGTCTTTTCTCCTTTTTGGGACCTTCCTTTTCTCTTCTGTAAAATCAAAGAGGAGCTAGATGATTCCCAAGATTCCTTCTTTCTCTGATCCCTTGAAGGTTGTTTTTTTTGGGGGGTGGGAGGAGGGGGCGGCCTCAAGTTCTCTTTCCATCCTCTCCCCGAAAGCTTCTCTGTTTGTTACAAAGCATCTACCATTCCTTGACTTCTTAAATGCCAAGGGTGAAGAAGCAAGTGATGCTTCTATAGAAGTACGTGACATGGTCAGTTTCAGTGTGAAACCTCCTCATGGCTCTCTTCCACTAAGGTGGGAGACTGACTAGCCCTTCTTGCCCTGGCCTTGTGCTGGCTGGGGTCTGTGGCTCCCCTCCAGAGAGGAGTGTCTGCTCTCCCCATCCCTTCCCCAAGTCAGGGCGCCTGGCATGCTGTTTGCCCCTCACTGCAGCAATAATTTCCTAGCTAGAGGTTCTGATCACAATTCATTCCAATTAATGTTATCAGCTAATGAACAAAAAATACAATATCAGTTCTTTGCTTAAAAAATAGGATCTTCGACCTTGTTTTTTAAAAGCAGTTTGTTTATAGCATAGAAATTAATTGACTTTAGCAGGGCTTGGTTAATTTCATTGTGGGAGGCTGTGTAACCTTTTCTGTTAAATTTTGCTTTTTGAATTGATTTGTAACTATTATACCATTATGTTTACAAACTTTTAAAAACAAATGTAGGGGGCAGGTAGGTGGCACAGTGGATAGAGCACCGGCCCTGGAGTCAGGAGCACCTGAGTTCAAATCTAGCCTCAGACATGTGATACTCAATAGCTGTGTGACCCTGGGCAAGTCACTTAACCCCAATGGCCTCACCAAAAAAAAAAAATGTAGGTAGGGGAAAGAAAGCACAATTCTCAGTTGTTTATGAAAACTATCTTTGGATATTATTTTTAAAAAATCAAGTGAGTCAATTCAGTTCAGCAAACATAAGGTGTCCCTTTTGTAAGAGTTAAGCAGATGAAAAATTTGTCTTGTGAAATATTTGAAAAGTATGCAGATTCCTCATATTTCTTTGTCTCATGTTTTCAAACCGAGTGGTAAATGAGAACGTCAGTCTAGTGATCTCTCGGCAGCTGCTGACCGACTTCTGCACACACCTGCCGAGTCTGCCTGACGGCACGGCCAAAGAAGTCTATCATTTCACCTTGGAAAAGATCCAGCCCAGAGTCATCTCATTTGAGGAGCAGGTTGCTTCAATAAGACAGCATCTTGCATCGATATATGAGAAAGAAGAAGACTGGAGAAATGCAGCCCAAGTGTTGGTGGGGATTCCCTTGGAGACAGGACAAAAGCAGTACAATGTGGATTATAAACTGGAGACCTATCTGAAAATCGCCAGGCTCTACCTGGAGGACGACGACCCTGTGCAGGCCGAGGCCTACATCAACCGAGCTTCCTTGCTGCAGAACGAGTCCGCCAATGAGCAGCTGCAGATCCACTACAAGGTGTGCTATGCTCGTGTTCTTGATTATAGAAGGAAGTTCATCGAAGCTGCCCAAAGATACAATGAGCTCTCCTATAAGACGATAGTCCATGAAAGTGAGAGACTAGAGGCCCTGAAACATGCTCTGCACTGTACCATCTTAGCGTCAGCAGGACAACAGCGCTCTCGAATGCTAGCTACTCTTTTTAAGGATGAAAGGTGTCAGCAGCTCGCAGCCTATGGAATCCTAGAGAAGATGTATCTAGATAGGATTATCAGAGGAAATCAGCTTCAAGAATTTGCTGCTATGTTGATGCCTCACCAAAAAGCAACTACAGCCGATGGTTCCAGCATCTTGGACAGAGCTGTCATTGAACACAATTTGTTGTCTGCAAGCAAGTTATATAATAATATTACATTTGAAGAGCTTGGAGCTCTATTAGAGATCCCTGCAGCTAAGGCAGAAAAGATAGCATCTCAGATGATTACCGAAGGCCGTATGAATGGATTTATCGATCAGATTGATGGAATAGTTCATTTTGAAACCCGTGAGGCTTTGCCAACGTGGGACAAACAGATTCAGTCACTTTGTTTCCAAGTGAATAACCTTTTGGAGAAGATAAGCCAGACAGCACCCGAGTGGACGGCGCAGGCCATGGAGGCCCAGATGGCCCAGTGACTCGACGCTCATTTGACGGCTTCTGCAGCGTCTCTGTGATCGTACAGATTAATTTCATTACGACTCCAAAGAATCTGTATTATGACCCTGCACATTTCCGTGTCCTCTTACAACAGAATCTATTTCAAGAAGCATTATTTCAGAGGAGATGGTATGAACGAACACTTTAAATTTCTCAGTCCTCAGTCCCCGTGTTTGGTGGGTGTCTGTCTATCAGACTGCTGCGGTTGTTTTGAAGGTGTTTGTTTAAATTGCTGGAGCGAAATCTCTGTGACTAAAAAGATTTATTATAGTTCTTCACCTTGTGCTACGTTGTACGTGTGCTTTATTCCTCTCAGATGTTCCAATAAAGAGGGGAAGGAGCCAAATACAAATGGGTTTTTGTGTTTTAATGATTCAGTATTTTTTCCTCCTCAGTTTTCTATGGTTACATGAGACAAAAATGCCTTTTCTACAGCTTCACGTCTGTTCCTAACACTTTTCTTTAATCAGAAATGAAGCCGTATTGCTACATGCCCCTTATTTATATGCATCGGTGTTTGTTATATTAAAAAAACCCATTCCTACATGTGAGATTTGGGGAGTCATTGTATTATTATTAGAGCTATATTTTAAAATAAGGACCCCAGGGAAATGGAACGAAAATATAACTAAGTCATTGATAGAAATCTCCTCAGAAGGATGCAAAACCTTAGTCTTTGTAACTCATAGACCAAAGTTCACTGGCAGTAAGCTGAAATTTCTAAGAACCAAATTTTTGTAATAATGTGAATAAAAAGTATAAAAATCAGTATTAACATAAAATATGTACACAG

>UoN.Pci.T.28362_UoN.Pci.T.28362.3

GTCCTTCGCCGGGGCTGGGGGCGCGGCCGGGGCCGGAGTAAAGATGGCGGCGGCGGTGAGACAGGATTTGGGCCAGCTTATGAACTCGAGCGGGTCTCACAAGGACCTGGCGGGCAAGTATCGACAGATATTGGAAAAAGCCATTCAGTTGCCCGGGGCAGAACAGCTTGAAGCTTTGAAAGCCTTTGTGGAAGCAAGTAAGTGGAACTGAAATGTAATACTTGGTTTCGTGTTTCCTGTAGTGAGCTTTGAAAATGTGAGCTAATCATATCAGTGTTGGTGCCCCCTTGTGTTGTGTTTTTTGATATCACATAGAGATTTTTAAATTATATATGCCATGCGATTTATTTTGAAACAAATGAAAGATTGCAATCCTTAATTTAGCGGCATATTATAGTAGTTAAGTGAGTTATTTTTGAATTATGAAATCTCTTATCAGAAATGCTTAATTACTTCAGGCATCATTCAGATTTTAAACATTTAATTTATGATGAAATTTGCCCTATAGTTTATAATTCAATTCAGTAATCATTTGTTACCTGCTGTGGTCACTATAGGCACTGAGGACATAAAACTAGAAGCAGGGCCTGCCCTCCGGGATCTTGCCTCCCCTGGGAGGATGAGGCCCACCCCCATGCAAGTAAACACCTGGGGATTGGAGGAAGGGGAGAGCATTGACCAAGGGAGGGCACCGGGAAGGGTATGGTGGAGGTGTAACGCCGAGCTTAGCCTCCCGGGAATTAACGAATAAGTAAAAATATTTACTGAGCACTTACTGTGTGCCTAGTACGGTGCCATGTGCTGGATACAGAATTTCCGACAGTTCCTGCCCTCAGGGATCTTAAATCCTAATGGTGACCAGGGAGGCTGTTTTGGTTTTTAAGTTATAAGGATGGACGGTGGGGCCACGGGAGGAGGATTTTAATATACCCCTTTCTAGGATCAGTGATAGAGCTGAATTAACAATAATAATAATGTAATAGTAGTAGCTATGTATTATCTCATTTAATCATAACACAACTCTGGGAGGTAAGTGGGATTACTATCTCCATTACAGATGAGGAAACTGAGGCAGCGCCAGGTTAAGTGGCTTGCCCAGGGTCACACAGCTAGTAAGCGTCTGAGGCCAGAGTCTACACTGTAGCCCAGCACTCTGCCCATCATACTTCCTCATTTGATTTGAATAGTTCTCACACTGGCAAGGGGGTAAGGCTGGTGCTGACAAGGTGGCGGTCATAGCCAGAGCGTGGGTTCCAGTCTTGTTGTTGGCAGCTGTCGCGATAGCCTGGGAAGGGAAGAATTTGAAGAAGAGGCGAGATCAGGGAACTCGTAGAAGAAGGCCTCAGTTTTCTTAGGTAAGTAGGGGGCCAGGTCATCCAAGACTCCAGAGACAAAGACATTTCATGGCCGAGGAGCTTTGGAACACTTGCTGTGAGGCTCCTGCCATTCGAGCCGAGGCCCAGGGTTTGCTGGGAAGCAGTGGCCGAGGTGAAGTGGAGTAATGTGGATTGGTGGCACACCAGCCGGCATGGCTTTGTGACTTTTTCTAGGAGTATTCCAAAGCTTGGAAGTCAAGGCTAGAAAAGGAACGACTGAGGGTTAGGATTAGAGTTAGGCAGACAGTTTTAAGTGGCATTTATTGGAAGAGCTTCTTCAGCTGCTGCTGTTATTTTATAATAAGACCAGGCCCCAGAATGTCAGAAGGACATACCTACACATGGGACATTTGAAGAGCCTCTGTATTCCGCTAAGGAAGAAGCAAGGGACTCAGGCAGAAGGAGGAGCCATGTTAGCGACCAGGTGGAGGGCTTCTGGGCTGGGGCAGAGCTGCCAGGGGCCAAGCTGTTGCTGAGTTTAGGGCTGAGGAGGGAGCCCTGGCCTCTGCCCTTAAAGCCAAGTGGCTCCTCAGGGACCTGCAGGCCAGCTAGCCCCAGTACCAGAGGGGCCCTTTCGGGCTAGGCAGGATGGGACAAGGTGACCTATTCAGGGAGCCTCTTCTGTCCCTTGAAAAGCCTGGGGCCCTTCCCATCTTCCAGATTTTTACAAAAGGATGATTAATCATCCCCGAATAAACTGCTCCTGAAATTCAAGTGGGGAGAAACCAAAAAGCACTTGGGTACCTCTGTTTGTATCTGTACTTTCTCAAGGAGTTTGGCTTTAGTCAGATGGAAAATGGGGCGGTAGTGTGTTGTGGTAGAAAGGGTGCAGAAGCCACGTTTAGGCCTCAGGACGTTCATTTTATGGACAAAGGCCAGAAATGGGGTCCTGCAAATCCCTCCCCAGCCTAGGAAGTGTCCACAGGTTCTCGGCAGTCTTCCCTGAGAGAAGCAAGATGAGGCCGGGCCCTGCCTCCAACTAGCCCTGTCCCACTTCTCCGTCTCTTTCAGCTTTTCTGGGGTGGGGTGGGGAGCTTGGAGCACCTGGGAGGAAGGAGAGCTGTTCTTTGTGCTCAGCTGCTTGGTCTGAGTTTTGACGCCCTAGGTGACGCTCCAGAAGAGCTGGTCCCCTGGTCCTTCAAAGGTGGCCAGAGAGCAAAAGCAGATTTTAGTCTCTTCTGTGACCCTAACTAGGGGTCTCTCTTTCCCACTCTTTAAAGTGAAGGGGTTGAACAGAGGTTCCCTTCCAGGCCTGACAGCCTGTCAGATTTGGGTTCCCCTCATCTCTAGTGAGACTAGTGTCACCAGTGCTGCTCTGTGACTTTAGACACGTCTTTTCTCCTTTTTGGGACCTTCCTTTTCTCTTCTGTAAAATCAAAGAGGAGCTAGATGATTCCCAAGATTCCTTCTTTCTCTGATCCCTTGAAGGTTGTTTTTTTTGGGGGGTGGGAGGAGGGGGCGGCCTCAAGTTCTCTTTCCATCCTCTCCCCGAAAGCTTCTCTGTTTGTTACAAAGCATCTACCATTCCTTGACTTCTTAAATGCCAAGGGTGAAGAAGCAAGTGATGCTTCTATAGAAGTACGTGACATGGTCAGTTTCAGTGTGAAACCTCCTCATGGCTCTCTTCCACTAAGGTGGGAGACTGACTAGCCCTTCTTGCCCTGGCCTTGTGCTGGCTGGGGTCTGTGGCTCCCCTCCAGAGAGGAGTGTCTGCTCTCCCCATCCCTTCCCCAAGTCAGGGCGCCTGGCATGCTGTTTGCCCCTCACTGCAGCAATAATTTCCTAGCTAGAGGTTCTGATCACAATTCATTCCAATTAATGTTATCAGCTAATGAACAAAAAATACAATATCAGTTCTTTGCTTAAAAAATAGGATCTTCGACCTTGTTTTTTAAAAGCAGTTTGTTTATAGCATAGAAATTAATTGACTTTAGCAGGGCTTGGTTAATTTCATTGTGGGAGGCTGTGTAACCTTTTCTGTTAAATTTTGCTTTTTGAATTGATTTGTAACTATTATACCATTATGTTTACAAACTTTTAAAAACAAATGTAGGGGGCAGGTAGGTGGCACAGTGGATAGAGCACCGGCCCTGGAGTCAGGAGCACCTGAGTTCAAATCTAGCCTCAGACATGTGATACTCAATAGCTGTGTGACCCTGGGCAAGTCACTTAACCCCAATGGCCTCACCAAAAAAAAAAAATGTAGGTAGGGGAAAGAAAGCACAATTCTCAGTTGTTTATGAAAACTATCTTTGGATATTATTTTTAAAAAATCAAGTGAGTCAATTCAGTTCAGCAAACATAAGGTGTCCCTTTTGTAAGAGTTAAGCAGATGAAAAATTTGTCTTGTGAAATATTTGAAAAGTATGCAGATTCCTCATATTTCTTTGTCTCATGTTTTCAAACCGAGTGGTAAATGAGAACGTCAGTCTAGTGATCTCTCGGCAGCTGCTGACCGACTTCTGCACACACCTGCCGAGTCTGCCTGACGGCACGGCCAAAGAAGTCTATCATTTCACCTTGGAAAAGATCCAGCCCAGAGTCATCTCATTTGAGGAGCAGGTTGCTTCAATAAGACAGCATCTTGCATCGATATATGAGAAAGAAGAAGACTGGAGAAATGCAGCCCAAGTGTTGGTGGGGATTCCCTTGGAGACAGGACAAAAGCAGTACAATGTGGATTATAAACTGGAGACCTATCTGAAAATCGCCAGGCTCTACCTGGAGGACGACGACCCTGTGCAGGCCGAGGCCTACATCAACCGAGCTTCCTTGCTGCAGAACGAGTCCGCCAATGAGCAGCTGCAGATCCACTACAAGGTGTGCTATGCTCGTGTTCTTGATTATAGAAGGAAGTTCATCGAAGCTGCCCAAAGATACAATGAGCTCTCCTATAAGACGATAGTCCATGAAAGTGAGAGACTAGAGGCCCTGAAACATGCTCTGCACTGTACCATCTTAGCGTCAGCAGGACAACAGCGCTCTCGAATGCTAGCTACTCTTTTTAAGGATGAAAGGTGTCAGCAGCTCGCAGCCTATGGAATCCTAGAGAAGATGTATCTAGATAGGATTATCAGAGGAAATCAGCTTCAAGAATTTGCTGCTATGTTGATGCCTCACCAAAAAGCAACTACAGCCGATGGTTCCAGCATCTTGGACAGAGCTGTCATTGAACACAATTTGTTGTCTGCAAGCAAGTTATATAATAATATTACATTTGAAGAGCTTGGAGCTCTATTAGAGATCCCTGCAGCTAAGGCAGAAAAGATAGCATCTCAGATGATTACCGAAGGCCGTATGAATGGATTTATCGATCAGATTGATGGAATAGTTCATTTTGAAACCCGTGAGGCTTTGCCAACGTGGGACAAACAGATTCAGTCACTTTGTTTCCAAGTGAATAACCTTTTGGAGAAGATAAGCCAGACAGCACCCGAGTGGACGGCGCAGGCCATGGAGGCCCAGATGGCCCAGTGACTCGACGCTCATTTGACGGCTTCTGCAGCGTCTCTGTGATCGTACAGATTAATTTCATTACGACTCCAAAGAATCTGTATTATGACCCTGCACATTTCCGTGTCCTCTTACAACAGAATCTATTTCAAGAAGCATTATTTCAGAGGAGATGGTATGAACGAACACTTTAAATTTCTCAGTCCTCAGTCCCCGTGTTTGGTGGGTGTCTGTCTATCAGACTGCTGCGGTTGTTTTGAAGGTGTTTGTTTAAATTGCTGGAGCGAAATCTCTGTGACTAAAAAGATTTATTATAGTTCTTCACCTTGTGCTACGTTGTACGTGTGCTTTATTCCTCTCAGATGTTCCAATAAAGAGGGGAAGGAGCCAAATACAAATGGGTTTTTGTGTTTTAATGATTCAGTATTTTTTCCTCCTCAGTTTTCTATGGTTACATGAGACAAAAATGCCTTTTCTACAGCTTCACGTCTGTTCCTAACACTTTTCTTTAATCAGAAATGAAGCCGTATTGCTACATGCCCCTTATTTATATGCATCGGTGTTTGTTATATTAAAAAAACCCATTCCTACATGTGAGATTTGGGGAGTCATTGTATTATTATTAGAGCTATATTTTAAAATAAGGACCCCAGGGAAAT

>UoN.Pci.T.29759_UoN.Pci.T.29759.1

AGAGGCAATGGATTCAAGACACAAATTGAGACCCACATATTTGGACATGGCCAATGCAGAAGTTTGTTTCATAGAATAAGCGTATTTATTGTGAGGTTTTGGTTTTCTTTTTTTTTTTTGCAATGGGGCAAGAGAGATAAAAAAAATATTTAATTGAAAAAAGTAAAGTCTTTTGAAAAGAATGCATAATCTCCTTGAGGGCAGGTCTTTGTCTCCCCACTGTTTAGCACAGAGAAGGTACATCACAAGTGCTTACTGATTGATTCCAGAAGATTCCTGATGACACATGGTACCCACCTCCTGAGAGAGGAGTGCAGAGTGAGACATATATACACTTCTGGACATGGACAATTTGGGAATTTGTTTTGCTCAAATAAACATATCACCAGGGCTTTGTTTTTTTCTTTCTTTTTTTCAACTTGGGAAGAAGATGGCAGGGAGAAAAGCCTAGTTTTAGTTAACTGAAAAAGAAAATAAAATTTAATTAAAAAAATAAACCACCCAAAGCTGCACAGCCCCAGAATGCCAAGCCACTGATGATTCCTATCCTCAGGATCAGTGAAAGTCCCCTCTCCCGCCACTTAGGGACAGCCTGTCTGAAGATGTCATTTGGCTTTTTGGCCACTTGAGTACAGGCTGGGGGGGGGGAGATTCTAGGATGAAAAGAATCCCCATACTGCCAGGCTTCCCCATCCACCTCTTGACCACCCTTCACCCTTCTCTTCTCTCTGAGCTACAAAGCTCAGCTGGGTTCAGATTATACTTATACAACTCCCAACCCCAACCAAATTAGAACATGGGCCGGGGGTATGAGGGGTGTGTGGGATGACTGAGACAGACATGTATCAAATATTTATTGAACACAGAGATGAAGCCGGAAGCTTAGTAGCACAGGGGCTGTGGTAAGGTATAGGGACCACATGGAGATCAGATGAGTACAGAGTGTGACACAGCACATTGGACAAACAGTATCTGTCAGGCCTTTATACCTGGCAGTGCCAGTAGTTTGGTCCACAAAGCTTCTAATCTTCACAGCCTGCATTGAGCATCTTTCTCTCTTTGGCCCCTTCACCAGGTTGGGGTCTGGTCTTGCTCCCCAGGGAAGTGAATTTAGGGCCAGGGATTTGTCTAGGAGAACCTGTACCCTGGGAAGAGCCCAAGGTTGTGTCAGCCTCTGAAAAGCTGACTTTCCACTGAGCAAGAGGTAGAGAAATGAAGGGACCTATAGGAATGTGAAGGAAAGCTCGTCTGCAGAGCCCAAGAGGGATAGGAGAGAGGGAAGGAGGTTGGCGTCTCCTTTGCCCATAAATCTGGGAGAGGGGCCAAGGGAGAAGAGCTGGAATGCCCACTTCTTGCTCCTCTCACACGGGCTGGATCCCAGGATGAAGGAGGCAGTCAGTCATAGATGGAACTCAATTCAGGCCAGTCCCATAGGACCAAAATGTACACCTTTCAAGGGGCATCTGGGAGCATCAGCTGTGTCCTAAGATTAGGACTGCACCCCTGTCCCAGTCCACCCTGGCCTGAGAGGAGGTGGTGAGGCCGGGACACAGAACCCTCTCTGATCTCTGCCCCCAAAGTCTCCTCCAAGCCCCGATGGGATGCAGCAGACAGGGTCCAAGATTTGACCTCCAGCTCCTCCCCAAGTCTGGCTATGCCTCCTCCTTCAATTTGTCTTCTTGGCTGGCTTCCTATTCTTCAGCATTACGTAAGTGATGAACAGCCCCACAAACAGCAACAGGACCAGGCCAGCTGCTAGGGGAATGATAATGGCATACTCTCTGGGCAGGAAGTACGTGTGGATGATGTGGTCACTGTCTATGAAAGGCAGAATGATCACCCAGATTGTGTAGTAGATGAAGATCACAAGACTGAAGACCACCAGGCCAAACCCAACCACCTGGTCTGTTCTTGTGGCCATTTCTGCCGAATCTTAGTTGGAGCAGGACACATGCCACTTCCGGACTAATCCATTTTCTTCCCCCCGGGGGCAGAATCACGGTGCATCCTGGGAGTCGTAGTTCCTCGCCTTCCCATCCCGGAGATCGGCCACGTCTCTCTGAACGCCGAAGCTCCACCTCCCAGAGCTGGCG

>UoN.Pci.T.30228_UoN.Pci.T.30228.1

CCCCCCCCAGCCCGGGGCTCCGGGTAAAGTTTCATGCTCGGCACGTGACTCGCCATGGCCGCTGTTCTCTCCCCGCGCCCCTGAGCCCAGCAGCGCGGCCCCTGTCCCGGACTCTCCCCCTCCGAGGTCCCAGACCCGAGCCCTCACCCGAGGACTAGGAGGAGGGAGGGAGGGGCCCGGGCGCGCCGCGCTCCGCTCGGCAGACAGACTGACGGACCAGCTCCTCCGGCTGCCAGCATCAGAAGACATGGTTTCCTCAGCAGCACCAGCATCATCAGAGCTCATCTAGGTGAGGATATTCTACCCAGCAGCCAGCCGTGGTGGGTGCCATTCAGGAAGCTGCCTCAGCTTTCCAAGCCTCATTCCAGCTTATCCTAGCCACCGCCGACCTGAGCAGCGCCCTCCCTTCCCTTTCCCACCTTGCCCCTGGTTTGGGCGGTGGCTCCAGAAGGCCAAGATCACCCTGTTGGGCACTGCCCTCTTCAGCTTCAGGACAGGAAGAGGGGGTGGGTTGATCACCCACAACCCCTCCCAACTCCAGGCCCCAATGAGCTGAGAAGGGGCAGCCAGAAGCCCCTCAAACCCTCCCAGCTGGACTTTCTGTCTATATCTCTGACTCTCATCGGGGCATGGCCAGCAATAGCAGCTCATGCCCAACACCTGGGGGTGGGCACCTTAACGGGTACCCGGTGCCTCACTATGCCTTCTTCTTCCCTCCCATGCTGGGTGGACTTTCTCCACCTGGCACCCTGACAAGTATCCAGCACCAGCTTCCTGTCAGTGGATATAGCACTCCATCACCAGCCACCATTGAAACTCAGAGCAGCAGCTCGGAGGAGATTGTGCCCAGCCCCCCTTCGCCACCTCCTCTCCCCCGAATCTACAAGCCGTGTTTTGTCTGCCAGGACAAGTCCTCGGGGTACCACTATGGGGTCAGTGCCTGTGAAGGCTGTAAGGGCTTCTTTCGTCGAAGCATCCAGAAAAACATGGTATATACATGTCATCGGGACAAGAACTGCGTCATCAACAAGGTGACGAGAAATCGCTGTCAGTACTGCCGGCTACAGAAATGCTTTGAAGTGGGCATGTCCAAGGAGTCTGTGAGGAATGATCGGAATAAGAAAAAGAAAGAGATGCCCAAAGCAGAGTGTTCAGAGAGCTACACGCTGACCCCCGAGGTGGAGGAGTTGATTGAGAAGGTTCGAAAAGCCCATCAGGAAACCTTTCCTGCCCTCTGCCAGCTTGGGAAATACACTACGAACAACAGCTCGGAACAGAGGGTCTCCTTAGATATTGACCTCTGGGACAAGTTCAGTGAACTCTCCACCAAATGTATCATCAAGACTGTGGAGTTTGCCAAGCAGCTTCCCGGCTTCACCACTCTTACCATTGCTGATCAGATCACCCTTCTCAAGGCTGCCTGCTTGGACATCCTGATTCTTCGGATCTGCACGAGGTACACACCAGAACAGGACACAATGACCTTCTCAGATGGGTTGACCCTGAATCGGACGCAGATGCACAATGCCGGTTTTGGGCCCCTCACCGACTTGGTCTTTGCCTTCGCCAACCAGCTGCTGCCACTGGAGATGGATGATGCTGAGACTGGGCTCCTCAGTGCCATCTGTCTCATCTGTGGAGACCGGCAGGACTTGGAGCAGCCAGACAAAGTGGACACTCTGCAGGAGCCACTTTTGGAAGCCCTCAAGGTCTATGTGAGGAAACGGCGGCCCAGTCGGCCTCACATGTTCCCTAAAATGCTGATGAAGATCACGGACCTTCGAAGCATTAGCGCCAAGGGAGCTGAGCGAGTGATCACATTGAAGATGGAGATCCCAGGCTCGATGCCACCCCTCATCCAGGAGATGCTAGAGAATTCAGAGGGACTGGACACTCTGAGTGGACAGCAGGGGGGTGGGGGGAGGGAGGGGGATGGGAGCAGCCTGGCCCCACCACCGGGCAGCTGCAGCCCCAGCCTCTCCCCCAGCTCCAACAGAAGCAGCCCAGCCACCCATTCTCCCTGACTGAACCCCAGCTCTCCCCCCTGCGGAAACACGGACACAGCCCTCACCCCACAGCCTGGCTTTCTCTGCCTTCAAAAAGAAACCACCATGAAACACATCTCCCCTCTCCCTTCACCCTCCCCCCCAGGACTGAAGGAGGTCTGTAAACCTCGTGGGGGCCTGGGGTCTGGATGGGAGGATGGAAGAGGTGGGAAGGATGGAACCGAGTCTCCTCCCACTCCATCTCTGCGCTGGACTTAACTGCAGCCTGAACTGGTTATCATCCATTATCCTGGATGGACCTCCATGGCACGGTGGGGGTTGGGGCAAGGGACCGAGGGCTGAAGGGTGCCCTCTTTCCAAATCCCAGCCCTGGGCCCAAGCAGTGAGACCCTGCCAGGATGTGGTGGGAGAGGGTGGAGAGAATGCCATGCTCATAGAAGCGCCTCTTTACTCACCACCCCCTACTCTCCACTCCACCCCCCAGCCTCATGTTCATCACCAGCAAATTCCGGGGGCTTTCAGCCCCACCATTCTCAAAACTAGCAAGCCATCCACCCCCTCACCGAGCTGGGGGAAAGTCTGGGGGCAGAGGTGGGGGGGGTTAGAGTCCCCCATGCTGTGGAATCCCCTCAGGTTGGTGATGGGGGGCAGGGCTTCTCCCCTGTACATACTCCGCTGAATTCTGCCCTGCCCCCCTTCCCTTTCCTCCCAGATATTACCACCTCCCCCGCTGGTTTTGTTTTTATTTTAATTTTTTTTGTTTTTATTTTTTTTTTAAATAAGAAGTTTCATTTTAAGCACATTTGTACTGAAGGAATCTGTGCTGTGCATTGGGGGGG

>UoN.Pci.T.28072_UoN.Pci.T.28072.1

TAGATTGAAGCATATATAGTGATAGATTTTTTAAAAAATCAAAATCTGAAAAGTAAACTTTATTGACATCAATAATAAAGTTGACTAAGTTTTCAACATTTAGGACTTTCACATTCTCGCTGTTTATTCAATTATTCAAACATCCTTTTTTCTAAATATACTTTGCTGATTAAGAAATTAAAAACCTTGTGTACATTAGAACATATTCAACAATTATACAACAGATTGCGCTCTCCAGTCAAGTCTTCTTAGAGGGGGAAAAAAACCAACTTTGTTCATCACAACCAGGACAGGATAAAATACCACTCTCCTTGGGAGAGATTTGTACTCCTTAATTGAAGTGCTGGTGCCTTCCTTCTTTCTGATGTTGTTTTAAGCCCAGGTAGTAACCAGTATGATAGCCACTCATGTACCAGGCTATTAACATACTTCCCAAAGCATCAGCATCTTCAGGAGAATCCGGAGACATGGGAGGTGGTGGAGGAATCAATGGTGGTCCTGAAGGAAATGGTGGGGGCCAGCCTGTGAGGAAGGGTGGTGGGCCACTGAATTTCAGTCCAGGCTTGCCTAGCCCTGGCACTGGAAGAGGAAGTGGTGGAGGAGGAGGGAAATAAGAATTCCAATGAGCAGCTTTGGATTTGATACGATTCTGTTTGCTTCCAGGTGATCTGGAGGATTTTTCACTTTCATCTGTTGAATACTGACTTTGACTTTCATTTTCATTTTCATGTATAGGTTCTTCATCACGAACTCCTTCGCTGTTTGGAAGAAGAAGATCTGACAAATTCTGTTCTTCTTTATTTCCATAGCCAGTATAAACCACAACACATGTCCCTCTCTTCAGGTCAGTTGATGCAATAGTAGCTGGATATATGTTACCATCTTCAGACCAAACTGCACAACAAGCATCACCAACTTTCCACTGTTTCAATGGGGCTGGGTTACTTTTTTTCTTGTTTCTGTTCTTAATATTTTTTCTTTTTGGTCCAGCATTCTTTTGTTTGTCTGAAGTTTCAGAAATTTCACCATTCTTTAAAGCATTCTTAAATGATGCCACAGCTTTGTCATATGCTTTTATCAAGGCTGTATCATCCCAAATATCAGAATCATCACTCTGCCCGGTACCTCTGCGGAACAATACCGGTTCCTCGTGTTCTCTGACGCCGCCGCCTAACGCAGCCATGGCGAGCGGAACAACCCACCCTAAGCGCAGGCTCCAAGGCTCCGCTCCGCCCGGCCCCGCCTCTGGGAG

>UoN.Pci.T.32059_UoN.Pci.T.32059.1

GAGGAGGACTGCACTGAGGAGCTCTTTGACTTCCTGCATGCCCGTGACCACTGCGTGAGTCACTGCTTCTGGCCCGAGGACTAGGCAGAGCAGGCCGCTTCTCTGTGGCCCCATCCCCTCTCTGGGCCTTGGTTTCTTCTTCTATAAAAGGAGGTTTGACTCAGTAGCCTCTGAGGTCCCTAGAACTAAGGACTTCCCTGGAATTCTGGGTAACCAGAAGCAGCTGGAGCAAGTGGGAGCTCAATAGACACCTACCTAGGGTTGGCCTAGGGAGCAGAGCACCCAGATCACCCCTGGTGGGTCCTAGGTGCTAGGTCTTAGGCCTTTGCTGTCAGCAGCTTCGGGGGCAATGCTGCTTGCTTGTGTTTTCCTCTGGTCTGGGCCCTGGTAATGCTCAGTGCTGCTGGGCGGTTCAGTGGTTAGAGCACTGGACTCAAAGCCAGGAAGACCTGCCTGAGTTCAGATATGGCCTCAAGACTTCCACCTGTGTGGCCTGGGCAAGTCCCCTGCCCTCTTTGAGCCTCTGAGTTCTTACCTGTATAACGGGGGCCCACATTACAACCTCACATTAGCTGCCATTGTGGGGGGGGGCAGGTATTCCCACCATGTGTTTGGCGCCTCCTTTGAGGCACTGTTCGGCCTTTAATATGGAATTAAATGGACCCCCGAGGGATCTTTGCTCACCTTGCTTTTCCTTCTCCCAGGTGGCCCACACACTCTTTGAAAGCGTGAAATAAATGTGTGTGGCTGTGCCAGCTGCTCCAG

>UoN.Pci.T.31824_UoN.Pci.T.31824.1

CATGATCCTAGGCAGCTGGTAAGAAATCACCAGTTTATTGAATGAAAAATCCAACAGCAACTCCCCCCATACCATGGCCCCAGCCATGAGGACAGCTGAGCCCAGGCAAGGTACTTTGGACAGCCGGACTGAGTCACAGGACAGGAAACTGAGCTAGGATGACACCTTTCAGAACAAAGTTCTGTCCAAACCTCTGGGGCAGGGAAGAGGACCTCAGTGGACAGAACACCGGAGGACTGAGCTGCGTTAAAATCCCCCGAATTTGGGGTAGGGGAGGGGGAAGCCAGGGCTCAGACATCTGTGTCCATGAGGAGGAGGAGAGAATGAGGCTGGAGGGGAGGGATCCTGGCAGGGTTTCGGCCCCATTAGGGCGGGAAGCTCACAAAGGGAGGAGGGGAGAAAACACAGCTGCAAGGTGGGCAGATGGAGTGCTGGAGGAGCCGGAGTGCACAGGGCCTGGAGTACATGGGTGGGCAGGGTGGGTCATGGGAGATAGAAGCCAATAAGCGCTCAAGGACACACGCACAACATGACACTTCTGGGGATGCTGGGCAGGGCCTGGGGGAGGGGGGCAGGAGATGGGCAGGCACAGCCACCCTTCCTGCCTCCATCCCAGTGGGCTCAGGCTGACCTTGGGCCTCCTTGGGGAAAGCCAAGTTGACTTGAGGCAGAGATGACATTTGTGGGAAGGGGACGGCAGTGACTAACTCCTGACAAACCGCTGGGTACTCACAGGAGACTGTAGCCTGGAAGGAGGGGGACAAGAAACTTTGGGAGACAGGGGCCTAAATGCAGAGAGGCAGCGGCCAGCAACGCTTTGTCCCCTGGGTGGACTTGGGCTCCAGGGGCTGGGGGGTGGGGCTCCCTTGGAAGAGTTGGCTTAAATTCCAGTGATGAGGCTCAAGGCCCAAGTGAGGAGGTGGGGAGGAGAGGTACAGGGGAGGGGGGAGTGACCAGCTTCCCCAGTGGCAGTGTCCCTCAGGCCCCAGGCCTGCTGGCATCCCCCTCCCCCAGGAGGTACCCAGACCAGCAGATGACACGAGGGAGAACGGCGGAGTCCTCGGCCAGGCAGCGGGGCGGTCAGGTGGACTTGCTACCACTGAACAGGCCCACTGGGAAATGCTTGACGTGGGTGGGCCACTGAGCAGCCAGCTGGAAAAACTTGATGTCACTCCACTCCACGCCGTCAATGGACACCCTCAACGTGGTCTGCTGCTGCTTGGCTGAGCAGATGAGGCGGCTGATGCCCTCGATGACCTGGCTCTTAGAATCCACCTCCTTCTCTCGAGGCTTCTTGCTCAGGAAGATGGTGGGCACTTTCTTGTTCTTCTCCTTGGTGACCACGGTCATGGCCATGGTGCCGGAGAGCTGCGCCTCGTTGCCGTGGGGCAGCCGGGACACCTGCACCGAGCGGAAGACGCTCTTGAGGGTGTTCTTGGCGCTGGCGTCCCTCTTCTCGCCTTCCCGCCGCCGGTCCCCGGGGTGGGCCAGCCAGTAGTCGACCTGCAGGCCGATGACGTCCCCATAAGGGCTGTTGGGACTCCCAACCACAGCCAGCACGCTGCTCATGGACGGGGAAGAAGGGGGCGTGGCCGAGGCATCCCGACTCGGGCCAGAGCCGGAAGGCGGGGACGTGGAGGGGACAGTGAGGCCGACCGCAGGCACGTCGTCACCGTCACCTGCTGTGAGAGGGGAGTCTTCCACCAGGCCCACTTTGACCACCCCAATGAAGGGAATAAACTTCTGGTAGGAATCTTCATCTTGGAATTTGTGTTTGCAGGTCAGCATGGCCTCCGCCACGGGCAGCTGGTGTGTCACGCCGGCCCCGCTCACGTACTGCATCACACGGCCCGCCACATCCAGCGGCTCTGAAACTGGGGGCTCCGAGCGGCTGAATAAGTCCCTCCAGGCCGGGTCGAGGAAGGTGCTGCTGTACCTGCTGTCCACCGAGCCGATGTACTTGGCGACAGGGTGAGAGCCCAGGGGGACGATGAGGAAGCGCATGTAGCCCAGCCAGTCGGACGTCTTGTTGGCCAAGGCCTTGACAAAGAACCGGAGGATGGAGCTCAGGTAGCTCTGCCCGCCCACAGCCACCACCTTCACAGGCCTCGGCATGGATGAGTTGCAATTGCAGTATCTCTGGATTCGGGTGAGCAGGGCCGAGAGCACGGCTTGGACTTCCACGGTGGAACACGTGCACACAACGGGCCTCCGCTGGTCCTGAAGCAGCTCTGCCACATACTGGCCCTGCCAGTCAGCGGTGTTCACGAGGATGACGTTTTCTGGGAGGGCCGCATCAGACACCAGGATCTGATTCAGCTGGTCATACACCACCTTTCTTGGGATCTGGGTGCTGTGGCCGAGGTCAGGAGACCGCTCGCTGTCCGAACTGTTGGTCCTCTCGCTCAGAGGCTTGGAGAGCTGCCGCTCCCTCAGGGGGGTGCTCCTCTTCTGCCTGGGGGTGTGTACCCCGTCCATTCTGCTGGGGGAGGCGGAGCCCTGGAGATCCGCCTTGCCGGACTTCATGGGCGTTTTCACTTTCTCTGGCACAACCAGGGTGGTGCTGCTGTCTCCAAACATGTCCTGGTCGCTGATGTCCAGAGGATCGTTCTCGGTCAGACTGTCATCTGGGTTTTTGATCCAAGAAGTAGCTTTGACTCTGTCCAGAGCAGTCAGTTCCATCGGGCTGGTGGCGTCCTTTCCCAGGCTGCCTTTACTGTTGAGGCTGCCAATCTCTGTCTGGGAACTGGACTGAGACATTCCCTCAAAGAAAGGCCTATGAGGAAGGAGGAGAGCCAGCATCACTGATGGGGACGGTTTCACAGAAGGGCCTCAGGGGCATCTGCCTACAGGGAACAATGCGGCCAGGCCCCACGGGAGGAGCAGGTGGGGGGCCCAGCTCTTCAGTGCCACTCTACGTTCCTGCCACCCATGGCTGTGAGCAGGACTGGAAGGTAATGGAAGGGCGGGTGAGGGGAGGCTTATTTGGGGCCTTCAGTGAGGACGGGCGCCTGTTCTCGGTCCTAAGGGGCACGTGCACTCTGTTCCCAGGATGCCGGAGCTCCGGCGAGTCCCTGAGGCCTGGGGGTCAGGGGCGGGGGCGGGGGCCCACTTGAGCTTGGGCTTGGGGGTGCTCAGGACGCTCTCCGTCTCCTCCATCTCGGGCCCACTGTCACTGGGGTTGTACATCTCCAGGCTGTCATACAGCTCGTCCAGGTCTTCTTCTACTTCCCGGATCTGCTCCCGTGATACGTGCTCCAGCCCAAAGCCCACCTGTGGGACAAGATCCTAGGAGTCAAGGCCTCCACCTGCTTCAGCGAGGGGGTGCCCAACAGGCCCGCTGAGGGGCAGCAGAGGGTGCAAGGTGGATCGGGCTCCTTGCCACTCAAGGCCTGGGCCACCTTACTAAGCTGCTTCATCTCTGGGCCTCAGTTTCCACACCTATAAAAAGGAGGGCGTCAAGCCAGACGTATCGCGCGGTTCCTCCCAGCTTGAGGCCTGTGAGCCTTGCTGGACAACGCTGTTCCACCCCTGAGAGGTCACCTAATCCAACCCTTTTCTTTTAGAGGAGGAAACTGAGGCAATTTCTTCCTCTGGGTACAAATCCCTGGGCACGGGCATTAGTAACGAATCTCAGGGCAATCGTTGGTGCCCTTTCCTGACTCTTTTCAGGGAAGCTCCCTGACCCCTCAGAGGGCGGAGGGCCTCGAAACATGCAGGTGTCCTTCCCAAGGGAGCCCCAGAGGCTTTTGAGAGGTGTCGCTCTTATGGCTGGTTGGCTTGCCGGGCCACCTGCCCGCCCTCCCCCCACCCACATCCTGGGGCAGCTGGACACGGAGCCTCACGGCCCCCTAGCACACCACGGGGAGCGAGCGGGGCAACCAGACCAGGCATCTGAACTCTGACGAGGGCTGGCAAGGAGGGCCCTTCCTGTGGCACGCTCACGAGAGAGGGGCCAGGGAGGCAGAAGCTCAAGTGATCCCACTGATTCTGCCCCTTCTCACCAGCCCTCGGAGGAGATCAGAGCCCTCGTTTATCTGTGACTATCGGGTCTTGAGAGAGGATGGCCAGTGAGAACCTGAGTAACTCCTGCTGGCATGACTGCAAATCTCCCCACAATCCCTTGGGAATCCATCCTGCCCAGCCCCAGGCAGGGGTCAGGACGGGGGCCCTGGTACCTCATCAGAGACTTTAAACCTCTTCAGCAGGGCCACGAACTTCTGCTTGATGTTGGGTTGCCTTGTGATGGCAGAGGCTGAGGTCAGTTTCCTCCGGGTCTTCTTCACCTTCCGTAGATCCTCATCTTCATAGAACAAGTCCTGGCCGTGCAAAGGGTCATCGCTCCCCTCCTGCTCTGATGAGAAGCTTTCTTCCTCCTCTTCCGAGTAGTTGTCGATATCAGGAGAACGGTCAGACAGCTTGGCCTTGATCCCTTCCTGGTCGATGGGCTGGCTCGACAGCGAGTAGATCTTGATTTCGGCCACAGGGATGGAGACGTCCTTCACGCTGCTGTGCAGGCCCAGCACGAGGGCGCCTTCACTGGGATGCTGCATGACCTCCGCCATGTTGATCAGCCCCACGGCCAGCGTCTTGTAGCCGAGGATTGTCCGGTTCTTGTAGCGCTTTCTCCTCTGCAGCATGATCTGAAGCTTGTTGGCATCGCGCTTAAGGAAGTGAGGGTACTGGAGGGAGAAGGTCAGCTGAAGCTCCGTCTCTGCCAGGCCGCTGGCTGGAAGGAGGATCTCATTGGACCGAAGGATCCTTTTTGAGCCCTGCAGTTTCACCGCAATGACCACGGAGTTCAGGTCTTTGTCCATTTCCTTCAGCATGACAAGTTTCTTCAGGGTCAGGCTGAACAGCCTGGGCACGCAGCTGGCCGAGCTCCGGTCCACCTCCCAGGTGGCGTACAGGTTCATCTGCACCGGGGCGGGCGCGGGGCTGGCGGCGGGGGGCCGCCCGGGGGCGGCGGCCGGGCCCGG

>UoN.Pci.T.32922_UoN.Pci.T.32922.1

GAGAGAGAGAGGGGCAGAGAGAGAGCGAGAGAGAGAGCCGTGGAAATGAAAGTAAAGGGCTAAGCGGCCACATGGAGGGAGCTGCCTGGGCAGCAGTTCCAGGAGCAAAATGCTTCCTTCGATAATTAAATAGCATTTACTCTTATTATTACTAATAATAATATAATAATAATATCTCTAGTAATATTATACCACTTATCAAGGCCCGTTGTAGGCACGGTGGGGGGACTGCCAAGCAAGGAAAGAGACTAATGGAGAGGCAGAAAAGAAAAGAAGAAATAGAGAAAGGACTTCAATTCATCCAGTCCACGATATCACTAAAGCAAGAAGATTATGAGGCCTTTCTCCACAAGTTGGTTTGGAACCTGTTTGCTGAGGGGAATGATCTGTTCCGGGAGAAGGATTTCAAGCAGGCATTGGTGCAGTATGTGGAGGGGCTGAACGTGGCTGACTATGCTGCCTCGGACGAGGTGACGCTGCCCCGAGAACTCCTCTGTAAGCTGCACGTCAACCGGGCCGCCTGCTATTTCAACATGGGCCTGTATGAGAAGGCACTGGAAGACAGTGAGAAGGCCCTGGGACTGGACCCAGAGAACATCCGAGCTTTGTTCCGGAAAGCTAGTTCCTTGAATGAGCTGGGGCGCCATAAGGAAGCCTACGAGTGCAGTAAAACGTGCTCCATCTCTCTTCCCCACGATGAAAGTGTCACTCAGCTCAGTCGGGAGCTGGCCCAGAAGCTGAGGTTGCCCTGCCGAAAAGCATATAAGAGACCTCAGGAATTGGAAACCTTCTCGCTGCTTAGTAACGGCACCTCGGCTCCCTTAGCAGATCAGGGAACATCCAATGGGCTTGGATCTATAGATGACATCGAAACAGACTGCTCCATGGATCTAAGGTGTCTCACAGCCCCAGCAACCACCTCCATCCCTGGCTGTGACATCACCCTCCTTTCGGACCCCGAGGCCAAGGGCTCCACCACAACCCTTTTCCCTCCCACCACCGACCTGCTGGCCCCTCCAGATGGGGCTGGACCTGAGAGCATGGAGGACTTCTCTGATGGCGATGTCTTTGGGCCAGAACTGGACTCACTCCTGGATTCATTGTCTCTGGTCCAAGGCTCCATTCCAGCGACTGTGCCCAGTGAAATGCCCCAGCGGATCCCTGTGTTCCCAGGTGGGGCGCCCCTATTGCCCCCTGTGGTGAGTGGCACCATCCCTGTCTCCAATCCACTGCCCCCCGCATCCTTTGGCCTCGTCATGGACCCCACCAAGAAGATGGCCTCCTCCATGCTGGAAGCCTTTGACTCTCCGGTTTCCTCACTGGACCCTTTGGATCCTCTGGACTTGCTTCCGTACACAGATGCCCACCTCGAAGCCCTGGACAGCTTGGGGCCTGTCCGGGGCTCCCTGGACACACTGGATTCATTCCCAGTAGAGGACACCTCCTCTCAGGACCTACGACCACCCAGCAGCACCCAGAAGCCGGCCCCTGCACCGGCCAGCACAAGTCACTCCCCTCTGCCCAAGGTGGCTGATCTGCTGCCCCAGCAAGAGTCCGCCATGCCCAATACCGCCCTGCTTGTCAAGAACCCCCTGGCTTCTACCCACCTCTTCAAGCAGGCCTGCCACCTCTGCTATCCCAAAACAGGTCCCAAGGCTGGTGACTACAGCTACCGGGAAGACCTAGAGCACAAGTGTAAAAGGGACATTCTGCTGGGGAGGATCCGAAACTCCGAGGACAAGACGTGGAAGCGGATCCGGCCCCGGCCCACCAAGACCAGCTTCATAGGCTCCTACTATCTGTGCAAAGACATGCTTAACAAGCAGGACTGTAAGTACGGGGATAACTGCACCTTCGCCTACCATCAGGAGGAGATCGACGTGTGGACTGAGGAGAGGAAGGGTACCCTCAACAGAGACCTGCTCTTCGACCCCCTCGGGGGAGTCAAGCGGGGCAGCCTCACCATCGCCAAGCTTCTCAAAGAGCACCAGGGAATCTTCACCTTCCTCTGTGAGATTTGCTTCGACAGCAAGCCCCGGATCATCAGCAAGGGGGCCAAGGACTCTCCTACCGTCTGCTCCAACCTGGCCGCCAAGCACAGCTTCCATGACAACAAGTGTCTGGTTCACATCGTGCGCTCCACTGCCCTGAAATACTCAAAGATCCGCCAGTTCCAAGAGCACTTCCAGTTCGATGTGTGCCGCCACGAGGTACGCTATGGCTGTCTTCGAGAGGACAGCTGCCACTTTGCCCACAGCTTCATTGAACTCAAGGTCTGGCTGCTGCAGCAATACTCAGGAATGACCCACGAGGACATCGTCCAAGAGTCCAAGAAGTGCTGGCAGCAGATGGAGGCCCATGCAGGCAAAGCTGCCAACAGTTTGACCAGCTCTCGGGTCCCCCCACCCAGTACCTTTGACCTGCAAATGAAGTTTGTGTGTGGCCAATGCTGGAGGAATGGACAGGTGGTGGAGCCCGACAAGGACCTCAAATACTGCAGCGCCAAAGCCCGACACTGCTGGACCAAGGAGCGGCGTGTCCTGCTGGTGATGTCCAAAGTCAAGAGGAAGTGGGTGTCTGTCCGTCCGCTCCCCTCTATCCGCAGCTTCCCTCAACAGTATGATCTATGCATCCATGCCCAGAATGGCCGGAAGTGTCAGTACGTGGGGAACTGTTCCTTTGCCCACAGCCCAGAGGAGAGAGACATGTGGACTTTCATGAAAGAGAATAAGATCCTAGACATGCAGCAGACCTATGACATGTGGCTGAAGAAACACAACCCTGGGAAGCCTGGGGAAGGCACACAGGTCACCTCTCGGGAAGGAGAGAAGCAGATCCAGATGCCCACTGACTATGCGGACATCATGGTAATGACGCCTTCCCAGCCCCACTTTACAGCCTTGGTAGCCGTGGCTTACGTGGAGAGCTGGCGTGTTTGACTCGTAGGTGACCCCCAGCCAGGAGGGAACCAGAATGTGTTGGGTAACCCGAGTAGAAGAATCCAGCAAGGTTAACTAGAATGGCGACAATGATAAAGTCCTGTACCTTGGCTTTCCCAAAACAAGAAACAAAAGCCAGTTGTACAAATGCCGAATAGGGGGTATGGGACAAGAGAAAGACTTAACTTCTCAATAACATGGCAGCCCCCCCCCCAACCCTAATTCAGTGATAGGGCAATGGTCAGAGCAAGAAGCAGGATAGTCCTAATGCCCTCTGGCCCGGGCAGACCACATCTGGATGGAAAGCTAGCCCCTATCCTCTGGATATGGTGAGGAATGTCAAGGTCTTTCCTCATATGAAACAACAGAAGAAACTTACGGTGGTTAGACTGCAAAGAAAGGATTTAGGGCAAGGGAACGCCCTGTGACCCCACCTGCAAATAACCTGCCATTGGCTTTACCTTTATTTCCGGGGGCAGAGCTAGTGGGGAACAGTTACACAGAGCCTTTTTCTGAGAACAGAATTTCCTAAACATTAGAAATGTCCCGCAGTGGAGTGGGCTGTCTTCCAAGGTGATGAGCTCCCCATCTCTAAAGAAATTCCAGCAGAAGCTGGATAGCTGCTACGAGGAGGGGTGTTTTGTGGAGGAGATTCACTCCTTAGGCTTAATGTATTGGGGTTAGACTAGATATCCCTGAGGCCCTTCCCAGCTCTTCAATTTTGTGATTCCTCTCCCCTACCCTATTCCTCTCTCTCTTTTAAAAAAGGTTTCGTTTTCTTGACTTTCCTTTTAAAAACATCATTGTTGTTCCTAATGAGTTCCCCACCTACCCCCCGACATGAAAGTCCTTCCTCGTAACAAATAAATACAGTTAAGAAGAACAAGTCAACACAGTGGCCACATCTGAAAACATACGTCTCATTCCGCACTCGGAATCTACCACCCCTCAATCCCCTGAAGTCACAATTAGTTGTGGCTAGGTCCCAATTAATGCAAATAATGAATCCCTTGAAATGGTCTTCTCTATTCAGATTCACATTATTCAGATTTCTGGTTCTGTAAAACATGGTCATTGGTTCCATTCTAGTGGAAAGGAGCCGGACAAATCCCAGTAACGGGACCGCTTCGTGGGATTCGCCACTAGCCCTAGTTTGACCTAACTGAACTGCATTGATCAGAGCTTTGAAGTCTTTTAATGGCGGTTTCCTTGTCATGGTTGGGGGTGTCATGTGGTGTCCTGCTCTCCTCTCCCCCCACATCTCTCCCATCCCATTACCTGCTTTCTCCCACCAAGGTTACTCTCCACGTAGTTGTTCTGGGTTGGGATGCAACGGGACAAATGCTCTTAGTGGCTGAGCATGGGAGCTGGGGGCTGGGTGAAAGTGTTGGCAGCCAGAGGGCCAGGGTGGTTTTGGGGTGGGGGTGAGCACCTTTGTTTGTCACCTCTGCCGTGGGGCCCTCAGGGTGGAGAGGGAGAACTTTCAGGAACTGGCTTGGAGAAGGCTAGGAGGAAAGCAGAGGGGGCCAGCCTGGGGAAGGAGAGCCGCAGAGATGGGCCTTGGAGCCCATGGGAGTGCTCAGAAGGCTTGCCCAGCACCCTCCCCCCCCCCGCTCCCCACAGATGGGATTCCACTGCTGGCTTTGTGGCAAAAACAGCAACAGTAAGAAGCAGTGGCAGCAGCACATCCAGTCGGAGAAGCACAAAGAGAAGGTCTTCACATCAGATAGCGACTCCACCTGTTGGACCTTCCGCTTCCCCATGGGCGAGTTCCGGCTGTGTGAAAGGTTCCAGAAAAAAAAGGCTTGCCCAGATGGGGAGAAGTGTCGCTGTGCCCACGGGCAGGAGGAGCTGACTGAGTGGCTGGATCGGCGGGAGGTGCTGAAACAGAAGTTGGCCAAGGCTCGCAAGGATATGCTGCTCTGCCCAATGGATGACGACTTTGGCAAATACAACTTCCTATTGCAAGACAACGTATAGGGGGCCCCGGGGACGGGACTCTGGGGGTGGGACTTAGGGAATGAGAAGAAAGATGGGAGGATGAGATGAGATTCCCATGGCCTCCGCAGCAGCATCTTTGGGTCCCCTCTCTGTTAGCCATCCCTGCTGCAGGGGCCAAAGGGCAGGACGTCCTCAGCTCTTTGGCTCCCTGTGACTGGCGTGGCTTTCTTCCTTGTTCTCAGATGGGTGTCAGAAAAGCCAGGCTGGGGAAAGTGGAGGGGATGGAGGAGGACTTGGGAAGTAGATCTCTGGACAAACCTTTTCTCTCCCTGCATCTCTATAGCACGCCCACACACTTGGGGTCCCACTTCATTCCCCGCTGTGTGGGGCAGGAGTACACCCACATACGTGCACATAATCAGAATAATAATGATGACGATGATAACAATAGCTCACATTTCTATAGTGTTTTCTCACAATGACCCTGGGAGGTAGATAGCTCAAGTATTATCACCCCCATCTTACAGATGGGGAAACTGAGATTCAGAGAGGACAAAGTGGCTTGATCACAGTTACATGGCCAGTCAGTGTTTGGAGACAAGAGGGAACACACACACACACACACACACACACACACACTGCTCAGATCAGTTAGACATGTCCCAGGTCATTATTGTCATCCTGTGGTATTAGATAGATCTATTGTCTCCCAGAGTAGGGTGGAACAGGACAGACAGATGCTGTCTGGTGGCTTCAACCCAAAGTTTTGTGTTTTGTTTCCAAAAACCTGTGGTCACTGGCATCAGGGATCCCCCAGGGTTATCCTCTTGGGGGGTTGGCTGAATTGGTTTTTTCCCCGTTACTCTGGGCTCCTGATATCAGCCCTTCTCTCTGGGATGCAGGAGATGGGGACAGGGACACAGTCTTCCCCAGGGCACAGCTGGAGACCTCAAAGAGCACAGATACCCCATTTAGCTTTAAACCCTAAACTTCCTTAATGATCCACATGGTGGAGCTCAGTATTCCCTTCAGTGTCACTCCCCAGCTGCCAAAGGAGACCAGTTCTCTGGTCTTTCCCTCAGCCCTTTGTCCTTTTGGCTGGAGACACCCTTCACACCCTTCTTGTAGCTCTGTTTCCCTTCTGTGCCCTACCTGGGATCCACACAGCTGTGGAACTTATGGGTCTTGCAGGCCCTGATCTTAACCTGACTCTCTTGTCCTGTCTCTCTCCCTTCAGTGATCCAAGAGAAAAGTGGCAGATGAAGCATGGGGCAGGATGGGTGGGCGGTGATCCCAATCCAGGACATGTTAGGGGATCCCTCCAGAGAAAGGACCATCCATGTTGAACCTGGATTCTCCAGGTCCAGTAATCCCTGTACCAGATCCCAAACAGCCCACCAGCACCCCTAGATTATCAGCTGGGCCTGGTTTAGGGAAGTGAGGGAGGAAGGAGTGGAGATGGGAAGCCAGCCGAGGGCAGGCTTGTCCTCAGCAGCTCTTTCTTCCCAGGACATCTCCTTCCTAACCAGGGACAGCTTTGTCCAGAGAGTTCAATTAGATTTCAGTGGATATAGAATGGGAAGATAGACTGAAGTGGGAGGGAAGGGTGCTACTTATACTAAAGCCAATGGAGCTAAAAGTCTGAGTAACTCTTAGGCATGCCCCTTCCATTCCTGCACCTCACCCACAACTCTGATATTGATTAGGAATAGGGACCCTGCTGAGCATAAATCAGGGGGCACTACTCCTGCTACCCTCCTGGGTCACTTCTGACAGAAATAGCTCCGGTTTCTCTAGCTCCGTCAAAATCTATGAAGTGCTCCCAATGAGGGAAGTAGGCCATTTCACAGAGGAGAAAACTGAGGCTCAGAGAGGAGGAGTCACTTTCACCTGATCACACAGCTAGCAAACATGAAAGCTGGAACTCAGACCTGGGACTTTCCACTTTGGGCCCACAGGCACCTCTGCTACCATCCTGGGGACCATGGACTGCAGCACCAGGACCTAGGGAGAATGCTGCTCCCCAAACCCCCTGGGTTCTGAATCCAGCTCTTCCAAACTTGCTGTGGGACCTTAAATGAGTCACTTCACTGCTCTGGGCCTCAGTTTCCTCATCTGTAAACATGGGGGACCAGATGGTTTCTCAGGTCCCTTCCTGCTTTAAATCTATCATTCTGTGGTCCTAAGTAATTTCCCCTTTCTGGCCGTCAGTTTCCTCATCTCTAAAGTGAGAGGGGCTGAAATAGATGACTTCTGAGGTCCCTTCCAGCTCTACATCTATGATCCTATAGTCTGAGGTCCCTTCCCTTTTCTGGACCTCAGTTTCTCATCTGTAAAATAAGGGGCTGAACTAGGTAGCCTTTCACATCCCTTTGAGCTCTAAAACTATGGTCTTTCTGGCCAAGGGGAACTGCCCCCCCCATTGAAGTAGTGCACAAGGTCACCTAGTTTGTACCTACTCCCCTAATTTGACATACCCTGGGTTCAGAATGGATAGGGGGAAGGCTTCACCCAACTGTTCTGGTCCCATCCCCCACCTTAGTCCACCAGACTTCCCACTTCTTCACCTTTTATCTGACACCTCAAAATAGCATTTTTCTGTGCCCACCTCCCCCCAACCTTGTCCACCCCTGCCAGATTCAGGCATTGGTCCCCCAGACATTCCAAAAAGCCCCATACCACTGGATTGGGACCTTAGGCTTGGGGGCAAGGCATGGGGGGAGAGGAGGAAGGGCAGACTTTCTCTTCTTATCTCAAGTCTGTGTGGAAGTGTATGGCTCATGGGATACCTTTGTTCCTTAGGCCTCTCAGAAGGCAATGTTGATGACATAGAAACTGGAGCGGGTAGGGTCTTCCCTCTACCACCCACTCTTGGGACCCCCACCCTTTATTCTATGATCATGGCATGCACCCTGTCCTGTCCCCCTCCTCCCAGTGAACCAGGATGATGGAGCCTGGGGAGGAGAGAGGAATTAAAGGGTATTTGATTTGGGGGTCCACGGGGAGGGATTGCTGTGAACGTAGATACTTGTTTTCCCTGATATGATAACCTAACATGTGGTATTACTGTCTCTTGTCACCAGCCTTGTTTCCCTGTGTATATATGTCGGCCCCGTGATGCATATATACACAGGTATTAAATATATCACTCTATATAATATTATATATGTGTGTGGTATCCAAGGAATTGCTTGTAAGGAAGGTGAAGGATAAAGGATTTGGCTGGGAAATGCAGCAGGAGCCCTGGGTTTGACTTGGCTTCCCTAGCTGGGAGCAGGGTGAGTGGGGGCTGCTGCCCTTCACCTCAGATCAGCAAGGTGCCTGATGGTATATAGGAAGGTAGCATTGGAGGACATTGAACAAGAGAGACGTGGGTGGCGATGCCTGGCTCTGTCCAGCGTCTTCCTTCCCCACCCAGCTTGACGGAGGCAGAAAGAGCGGGCTCAGTGGAGAAACTGTGTTCTTAGCCAGAGGGGCAGGGCCCAGGTTAACTAGGGCTGCCAAACTTCCAAAACCTGTAGGTCCAACTCTGCCTGTCCAATCCAGAGAAGCTGTCCATGGGAGCTGTAGGGAGGGGCCCAGGTTGGGAGGCAGAGGATTCGAATTTGAATGCTGTCTCAACCATTTCTTATACGTGGCTAATAGCACAACCTCCCTGGGCCACTTTTCTTGGCTATTAGATGGAGGCTTGGCCTAGATGGTCTCTGAGGTGTCTCCCAGCTCTAGACCTACAATCGTGCATGGAGAAGAAAAGAAAATGGAGCTGAAGGAGGCCATGTCCTCCTGGGGCCAGGGAGGCCTCTTCCCTAGGGCGGGCTTCTGTGCCCCCCTGGAAAGCAGGTGGATGCACATCCGAGAGAAGAGATTGGAGAAGAAGGGGCTCTAAAGGGAGAAGAATAAGCGGGTAATTTGGAGGATGATGGAAAGGACTGCAGTGGTCTTCAGATTTAGACTCAAAATCCAAGCTCAGAGTTGGGACCAAAGCTGCCAGGCTGCTCATGGACAGGCGAGGATGAGACCAAGGGGAGACGCATGTATACATATATGTGTGTACATATAACTGCACGCATATATGTATATGTACATGCACACGTGTGTGTGGTCACACCCCCTCCCCCCTGCACGGACTGTGGGGGGAACGTGTCAGAAATGAAGGAAGAAGGGGGGGAATGTTGCATTTCACAGCCAGGTAATGATGGGGTGAATAGTCCTTTAAAATGTCTGTGTATTAAACATTTTAAGAATACCACACTTTAATATTAAATATTCATAAGGCCCTAGTATCTGGATAATATGGTAGATGTTTTAATAACAATTTTTTGGTCCTTCTTAAAAATAAAACAGACAAAACTTGCATCTTTTGGCCTTTGTACTAGAAAATAAAAGTGCACTTGAACCCTG

>UoN.Pci.T.32922_UoN.Pci.T.32922.2

GGCAGAGAGAGAGCGAGAGAGAGAGCCGTGGAAATGAAAGTAAAGGGCTAAGCGGCCACATGGAGGGAGCTGCCTGGGCAGCAGTTCCAGGAGCAAAATGCTTCCTTCGATAATTAAATAGCATTTACTCTTATTATTACTAATAATAATATAATAATAATATCTCTAGTAATATTATACCACTTATCAAGGCCCGTTGTAGGCACGGTGGGGGGACTGCCAAGCAAGGAAAGAGACTAATGGAGAGGCAGAAAAGAAAAGAAGAAATAGAGAAAGGACTTCAATTCATCCAGTCCACGATATCACTAAAGCAAGAAGATTATGAGGCCTTTCTCCACAAGTTGGTTTGGAACCTGTTTGCTGAGGGGAATGATCTGTTCCGGGAGAAGGATTTCAAGCAGGCATTGGTGCAGTATGTGGAGGGGCTGAACGTGGCTGACTATGCTGCCTCGGACGAGGTGACGCTGCCCCGAGAACTCCTCTGTAAGCTGCACGTCAACCGGGCCGCCTGCTATTTCAACATGGGCCTGTATGAGAAGGCACTGGAAGACAGTGAGAAGGCCCTGGGACTGGACCCAGAGAACATCCGAGCTTTGTTCCGGAAAGCTAGTTCCTTGAATGAGCTGGGGCGCCATAAGGAAGCCTACGAGTGCAGTAAAACGTGCTCCATCTCTCTTCCCCACGATGAAAGTGTCACTCAGCTCAGTCGGGAGCTGGCCCAGAAGCTGAGGTTGCCCTGCCGAAAAGCATATAAGAGACCTCAGGAATTGGAAACCTTCTCGCTGCTTAGTAACGGCACCTCGGCTCCCTTAGCAGATCAGGGAACATCCAATGGGCTTGGATCTATAGATGACATCGAAACAGACTGCTCCATGGATCTAAGGTGTCTCACAGCCCCAGCAACCACCTCCATCCCTGGCTGTGACATCACCCTCCTTTCGGACCCCGAGGCCAAGGGCTCCACCACAACCCTTTTCCCTCCCACCACCGACCTGCTGGCCCCTCCAGATGGGGCTGGACCTGAGAGCATGGAGGACTTCTCTGATGGCGATGTCTTTGGGCCAGAACTGGACTCACTCCTGGATTCATTGTCTCTGGTCCAAGGCTCCATTCCAGCGACTGTGCCCAGTGAAATGCCCCAGCGGATCCCTGTGTTCCCAGGTGGGGCGCCCCTATTGCCCCCTGTGGTGAGTGGCACCATCCCTGTCTCCAATCCACTGCCCCCCGCATCCTTTGGCCTCGTCATGGACCCCACCAAGAAGATGGCCTCCTCCATGCTGGAAGCCTTTGACTCTCCGGTTTCCTCACTGGACCCTTTGGATCCTCTGGACTTGCTTCCGTACACAGATGCCCACCTCGAAGCCCTGGACAGCTTGGGGCCTGTCCGGGGCTCCCTGGACACACTGGATTCATTCCCAGTAGAGGACACCTCCTCTCAGGACCTACGACCACCCAGCAGCACCCAGAAGCCGGCCCCTGCACCGGCCAGCACAAGTCACTCCCCTCTGCCCAAGGTGGCTGATCTGCTGCCCCAGCAAGAGTCCGCCATGCCCAATACCGCCCTGCTTGTCAAGAACCCCCTGGCTTCTACCCACCTCTTCAAGCAGGCCTGCCACCTCTGCTATCCCAAAACAGGTCCCAAGGCTGGTGACTACAGCTACCGGGAAGACCTAGAGCACAAGTGTAAAAGGGACATTCTGCTGGGGAGGATCCGAAACTCCGAGGACAAGACGTGGAAGCGGATCCGGCCCCGGCCCACCAAGACCAGCTTCATAGGCTCCTACTATCTGTGCAAAGACATGCTTAACAAGCAGGACTGTAAGTACGGGGATAACTGCACCTTCGCCTACCATCAGGAGGAGATCGACGTGTGGACTGAGGAGAGGAAGGGTACCCTCAACAGAGACCTGCTCTTCGACCCCCTCGGGGGAGTCAAGCGGGGCAGCCTCACCATCGCCAAGCTTCTCAAAGAGCACCAGGGAATCTTCACCTTCCTCTGTGAGATTTGCTTCGACAGCAAGCCCCGGATCATCAGCAAGGGGGCCAAGGACTCTCCTACCGTCTGCTCCAACCTGGCCGCCAAGCACAGCTTCCATGACAACAAGTGTCTGGTTCACATCGTGCGCTCCACTGCCCTGAAATACTCAAAGATCCGCCAGTTCCAAGAGCACTTCCAGTTCGATGTGTGCCGCCACGAGGTACGCTATGGCTGTCTTCGAGAGGACAGCTGCCACTTTGCCCACAGCTTCATTGAACTCAAGGTCTGGCTGCTGCAGCAATACTCAGGAATGACCCACGAGGACATCGTCCAAGAGTCCAAGAAGTGCTGGCAGCAGATGGAGGCCCATGCAGGCAAAGCTGCCAACAGTTTGACCAGCTCTCGGGTCCCCCCACCCAGTACCTTTGACCTGCAAATGAAGTTTGTGTGTGGCCAATGCTGGAGGAATGGACAGGTGGTGGAGCCCGACAAGGACCTCAAATACTGCAGCGCCAAAGCCCGACACTGCTGGACCAAGGAGCGGCGTGTCCTGCTGGTGATGTCCAAAGTCAAGAGGAAGTGGGTGTCTGTCCGTCCGCTCCCCTCTATCCGCAGCTTCCCTCAACAGTATGATCTATGCATCCATGCCCAGAATGGCCGGAAGTGTCAGTACGTGGGGAACTGTTCCTTTGCCCACAGCCCAGAGGAGAGAGACATGTGGACTTTCATGAAAGAGAATAAGATCCTAGACATGCAGCAGACCTATGACATGTGGCTGAAGAAACACAACCCTGGGAAGCCTGGGGAAGGCACACAGGTCACCTCTCGGGAAGGAGAGAAGCAGATCCAGATGCCCACTGACTATGCGGACATCATGGTAATGACGCCTTCCCAGCCCCACTTTACAGCCTTGGTAGCCGTGGCTTACGTGGAGAGCTGGCGTGTTTGACTCGTAGGTGACCCCCAGCCAGGAGGGAACCAGAATGTGTTGGGTAACCCGAGTAGAAGAATCCAGCAAGGTTAACTAGAATGGCGACAATGATAAAGTCCTGTACCTTGGCTTTCCCAAAACAAGAAACAAAAGCCAGTTGTACAAATGCCGAATAGGGGGTATGGGACAAGAGAAAGACTTAACTTCTCAATAACATGGCAGCCCCCCCCCCAACCCTAATTCAGTGATAGGGCAATGGTCAGAGCAAGAAGCAGGATAGTCCTAATGCCCTCTGGCCCGGGCAGACCACATCTGGATGGAAAGCTAGCCCCTATCCTCTGGATATGGTGAGGAATGTCAAGGTCTTTCCTCATATGAAACAACAGAAGAAACTTACGGTGGTTAGACTGCAAAGAAAGGATTTAGGGCAAGGGAACGCCCTGTGACCCCACCTGCAAATAACCTGCCATTGGCTTTACCTTTATTTCCGGGGGCAGAGCTAGTGGGGAACAGTTACACAGAGCCTTTTTCTGAGAACAGAATTTCCTAAACATTAGAAATGTCCCGCAGTGGAGTGGGCTGTCTTCCAAGGTGATGAGCTCCCCATCTCTAAAGAAATTCCAGCAGAAGCTGGATAGCTGCTACGAGGAGGGGTGTTTTGTGGAGGAGATTCACTCCTTAGGCTTAATGTATTGGGGTTAGACTAGATATCCCTGAGGCCCTTCCCAGCTCTTCAATTTTGTGATTCCTCTCCCCTACCCTATTCCTCTCTCTCTTTTAAAAAAGGTTTCGTTTTCTTGACTTTCCTTTTAAAAACATCATTGTTGTTCCTAATGAGTTCCCCACCTACCCCCCGACATGAAAGTCCTTCCTCGTAACAAATAAATACAGTTAAGAAGAACAAGTCAACACAGTGGCCACATCTGAAAACATACGTCTCATTCCGCACTCGGAATCTACCACCCCTCAATCCCCTGAAGTCACAATTAGTTGTGGCTAGGTCCCAATTAATGCAAATAATGAATCCCTTGAAATGGTCTTCTCTATTCAGATTCACATTATTCAGATTTCTGGTTCTGTAAAACATGGTCATTGGTTCCATTCTAGTGGAAAGGAGCCGGACAAATCCCAGTAACGGGACCGCTTCGTGGGATTCGCCACTAGCCCTAGTTTGACCTAACTGAACTGCATTGATCAGAGCTTTGAAGTCTTTTAATGGCGGTTTCCTTGTCATGGTTGGGGGTGTCATGTGGTGTCCTGCTCTCCTCTCCCCCCACATCTCTCCCATCCCATTACCTGCTTTCTCCCACCAAGGTTACTCTCCACGTAGTTGTTCTGGGTTGGGATGCAACGGGACAAATGCTCTTAGTGGCTGAGCATGGGAGCTGGGGGCTGGGTGAAAGTGTTGGCAGCCAGAGGGCCAGGGTGGTTTTGGGGTGGGGGTGAGCACCTTTGTTTGTCACCTCTGCCGTGGGGCCCTCAGGGTGGAGAGGGAGAACTTTCAGGAACTGGCTTGGAGAAGGCTAGGAGGAAAGCAGAGGGGGCCAGCCTGGGGAAGGAGAGCCGCAGAGATGGGCCTTGGAGCCCATGGGAGTGCTCAGAAGGCTTGCCCAGCACCCTCCCCCCCCCCGCTCCCCACAGATGGGATTCCACTGCTGGCTTTGTGGCAAAAACAGCAACAGTAAGAAGCAGTGGCAGCAGCACATCCAGTCGGAGAAGCACAAAGAGAAGGTCTTCACATCAGATAGCGACTCCACCTGTTGGACCTTCCGCTTCCCCATGGGCGAGTTCCGGCTGTGTGAAAGGTTCCAGAAAAAAAAGGCTTGCCCAGATGGGGAGAAGTGTCGCTGTGCCCACGGGCAGGAGGAGCTGACTGAGTGGCTGGATCGGCGGGAGGTGCTGAAACAGAAGTTGGCCAAGGCTCGCAAGGATATGCTGCTCTGCCCAATGGATGACGACTTTGGCAAATACAACTTCCTATTGCAAGACAACGTATAGGGGGCCCCGGGGACGGGACTCTGGGGGTGGGACTTAGGGAATGAGAAGAAAGATGGGAGGATGAGATGAGATTCCCATGGCCTCCGCAGCAGCATCTTTGGGTCCCCTCTCTGTTAGCCATCCCTGCTGCAGGGGCCAAAGGGCAGGACGTCCTCAGCTCTTTGGCTCCCTGTGACTGGCGTGGCTTTCTTCCTTGTTCTCAGATGGGTGTCAGAAAAGCCAGGCTGGGGAAAGTGGAGGGGATGGAGGAGGACTTGGGAAGTAGATCTCTGGACAAACCTTTTCTCTCCCTGCATCTCTATAGCACGCCCACACACTTGGGGTCCCACTTCATTCCCCGCTGTGTGGGGCAGGAGTACACCCACATACGTGCACATAATCAGAATAATAATGATGACGATGATAACAATAGCTCACATTTCTATAGTGTTTTCTCACAATGACCCTGGGAGGTAGATAGCTCAAGTATTATCACCCCCATCTTACAGATGGGGAAACTGAGATTCAGAGAGGACAAAGTGGCTTGATCACAGTTACATGGCCAGTCAGTGTTTGGAGACAAGAGGGAACACACACACACACACACACACACACACACACTGCTCAGATCAGTTAGACATGTCCCAGGTCATTATTGTCATCCTGTGGTATTAGATAGATCTATTGTCTCCCAGAGTAGGGTGGAACAGGACAGACAGATGCTGTCTGGTGGCTTCAACCCAAAGTTTTGTGTTTTGTTTCCAAAAACCTGTGGTCACTGGCATCAGGGATCCCCCAGGGTTATCCTCTTGGGGGGTTGGCTGAATTGGTTTTTTCCCCGTTACTCTGGGCTCCTGATATCAGCCCTTCTCTCTGGGATGCAGGAGATGGGGACAGGGACACAGTCTTCCCCAGGGCACAGCTGGAGACCTCAAAGAGCACAGATACCCCATTTAGCTTTAAACCCTAAACTTCCTTAATGATCCACATGGTGGAGCTCAGTATTCCCTTCAGTGTCACTCCCCAGCTGCCAAAGGAGACCAGTTCTCTGGTCTTTCCCTCAGCCCTTTGTCCTTTTGGCTGGAGACACCCTTCACACCCTTCTTGTAGCTCTGTTTCCCTTCTGTGCCCTACCTGGGATCCACACAGCTGTGGAACTTATGGGTCTTGCAGGCCCTGATCTTAACCTGACTCTCTTGTCCTGTCTCTCTCCCTTCAGTGATCCAAGAGAAAAGTGGCAGATGAAGCATGGGGCAGGATGGGTGGGCGGTGATCCCAATCCAGGACATGTTAGGGGATCCCTCCAGAGAAAGGACCATCCATGTTGAACCTGGATTCTCCAGGTCCAGTAATCCCTGTACCAGATCCCAAACAGCCCACCAGCACCCCTAGATTATCAGCTGGGCCTGGTTTAGGGAAGTGAGGGAGGAAGGAGTGGAGATGGGAAGCCAGCCGAGGGCAGGCTTGTCCTCAGCAGCTCTTTCTTCCCAGGACATCTCCTTCCTAACCAGGGACAGCTTTGTCCAGAGAGTTCAATTAGATTTCAGTGGATATAGAATGGGAAGATAGACTGAAGTGGGAGGGAAGGGTGCTACTTATACTAAAGCCAATGGAGCTAAAAGTCTGAGTAACTCTTAGGCATGCCCCTTCCATTCCTGCACCTCACCCACAACTCTGATATTGATTAGGAATAGGGACCCTGCTGAGCATAAATCAGGGGGCACTACTCCTGCTACCCTCCTGGGTCACTTCTGACAGAAATAGCTCCGGTTTCTCTAGCTCCGTCAAAATCTATGAAGTGCTCCCAATGAGGGAAGTAGGCCATTTCACAGAGGAGAAAACTGAGGCTCAGAGAGGAGGAGTCACTTTCACCTGATCACACAGCTAGCAAACATGAAAGCTGGAACTCAGACCTGGGACTTTCCACTTTGGGCCCACAGGCACCTCTGCTACCATCCTGGGGACCATGGACTGCAGCACCAGGACCTAGGGAGAATGCTGCTCCCCAAACCCCCTGGGTTCTGAATCCAGCTCTTCCAAACTTGCTGTGGGACCTTAAATGAGTCACTTCACTGCTCTGGGCCTCAGTTTCCTCATCTGTAAACATGGGGGACCAGATGGTTTCTCAGGTCCCTTCCTGCTTTAAATCTATCATTCTGTGGTCCTAAGTAATTTCCCCTTTCTGGCCGTCAGTTTCCTCATCTCTAAAGTGAGAGGGGCTGAAATAGATGACTTCTGAGGTCCCTTCCAGCTCTACATCTATGATCCTATAGTCTGAGGTCCCTTCCCTTTTCTGGACCTCAGTTTCTCATCTGTAAAATAAGGGGCTGAACTAGGTAGCCTTTCACATCCCTTTGAGCTCTAAAACTATGGTCTTTCTGGCCAAGGGGAACTGCCCCCCCCATTGAAGTAGTGCACAAGGTCACCTAGTTTGTACCTACTCCCCTAATTTGACATACCCTGGGTTCAGAATGGATAGGGGGAAGGCTTCACCCAACTGTTCTGGTCCCATCCCCCACCTTAGTCCACCAGACTTCCCACTTCTTCACCTTTTATCTGACACCTCAAAATAGCATTTTTCTGTGCCCACCTCCCCCCAACCTTGTCCACCCCTGCCAGATTCAGGCATTGGTCCCCCAGACATTCCAAAAAGCCCCATACCACTGGATTGGGACCTTAGGCTTGGGGGCAAGGCATGGGGGGAGAGGAGGAAGGGCAGACTTTCTCTTCTTATCTCAAGTCTGTGTGGAAGTGTATGGCTCATGGGATACCTTTGTTCCTTAGGCCTCTCAGAAGGCAATGTTGATGACATAGAAACTGGAGCGGGTAGGGTCTTCCCTCTACCACCCACTCTTGGGACCCCCACCCTTTATTCTATGATCATGGCATGCACCCTGTCCTGTCCCCCTCCTCCCAGTGAACCAGGATGATGGAGCCTGGGGAGGAGAGAGGAATTAAAGGGTATTTGATTTGGGGGTCCACGGGGAGGGATTGCTGTGAACGTAGATACTTGTTTTCCCTGATATGATAACCTAACATGTGGTATTACTGTCTCTTGTCACCAGCCTTGTTTCCCTGTGTATATATGTCGGCCCCGTGATGCATATATACACAGGTATTAAATATATCACTCTATATAATATTATATATGTGTGTGGTATCCAAGGAATTGCTTGTAAGGAAGGTGAAGGATAAAGGATTTGGCTGGGAAATGCAGCAGGAGCCCTGGGTTTGACTTGGCTTCCCTAGCTGGGAGCAGGGTGAGTGGGGGCTGCTGCCCTTCACCTCAGATCAGCAAGGTGCCTGATGGTATATAGGAAGGTAGCATTGGAGGACATTGAACAAGAGAGACGTGGGTGGCGATGCCTGGCTCTGTCCAGCGTCTTCCTTCCCCACCCAGCTTGACGGAGGCAGAAAGAGCGGGCTCAGTGGAGAAACTGTGTTCTTAGCCAGAGGGGCAGGGCCCAGGTTAACTAGGGCTGCCAAACTTCCAAAACCTGTAGGTCCAACTCTGCCTGTCCAATCCAGAGAAGCTGTCCATGGGAGCTGTAGGGAGGGGCCCAGGTTGGGAGGCAGAGGATTCGAATTTGAATGCTGTCTCAACCATTTCTTATACGTGGCTAATAGCACAACCTCCCTGGGCCACTTTTCTTGGCTATTAGATGGAGGCTTGGCCTAGATGGTCTCTGAGGTGTCTCCCAGCTCTAGACCTACAATCGTGCATGGAGAAGAAAAGAAAATGGAGCTGAAGGAGGCCATGTCCTCCTGGGGCCAGGGAGGCCTCTTCCCTAGGGCGGGCTTCTGTGCCCCCCTGGAAAGCAGGTGGATGCACATCCGAGAGAAGAGATTGGAGAAGAAGGGGCTCTAAAGGGAGAAGAATAAGCGGGTAATTTGGAGGATGATGGAAAGGACTGCAGTGGTCTTCAGATTTAGACTCAAAATCCAAGCTCAGAGTTGGGACCAAAGCTGCCAGGCTGCTCATGGACAGGCGAGGATGAGACCAAGGGGAGACGCATGTATACATATATGTGTGTACATATAACTGCACGCATATATGTATATGTACATGCACACGTGTGTGTGGTCACACCCCCTCCCCCCTGCACGGACTGTGGGGGGAACGTGTCAGAAATGAAGGAAGAAGGGGGGGAATGTTGCATTTCACAGCCAGGTAATGATGGGGTGAATAGTCCTTTAAAATGTCTGTGTATTAAACATTTTAAGAATACCACACTTTAATATTAAATATTCATAAGGCCCTAGTATCTGGATAATATGGTAGATGTTTTAATAACAATTTTTTGGTCCTTCTTAAAAATAAAACAGACAAAACTTGCATCTTTTGGCCTTTGTACTAGAAAATAAAAGTGCACTTGAACCCTG

>UoN.Pci.T.32974_UoN.Pci.T.32974.1

TAAAATAGCAACACGATGACATTTATTGGCTGATGTCACTGTCTGCATTAGATCTAATGATCTGAAACAGTATTGTCAATAGGTGAAAAAGATACATACGTCACGGATGCTTTAGCTTGAAAATCATGATTCCCACCAATCTGGTTTTGAGAAATGATGGTTGGTTGGTTGGTTTGTTTTTTTCCTGTACAGCATGCCTAGCACAATTAATACAATAAAAAGAATTTACAGTTTGACTCATATGGGAACTGTATCCATTAAAGATGTTTGGCTCAGTTGTCTGCAATTTTATTTTTGGGAAGCCATGATAAAATTTCCCTGCTCCAAAGGGATCAGTTCTTTGGGGATCTCAACTTGAAATATATCCTTTCCGCCCTTAGCAAGTATTGGTTTTAACAGCCATCTCGGGTTTGAAAGACTTGTCAGGTACTTTTGTTTCAAATCAGTCAGCACGGCTTTGTTTTCCAGGTCCACCAACTTCTCCAGAGTCAGATTTCTAGGACACTGCAATGTTTCCCCAATATCCACAAGCCCAGCAATAACTCCTCGACCAAATTTCTCCCCTTCGTCCAATAGCGCCTGAAGTTGAGTGCTTGTCATCCCAAGCCTCTCCACCAGGATATACTTCCAGGATTGTTCTTCCCAGTCTTTTTGAGCGATGTGAATAGCAATGGTGCAGCTGTGCTGGTTTGCCAGCAAAGGACGCCAACGAGTCTCTAGGGTTTTGACTCCATTTAGGACCAGCCCAGCATAAGGCTGCCGGAAGGAAAGACAACCAAATTTCATCTTTCTCAAGCCTCTGATTCATTTATCATTTCACCCGGGAGGGCGGCGGGCGGCGGGCAGCGGGGCTTGCTTACAGAGAGGGAGTGCAGGAGAGCAGAGCGGAGCCTCTCGGCGGTAGGGCAGCGGCGAGCCGCGGGGAGCAGTGGGCCGCCCGGCCGGGCGGTGAAAGGAGGAGGAAGAGGAGGAGGAGGAGGAGCTTGGGGA

>UoN.Pci.T.32974_UoN.Pci.T.32974.2

TAAAATAGCAACACGATGACATTTATTGGCTGATGTCACTGTCTGCATTAGATCTAATGATCTGAAACAGTATTGTCAATAGGTGAAAAAGATACATACGTCACGGATGCTTTAGCTTGAAAATCATGATTCCCACCAATCTGGTTTTGAGAAATGATGGTTGGTTGGTTGGTTTGTTTTTTTCCTGTACAGCATGCCTAGCACAATTAATACAATAAAAAGAATTTACAGTTTGACTCATATGGGAACTGTATCCATTAAAGATGTTTGGCTCAGTTGTCTGCAATTTTATTTTTGGGAAGCCATGATAAAATTTCCCTGCTCCAAAGGGATCAGTTCTTTGGGGATCTCAACTTGAAATATATCCTTTCCGCCCTTAGCAAGTATTGGTTTTAACAGCCATCTCGGGTTTGAAAGACTTGTCAGGTACTTTTGTTTCAAATCAGTCAGCACGGCTTTGTTTTCCAGGTCCACCAACTTCTCCAGAGTCAGATTTCTAGGACACTGCAATGTTTCCCCAATATCCACAAGCCCAGCAATAACTCCTCGACCAAATTTCTCCCCTTCGTCCAATAGCGCCTGAAGTTGAGTGCTTGTCATCCCAAGCCTCTCCACCAGGATATACTTCCAGGATTGTTCTTCCCAGTCTTTTTGAGCGATGTGAATAGCAATGGTGCAGCTGTGCTGGTTTGCCAGCAAAGGACGCCAACGAGTCTCTAGGGTTTTGACTCCATTTAGGACCAGCCCAGCATAAGGCTGCCGGAAGGAAAGACAACCAAATTTCATCTTTCTCAAGCCTCTGATTCATTTATCATTTCACCCGGGAGGGCGGCGGGCGGCGGGCAGCGGGGCTTGCTTACAGAGAGGGAGTGCAGGAGAGCAGAGCGGAGCCTCTCGGCGGTAGGGCAGCGGCGAGCCGCGGGGAGCAGTGGGCCGCCCGGCCGGGCGG

>UoN.Pci.T.33201_UoN.Pci.T.33201.1

CAGTAAGAATCTTTATCTCCTCCTCATATTTGTCTTCTTTCTGAGAATACTGCAAGACAATTAAAGTAATAATGGTTAGACCAAATTATCCTCATTGACACTAACCCACCCCCCCAAGAGCCAATGGAAATTAATGCACTTAATGCCTCATGAGGAGAGGGGGAGTGGGTAAGGGGAGTTCTTTTAAAATTCATCTCATGTTCTAATTATCCACAGGCCTGATGTTCCAGGGCCCTTTTGATGGCAAAACTGCATTTCTTGATTGTCCTAAACTTTAAGAAGCTACCTTCTTTCCCTATTCACAAGGAAAGCCAGGGTCAAGATAACCCTTAATACACAGTCATTCACACTGATGGCAAGTGAAAAAATTAGTCCCCTGTACCTCTCACTAGACCTCAAATATGATACTACCTGCAAACCTATTTAGGTCAATGATAACTGCCTGTTTATTAAAAATTACAGGCCTGCCCTTTTCATACCCAGAAAGCTATTTTAAACTTTAAAGCTCAGAGGGCATGAAGAATCAGTATCAGCCACCATGATTTGAACAGAGCAGTGGCCAGGAAAAAAAAAAAAAAGGAGGGTTTCTCGTGCTGGATGCACATGGTAGGGCTGGGCCCAGGCCCCAAGCCTGTCCCTTTCACGAACCAGCCCCTACCTTCTCCGCCTGAGCCTCAAGAGATTTGAGGTTGTTGGTGACATTCTTCAGCTCCTCCTCCAGCTCAGAACACTTACTGTGGATGTTTTAAAGAAGGCAGGAGAAGAAAAGGGAAGAAGAAAACAAAGAGGAGAGAGACACACAGATGTGAATCTATAGGTAGAGAGGGTTGAGAAGTCACCAAGGGAGAGAAGTACTAAGACAACTCTGAACAGCCCCATTCTTTCCACCCCCACACTACTAGCAGCACAGGGGAAGATTAACCCCTTCTAGGTTGGAACCCAGTATGCTGAAAGACTAATGATGTAAGAGAAGAACCCCTAAAAGACATGCTCCTGATGGGACATTTTTCAGGAACAGGGCACAGCATTGCCCAGGGTGGAGATGGGAGCTGCTGCCCAGCCAACCATCCCTAATCCCCCAAGAGAAGGACAGGCCCTGTGGAAAGGCAGTAAAGCAGCTAGGAAGGAAGAAGTGAAGGATGCCAAGTCAGTGGGAAAGAATGGAGCACTCCATGAGAGAGATGAGAAGACTAGTCAAACAAACCAGTGGAGGGGAGGCAGCTGCAAAACAAAATCAAACCACAAACACAAACACGCACACACGCGCACACCCTTTCAGCAGGTGCAACAGTTAGTACCTTTTCTTCAGCAGCATTCAGACACTTCAGGTTCTGGTCCATCAGTCTGATCTGCTCATCCATCTCCCGGCAACGGCTGTTAGTGTAGTCACAGGGGAAGGAGTGGAGGGGTGGGATGGCACAAGCCAGATTGTGGGGAGGGAGAAAGGTCAAAAAGAAGCACAGCAAGAATACAAGCAGCAGAGCAAAAGAAAAAAAGGGAAGGAGAAAAAACAAACCACCATGGTCATGACATGCTGTGTCTGGGATACAGGTAGGGGGATGGGTGGGAAGCATAAGTATGCTCATGTTAGGGGTGACTTTGTTACACAAGGCCCAGAAGATGCTAAGATAGAGCAGTAAGTGTCTGTCCTTTTCCTAATTAACAAATATGCCAAGTAACAATGGCAGCCTCTGTACTCCTCTCCCTCCATGCCACCTAGGACTTGGGGAGGAGAGGAAGACATACAAGAGCAGAACAGTGGCCCCATTTTGAGGCTAGGATGGCAGAGACTCTAGGTTACACGTTTTCCCCACCCATCACTGAGTAACTTCTGAATATTCTACACAAAAGCCATTCCACAGCCAAGTCTTCTACAATCTTCTCTTTGAAGCCCATTTTATTCTTCAAGTAAGCGCCAGACTTCTCCAACCCCAAAGGCCTTGTTTAACAAGATTGAAGGAATGCTGGTTTTTTCCACATTAGTGACCATAACAAAGGAAAGGTGACCAATGGTTGTAACAAGGTTTTGGAAGTTAGATACTCACGACTCAGCCAGCTCGGCCCGCTCCTCTGTGCGCTCCAAGTCCCCCTCAATAATCACCAA

>UoN.Pci.T.33201_UoN.Pci.T.33201.2

CAGTAAGAATCTTTATCTCCTCCTCATATTTGTCTTCTTTCTGAGAATACTGCAAGACAATTAAAGTAATAATGGTTAGACCAAATTATCCTCATTGACACTAACCCACCCCCCCAAGAGCCAATGGAAATTAATGCACTTAATGCCTCATGAGGAGAGGGGGAGTGGGTAAGGGGAGTTCTTTTAAAATTCATCTCATGTTCTAATTATCCACAGGCCTGATGTTCCAGGGCCCTTTTGATGGCAAAACTGCATTTCTTGATTGTCCTAAACTTTAAGAAGCTACCTTCTTTCCCTATTCACAAGGAAAGCCAGGGTCAAGATAACCCTTAATACACAGTCATTCACACTGATGGCAAGTGAAAAAATTAGTCCCCTGTACCTCTCACTAGACCTCAAATATGATACTACCTGCAAACCTATTTAGGTCAATGATAACTGCCTGTTTATTAAAAATTACAGGCCTGCCCTTTTCATACCCAGAAAGCTATTTTAAACTTTAAAGCTCAGAGGGCATGAAGAATCAGTATCAGCCACCATGATTTGAACAGAGCAGTGGCCAGGAAAAAAAAAAAAAAGGAGGGTTTCTCGTGCTGGATGCACATGGTAGGGCTGGGCCCAGGCCCCAAGCCTGTCCCTTTCACGAACCAGCCCCTACCTTCTCCGCCTGAGCCTCAAGAGATTTGAGGTTGTTGGTGACATTCTTCAGCTCCTCCTCCAGCTCAGAACACTTACTGTGGATGTTTTAAAGAAGGCAGGAGAAGAAAAGGGAAGAAGAAAACAAAGAGGAGAGAGACACACAGATGTGAATCTATAGGTAGAGAGGGTTGAGAAGTCACCAAGGGAGAGAAGTACTAAGACAACTCTGAACAGCCCCATTCTTTCCACCCCCACACTACTAGCAGCACAGGGGAAGATTAACCCCTTCTAGGTTGGAACCCAGTATGCTGAAAGACTAATGATGTAAGAGAAGAACCCCTAAAAGACATGCTCCTGATGGGACATTTTTCAGGAACAGGGCACAGCATTGCCCAGGGTGGAGATGGGAGCTGCTGCCCAGCCAACCATCCCTAATCCCCCAAGAGAAGGACAGGCCCTGTGGAAAGGCAGTAAAGCAGCTAGGAAGGAAGAAGTGAAGGATGCCAAGTCAGTGGGAAAGAATGGAGCACTCCATGAGAGAGATGAGAAGACTAGTCAAACAAACCAGTGGAGGGGAGGCAGCTGCAAAACAAAATCAAACCACAAACACAAACACGCACACACGCGCACACCCTTTCAGCAGGTGCAACAGTTAGTACCTTTTCTTCAGCAGCATTCAGACACTTCAGGTTCTGGTCCATCAGTCTGATCTGCTCATCCATCTCCCGGCAACGGCTGTTAGTGTAGTCACAGGGGAAGGAGTGGAGGGGTGGGATGGCACAAGCCAGATTGTGGGGAGGGAGAAAGGTCAAAAAGAAGCACAGCAAGAATACAAGCAGCAGAGCAAAAGAAAAAAAGGGAAGGAGAAAAAACAAACCACCATGGTCATGACATGCTGTGTCTGGGATACAGGTAGGGGGATGGGTGGGAAGCATAAGTATGCTCATGTTAGGGGTGACTTTGTTACACAAGGCCCAGAAGATGCTAAGATAGAGCAGTAAGTGTCTGTCCTTTTCCTAATTAACAAATATGCCAAGTAACAATGGCAGCCTCTGTACTCCTCTCCCTCCATGCCACCTAGGACTTGGGGAGGAGAGGAAGACATACAAGAGCAGAACAGTGGCCCCATTTTGAGGCTAGGATGGCAGAGACTCTAGGTTACACGTTTTCCCCACCCATCACTGAGTAACTTCTGAATATTCTACACAAAAGCCATTCCACAGCCAAGTCTTCTACAATCTTCTCTTTGAAGCCCATTTTATTCTTCAAGTAAGCGCCAGACTTCTCCAACCCCAAAGGCCTTGTTTAACAAGATTGAAGGAATGCTGGTTTTTTCCACATTAGTGACCATAACAAAGGAAAGGTGACCAATGGTTGTAACAAGGTTTTGGAAGTTAGATACTCACGACTCAGCCAGCTCGGCCCGCTCCTCTGTGCGCTCCAAGTCCCCCTCAATAATCACCAA

>UoN.Pci.T.33493_UoN.Pci.T.33493.1

GAATCAGACAGCAGCTTTTACATCAGTTAAAATTTATCATCAGCATCGTCATACATCATTTGCATAGATTGGTTTCTAACATCCATGTAATATGACACCAGTTGTAACACCATGCACGTTATTCATCCTTTGTTCTCAAAGAGGACTGATGACATCATGAGGGTGATGTCTTAATTGTGAATTGGATTTCAGTGAGGCAGAGCTGTATAGGCACCATACATATATAAAATACAGTGGAAGGGCTAGTCACTAGGAGGAGGCGGCTGCCAGAGCCTCCCTAGTGCTGTAGCCCTTCATATCAAACACTCCATTGTCTAGTTGATAAAGGTGAATAAATGAAAAATACTATTCCCAAAATAACAAAGGCAGGGGGGAGAAAGATTCCCGTATGCAAAGGATTCCAAACACCATCATTAGGCAAGGGAGAAGACAGCCAAGCTGTAAAAACACCAGAAGAATGCAAAGCTACTGAAGGAGAGTCTTTTCCCCTCCAGTTTCCAAATGCAAAGAAACACTTGGCATGAAATGTCAGTGAGCTTCGACTTTATAAAACTTGCATTCTCATTGGTCTTGCTCTGCTGCCTTTACTCCCCACCCCCTCACTTCTGCCCAAAACCCACGGAGTCACTGAAAGCCAGGAAATATCAAATAGTTTTAGAGAGAACTGCTGTTACCTGGTCAGCATTATTTTACTCTTTTGAATTTTACTAGTTAGTCTGGGATTTGGCTAAAGTACCATCTGACCTTCTTTTCTTGGTATGTTTGCTGTGGAAATACAACACTTATTTTAGAATTGTGTGGATGATTGTCTTTGAAAATGTCTAATAAGCTGGACTAGTCAAAATACATCACAAGAGCTACTATTAACTATTGTGAGGTAAGTGCTAGAGATTTTCATCAAGTCCCAAACCCCCTGTCTGCATCAAAGTGAACCAAAATCAGTGTTAGGAGTCTAGATTTCTGGCTGCATATACCAATGGGGTACAAGACTTTTAATCACAAGGTGGCTACTGTTAGTGTTTGCATATTCCTTATGGACAATCTTGTGTTCTGAGGAAAGGCTATAATGTAGATGGTTTGTGACAGAGGTAAGTGACTAGTACAGACTAACTAACACAAAGGGCTACTTTGTGCCCTGGTTTTAATATCTATCTTAAGGCTATACGCTCACTGGAGGTTTGGATATTAATTGTTTATTTCTCTATTTTTACTCTGACTCTACTATTGGATGAGTTTTGTGAACTCTCTCTGGCCCCACAGTTCTTTTACTTTTCCATGTTGTACTGATTTTAAGAGGAATTATTCAATAGGACATAACCAAGACAGTGTACTCATTATCTTTTATTTCATGATTATTAAAGATAAAGCTGAACCCAAAGCTATCATTTTAGTTGGTGCCCACTGAAATTATACGACTTCATTTGTTTATAATTCTGAGTTATATATTTGATTCACCTTTCTATACTGTATATTATGACCATGATTTTAGGAACCTGATAGGAATTTGACCCAGCAGTGGATCACCTCTAGAACAAGAAGTTCTAGGACACAAAACCACTTTCCTGAGAATCGTGACTAGTATCAGTGCTTGTTAATGTTAGAACAAAAACAAAATTTTAAGTTGAAGTATTCTCAATTTCCAAAAAAACCCTATAGTTACTAAAATAAGGTTTTTAGAATGTTAGGCCATTTACAAGATTAATCTTCTCCCAGCACTCAGTTCATCTGTGAGGTGAGTGGGATGGCCTCTCAGGTCCCTAAATCTGTGATCCTATGCAAAGACAGCCTGGATGTCTAGCAGATGGAAAGAAAATATGAAGAACAGGAGAAAGGACTTTAAAGAACATCCTTCAGCCTGTCATTTAAATATCTGGGGTCTTTTCCAGCTTCCAAACTACAAATCTGATTTTAAACTCATTCCATCCCAAGTCAAAATCTGGTAAAAGCCTTTAGAAGCTAGAAGATTACACCAGAATTTTTTTTATCTCATAATGTTTGGAGTCACAAGATGGAGACTGAAGAAAATAGTGGACTAGAGTAATGTACTCTGAAGTTCTTCCATAGGATTTGGGGTGAACTGAATGCCCTTTTAATATTGATCTGGCTATGAAGTTATAAGATACTAGATCATTTGGAAATGTGAAAGATAATAGGAGAAGTAACATTTCCAGAAGGAAAAAAATTTTTTCTGGTTTATAATTTTAGAAATGAAAATAAAATGTTCTGGCAGGATAGGAGGTGTTGAAAGAGAAGCAAAATTGGTGTGTGTTTCGGCACATCATATAAGATTCCATTTTCCAAGTACCTGGAAGTTACTGTAACACTCCAATTTGTTGAAGCTTTTGTAGCAACTGGCCATTCTGTTTCAGGGTTGTGAGATCCGAGTTTCCACCAATACACTCTTTACCAATAAATATCCGAGGCACGGTTCTTGCCCCTGTTAGCTGTTGCAAATAATCTTGGATCGCATTTGTATCACTGTGGGCTGTTATGTCGACAAATTCCAAGGACTCCGGTTTAATAGGAAGCTGCTTGAGAAGCTCCATAGTTTTTCTGCAGTGAGGGCAGGTGGGTTTGATGAAGACCACCACCTTATCAGCTTTGATCTTGCTATTCACGAACTGCTGGGCCATCTTTACCACATTTGTGACCTCCTAAAGAGTAAATACCTAAGCTGGGGCAATGCTTGGGGCTTTATTTAACTGTCCTGCTGCT

>UoN.Pci.T.33766_UoN.Pci.T.33766.1
[truncated: 649,250 more chars]
